# Genetically Predicted Causality of 28 Gut Microbiome Families and Type 2 Diabetes Mellitus Risk

**DOI:** 10.3389/fendo.2022.780133

**Published:** 2022-02-03

**Authors:** Kun Xiang, Jing-Jing Zhang, Yuan-Yuan Xu, Xing Zhong, Jing Ni, Hai-Feng Pan

**Affiliations:** ^1^ Department of Epidemiology and Biostatistics, School of Public Health, Anhui Medical University, Hefei, China; ^2^ Inflammation and Immune Mediated Diseases Laboratory of Anhui Province, Hefei, China; ^3^ Department of Nutrition and Food Hygiene, School of Public Health, Anhui Medical University, Hefei, China; ^4^ Department of Outpatient Wound Care Center, 901 Hospital of Joint Logistics Support Force of People Liberation Army, Hefei, China; ^5^ Department of Endocrinology, The Second Affiliated Hospital of Anhui Medical University, Hefei, China

**Keywords:** causality, gut microbiome, mechanism, Mendelian randomization, type 2 diabetes mellitus

## Abstract

Mounting evidence indicates that gut microbiome may be involved in the pathogenesis of type 2 diabetes mellitus (T2DM). However, there is no consensus on whether there is a causal link between gut microbiome and T2DM risk. In the present study, the Mendelian randomization (MR) analysis was performed to investigate whether gut microbiome was causally linked to T2DM risk. The single nucleotide polymorphisms (SNPs) that were significantly related to exposure from published available genome-wide association study (GWAS) were selected as instrumental variables (IVs). The robust methods including inverse variance weighting (IVW), MR Egger, and weighted median were conducted to infer the causal links. Mendelian randomization pleiotropy residual sum and outlier (MR-PRESSO) and MR-Egger regression were used to test whether there was horizontal pleiotropy and identify outlier SNPs. The estimates of IVW suggested that *Streptococcaceae* (odds ratio (OR) = 1.17, 95% confidence interval (CI), 1.04–1.31, *p* = 0.009) was associated with higher risk of T2DM in European population. In Asian population, the MR IVW estimates revealed that there was a causal link between *Acidaminococcaceae* and T2DM risk (OR = 1.17, 95% CI, 1.04–1.31, *p* = 0.008). There was no evidence of notable heterogeneity and horizontal pleiotropy. However, after false discovery rate (FDR) correction, the causal link between gut microbiome and T2DM was absent (FDR, *p* > 0.05). In summary, using genetic instruments, this study does not find evidence of association between the 28 gut microbiome families and T2DM risk. However, *Streptococcaceae* and *Acidaminococcaceae* may have a borderline positive correlation with T2DM risk.

## 1 Introduction

Type 2 diabetes mellitus (T2DM) is a metabolic disorder characterized by insulin resistance and β-cell dysfunction, which occurs mostly in middle-aged or elderly individuals (1). As a major component of the global disease burden, the prevalence of T2DM is increasing (2). It is estimated that by 2040, there will be 642 million adults worldwide with diabetes, and most of which are T2DM (3). The development of T2DM is mainly triggered by genetic factors and unhealthy lifestyle. Obesity is the primary predictor of T2DM which is responsible for more than half of diabetes cases (4). T2DM is a lifelong disease, and there is no cure. Mounting evidence suggests that gut microbiome may be involved in the pathogenesis of T2DM (5, 6).

The gut microbiome is a complex microbial community composed of a variety of bacteria living in the intestine. Recently, there has been considerable interest in the roles of the gut microbiome in regulating host physiological activities. Studies demonstrated that the gut microbiome possessed the properties that drove the development and maturation of the immune system (7) and maintained the homeostasis (8). Also, previous evidence showed that gut microbiome contributed to the development of numerous diseases by regulating cell differentiation (9), affecting the release of cytokines (10), and regulating drug absorption and metabolism (11). Concerning T2DM, gut microbiome dysbiosis is a clinical manifestation of the chronic disease (12, 13). The reason for the involvement of gut microbiome in the pathogenesis of T2DM may be that gut microbiome dysbiosis results in increased membrane transport of sugars and decreased branched-chain amino acid transport and butyrate biosynthesis, which lead to an unbalanced oxidative stress response (14). However, no consensus is reached on whether there is a causal link between the gut microbiome composition and T2DM risk.

Mendelian randomization (MR) is a commonly used approach to uncover the causal link between exposure and outcome (15), in which the genetic variations that are significantly related to exposure serve as instrumental variables (IVs). Being different from traditional observational studies, MR approach can minimize the influence of the confounding factors and reverse causation on the outcome (16). In the present study, the large-scale genome-wide association study (GWAS) summary-level data were used to perform two-sample MR analysis to infer the causality of gut microbiome composition and T2DM risk.

## 2 Materials and Methods

### 2.1 Data Sources and Instrumental Variable Selection

The single nucleotide polymorphisms (SNPs) that served as IVs were from the latest GWAS, involving 18,473 subjects, which explore the influence of host genetics on the gut microbiome composition (17). The corresponding summary-level genetic data of T2DM risk were derived from a large GWAS involving 77,418 T2DM cases and 356,122 healthy controls of East Asian individuals (18). For the analysis of the European ancestry, the data of T2DM were obtained from a meta-analysis of GWAS with 62,892 T2DM cases and 596,424 controls (19). The corresponding information of SNPs was abstracted, including effect allele, other allele, effect size, standard error, and *p*-value.

The steps for selecting optimal IVs were as follows. First, SNPs with a *p*-value less than the locus-wide significance level (1 × 10^−5^) were selected. Second, the genetic variations would be excluded if the minor allele frequency (MAF) is less than 0.01 (20). Third, in order to avoid the impact of linkage disequilibrium (LD) between the variables of interest on the results, the clumping process was performed, in which *R* ^2^ < 0.001 and clumping distance = 10,000 kb. Fourth, the corresponding data of the above selected SNPs were extracted from the outcome GWAS. When selected SNPs were absent from the outcome GWAS, the proxy SNPs with high LD (*r* ^2^ > 0.90) would be chosen to substitute the variants of interest. Fifth, in harmonizing process, the palindromic SNPs were excluded to ensure the effects of SNPs on exposure correspond to the same allele as the effects on the outcome. These stringent selected steps were conducive to ensure the authenticity of the results.

These selected IVs must meet the following three core assumptions. Firstly, IVs are significantly correlated with exposure which means that the variants of interest can predict exposure effectively. In the present study, *F* statistic was performed to confirm whether the estimates were affected by weak IVs. *F* statistic is expressed as *R* ^2^(*n*-*k*-1)/*k*(1-*R* ^2^) where *R* ^2^ represents the estimated variance of exposure explained by the selected IVs, *k* is the number of IVs, and *n* refers to the sample size. Secondly, the IVs have to be independent of the outcome, namely the IVs can only affect outcome through exposure. Herein, MR-Egger regression and Mendelian randomization pleiotropy residual sum and outlier (MR-PRESSO) were used to confirm whether there was horizontal pleiotropy between IVs and outcome. Thirdly, the IVs must be independent of the confounding factors associated with exposure or outcome.

### 2.2 Statistical Analysis

The GWAS summary-level data were merged to infer the causal link between gut microbiome composition and T2DM risk. In the present study, the robust methods including inverse variance weighting (IVW), MR Egger, and weighted median were conducted to infer the causal links. IVW is a traditional method that merges the Wald ratio estimates of each IV in a meta-analysis manner (21). IVW equates to implement a weighted linear regression of the associations of the IVs with the outcome on the IVs with the exposure and intercept is constrained to zero (16). In the absence of horizontal pleiotropy, IVW enables to obtain unbiased estimates (22). MR Egger takes into account the pleiotropic effects, and the causal estimates represent the dose-response relationship between the genotype and outcome (23). When the Instrument Strength Independent of Direct Effect (InSIDE) hypothesis holds, MR Egger can get consistent causal effect estimates. Weighted median method allows some genetic variants are invalid, but only if at least half of them are valid instruments (24).

The MR-Egger regression and MR-PRESSO were used to confirm whether there was horizontal pleiotropy. MR-Egger regression has the property that confirms the pleiotropy between genetic instruments and outcome, and *p*-value greater than 0.05 was regarded as no horizontal pleiotropy. However, MR-Egger regression has lower precision and statistical power. MR-PRESSO can detect horizontal pleiotropy and identify pleiotropic outliers (25). If there was horizontal pleiotropy, the analyses were repeated after removing these pleiotropic SNPs. Heterogeneity between genetic instruments was quantified by Cochran *Q* statistic. Leave-one-out sensitivity analysis was used to test whether the overall estimates were affected by strongly influencing SNPs. In addition, the Benjamini-Hochberg method was used to correct the false-discovery rate (FDR) for multiple tests. The statistical analyses were conducted by TwoSampleMR (26) and MRPRESSO (25) packages in R (version 4.0.3).

## 3 Results

### 3.1 The Selection of Instrumental Variables

Initially, SNPs that were significantly related to the 28 gut microbiome families were selected. When excluding SNPs that with LD and were absent in the outcome GWAS, the remained variables of interest were selected as potential IVs. The detailed information of the selected IVs is shown in **Supplementary Tables 1**, **2**.

### 3.2 The Estimates of Gut Microbiome With T2DM

#### 3.2.1 European

**Figure 1:**
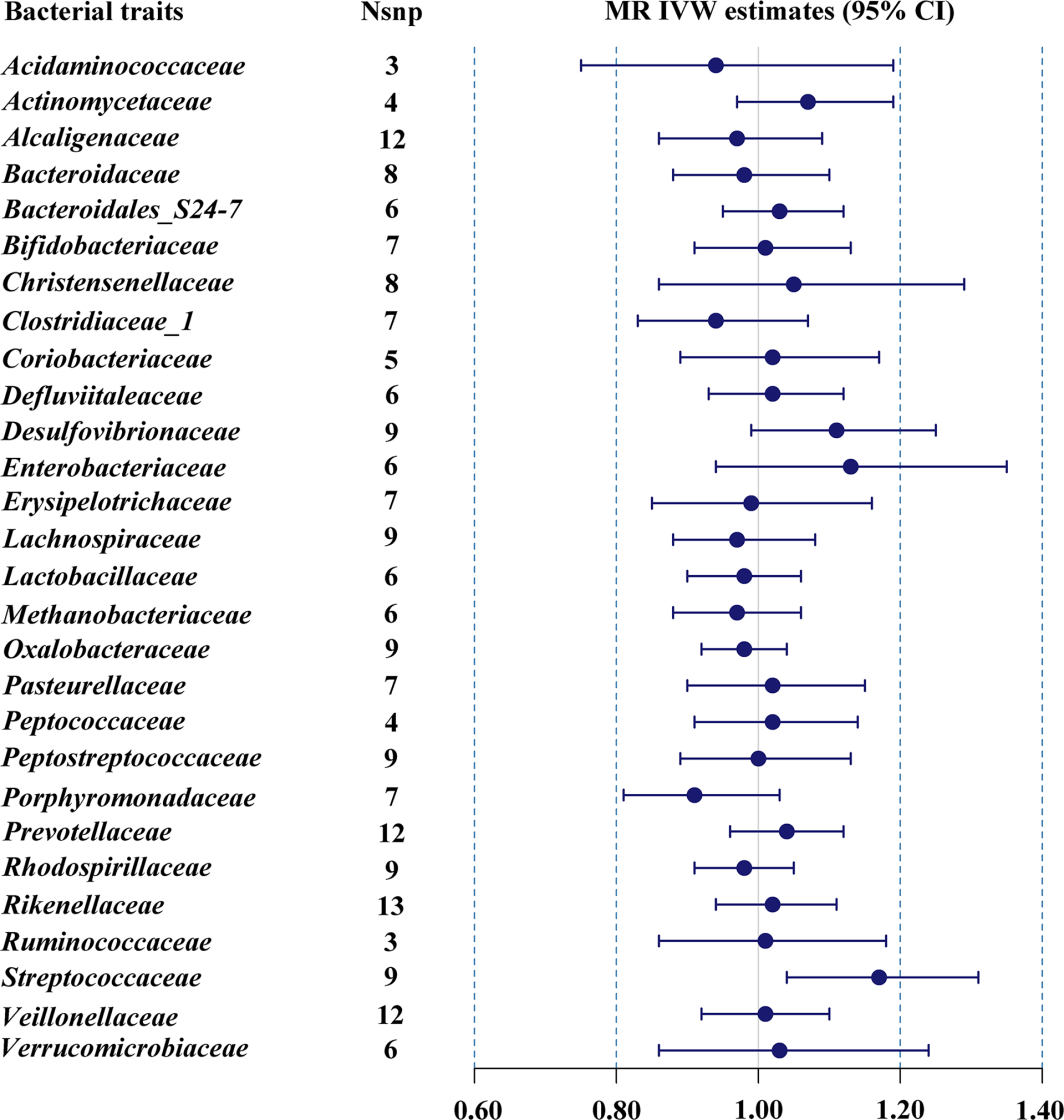
MR IVW estimates of genetic instruments for gut microbiome and T2DM among individuals of European descent.

The estimates of IVW indicated that genetically predicted *Streptococcaceae* (odds ratio (OR) = 1.17, 95% confidence interval (CI), 1.04–1.31, *p* = 0.009) was positively related to T2DM risk (**Table 1**; **Figure 1**). However, MR Egger and weighted median found no evidence of the association between exposure and outcome. The *Q* statistic showed that there was no notable heterogeneity (*p* = 0.270). MR-Egger regression and MR-PRESSO analysis further suggested no horizontal pleiotropy (*p* = 0.492 and *p* = 0.331, respectively). The results of MR-PRESSO analyses found evidence for significant horizontal pleiotropy between the IVs of *Christensenellaceae* (*p* = 0.004), *Enterobacteriaceae* (*p* = 0.041), *Methanobacteriaceae* (*p* = 0.046), *Peptostreptococcaceae* (*p* = 0.043), and *Verrucomicrobiaceae* (*p* = 0.020) and outcome. The causal effect estimates were recalculated after removing the outlier SNPs, and the results did not change substantially, except for *Methanobacteriaceae* (OR = 0.93, 95% CI, 0.88–0.99, *p* = 0.029). Leave-one-out sensitivity analysis showed that there were two strongly influencing SNPs (rs17791387, rs186073) in the IVs of *Desulfovibrionaceae* and one strongly influencing SNP (rs11123059) in the IVs of *Methanobacteriaceae* (**Supplementary Figure 1**). After removing the strongly influencing SNPs, the results changed significantly (*Desulfovibrionaceae*: OR = 1.18, 95% CI, 1.07–1.30, *p* = 0.001; *Methanobacteriaceae*: OR = 0.93, 95% CI, 0.88–0.99, *p* = 0.029). The detailed results are shown in **Supplementary Table 3**.

**Table 1:** MR estimates of IVs for gut microbiome and T2DM.

Ethnicity	Bacterial traits	Nsnp	Methods	Beta	SE	OR (95% CI)	*p*-value	FDR *p*-value
European	*Streptococcaceae*	9	IVW	0.15	0.06	1.17 (1.04–1.31)	0.009	0.962
			MR Egger	−0.02	0.24	0.98 (0.61–1.58)	0.948	0.965
			Weighted median	0.14	0.08	1.15 (0.99–1.34)	0.071	0.663
Asian	*Acidaminococcaceae*	3	IVW	0.16	0.06	1.17 (1.04–1.31)	0.008	0.224
			MR Egger	0.25	0.14	1.28 (0.98–1.67)	0.322	0.939
			Weighted median	0.15	0.08	1.16 (0.99–1.35)	0.051	0.607

MR, Mendelian randomization; SNP, single nucleotide polymorphism; IVW, inverse variance weighted; IVs, instrumental variables; T2DM, type 2 diabetes mellitus; FDR, false-discovery rate; OR, odds ratio.

#### 3.2.2 Asian

**Figure 2:**
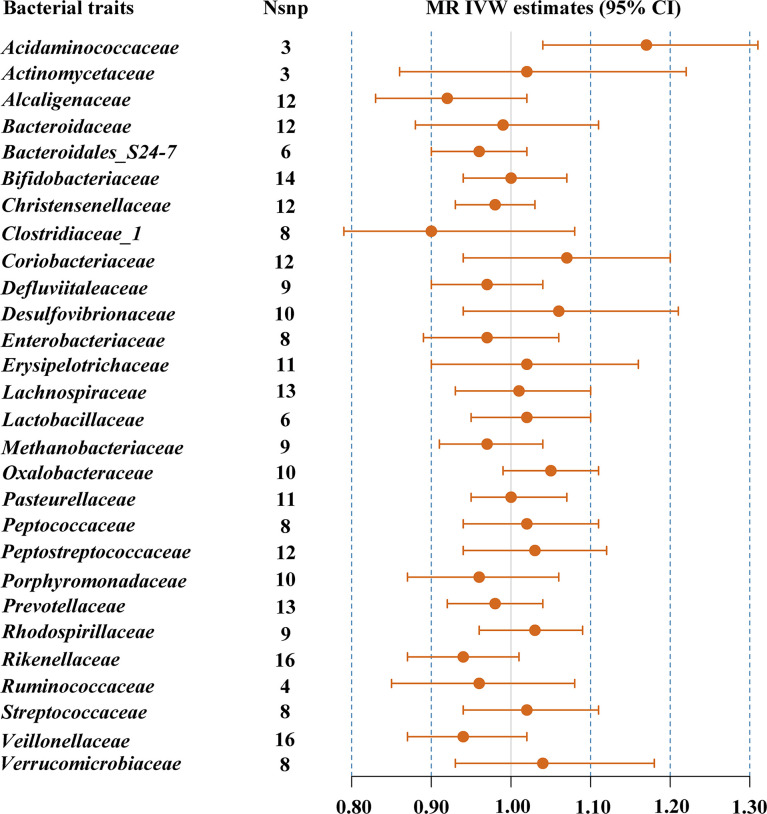
MR IVW estimates of genetic instruments for gut microbiome and T2DM among individuals of Asian descent.

The results of IVW indicated that *Acidaminococcaceae* (OR = 1.17, 95% CI, 1.04–1.31, *p* = 0.008) was the risk factor for T2DM (**Table 1**; **Figure 2**). There was no evidence of notable heterogeneity (*p* = 0.751) across instrument SNP effects. MR-Egger regression showed that there was no horizontal pleiotropy between the variants of interest and outcome (*p* = 0.593). However, there were not enough SNPs for MR-PRESSO analysis. The results of MR-PRESSO suggested that there was significant horizontal pleiotropy of *Bacteroidaceae* (*p* = 0.014) and rs234027 was a pleiotropic SNP. In addition, MR-PRESSO detected two outliers (rs6060237 and rs7199026) in the analysis of *Desulfovibrionaceae*. After removal of these outliers, MR estimates remained null. In the sensitivity analysis, strongly influencing SNPs were identified in the IVs of *Acidaminococcaceae* (rs6589457), *Alcaligenaceae* (rs7638039), *Clostridiaceae_1* (rs11752225 and rs6934446), *Oxalobacteraceae* (rs36018452), *Rikenellaceae* (rs2290844, rs4264350 and rs7832304), and *Verrucomicrobiaceae* (rs9349825) (**Supplementary Figure 2**). Most results changed significantly after removing strongly influencing SNPs (*Acidaminococcaceae*: OR = 1.09, 95% CI, 0.89–1.34, *p* = 0.399; *Alcaligenaceae*: OR = 0.89, 95% CI, 0.81–0.98, *p* = 0.018; *Clostridiaceae_1*: OR = 0.83, 95% CI, 0.74–0.93, *p* = 0.001; *Oxalobacteraceae*: OR = 1.07, 95% CI, 1.01–1.13, *p* = 0.024; *Rikenellaceae*: OR = 0.90, 95% CI, 0.84–0.96, *p* = 0.002; *Verrucomicrobiaceae*: OR = 1.10, 95% CI, 1.00–1.21, *p* = 0.042). However, after FDR correction, the causal effect between gut microbiome and T2DM was absent (FDR *p* > 0.05). The detailed results are shown in **Supplementary Table 4**.

#### 3.2.3 Further Analyses

The inverse MR was conducted to explore whether there was causal link between T2DM and gut microbiome. SNPs (*p* < 5 × 10^−8^) significantly associated with T2DM risk were used as IVs (**Supplementary Tables 5, 6**). For European, the IVW estimates showed that T2DM was related to a decrease in the abundance of *Bacteroidaceae* (OR = 0.97, 95% CI, 0.94–0.99, *P* = 0.042) and *Oxalobacteraceae* (OR = 0.94, 95% CI, 0.88–0.99, *p* = 0.030). The results of MR-Egger regression and MR-PRESSO suggested that there was no significant horizontal pleiotropy. However, the results changed substantially after FDR correction. For Asian, the results of IVW indicated that T2DM was associated with reduced *Oxalobacteraceae* (OR = 0.94, 95% CI, 0.88–0.99, *p* = 0.036). There was no significant horizontal pleiotropy between IVs and outcome, but no evidence for genetic correlations of T2DM with gut microbiome after FDR correction. Exact values are listed in **Supplementary Tables 7**, **8**.

## 4 Discussion

In the present study, the published available GWAS summary-level data were used to perform two-sample MR. The results revealed that genetically predicted level of some gut microbiome families was causally related to T2DM risk. However, there was no evidence for genetic correlation of the abundance of gut microbiome with T2DM after FDR correction.

The gut microbiome is a complex colony of microorganisms living in the gastrointestinal tract of the host. The gut microbiome has a critical physiological role in metabolism and mounting evidence demonstrates that gut microbiome compositions are involved in numerous metabolic disorders. A study with 292 Danish individuals demonstrated that compared with subjects with high intestinal flora richness, subjects with low intestinal flora richness were characterized by obesity, insulin resistance and dyslipidaemia (27). Cotillard et al. reported that subjects with reduced gut microbial gene richness showed significant metabolic disturbance and low-grade inflammation, which were characteristics of T2DM (28). A metagenome-wide association study (MGWAS) indicated that T2DM patients were accompanied by moderate degree of gut microbiome dysbiosis and the gut microbial markers might help classify T2DM (29). In addition, compared with participants without diabetes, patients with T2DM had a lower richness of gut microbiome (30). The mathematical model of the metagenomic profiles established based on the gut microbiome could identify T2DM with high accuracy (31). Applying this model to women with impaired glucose tolerance, it could identify women with diabetes-like metabolism. A study indicated that insulin resistance was closely related to gut microbial variations and gut microbiome could be used to develop precise medical strategies to prevent and delay T2DM (32). Recently, a separate-sample MR suggested that *Anaerostipes* was a protective factor in the development of T2DM (33). A study showed that constructed microbiome risk score was consistently associated with T2DM and future glucose increment and was related to a variety of blood metabolites derived from gut microbiome (34). Maskarinec et al. demonstrated that T2DM was related to the abundance of some intestinal floras and gut microbiome might cause chronic systemic inflammation and T2DM through bacterial translocation (35). A study showed that microbial-derived or microbial-modified metabolites in serum could predict the risk of T2DM (36). However, the available evidence was inconsistent and there was no consensus on whether there was causal link between gut microbiome and the occurrence of T2DM. In addition, the taxonomic groups of gut microbiome that are responsible for T2DM were unclear.

There has been considerable interest in the potential molecular mechanisms of gut microbiome in the onset and progression of T2DM. Gut microbiome composition was involved in the pathogenesis of T2DM by regulating inflammation, modulating energy homeostasis, interacting with diet, affecting intestinal permeability, insulin sensitivity, glucose, and lipid metabolism (37). Gut microbiome and microbial products induced the production of interleukin (IL)-10 which improved glucose metabolism and prevented aging-related insulin resistance (38, 39). Gut microbiome composition improved IL-22 production and Treg cell differentiation, suggesting that they had the properties that restored insulin sensitivity and alleviated the symptoms of T2DM (40, 41). Increased intestinal permeability is one of the clinical signs of T2DM. *Akkermansia muciniphila* activated AMPK in the epithelium to improve the tight junctions of the intestine and thereby reduced the intestinal permeability. In addition, in the adipose tissue, *Akkermansia muciniphila* increased the levels of 2-acylglycerol, 2-palmitoylglycerol, and 2-oleoyl glycerol which increased fatty acid oxidation and fat cell differentiation (42). A study indicated that berberine had the property of improving insulin resistance by decreasing the relative abundance of gut microbiome, including *Streptococcaceae* (43). In addition, human milk insulin was negatively associated with *Streptococcaceae*, indicating that it might be related to the occurrence and development of diabetes (44). These available evidences indicate that gut microbiome composition may be involved in the course of T2DM and affect disease symptoms.

Since the implementation of the MR approach reduced the interference of confounding factors and the reverse causality of the results, the present study might be more convincing than observational studies. However, some limitations should be mentioned. First, given the absence of the data of basic demographic information and clinical manifestations, further subgroup analysis could not be carried out. Second, the current understanding of the gut microbiome limited our study. We lacked sufficient clues to infer the molecular mechanisms of gut microbiome and T2DM due to the absence of epidemiological studies on gut microbiome and metabolic disorders. Third, SNPs obtained based on genome-wide statistical significance threshold (5 × 10^−8^) were too limited for further study, therefore only the SNPs that met the locus-wide significance level (1 × 10^−5^) were selected. These restrictions limited the generalizability of the results and the accuracy of the study might have been compromised.

In summary, the study is leveraging MR to find that there is no evidence of the association between the 28 gut microbiome families and T2DM risk. However, in view of the biological plausibility, further studies are needed to explore the relationship between gut microbiome and the risk of T2DM, which is conducive to exploring the pathogenesis of diabetes.

## Data Availability Statement

The original contributions presented in the study are included in the article/**Supplementary Material**. Further inquiries can be directed to the corresponding authors.

## Ethics Statement

The article does not contain any studies with human participants or animals performed by any of the authors.

## Author Contributions

Conceptualization: H-FP and JN. Writing—original draft and writing—review and editing: KX and J-JZ. Data curation: Y-YX and XZ. All authors discussed the results and contributed to the final manuscript.

## Funding

This study was funded by the National Natural Science Foundation of China (82103932), Nature Science Foundation of Anhui Province of China (2018085QH361) and Nature Science Foundation of Anhui Medical University (2020xkj011).

## Conflict of Interest

The authors declare that the research was conducted in the absence of any commercial or financial relationships that could be construed as a potential conflict of interest.

## Publisher’s Note

All claims expressed in this article are solely those of the authors and do not necessarily represent those of their affiliated organizations, or those of the publisher, the editors and the reviewers. Any product that may be evaluated in this article, or claim that may be made by its manufacturer, is not guaranteed or endorsed by the publisher.

## Supplementary Material

Supplementary Figure 1 Sensitivity analysis of the genetic risk of T2DM with gut microbiome in European descent (**A**: *Desulfovibrionaceae*; **B**: *Methanobacteriaceae*).

Supplementary Figure 2 Sensitivity analysis of the genetic risk of T2DM with gut microbiome in Asian descent (**A**: *Acidaminococcaceae*; **B**: *Alcaligenaceae*; **C**: *Clostridiaceae_1*; **D**: *Oxalobacteraceae*; **E**: *Rikenellaceae*; **F**: *Verrucomicrobiaceae*).
