# Supplementary figures and images for: Genetically Predicted Causality of 28 Gut Microbiome Families and Type 2 Diabetes Mellitus Risk
Source: Front Endocrinol (Lausanne). 2022 Feb 3;13:780133. doi: 10.3389/fendo.2022.780133 (PMC8851667; doi:10.3389/fendo.2022.780133)

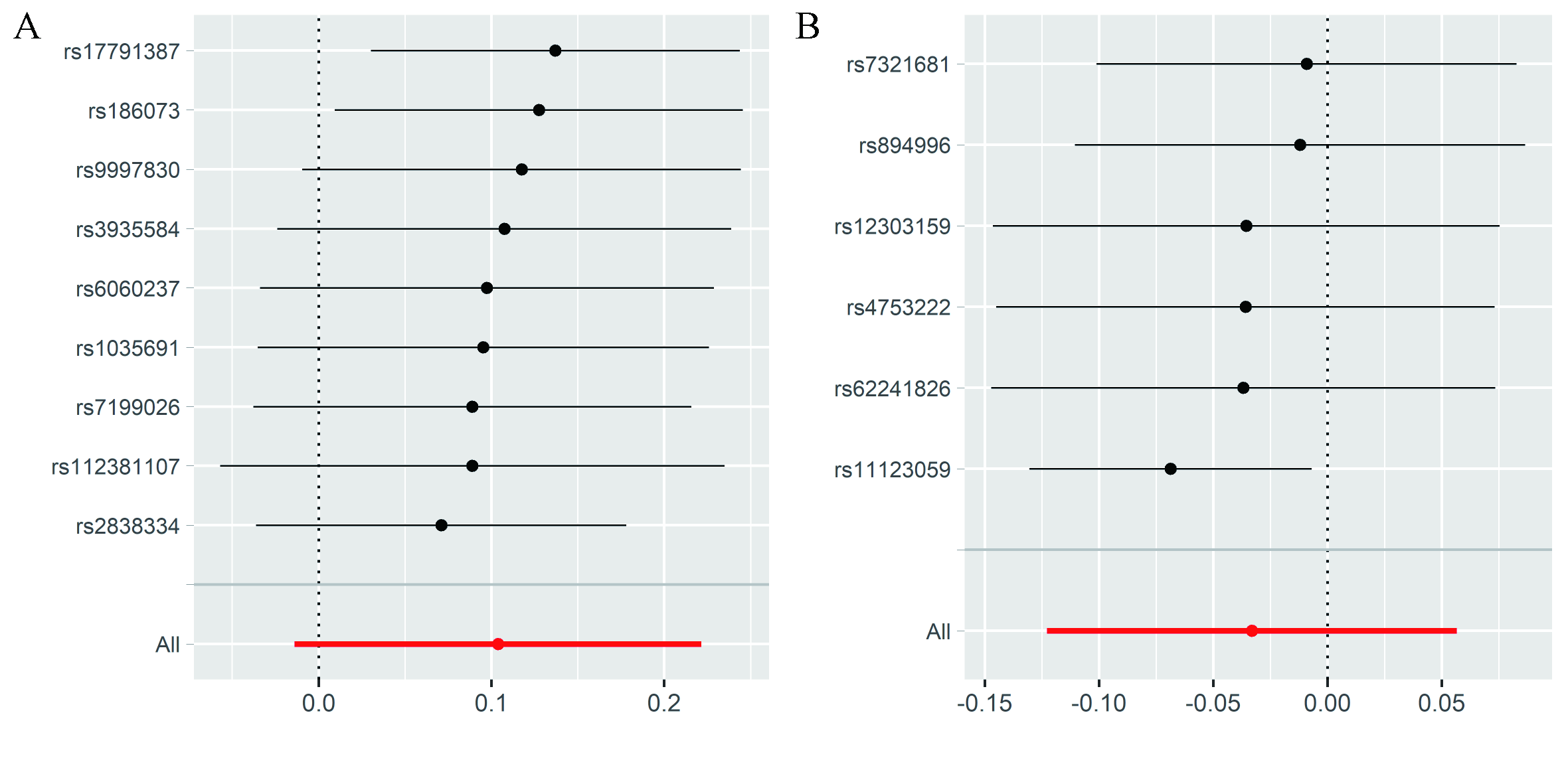

Supplement: Supplementary Figure 1 — Sensitivity analysis of the genetic risk of T2DM with gut microbiome in European descent (A: Desulfovibrionaceae; B: Methanobacteriaceae). [file Image_1.tif]

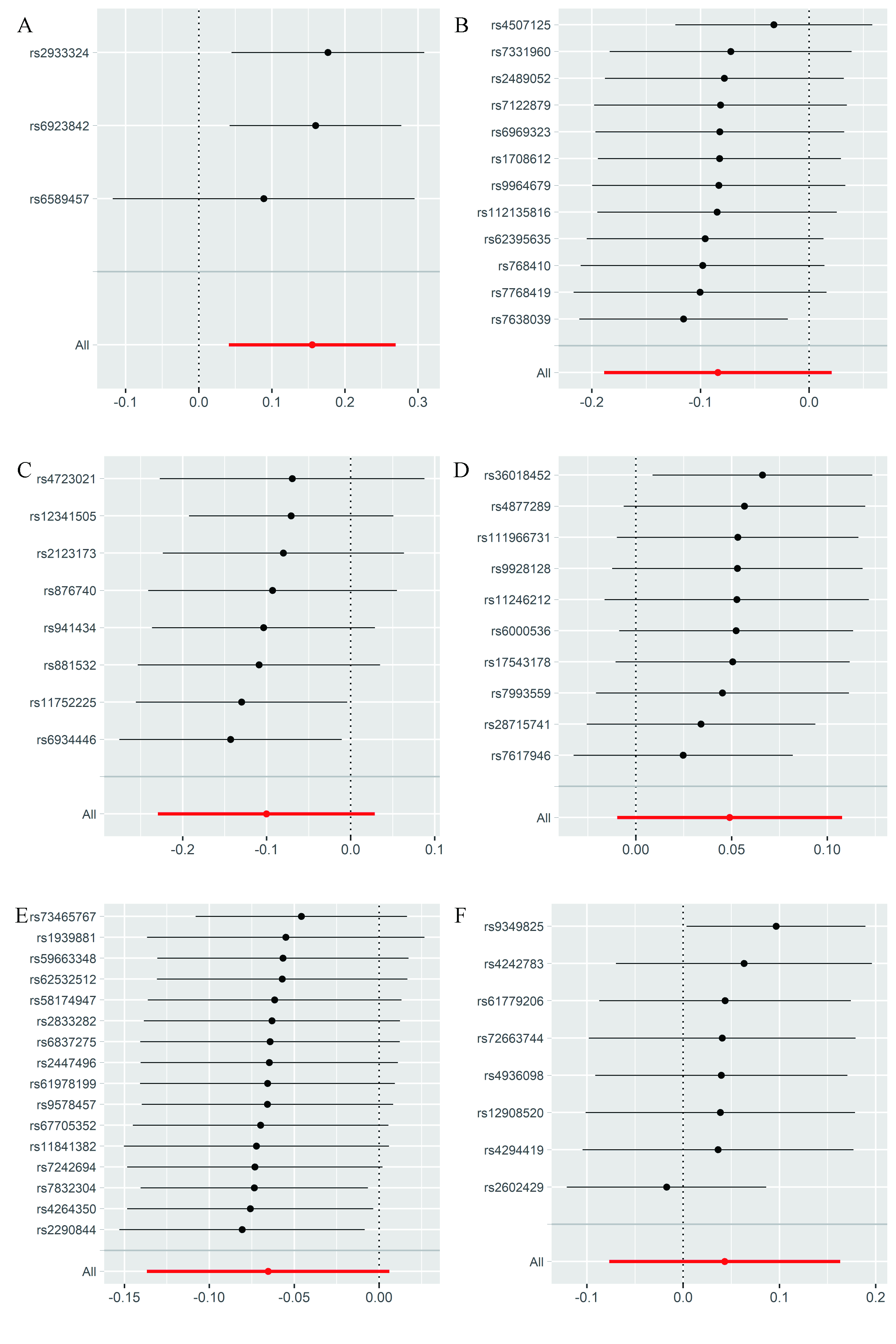

Supplement: Supplementary Figure 2 — Sensitivity analysis of the genetic risk of T2DM with gut microbiome in Asian descent (A: Acidaminococcaceae; B: Alcaligenaceae; C: Clostridiaceae_1; D: Oxalobacteraceae; E: Rikenellaceae; F: Verrucomicrobiaceae). [file Image_2.tif]
