# Supplementary material for: Genetically Predicted Causality of 28 Gut Microbiome Families and Type 2 Diabetes Mellitus Risk
Source: Front Endocrinol (Lausanne). 2022 Feb 3;13:780133. doi: 10.3389/fendo.2022.780133 (PMC8851667; doi:10.3389/fendo.2022.780133)
Supplement: Supplementary file 3 [file Table_1.docx]

| **Supplementary Table 1. SNPs used as IVs from gut microbiome and T2DM GWAS (European)** | | | | | | | | | | | | |
| --- | --- | --- | --- | --- | --- | --- | --- | --- | --- | --- | --- | --- |
| **Bacterial traits** | **SNP** | **Effect allele** | **Other allele** | **Gut microbiome** | | | **T2DM** | | | **Proxy SNP** | **Target effect allele** | **Target other allele** |
|  |  |  |  | **Beta** | **SE** | ***P* value** | **Beta** | **SE** | ***P* value** |  |  |  |
| *Acidaminococcaceae* | rs2933324 | G | A | 0.065 | 0.014 | 2.910E-06 | 0.014 | 0.009 | 0.117 |  |  |  |
|  | rs6589457 | A | G | 0.166 | 0.035 | 2.320E-06 | -0.020 | 0.018 | 0.268 |  |  |  |
|  | rs6923842 | C | T | 0.080 | 0.017 | 1.530E-06 | -0.017 | 0.010 | 0.084 |  |  |  |
| *Actinomycetaceae* | rs12771712 | C | T | 0.094 | 0.021 | 7.960E-06 | -0.001 | 0.011 | 0.957 |  |  |  |
|  | rs2889192 | G | T | 0.089 | 0.019 | 2.760E-06 | 0.004 | 0.010 | 0.670 |  |  |  |
|  | rs34583783 | G | T | 0.124 | 0.026 | 4.420E-06 | 0.021 | 0.012 | 0.092 |  |  |  |
|  | rs4073240 | G | A | 0.073 | 0.016 | 7.530E-06 | 0.004 | 0.008 | 0.663 |  |  |  |
| *Alcaligenaceae* | rs112135816 | G | T | 0.077 | 0.017 | 6.560E-06 | -0.003 | 0.012 | 0.794 |  |  |  |
|  | rs1708612 | A | G | 0.059 | 0.013 | 7.150E-06 | 0.014 | 0.009 | 0.125 |  |  |  |
|  | rs2489052 | C | A | 0.051 | 0.011 | 6.510E-06 | -0.002 | 0.008 | 0.832 |  |  |  |
|  | rs4507125 | A | C | -0.060 | 0.013 | 5.890E-06 | 0.024 | 0.010 | 0.014 |  |  |  |
|  | rs62395635 | C | T | -0.111 | 0.024 | 2.980E-06 | -0.007 | 0.016 | 0.676 |  |  |  |
|  | rs6969323 | A | C | -0.059 | 0.013 | 4.320E-06 | 0.003 | 0.009 | 0.748 |  |  |  |
|  | rs7122879 | C | T | -0.058 | 0.013 | 9.160E-06 | -0.001 | 0.009 | 0.955 |  |  |  |
|  | rs7331960 | A | G | 0.049 | 0.011 | 8.290E-06 | -0.004 | 0.008 | 0.633 |  |  |  |
|  | rs7638039 | C | T | -0.062 | 0.013 | 1.400E-06 | 0.007 | 0.009 | 0.450 |  |  |  |
|  | rs768410 | G | T | 0.049 | 0.011 | 9.180E-06 | 0.013 | 0.008 | 0.110 |  |  |  |
|  | rs7768419 | A | G | -0.126 | 0.028 | 8.850E-06 | -0.014 | 0.017 | 0.417 | rs6935444 | A | T |
|  | rs9964679 | A | G | 0.053 | 0.012 | 5.870E-06 | -0.024 | 0.009 | 0.006 |  |  |  |
| *Bacteroidaceae* | rs11585893 | A | G | -0.073 | 0.015 | 2.150E-06 | 0.012 | 0.011 | 0.295 | rs61776263 | C | G |
|  | rs16945128 | A | C | -0.078 | 0.018 | 7.330E-06 | 0.015 | 0.013 | 0.281 |  |  |  |
|  | rs17619981 | G | T | -0.087 | 0.019 | 3.210E-06 | 0.004 | 0.013 | 0.780 |  |  |  |
|  | rs2023437 | C | T | 0.078 | 0.017 | 4.770E-06 | 0.000 | 0.012 | 0.981 |  |  |  |
|  | rs234027 | G | A | 0.051 | 0.011 | 8.430E-06 | 0.006 | 0.008 | 0.513 |  |  |  |
|  | rs3113372 | C | T | 0.048 | 0.011 | 9.110E-06 | 0.012 | 0.008 | 0.134 |  |  |  |
|  | rs66474973 | G | T | 0.078 | 0.016 | 2.110E-06 | 0.003 | 0.012 | 0.835 |  |  |  |
|  | rs6795673 | C | T | 0.054 | 0.010 | 3.110E-07 | -0.005 | 0.008 | 0.527 |  |  |  |
| *Bacteroidales_S24-7* | rs12748533 | G | T | -0.079 | 0.017 | 4.780E-06 | -0.006 | 0.009 | 0.465 |  |  |  |
|  | rs2899398 | C | T | -0.077 | 0.017 | 8.790E-06 | -0.008 | 0.009 | 0.362 |  |  |  |
|  | rs61508842 | C | T | -0.121 | 0.027 | 9.030E-06 | 0.010 | 0.013 | 0.434 |  |  |  |
|  | rs6742712 | C | T | -0.168 | 0.034 | 1.440E-06 | -0.004 | 0.017 | 0.812 |  |  |  |
|  | rs696267 | C | T | 0.082 | 0.017 | 1.700E-06 | 0.002 | 0.008 | 0.779 |  |  |  |
|  | rs80098946 | A | G | 0.142 | 0.033 | 9.160E-06 | 0.007 | 0.015 | 0.639 |  |  |  |
| *Bifidobacteriaceae* | rs10831953 | A | G | -0.054 | 0.012 | 7.950E-06 | 0.000 | 0.008 | 0.972 |  |  |  |
|  | rs13020688 | A | G | -0.058 | 0.012 | 1.990E-06 | 0.002 | 0.008 | 0.770 |  |  |  |
|  | rs1961273 | C | T | 0.067 | 0.013 | 2.700E-07 | -0.007 | 0.009 | 0.419 |  |  |  |
|  | rs677010 | C | T | 0.090 | 0.023 | 8.660E-06 | 0.027 | 0.017 | 0.102 |  |  |  |
|  | rs7174549 | C | T | 0.054 | 0.012 | 9.450E-06 | 0.007 | 0.008 | 0.437 |  |  |  |
|  | rs7971116 | A | G | -0.054 | 0.012 | 8.610E-06 | -0.003 | 0.008 | 0.666 |  |  |  |
|  | rs857444 | T | C | -0.054 | 0.012 | 5.510E-06 | 0.006 | 0.008 | 0.462 |  |  |  |
| *Christensenellaceae* | rs12657403 | A | G | 0.078 | 0.017 | 5.200E-06 | 0.023 | 0.012 | 0.066 |  |  |  |
|  | rs4953394 | G | T | -0.048 | 0.011 | 8.340E-06 | 0.016 | 0.008 | 0.040 |  |  |  |
|  | rs5752918 | A | G | -0.065 | 0.015 | 9.670E-06 | -0.005 | 0.011 | 0.637 |  |  |  |
|  | rs6813335 | C | T | -0.048 | 0.011 | 8.310E-06 | -0.019 | 0.008 | 0.016 |  |  |  |
|  | rs7211194 | C | T | 0.049 | 0.011 | 9.150E-06 | 0.021 | 0.008 | 0.006 |  |  |  |
|  | rs78521377 | C | T | 0.120 | 0.027 | 8.920E-06 | -0.026 | 0.018 | 0.158 |  |  |  |
|  | rs8110909 | A | G | -0.051 | 0.012 | 7.620E-06 | 0.004 | 0.008 | 0.656 |  |  |  |
|  | rs870002 | T | C | -0.049 | 0.011 | 4.850E-06 | 0.008 | 0.008 | 0.327 |  |  |  |
| *Clostridiaceae_1* | rs11752225 | C | T | 0.071 | 0.016 | 8.470E-06 | -0.018 | 0.011 | 0.094 |  |  |  |
|  | rs12341505 | A | G | -0.080 | 0.018 | 5.470E-06 | -0.012 | 0.012 | 0.320 |  |  |  |
|  | rs2123173 | T | C | 0.074 | 0.016 | 5.710E-06 | 0.011 | 0.011 | 0.289 |  |  |  |
|  | rs4723021 | C | T | 0.106 | 0.024 | 6.280E-06 | -0.012 | 0.017 | 0.484 |  |  |  |
|  | rs6934446 | A | G | -0.073 | 0.017 | 6.390E-06 | -0.011 | 0.012 | 0.377 |  |  |  |
|  | rs881532 | G | A | 0.053 | 0.012 | 6.730E-06 | -0.014 | 0.008 | 0.081 |  |  |  |
|  | rs941434 | A | C | -0.179 | 0.039 | 4.800E-06 | 0.024 | 0.017 | 0.147 |  |  |  |
| *Coriobacteriaceae* | rs11250875 | C | T | -0.062 | 0.013 | 2.550E-06 | 0.005 | 0.010 | 0.605 |  |  |  |
|  | rs2442778 | A | G | 0.116 | 0.026 | 9.010E-06 | 0.003 | 0.018 | 0.876 |  |  |  |
|  | rs34739816 | G | T | 0.095 | 0.021 | 4.610E-06 | 0.010 | 0.014 | 0.494 | rs12950162 | C | T |
|  | rs34867574 | G | T | -0.061 | 0.014 | 9.090E-06 | -0.005 | 0.010 | 0.627 |  |  |  |
|  | rs719099 | A | G | 0.077 | 0.016 | 5.630E-07 | -0.001 | 0.012 | 0.933 |  |  |  |
| *Defluviitaleaceae* | rs112893842 | C | T | -0.108 | 0.023 | 4.300E-06 | -0.001 | 0.012 | 0.914 |  |  |  |
|  | rs1908593 | T | C | 0.071 | 0.016 | 6.580E-06 | -0.002 | 0.008 | 0.774 |  |  |  |
|  | rs4344384 | G | T | 0.070 | 0.016 | 7.980E-06 | 0.005 | 0.008 | 0.576 |  |  |  |
|  | rs540220 | C | T | 0.124 | 0.029 | 9.290E-06 | 0.032 | 0.015 | 0.030 |  |  |  |
|  | rs7664875 | A | G | -0.135 | 0.029 | 3.960E-06 | 0.008 | 0.015 | 0.594 |  |  |  |
|  | rs9725395 | A | G | -0.139 | 0.029 | 2.940E-06 | 0.011 | 0.015 | 0.471 |  |  |  |
| *Desulfovibrionaceae* | rs1035691 | A | G | -0.056 | 0.012 | 8.250E-06 | -0.010 | 0.008 | 0.204 |  |  |  |
|  | rs112381107 | C | T | 0.211 | 0.046 | 2.820E-06 | 0.030 | 0.018 | 0.094 |  |  |  |
|  | rs17791387 | A | G | -0.071 | 0.015 | 3.140E-06 | 0.014 | 0.010 | 0.159 |  |  |  |
|  | rs186073 | C | T | -0.054 | 0.012 | 5.470E-06 | 0.008 | 0.008 | 0.359 | rs1827864 | C | T |
|  | rs2838334 | A | G | -0.058 | 0.012 | 2.700E-06 | -0.024 | 0.008 | 0.005 |  |  |  |
|  | rs3935584 | C | T | -0.051 | 0.012 | 9.950E-06 | -0.003 | 0.008 | 0.676 |  |  |  |
|  | rs6060237 | A | G | -0.083 | 0.017 | 3.180E-07 | -0.014 | 0.012 | 0.258 |  |  |  |
|  | rs7199026 | A | C | 0.053 | 0.012 | 5.510E-06 | 0.014 | 0.008 | 0.089 |  |  |  |
|  | rs9997830 | G | T | 0.052 | 0.012 | 9.820E-06 | -0.002 | 0.008 | 0.776 |  |  |  |
| *Enterobacteriaceae* | rs11026529 | A | G | 0.082 | 0.019 | 9.630E-06 | 0.004 | 0.012 | 0.737 |  |  |  |
|  | rs2374342 | A | C | -0.057 | 0.013 | 7.410E-06 | 0.000 | 0.008 | 0.972 |  |  |  |
|  | rs504442 | T | G | 0.083 | 0.019 | 6.710E-06 | -0.012 | 0.012 | 0.319 |  |  |  |
|  | rs62210022 | C | T | -0.069 | 0.015 | 7.640E-06 | -0.009 | 0.010 | 0.375 |  |  |  |
|  | rs78143293 | A | G | -0.084 | 0.017 | 1.270E-06 | -0.007 | 0.011 | 0.523 |  |  |  |
|  | rs922773 | C | T | -0.081 | 0.018 | 6.500E-06 | -0.040 | 0.011 | 0.000 |  |  |  |
| *Erysipelotrichaceae* | rs12878013 | T | C | -0.088 | 0.020 | 7.650E-06 | 0.007 | 0.016 | 0.669 |  |  |  |
|  | rs1884466 | T | C | 0.048 | 0.011 | 7.750E-06 | 0.003 | 0.008 | 0.747 |  |  |  |
|  | rs28703642 | G | T | -0.053 | 0.012 | 6.960E-06 | 0.011 | 0.009 | 0.197 |  |  |  |
|  | rs35161940 | C | T | 0.081 | 0.017 | 1.670E-06 | 0.002 | 0.012 | 0.892 |  |  |  |
|  | rs4078432 | T | C | 0.061 | 0.013 | 3.890E-06 | 0.018 | 0.010 | 0.053 |  |  |  |
|  | rs7100916 | G | T | -0.053 | 0.012 | 7.170E-06 | 0.016 | 0.009 | 0.060 |  |  |  |
|  | rs989094 | C | T | -0.050 | 0.011 | 9.410E-06 | -0.005 | 0.008 | 0.508 |  |  |  |
| *Lachnospiraceae* | rs10402491 | C | T | 0.066 | 0.015 | 7.140E-06 | 0.014 | 0.012 | 0.244 |  |  |  |
|  | rs10507119 | G | T | 0.072 | 0.017 | 8.770E-06 | 0.004 | 0.013 | 0.753 |  |  |  |
|  | rs10980809 | C | T | -0.049 | 0.011 | 5.100E-06 | 0.010 | 0.008 | 0.219 |  |  |  |
|  | rs11139361 | C | T | 0.049 | 0.011 | 4.270E-06 | 0.007 | 0.008 | 0.398 |  |  |  |
|  | rs1205442 | A | C | 0.049 | 0.011 | 8.870E-06 | -0.017 | 0.008 | 0.038 |  |  |  |
|  | rs1865372 | G | T | -0.084 | 0.019 | 8.620E-06 | 0.003 | 0.014 | 0.836 |  |  |  |
|  | rs1991016 | T | C | -0.049 | 0.011 | 7.990E-06 | 0.000 | 0.008 | 0.959 |  |  |  |
|  | rs2910921 | C | T | -0.160 | 0.036 | 8.420E-06 | 0.000 | 0.019 | 0.984 |  |  |  |
|  | rs8060299 | G | T | -0.121 | 0.024 | 5.690E-07 | 0.008 | 0.017 | 0.629 |  |  |  |
| *Lactobacillaceae* | rs12549798 | G | T | 0.077 | 0.017 | 7.370E-06 | -0.007 | 0.008 | 0.394 |  |  |  |
|  | rs6092148 | G | T | -0.075 | 0.017 | 9.580E-06 | -0.001 | 0.008 | 0.945 |  |  |  |
|  | rs71416606 | C | T | -0.221 | 0.048 | 5.310E-06 | 0.012 | 0.019 | 0.510 |  |  |  |
|  | rs821171 | C | T | 0.078 | 0.017 | 4.310E-06 | 0.002 | 0.008 | 0.762 |  |  |  |
|  | rs921925 | C | A | -0.098 | 0.020 | 8.150E-07 | -0.006 | 0.010 | 0.521 |  |  |  |
|  | rs9986520 | C | T | 0.131 | 0.030 | 8.040E-06 | -0.012 | 0.015 | 0.395 |  |  |  |
| Methanobacteriaceae | rs11018664 | A | G | 0.110 | 0.025 | 9.00E-06 | -0.002 | 0.008 | 0.773 | rs4753222 | A | G |
|  | rs11123059 | A | G | 0.108 | 0.023 | 4.87E-06 | 0.018 | 0.008 | 0.022 |  |  |  |
|  | rs12303159 | A | C | -0.223 | 0.049 | 8.56E-06 | 0.005 | 0.015 | 0.725 |  |  |  |
|  | rs62241826 | C | T | -0.190 | 0.041 | 6.94E-06 | 0.003 | 0.013 | 0.821 |  |  |  |
|  | rs894996 | A | C | -0.217 | 0.045 | 1.88E-06 | 0.029 | 0.015 | 0.055 |  |  |  |
|  | rs9532770 | C | T | 0.124 | 0.028 | 9.35E-06 | -0.020 | 0.009 | 0.029 | rs7321681 | G | T |
| *Oxalobacteraceae* | rs111966731 | C | T | -0.200 | 0.044 | 6.140E-06 | 0.007 | 0.017 | 0.669 |  |  |  |
|  | rs12002250 | A | C | 0.196 | 0.045 | 5.520E-06 | -0.018 | 0.017 | 0.303 |  |  |  |
|  | rs17543178 | A | G | 0.142 | 0.028 | 4.770E-07 | -0.003 | 0.011 | 0.762 |  |  |  |
|  | rs36018452 | A | G | -0.180 | 0.040 | 7.810E-06 | 0.026 | 0.016 | 0.101 | rs62341594 | C | T |
|  | rs4877289 | A | G | 0.088 | 0.020 | 7.350E-06 | 0.011 | 0.008 | 0.142 |  |  |  |
|  | rs6000536 | C | T | -0.117 | 0.024 | 8.470E-07 | -0.012 | 0.010 | 0.229 |  |  |  |
|  | rs61661446 | A | G | -0.204 | 0.046 | 8.330E-06 | 0.011 | 0.017 | 0.506 |  |  |  |
|  | rs7617946 | C | T | 0.098 | 0.022 | 7.010E-06 | 0.001 | 0.009 | 0.907 |  |  |  |
|  | rs9928128 | C | T | 0.088 | 0.020 | 9.550E-06 | -0.008 | 0.008 | 0.286 |  |  |  |
| *Pasteurellaceae* | rs10965428 | A | C | 0.118 | 0.026 | 5.750E-06 | -0.004 | 0.014 | 0.774 |  |  |  |
|  | rs12050685 | A | G | -0.067 | 0.015 | 8.070E-06 | 0.018 | 0.008 | 0.034 |  |  |  |
|  | rs16970009 | A | G | 0.187 | 0.043 | 7.320E-06 | -0.023 | 0.019 | 0.217 |  |  |  |
|  | rs4822728 | C | T | -0.069 | 0.015 | 4.050E-06 | -0.006 | 0.008 | 0.484 |  |  |  |
|  | rs9382510 | C | T | -0.085 | 0.017 | 5.100E-07 | -0.018 | 0.009 | 0.048 |  |  |  |
|  | rs9402707 | C | T | -0.074 | 0.016 | 8.730E-06 | -0.013 | 0.009 | 0.142 | rs4840038 | A | G |
|  | rs9895850 | C | T | 0.176 | 0.041 | 9.080E-06 | 0.012 | 0.018 | 0.488 |  |  |  |
| *Peptococcaceae* | rs11136654 | G | T | 0.089 | 0.018 | 2.590E-06 | -0.003 | 0.011 | 0.789 |  |  |  |
|  | rs12144792 | C | T | 0.064 | 0.014 | 6.140E-06 | 0.003 | 0.008 | 0.696 |  |  |  |
|  | rs35703006 | G | T | 0.082 | 0.016 | 3.820E-07 | -0.005 | 0.010 | 0.644 |  |  |  |
|  | rs75430375 | C | T | -0.147 | 0.032 | 3.170E-06 | -0.012 | 0.015 | 0.421 | rs114642023 | A | C |
| *Peptostreptococcaceae* | rs10805326 | A | G | -0.056 | 0.012 | 5.440E-06 | 0.005 | 0.009 | 0.565 | rs4698729 | C | T |
|  | rs11149051 | C | T | 0.064 | 0.014 | 8.170E-06 | 0.020 | 0.010 | 0.039 |  |  |  |
|  | rs117644882 | C | T | -0.170 | 0.038 | 7.760E-06 | -0.035 | 0.018 | 0.050 |  |  |  |
|  | rs12377846 | A | C | 0.247 | 0.050 | 7.230E-07 | -0.035 | 0.019 | 0.067 |  |  |  |
|  | rs12993096 | C | T | 0.057 | 0.013 | 6.180E-06 | 0.005 | 0.009 | 0.584 |  |  |  |
|  | rs17661170 | A | G | -0.063 | 0.013 | 5.700E-07 | -0.013 | 0.009 | 0.136 |  |  |  |
|  | rs2668550 | T | C | -0.064 | 0.014 | 9.870E-06 | 0.010 | 0.010 | 0.340 |  |  |  |
|  | rs2883972 | A | G | -0.070 | 0.015 | 3.120E-06 | 0.009 | 0.010 | 0.351 |  |  |  |
|  | rs77540684 | G | T | -0.106 | 0.024 | 7.730E-06 | 0.008 | 0.017 | 0.622 | rs11259222 | A | G |
| *Porphyromonadaceae* | rs1029811 | T | G | 0.068 | 0.015 | 2.360E-06 | -0.007 | 0.011 | 0.505 |  |  |  |
|  | rs10762312 | A | G | 0.052 | 0.012 | 9.220E-06 | -0.006 | 0.009 | 0.489 |  |  |  |
|  | rs1125465 | T | C | -0.060 | 0.012 | 1.310E-06 | 0.002 | 0.009 | 0.812 |  |  |  |
|  | rs12700163 | C | T | 0.053 | 0.012 | 7.590E-06 | -0.004 | 0.009 | 0.656 |  |  |  |
|  | rs2675411 | G | A | -0.054 | 0.012 | 8.440E-06 | 0.004 | 0.009 | 0.629 |  |  |  |
|  | rs35233670 | C | T | 0.047 | 0.011 | 9.560E-06 | -0.013 | 0.008 | 0.102 |  |  |  |
|  | rs35961441 | A | C | 0.091 | 0.021 | 8.160E-06 | 0.003 | 0.014 | 0.850 |  |  |  |
| *Prevotellaceae* | rs11102139 | C | T | 0.081 | 0.017 | 1.200E-06 | 0.009 | 0.011 | 0.425 |  |  |  |
|  | rs11151191 | C | T | -0.077 | 0.017 | 7.260E-06 | -0.001 | 0.011 | 0.939 |  |  |  |
|  | rs12057990 | C | T | 0.061 | 0.013 | 4.170E-06 | 0.005 | 0.009 | 0.583 | rs6692269 | A | C |
|  | rs12873417 | C | T | 0.059 | 0.013 | 2.980E-06 | -0.013 | 0.009 | 0.144 |  |  |  |
|  | rs13069367 | A | C | -0.053 | 0.012 | 9.200E-06 | -0.003 | 0.008 | 0.732 |  |  |  |
|  | rs2206482 | T | G | -0.056 | 0.012 | 1.590E-06 | -0.017 | 0.008 | 0.031 |  |  |  |
|  | rs3758087 | T | C | 0.057 | 0.012 | 6.710E-06 | 0.004 | 0.008 | 0.635 |  |  |  |
|  | rs4685827 | C | T | 0.066 | 0.014 | 5.430E-06 | -0.004 | 0.010 | 0.689 |  |  |  |
|  | rs6720263 | A | G | 0.059 | 0.012 | 5.300E-07 | 0.002 | 0.008 | 0.849 |  |  |  |
|  | rs7252711 | A | G | -0.073 | 0.016 | 6.590E-06 | 0.001 | 0.012 | 0.955 |  |  |  |
|  | rs77380553 | A | G | -0.075 | 0.017 | 5.650E-06 | 0.006 | 0.011 | 0.570 |  |  |  |
|  | rs912860 | G | A | -0.229 | 0.048 | 9.300E-07 | -0.018 | 0.025 | 0.489 |  |  |  |
| *Rhodospirillaceae* | rs1035406 | A | G | 0.113 | 0.025 | 6.380E-06 | 0.010 | 0.012 | 0.413 | rs12522947 | C | G |
|  | rs11630875 | C | T | -0.093 | 0.020 | 5.490E-06 | 0.004 | 0.010 | 0.722 |  |  |  |
|  | rs2162883 | C | T | 0.088 | 0.019 | 8.040E-06 | -0.004 | 0.010 | 0.716 |  |  |  |
|  | rs34142542 | C | T | -0.070 | 0.016 | 8.090E-06 | 0.004 | 0.008 | 0.633 |  |  |  |
|  | rs3768869 | A | G | -0.092 | 0.020 | 6.730E-06 | -0.011 | 0.010 | 0.287 |  |  |  |
|  | rs55661963 | A | C | -0.129 | 0.029 | 5.490E-06 | 0.020 | 0.016 | 0.201 |  |  |  |
|  | rs62255666 | A | G | -0.082 | 0.018 | 4.620E-06 | 0.002 | 0.009 | 0.821 |  |  |  |
|  | rs72714493 | A | G | 0.082 | 0.018 | 6.760E-06 | -0.010 | 0.009 | 0.253 |  |  |  |
|  | rs7623329 | A | C | -0.070 | 0.016 | 9.350E-06 | 0.001 | 0.008 | 0.875 |  |  |  |
| *Rikenellaceae* | rs11841382 | G | T | 0.077 | 0.018 | 7.530E-06 | 0.005 | 0.015 | 0.753 |  |  |  |
|  | rs1939881 | G | A | -0.104 | 0.021 | 8.770E-07 | -0.001 | 0.016 | 0.939 |  |  |  |
|  | rs2290844 | C | T | 0.083 | 0.019 | 6.530E-06 | 0.020 | 0.014 | 0.169 |  |  |  |
|  | rs2833282 | A | G | -0.071 | 0.016 | 4.320E-06 | 0.005 | 0.012 | 0.700 |  |  |  |
|  | rs4264350 | C | T | 0.052 | 0.011 | 1.330E-06 | 0.005 | 0.008 | 0.530 |  |  |  |
|  | rs58174947 | A | G | -0.048 | 0.011 | 9.540E-06 | -0.005 | 0.008 | 0.548 |  |  |  |
|  | rs59663348 | A | G | -0.057 | 0.012 | 5.060E-06 | -0.014 | 0.010 | 0.143 |  |  |  |
|  | rs61978199 | C | T | -0.147 | 0.034 | 8.690E-06 | 0.019 | 0.019 | 0.309 |  |  |  |
|  | rs62532512 | A | C | 0.051 | 0.011 | 2.250E-06 | -0.003 | 0.008 | 0.675 |  |  |  |
|  | rs67705352 | G | T | 0.054 | 0.011 | 9.270E-07 | 0.007 | 0.008 | 0.382 |  |  |  |
|  | rs73465767 | C | T | 0.086 | 0.020 | 9.580E-06 | -0.025 | 0.016 | 0.113 |  |  |  |
|  | rs7832304 | G | T | 0.073 | 0.016 | 7.860E-06 | 0.006 | 0.012 | 0.600 |  |  |  |
|  | rs9578457 | A | G | 0.141 | 0.032 | 3.990E-06 | -0.008 | 0.017 | 0.666 |  |  |  |
| *Ruminococcaceae* | rs10093275 | C | T | 0.054 | 0.012 | 4.680E-06 | -0.001 | 0.009 | 0.879 |  |  |  |
|  | rs10172096 | A | G | 0.160 | 0.035 | 3.510E-06 | 0.004 | 0.020 | 0.839 |  |  |  |
|  | rs1572312 | T | G | 0.083 | 0.017 | 1.180E-06 | 0.000 | 0.013 | 0.993 |  |  |  |
| *Streptococcaceae* | rs13379080 | C | T | -0.084 | 0.018 | 4.570E-06 | -0.009 | 0.013 | 0.521 |  |  |  |
|  | rs16950051 | A | G | 0.105 | 0.024 | 7.240E-06 | 0.033 | 0.015 | 0.026 |  |  |  |
|  | rs17613937 | A | G | -0.088 | 0.019 | 9.130E-06 | -0.008 | 0.013 | 0.568 |  |  |  |
|  | rs2952189 | T | C | -0.070 | 0.015 | 3.510E-06 | -0.022 | 0.011 | 0.051 |  |  |  |
|  | rs34070557 | C | T | 0.049 | 0.011 | 8.650E-06 | 0.013 | 0.008 | 0.106 |  |  |  |
|  | rs77968078 | A | G | 0.099 | 0.022 | 7.970E-06 | -0.022 | 0.016 | 0.186 |  |  |  |
|  | rs7916711 | A | G | 0.094 | 0.021 | 9.040E-06 | -0.002 | 0.017 | 0.890 |  |  |  |
|  | rs7971284 | A | G | 0.081 | 0.018 | 7.650E-06 | 0.011 | 0.013 | 0.406 |  |  |  |
|  | rs9858426 | A | G | -0.101 | 0.023 | 9.600E-06 | -0.029 | 0.015 | 0.049 |  |  |  |
| *Veillonellaceae* | rs11700976 | A | C | -0.050 | 0.011 | 8.030E-06 | 0.001 | 0.008 | 0.879 |  |  |  |
|  | rs12668619 | A | G | 0.054 | 0.012 | 3.570E-06 | -0.006 | 0.008 | 0.461 |  |  |  |
|  | rs2036713 | C | A | -0.051 | 0.011 | 6.830E-06 | 0.002 | 0.008 | 0.823 |  |  |  |
|  | rs2561116 | T | G | -0.084 | 0.019 | 6.510E-06 | 0.004 | 0.013 | 0.743 |  |  |  |
|  | rs2585520 | T | G | 0.088 | 0.020 | 8.370E-06 | 0.000 | 0.014 | 0.992 |  |  |  |
|  | rs4461038 | A | G | -0.054 | 0.012 | 6.270E-06 | -0.008 | 0.008 | 0.310 |  |  |  |
|  | rs4736971 | A | C | -0.052 | 0.012 | 6.230E-06 | -0.003 | 0.008 | 0.753 |  |  |  |
|  | rs4797169 | C | T | -0.060 | 0.013 | 3.070E-06 | 0.001 | 0.009 | 0.914 |  |  |  |
|  | rs66716149 | C | T | -0.057 | 0.012 | 1.360E-06 | -0.004 | 0.008 | 0.569 | rs12741784 | C | T |
|  | rs6909981 | C | T | -0.063 | 0.014 | 6.310E-06 | -0.010 | 0.010 | 0.304 |  |  |  |
|  | rs79535861 | A | C | 0.104 | 0.021 | 6.440E-07 | -0.004 | 0.013 | 0.746 |  |  |  |
|  | rs9345168 | A | C | -0.051 | 0.011 | 7.370E-06 | 0.004 | 0.008 | 0.594 |  |  |  |
| *Verrucomicrobiaceae* | rs12908520 | A | G | -0.059 | 0.013 | 5.840E-06 | -0.013 | 0.008 | 0.116 |  |  |  |
|  | rs4242783 | A | G | -0.068 | 0.015 | 3.310E-06 | -0.010 | 0.009 | 0.259 |  |  |  |
|  | rs4936098 | A | G | 0.066 | 0.014 | 6.830E-07 | 0.010 | 0.007 | 0.171 |  |  |  |
|  | rs61779206 | C | T | 0.076 | 0.017 | 5.640E-06 | 0.004 | 0.010 | 0.692 |  |  |  |
|  | rs72663744 | C | T | -0.064 | 0.015 | 9.820E-06 | 0.029 | 0.009 | 0.001 |  |  |  |
|  | rs9349825 | A | G | -0.071 | 0.015 | 1.700E-06 | 0.002 | 0.009 | 0.805 |  |  |  |

Abbreviations: SNP, single nucleotide polymorphism; IVs, instrumental variables; T2DM, type 2 diabetes mellitus; GWAS, genome-wide association study.
