# Supplementary material for: Genetically Predicted Causality of 28 Gut Microbiome Families and Type 2 Diabetes Mellitus Risk
Source: Front Endocrinol (Lausanne). 2022 Feb 3;13:780133. doi: 10.3389/fendo.2022.780133 (PMC8851667; doi:10.3389/fendo.2022.780133)
Supplement: Supplementary file 4 [file Table_2.docx]

| **Supplementary Table 2. SNPs used as IVs from gut microbiome and T2DM GWAS (Asian)** | | | | | | | | | | | | |
| --- | --- | --- | --- | --- | --- | --- | --- | --- | --- | --- | --- | --- |
| **Bacterial traits** | **SNP** | **Effect allele** | **Other allele** | **Gut microbiome** | | | **T2DM** | | | **Proxy SNP** | **Target effect allele** | **Target other allele** |
|  |  |  |  | **Beta** | **SE** | ***P* value** | **Beta** | **SE** | ***P* value** |  |  |  |
| *Acidaminococcaceae* | rs2933324 | G | A | 0.065 | 0.014 | 2.910E-06 | 0.006 | 0.008 | 0.438 |  |  |  |
|  | rs6589457 | A | G | 0.166 | 0.035 | 2.320E-06 | 0.031 | 0.012 | 0.008 |  |  |  |
|  | rs6923842 | C | T | 0.080 | 0.017 | 1.530E-06 | 0.007 | 0.020 | 0.741 |  |  |  |
| *Actinomycetaceae* | rs12771712 | C | T | 0.094 | 0.021 | 7.960E-06 | -0.007 | 0.007 | 0.304 |  |  |  |
|  | rs34583783 | G | T | 0.124 | 0.026 | 4.420E-06 | -0.003 | 0.013 | 0.809 |  |  |  |
|  | rs4073240 | G | A | 0.073 | 0.016 | 7.530E-06 | 0.016 | 0.007 | 0.026 |  |  |  |
| *Alcaligenaceae* | rs112135816 | G | T | 0.077 | 0.017 | 6.560E-06 | 0.000 | 0.034 | 0.998 |  |  |  |
|  | rs1708612 | A | G | 0.059 | 0.013 | 7.150E-06 | -0.007 | 0.013 | 0.582 |  |  |  |
|  | rs2489052 | C | A | 0.051 | 0.011 | 6.510E-06 | -0.013 | 0.012 | 0.278 |  |  |  |
|  | rs4507125 | A | C | -0.060 | 0.013 | 5.890E-06 | 0.025 | 0.007 | 0.000 |  |  |  |
|  | rs62395635 | C | T | -0.111 | 0.024 | 2.980E-06 | -0.012 | 0.020 | 0.548 |  |  |  |
|  | rs6969323 | A | C | -0.059 | 0.013 | 4.320E-06 | 0.006 | 0.009 | 0.493 |  |  |  |
|  | rs7122879 | C | T | -0.058 | 0.013 | 9.160E-06 | 0.006 | 0.008 | 0.424 |  |  |  |
|  | rs7331960 | A | G | 0.049 | 0.011 | 8.290E-06 | -0.011 | 0.008 | 0.148 |  |  |  |
|  | rs7638039 | C | T | -0.062 | 0.013 | 1.400E-06 | -0.014 | 0.009 | 0.115 |  |  |  |
|  | rs768410 | G | T | 0.049 | 0.011 | 9.180E-06 | 0.002 | 0.007 | 0.743 |  |  |  |
|  | rs7768419 | A | G | -0.126 | 0.028 | 8.850E-06 | -0.001 | 0.014 | 0.948 |  |  |  |
|  | rs9964679 | A | G | 0.053 | 0.012 | 5.870E-06 | -0.005 | 0.007 | 0.480 |  |  |  |
| *Bacteroidaceae* | rs11585893 | A | G | -0.073 | 0.015 | 2.150E-06 | 0.010 | 0.008 | 0.217 |  |  |  |
|  | rs12040461 | A | G | -0.059 | 0.014 | 9.470E-06 | 0.018 | 0.007 | 0.013 |  |  |  |
|  | rs13207588 | A | G | -0.058 | 0.013 | 9.000E-06 | 0.009 | 0.016 | 0.558 |  |  |  |
|  | rs16945128 | A | C | -0.078 | 0.018 | 7.330E-06 | -0.091 | 0.073 | 0.210 | rs16945163 | A | T |
|  | rs17619981 | G | T | -0.087 | 0.019 | 3.210E-06 | 0.023 | 0.029 | 0.417 |  |  |  |
|  | rs234027 | G | A | 0.051 | 0.011 | 8.430E-06 | 0.027 | 0.007 | 0.000 |  |  |  |
|  | rs28365789 | A | G | -0.105 | 0.026 | 8.750E-06 | -0.005 | 0.007 | 0.511 |  |  |  |
|  | rs3113372 | C | T | 0.048 | 0.011 | 9.110E-06 | 0.005 | 0.007 | 0.499 |  |  |  |
|  | rs66474973 | G | T | 0.078 | 0.016 | 2.110E-06 | 0.004 | 0.024 | 0.872 |  |  |  |
|  | rs66710942 | C | T | 0.048 | 0.011 | 8.610E-06 | -0.004 | 0.008 | 0.573 |  |  |  |
|  | rs6795673 | C | T | 0.054 | 0.010 | 3.110E-07 | -0.002 | 0.007 | 0.783 |  |  |  |
|  | rs7322219 | C | T | 0.052 | 0.012 | 6.170E-06 | -0.009 | 0.008 | 0.270 |  |  |  |
| *Bacteroidales_S24-7* | rs10872669 | A | G | -0.127 | 0.027 | 4.090E-06 | 0.006 | 0.032 | 0.863 |  |  |  |
|  | rs12748533 | G | T | -0.079 | 0.017 | 4.780E-06 | 0.002 | 0.007 | 0.758 |  |  |  |
|  | rs2899398 | C | T | -0.077 | 0.017 | 8.790E-06 | 0.007 | 0.008 | 0.355 |  |  |  |
|  | rs6742712 | C | T | -0.168 | 0.034 | 1.440E-06 | 0.012 | 0.008 | 0.112 |  |  |  |
|  | rs696267 | C | T | 0.082 | 0.017 | 1.700E-06 | 0.004 | 0.012 | 0.739 |  |  |  |
|  | rs738193 | C | T | -0.084 | 0.016 | 3.600E-07 | -0.004 | 0.007 | 0.561 |  |  |  |
| *Bifidobacteriaceae* | rs10831953 | A | G | -0.054 | 0.012 | 7.950E-06 | -0.003 | 0.013 | 0.810 |  |  |  |
|  | rs12446429 | C | T | -0.084 | 0.019 | 3.750E-06 | 0.009 | 0.010 | 0.353 |  |  |  |
|  | rs13020688 | A | G | -0.058 | 0.012 | 1.990E-06 | 0.011 | 0.019 | 0.564 |  |  |  |
|  | rs1961273 | C | T | 0.067 | 0.013 | 2.700E-07 | 0.001 | 0.007 | 0.837 |  |  |  |
|  | rs479980 | A | C | 0.054 | 0.012 | 7.880E-06 | -0.027 | 0.027 | 0.312 |  |  |  |
|  | rs5001584 | A | G | 0.054 | 0.012 | 9.330E-06 | -0.008 | 0.007 | 0.280 |  |  |  |
|  | rs540489 | G | T | 0.063 | 0.014 | 5.140E-06 | -0.011 | 0.007 | 0.150 |  |  |  |
|  | rs677010 | C | T | 0.090 | 0.023 | 8.660E-06 | 0.008 | 0.007 | 0.228 |  |  |  |
|  | rs7174549 | C | T | 0.054 | 0.012 | 9.450E-06 | 0.012 | 0.011 | 0.288 |  |  |  |
|  | rs7322849 | C | T | -0.109 | 0.020 | 3.290E-08 | -0.001 | 0.007 | 0.942 |  |  |  |
|  | rs73797465 | G | T | 0.091 | 0.021 | 9.480E-06 | 0.015 | 0.011 | 0.170 |  |  |  |
|  | rs79106992 | C | T | 0.083 | 0.018 | 5.670E-06 | -0.007 | 0.014 | 0.635 |  |  |  |
|  | rs7971116 | A | G | -0.054 | 0.012 | 8.610E-06 | -0.001 | 0.008 | 0.882 |  |  |  |
|  | rs857444 | T | C | -0.054 | 0.012 | 5.510E-06 | 0.002 | 0.007 | 0.837 |  |  |  |
| *Christensenellaceae* | rs12657403 | A | G | 0.078 | 0.017 | 5.200E-06 | -0.006 | 0.008 | 0.482 |  |  |  |
|  | rs13132953 | T | G | -0.050 | 0.011 | 6.100E-06 | 0.007 | 0.007 | 0.339 |  |  |  |
|  | rs4076564 | G | A | -0.189 | 0.039 | 8.060E-06 | -0.005 | 0.009 | 0.598 |  |  |  |
|  | rs4953394 | G | T | -0.048 | 0.011 | 8.340E-06 | -0.003 | 0.009 | 0.723 |  |  |  |
|  | rs5752918 | A | G | -0.065 | 0.015 | 9.670E-06 | -0.003 | 0.021 | 0.875 |  |  |  |
|  | rs62573205 | A | G | 0.064 | 0.013 | 2.070E-06 | -0.017 | 0.007 | 0.015 |  |  |  |
|  | rs6813335 | C | T | -0.048 | 0.011 | 8.310E-06 | -0.006 | 0.007 | 0.403 |  |  |  |
|  | rs7211194 | C | T | 0.049 | 0.011 | 9.150E-06 | -0.008 | 0.008 | 0.292 |  |  |  |
|  | rs77867022 | G | T | -0.160 | 0.038 | 8.810E-06 | -0.001 | 0.007 | 0.905 |  |  |  |
|  | rs78521377 | C | T | 0.120 | 0.027 | 8.920E-06 | 0.007 | 0.013 | 0.604 |  |  |  |
|  | rs8110909 | A | G | -0.051 | 0.012 | 7.620E-06 | 0.010 | 0.007 | 0.137 |  |  |  |
|  | rs870002 | T | C | -0.049 | 0.011 | 4.850E-06 | 0.010 | 0.009 | 0.275 |  |  |  |
| *Clostridiaceae_1* | rs11752225 | C | T | 0.071 | 0.016 | 8.470E-06 | 0.012 | 0.010 | 0.237 |  |  |  |
|  | rs12341505 | A | G | -0.080 | 0.018 | 5.470E-06 | 0.033 | 0.013 | 0.008 |  |  |  |
|  | rs2123173 | T | C | 0.074 | 0.016 | 5.710E-06 | -0.017 | 0.009 | 0.065 |  |  |  |
|  | rs4723021 | C | T | 0.106 | 0.024 | 6.280E-06 | -0.019 | 0.009 | 0.038 |  |  |  |
|  | rs6934446 | A | G | -0.073 | 0.017 | 6.390E-06 | -0.007 | 0.008 | 0.405 |  |  |  |
|  | rs876740 | T | C | 0.053 | 0.012 | 9.850E-06 | -0.008 | 0.007 | 0.240 |  |  |  |
|  | rs881532 | G | A | 0.053 | 0.012 | 6.730E-06 | -0.001 | 0.008 | 0.951 |  |  |  |
|  | rs941434 | A | C | -0.179 | 0.039 | 4.800E-06 | -0.139 | 0.131 | 0.288 |  |  |  |
| *Coriobacteriaceae* | rs11073596 | G | T | -0.051 | 0.011 | 8.690E-06 | -0.010 | 0.008 | 0.214 |  |  |  |
|  | rs11250875 | C | T | -0.062 | 0.013 | 2.550E-06 | -0.003 | 0.007 | 0.661 |  |  |  |
|  | rs11656361 | A | C | 0.077 | 0.017 | 7.630E-06 | 0.002 | 0.015 | 0.869 |  |  |  |
|  | rs1397793 | G | A | -0.050 | 0.011 | 8.230E-06 | -0.026 | 0.007 | 0.000 |  |  |  |
|  | rs1834859 | G | T | -0.057 | 0.013 | 8.260E-06 | -0.009 | 0.008 | 0.300 |  |  |  |
|  | rs240104 | T | C | -0.060 | 0.013 | 1.440E-06 | 0.009 | 0.012 | 0.414 |  |  |  |
|  | rs2442778 | A | G | 0.116 | 0.026 | 9.010E-06 | 0.000 | 0.013 | 0.987 |  |  |  |
|  | rs34739816 | G | T | 0.095 | 0.021 | 4.610E-06 | 0.167 | 0.117 | 0.153 | rs74767487 | A | G |
|  | rs34867574 | G | T | -0.061 | 0.014 | 9.090E-06 | 0.020 | 0.016 | 0.208 |  |  |  |
|  | rs45480394 | G | T | 0.052 | 0.011 | 5.190E-06 | -0.018 | 0.011 | 0.112 |  |  |  |
|  | rs67561917 | A | G | -0.071 | 0.015 | 5.790E-06 | -0.001 | 0.012 | 0.924 |  |  |  |
|  | rs719099 | A | G | 0.077 | 0.016 | 5.630E-07 | 0.001 | 0.009 | 0.880 |  |  |  |
| *Defluviitaleaceae* | rs112893842 | C | T | -0.108 | 0.023 | 4.300E-06 | -0.001 | 0.008 | 0.858 |  |  |  |
|  | rs1582238 | T | C | 0.075 | 0.017 | 6.280E-06 | -0.016 | 0.009 | 0.080 |  |  |  |
|  | rs1908593 | T | C | 0.071 | 0.016 | 6.580E-06 | 0.002 | 0.007 | 0.732 |  |  |  |
|  | rs4344384 | G | T | 0.070 | 0.016 | 7.980E-06 | -0.005 | 0.007 | 0.458 |  |  |  |
|  | rs4677103 | A | G | 0.097 | 0.020 | 1.020E-06 | -0.001 | 0.009 | 0.944 |  |  |  |
|  | rs540220 | C | T | 0.124 | 0.029 | 9.290E-06 | -0.011 | 0.014 | 0.426 |  |  |  |
|  | rs72731813 | C | T | -0.143 | 0.029 | 6.720E-07 | -0.193 | 0.105 | 0.067 |  |  |  |
|  | rs7664875 | A | G | -0.135 | 0.029 | 3.960E-06 | 0.011 | 0.037 | 0.758 |  |  |  |
|  | rs9608282 | G | T | -0.136 | 0.030 | 7.160E-06 | 0.007 | 0.015 | 0.655 |  |  |  |
| *Desulfovibrionaceae* | rs1035691 | A | G | -0.056 | 0.012 | 8.250E-06 | -0.013 | 0.032 | 0.690 |  |  |  |
|  | rs112381107 | C | T | 0.211 | 0.046 | 2.820E-06 | -0.015 | 0.014 | 0.287 |  |  |  |
|  | rs17791387 | A | G | -0.071 | 0.015 | 3.140E-06 | 0.003 | 0.010 | 0.778 |  |  |  |
|  | rs186073 | C | T | -0.054 | 0.012 | 5.470E-06 | -0.011 | 0.007 | 0.113 |  |  |  |
|  | rs2692012 | A | G | 0.111 | 0.025 | 2.400E-06 | 0.004 | 0.008 | 0.670 |  |  |  |
|  | rs2838334 | A | G | -0.058 | 0.012 | 2.700E-06 | 0.002 | 0.008 | 0.774 |  |  |  |
|  | rs3935584 | C | T | -0.051 | 0.012 | 9.950E-06 | -0.004 | 0.013 | 0.747 |  |  |  |
|  | rs6060237 | A | G | -0.083 | 0.017 | 3.180E-07 | -0.044 | 0.014 | 0.001 |  |  |  |
|  | rs7199026 | A | C | 0.053 | 0.012 | 5.510E-06 | 0.030 | 0.007 | 0.000 |  |  |  |
|  | rs9997830 | G | T | 0.052 | 0.012 | 9.820E-06 | -0.002 | 0.007 | 0.817 |  |  |  |
| *Enterobacteriaceae* | rs11026529 | A | G | 0.082 | 0.019 | 9.630E-06 | 0.003 | 0.007 | 0.706 |  |  |  |
|  | rs2374342 | A | C | -0.057 | 0.013 | 7.410E-06 | 0.005 | 0.007 | 0.455 |  |  |  |
|  | rs35673018 | A | G | -0.089 | 0.020 | 9.070E-06 | -0.005 | 0.012 | 0.676 |  |  |  |
|  | rs504442 | T | G | 0.083 | 0.019 | 6.710E-06 | -0.011 | 0.015 | 0.453 |  |  |  |
|  | rs62210022 | C | T | -0.069 | 0.015 | 7.640E-06 | -0.009 | 0.021 | 0.674 |  |  |  |
|  | rs78143293 | A | G | -0.084 | 0.017 | 1.270E-06 | 0.005 | 0.011 | 0.640 |  |  |  |
|  | rs79757635 | A | C | -0.076 | 0.017 | 7.680E-06 | 0.003 | 0.009 | 0.744 |  |  |  |
|  | rs922773 | C | T | -0.081 | 0.018 | 6.500E-06 | 0.012 | 0.013 | 0.336 |  |  |  |
| *Erysipelotrichaceae* | rs10072629 | A | C | 0.048 | 0.011 | 9.120E-06 | -0.003 | 0.009 | 0.742 |  |  |  |
|  | rs12878013 | T | C | -0.088 | 0.020 | 7.650E-06 | 0.077 | 0.039 | 0.048 |  |  |  |
|  | rs1884466 | T | C | 0.048 | 0.011 | 7.750E-06 | 0.012 | 0.007 | 0.082 |  |  |  |
|  | rs28703642 | G | T | -0.053 | 0.012 | 6.960E-06 | 0.018 | 0.007 | 0.013 |  |  |  |
|  | rs290833 | G | T | 0.050 | 0.011 | 7.870E-06 | 0.004 | 0.007 | 0.541 |  |  |  |
|  | rs35161940 | C | T | 0.081 | 0.017 | 1.670E-06 | 0.008 | 0.018 | 0.669 |  |  |  |
|  | rs4078432 | T | C | 0.061 | 0.013 | 3.890E-06 | 0.004 | 0.008 | 0.639 |  |  |  |
|  | rs62504402 | C | T | -0.065 | 0.013 | 2.900E-07 | -0.030 | 0.019 | 0.120 |  |  |  |
|  | rs7100916 | G | T | -0.053 | 0.012 | 7.170E-06 | 0.009 | 0.010 | 0.333 |  |  |  |
|  | rs7826267 | G | T | 0.085 | 0.020 | 6.670E-06 | 0.006 | 0.009 | 0.516 |  |  |  |
|  | rs989094 | C | T | -0.050 | 0.011 | 9.410E-06 | -0.008 | 0.008 | 0.300 |  |  |  |
| *Lachnospiraceae* | rs10402491 | C | T | 0.066 | 0.015 | 7.140E-06 | 0.012 | 0.007 | 0.083 |  |  |  |
|  | rs10507119 | G | T | 0.072 | 0.017 | 8.770E-06 | -0.014 | 0.012 | 0.230 |  |  |  |
|  | rs10980809 | C | T | -0.049 | 0.011 | 5.100E-06 | -0.003 | 0.007 | 0.675 |  |  |  |
|  | rs11139361 | C | T | 0.049 | 0.011 | 4.270E-06 | 0.003 | 0.010 | 0.739 |  |  |  |
|  | rs1205442 | A | C | 0.049 | 0.011 | 8.870E-06 | -0.010 | 0.008 | 0.182 |  |  |  |
|  | rs12562372 | G | T | -0.050 | 0.011 | 2.400E-06 | 0.008 | 0.008 | 0.299 |  |  |  |
|  | rs1865372 | G | T | -0.084 | 0.019 | 8.620E-06 | 0.018 | 0.087 | 0.836 | rs10933378 | C | T |
|  | rs1991016 | T | C | -0.049 | 0.011 | 7.990E-06 | -0.003 | 0.011 | 0.798 |  |  |  |
|  | rs2910921 | C | T | -0.160 | 0.036 | 8.420E-06 | -0.002 | 0.011 | 0.876 |  |  |  |
|  | rs62078073 | G | T | 0.052 | 0.012 | 7.980E-06 | 0.046 | 0.025 | 0.061 |  |  |  |
|  | rs79086868 | C | T | 0.078 | 0.016 | 2.840E-06 | -0.023 | 0.023 | 0.309 |  |  |  |
|  | rs8060299 | G | T | -0.121 | 0.024 | 5.690E-07 | 0.009 | 0.024 | 0.701 | rs4617895 | A | G |
|  | rs959845 | C | T | -0.049 | 0.011 | 6.620E-06 | -0.007 | 0.008 | 0.388 |  |  |  |
| *Lactobacillaceae* | rs11674854 | C | T | -0.081 | 0.017 | 3.290E-06 | 0.008 | 0.008 | 0.323 |  |  |  |
|  | rs12549798 | G | T | 0.077 | 0.017 | 7.370E-06 | -0.004 | 0.007 | 0.585 |  |  |  |
|  | rs6092148 | G | T | -0.075 | 0.017 | 9.580E-06 | -0.002 | 0.007 | 0.757 |  |  |  |
|  | rs821171 | C | T | 0.078 | 0.017 | 4.310E-06 | 0.003 | 0.008 | 0.729 |  |  |  |
|  | rs921925 | C | A | -0.098 | 0.020 | 8.150E-07 | -0.017 | 0.009 | 0.064 |  |  |  |
|  | rs9986520 | C | T | 0.131 | 0.030 | 8.040E-06 | 0.005 | 0.009 | 0.582 |  |  |  |
| *Methanobacteriaceae* | rs10424197 | A | G | 0.111 | 0.025 | 9.280E-06 | 0.000 | 0.007 | 0.988 |  |  |  |
|  | rs11018664 | A | G | 0.110 | 0.025 | 9.000E-06 | -0.031 | 0.011 | 0.005 | rs4753222 | A | G |
|  | rs11123059 | A | G | 0.108 | 0.023 | 4.870E-06 | -0.017 | 0.010 | 0.086 |  |  |  |
|  | rs12303159 | A | C | -0.223 | 0.049 | 8.560E-06 | 0.165 | 0.191 | 0.388 |  |  |  |
|  | rs62241826 | C | T | -0.190 | 0.041 | 6.940E-06 | 0.079 | 0.119 | 0.506 |  |  |  |
|  | rs6508769 | C | T | -0.154 | 0.034 | 8.230E-06 | -0.015 | 0.012 | 0.208 |  |  |  |
|  | rs73068003 | G | T | -0.158 | 0.035 | 8.440E-06 | 0.004 | 0.010 | 0.695 |  |  |  |
|  | rs894996 | A | C | -0.217 | 0.045 | 1.880E-06 | -0.004 | 0.016 | 0.803 |  |  |  |
|  | rs9532770 | C | T | 0.124 | 0.028 | 9.350E-06 | -0.003 | 0.008 | 0.732 |  |  |  |
| *Oxalobacteraceae* | rs111966731 | C | T | -0.200 | 0.044 | 6.140E-06 | 0.003 | 0.022 | 0.881 |  |  |  |
|  | rs11246212 | C | T | -0.131 | 0.029 | 8.200E-06 | -0.004 | 0.008 | 0.587 |  |  |  |
|  | rs17543178 | A | G | 0.142 | 0.028 | 4.770E-07 | -0.024 | 0.046 | 0.599 |  |  |  |
|  | rs28715741 | A | C | -0.109 | 0.025 | 8.930E-06 | -0.017 | 0.008 | 0.044 |  |  |  |
|  | rs36018452 | A | G | -0.180 | 0.040 | 7.810E-06 | 0.013 | 0.014 | 0.350 |  |  |  |
|  | rs4877289 | A | G | 0.088 | 0.020 | 7.350E-06 | -0.002 | 0.008 | 0.775 |  |  |  |
|  | rs6000536 | C | T | -0.117 | 0.024 | 8.470E-07 | 0.011 | 0.021 | 0.602 |  |  |  |
|  | rs7617946 | C | T | 0.098 | 0.022 | 7.010E-06 | 0.019 | 0.007 | 0.006 |  |  |  |
|  | rs7993559 | A | C | -0.092 | 0.020 | 4.030E-06 | -0.007 | 0.007 | 0.324 |  |  |  |
|  | rs9928128 | C | T | 0.088 | 0.020 | 9.550E-06 | 0.001 | 0.007 | 0.846 |  |  |  |
| *Pasteurellaceae* | rs10123426 | A | G | 0.127 | 0.029 | 7.420E-06 | -0.014 | 0.011 | 0.201 |  |  |  |
|  | rs10965428 | A | C | 0.118 | 0.026 | 5.750E-06 | 0.019 | 0.018 | 0.275 |  |  |  |
|  | rs12050685 | A | G | -0.067 | 0.015 | 8.070E-06 | 0.007 | 0.012 | 0.552 |  |  |  |
|  | rs16970009 | A | G | 0.187 | 0.043 | 7.320E-06 | 0.000 | 0.020 | 0.992 |  |  |  |
|  | rs40513 | T | G | -0.117 | 0.026 | 6.890E-06 | 0.001 | 0.014 | 0.931 |  |  |  |
|  | rs4822728 | C | T | -0.069 | 0.015 | 4.050E-06 | -0.010 | 0.007 | 0.132 |  |  |  |
|  | rs6972479 | A | G | -0.077 | 0.017 | 8.880E-06 | -0.032 | 0.027 | 0.228 |  |  |  |
|  | rs76022354 | C | T | 0.243 | 0.050 | 1.830E-06 | -0.030 | 0.025 | 0.224 |  |  |  |
|  | rs9382510 | C | T | -0.085 | 0.017 | 5.100E-07 | -0.003 | 0.007 | 0.622 |  |  |  |
|  | rs9402707 | C | T | -0.074 | 0.016 | 8.730E-06 | 0.000 | 0.010 | 0.992 |  |  |  |
|  | rs9895850 | C | T | 0.176 | 0.041 | 9.080E-06 | 0.002 | 0.011 | 0.843 |  |  |  |
| *Peptococcaceae* | rs11136654 | G | T | 0.089 | 0.018 | 2.590E-06 | 0.002 | 0.008 | 0.844 |  |  |  |
|  | rs12144792 | C | T | 0.064 | 0.014 | 6.140E-06 | 0.008 | 0.011 | 0.449 |  |  |  |
|  | rs12634826 | G | T | 0.072 | 0.015 | 1.680E-06 | 0.005 | 0.008 | 0.515 |  |  |  |
|  | rs12992764 | G | T | -0.067 | 0.014 | 2.040E-06 | 0.004 | 0.007 | 0.554 |  |  |  |
|  | rs28584387 | C | T | -0.067 | 0.015 | 7.960E-06 | -0.003 | 0.010 | 0.760 |  |  |  |
|  | rs35703006 | G | T | 0.082 | 0.016 | 3.820E-07 | 0.007 | 0.007 | 0.304 |  |  |  |
|  | rs61870295 | A | G | -0.136 | 0.029 | 1.780E-06 | 0.119 | 0.107 | 0.268 |  |  |  |
|  | rs75898026 | A | G | -0.081 | 0.017 | 2.640E-06 | 0.010 | 0.011 | 0.351 |  |  |  |
| *Peptostreptococcaceae* | rs10805326 | A | G | -0.056 | 0.012 | 5.440E-06 | 0.024 | 0.013 | 0.053 |  |  |  |
|  | rs11149051 | C | T | 0.064 | 0.014 | 8.170E-06 | 0.001 | 0.008 | 0.910 |  |  |  |
|  | rs117644882 | C | T | -0.170 | 0.038 | 7.760E-06 | -0.050 | 0.059 | 0.403 |  |  |  |
|  | rs12993096 | C | T | 0.057 | 0.013 | 6.180E-06 | -0.002 | 0.007 | 0.735 |  |  |  |
|  | rs1467258 | A | G | -0.073 | 0.016 | 7.120E-06 | 0.000 | 0.010 | 0.967 |  |  |  |
|  | rs17661170 | A | G | -0.063 | 0.013 | 5.700E-07 | -0.014 | 0.007 | 0.045 |  |  |  |
|  | rs2668550 | T | C | -0.064 | 0.014 | 9.870E-06 | -0.001 | 0.008 | 0.912 |  |  |  |
|  | rs2883972 | A | G | -0.070 | 0.015 | 3.120E-06 | -0.009 | 0.022 | 0.677 |  |  |  |
|  | rs59865771 | C | T | -0.057 | 0.013 | 7.210E-06 | -0.010 | 0.008 | 0.242 |  |  |  |
|  | rs59987323 | C | T | -0.055 | 0.011 | 1.610E-06 | 0.000 | 0.009 | 0.972 |  |  |  |
|  | rs77540684 | G | T | -0.106 | 0.024 | 7.730E-06 | 0.010 | 0.012 | 0.407 |  |  |  |
|  | rs77702691 | A | G | -0.087 | 0.020 | 9.670E-06 | -0.035 | 0.044 | 0.422 |  |  |  |
| *Porphyromonadaceae* | rs1029811 | T | G | 0.068 | 0.015 | 2.360E-06 | 0.008 | 0.013 | 0.515 |  |  |  |
|  | rs10762312 | A | G | 0.052 | 0.012 | 9.220E-06 | 0.002 | 0.007 | 0.754 |  |  |  |
|  | rs10858364 | G | T | 0.056 | 0.012 | 3.280E-06 | -0.001 | 0.009 | 0.889 |  |  |  |
|  | rs1125465 | T | C | -0.060 | 0.012 | 1.310E-06 | 0.003 | 0.008 | 0.739 |  |  |  |
|  | rs12700163 | C | T | 0.053 | 0.012 | 7.590E-06 | -0.017 | 0.014 | 0.240 |  |  |  |
|  | rs2675411 | G | A | -0.054 | 0.012 | 8.440E-06 | 0.001 | 0.007 | 0.918 |  |  |  |
|  | rs35233670 | C | T | 0.047 | 0.011 | 9.560E-06 | -0.008 | 0.007 | 0.267 |  |  |  |
|  | rs35961441 | A | C | 0.091 | 0.021 | 8.160E-06 | 0.011 | 0.029 | 0.710 |  |  |  |
|  | rs7038649 | C | T | -0.058 | 0.013 | 7.670E-06 | 0.005 | 0.008 | 0.497 |  |  |  |
|  | rs7330827 | C | T | 0.104 | 0.024 | 8.040E-06 | -0.017 | 0.022 | 0.439 | rs117538637 | G | T |
| *Prevotellaceae* | rs11102139 | C | T | 0.081 | 0.017 | 1.200E-06 | -0.011 | 0.007 | 0.137 |  |  |  |
|  | rs11151191 | C | T | -0.077 | 0.017 | 7.260E-06 | -0.006 | 0.018 | 0.749 |  |  |  |
|  | rs12057990 | C | T | 0.061 | 0.013 | 4.170E-06 | 0.001 | 0.007 | 0.918 |  |  |  |
|  | rs12873417 | C | T | 0.059 | 0.013 | 2.980E-06 | -0.009 | 0.010 | 0.387 |  |  |  |
|  | rs13069367 | A | C | -0.053 | 0.012 | 9.200E-06 | 0.001 | 0.007 | 0.868 |  |  |  |
|  | rs2206482 | T | G | -0.056 | 0.012 | 1.590E-06 | 0.002 | 0.007 | 0.824 |  |  |  |
|  | rs34660375 | A | G | -0.081 | 0.018 | 6.330E-06 | 0.007 | 0.011 | 0.530 |  |  |  |
|  | rs3758087 | T | C | 0.057 | 0.012 | 6.710E-06 | -0.007 | 0.008 | 0.399 |  |  |  |
|  | rs4685827 | C | T | 0.066 | 0.014 | 5.430E-06 | -0.004 | 0.009 | 0.638 |  |  |  |
|  | rs6720263 | A | G | 0.059 | 0.012 | 5.300E-07 | 0.000 | 0.007 | 0.964 |  |  |  |
|  | rs7252711 | A | G | -0.073 | 0.016 | 6.590E-06 | -0.004 | 0.008 | 0.662 |  |  |  |
|  | rs77380553 | A | G | -0.075 | 0.017 | 5.650E-06 | -0.018 | 0.026 | 0.482 |  |  |  |
|  | rs912860 | G | A | -0.229 | 0.048 | 9.300E-07 | -0.003 | 0.011 | 0.772 |  |  |  |
| *Rhodospirillaceae* | rs1035406 | A | G | 0.113 | 0.025 | 6.380E-06 | 0.003 | 0.008 | 0.739 |  |  |  |
|  | rs11630875 | C | T | -0.093 | 0.020 | 5.490E-06 | 0.002 | 0.008 | 0.832 |  |  |  |
|  | rs2162883 | C | T | 0.088 | 0.019 | 8.040E-06 | -0.001 | 0.007 | 0.929 |  |  |  |
|  | rs34142542 | C | T | -0.070 | 0.016 | 8.090E-06 | -0.010 | 0.022 | 0.656 |  |  |  |
|  | rs3768869 | A | G | -0.092 | 0.020 | 6.730E-06 | -0.012 | 0.007 | 0.085 |  |  |  |
|  | rs55661963 | A | C | -0.129 | 0.029 | 5.490E-06 | 0.016 | 0.049 | 0.749 |  |  |  |
|  | rs62255666 | A | G | -0.082 | 0.018 | 4.620E-06 | -0.002 | 0.007 | 0.778 |  |  |  |
|  | rs72714493 | A | G | 0.082 | 0.018 | 6.760E-06 | -0.016 | 0.014 | 0.227 |  |  |  |
|  | rs7623329 | A | C | -0.070 | 0.016 | 9.350E-06 | -0.005 | 0.007 | 0.511 |  |  |  |
| *Rikenellaceae* | rs11841382 | G | T | 0.077 | 0.018 | 7.530E-06 | -0.001 | 0.007 | 0.873 |  |  |  |
|  | rs1939881 | G | A | -0.104 | 0.021 | 8.770E-07 | 0.011 | 0.007 | 0.123 |  |  |  |
|  | rs2290844 | C | T | 0.083 | 0.019 | 6.530E-06 | 0.008 | 0.009 | 0.351 |  |  |  |
|  | rs2447496 | G | A | -0.055 | 0.012 | 6.480E-06 | 0.004 | 0.008 | 0.577 |  |  |  |
|  | rs2833282 | A | G | -0.071 | 0.016 | 4.320E-06 | 0.008 | 0.010 | 0.443 |  |  |  |
|  | rs4264350 | C | T | 0.052 | 0.011 | 1.330E-06 | 0.006 | 0.007 | 0.402 |  |  |  |
|  | rs58174947 | A | G | -0.048 | 0.011 | 9.540E-06 | 0.008 | 0.008 | 0.311 |  |  |  |
|  | rs59663348 | A | G | -0.057 | 0.012 | 5.060E-06 | 0.011 | 0.007 | 0.113 |  |  |  |
|  | rs61978199 | C | T | -0.147 | 0.034 | 8.690E-06 | 0.008 | 0.026 | 0.765 |  |  |  |
|  | rs62532512 | A | C | 0.051 | 0.011 | 2.250E-06 | -0.011 | 0.007 | 0.117 |  |  |  |
|  | rs67705352 | G | T | 0.054 | 0.011 | 9.270E-07 | 0.001 | 0.007 | 0.913 |  |  |  |
|  | rs6837275 | A | G | 0.055 | 0.012 | 2.660E-06 | -0.005 | 0.007 | 0.502 |  |  |  |
|  | rs7242694 | C | T | -0.062 | 0.013 | 5.190E-06 | -0.002 | 0.007 | 0.767 |  |  |  |
|  | rs73465767 | C | T | 0.086 | 0.020 | 9.580E-06 | -0.041 | 0.013 | 0.001 |  |  |  |
|  | rs7832304 | G | T | 0.073 | 0.016 | 7.860E-06 | 0.029 | 0.017 | 0.088 |  |  |  |
|  | rs9578457 | A | G | 0.141 | 0.032 | 3.990E-06 | 0.004 | 0.063 | 0.954 |  |  |  |
| *Ruminococcaceae* | rs10093275 | C | T | 0.054 | 0.012 | 4.680E-06 | -0.013 | 0.008 | 0.096 |  |  |  |
|  | rs10172096 | A | G | 0.160 | 0.035 | 3.510E-06 | 0.007 | 0.011 | 0.507 |  |  |  |
|  | rs1572312 | T | G | 0.083 | 0.017 | 1.180E-06 | -0.013 | 0.016 | 0.419 |  |  |  |
|  | rs56199908 | C | T | 0.199 | 0.041 | 1.660E-06 | -0.019 | 0.020 | 0.339 |  |  |  |
| *Streptococcaceae* | rs111893872 | C | T | -0.069 | 0.015 | 9.390E-06 | -0.026 | 0.034 | 0.446 |  |  |  |
|  | rs13379080 | C | T | -0.084 | 0.018 | 4.570E-06 | 0.005 | 0.013 | 0.711 |  |  |  |
|  | rs17613937 | A | G | -0.088 | 0.019 | 9.130E-06 | 0.013 | 0.009 | 0.138 |  |  |  |
|  | rs1964822 | T | C | -0.054 | 0.012 | 9.220E-06 | 0.002 | 0.008 | 0.758 |  |  |  |
|  | rs2952189 | T | C | -0.070 | 0.015 | 3.510E-06 | 0.001 | 0.015 | 0.937 |  |  |  |
|  | rs34070557 | C | T | 0.049 | 0.011 | 8.650E-06 | -0.001 | 0.009 | 0.886 |  |  |  |
|  | rs77968078 | A | G | 0.099 | 0.022 | 7.970E-06 | 0.015 | 0.011 | 0.166 |  |  |  |
|  | rs7916711 | A | G | 0.094 | 0.021 | 9.040E-06 | 0.009 | 0.007 | 0.199 |  |  |  |
| *Veillonellaceae* | rs114889439 | A | G | -0.254 | 0.054 | 6.180E-06 | 0.179 | 0.056 | 0.001 |  |  |  |
|  | rs11700976 | A | C | -0.050 | 0.011 | 8.030E-06 | 0.006 | 0.022 | 0.774 |  |  |  |
|  | rs12668619 | A | G | 0.054 | 0.012 | 3.570E-06 | -0.016 | 0.008 | 0.043 |  |  |  |
|  | rs1442060 | A | G | 0.051 | 0.011 | 5.720E-06 | 0.005 | 0.007 | 0.514 |  |  |  |
|  | rs2036713 | C | A | -0.051 | 0.011 | 6.830E-06 | -0.007 | 0.007 | 0.282 |  |  |  |
|  | rs2561116 | T | G | -0.084 | 0.019 | 6.510E-06 | 0.010 | 0.010 | 0.307 |  |  |  |
|  | rs2585520 | T | G | 0.088 | 0.020 | 8.370E-06 | -0.002 | 0.008 | 0.817 |  |  |  |
|  | rs4461038 | A | G | -0.054 | 0.012 | 6.270E-06 | 0.015 | 0.010 | 0.143 |  |  |  |
|  | rs4736971 | A | C | -0.052 | 0.012 | 6.230E-06 | 0.019 | 0.008 | 0.023 |  |  |  |
|  | rs4797169 | C | T | -0.060 | 0.013 | 3.070E-06 | 0.008 | 0.008 | 0.305 |  |  |  |
|  | rs66716149 | C | T | -0.057 | 0.012 | 1.360E-06 | -0.071 | 0.057 | 0.208 |  |  |  |
|  | rs6692542 | A | G | 0.053 | 0.012 | 8.300E-06 | -0.022 | 0.013 | 0.081 |  |  |  |
|  | rs6909981 | C | T | -0.063 | 0.014 | 6.310E-06 | -0.010 | 0.009 | 0.260 |  |  |  |
|  | rs73557284 | A | G | 0.205 | 0.047 | 7.160E-06 | -0.003 | 0.009 | 0.719 |  |  |  |
|  | rs79535861 | A | C | 0.104 | 0.021 | 6.440E-07 | -0.022 | 0.022 | 0.317 |  |  |  |
|  | rs9345168 | A | C | -0.051 | 0.011 | 7.370E-06 | -0.005 | 0.011 | 0.630 |  |  |  |
| *Verrucomicrobiaceae* | rs12908520 | A | G | -0.059 | 0.013 | 5.840E-06 | -0.004 | 0.007 | 0.541 |  |  |  |
|  | rs2602429 | C | T | 0.074 | 0.016 | 3.170E-06 | 0.019 | 0.007 | 0.005 |  |  |  |
|  | rs4242783 | A | G | -0.068 | 0.015 | 3.310E-06 | 0.005 | 0.008 | 0.494 |  |  |  |
|  | rs4294419 | A | G | 0.064 | 0.014 | 4.800E-06 | 0.005 | 0.007 | 0.466 |  |  |  |
|  | rs4936098 | A | G | 0.066 | 0.014 | 6.830E-07 | 0.009 | 0.016 | 0.547 |  |  |  |
|  | rs61779206 | C | T | 0.076 | 0.017 | 5.640E-06 | 0.002 | 0.024 | 0.935 |  |  |  |
|  | rs72663744 | C | T | -0.064 | 0.015 | 9.820E-06 | -0.004 | 0.008 | 0.617 |  |  |  |
|  | rs9349825 | A | G | -0.071 | 0.015 | 1.700E-06 | 0.018 | 0.008 | 0.025 |  |  |  |

Abbreviations: SNP, single nucleotide polymorphism; IVs, instrumental variables; T2DM, type 2 diabetes mellitus; GWAS, genome-wide association study.
