# Supplementary material for: Genetically Predicted Causality of 28 Gut Microbiome Families and Type 2 Diabetes Mellitus Risk
Source: Front Endocrinol (Lausanne). 2022 Feb 3;13:780133. doi: 10.3389/fendo.2022.780133 (PMC8851667; doi:10.3389/fendo.2022.780133)
Supplement: Supplementary file 5 [file Table_3.docx]

| **\Supplementary Table 3. MR estimates of IVs for gut microbiome and T2DM (European)** | | | | | | | | | | | | | | | | | |
| --- | --- | --- | --- | --- | --- | --- | --- | --- | --- | --- | --- | --- | --- | --- | --- | --- | --- |
| **Exposure** | **Nsnp** | **Methods** | **Beta** | **SE** | **OR (95% CI)** | ***P* value** | **FDR *P* value** | **Horizontal pleiotropy** | | | | | | | **Heterogeneity** | | ***F* statistic** |
|  |  |  |  |  |  |  |  | **MR-Egger regression** | | | **MR-PRESSO** | | | | **Cochran’s *Q*** | ***P* value** |  |
|  |  |  |  |  |  |  |  | **Egger intercept** | **SE** | ***P* value** | **Global test *P* value** | **Outliers** | **OR (95% CI)** | ***P* value** |  |  |  |
| *Acidaminococcaceae* | 3^a^ | IVW | -0.06 | 0.12 | 0.94 (0.75-1.19) | 0.614 | 0.962 | 0.02 | 0.04 | 0.632 | - | - | - | - | 5.92 | 0.052 | 24.03 |
|  |  | MR Egger | -0.30 | 0.40 | 0.74 (0.34-1.61) | 0.586 | 0.965 |  |  |  |  |  |  |  |  |  |  |
|  |  | Weighted median | -0.12 | 0.10 | 0.88 (0.73-1.07) | 0.197 | 0.862 |  |  |  |  |  |  |  |  |  |  |
| *Actinomycetaceae* | 4 | IVW | 0.07 | 0.05 | 1.07 (0.97-1.19) | 0.191 | 0.962 | -0.02 | 0.03 | 0.479 | 0.685 | - | - | - | 1.53 | 0.676 | 20.95 |
|  |  | MR Egger | 0.32 | 0.29 | 1.37 (0.78-2.44) | 0.390 | 0.965 |  |  |  |  |  |  |  |  |  |  |
|  |  | Weighted median | 0.05 | 0.07 | 1.05 (0.92-1.20) | 0.493 | 0.862 |  |  |  |  |  |  |  |  |  |  |
| *Alcaligenaceae* | 12 | IVW | -0.03 | 0.06 | 0.97 (0.86-1.09) | 0.574 | 0.962 | -0.01 | 0.01 | 0.484 | 0.066 | - | - | - | 19.83 | 0.048 | 28.68 |
|  |  | MR Egger | 0.14 | 0.24 | 1.15 (0.71-1.84) | 0.585 | 0.965 |  |  |  |  |  |  |  |  |  |  |
|  |  | Weighted median | -0.04 | 0.07 | 0.97 (0.85-1.10) | 0.596 | 0.862 |  |  |  |  |  |  |  |  |  |  |
| *Bacteroidaceae* | 8 | IVW | -0.02 | 0.06 | 0.98 (0.88-1.10) | 0.766 | 0.962 | 0.02 | 0.02 | 0.220 | 0.610 | - | - | - | 5.43 | 0.608 | 26.95 |
|  |  | MR Egger | -0.35 | 0.25 | 0.70 (0.43-1.15) | 0.211 | 0.965 |  |  |  |  |  |  |  |  |  |  |
|  |  | Weighted median | -0.03 | 0.07 | 0.97 (0.84-1.12) | 0.684 | 0.862 |  |  |  |  |  |  |  |  |  |  |
| *Bacteroidales_S24-7* | 6 | IVW | 0.03 | 0.04 | 1.03 (0.95-1.12) | 0.461 | 0.962 | 0.01 | 0.02 | 0.557 | 0.874 | - | - | - | 1.78 | 0.878 | 25.29 |
|  |  | MR Egger | -0.06 | 0.15 | 0.94 (0.69-1.27) | 0.704 | 0.965 |  |  |  |  |  |  |  |  |  |  |
|  |  | Weighted median | 0.04 | 0.05 | 1.04 (0.94-1.15) | 0.493 | 0.862 |  |  |  |  |  |  |  |  |  |  |
| *Bifidobacteriaceae* | 7 | IVW | 0.01 | 0.06 | 1.01 (0.91-1.13) | 0.832 | 0.962 | -0.02 | 0.02 | 0.431 | 0.589 | - | - | - | 4.72 | 0.581 | 34.12 |
|  |  | MR Egger | 0.37 | 0.42 | 1.44 (0.64-3.26) | 0.420 | 0.965 |  |  |  |  |  |  |  |  |  |  |
|  |  | Weighted median | -0.02 | 0.07 | 0.98 (0.85-1.13) | 0.800 | 0.862 |  |  |  |  |  |  |  |  |  |  |
| *Christensenellaceae* | 8 | IVW | 0.05 | 0.10 | 1.05 (0.86-1.29) | 0.621 | 0.962 | 0.01 | 0.02 | 0.689 | 0.004 | rs7211194 | 0.99 (0.82-1.21) | 0.958 | 23.43 | 0.001 | 22.78 |
|  |  | MR Egger | -0.13 | 0.43 | 0.88 (0.38-2.07) | 0.781 | 0.965 |  |  |  |  |  | 0.98 (0.43-2.21) | 0.955 |  |  |  |
|  |  | Weighted median | -0.03 | 0.10 | 0.97 (0.81-1.17) | 0.783 | 0.862 |  |  |  |  |  | 0.92 (0.76-1.12) | 0.410 |  |  |  |
| *Clostridiaceae_1* | 7 | IVW | -0.06 | 0.07 | 0.94 (0.83-1.07) | 0.343 | 0.962 | 0.00 | 0.02 | 0.921 | 0.184 | - | - | - | 9.92 | 0.128 | 22.44 |
|  |  | MR Egger | -0.08 | 0.20 | 0.92 (0.62-1.36) | 0.698 | 0.965 |  |  |  |  |  |  |  |  |  |  |
|  |  | Weighted median | -0.12 | 0.07 | 0.89 (0.77-1.02) | 0.100 | 0.700 |  |  |  |  |  |  |  |  |  |  |
| *Coriobacteriaceae* | 5 | IVW | 0.02 | 0.07 | 1.02 (0.89-1.17) | 0.747 | 0.962 | -0.01 | 0.02 | 0.758 | 0.923 | - | - | - | 0.89 | 0.926 | 29.83 |
|  |  | MR Egger | 0.12 | 0.30 | 1.13 (0.62-2.04) | 0.714 | 0.965 |  |  |  |  |  |  |  |  |  |  |
|  |  | Weighted median | 0.02 | 0.09 | 1.02 (0.86-1.21) | 0.788 | 0.862 |  |  |  |  |  |  |  |  |  |  |
| *Defluviitaleaceae* | 6 | IVW | 0.02 | 0.05 | 1.02 (0.93-1.12) | 0.673 | 0.962 | 0.00 | 0.02 | 0.951 | 0.353 | - | - | - | 5.74 | 0.333 | 24.69 |
|  |  | MR Egger | 0.01 | 0.19 | 1.01 (0.69-1.47) | 0.965 | 0.965 |  |  |  |  |  |  |  |  |  |  |
|  |  | Weighted median | -0.02 | 0.06 | 0.98 (0.87-1.10) | 0.741 | 0.862 |  |  |  |  |  |  |  |  |  |  |
| *Desulfovibrionaceae* | 9 | IVW | 0.10 | 0.06 | 1.11 (0.99-1.25) | 0.084 | 0.962 | 0.00 | 0.01 | 0.794 | 0.110 | - | - | - | 14.40 | 0.072 | 29.69 |
|  |  | MR Egger | 0.14 | 0.16 | 1.16 (0.84-1.59) | 0.403 | 0.965 |  |  |  |  |  |  |  |  |  |  |
|  |  | Weighted median | 0.14 | 0.06 | 1.15 (1.01-1.31) | 0.032 | 0.663 |  |  |  |  |  |  |  |  |  |  |
| *Enterobacteriaceae* | 6 | IVW | 0.12 | 0.09 | 1.13 (0.94-1.35) | 0.191 | 0.962 | -0.02 | 0.05 | 0.713 | 0.041 | rs922773 | 1.03 (0.91-1.16) | 0.668 | 12.74 | 0.026 | 27.63 |
|  |  | MR Egger | 0.38 | 0.66 | 1.46 (0.40-5.32) | 0.599 | 0.965 |  |  |  |  |  | 0.97 (0.44-2.12) | 0.943 |  |  |  |
|  |  | Weighted median | 0.06 | 0.08 | 1.07 (0.92-1.24) | 0.408 | 0.862 |  |  |  |  |  | 1.06 (0.91-1.23) | 0.489 |  |  |  |
| *Erysipelotrichaceae* | 7 | IVW | -0.01 | 0.08 | 0.99 (0.85-1.16) | 0.907 | 0.962 | -0.01 | 0.03 | 0.829 | 0.149 | - | - | - | 9.69 | 0.138 | 23.98 |
|  |  | MR Egger | 0.09 | 0.43 | 1.09 (0.47-2.56) | 0.848 | 0.965 |  |  |  |  |  |  |  |  |  |  |
|  |  | Weighted median | 0.03 | 0.09 | 1.03 (0.86-1.23) | 0.777 | 0.862 |  |  |  |  |  |  |  |  |  |  |
| *Lachnospiraceae* | 9 | IVW | -0.03 | 0.05 | 0.97 (0.88-1.08) | 0.578 | 0.962 | -0.01 | 0.01 | 0.605 | 0.480 | - | - | - | 7.91 | 0.442 | 29.68 |
|  |  | MR Egger | 0.04 | 0.14 | 1.04 (0.79-1.38) | 0.773 | 0.965 |  |  |  |  |  |  |  |  |  |  |
|  |  | Weighted median | 0.00 | 0.07 | 1.00 (0.87-1.14) | 0.955 | 0.955 |  |  |  |  |  |  |  |  |  |  |
| *Lactobacillaceae* | 6 | IVW | -0.02 | 0.04 | 0.98 (0.90-1.06) | 0.579 | 0.962 | 0.01 | 0.01 | 0.617 | 0.838 | - | - | - | 2.07 | 0.839 | 25.79 |
|  |  | MR Egger | -0.08 | 0.12 | 0.92 (0.72-1.17) | 0.525 | 0.965 |  |  |  |  |  |  |  |  |  |  |
|  |  | Weighted median | -0.02 | 0.05 | 0.98 (0.89-1.07) | 0.629 | 0.862 |  |  |  |  |  |  |  |  |  |  |
| *Methanobacteriaceae* | 6 | IVW | -0.03 | 0.05 | 0.97 (0.88- 1.06) | 0.469 | 0.770 | 0.02 | 0.02 | 0.437 | 0.046 | rs11123059 | 0.93 (0.88- 0.99) | 0.029 | 12.64 | 0.027 | 20.62 |
|  |  | MR Egger | -0.16 | 0.16 | 0.85 (0.62-1.16) | 0.360 | 0.965 |  |  |  |  |  | 0.95 (0.75-1.22) | 0.722 |  |  |  |
|  |  | Weighted median | -0.02 | 0.04 | 0.98 (0.91- 1.06) | 0.603 | 0.862 |  |  |  |  |  | 0.98 (0.90- 1.06) | 0.607 |  |  |  |
| *Oxalobacteraceae* | 9 | IVW | -0.02 | 0.03 | 0.98 (0.92-1.04) | 0.458 | 0.962 | 0.02 | 0.01 | 0.162 | 0.411 | - | - | - | 8.67 | 0.371 | 21.99 |
|  |  | MR Egger | -0.15 | 0.09 | 0.86 (0.72-1.02) | 0.127 | 0.965 |  |  |  |  |  |  |  |  |  |  |
|  |  | Weighted median | -0.03 | 0.04 | 0.97 (0.89-1.04) | 0.392 | 0.862 |  |  |  |  |  |  |  |  |  |  |
| *Pasteurellaceae* | 7 | IVW | 0.02 | 0.06 | 1.02 (0.90-1.15) | 0.736 | 0.962 | 0.01 | 0.02 | 0.741 | 0.077 | - | - | - | 12.90 | 0.045 | 32.28 |
|  |  | MR Egger | -0.04 | 0.19 | 0.96 (0.67-1.39) | 0.839 | 0.965 |  |  |  |  |  |  |  |  |  |  |
|  |  | Weighted median | 0.07 | 0.06 | 1.07 (0.95-1.21) | 0.279 | 0.862 |  |  |  |  |  |  |  |  |  |  |
| *Peptococcaceae* | 4 | IVW | 0.02 | 0.06 | 1.02 (0.91-1.14) | 0.762 | 0.962 | -0.01 | 0.02 | 0.703 | 0.811 | - | - | - | 0.99 | 0.804 | 27.60 |
|  |  | MR Egger | 0.10 | 0.20 | 1.11 (0.75-1.63) | 0.662 | 0.965 |  |  |  |  |  |  |  |  |  |  |
|  |  | Weighted median | 0.03 | 0.07 | 1.03 (0.90-1.17) | 0.702 | 0.862 |  |  |  |  |  |  |  |  |  |  |
| *Peptostreptococcaceae* | 9 | IVW | 0.00 | 0.06 | 1.00 (0.89-1.13) | 0.951 | 0.962 | 0.01 | 0.01 | 0.506 | 0.043 | rs12377846 | 1.07 (0.94-1.21) | 0.293 | 16.27 | 0.039 | 32.64 |
|  |  | MR Egger | -0.08 | 0.13 | 0.93 (0.72-1.19) | 0.575 | 0.965 |  |  |  |  |  | 1.23 (0.83-1.83) | 0.344 |  |  |  |
|  |  | Weighted median | -0.09 | 0.06 | 0.91 (0.80-1.03) | 0.143 | 0.862 |  |  |  |  |  | 1.07 (0.92-1.24) | 0.396 |  |  |  |
| *Porphyromonadaceae* | 7 | IVW | -0.09 | 0.06 | 0.91 (0.81-1.03) | 0.144 | 0.962 | -0.02 | 0.02 | 0.317 | 0.939 | - | - | - | 1.98 | 0.921 | 36.25 |
|  |  | MR Egger | 0.27 | 0.33 | 1.31 (0.69-2.51) | 0.448 | 0.965 |  |  |  |  |  |  |  |  |  |  |
|  |  | Weighted median | -0.08 | 0.07 | 0.92 (0.80-1.07) | 0.287 | 0.862 |  |  |  |  |  |  |  |  |  |  |
| *Prevotellaceae* | 12 | IVW | 0.04 | 0.04 | 1.04 (0.96-1.12) | 0.381 | 0.962 | 0.00 | 0.01 | 0.937 | 0.683 | - | - | - | 8.37 | 0.679 | 33.22 |
|  |  | MR Egger | 0.05 | 0.14 | 1.05 (0.80-1.37) | 0.744 | 0.965 |  |  |  |  |  |  |  |  |  |  |
|  |  | Weighted median | 0.05 | 0.05 | 1.05 (0.95-1.17) | 0.354 | 0.862 |  |  |  |  |  |  |  |  |  |  |
| *Rhodospirillaceae* | 9 | IVW | -0.03 | 0.04 | 0.98 (0.91-1.05) | 0.507 | 0.962 | 0.00 | 0.02 | 0.872 | 0.749 | - | - | - | 4.89 | 0.769 | 30.15 |
|  |  | MR Egger | 0.01 | 0.22 | 1.01 (0.66-1.55) | 0.962 | 0.965 |  |  |  |  |  |  |  |  |  |  |
|  |  | Weighted median | -0.04 | 0.05 | 0.96 (0.87-1.06) | 0.461 | 0.862 |  |  |  |  |  |  |  |  |  |  |
| *Rikenellaceae* | 13 | IVW | 0.02 | 0.04 | 1.02 (0.94-1.11) | 0.654 | 0.962 | 0.01 | 0.01 | 0.153 | 0.637 | - | - | - | 9.75 | 0.638 | 22.87 |
|  |  | MR Egger | -0.16 | 0.13 | 0.85 (0.66-1.09) | 0.224 | 0.965 |  |  |  |  |  |  |  |  |  |  |
|  |  | Weighted median | 0.03 | 0.06 | 1.03 (0.91-1.16) | 0.655 | 0.862 |  |  |  |  |  |  |  |  |  |  |
| *Ruminococcaceae* | 3^a^ | IVW | 0.01 | 0.08 | 1.01 (0.86-1.18) | 0.948 | 0.962 | 0.00 | 0.02 | 0.847 | - | - | - | - | 0.06 | 0.971 | 40.07 |
|  |  | MR Egger | 0.05 | 0.20 | 1.05 (0.71-1.55) | 0.844 | 0.965 |  |  |  |  |  |  |  |  |  |  |
|  |  | Weighted median | 0.01 | 0.09 | 1.01 (0.84-1.21) | 0.937 | 0.955 |  |  |  |  |  |  |  |  |  |  |
| *Streptococcaceae* | 9 | IVW | 0.15 | 0.06 | 1.17 (1.04-1.31) | 0.009 | 0.962 | 0.01 | 0.02 | 0.492 | 0.331 | - | - | - | 9.92 | 0.270 | 20.30 |
|  |  | MR Egger | -0.02 | 0.24 | 0.98 (0.61-1.58) | 0.948 | 0.965 |  |  |  |  |  |  |  |  |  |  |
|  |  | Weighted median | 0.14 | 0.08 | 1.15 (0.99-1.34) | 0.071 | 0.663 |  |  |  |  |  |  |  |  |  |  |
| *Veillonellaceae* | 12 | IVW | 0.01 | 0.04 | 1.01 (0.92-1.10) | 0.882 | 0.962 | 0.00 | 0.01 | 0.825 | 0.987 | - | - | - | 3.61 | 0.980 | 26.56 |
|  |  | MR Egger | -0.04 | 0.20 | 0.96 (0.65-1.43) | 0.853 | 0.965 |  |  |  |  |  |  |  |  |  |  |
|  |  | Weighted median | -0.02 | 0.05 | 0.98 (0.88-1.09) | 0.700 | 0.862 |  |  |  |  |  |  |  |  |  |  |
| *Verrucomicrobiaceae* | 6 | IVW | 0.03 | 0.09 | 1.03 (0.86-1.24) | 0.749 | 0.770 | 0.01 | 0.09 | 0.891 | 0.020 | rs72663744 | 1.11 (0.99-1.24) | 0.055 | 15.83 | 0.007 | 21.69 |
|  |  | MR Egger | -0.17 | 1.37 | 0.84 (0.06-12.37) | 0.907 | 0.965 |  |  |  |  |  | 0.49 (0.12-1.96) | 0.389 |  |  |  |
|  |  | Weighted median | 0.12 | 0.07 | 1.12 (0.97-1.30) | 0.114 | 0.663 |  |  |  |  |  | 1.15 (0.99-1.33) | 0.059 |  |  |  |
| a. Not enough SNPs for MR-PRESSO analysis  Abbreviations: MR, Mendelian randomization; SNP, single nucleotide polymorphism; IVW, inverse variance weighted; IVs, instrumental variables; FDR, false discovery rate; T2DM, type 2 diabetes mellitus; OR, odds ratio; MR-PRESSO, Mendelian randomization pleiotropy residual sum and outlier. | | | | | | | | | | | | | | | | | |
