# Supplementary material for: Genetically Predicted Causality of 28 Gut Microbiome Families and Type 2 Diabetes Mellitus Risk
Source: Front Endocrinol (Lausanne). 2022 Feb 3;13:780133. doi: 10.3389/fendo.2022.780133 (PMC8851667; doi:10.3389/fendo.2022.780133)
Supplement: Supplementary file 6 [file Table_4.docx]

| **Supplementary Table 4. MR estimates of IVs for gut microbiome and T2DM (Asian)** | | | | | | | | | | | | | | | | | |
| --- | --- | --- | --- | --- | --- | --- | --- | --- | --- | --- | --- | --- | --- | --- | --- | --- | --- |
| **Exposure** | **Nsnp** | **Methods** | **Beta** | **SE** | **OR (95% CI)** | ***P* value** | **FDR *P* value** | **Horizontal pleiotropy** | | | | | | | **Heterogeneity** | | ***F* statistic** |
|  |  |  |  |  |  |  |  | **MR-Egger regression** | | | **MR-PRESSO** | | | | **Cochran’s *Q*** | ***P* value** |  |
|  |  |  |  |  |  |  |  | **Egger intercept** | **SE** | ***P* value** | **Global test *P* value** | **Outliers** | **OR (95% CI)** | ***P* value** |  |  |  |
| *Acidaminococcaceae* | 3^a^ | IVW | 0.16 | 0.06 | 1.17 (1.04-1.31) | 0.008 | 0.224 | -0.01 | 0.01 | 0.593 | - | - | - | - | 0.57 | 0.751 | 29.82 |
|  |  | MR Egger | 0.25 | 0.14 | 1.28 (0.98-1.67) | 0.322 | 0.939 |  |  |  |  |  |  |  |  |  |  |
|  |  | Weighted median | 0.15 | 0.08 | 1.16 (0.99-1.35) | 0.051 | 0.607 |  |  |  |  |  |  |  |  |  |  |
| *Actinomycetaceae* | 3^a^ | IVW | 0.02 | 0.09 | 1.02 (0.86-1.22) | 0.825 | 0.939 | 0.05 | 0.04 | 0.453 | - | - | - | - | 5.92 | 0.052 | 20.38 |
|  |  | MR Egger | -0.49 | 0.45 | 0.61 (0.25-1.47) | 0.471 | 0.939 |  |  |  |  |  |  |  |  |  |  |
|  |  | Weighted median | -0.04 | 0.07 | 0.96 (0.84-1.10) | 0.555 | 0.897 |  |  |  |  |  |  |  |  |  |  |
| *Alcaligenaceae* | 12 | IVW | -0.08 | 0.05 | 0.92 (0.83-1.02) | 0.116 | 0.700 | -0.01 | 0.01 | 0.328 | 0.106 | - | - | - | 16.93 | 0.110 | 28.68 |
|  |  | MR Egger | 0.12 | 0.21 | 1.13 (0.75-1.69) | 0.570 | 0.939 |  |  |  |  |  |  |  |  |  |  |
|  |  | Weighted median | -0.09 | 0.06 | 0.91 (0.81-1.03) | 0.152 | 0.897 |  |  |  |  |  |  |  |  |  |  |
| *Bacteroidaceae* | 12 | IVW | -0.01 | 0.06 | 0.99 (0.88-1.11) | 0.805 | 0.700 | 0.01 | 0.01 | 0.955 | 0.014 | rs234027 | 0.94 (0.87-1.03) | 0.175 | 25.74 | 0.007 | 27.11 |
|  |  | MR Egger | -0.03 | 0.22 | 0.97 (0.63-1.51) | 0.905 | 0.939 |  |  |  |  |  | 1.10 (0.83-1.47) | 0.513 |  |  |  |
|  |  | Weighted median | -0.02 | 0.06 | 0.98 (0.87-1.10) | 0.722 | 0.897 |  |  |  |  |  | 0.97 (0.86-1.09) | 0.618 |  |  |  |
| *Bacteroidales_S24-7* | 6 | IVW | -0.04 | 0.03 | 0.96 (0.90-1.02) | 0.210 | 0.735 | 0.01 | 0.01 | 0.423 | 0.746 | - | - | - | 2.39 | 0.793 | 23.23 |
|  |  | MR Egger | -0.12 | 0.10 | 0.88 (0.73-1.07) | 0.276 | 0.939 |  |  |  |  |  |  |  |  |  |  |
|  |  | Weighted median | -0.06 | 0.04 | 0.95 (0.87-1.03) | 0.204 | 0.897 |  |  |  |  |  |  |  |  |  |  |
| *Bifidobacteriaceae* | 14 | IVW | 0.00 | 0.03 | 1.00 (0.94-1.07) | 0.963 | 0.963 | -0.01 | 0.01 | 0.326 | 0.661 | - | - | - | 10.32 | 0.668 | 29.80 |
|  |  | MR Egger | 0.12 | 0.12 | 1.13 (0.89-1.44) | 0.337 | 0.939 |  |  |  |  |  |  |  |  |  |  |
|  |  | Weighted median | 0.01 | 0.05 | 1.01 (0.92-1.11) | 0.829 | 0.897 |  |  |  |  |  |  |  |  |  |  |
| *Christensenellaceae* | 12 | IVW | -0.02 | 0.03 | 0.98 (0.93-1.03) | 0.430 | 0.741 | -0.01 | 0.00 | 0.065 | 0.361 | - | - | - | 12.58 | 0.321 | 29.09 |
|  |  | MR Egger | 0.07 | 0.05 | 1.07 (0.97-1.18) | 0.207 | 0.939 |  |  |  |  |  |  |  |  |  |  |
|  |  | Weighted median | 0.01 | 0.03 | 1.01 (0.94-1.08) | 0.760 | 0.897 |  |  |  |  |  |  |  |  |  |  |
| *Clostridiaceae_1* | 8 | IVW | -0.10 | 0.07 | 0.90 (0.79-1.03) | 0.128 | 0.700 | 0.01 | 0.02 | 0.618 | 0.082 | - | - | - | 14.56 | 0.042 | 22.18 |
|  |  | MR Egger | -0.25 | 0.29 | 0.78 (0.44-1.38) | 0.425 | 0.939 |  |  |  |  |  |  |  |  |  |  |
|  |  | Weighted median | -0.16 | 0.07 | 0.86 (0.75-0.98) | 0.022 | 0.607 |  |  |  |  |  |  |  |  |  |  |
| *Coriobacteriaceae* | 12 | IVW | 0.06 | 0.06 | 1.07 (0.94-1.20) | 0.299 | 0.741 | 0.02 | 0.02 | 0.275 | 0.059 | - | - | - | 21.22 | 0.031 | 24.85 |
|  |  | MR Egger | -0.22 | 0.25 | 0.81 (0.49-1.32) | 0.408 | 0.939 |  |  |  |  |  |  |  |  |  |  |
|  |  | Weighted median | 0.02 | 0.06 | 1.02 (0.90-1.15) | 0.743 | 0.897 |  |  |  |  |  |  |  |  |  |  |
| *Defluviitaleaceae* | 9 | IVW | -0.03 | 0.04 | 0.97 (0.90-1.04) | 0.344 | 0.741 | -0.01 | 0.01 | 0.697 | 0.602 | - | - | - | 7.15 | 0.520 | 24.25 |
|  |  | MR Egger | 0.03 | 0.16 | 1.03 (0.76-1.40) | 0.867 | 0.982 |  |  |  |  |  |  |  |  |  |  |
|  |  | Weighted median | -0.01 | 0.05 | 0.99 (0.90-1.08) | 0.808 | 0.897 |  |  |  |  |  |  |  |  |  |  |
| *Desulfovibrionaceae* | 10 | IVW | 0.06 | 0.07 | 1.06 (0.94-1.21) | 0.335 | 0.958 | 0.02 | 0.01 | 0.148 | 0.002 | rs6060237, rs7199026 | 1.00 (0.92-1.08) | 0.924 | 28.42 | 0.001 | 33.10 |
|  |  | MR Egger | -0.13 | 0.13 | 0.88 (0.68-1.14) | 0.369 | 0.939 |  |  |  |  |  | 0.92 (0.78-1.08) | 0.341 |  |  |  |
|  |  | Weighted median | -0.03 | 0.05 | 0.97 (0.88-1.07) | 0.534 | 0.897 |  |  |  |  |  | 0.96 (0.87-1.07) | 0.476 |  |  |  |
| *Enterobacteriaceae* | 8 | IVW | -0.03 | 0.05 | 0.97 (0.89-1.06) | 0.529 | 0.741 | -0.01 | 0.02 | 0.606 | 0.911 | - | - | - | 2.47 | 0.930 | 28.57 |
|  |  | MR Egger | 0.14 | 0.31 | 1.15 (0.62-2.12) | 0.672 | 0.982 |  |  |  |  |  |  |  |  |  |  |
|  |  | Weighted median | -0.03 | 0.06 | 0.97 (0.86-1.09) | 0.564 | 0.897 |  |  |  |  |  |  |  |  |  |  |
| *Erysipelotrichaceae* | 11 | IVW | 0.02 | 0.07 | 1.02 (0.90-1.16) | 0.724 | 0.881 | 0.00 | 0.02 | 0.980 | 0.057 | - | - | - | 18.55 | 0.046 | 23.30 |
|  |  | MR Egger | 0.03 | 0.35 | 1.03 (0.52-2.05) | 0.929 | 0.982 |  |  |  |  |  |  |  |  |  |  |
|  |  | Weighted median | 0.07 | 0.07 | 1.07 (0.93-1.24) | 0.314 | 0.897 |  |  |  |  |  |  |  |  |  |  |
| *Lachnospiraceae* | 13 | IVW | 0.01 | 0.04 | 1.01 (0.93-1.10) | 0.838 | 0.939 | 0.00 | 0.01 | 0.910 | 0.380 | - | - | - | 13.10 | 0.362 | 27.41 |
|  |  | MR Egger | 0.00 | 0.11 | 1.00 (0.81-1.23) | 0.982 | 0.982 |  |  |  |  |  |  |  |  |  |  |
|  |  | Weighted median | 0.02 | 0.06 | 1.02 (0.91-1.15) | 0.725 | 0.897 |  |  |  |  |  |  |  |  |  |  |
| *Lactobacillaceae* | 6 | IVW | 0.02 | 0.04 | 1.02 (0.95-1.10) | 0.525 | 0.741 | -0.01 | 0.02 | 0.449 | 0.475 | - | - | - | 4.81 | 0.439 | 25.23 |
|  |  | MR Egger | 0.17 | 0.18 | 1.18 (0.84-1.67) | 0.396 | 0.939 |  |  |  |  |  |  |  |  |  |  |
|  |  | Weighted median | 0.03 | 0.05 | 1.03 (0.94-1.14) | 0.476 | 0.897 |  |  |  |  |  |  |  |  |  |  |
| *Methanobacteriaceae* | 9 | IVW | -0.03 | 0.03 | 0.97 (0.91-1.04) | 0.385 | 0.741 | -0.03 | 0.02 | 0.204 | 0.178 | - | - | - | 12.71 | 0.122 | 21.32 |
|  |  | MR Egger | 0.18 | 0.16 | 1.20 (0.89-1.63) | 0.276 | 0.939 |  |  |  |  |  |  |  |  |  |  |
|  |  | Weighted median | -0.01 | 0.04 | 0.99 (0.92-1.06) | 0.685 | 0.897 |  |  |  |  |  |  |  |  |  |  |
| *Oxalobacteraceae* | 10 | IVW | 0.05 | 0.03 | 1.05 (0.99-1.11) | 0.102 | 0.700 | 0.02 | 0.01 | 0.205 | 0.298 | - | - | - | 11.04 | 0.273 | 21.29 |
|  |  | MR Egger | -0.11 | 0.12 | 0.89 (0.70-1.13) | 0.374 | 0.939 |  |  |  |  |  |  |  |  |  |  |
|  |  | Weighted median | 0.03 | 0.04 | 1.03 (0.95-1.11) | 0.472 | 0.897 |  |  |  |  |  |  |  |  |  |  |
| *Pasteurellaceae* | 11 | IVW | 0.00 | 0.03 | 1.00 (0.95-1.07) | 0.893 | 0.958 | 0.01 | 0.01 | 0.196 | 0.578 | - | - | - | 8.65 | 0.565 | 29.97 |
|  |  | MR Egger | -0.10 | 0.08 | 0.90 (0.77-1.06) | 0.246 | 0.939 |  |  |  |  |  |  |  |  |  |  |
|  |  | Weighted median | 0.01 | 0.04 | 1.01 (0.93-1.09) | 0.872 | 0.897 |  |  |  |  |  |  |  |  |  |  |
| *Peptococcaceae* | 8 | IVW | 0.02 | 0.04 | 1.02 (0.94-1.11) | 0.618 | 0.797 | 0.01 | 0.03 | 0.822 | 0.754 | - | - | - | 4.38 | 0.735 | 25.17 |
|  |  | MR Egger | -0.06 | 0.36 | 0.94 (0.46-1.90) | 0.867 | 0.982 |  |  |  |  |  |  |  |  |  |  |
|  |  | Weighted median | 0.04 | 0.05 | 1.04 (0.93-1.15) | 0.497 | 0.897 |  |  |  |  |  |  |  |  |  |  |
| *Peptostreptococcaceae* | 12 | IVW | 0.03 | 0.04 | 1.03 (0.94-1.12) | 0.518 | 0.741 | 0.00 | 0.02 | 0.818 | 0.421 | - | - | - | 11.05 | 0.439 | 21.11 |
|  |  | MR Egger | -0.03 | 0.23 | 0.97 (0.62-1.53) | 0.914 | 0.982 |  |  |  |  |  |  |  |  |  |  |
|  |  | Weighted median | 0.01 | 0.06 | 1.01 (0.90-1.14) | 0.865 | 0.897 |  |  |  |  |  |  |  |  |  |  |
| *Porphyromonadaceae* | 10 | IVW | -0.04 | 0.05 | 0.96 (0.87-1.06) | 0.378 | 0.741 | -0.01 | 0.02 | 0.772 | 0.925 | - | - | - | 3.70 | 0.930 | 31.62 |
|  |  | MR Egger | 0.05 | 0.32 | 1.05 (0.56-1.98) | 0.878 | 0.982 |  |  |  |  |  |  |  |  |  |  |
|  |  | Weighted median | -0.03 | 0.06 | 0.97 (0.86-1.10) | 0.677 | 0.897 |  |  |  |  |  |  |  |  |  |  |
| *Prevotellaceae* | 13 | IVW | -0.02 | 0.03 | 0.98 (0.92-1.04) | 0.475 | 0.741 | 0.00 | 0.01 | 0.442 | 0.955 | - | - | - | 4.74 | 0.966 | 32.36 |
|  |  | MR Egger | 0.02 | 0.06 | 1.03 (0.90-1.16) | 0.708 | 0.982 |  |  |  |  |  |  |  |  |  |  |
|  |  | Weighted median | 0.01 | 0.04 | 1.01 (0.93-1.10) | 0.773 | 0.897 |  |  |  |  |  |  |  |  |  |  |
| *Rhodospirillaceae* | 9 | IVW | 0.03 | 0.03 | 1.03 (0.96-1.09) | 0.403 | 0.741 | 0.00 | 0.02 | 0.861 | 0.806 | - | - | - | 4.70 | 0.789 | 27.83 |
|  |  | MR Egger | -0.01 | 0.23 | 0.99 (0.63-1.54) | 0.953 | 0.982 |  |  |  |  |  |  |  |  |  |  |
|  |  | Weighted median | 0.02 | 0.04 | 1.02 (0.94-1.11) | 0.590 | 0.897 |  |  |  |  |  |  |  |  |  |  |
| *Rikenellaceae* | 16 | IVW | -0.07 | 0.04 | 0.94 (0.87-1.01) | 0.073 | 0.700 | 0.00 | 0.01 | 0.888 | 0.179 | - | - | - | 20.67 | 0.148 | 25.35 |
|  |  | MR Egger | -0.09 | 0.14 | 0.92 (0.69-1.22) | 0.563 | 0.939 |  |  |  |  |  |  |  |  |  |  |
|  |  | Weighted median | -0.08 | 0.04 | 0.92 (0.85-1.01) | 0.065 | 0.607 |  |  |  |  |  |  |  |  |  |  |
| *Ruminococcaceae* | 4 | IVW | -0.04 | 0.06 | 0.96 (0.85-1.08) | 0.489 | 0.741 | -0.02 | 0.01 | 0.271 | 0.348 | - | - | - | 4.13 | 0.248 | 37.78 |
|  |  | MR Egger | 0.10 | 0.10 | 1.10 (0.90-1.35) | 0.455 | 0.939 |  |  |  |  |  |  |  |  |  |  |
|  |  | Weighted median | -0.03 | 0.06 | 0.97 (0.85-1.10) | 0.600 | 0.897 |  |  |  |  |  |  |  |  |  |  |
| *Streptococcaceae* | 8 | IVW | 0.02 | 0.04 | 1.02 (0.94-1.11) | 0.626 | 0.797 | -0.01 | 0.01 | 0.431 | 0.428 | - | - | - | 6.37 | 0.498 | 20.26 |
|  |  | MR Egger | 0.17 | 0.18 | 1.84 (0.83-1.69) | 0.385 | 0.939 |  |  |  |  |  |  |  |  |  |  |
|  |  | Weighted median | 0.01 | 0.06 | 1.01 (0.89-1.14) | 0.897 | 0.897 |  |  |  |  |  |  |  |  |  |  |
| *Veillonellaceae* | 16 | IVW | -0.06 | 0.04 | 0.94 (0.87-1.02) | 0.157 | 0.700 | 0.00 | 0.01 | 0.772 | 0.065 | - | - | - | 28.89 | 0.017 | 28.87 |
|  |  | MR Egger | -0.04 | 0.08 | 0.96 (0.82-1.14) | 0.678 | 0.982 |  |  |  |  |  |  |  |  |  |  |
|  |  | Weighted median | -0.02 | 0.04 | 0.98 (0.90-1.07) | 0.695 | 0.897 |  |  |  |  |  |  |  |  |  |  |
| *Verrucomicrobiaceae* | 8 | IVW | 0.04 | 0.06 | 1.04 (0.93-1.18) | 0.481 | 0.741 | -0.01 | 0.06 | 0.922 | 0.067 | - | - | - | 13.78 | 0.055 | 24.44 |
|  |  | MR Egger | 0.13 | 0.85 | 1.14 (0.21-6.06) | 0.884 | 0.982 |  |  |  |  |  |  |  |  |  |  |
|  |  | Weighted median | 0.07 | 0.07 | 1.07 (0.94-1.22) | 0.297 | 0.897 |  |  |  |  |  |  |  |  |  |  |

a. Not enough SNPs for MR-PRESSO analysis

Abbreviations: MR, Mendelian randomization; SNP, single nucleotide polymorphism; IVW, inverse variance weighted; IVs, instrumental variables; FDR, false discovery rate; T2DM, type 2 diabetes mellitus; OR, odds ratio; MR-PRESSO, Mendelian randomization pleiotropy residual sum and outlier.
