# Supplementary material for: Genetically Predicted Causality of 28 Gut Microbiome Families and Type 2 Diabetes Mellitus Risk
Source: Front Endocrinol (Lausanne). 2022 Feb 3;13:780133. doi: 10.3389/fendo.2022.780133 (PMC8851667; doi:10.3389/fendo.2022.780133)
Supplement: Supplementary file 7 [file Table_5.docx]

| **Supplementary Table 5. SNPs used as IVs from T2DM and gut microbiome GWAS (European)** | | | | | | | | | |
| --- | --- | --- | --- | --- | --- | --- | --- | --- | --- |
| **Bacterial traits** | **SNP** | **Effect allele** | **Other allele** | **T2DM** | | | **Gut microbiome** | | |
|  |  |  |  | **Beta** | **SE** | ***P* value** | **Beta** | **SE** | ***P* value** |
| *Acidaminococcaceae* | rs10077431 | A | C | -0.049 | 0.009 | 4.75E-08 | 0.001 | 0.016 | 0.915 |
|  | rs10087241 | A | G | -0.048 | 0.008 | 2.80E-09 | 0.003 | 0.013 | 0.811 |
|  | rs10100265 | A | C | 0.049 | 0.008 | 6.29E-10 | 0.010 | 0.013 | 0.438 |
|  | rs10114341 | C | T | -0.041 | 0.007 | 1.15E-08 | 0.022 | 0.013 | 0.076 |
|  | rs10401969 | C | T | 0.092 | 0.013 | 4.13E-12 | 0.044 | 0.024 | 0.073 |
|  | rs1050226 | G | A | -0.049 | 0.007 | 3.34E-11 | 0.008 | 0.013 | 0.517 |
|  | rs1061813 | A | G | -0.043 | 0.007 | 3.37E-09 | 0.021 | 0.013 | 0.096 |
|  | rs1063355 | T | G | -0.071 | 0.008 | 3.72E-19 | 0.008 | 0.013 | 0.557 |
|  | rs10740322 | A | G | 0.048 | 0.009 | 2.11E-08 | 0.013 | 0.014 | 0.367 |
|  | rs10811661 | C | T | -0.157 | 0.010 | 4.13E-58 | 0.010 | 0.017 | 0.571 |
|  | rs10842994 | C | T | 0.076 | 0.009 | 1.02E-16 | 0.022 | 0.016 | 0.129 |
|  | rs10974438 | A | C | -0.059 | 0.008 | 3.01E-15 | 0.008 | 0.013 | 0.535 |
|  | rs11098676 | C | T | 0.054 | 0.010 | 2.03E-08 | 0.018 | 0.015 | 0.230 |
|  | rs11107116 | G | T | -0.047 | 0.009 | 3.75E-08 | 0.013 | 0.015 | 0.379 |
|  | rs1111875 | T | C | -0.095 | 0.007 | 3.61E-39 | 0.005 | 0.013 | 0.687 |
|  | rs11257655 | C | T | -0.074 | 0.009 | 1.97E-17 | 0.028 | 0.015 | 0.061 |
|  | rs1127655 | C | T | 0.044 | 0.008 | 2.47E-08 | -0.011 | 0.013 | 0.408 |
|  | rs11708067 | A | G | 0.097 | 0.009 | 5.93E-29 | 0.003 | 0.015 | 0.856 |
|  | rs11925227 | A | G | -0.053 | 0.010 | 2.25E-08 | -0.028 | 0.017 | 0.098 |
|  | rs11926707 | C | T | 0.046 | 0.008 | 1.69E-08 | 0.000 | 0.013 | 0.980 |
|  | rs12088739 | A | G | 0.088 | 0.013 | 9.79E-12 | 0.020 | 0.022 | 0.352 |
|  | rs12299509 | A | G | -0.047 | 0.007 | 2.09E-10 | 0.027 | 0.013 | 0.035 |
|  | rs12617659 | C | T | 0.069 | 0.010 | 2.83E-11 | -0.028 | 0.018 | 0.135 |
|  | rs12910825 | A | G | -0.052 | 0.007 | 2.16E-12 | 0.001 | 0.013 | 0.946 |
|  | rs12945601 | C | T | -0.048 | 0.008 | 1.72E-09 | 0.005 | 0.013 | 0.743 |
|  | rs12970134 | A | G | 0.056 | 0.008 | 5.31E-12 | 0.014 | 0.014 | 0.386 |
|  | rs13239186 | C | T | -0.054 | 0.009 | 2.70E-10 | -0.008 | 0.014 | 0.574 |
|  | rs13330951 | A | G | 0.046 | 0.008 | 1.54E-08 | 0.015 | 0.013 | 0.189 |
|  | rs13389219 | C | T | 0.072 | 0.007 | 2.11E-22 | 0.005 | 0.013 | 0.636 |
|  | rs1359790 | G | A | 0.080 | 0.008 | 2.80E-23 | 0.009 | 0.014 | 0.525 |
|  | rs1496653 | G | A | -0.077 | 0.009 | 2.57E-18 | 0.013 | 0.016 | 0.385 |
|  | rs1552224 | C | A | -0.103 | 0.010 | 8.64E-25 | -0.021 | 0.018 | 0.224 |
|  | rs16988333 | A | G | 0.075 | 0.013 | 9.17E-09 | -0.033 | 0.025 | 0.195 |
|  | rs17086692 | G | T | 0.047 | 0.008 | 2.48E-08 | 0.018 | 0.014 | 0.186 |
|  | rs17168486 | C | T | -0.074 | 0.009 | 2.18E-15 | -0.005 | 0.016 | 0.904 |
|  | rs17405722 | A | G | 0.087 | 0.015 | 2.28E-09 | -0.007 | 0.026 | 0.796 |
|  | rs17631783 | C | T | 0.049 | 0.009 | 3.95E-08 | -0.013 | 0.015 | 0.361 |
|  | rs17791513 | A | G | 0.103 | 0.015 | 4.61E-12 | 0.006 | 0.026 | 0.935 |
|  | rs1801214 | C | T | 0.090 | 0.007 | 5.52E-34 | 0.011 | 0.013 | 0.379 |
|  | rs1899951 | T | C | -0.112 | 0.011 | 1.64E-24 | 0.042 | 0.019 | 0.020 |
|  | rs2237892 | C | T | 0.096 | 0.016 | 8.75E-10 | 0.030 | 0.025 | 0.293 |
|  | rs2246618 | C | T | -0.051 | 0.008 | 1.20E-09 | 0.006 | 0.014 | 0.702 |
|  | rs2261181 | C | T | -0.099 | 0.012 | 9.18E-17 | 0.028 | 0.021 | 0.208 |
|  | rs2294120 | G | A | -0.044 | 0.008 | 1.62E-08 | -0.012 | 0.013 | 0.326 |
|  | rs2296173 | A | G | -0.065 | 0.009 | 7.66E-14 | 0.000 | 0.016 | 0.895 |
|  | rs2299383 | C | T | -0.041 | 0.007 | 1.49E-08 | -0.005 | 0.013 | 0.701 |
|  | rs243019 | T | C | -0.057 | 0.007 | 2.29E-15 | 0.007 | 0.013 | 0.592 |
|  | rs2493394 | G | A | 0.073 | 0.011 | 1.15E-10 | -0.002 | 0.021 | 0.935 |
|  | rs2796441 | G | A | 0.072 | 0.007 | 1.96E-22 | -0.010 | 0.013 | 0.434 |
|  | rs2820426 | G | A | 0.052 | 0.007 | 1.30E-12 | -0.004 | 0.013 | 0.758 |
|  | rs2867125 | T | C | -0.060 | 0.010 | 4.33E-10 | -0.002 | 0.017 | 0.863 |
|  | rs2908282 | G | A | -0.055 | 0.009 | 4.25E-09 | 0.005 | 0.017 | 0.715 |
|  | rs2925979 | T | C | 0.053 | 0.008 | 9.06E-12 | -0.006 | 0.014 | 0.700 |
|  | rs2943656 | A | G | -0.090 | 0.007 | 6.70E-34 | 0.003 | 0.013 | 0.757 |
|  | rs3217992 | T | C | 0.053 | 0.007 | 7.23E-13 | -0.013 | 0.013 | 0.321 |
|  | rs340874 | T | C | -0.063 | 0.007 | 8.41E-18 | -0.003 | 0.013 | 0.780 |
|  | rs348330 | G | A | 0.049 | 0.008 | 1.86E-09 | 0.005 | 0.013 | 0.722 |
|  | rs3756784 | G | T | 0.051 | 0.009 | 2.59E-08 | 0.015 | 0.016 | 0.349 |
|  | rs3802177 | G | A | 0.122 | 0.008 | 2.32E-52 | -0.019 | 0.014 | 0.153 |
|  | rs459193 | G | A | 0.071 | 0.008 | 8.81E-18 | -0.008 | 0.015 | 0.555 |
|  | rs4622883 | A | G | 0.044 | 0.008 | 3.02E-08 | 0.015 | 0.013 | 0.230 |
|  | rs4686471 | C | T | 0.053 | 0.008 | 4.28E-11 | 0.005 | 0.013 | 0.759 |
|  | rs4812829 | A | G | 0.053 | 0.010 | 2.44E-08 | -0.028 | 0.016 | 0.074 |
|  | rs4823182 | A | G | -0.048 | 0.008 | 3.36E-10 | -0.007 | 0.013 | 0.636 |
|  | rs4865796 | A | G | 0.053 | 0.008 | 1.33E-11 | -0.010 | 0.014 | 0.474 |
|  | rs516946 | T | C | -0.082 | 0.009 | 3.16E-22 | -0.009 | 0.015 | 0.677 |
|  | rs5215 | C | T | 0.068 | 0.007 | 2.09E-20 | 0.003 | 0.013 | 0.795 |
|  | rs576674 | A | G | -0.065 | 0.010 | 1.79E-11 | 0.010 | 0.017 | 0.518 |
|  | rs6059662 | A | G | -0.045 | 0.008 | 1.51E-08 | -0.008 | 0.014 | 0.583 |
|  | rs61953351 | G | T | 0.070 | 0.009 | 1.98E-14 | 0.007 | 0.015 | 0.640 |
|  | rs622217 | C | T | -0.049 | 0.008 | 3.13E-10 | 0.014 | 0.013 | 0.254 |
|  | rs6515236 | A | C | 0.050 | 0.009 | 3.34E-08 | -0.035 | 0.014 | 0.013 |
|  | rs67232546 | C | T | -0.060 | 0.010 | 4.66E-10 | -0.009 | 0.016 | 0.571 |
|  | rs6767484 | A | G | -0.121 | 0.008 | 2.70E-56 | -0.003 | 0.014 | 0.856 |
|  | rs6785040 | C | T | -0.063 | 0.011 | 1.26E-08 | -0.012 | 0.017 | 0.468 |
|  | rs6795735 | C | T | 0.056 | 0.007 | 1.63E-14 | 0.004 | 0.013 | 0.771 |
|  | rs6878122 | A | G | -0.056 | 0.008 | 1.19E-12 | -0.001 | 0.014 | 0.904 |
|  | rs6960043 | C | T | 0.064 | 0.007 | 3.61E-19 | -0.010 | 0.013 | 0.439 |
|  | rs7144011 | G | T | -0.048 | 0.009 | 1.64E-08 | 0.009 | 0.016 | 0.574 |
|  | rs7177055 | A | G | 0.065 | 0.008 | 2.75E-16 | -0.011 | 0.014 | 0.450 |
|  | rs7240767 | C | T | 0.045 | 0.008 | 2.16E-08 | -0.007 | 0.013 | 0.626 |
|  | rs72892910 | G | T | -0.065 | 0.010 | 6.43E-11 | 0.033 | 0.016 | 0.047 |
|  | rs735949 | C | T | -0.071 | 0.011 | 1.95E-11 | -0.001 | 0.019 | 0.983 |
|  | rs753270 | C | T | 0.053 | 0.008 | 2.70E-11 | -0.004 | 0.013 | 0.795 |
|  | rs7561798 | A | G | -0.040 | 0.007 | 2.79E-08 | -0.006 | 0.013 | 0.643 |
|  | rs7572970 | A | G | -0.059 | 0.009 | 1.39E-11 | -0.006 | 0.014 | 0.655 |
|  | rs7607777 | G | T | 0.137 | 0.013 | 9.40E-28 | 0.002 | 0.021 | 0.842 |
|  | rs7674212 | G | T | 0.047 | 0.008 | 6.18E-10 | 0.004 | 0.013 | 0.724 |
|  | rs7685296 | C | T | 0.051 | 0.008 | 2.32E-10 | -0.003 | 0.014 | 0.840 |
|  | rs7756992 | A | G | -0.130 | 0.008 | 6.00E-62 | 0.014 | 0.014 | 0.304 |
|  | rs7786095 | A | G | 0.074 | 0.013 | 9.64E-09 | -0.008 | 0.022 | 0.685 |
|  | rs780094 | T | C | -0.069 | 0.007 | 5.16E-21 | -0.017 | 0.013 | 0.186 |
|  | rs7845219 | C | T | -0.042 | 0.007 | 4.54E-09 | 0.011 | 0.013 | 0.406 |
|  | rs7903146 | C | T | -0.306 | 0.008 | 1.00E-200 | 0.004 | 0.014 | 0.742 |
|  | rs7929543 | A | C | -0.083 | 0.014 | 2.20E-09 | 0.007 | 0.022 | 0.729 |
|  | rs7955901 | C | T | 0.044 | 0.007 | 7.16E-10 | -0.003 | 0.013 | 0.814 |
|  | rs8068804 | A | G | 0.059 | 0.008 | 4.41E-14 | -0.006 | 0.014 | 0.641 |
|  | rs8108269 | G | T | 0.064 | 0.008 | 3.11E-16 | -0.008 | 0.014 | 0.539 |
|  | rs825476 | C | T | -0.052 | 0.007 | 6.80E-13 | 0.003 | 0.013 | 0.781 |
|  | rs840967 | A | C | -0.050 | 0.008 | 5.44E-10 | -0.005 | 0.013 | 0.749 |
|  | rs849135 | A | G | -0.100 | 0.007 | 1.04E-43 | -0.012 | 0.013 | 0.349 |
|  | rs853974 | C | T | -0.060 | 0.009 | 7.86E-12 | 0.014 | 0.014 | 0.333 |
|  | rs9369425 | A | G | -0.055 | 0.009 | 1.13E-10 | -0.002 | 0.014 | 0.849 |
|  | rs9894220 | A | G | 0.059 | 0.008 | 1.52E-13 | 0.021 | 0.013 | 0.103 |
|  | rs9928094 | A | G | -0.105 | 0.007 | 3.59E-47 | 0.009 | 0.013 | 0.497 |
|  | rs993380 | A | G | 0.051 | 0.008 | 4.59E-10 | -0.006 | 0.013 | 0.692 |
|  | rs9940149 | A | G | -0.058 | 0.010 | 9.29E-10 | 0.000 | 0.016 | 0.924 |
| *Actinomycetaceae* | rs10077431 | A | C | -0.049 | 0.009 | 4.75E-08 | 0.011 | 0.020 | 0.568 |
|  | rs10087241 | A | G | -0.048 | 0.008 | 2.80E-09 | -0.027 | 0.016 | 0.125 |
|  | rs10100265 | A | C | 0.049 | 0.008 | 6.29E-10 | -0.021 | 0.016 | 0.197 |
|  | rs10114341 | C | T | -0.041 | 0.007 | 1.15E-08 | -0.021 | 0.016 | 0.185 |
|  | rs10401969 | C | T | 0.092 | 0.013 | 4.13E-12 | 0.038 | 0.030 | 0.153 |
|  | rs1050226 | G | A | -0.049 | 0.007 | 3.34E-11 | -0.007 | 0.016 | 0.686 |
|  | rs1061813 | A | G | -0.043 | 0.007 | 3.37E-09 | -0.012 | 0.016 | 0.480 |
|  | rs1063355 | T | G | -0.071 | 0.008 | 3.72E-19 | 0.042 | 0.016 | 0.010 |
|  | rs10740322 | A | G | 0.048 | 0.009 | 2.11E-08 | 0.024 | 0.017 | 0.167 |
|  | rs10811661 | C | T | -0.157 | 0.010 | 4.13E-58 | 0.016 | 0.021 | 0.423 |
|  | rs10842994 | C | T | 0.076 | 0.009 | 1.02E-16 | -0.021 | 0.020 | 0.347 |
|  | rs10974438 | A | C | -0.059 | 0.008 | 3.01E-15 | -0.028 | 0.017 | 0.097 |
|  | rs11098676 | C | T | 0.054 | 0.010 | 2.03E-08 | 0.016 | 0.020 | 0.376 |
|  | rs11107116 | G | T | -0.047 | 0.009 | 3.75E-08 | 0.011 | 0.019 | 0.567 |
|  | rs1111875 | T | C | -0.095 | 0.007 | 3.61E-39 | 0.007 | 0.016 | 0.653 |
|  | rs11257655 | C | T | -0.074 | 0.009 | 1.97E-17 | -0.001 | 0.020 | 0.978 |
|  | rs1127655 | C | T | 0.044 | 0.008 | 2.47E-08 | 0.028 | 0.016 | 0.089 |
|  | rs11708067 | A | G | 0.097 | 0.009 | 5.93E-29 | -0.026 | 0.019 | 0.181 |
|  | rs11925227 | A | G | -0.053 | 0.010 | 2.25E-08 | 0.027 | 0.021 | 0.201 |
|  | rs11926707 | C | T | 0.046 | 0.008 | 1.69E-08 | 0.000 | 0.017 | 0.980 |
|  | rs12088739 | A | G | 0.088 | 0.013 | 9.79E-12 | -0.031 | 0.028 | 0.258 |
|  | rs12299509 | A | G | -0.047 | 0.007 | 2.09E-10 | 0.002 | 0.016 | 0.874 |
|  | rs12617659 | C | T | 0.069 | 0.010 | 2.83E-11 | 0.034 | 0.023 | 0.156 |
|  | rs12910825 | A | G | -0.052 | 0.007 | 2.16E-12 | -0.001 | 0.017 | 0.949 |
|  | rs12945601 | C | T | -0.048 | 0.008 | 1.72E-09 | 0.000 | 0.017 | 0.981 |
|  | rs12970134 | A | G | 0.056 | 0.008 | 5.31E-12 | 0.001 | 0.018 | 0.989 |
|  | rs13239186 | C | T | -0.054 | 0.009 | 2.70E-10 | -0.028 | 0.017 | 0.114 |
|  | rs13330951 | A | G | 0.046 | 0.008 | 1.54E-08 | -0.005 | 0.016 | 0.784 |
|  | rs13389219 | C | T | 0.072 | 0.007 | 2.11E-22 | 0.032 | 0.017 | 0.060 |
|  | rs1359790 | G | A | 0.080 | 0.008 | 2.80E-23 | -0.028 | 0.018 | 0.109 |
|  | rs1496653 | G | A | -0.077 | 0.009 | 2.57E-18 | -0.021 | 0.020 | 0.297 |
|  | rs1552224 | C | A | -0.103 | 0.010 | 8.64E-25 | -0.010 | 0.022 | 0.695 |
|  | rs16988333 | A | G | 0.075 | 0.013 | 9.17E-09 | 0.027 | 0.030 | 0.330 |
|  | rs17086692 | G | T | 0.047 | 0.008 | 2.48E-08 | 0.027 | 0.017 | 0.102 |
|  | rs17168486 | C | T | -0.074 | 0.009 | 2.18E-15 | -0.020 | 0.021 | 0.377 |
|  | rs17405722 | A | G | 0.087 | 0.015 | 2.28E-09 | 0.011 | 0.030 | 0.758 |
|  | rs17631783 | C | T | 0.049 | 0.009 | 3.95E-08 | 0.025 | 0.018 | 0.169 |
|  | rs17791513 | A | G | 0.103 | 0.015 | 4.61E-12 | 0.004 | 0.033 | 0.919 |
|  | rs1801214 | C | T | 0.090 | 0.007 | 5.52E-34 | 0.011 | 0.016 | 0.499 |
|  | rs1899951 | T | C | -0.112 | 0.011 | 1.64E-24 | -0.023 | 0.024 | 0.338 |
|  | rs2237892 | C | T | 0.096 | 0.016 | 8.75E-10 | 0.059 | 0.037 | 0.108 |
|  | rs2246618 | C | T | -0.051 | 0.008 | 1.20E-09 | 0.021 | 0.017 | 0.221 |
|  | rs2261181 | C | T | -0.099 | 0.012 | 9.18E-17 | 0.015 | 0.027 | 0.608 |
|  | rs2294120 | G | A | -0.044 | 0.008 | 1.62E-08 | -0.017 | 0.016 | 0.273 |
|  | rs2296173 | A | G | -0.065 | 0.009 | 7.66E-14 | -0.014 | 0.020 | 0.481 |
|  | rs2299383 | C | T | -0.041 | 0.007 | 1.49E-08 | 0.010 | 0.016 | 0.559 |
|  | rs243019 | T | C | -0.057 | 0.007 | 2.29E-15 | -0.029 | 0.016 | 0.078 |
|  | rs2493394 | G | A | 0.073 | 0.011 | 1.15E-10 | 0.009 | 0.026 | 0.636 |
|  | rs2796441 | G | A | 0.072 | 0.007 | 1.96E-22 | -0.015 | 0.016 | 0.364 |
|  | rs2820426 | G | A | 0.052 | 0.007 | 1.30E-12 | 0.008 | 0.017 | 0.634 |
|  | rs2867125 | T | C | -0.060 | 0.010 | 4.33E-10 | 0.004 | 0.021 | 0.834 |
|  | rs2908282 | G | A | -0.055 | 0.009 | 4.25E-09 | -0.024 | 0.022 | 0.260 |
|  | rs2925979 | T | C | 0.053 | 0.008 | 9.06E-12 | -0.013 | 0.017 | 0.428 |
|  | rs2943656 | A | G | -0.090 | 0.007 | 6.70E-34 | -0.013 | 0.017 | 0.507 |
|  | rs3217992 | T | C | 0.053 | 0.007 | 7.23E-13 | 0.034 | 0.017 | 0.046 |
|  | rs340874 | T | C | -0.063 | 0.007 | 8.41E-18 | -0.022 | 0.016 | 0.166 |
|  | rs348330 | G | A | 0.049 | 0.008 | 1.86E-09 | 0.004 | 0.017 | 0.831 |
|  | rs3756784 | G | T | 0.051 | 0.009 | 2.59E-08 | 0.021 | 0.020 | 0.305 |
|  | rs3802177 | G | A | 0.122 | 0.008 | 2.32E-52 | 0.015 | 0.017 | 0.399 |
|  | rs459193 | G | A | 0.071 | 0.008 | 8.81E-18 | -0.005 | 0.019 | 0.743 |
|  | rs4622883 | A | G | 0.044 | 0.008 | 3.02E-08 | 0.019 | 0.016 | 0.244 |
|  | rs4686471 | C | T | 0.053 | 0.008 | 4.28E-11 | -0.020 | 0.017 | 0.278 |
|  | rs4812829 | A | G | 0.053 | 0.010 | 2.44E-08 | 0.007 | 0.021 | 0.717 |
|  | rs4823182 | A | G | -0.048 | 0.008 | 3.36E-10 | 0.019 | 0.017 | 0.254 |
|  | rs4865796 | A | G | 0.053 | 0.008 | 1.33E-11 | -0.005 | 0.017 | 0.816 |
|  | rs516946 | T | C | -0.082 | 0.009 | 3.16E-22 | -0.012 | 0.019 | 0.528 |
|  | rs5215 | C | T | 0.068 | 0.007 | 2.09E-20 | -0.011 | 0.017 | 0.495 |
|  | rs576674 | A | G | -0.065 | 0.010 | 1.79E-11 | 0.021 | 0.022 | 0.349 |
|  | rs6059662 | A | G | -0.045 | 0.008 | 1.51E-08 | -0.030 | 0.017 | 0.101 |
|  | rs61953351 | G | T | 0.070 | 0.009 | 1.98E-14 | 0.004 | 0.019 | 0.795 |
|  | rs622217 | C | T | -0.049 | 0.008 | 3.13E-10 | -0.025 | 0.016 | 0.124 |
|  | rs6515236 | A | C | 0.050 | 0.009 | 3.34E-08 | -0.007 | 0.019 | 0.714 |
|  | rs67232546 | C | T | -0.060 | 0.010 | 4.66E-10 | 0.037 | 0.020 | 0.054 |
|  | rs6767484 | A | G | -0.121 | 0.008 | 2.70E-56 | -0.018 | 0.017 | 0.282 |
|  | rs6785040 | C | T | -0.063 | 0.011 | 1.26E-08 | 0.012 | 0.022 | 0.563 |
|  | rs6795735 | C | T | 0.056 | 0.007 | 1.63E-14 | -0.011 | 0.016 | 0.515 |
|  | rs6878122 | A | G | -0.056 | 0.008 | 1.19E-12 | 0.010 | 0.018 | 0.594 |
|  | rs6960043 | C | T | 0.064 | 0.007 | 3.61E-19 | 0.004 | 0.016 | 0.829 |
|  | rs7144011 | G | T | -0.048 | 0.009 | 1.64E-08 | 0.031 | 0.020 | 0.124 |
|  | rs7177055 | A | G | 0.065 | 0.008 | 2.75E-16 | 0.014 | 0.018 | 0.439 |
|  | rs7240767 | C | T | 0.045 | 0.008 | 2.16E-08 | -0.004 | 0.017 | 0.789 |
|  | rs72892910 | G | T | -0.065 | 0.010 | 6.43E-11 | -0.005 | 0.021 | 0.871 |
|  | rs735949 | C | T | -0.071 | 0.011 | 1.95E-11 | -0.048 | 0.024 | 0.041 |
|  | rs753270 | C | T | 0.053 | 0.008 | 2.70E-11 | -0.025 | 0.016 | 0.134 |
|  | rs7561798 | A | G | -0.040 | 0.007 | 2.79E-08 | 0.019 | 0.016 | 0.238 |
|  | rs7572970 | A | G | -0.059 | 0.009 | 1.39E-11 | 0.018 | 0.018 | 0.340 |
|  | rs7607777 | G | T | 0.137 | 0.013 | 9.40E-28 | -0.003 | 0.026 | 0.800 |
|  | rs7674212 | G | T | 0.047 | 0.008 | 6.18E-10 | -0.018 | 0.016 | 0.277 |
|  | rs7685296 | C | T | 0.051 | 0.008 | 2.32E-10 | -0.013 | 0.018 | 0.453 |
|  | rs7756992 | A | G | -0.130 | 0.008 | 6.00E-62 | 0.027 | 0.018 | 0.113 |
|  | rs7786095 | A | G | 0.074 | 0.013 | 9.64E-09 | 0.005 | 0.027 | 0.855 |
|  | rs780094 | T | C | -0.069 | 0.007 | 5.16E-21 | -0.025 | 0.017 | 0.140 |
|  | rs7845219 | C | T | -0.042 | 0.007 | 4.54E-09 | 0.007 | 0.016 | 0.696 |
|  | rs7903146 | C | T | -0.306 | 0.008 | 1.00E-200 | -0.007 | 0.018 | 0.685 |
|  | rs7929543 | A | C | -0.083 | 0.014 | 2.20E-09 | -0.043 | 0.028 | 0.116 |
|  | rs7955901 | C | T | 0.044 | 0.007 | 7.16E-10 | 0.001 | 0.016 | 0.945 |
|  | rs8068804 | A | G | 0.059 | 0.008 | 4.41E-14 | -0.014 | 0.017 | 0.445 |
|  | rs8108269 | G | T | 0.064 | 0.008 | 3.11E-16 | 0.032 | 0.018 | 0.071 |
|  | rs825476 | C | T | -0.052 | 0.007 | 6.80E-13 | 0.019 | 0.016 | 0.240 |
|  | rs840967 | A | C | -0.050 | 0.008 | 5.44E-10 | -0.011 | 0.016 | 0.542 |
|  | rs849135 | A | G | -0.100 | 0.007 | 1.04E-43 | 0.002 | 0.016 | 0.892 |
|  | rs853974 | C | T | -0.060 | 0.009 | 7.86E-12 | 0.011 | 0.018 | 0.513 |
|  | rs9369425 | A | G | -0.055 | 0.009 | 1.13E-10 | 0.008 | 0.018 | 0.689 |
|  | rs9894220 | A | G | 0.059 | 0.008 | 1.52E-13 | 0.011 | 0.016 | 0.506 |
|  | rs9928094 | A | G | -0.105 | 0.007 | 3.59E-47 | -0.011 | 0.016 | 0.495 |
|  | rs993380 | A | G | 0.051 | 0.008 | 4.59E-10 | -0.003 | 0.017 | 0.860 |
|  | rs9940149 | A | G | -0.058 | 0.010 | 9.29E-10 | 0.030 | 0.021 | 0.175 |
| *Alcaligenaceae* | rs10077431 | A | C | -0.049 | 0.009 | 4.75E-08 | -0.004 | 0.014 | 0.818 |
|  | rs10087241 | A | G | -0.048 | 0.008 | 2.80E-09 | 0.006 | 0.011 | 0.680 |
|  | rs10100265 | A | C | 0.049 | 0.008 | 6.29E-10 | -0.011 | 0.011 | 0.299 |
|  | rs10114341 | C | T | -0.041 | 0.007 | 1.15E-08 | 0.015 | 0.011 | 0.161 |
|  | rs10401969 | C | T | 0.092 | 0.013 | 4.13E-12 | 0.025 | 0.021 | 0.258 |
|  | rs1050226 | G | A | -0.049 | 0.007 | 3.34E-11 | -0.014 | 0.011 | 0.195 |
|  | rs1061813 | A | G | -0.043 | 0.007 | 3.37E-09 | 0.003 | 0.011 | 0.773 |
|  | rs1063355 | T | G | -0.071 | 0.008 | 3.72E-19 | -0.010 | 0.011 | 0.396 |
|  | rs10740322 | A | G | 0.048 | 0.009 | 2.11E-08 | -0.009 | 0.012 | 0.410 |
|  | rs10811661 | C | T | -0.157 | 0.010 | 4.13E-58 | -0.007 | 0.015 | 0.631 |
|  | rs10842994 | C | T | 0.076 | 0.009 | 1.02E-16 | -0.002 | 0.014 | 0.874 |
|  | rs10974438 | A | C | -0.059 | 0.008 | 3.01E-15 | -0.010 | 0.011 | 0.372 |
|  | rs11098676 | C | T | 0.054 | 0.010 | 2.03E-08 | 0.006 | 0.013 | 0.618 |
|  | rs11107116 | G | T | -0.047 | 0.009 | 3.75E-08 | -0.001 | 0.013 | 0.958 |
|  | rs1111875 | T | C | -0.095 | 0.007 | 3.61E-39 | 0.013 | 0.011 | 0.251 |
|  | rs11257655 | C | T | -0.074 | 0.009 | 1.97E-17 | 0.005 | 0.013 | 0.658 |
|  | rs1127655 | C | T | 0.044 | 0.008 | 2.47E-08 | -0.003 | 0.011 | 0.799 |
|  | rs11708067 | A | G | 0.097 | 0.009 | 5.93E-29 | -0.012 | 0.013 | 0.399 |
|  | rs11925227 | A | G | -0.053 | 0.010 | 2.25E-08 | -0.005 | 0.014 | 0.731 |
|  | rs11926707 | C | T | 0.046 | 0.008 | 1.69E-08 | 0.005 | 0.011 | 0.670 |
|  | rs12088739 | A | G | 0.088 | 0.013 | 9.79E-12 | -0.005 | 0.019 | 0.737 |
|  | rs12299509 | A | G | -0.047 | 0.007 | 2.09E-10 | -0.002 | 0.011 | 0.882 |
|  | rs12617659 | C | T | 0.069 | 0.010 | 2.83E-11 | -0.012 | 0.016 | 0.467 |
|  | rs12910825 | A | G | -0.052 | 0.007 | 2.16E-12 | 0.003 | 0.011 | 0.790 |
|  | rs12945601 | C | T | -0.048 | 0.008 | 1.72E-09 | -0.020 | 0.011 | 0.066 |
|  | rs12970134 | A | G | 0.056 | 0.008 | 5.31E-12 | -0.002 | 0.012 | 0.848 |
|  | rs13239186 | C | T | -0.054 | 0.009 | 2.70E-10 | 0.008 | 0.012 | 0.564 |
|  | rs13330951 | A | G | 0.046 | 0.008 | 1.54E-08 | 0.010 | 0.011 | 0.397 |
|  | rs13389219 | C | T | 0.072 | 0.007 | 2.11E-22 | -0.006 | 0.011 | 0.535 |
|  | rs1359790 | G | A | 0.080 | 0.008 | 2.80E-23 | -0.009 | 0.012 | 0.532 |
|  | rs1496653 | G | A | -0.077 | 0.009 | 2.57E-18 | 0.017 | 0.014 | 0.220 |
|  | rs1552224 | C | A | -0.103 | 0.010 | 8.64E-25 | 0.034 | 0.015 | 0.025 |
|  | rs16988333 | A | G | 0.075 | 0.013 | 9.17E-09 | -0.011 | 0.022 | 0.654 |
|  | rs17086692 | G | T | 0.047 | 0.008 | 2.48E-08 | -0.003 | 0.012 | 0.753 |
|  | rs17168486 | C | T | -0.074 | 0.009 | 2.18E-15 | 0.001 | 0.014 | 0.952 |
|  | rs17405722 | A | G | 0.087 | 0.015 | 2.28E-09 | -0.038 | 0.022 | 0.095 |
|  | rs17631783 | C | T | 0.049 | 0.009 | 3.95E-08 | 0.008 | 0.013 | 0.482 |
|  | rs17791513 | A | G | 0.103 | 0.015 | 4.61E-12 | 0.010 | 0.022 | 0.675 |
|  | rs1801214 | C | T | 0.090 | 0.007 | 5.52E-34 | -0.002 | 0.011 | 0.789 |
|  | rs1899951 | T | C | -0.112 | 0.011 | 1.64E-24 | 0.007 | 0.016 | 0.604 |
|  | rs2237892 | C | T | 0.096 | 0.016 | 8.75E-10 | 0.000 | 0.022 | 0.942 |
|  | rs2246618 | C | T | -0.051 | 0.008 | 1.20E-09 | -0.004 | 0.012 | 0.759 |
|  | rs2261181 | C | T | -0.099 | 0.012 | 9.18E-17 | -0.040 | 0.018 | 0.022 |
|  | rs2294120 | G | A | -0.044 | 0.008 | 1.62E-08 | -0.007 | 0.011 | 0.509 |
|  | rs2296173 | A | G | -0.065 | 0.009 | 7.66E-14 | 0.008 | 0.013 | 0.461 |
|  | rs2299383 | C | T | -0.041 | 0.007 | 1.49E-08 | 0.003 | 0.011 | 0.765 |
|  | rs243019 | T | C | -0.057 | 0.007 | 2.29E-15 | -0.009 | 0.011 | 0.428 |
|  | rs2493394 | G | A | 0.073 | 0.011 | 1.15E-10 | 0.014 | 0.018 | 0.488 |
|  | rs2796441 | G | A | 0.072 | 0.007 | 1.96E-22 | -0.026 | 0.011 | 0.018 |
|  | rs2820426 | G | A | 0.052 | 0.007 | 1.30E-12 | 0.010 | 0.011 | 0.375 |
|  | rs2867125 | T | C | -0.060 | 0.010 | 4.33E-10 | -0.014 | 0.015 | 0.319 |
|  | rs2908282 | G | A | -0.055 | 0.009 | 4.25E-09 | -0.007 | 0.014 | 0.599 |
|  | rs2925979 | T | C | 0.053 | 0.008 | 9.06E-12 | 0.004 | 0.012 | 0.742 |
|  | rs2943656 | A | G | -0.090 | 0.007 | 6.70E-34 | 0.011 | 0.012 | 0.336 |
|  | rs3217992 | T | C | 0.053 | 0.007 | 7.23E-13 | 0.008 | 0.011 | 0.483 |
|  | rs340874 | T | C | -0.063 | 0.007 | 8.41E-18 | -0.010 | 0.011 | 0.346 |
|  | rs348330 | G | A | 0.049 | 0.008 | 1.86E-09 | 0.005 | 0.012 | 0.650 |
|  | rs3756784 | G | T | 0.051 | 0.009 | 2.59E-08 | -0.006 | 0.013 | 0.697 |
|  | rs3802177 | G | A | 0.122 | 0.008 | 2.32E-52 | 0.017 | 0.012 | 0.147 |
|  | rs459193 | G | A | 0.071 | 0.008 | 8.81E-18 | 0.002 | 0.013 | 0.872 |
|  | rs4622883 | A | G | 0.044 | 0.008 | 3.02E-08 | -0.002 | 0.011 | 0.864 |
|  | rs4686471 | C | T | 0.053 | 0.008 | 4.28E-11 | 0.009 | 0.011 | 0.474 |
|  | rs4812829 | A | G | 0.053 | 0.010 | 2.44E-08 | -0.001 | 0.014 | 0.910 |
|  | rs4823182 | A | G | -0.048 | 0.008 | 3.36E-10 | 0.012 | 0.011 | 0.294 |
|  | rs4865796 | A | G | 0.053 | 0.008 | 1.33E-11 | -0.010 | 0.012 | 0.466 |
|  | rs516946 | T | C | -0.082 | 0.009 | 3.16E-22 | 0.007 | 0.013 | 0.590 |
|  | rs5215 | C | T | 0.068 | 0.007 | 2.09E-20 | 0.010 | 0.011 | 0.406 |
|  | rs576674 | A | G | -0.065 | 0.010 | 1.79E-11 | -0.006 | 0.014 | 0.656 |
|  | rs6059662 | A | G | -0.045 | 0.008 | 1.51E-08 | 0.013 | 0.012 | 0.275 |
|  | rs61953351 | G | T | 0.070 | 0.009 | 1.98E-14 | 0.028 | 0.013 | 0.027 |
|  | rs622217 | C | T | -0.049 | 0.008 | 3.13E-10 | 0.007 | 0.011 | 0.528 |
|  | rs6515236 | A | C | 0.050 | 0.009 | 3.34E-08 | 0.007 | 0.012 | 0.570 |
|  | rs67232546 | C | T | -0.060 | 0.010 | 4.66E-10 | -0.010 | 0.014 | 0.470 |
|  | rs6767484 | A | G | -0.121 | 0.008 | 2.70E-56 | 0.001 | 0.012 | 0.941 |
|  | rs6785040 | C | T | -0.063 | 0.011 | 1.26E-08 | 0.019 | 0.015 | 0.165 |
|  | rs6795735 | C | T | 0.056 | 0.007 | 1.63E-14 | -0.004 | 0.011 | 0.687 |
|  | rs6878122 | A | G | -0.056 | 0.008 | 1.19E-12 | 0.018 | 0.012 | 0.164 |
|  | rs6960043 | C | T | 0.064 | 0.007 | 3.61E-19 | -0.011 | 0.011 | 0.340 |
|  | rs7144011 | G | T | -0.048 | 0.009 | 1.64E-08 | -0.007 | 0.014 | 0.603 |
|  | rs7177055 | A | G | 0.065 | 0.008 | 2.75E-16 | -0.004 | 0.012 | 0.740 |
|  | rs7240767 | C | T | 0.045 | 0.008 | 2.16E-08 | 0.004 | 0.011 | 0.719 |
|  | rs72892910 | G | T | -0.065 | 0.010 | 6.43E-11 | -0.019 | 0.014 | 0.205 |
|  | rs735949 | C | T | -0.071 | 0.011 | 1.95E-11 | -0.012 | 0.016 | 0.475 |
|  | rs753270 | C | T | 0.053 | 0.008 | 2.70E-11 | 0.010 | 0.011 | 0.351 |
|  | rs7561798 | A | G | -0.040 | 0.007 | 2.79E-08 | 0.000 | 0.011 | 0.975 |
|  | rs7572970 | A | G | -0.059 | 0.009 | 1.39E-11 | 0.000 | 0.012 | 0.966 |
|  | rs7607777 | G | T | 0.137 | 0.013 | 9.40E-28 | 0.007 | 0.018 | 0.719 |
|  | rs7674212 | G | T | 0.047 | 0.008 | 6.18E-10 | 0.003 | 0.011 | 0.802 |
|  | rs7685296 | C | T | 0.051 | 0.008 | 2.32E-10 | 0.012 | 0.012 | 0.335 |
|  | rs7756992 | A | G | -0.130 | 0.008 | 6.00E-62 | 0.001 | 0.012 | 0.948 |
|  | rs7786095 | A | G | 0.074 | 0.013 | 9.64E-09 | 0.003 | 0.019 | 0.885 |
|  | rs780094 | T | C | -0.069 | 0.007 | 5.16E-21 | -0.010 | 0.011 | 0.358 |
|  | rs7845219 | C | T | -0.042 | 0.007 | 4.54E-09 | 0.007 | 0.011 | 0.559 |
|  | rs7903146 | C | T | -0.306 | 0.008 | 1.00E-200 | 0.014 | 0.012 | 0.264 |
|  | rs7929543 | A | C | -0.083 | 0.014 | 2.20E-09 | -0.001 | 0.019 | 0.928 |
|  | rs7955901 | C | T | 0.044 | 0.007 | 7.16E-10 | 0.010 | 0.011 | 0.348 |
|  | rs8068804 | A | G | 0.059 | 0.008 | 4.41E-14 | -0.007 | 0.012 | 0.551 |
|  | rs8108269 | G | T | 0.064 | 0.008 | 3.11E-16 | -0.001 | 0.012 | 0.935 |
|  | rs825476 | C | T | -0.052 | 0.007 | 6.80E-13 | -0.003 | 0.011 | 0.764 |
|  | rs840967 | A | C | -0.050 | 0.008 | 5.44E-10 | -0.011 | 0.011 | 0.354 |
|  | rs849135 | A | G | -0.100 | 0.007 | 1.04E-43 | -0.014 | 0.011 | 0.218 |
|  | rs853974 | C | T | -0.060 | 0.009 | 7.86E-12 | -0.019 | 0.012 | 0.124 |
|  | rs9369425 | A | G | -0.055 | 0.009 | 1.13E-10 | -0.009 | 0.012 | 0.426 |
|  | rs9894220 | A | G | 0.059 | 0.008 | 1.52E-13 | 0.017 | 0.011 | 0.141 |
|  | rs9928094 | A | G | -0.105 | 0.007 | 3.59E-47 | -0.011 | 0.011 | 0.349 |
|  | rs993380 | A | G | 0.051 | 0.008 | 4.59E-10 | -0.014 | 0.011 | 0.210 |
|  | rs9940149 | A | G | -0.058 | 0.010 | 9.29E-10 | -0.010 | 0.014 | 0.425 |
| *Bacteroidaceae* | rs10077431 | A | C | -0.049 | 0.009 | 4.75E-08 | -0.001 | 0.013 | 0.999 |
|  | rs10087241 | A | G | -0.048 | 0.008 | 2.80E-09 | 0.003 | 0.011 | 0.732 |
|  | rs10100265 | A | C | 0.049 | 0.008 | 6.29E-10 | -0.018 | 0.011 | 0.088 |
|  | rs10114341 | C | T | -0.041 | 0.007 | 1.15E-08 | -0.009 | 0.011 | 0.418 |
|  | rs10401969 | C | T | 0.092 | 0.013 | 4.13E-12 | -0.039 | 0.020 | 0.046 |
|  | rs1050226 | G | A | -0.049 | 0.007 | 3.34E-11 | -0.004 | 0.011 | 0.741 |
|  | rs1061813 | A | G | -0.043 | 0.007 | 3.37E-09 | -0.014 | 0.011 | 0.181 |
|  | rs1063355 | T | G | -0.071 | 0.008 | 3.72E-19 | -0.008 | 0.011 | 0.451 |
|  | rs10740322 | A | G | 0.048 | 0.009 | 2.11E-08 | -0.005 | 0.011 | 0.608 |
|  | rs10811661 | C | T | -0.157 | 0.010 | 4.13E-58 | -0.020 | 0.014 | 0.157 |
|  | rs10842994 | C | T | 0.076 | 0.009 | 1.02E-16 | -0.002 | 0.013 | 0.886 |
|  | rs10974438 | A | C | -0.059 | 0.008 | 3.01E-15 | 0.023 | 0.011 | 0.035 |
|  | rs11098676 | C | T | 0.054 | 0.010 | 2.03E-08 | 0.014 | 0.013 | 0.247 |
|  | rs11107116 | G | T | -0.047 | 0.009 | 3.75E-08 | 0.007 | 0.013 | 0.629 |
|  | rs1111875 | T | C | -0.095 | 0.007 | 3.61E-39 | 0.018 | 0.011 | 0.097 |
|  | rs11257655 | C | T | -0.074 | 0.009 | 1.97E-17 | 0.025 | 0.013 | 0.045 |
|  | rs1127655 | C | T | 0.044 | 0.008 | 2.47E-08 | -0.003 | 0.010 | 0.774 |
|  | rs11708067 | A | G | 0.097 | 0.009 | 5.93E-29 | -0.015 | 0.013 | 0.275 |
|  | rs11925227 | A | G | -0.053 | 0.010 | 2.25E-08 | 0.002 | 0.014 | 0.839 |
|  | rs11926707 | C | T | 0.046 | 0.008 | 1.69E-08 | 0.014 | 0.011 | 0.201 |
|  | rs12088739 | A | G | 0.088 | 0.013 | 9.79E-12 | -0.010 | 0.018 | 0.513 |
|  | rs12299509 | A | G | -0.047 | 0.007 | 2.09E-10 | 0.008 | 0.010 | 0.442 |
|  | rs12617659 | C | T | 0.069 | 0.010 | 2.83E-11 | -0.012 | 0.015 | 0.431 |
|  | rs12910825 | A | G | -0.052 | 0.007 | 2.16E-12 | -0.004 | 0.011 | 0.710 |
|  | rs12945601 | C | T | -0.048 | 0.008 | 1.72E-09 | -0.001 | 0.011 | 0.995 |
|  | rs12970134 | A | G | 0.056 | 0.008 | 5.31E-12 | 0.022 | 0.012 | 0.058 |
|  | rs13239186 | C | T | -0.054 | 0.009 | 2.70E-10 | -0.013 | 0.011 | 0.264 |
|  | rs13330951 | A | G | 0.046 | 0.008 | 1.54E-08 | -0.013 | 0.011 | 0.182 |
|  | rs13389219 | C | T | 0.072 | 0.007 | 2.11E-22 | 0.019 | 0.011 | 0.081 |
|  | rs1359790 | G | A | 0.080 | 0.008 | 2.80E-23 | -0.009 | 0.012 | 0.415 |
|  | rs1496653 | G | A | -0.077 | 0.009 | 2.57E-18 | 0.009 | 0.013 | 0.433 |
|  | rs1552224 | C | A | -0.103 | 0.010 | 8.64E-25 | 0.028 | 0.015 | 0.052 |
|  | rs16988333 | A | G | 0.075 | 0.013 | 9.17E-09 | -0.017 | 0.021 | 0.419 |
|  | rs17086692 | G | T | 0.047 | 0.008 | 2.48E-08 | -0.001 | 0.011 | 0.942 |
|  | rs17168486 | C | T | -0.074 | 0.009 | 2.18E-15 | -0.022 | 0.013 | 0.084 |
|  | rs17405722 | A | G | 0.087 | 0.015 | 2.28E-09 | -0.024 | 0.021 | 0.257 |
|  | rs17631783 | C | T | 0.049 | 0.009 | 3.95E-08 | 0.002 | 0.012 | 0.965 |
|  | rs17791513 | A | G | 0.103 | 0.015 | 4.61E-12 | 0.012 | 0.021 | 0.542 |
|  | rs1801214 | C | T | 0.090 | 0.007 | 5.52E-34 | -0.017 | 0.011 | 0.129 |
|  | rs1899951 | T | C | -0.112 | 0.011 | 1.64E-24 | 0.020 | 0.015 | 0.143 |
|  | rs2237892 | C | T | 0.096 | 0.016 | 8.75E-10 | -0.031 | 0.021 | 0.044 |
|  | rs2246618 | C | T | -0.051 | 0.008 | 1.20E-09 | -0.004 | 0.011 | 0.749 |
|  | rs2261181 | C | T | -0.099 | 0.012 | 9.18E-17 | 0.009 | 0.018 | 0.524 |
|  | rs2294120 | G | A | -0.044 | 0.008 | 1.62E-08 | -0.003 | 0.010 | 0.855 |
|  | rs2296173 | A | G | -0.065 | 0.009 | 7.66E-14 | 0.024 | 0.013 | 0.070 |
|  | rs2299383 | C | T | -0.041 | 0.007 | 1.49E-08 | 0.015 | 0.011 | 0.153 |
|  | rs243019 | T | C | -0.057 | 0.007 | 2.29E-15 | 0.007 | 0.011 | 0.532 |
|  | rs2493394 | G | A | 0.073 | 0.011 | 1.15E-10 | 0.003 | 0.017 | 0.941 |
|  | rs2796441 | G | A | 0.072 | 0.007 | 1.96E-22 | -0.026 | 0.011 | 0.017 |
|  | rs2820426 | G | A | 0.052 | 0.007 | 1.30E-12 | 0.013 | 0.011 | 0.215 |
|  | rs2867125 | T | C | -0.060 | 0.010 | 4.33E-10 | 0.006 | 0.014 | 0.797 |
|  | rs2908282 | G | A | -0.055 | 0.009 | 4.25E-09 | 0.011 | 0.014 | 0.410 |
|  | rs2925979 | T | C | 0.053 | 0.008 | 9.06E-12 | -0.002 | 0.011 | 0.851 |
|  | rs2943656 | A | G | -0.090 | 0.007 | 6.70E-34 | 0.007 | 0.011 | 0.557 |
|  | rs3217992 | T | C | 0.053 | 0.007 | 7.23E-13 | 0.001 | 0.011 | 0.943 |
|  | rs340874 | T | C | -0.063 | 0.007 | 8.41E-18 | -0.006 | 0.011 | 0.568 |
|  | rs348330 | G | A | 0.049 | 0.008 | 1.86E-09 | 0.012 | 0.011 | 0.259 |
|  | rs3756784 | G | T | 0.051 | 0.009 | 2.59E-08 | 0.002 | 0.013 | 0.945 |
|  | rs3802177 | G | A | 0.122 | 0.008 | 2.32E-52 | 0.010 | 0.011 | 0.421 |
|  | rs459193 | G | A | 0.071 | 0.008 | 8.81E-18 | -0.003 | 0.012 | 0.752 |
|  | rs4622883 | A | G | 0.044 | 0.008 | 3.02E-08 | 0.004 | 0.011 | 0.684 |
|  | rs4686471 | C | T | 0.053 | 0.008 | 4.28E-11 | 0.004 | 0.011 | 0.679 |
|  | rs4812829 | A | G | 0.053 | 0.010 | 2.44E-08 | -0.001 | 0.014 | 0.954 |
|  | rs4823182 | A | G | -0.048 | 0.008 | 3.36E-10 | -0.013 | 0.011 | 0.243 |
|  | rs4865796 | A | G | 0.053 | 0.008 | 1.33E-11 | -0.004 | 0.011 | 0.731 |
|  | rs516946 | T | C | -0.082 | 0.009 | 3.16E-22 | -0.002 | 0.012 | 0.886 |
|  | rs5215 | C | T | 0.068 | 0.007 | 2.09E-20 | -0.008 | 0.011 | 0.463 |
|  | rs576674 | A | G | -0.065 | 0.010 | 1.79E-11 | 0.008 | 0.014 | 0.550 |
|  | rs6059662 | A | G | -0.045 | 0.008 | 1.51E-08 | 0.003 | 0.012 | 0.786 |
|  | rs61953351 | G | T | 0.070 | 0.009 | 1.98E-14 | -0.015 | 0.012 | 0.248 |
|  | rs622217 | C | T | -0.049 | 0.008 | 3.13E-10 | 0.010 | 0.010 | 0.332 |
|  | rs6515236 | A | C | 0.050 | 0.009 | 3.34E-08 | 0.022 | 0.012 | 0.059 |
|  | rs67232546 | C | T | -0.060 | 0.010 | 4.66E-10 | -0.008 | 0.013 | 0.538 |
|  | rs6767484 | A | G | -0.121 | 0.008 | 2.70E-56 | -0.001 | 0.011 | 0.958 |
|  | rs6785040 | C | T | -0.063 | 0.011 | 1.26E-08 | 0.001 | 0.014 | 0.796 |
|  | rs6795735 | C | T | 0.056 | 0.007 | 1.63E-14 | 0.002 | 0.011 | 0.854 |
|  | rs6878122 | A | G | -0.056 | 0.008 | 1.19E-12 | 0.010 | 0.012 | 0.399 |
|  | rs6960043 | C | T | 0.064 | 0.007 | 3.61E-19 | 0.015 | 0.011 | 0.141 |
|  | rs7144011 | G | T | -0.048 | 0.009 | 1.64E-08 | 0.017 | 0.013 | 0.211 |
|  | rs7177055 | A | G | 0.065 | 0.008 | 2.75E-16 | -0.006 | 0.011 | 0.573 |
|  | rs7240767 | C | T | 0.045 | 0.008 | 2.16E-08 | 0.010 | 0.011 | 0.356 |
|  | rs72892910 | G | T | -0.065 | 0.010 | 6.43E-11 | 0.003 | 0.014 | 0.777 |
|  | rs735949 | C | T | -0.071 | 0.011 | 1.95E-11 | -0.012 | 0.016 | 0.488 |
|  | rs753270 | C | T | 0.053 | 0.008 | 2.70E-11 | -0.015 | 0.011 | 0.164 |
|  | rs7561798 | A | G | -0.040 | 0.007 | 2.79E-08 | 0.012 | 0.011 | 0.233 |
|  | rs7572970 | A | G | -0.059 | 0.009 | 1.39E-11 | -0.001 | 0.012 | 0.954 |
|  | rs7607777 | G | T | 0.137 | 0.013 | 9.40E-28 | 0.005 | 0.017 | 0.748 |
|  | rs7674212 | G | T | 0.047 | 0.008 | 6.18E-10 | -0.006 | 0.011 | 0.544 |
|  | rs7685296 | C | T | 0.051 | 0.008 | 2.32E-10 | 0.023 | 0.012 | 0.042 |
|  | rs7729395 | C | T | -0.137 | 0.016 | 1.10E-17 | 0.022 | 0.056 | 0.710 |
|  | rs7756992 | A | G | -0.130 | 0.008 | 6.00E-62 | -0.001 | 0.011 | 0.902 |
|  | rs7786095 | A | G | 0.074 | 0.013 | 9.64E-09 | -0.030 | 0.018 | 0.115 |
|  | rs780094 | T | C | -0.069 | 0.007 | 5.16E-21 | 0.006 | 0.011 | 0.562 |
|  | rs7845219 | C | T | -0.042 | 0.007 | 4.54E-09 | 0.004 | 0.011 | 0.668 |
|  | rs7903146 | C | T | -0.306 | 0.008 | 1.00E-200 | 0.025 | 0.012 | 0.034 |
|  | rs7929543 | A | C | -0.083 | 0.014 | 2.20E-09 | 0.005 | 0.018 | 0.783 |
|  | rs7955901 | C | T | 0.044 | 0.007 | 7.16E-10 | -0.006 | 0.011 | 0.584 |
|  | rs8068804 | A | G | 0.059 | 0.008 | 4.41E-14 | 0.001 | 0.011 | 0.887 |
|  | rs8108269 | G | T | 0.064 | 0.008 | 3.11E-16 | 0.012 | 0.011 | 0.295 |
|  | rs825476 | C | T | -0.052 | 0.007 | 6.80E-13 | -0.018 | 0.011 | 0.099 |
|  | rs840967 | A | C | -0.050 | 0.008 | 5.44E-10 | 0.004 | 0.011 | 0.697 |
|  | rs849135 | A | G | -0.100 | 0.007 | 1.04E-43 | -0.003 | 0.011 | 0.756 |
|  | rs853974 | C | T | -0.060 | 0.009 | 7.86E-12 | 0.006 | 0.012 | 0.571 |
|  | rs9369425 | A | G | -0.055 | 0.009 | 1.13E-10 | 0.007 | 0.012 | 0.581 |
|  | rs9894220 | A | G | 0.059 | 0.008 | 1.52E-13 | 0.017 | 0.011 | 0.121 |
|  | rs9928094 | A | G | -0.105 | 0.007 | 3.59E-47 | -0.007 | 0.011 | 0.484 |
|  | rs993380 | A | G | 0.051 | 0.008 | 4.59E-10 | -0.010 | 0.011 | 0.374 |
|  | rs9940149 | A | G | -0.058 | 0.010 | 9.29E-10 | -0.016 | 0.013 | 0.300 |
| *Bacteroidales_S24-7* | rs10077431 | A | C | -0.049 | 0.009 | 4.75E-08 | 0.017 | 0.020 | 0.326 |
|  | rs10087241 | A | G | -0.048 | 0.008 | 2.80E-09 | 0.034 | 0.016 | 0.036 |
|  | rs10100265 | A | C | 0.049 | 0.008 | 6.29E-10 | -0.010 | 0.016 | 0.546 |
|  | rs10114341 | C | T | -0.041 | 0.007 | 1.15E-08 | -0.021 | 0.016 | 0.228 |
|  | rs10401969 | C | T | 0.092 | 0.013 | 4.13E-12 | 0.016 | 0.030 | 0.635 |
|  | rs1050226 | G | A | -0.049 | 0.007 | 3.34E-11 | 0.025 | 0.016 | 0.129 |
|  | rs1061813 | A | G | -0.043 | 0.007 | 3.37E-09 | 0.024 | 0.016 | 0.151 |
|  | rs1063355 | T | G | -0.071 | 0.008 | 3.72E-19 | 0.003 | 0.016 | 0.827 |
|  | rs10740322 | A | G | 0.048 | 0.009 | 2.11E-08 | -0.011 | 0.017 | 0.491 |
|  | rs10811661 | C | T | -0.157 | 0.010 | 4.13E-58 | -0.007 | 0.021 | 0.666 |
|  | rs10842994 | C | T | 0.076 | 0.009 | 1.02E-16 | 0.009 | 0.020 | 0.704 |
|  | rs10974438 | A | C | -0.059 | 0.008 | 3.01E-15 | -0.021 | 0.016 | 0.200 |
|  | rs11098676 | C | T | 0.054 | 0.010 | 2.03E-08 | 0.012 | 0.019 | 0.535 |
|  | rs11107116 | G | T | -0.047 | 0.009 | 3.75E-08 | 0.000 | 0.019 | 0.938 |
|  | rs1111875 | T | C | -0.095 | 0.007 | 3.61E-39 | -0.005 | 0.016 | 0.784 |
|  | rs11257655 | C | T | -0.074 | 0.009 | 1.97E-17 | 0.009 | 0.019 | 0.656 |
|  | rs1127655 | C | T | 0.044 | 0.008 | 2.47E-08 | 0.009 | 0.016 | 0.571 |
|  | rs11708067 | A | G | 0.097 | 0.009 | 5.93E-29 | 0.015 | 0.019 | 0.421 |
|  | rs11925227 | A | G | -0.053 | 0.010 | 2.25E-08 | 0.019 | 0.021 | 0.385 |
|  | rs11926707 | C | T | 0.046 | 0.008 | 1.69E-08 | 0.004 | 0.017 | 0.813 |
|  | rs12088739 | A | G | 0.088 | 0.013 | 9.79E-12 | -0.024 | 0.028 | 0.409 |
|  | rs12299509 | A | G | -0.047 | 0.007 | 2.09E-10 | -0.031 | 0.016 | 0.051 |
|  | rs12617659 | C | T | 0.069 | 0.010 | 2.83E-11 | 0.011 | 0.023 | 0.648 |
|  | rs12910825 | A | G | -0.052 | 0.007 | 2.16E-12 | -0.017 | 0.016 | 0.290 |
|  | rs12945601 | C | T | -0.048 | 0.008 | 1.72E-09 | 0.000 | 0.016 | 0.985 |
|  | rs12970134 | A | G | 0.056 | 0.008 | 5.31E-12 | -0.020 | 0.018 | 0.279 |
|  | rs13239186 | C | T | -0.054 | 0.009 | 2.70E-10 | -0.036 | 0.017 | 0.040 |
|  | rs13330951 | A | G | 0.046 | 0.008 | 1.54E-08 | 0.014 | 0.016 | 0.342 |
|  | rs13389219 | C | T | 0.072 | 0.007 | 2.11E-22 | 0.042 | 0.017 | 0.008 |
|  | rs1359790 | G | A | 0.080 | 0.008 | 2.80E-23 | 0.028 | 0.018 | 0.127 |
|  | rs1496653 | G | A | -0.077 | 0.009 | 2.57E-18 | -0.020 | 0.020 | 0.269 |
|  | rs1552224 | C | A | -0.103 | 0.010 | 8.64E-25 | -0.005 | 0.022 | 0.954 |
|  | rs16988333 | A | G | 0.075 | 0.013 | 9.17E-09 | -0.026 | 0.031 | 0.409 |
|  | rs17086692 | G | T | 0.047 | 0.008 | 2.48E-08 | -0.019 | 0.017 | 0.257 |
|  | rs17168486 | C | T | -0.074 | 0.009 | 2.18E-15 | 0.013 | 0.020 | 0.537 |
|  | rs17405722 | A | G | 0.087 | 0.015 | 2.28E-09 | -0.023 | 0.032 | 0.411 |
|  | rs17631783 | C | T | 0.049 | 0.009 | 3.95E-08 | -0.017 | 0.018 | 0.364 |
|  | rs17791513 | A | G | 0.103 | 0.015 | 4.61E-12 | 0.014 | 0.032 | 0.512 |
|  | rs1801214 | C | T | 0.090 | 0.007 | 5.52E-34 | 0.013 | 0.016 | 0.457 |
|  | rs1899951 | T | C | -0.112 | 0.011 | 1.64E-24 | 0.022 | 0.023 | 0.347 |
|  | rs2237892 | C | T | 0.096 | 0.016 | 8.75E-10 | -0.031 | 0.032 | 0.228 |
|  | rs2246618 | C | T | -0.051 | 0.008 | 1.20E-09 | -0.029 | 0.017 | 0.083 |
|  | rs2261181 | C | T | -0.099 | 0.012 | 9.18E-17 | -0.003 | 0.027 | 0.992 |
|  | rs2294120 | G | A | -0.044 | 0.008 | 1.62E-08 | -0.018 | 0.016 | 0.268 |
|  | rs2296173 | A | G | -0.065 | 0.009 | 7.66E-14 | -0.001 | 0.020 | 0.909 |
|  | rs2299383 | C | T | -0.041 | 0.007 | 1.49E-08 | 0.014 | 0.016 | 0.366 |
|  | rs243019 | T | C | -0.057 | 0.007 | 2.29E-15 | 0.028 | 0.016 | 0.083 |
|  | rs2493394 | G | A | 0.073 | 0.011 | 1.15E-10 | -0.011 | 0.026 | 0.690 |
|  | rs2796441 | G | A | 0.072 | 0.007 | 1.96E-22 | -0.013 | 0.016 | 0.429 |
|  | rs2820426 | G | A | 0.052 | 0.007 | 1.30E-12 | -0.002 | 0.016 | 0.907 |
|  | rs2867125 | T | C | -0.060 | 0.010 | 4.33E-10 | 0.008 | 0.021 | 0.751 |
|  | rs2908282 | G | A | -0.055 | 0.009 | 4.25E-09 | -0.019 | 0.021 | 0.362 |
|  | rs2925979 | T | C | 0.053 | 0.008 | 9.06E-12 | -0.020 | 0.017 | 0.249 |
|  | rs2943656 | A | G | -0.090 | 0.007 | 6.70E-34 | 0.004 | 0.017 | 0.925 |
|  | rs3217992 | T | C | 0.053 | 0.007 | 7.23E-13 | -0.019 | 0.016 | 0.253 |
|  | rs340874 | T | C | -0.063 | 0.007 | 8.41E-18 | -0.015 | 0.016 | 0.362 |
|  | rs348330 | G | A | 0.049 | 0.008 | 1.86E-09 | -0.005 | 0.017 | 0.795 |
|  | rs3756784 | G | T | 0.051 | 0.009 | 2.59E-08 | 0.013 | 0.020 | 0.466 |
|  | rs3802177 | G | A | 0.122 | 0.008 | 2.32E-52 | -0.036 | 0.017 | 0.039 |
|  | rs459193 | G | A | 0.071 | 0.008 | 8.81E-18 | -0.008 | 0.018 | 0.662 |
|  | rs4622883 | A | G | 0.044 | 0.008 | 3.02E-08 | -0.015 | 0.016 | 0.319 |
|  | rs4686471 | C | T | 0.053 | 0.008 | 4.28E-11 | -0.003 | 0.017 | 0.961 |
|  | rs4812829 | A | G | 0.053 | 0.010 | 2.44E-08 | -0.025 | 0.020 | 0.168 |
|  | rs4823182 | A | G | -0.048 | 0.008 | 3.36E-10 | 0.013 | 0.017 | 0.413 |
|  | rs4865796 | A | G | 0.053 | 0.008 | 1.33E-11 | 0.003 | 0.017 | 0.848 |
|  | rs516946 | T | C | -0.082 | 0.009 | 3.16E-22 | 0.043 | 0.019 | 0.027 |
|  | rs5215 | C | T | 0.068 | 0.007 | 2.09E-20 | 0.016 | 0.016 | 0.327 |
|  | rs576674 | A | G | -0.065 | 0.010 | 1.79E-11 | 0.010 | 0.021 | 0.593 |
|  | rs6059662 | A | G | -0.045 | 0.008 | 1.51E-08 | -0.010 | 0.018 | 0.604 |
|  | rs61953351 | G | T | 0.070 | 0.009 | 1.98E-14 | 0.029 | 0.019 | 0.149 |
|  | rs622217 | C | T | -0.049 | 0.008 | 3.13E-10 | 0.001 | 0.016 | 0.971 |
|  | rs6515236 | A | C | 0.050 | 0.009 | 3.34E-08 | -0.019 | 0.018 | 0.273 |
|  | rs67232546 | C | T | -0.060 | 0.010 | 4.66E-10 | -0.008 | 0.020 | 0.727 |
|  | rs6767484 | A | G | -0.121 | 0.008 | 2.70E-56 | -0.030 | 0.017 | 0.082 |
|  | rs6785040 | C | T | -0.063 | 0.011 | 1.26E-08 | 0.004 | 0.021 | 0.823 |
|  | rs6795735 | C | T | 0.056 | 0.007 | 1.63E-14 | -0.013 | 0.016 | 0.381 |
|  | rs6878122 | A | G | -0.056 | 0.008 | 1.19E-12 | -0.042 | 0.018 | 0.018 |
|  | rs6960043 | C | T | 0.064 | 0.007 | 3.61E-19 | 0.017 | 0.016 | 0.284 |
|  | rs7144011 | G | T | -0.048 | 0.009 | 1.64E-08 | -0.014 | 0.020 | 0.477 |
|  | rs7177055 | A | G | 0.065 | 0.008 | 2.75E-16 | -0.017 | 0.017 | 0.289 |
|  | rs7240767 | C | T | 0.045 | 0.008 | 2.16E-08 | 0.013 | 0.016 | 0.404 |
|  | rs72892910 | G | T | -0.065 | 0.010 | 6.43E-11 | -0.026 | 0.021 | 0.234 |
|  | rs735949 | C | T | -0.071 | 0.011 | 1.95E-11 | 0.000 | 0.024 | 0.935 |
|  | rs753270 | C | T | 0.053 | 0.008 | 2.70E-11 | 0.035 | 0.016 | 0.032 |
|  | rs7561798 | A | G | -0.040 | 0.007 | 2.79E-08 | 0.010 | 0.016 | 0.544 |
|  | rs7572970 | A | G | -0.059 | 0.009 | 1.39E-11 | -0.008 | 0.018 | 0.684 |
|  | rs7607777 | G | T | 0.137 | 0.013 | 9.40E-28 | 0.054 | 0.027 | 0.051 |
|  | rs7674212 | G | T | 0.047 | 0.008 | 6.18E-10 | 0.008 | 0.016 | 0.632 |
|  | rs7685296 | C | T | 0.051 | 0.008 | 2.32E-10 | -0.004 | 0.018 | 0.815 |
|  | rs7756992 | A | G | -0.130 | 0.008 | 6.00E-62 | 0.015 | 0.017 | 0.368 |
|  | rs7786095 | A | G | 0.074 | 0.013 | 9.64E-09 | -0.003 | 0.028 | 0.903 |
|  | rs780094 | T | C | -0.069 | 0.007 | 5.16E-21 | -0.021 | 0.016 | 0.209 |
|  | rs7845219 | C | T | -0.042 | 0.007 | 4.54E-09 | -0.016 | 0.016 | 0.336 |
|  | rs7903146 | C | T | -0.306 | 0.008 | 1.00E-200 | 0.009 | 0.018 | 0.616 |
|  | rs7929543 | A | C | -0.083 | 0.014 | 2.20E-09 | 0.035 | 0.028 | 0.217 |
|  | rs7955901 | C | T | 0.044 | 0.007 | 7.16E-10 | -0.026 | 0.016 | 0.109 |
|  | rs8068804 | A | G | 0.059 | 0.008 | 4.41E-14 | -0.009 | 0.017 | 0.600 |
|  | rs8108269 | G | T | 0.064 | 0.008 | 3.11E-16 | 0.018 | 0.017 | 0.298 |
|  | rs825476 | C | T | -0.052 | 0.007 | 6.80E-13 | 0.009 | 0.016 | 0.573 |
|  | rs840967 | A | C | -0.050 | 0.008 | 5.44E-10 | -0.011 | 0.016 | 0.507 |
|  | rs849135 | A | G | -0.100 | 0.007 | 1.04E-43 | 0.033 | 0.016 | 0.044 |
|  | rs853974 | C | T | -0.060 | 0.009 | 7.86E-12 | -0.016 | 0.018 | 0.347 |
|  | rs9369425 | A | G | -0.055 | 0.009 | 1.13E-10 | 0.023 | 0.018 | 0.202 |
|  | rs9894220 | A | G | 0.059 | 0.008 | 1.52E-13 | 0.004 | 0.016 | 0.841 |
|  | rs9928094 | A | G | -0.105 | 0.007 | 3.59E-47 | -0.030 | 0.016 | 0.066 |
|  | rs993380 | A | G | 0.051 | 0.008 | 4.59E-10 | -0.006 | 0.017 | 0.742 |
|  | rs9940149 | A | G | -0.058 | 0.010 | 9.29E-10 | 0.010 | 0.020 | 0.630 |
| *Bifidobacteriaceae* | rs10077431 | A | C | -0.049 | 0.009 | 4.75E-08 | 0.017 | 0.015 | 0.296 |
|  | rs10401969 | C | T | 0.092 | 0.013 | 4.13E-12 | 0.030 | 0.022 | 0.200 |
|  | rs1050226 | G | A | -0.049 | 0.007 | 3.34E-11 | 0.015 | 0.012 | 0.192 |
|  | rs1063355 | T | G | -0.071 | 0.008 | 3.72E-19 | 0.010 | 0.012 | 0.402 |
|  | rs10740322 | A | G | 0.048 | 0.009 | 2.11E-08 | 0.021 | 0.012 | 0.082 |
|  | rs10974438 | A | C | -0.059 | 0.008 | 3.01E-15 | 0.015 | 0.012 | 0.214 |
|  | rs1127655 | C | T | 0.044 | 0.008 | 2.47E-08 | -0.023 | 0.012 | 0.046 |
|  | rs12088739 | A | G | 0.088 | 0.013 | 9.79E-12 | -0.016 | 0.020 | 0.410 |
|  | rs12299509 | A | G | -0.047 | 0.007 | 2.09E-10 | -0.025 | 0.012 | 0.029 |
|  | rs12970134 | A | G | 0.056 | 0.008 | 5.31E-12 | -0.021 | 0.013 | 0.112 |
|  | rs13239186 | C | T | -0.054 | 0.009 | 2.70E-10 | 0.013 | 0.013 | 0.322 |
|  | rs13330951 | A | G | 0.046 | 0.008 | 1.54E-08 | 0.012 | 0.012 | 0.351 |
|  | rs1496653 | G | A | -0.077 | 0.009 | 2.57E-18 | -0.012 | 0.014 | 0.390 |
|  | rs1552224 | C | A | -0.103 | 0.010 | 8.64E-25 | -0.025 | 0.016 | 0.099 |
|  | rs16988333 | A | G | 0.075 | 0.013 | 9.17E-09 | 0.040 | 0.023 | 0.088 |
|  | rs17168486 | C | T | -0.074 | 0.009 | 2.18E-15 | 0.039 | 0.015 | 0.007 |
|  | rs17405722 | A | G | 0.087 | 0.015 | 2.28E-09 | -0.018 | 0.023 | 0.405 |
|  | rs17631783 | C | T | 0.049 | 0.009 | 3.95E-08 | -0.025 | 0.013 | 0.065 |
|  | rs17791513 | A | G | 0.103 | 0.015 | 4.61E-12 | -0.038 | 0.023 | 0.120 |
|  | rs1899951 | T | C | -0.112 | 0.011 | 1.64E-24 | -0.030 | 0.017 | 0.117 |
|  | rs2261181 | C | T | -0.099 | 0.012 | 9.18E-17 | 0.017 | 0.019 | 0.379 |
|  | rs243019 | T | C | -0.057 | 0.007 | 2.29E-15 | -0.014 | 0.012 | 0.222 |
|  | rs2796441 | G | A | 0.072 | 0.007 | 1.96E-22 | -0.038 | 0.012 | 0.001 |
|  | rs2820426 | G | A | 0.052 | 0.007 | 1.30E-12 | 0.012 | 0.012 | 0.315 |
|  | rs2908282 | G | A | -0.055 | 0.009 | 4.25E-09 | 0.011 | 0.015 | 0.476 |
|  | rs2943656 | A | G | -0.090 | 0.007 | 6.70E-34 | 0.034 | 0.012 | 0.008 |
|  | rs3756784 | G | T | 0.051 | 0.009 | 2.59E-08 | 0.013 | 0.014 | 0.348 |
|  | rs3802177 | G | A | 0.122 | 0.008 | 2.32E-52 | -0.012 | 0.013 | 0.365 |
|  | rs4622883 | A | G | 0.044 | 0.008 | 3.02E-08 | -0.009 | 0.012 | 0.411 |
|  | rs4823182 | A | G | -0.048 | 0.008 | 3.36E-10 | 0.018 | 0.012 | 0.137 |
|  | rs4865796 | A | G | 0.053 | 0.008 | 1.33E-11 | -0.016 | 0.013 | 0.173 |
|  | rs5215 | C | T | 0.068 | 0.007 | 2.09E-20 | -0.016 | 0.012 | 0.201 |
|  | rs6059662 | A | G | -0.045 | 0.008 | 1.51E-08 | -0.014 | 0.013 | 0.291 |
|  | rs6515236 | A | C | 0.050 | 0.009 | 3.34E-08 | -0.022 | 0.013 | 0.086 |
|  | rs6767484 | A | G | -0.121 | 0.008 | 2.70E-56 | 0.015 | 0.013 | 0.253 |
|  | rs6785040 | C | T | -0.063 | 0.011 | 1.26E-08 | 0.015 | 0.016 | 0.273 |
|  | rs6878122 | A | G | -0.056 | 0.008 | 1.19E-12 | -0.011 | 0.013 | 0.380 |
|  | rs7240767 | C | T | 0.045 | 0.008 | 2.16E-08 | -0.011 | 0.012 | 0.366 |
|  | rs735949 | C | T | -0.071 | 0.011 | 1.95E-11 | 0.017 | 0.017 | 0.313 |
|  | rs753270 | C | T | 0.053 | 0.008 | 2.70E-11 | -0.017 | 0.012 | 0.153 |
|  | rs7561798 | A | G | -0.040 | 0.007 | 2.79E-08 | 0.015 | 0.012 | 0.200 |
|  | rs7572970 | A | G | -0.059 | 0.009 | 1.39E-11 | 0.015 | 0.013 | 0.265 |
|  | rs7674212 | G | T | 0.047 | 0.008 | 6.18E-10 | 0.012 | 0.012 | 0.315 |
|  | rs7729395 | C | T | -0.137 | 0.016 | 1.10E-17 | -0.096 | 0.057 | 0.097 |
|  | rs7786095 | A | G | 0.074 | 0.013 | 9.64E-09 | -0.026 | 0.020 | 0.209 |
|  | rs7903146 | C | T | -0.306 | 0.008 | 1.00E-200 | -0.040 | 0.013 | 0.002 |
|  | rs8068804 | A | G | 0.059 | 0.008 | 4.41E-14 | 0.031 | 0.013 | 0.016 |
|  | rs8108269 | G | T | 0.064 | 0.008 | 3.11E-16 | -0.015 | 0.013 | 0.219 |
|  | rs853974 | C | T | -0.060 | 0.009 | 7.86E-12 | 0.007 | 0.013 | 0.567 |
|  | rs9928094 | A | G | -0.105 | 0.007 | 3.59E-47 | -0.014 | 0.012 | 0.242 |
| *Christensenellaceae* | rs10077431 | A | C | -0.049 | 0.009 | 4.75E-08 | -0.030 | 0.014 | 0.033 |
|  | rs10087241 | A | G | -0.048 | 0.008 | 2.80E-09 | -0.001 | 0.011 | 0.963 |
|  | rs10100265 | A | C | 0.049 | 0.008 | 6.29E-10 | -0.001 | 0.011 | 0.930 |
|  | rs10114341 | C | T | -0.041 | 0.007 | 1.15E-08 | 0.003 | 0.011 | 0.699 |
|  | rs10401969 | C | T | 0.092 | 0.013 | 4.13E-12 | 0.019 | 0.021 | 0.322 |
|  | rs1050226 | G | A | -0.049 | 0.007 | 3.34E-11 | 0.007 | 0.011 | 0.517 |
|  | rs1061813 | A | G | -0.043 | 0.007 | 3.37E-09 | -0.011 | 0.011 | 0.335 |
|  | rs1063355 | T | G | -0.071 | 0.008 | 3.72E-19 | -0.003 | 0.011 | 0.781 |
|  | rs10740322 | A | G | 0.048 | 0.009 | 2.11E-08 | 0.009 | 0.012 | 0.438 |
|  | rs10811661 | C | T | -0.157 | 0.010 | 4.13E-58 | -0.002 | 0.014 | 0.818 |
|  | rs10842994 | C | T | 0.076 | 0.009 | 1.02E-16 | -0.027 | 0.014 | 0.051 |
|  | rs10974438 | A | C | -0.059 | 0.008 | 3.01E-15 | -0.009 | 0.011 | 0.430 |
|  | rs11098676 | C | T | 0.054 | 0.010 | 2.03E-08 | -0.041 | 0.013 | 0.002 |
|  | rs11107116 | G | T | -0.047 | 0.009 | 3.75E-08 | 0.011 | 0.013 | 0.442 |
|  | rs1111875 | T | C | -0.095 | 0.007 | 3.61E-39 | -0.007 | 0.011 | 0.561 |
|  | rs11257655 | C | T | -0.074 | 0.009 | 1.97E-17 | 0.017 | 0.013 | 0.220 |
|  | rs1127655 | C | T | 0.044 | 0.008 | 2.47E-08 | -0.004 | 0.011 | 0.686 |
|  | rs11708067 | A | G | 0.097 | 0.009 | 5.93E-29 | -0.008 | 0.013 | 0.515 |
|  | rs11925227 | A | G | -0.053 | 0.010 | 2.25E-08 | 0.008 | 0.014 | 0.567 |
|  | rs11926707 | C | T | 0.046 | 0.008 | 1.69E-08 | 0.003 | 0.011 | 0.803 |
|  | rs12088739 | A | G | 0.088 | 0.013 | 9.79E-12 | -0.017 | 0.019 | 0.420 |
|  | rs12299509 | A | G | -0.047 | 0.007 | 2.09E-10 | 0.012 | 0.011 | 0.273 |
|  | rs12617659 | C | T | 0.069 | 0.010 | 2.83E-11 | -0.007 | 0.016 | 0.607 |
|  | rs12910825 | A | G | -0.052 | 0.007 | 2.16E-12 | 0.021 | 0.011 | 0.055 |
|  | rs12945601 | C | T | -0.048 | 0.008 | 1.72E-09 | 0.003 | 0.011 | 0.764 |
|  | rs12970134 | A | G | 0.056 | 0.008 | 5.31E-12 | 0.000 | 0.012 | 0.968 |
|  | rs13239186 | C | T | -0.054 | 0.009 | 2.70E-10 | -0.025 | 0.012 | 0.033 |
|  | rs13330951 | A | G | 0.046 | 0.008 | 1.54E-08 | 0.007 | 0.011 | 0.559 |
|  | rs13389219 | C | T | 0.072 | 0.007 | 2.11E-22 | 0.015 | 0.011 | 0.183 |
|  | rs1359790 | G | A | 0.080 | 0.008 | 2.80E-23 | 0.008 | 0.012 | 0.574 |
|  | rs1496653 | G | A | -0.077 | 0.009 | 2.57E-18 | -0.006 | 0.013 | 0.631 |
|  | rs1552224 | C | A | -0.103 | 0.010 | 8.64E-25 | -0.026 | 0.015 | 0.101 |
|  | rs16988333 | A | G | 0.075 | 0.013 | 9.17E-09 | -0.024 | 0.021 | 0.263 |
|  | rs17086692 | G | T | 0.047 | 0.008 | 2.48E-08 | -0.025 | 0.012 | 0.034 |
|  | rs17168486 | C | T | -0.074 | 0.009 | 2.18E-15 | 0.012 | 0.014 | 0.293 |
|  | rs17405722 | A | G | 0.087 | 0.015 | 2.28E-09 | -0.013 | 0.022 | 0.547 |
|  | rs17631783 | C | T | 0.049 | 0.009 | 3.95E-08 | -0.010 | 0.012 | 0.527 |
|  | rs17791513 | A | G | 0.103 | 0.015 | 4.61E-12 | -0.002 | 0.022 | 0.997 |
|  | rs1801214 | C | T | 0.090 | 0.007 | 5.52E-34 | 0.005 | 0.011 | 0.656 |
|  | rs1899951 | T | C | -0.112 | 0.011 | 1.64E-24 | -0.001 | 0.016 | 0.906 |
|  | rs2237892 | C | T | 0.096 | 0.016 | 8.75E-10 | -0.003 | 0.022 | 0.989 |
|  | rs2246618 | C | T | -0.051 | 0.008 | 1.20E-09 | -0.006 | 0.012 | 0.548 |
|  | rs2261181 | C | T | -0.099 | 0.012 | 9.18E-17 | -0.013 | 0.018 | 0.638 |
|  | rs2294120 | G | A | -0.044 | 0.008 | 1.62E-08 | 0.012 | 0.011 | 0.284 |
|  | rs2296173 | A | G | -0.065 | 0.009 | 7.66E-14 | -0.018 | 0.013 | 0.167 |
|  | rs2299383 | C | T | -0.041 | 0.007 | 1.49E-08 | -0.011 | 0.011 | 0.328 |
|  | rs243019 | T | C | -0.057 | 0.007 | 2.29E-15 | 0.021 | 0.011 | 0.052 |
|  | rs2493394 | G | A | 0.073 | 0.011 | 1.15E-10 | 0.008 | 0.018 | 0.575 |
|  | rs2796441 | G | A | 0.072 | 0.007 | 1.96E-22 | 0.010 | 0.011 | 0.373 |
|  | rs2820426 | G | A | 0.052 | 0.007 | 1.30E-12 | -0.009 | 0.011 | 0.436 |
|  | rs2867125 | T | C | -0.060 | 0.010 | 4.33E-10 | -0.012 | 0.015 | 0.451 |
|  | rs2908282 | G | A | -0.055 | 0.009 | 4.25E-09 | 0.000 | 0.014 | 0.956 |
|  | rs2925979 | T | C | 0.053 | 0.008 | 9.06E-12 | 0.005 | 0.012 | 0.719 |
|  | rs2943656 | A | G | -0.090 | 0.007 | 6.70E-34 | 0.005 | 0.011 | 0.733 |
|  | rs3217992 | T | C | 0.053 | 0.007 | 7.23E-13 | 0.001 | 0.011 | 0.902 |
|  | rs340874 | T | C | -0.063 | 0.007 | 8.41E-18 | -0.010 | 0.011 | 0.383 |
|  | rs348330 | G | A | 0.049 | 0.008 | 1.86E-09 | -0.004 | 0.011 | 0.761 |
|  | rs3756784 | G | T | 0.051 | 0.009 | 2.59E-08 | -0.011 | 0.013 | 0.458 |
|  | rs3802177 | G | A | 0.122 | 0.008 | 2.32E-52 | -0.004 | 0.012 | 0.771 |
|  | rs459193 | G | A | 0.071 | 0.008 | 8.81E-18 | -0.006 | 0.013 | 0.659 |
|  | rs4622883 | A | G | 0.044 | 0.008 | 3.02E-08 | 0.003 | 0.011 | 0.826 |
|  | rs4686471 | C | T | 0.053 | 0.008 | 4.28E-11 | -0.010 | 0.011 | 0.453 |
|  | rs4812829 | A | G | 0.053 | 0.010 | 2.44E-08 | -0.008 | 0.014 | 0.517 |
|  | rs4823182 | A | G | -0.048 | 0.008 | 3.36E-10 | 0.000 | 0.011 | 0.966 |
|  | rs4865796 | A | G | 0.053 | 0.008 | 1.33E-11 | -0.014 | 0.012 | 0.283 |
|  | rs516946 | T | C | -0.082 | 0.009 | 3.16E-22 | 0.022 | 0.013 | 0.111 |
|  | rs5215 | C | T | 0.068 | 0.007 | 2.09E-20 | -0.019 | 0.011 | 0.099 |
|  | rs576674 | A | G | -0.065 | 0.010 | 1.79E-11 | -0.002 | 0.015 | 0.917 |
|  | rs6059662 | A | G | -0.045 | 0.008 | 1.51E-08 | 0.013 | 0.012 | 0.254 |
|  | rs61953351 | G | T | 0.070 | 0.009 | 1.98E-14 | 0.007 | 0.013 | 0.610 |
|  | rs622217 | C | T | -0.049 | 0.008 | 3.13E-10 | -0.021 | 0.011 | 0.056 |
|  | rs6515236 | A | C | 0.050 | 0.009 | 3.34E-08 | 0.008 | 0.012 | 0.555 |
|  | rs67232546 | C | T | -0.060 | 0.010 | 4.66E-10 | -0.023 | 0.014 | 0.111 |
|  | rs6767484 | A | G | -0.121 | 0.008 | 2.70E-56 | 0.004 | 0.012 | 0.730 |
|  | rs6785040 | C | T | -0.063 | 0.011 | 1.26E-08 | 0.023 | 0.015 | 0.146 |
|  | rs6795735 | C | T | 0.056 | 0.007 | 1.63E-14 | 0.009 | 0.011 | 0.398 |
|  | rs6878122 | A | G | -0.056 | 0.008 | 1.19E-12 | 0.018 | 0.012 | 0.105 |
|  | rs6960043 | C | T | 0.064 | 0.007 | 3.61E-19 | -0.006 | 0.011 | 0.586 |
|  | rs7144011 | G | T | -0.048 | 0.009 | 1.64E-08 | -0.020 | 0.014 | 0.129 |
|  | rs7177055 | A | G | 0.065 | 0.008 | 2.75E-16 | 0.009 | 0.012 | 0.434 |
|  | rs7240767 | C | T | 0.045 | 0.008 | 2.16E-08 | 0.001 | 0.011 | 0.935 |
|  | rs72892910 | G | T | -0.065 | 0.010 | 6.43E-11 | 0.001 | 0.014 | 0.934 |
|  | rs735949 | C | T | -0.071 | 0.011 | 1.95E-11 | -0.006 | 0.016 | 0.668 |
|  | rs753270 | C | T | 0.053 | 0.008 | 2.70E-11 | 0.021 | 0.011 | 0.063 |
|  | rs7561798 | A | G | -0.040 | 0.007 | 2.79E-08 | 0.001 | 0.011 | 0.949 |
|  | rs7572970 | A | G | -0.059 | 0.009 | 1.39E-11 | -0.005 | 0.012 | 0.701 |
|  | rs7607777 | G | T | 0.137 | 0.013 | 9.40E-28 | 0.006 | 0.018 | 0.788 |
|  | rs7674212 | G | T | 0.047 | 0.008 | 6.18E-10 | -0.002 | 0.011 | 0.843 |
|  | rs7685296 | C | T | 0.051 | 0.008 | 2.32E-10 | -0.017 | 0.012 | 0.180 |
|  | rs7729395 | C | T | -0.137 | 0.016 | 1.10E-17 | -0.020 | 0.057 | 0.755 |
|  | rs7756992 | A | G | -0.130 | 0.008 | 6.00E-62 | 0.013 | 0.012 | 0.263 |
|  | rs7786095 | A | G | 0.074 | 0.013 | 9.64E-09 | 0.010 | 0.019 | 0.600 |
|  | rs780094 | T | C | -0.069 | 0.007 | 5.16E-21 | 0.005 | 0.011 | 0.644 |
|  | rs7845219 | C | T | -0.042 | 0.007 | 4.54E-09 | -0.024 | 0.011 | 0.024 |
|  | rs7903146 | C | T | -0.306 | 0.008 | 1.00E-200 | 0.004 | 0.012 | 0.758 |
|  | rs7929543 | A | C | -0.083 | 0.014 | 2.20E-09 | -0.007 | 0.019 | 0.704 |
|  | rs7955901 | C | T | 0.044 | 0.007 | 7.16E-10 | -0.017 | 0.011 | 0.115 |
|  | rs8068804 | A | G | 0.059 | 0.008 | 4.41E-14 | -0.023 | 0.012 | 0.053 |
|  | rs8108269 | G | T | 0.064 | 0.008 | 3.11E-16 | -0.018 | 0.012 | 0.123 |
|  | rs825476 | C | T | -0.052 | 0.007 | 6.80E-13 | 0.006 | 0.011 | 0.588 |
|  | rs840967 | A | C | -0.050 | 0.008 | 5.44E-10 | 0.012 | 0.011 | 0.316 |
|  | rs849135 | A | G | -0.100 | 0.007 | 1.04E-43 | -0.006 | 0.011 | 0.558 |
|  | rs853974 | C | T | -0.060 | 0.009 | 7.86E-12 | -0.008 | 0.012 | 0.456 |
|  | rs9369425 | A | G | -0.055 | 0.009 | 1.13E-10 | 0.015 | 0.012 | 0.157 |
|  | rs9894220 | A | G | 0.059 | 0.008 | 1.52E-13 | -0.010 | 0.011 | 0.360 |
|  | rs9928094 | A | G | -0.105 | 0.007 | 3.59E-47 | -0.015 | 0.011 | 0.167 |
|  | rs993380 | A | G | 0.051 | 0.008 | 4.59E-10 | 0.004 | 0.011 | 0.687 |
|  | rs9940149 | A | G | -0.058 | 0.010 | 9.29E-10 | -0.012 | 0.014 | 0.396 |
| *Clostridiaceae_1* | rs10077431 | A | C | -0.049 | 0.009 | 4.75E-08 | -0.009 | 0.015 | 0.607 |
|  | rs10087241 | A | G | -0.048 | 0.008 | 2.80E-09 | -0.006 | 0.012 | 0.594 |
|  | rs10100265 | A | C | 0.049 | 0.008 | 6.29E-10 | -0.032 | 0.012 | 0.007 |
|  | rs10114341 | C | T | -0.041 | 0.007 | 1.15E-08 | 0.016 | 0.012 | 0.196 |
|  | rs10401969 | C | T | 0.092 | 0.013 | 4.13E-12 | 0.044 | 0.022 | 0.042 |
|  | rs1050226 | G | A | -0.049 | 0.007 | 3.34E-11 | 0.010 | 0.012 | 0.387 |
|  | rs1061813 | A | G | -0.043 | 0.007 | 3.37E-09 | 0.016 | 0.012 | 0.225 |
|  | rs1063355 | T | G | -0.071 | 0.008 | 3.72E-19 | -0.005 | 0.012 | 0.702 |
|  | rs10740322 | A | G | 0.048 | 0.009 | 2.11E-08 | 0.013 | 0.013 | 0.301 |
|  | rs10811661 | C | T | -0.157 | 0.010 | 4.13E-58 | 0.012 | 0.016 | 0.557 |
|  | rs10842994 | C | T | 0.076 | 0.009 | 1.02E-16 | 0.000 | 0.015 | 0.995 |
|  | rs10974438 | A | C | -0.059 | 0.008 | 3.01E-15 | 0.003 | 0.012 | 0.809 |
|  | rs11098676 | C | T | 0.054 | 0.010 | 2.03E-08 | -0.013 | 0.014 | 0.306 |
|  | rs11107116 | G | T | -0.047 | 0.009 | 3.75E-08 | 0.009 | 0.014 | 0.476 |
|  | rs1111875 | T | C | -0.095 | 0.007 | 3.61E-39 | -0.001 | 0.012 | 0.950 |
|  | rs11257655 | C | T | -0.074 | 0.009 | 1.97E-17 | -0.012 | 0.014 | 0.410 |
|  | rs1127655 | C | T | 0.044 | 0.008 | 2.47E-08 | -0.008 | 0.012 | 0.480 |
|  | rs11708067 | A | G | 0.097 | 0.009 | 5.93E-29 | -0.012 | 0.014 | 0.334 |
|  | rs11925227 | A | G | -0.053 | 0.010 | 2.25E-08 | 0.022 | 0.016 | 0.191 |
|  | rs11926707 | C | T | 0.046 | 0.008 | 1.69E-08 | 0.004 | 0.012 | 0.760 |
|  | rs12088739 | A | G | 0.088 | 0.013 | 9.79E-12 | 0.017 | 0.021 | 0.397 |
|  | rs12299509 | A | G | -0.047 | 0.007 | 2.09E-10 | 0.003 | 0.012 | 0.833 |
|  | rs12617659 | C | T | 0.069 | 0.010 | 2.83E-11 | 0.003 | 0.017 | 0.851 |
|  | rs12910825 | A | G | -0.052 | 0.007 | 2.16E-12 | 0.015 | 0.012 | 0.218 |
|  | rs12945601 | C | T | -0.048 | 0.008 | 1.72E-09 | -0.016 | 0.012 | 0.196 |
|  | rs12970134 | A | G | 0.056 | 0.008 | 5.31E-12 | 0.026 | 0.014 | 0.054 |
|  | rs13239186 | C | T | -0.054 | 0.009 | 2.70E-10 | 0.007 | 0.013 | 0.537 |
|  | rs13330951 | A | G | 0.046 | 0.008 | 1.54E-08 | 0.006 | 0.012 | 0.607 |
|  | rs13389219 | C | T | 0.072 | 0.007 | 2.11E-22 | -0.004 | 0.012 | 0.762 |
|  | rs1359790 | G | A | 0.080 | 0.008 | 2.80E-23 | 0.017 | 0.013 | 0.195 |
|  | rs1496653 | G | A | -0.077 | 0.009 | 2.57E-18 | -0.014 | 0.015 | 0.358 |
|  | rs1552224 | C | A | -0.103 | 0.010 | 8.64E-25 | 0.021 | 0.016 | 0.196 |
|  | rs16988333 | A | G | 0.075 | 0.013 | 9.17E-09 | 0.006 | 0.023 | 0.802 |
|  | rs17086692 | G | T | 0.047 | 0.008 | 2.48E-08 | -0.006 | 0.013 | 0.615 |
|  | rs17168486 | C | T | -0.074 | 0.009 | 2.18E-15 | 0.011 | 0.015 | 0.427 |
|  | rs17405722 | A | G | 0.087 | 0.015 | 2.28E-09 | -0.021 | 0.024 | 0.374 |
|  | rs17631783 | C | T | 0.049 | 0.009 | 3.95E-08 | -0.024 | 0.014 | 0.113 |
|  | rs17791513 | A | G | 0.103 | 0.015 | 4.61E-12 | -0.061 | 0.024 | 0.015 |
|  | rs1801214 | C | T | 0.090 | 0.007 | 5.52E-34 | 0.002 | 0.012 | 0.945 |
|  | rs1899951 | T | C | -0.112 | 0.011 | 1.64E-24 | -0.029 | 0.018 | 0.125 |
|  | rs2237892 | C | T | 0.096 | 0.016 | 8.75E-10 | 0.010 | 0.024 | 0.578 |
|  | rs2246618 | C | T | -0.051 | 0.008 | 1.20E-09 | 0.022 | 0.013 | 0.095 |
|  | rs2261181 | C | T | -0.099 | 0.012 | 9.18E-17 | -0.010 | 0.020 | 0.728 |
|  | rs2294120 | G | A | -0.044 | 0.008 | 1.62E-08 | -0.013 | 0.012 | 0.268 |
|  | rs2296173 | A | G | -0.065 | 0.009 | 7.66E-14 | -0.035 | 0.015 | 0.016 |
|  | rs2299383 | C | T | -0.041 | 0.007 | 1.49E-08 | -0.010 | 0.012 | 0.408 |
|  | rs243019 | T | C | -0.057 | 0.007 | 2.29E-15 | -0.029 | 0.012 | 0.016 |
|  | rs2493394 | G | A | 0.073 | 0.011 | 1.15E-10 | -0.002 | 0.019 | 0.970 |
|  | rs2796441 | G | A | 0.072 | 0.007 | 1.96E-22 | -0.006 | 0.012 | 0.607 |
|  | rs2820426 | G | A | 0.052 | 0.007 | 1.30E-12 | 0.016 | 0.012 | 0.179 |
|  | rs2867125 | T | C | -0.060 | 0.010 | 4.33E-10 | 0.003 | 0.016 | 0.876 |
|  | rs2908282 | G | A | -0.055 | 0.009 | 4.25E-09 | -0.033 | 0.016 | 0.034 |
|  | rs2925979 | T | C | 0.053 | 0.008 | 9.06E-12 | 0.022 | 0.013 | 0.099 |
|  | rs2943656 | A | G | -0.090 | 0.007 | 6.70E-34 | -0.025 | 0.013 | 0.050 |
|  | rs3217992 | T | C | 0.053 | 0.007 | 7.23E-13 | 0.005 | 0.012 | 0.669 |
|  | rs340874 | T | C | -0.063 | 0.007 | 8.41E-18 | 0.005 | 0.012 | 0.670 |
|  | rs348330 | G | A | 0.049 | 0.008 | 1.86E-09 | -0.012 | 0.013 | 0.312 |
|  | rs3756784 | G | T | 0.051 | 0.009 | 2.59E-08 | -0.004 | 0.015 | 0.705 |
|  | rs3802177 | G | A | 0.122 | 0.008 | 2.32E-52 | 0.009 | 0.013 | 0.506 |
|  | rs459193 | G | A | 0.071 | 0.008 | 8.81E-18 | 0.016 | 0.014 | 0.210 |
|  | rs4622883 | A | G | 0.044 | 0.008 | 3.02E-08 | 0.003 | 0.012 | 0.775 |
|  | rs4686471 | C | T | 0.053 | 0.008 | 4.28E-11 | -0.004 | 0.012 | 0.643 |
|  | rs4812829 | A | G | 0.053 | 0.010 | 2.44E-08 | 0.008 | 0.015 | 0.617 |
|  | rs4823182 | A | G | -0.048 | 0.008 | 3.36E-10 | 0.007 | 0.012 | 0.589 |
|  | rs4865796 | A | G | 0.053 | 0.008 | 1.33E-11 | -0.013 | 0.013 | 0.278 |
|  | rs516946 | T | C | -0.082 | 0.009 | 3.16E-22 | -0.008 | 0.014 | 0.556 |
|  | rs5215 | C | T | 0.068 | 0.007 | 2.09E-20 | -0.004 | 0.012 | 0.745 |
|  | rs576674 | A | G | -0.065 | 0.010 | 1.79E-11 | -0.036 | 0.016 | 0.018 |
|  | rs6059662 | A | G | -0.045 | 0.008 | 1.51E-08 | 0.008 | 0.013 | 0.523 |
|  | rs61953351 | G | T | 0.070 | 0.009 | 1.98E-14 | 0.001 | 0.014 | 0.893 |
|  | rs622217 | C | T | -0.049 | 0.008 | 3.13E-10 | -0.001 | 0.012 | 0.928 |
|  | rs6515236 | A | C | 0.050 | 0.009 | 3.34E-08 | -0.002 | 0.014 | 0.862 |
|  | rs67232546 | C | T | -0.060 | 0.010 | 4.66E-10 | -0.005 | 0.015 | 0.731 |
|  | rs6767484 | A | G | -0.121 | 0.008 | 2.70E-56 | 0.035 | 0.013 | 0.007 |
|  | rs6785040 | C | T | -0.063 | 0.011 | 1.26E-08 | 0.022 | 0.016 | 0.148 |
|  | rs6795735 | C | T | 0.056 | 0.007 | 1.63E-14 | -0.006 | 0.012 | 0.634 |
|  | rs6878122 | A | G | -0.056 | 0.008 | 1.19E-12 | 0.004 | 0.013 | 0.758 |
|  | rs6960043 | C | T | 0.064 | 0.007 | 3.61E-19 | 0.019 | 0.012 | 0.120 |
|  | rs7144011 | G | T | -0.048 | 0.009 | 1.64E-08 | -0.002 | 0.015 | 0.877 |
|  | rs7177055 | A | G | 0.065 | 0.008 | 2.75E-16 | 0.000 | 0.013 | 0.978 |
|  | rs7240767 | C | T | 0.045 | 0.008 | 2.16E-08 | -0.009 | 0.012 | 0.467 |
|  | rs72892910 | G | T | -0.065 | 0.010 | 6.43E-11 | 0.014 | 0.016 | 0.387 |
|  | rs735949 | C | T | -0.071 | 0.011 | 1.95E-11 | -0.021 | 0.018 | 0.238 |
|  | rs753270 | C | T | 0.053 | 0.008 | 2.70E-11 | 0.019 | 0.012 | 0.118 |
|  | rs7561798 | A | G | -0.040 | 0.007 | 2.79E-08 | 0.001 | 0.012 | 0.923 |
|  | rs7572970 | A | G | -0.059 | 0.009 | 1.39E-11 | 0.003 | 0.013 | 0.878 |
|  | rs7607777 | G | T | 0.137 | 0.013 | 9.40E-28 | 0.015 | 0.020 | 0.425 |
|  | rs7674212 | G | T | 0.047 | 0.008 | 6.18E-10 | 0.005 | 0.012 | 0.657 |
|  | rs7685296 | C | T | 0.051 | 0.008 | 2.32E-10 | -0.005 | 0.013 | 0.711 |
|  | rs7756992 | A | G | -0.130 | 0.008 | 6.00E-62 | 0.013 | 0.013 | 0.304 |
|  | rs7786095 | A | G | 0.074 | 0.013 | 9.64E-09 | -0.015 | 0.020 | 0.523 |
|  | rs780094 | T | C | -0.069 | 0.007 | 5.16E-21 | -0.013 | 0.012 | 0.262 |
|  | rs7845219 | C | T | -0.042 | 0.007 | 4.54E-09 | 0.032 | 0.012 | 0.008 |
|  | rs7903146 | C | T | -0.306 | 0.008 | 1.00E-200 | -0.004 | 0.013 | 0.757 |
|  | rs7929543 | A | C | -0.083 | 0.014 | 2.20E-09 | -0.017 | 0.021 | 0.426 |
|  | rs7955901 | C | T | 0.044 | 0.007 | 7.16E-10 | -0.021 | 0.012 | 0.082 |
|  | rs8068804 | A | G | 0.059 | 0.008 | 4.41E-14 | 0.002 | 0.013 | 0.839 |
|  | rs8108269 | G | T | 0.064 | 0.008 | 3.11E-16 | -0.001 | 0.013 | 0.889 |
|  | rs825476 | C | T | -0.052 | 0.007 | 6.80E-13 | 0.018 | 0.012 | 0.130 |
|  | rs840967 | A | C | -0.050 | 0.008 | 5.44E-10 | 0.006 | 0.012 | 0.605 |
|  | rs849135 | A | G | -0.100 | 0.007 | 1.04E-43 | -0.005 | 0.012 | 0.651 |
|  | rs853974 | C | T | -0.060 | 0.009 | 7.86E-12 | 0.015 | 0.013 | 0.268 |
|  | rs9369425 | A | G | -0.055 | 0.009 | 1.13E-10 | -0.005 | 0.013 | 0.733 |
|  | rs9894220 | A | G | 0.059 | 0.008 | 1.52E-13 | -0.004 | 0.012 | 0.760 |
|  | rs9928094 | A | G | -0.105 | 0.007 | 3.59E-47 | 0.002 | 0.012 | 0.838 |
|  | rs993380 | A | G | 0.051 | 0.008 | 4.59E-10 | 0.005 | 0.012 | 0.707 |
|  | rs9940149 | A | G | -0.058 | 0.010 | 9.29E-10 | -0.002 | 0.015 | 0.867 |
| *Coriobacteriaceae* | rs10077431 | A | C | -0.049 | 0.009 | 4.75E-08 | 0.004 | 0.013 | 0.866 |
|  | rs10087241 | A | G | -0.048 | 0.008 | 2.80E-09 | -0.024 | 0.011 | 0.039 |
|  | rs10100265 | A | C | 0.049 | 0.008 | 6.29E-10 | 0.028 | 0.011 | 0.010 |
|  | rs10114341 | C | T | -0.041 | 0.007 | 1.15E-08 | -0.006 | 0.011 | 0.591 |
|  | rs10401969 | C | T | 0.092 | 0.013 | 4.13E-12 | 0.027 | 0.020 | 0.212 |
|  | rs1050226 | G | A | -0.049 | 0.007 | 3.34E-11 | 0.010 | 0.011 | 0.372 |
|  | rs1061813 | A | G | -0.043 | 0.007 | 3.37E-09 | -0.007 | 0.011 | 0.544 |
|  | rs1063355 | T | G | -0.071 | 0.008 | 3.72E-19 | 0.001 | 0.011 | 0.948 |
|  | rs10740322 | A | G | 0.048 | 0.009 | 2.11E-08 | 0.018 | 0.011 | 0.101 |
|  | rs10811661 | C | T | -0.157 | 0.010 | 4.13E-58 | 0.004 | 0.014 | 0.804 |
|  | rs10842994 | C | T | 0.076 | 0.009 | 1.02E-16 | -0.006 | 0.013 | 0.632 |
|  | rs10974438 | A | C | -0.059 | 0.008 | 3.01E-15 | -0.003 | 0.011 | 0.798 |
|  | rs11098676 | C | T | 0.054 | 0.010 | 2.03E-08 | -0.007 | 0.013 | 0.622 |
|  | rs11107116 | G | T | -0.047 | 0.009 | 3.75E-08 | 0.019 | 0.013 | 0.162 |
|  | rs1111875 | T | C | -0.095 | 0.007 | 3.61E-39 | 0.011 | 0.011 | 0.336 |
|  | rs11257655 | C | T | -0.074 | 0.009 | 1.97E-17 | 0.012 | 0.013 | 0.302 |
|  | rs1127655 | C | T | 0.044 | 0.008 | 2.47E-08 | -0.003 | 0.011 | 0.762 |
|  | rs11708067 | A | G | 0.097 | 0.009 | 5.93E-29 | 0.012 | 0.013 | 0.388 |
|  | rs11925227 | A | G | -0.053 | 0.010 | 2.25E-08 | 0.033 | 0.014 | 0.016 |
|  | rs11926707 | C | T | 0.046 | 0.008 | 1.69E-08 | -0.015 | 0.011 | 0.163 |
|  | rs12088739 | A | G | 0.088 | 0.013 | 9.79E-12 | 0.017 | 0.019 | 0.334 |
|  | rs12299509 | A | G | -0.047 | 0.007 | 2.09E-10 | -0.003 | 0.011 | 0.730 |
|  | rs12617659 | C | T | 0.069 | 0.010 | 2.83E-11 | 0.008 | 0.015 | 0.670 |
|  | rs12910825 | A | G | -0.052 | 0.007 | 2.16E-12 | 0.011 | 0.011 | 0.282 |
|  | rs12945601 | C | T | -0.048 | 0.008 | 1.72E-09 | -0.013 | 0.011 | 0.229 |
|  | rs12970134 | A | G | 0.056 | 0.008 | 5.31E-12 | 0.000 | 0.012 | 0.992 |
|  | rs13239186 | C | T | -0.054 | 0.009 | 2.70E-10 | 0.004 | 0.012 | 0.723 |
|  | rs13330951 | A | G | 0.046 | 0.008 | 1.54E-08 | -0.008 | 0.011 | 0.510 |
|  | rs13389219 | C | T | 0.072 | 0.007 | 2.11E-22 | 0.011 | 0.011 | 0.397 |
|  | rs1359790 | G | A | 0.080 | 0.008 | 2.80E-23 | -0.014 | 0.012 | 0.272 |
|  | rs1496653 | G | A | -0.077 | 0.009 | 2.57E-18 | -0.013 | 0.013 | 0.315 |
|  | rs1552224 | C | A | -0.103 | 0.010 | 8.64E-25 | -0.002 | 0.015 | 0.903 |
|  | rs16988333 | A | G | 0.075 | 0.013 | 9.17E-09 | 0.033 | 0.021 | 0.122 |
|  | rs17086692 | G | T | 0.047 | 0.008 | 2.48E-08 | 0.003 | 0.011 | 0.817 |
|  | rs17168486 | C | T | -0.074 | 0.009 | 2.18E-15 | -0.002 | 0.014 | 0.974 |
|  | rs17405722 | A | G | 0.087 | 0.015 | 2.28E-09 | -0.005 | 0.021 | 0.775 |
|  | rs17631783 | C | T | 0.049 | 0.009 | 3.95E-08 | -0.036 | 0.012 | 0.002 |
|  | rs17791513 | A | G | 0.103 | 0.015 | 4.61E-12 | -0.057 | 0.021 | 0.012 |
|  | rs1801214 | C | T | 0.090 | 0.007 | 5.52E-34 | 0.013 | 0.011 | 0.197 |
|  | rs1899951 | T | C | -0.112 | 0.011 | 1.64E-24 | -0.010 | 0.016 | 0.560 |
|  | rs2237892 | C | T | 0.096 | 0.016 | 8.75E-10 | 0.039 | 0.021 | 0.031 |
|  | rs2246618 | C | T | -0.051 | 0.008 | 1.20E-09 | -0.008 | 0.011 | 0.474 |
|  | rs2261181 | C | T | -0.099 | 0.012 | 9.18E-17 | 0.018 | 0.018 | 0.419 |
|  | rs2294120 | G | A | -0.044 | 0.008 | 1.62E-08 | -0.002 | 0.011 | 0.854 |
|  | rs2296173 | A | G | -0.065 | 0.009 | 7.66E-14 | -0.019 | 0.013 | 0.158 |
|  | rs2299383 | C | T | -0.041 | 0.007 | 1.49E-08 | 0.016 | 0.011 | 0.137 |
|  | rs243019 | T | C | -0.057 | 0.007 | 2.29E-15 | -0.014 | 0.011 | 0.207 |
|  | rs2493394 | G | A | 0.073 | 0.011 | 1.15E-10 | 0.000 | 0.017 | 0.979 |
|  | rs2796441 | G | A | 0.072 | 0.007 | 1.96E-22 | -0.007 | 0.011 | 0.502 |
|  | rs2820426 | G | A | 0.052 | 0.007 | 1.30E-12 | -0.002 | 0.011 | 0.862 |
|  | rs2867125 | T | C | -0.060 | 0.010 | 4.33E-10 | -0.005 | 0.014 | 0.807 |
|  | rs2908282 | G | A | -0.055 | 0.009 | 4.25E-09 | 0.001 | 0.014 | 0.951 |
|  | rs2925979 | T | C | 0.053 | 0.008 | 9.06E-12 | 0.000 | 0.012 | 0.969 |
|  | rs2943656 | A | G | -0.090 | 0.007 | 6.70E-34 | 0.009 | 0.011 | 0.452 |
|  | rs3217992 | T | C | 0.053 | 0.007 | 7.23E-13 | 0.007 | 0.011 | 0.567 |
|  | rs340874 | T | C | -0.063 | 0.007 | 8.41E-18 | 0.007 | 0.011 | 0.538 |
|  | rs348330 | G | A | 0.049 | 0.008 | 1.86E-09 | -0.014 | 0.011 | 0.210 |
|  | rs3756784 | G | T | 0.051 | 0.009 | 2.59E-08 | 0.001 | 0.013 | 0.924 |
|  | rs3802177 | G | A | 0.122 | 0.008 | 2.32E-52 | -0.009 | 0.012 | 0.442 |
|  | rs459193 | G | A | 0.071 | 0.008 | 8.81E-18 | -0.010 | 0.012 | 0.439 |
|  | rs4622883 | A | G | 0.044 | 0.008 | 3.02E-08 | -0.016 | 0.011 | 0.122 |
|  | rs4686471 | C | T | 0.053 | 0.008 | 4.28E-11 | -0.006 | 0.011 | 0.547 |
|  | rs4812829 | A | G | 0.053 | 0.010 | 2.44E-08 | -0.007 | 0.014 | 0.580 |
|  | rs4823182 | A | G | -0.048 | 0.008 | 3.36E-10 | -0.005 | 0.011 | 0.666 |
|  | rs4865796 | A | G | 0.053 | 0.008 | 1.33E-11 | -0.013 | 0.012 | 0.299 |
|  | rs516946 | T | C | -0.082 | 0.009 | 3.16E-22 | 0.022 | 0.013 | 0.084 |
|  | rs5215 | C | T | 0.068 | 0.007 | 2.09E-20 | -0.004 | 0.011 | 0.725 |
|  | rs576674 | A | G | -0.065 | 0.010 | 1.79E-11 | -0.006 | 0.014 | 0.670 |
|  | rs6059662 | A | G | -0.045 | 0.008 | 1.51E-08 | -0.010 | 0.012 | 0.423 |
|  | rs61953351 | G | T | 0.070 | 0.009 | 1.98E-14 | 0.013 | 0.012 | 0.267 |
|  | rs622217 | C | T | -0.049 | 0.008 | 3.13E-10 | 0.001 | 0.011 | 0.956 |
|  | rs6515236 | A | C | 0.050 | 0.009 | 3.34E-08 | -0.007 | 0.012 | 0.549 |
|  | rs67232546 | C | T | -0.060 | 0.010 | 4.66E-10 | 0.004 | 0.013 | 0.780 |
|  | rs6767484 | A | G | -0.121 | 0.008 | 2.70E-56 | 0.004 | 0.012 | 0.742 |
|  | rs6785040 | C | T | -0.063 | 0.011 | 1.26E-08 | 0.024 | 0.014 | 0.088 |
|  | rs6795735 | C | T | 0.056 | 0.007 | 1.63E-14 | 0.002 | 0.011 | 0.879 |
|  | rs6878122 | A | G | -0.056 | 0.008 | 1.19E-12 | -0.001 | 0.012 | 0.964 |
|  | rs6960043 | C | T | 0.064 | 0.007 | 3.61E-19 | -0.003 | 0.011 | 0.796 |
|  | rs7144011 | G | T | -0.048 | 0.009 | 1.64E-08 | -0.019 | 0.013 | 0.165 |
|  | rs7177055 | A | G | 0.065 | 0.008 | 2.75E-16 | 0.004 | 0.012 | 0.775 |
|  | rs7240767 | C | T | 0.045 | 0.008 | 2.16E-08 | -0.005 | 0.011 | 0.613 |
|  | rs72892910 | G | T | -0.065 | 0.010 | 6.43E-11 | -0.026 | 0.014 | 0.067 |
|  | rs735949 | C | T | -0.071 | 0.011 | 1.95E-11 | -0.015 | 0.016 | 0.344 |
|  | rs753270 | C | T | 0.053 | 0.008 | 2.70E-11 | 0.011 | 0.011 | 0.298 |
|  | rs7561798 | A | G | -0.040 | 0.007 | 2.79E-08 | 0.002 | 0.011 | 0.876 |
|  | rs7572970 | A | G | -0.059 | 0.009 | 1.39E-11 | -0.017 | 0.012 | 0.177 |
|  | rs7607777 | G | T | 0.137 | 0.013 | 9.40E-28 | -0.041 | 0.018 | 0.030 |
|  | rs7674212 | G | T | 0.047 | 0.008 | 6.18E-10 | 0.001 | 0.011 | 0.919 |
|  | rs7685296 | C | T | 0.051 | 0.008 | 2.32E-10 | -0.008 | 0.012 | 0.448 |
|  | rs7729395 | C | T | -0.137 | 0.016 | 1.10E-17 | -0.035 | 0.056 | 0.517 |
|  | rs7756992 | A | G | -0.130 | 0.008 | 6.00E-62 | 0.004 | 0.012 | 0.731 |
|  | rs7786095 | A | G | 0.074 | 0.013 | 9.64E-09 | 0.015 | 0.018 | 0.414 |
|  | rs780094 | T | C | -0.069 | 0.007 | 5.16E-21 | 0.002 | 0.011 | 0.868 |
|  | rs7845219 | C | T | -0.042 | 0.007 | 4.54E-09 | -0.001 | 0.011 | 0.943 |
|  | rs7903146 | C | T | -0.306 | 0.008 | 1.00E-200 | -0.020 | 0.012 | 0.099 |
|  | rs7929543 | A | C | -0.083 | 0.014 | 2.20E-09 | -0.010 | 0.018 | 0.727 |
|  | rs7955901 | C | T | 0.044 | 0.007 | 7.16E-10 | 0.001 | 0.011 | 0.914 |
|  | rs8068804 | A | G | 0.059 | 0.008 | 4.41E-14 | 0.006 | 0.012 | 0.620 |
|  | rs8108269 | G | T | 0.064 | 0.008 | 3.11E-16 | -0.007 | 0.012 | 0.595 |
|  | rs825476 | C | T | -0.052 | 0.007 | 6.80E-13 | 0.007 | 0.011 | 0.510 |
|  | rs840967 | A | C | -0.050 | 0.008 | 5.44E-10 | -0.017 | 0.011 | 0.109 |
|  | rs849135 | A | G | -0.100 | 0.007 | 1.04E-43 | 0.007 | 0.011 | 0.515 |
|  | rs853974 | C | T | -0.060 | 0.009 | 7.86E-12 | -0.007 | 0.012 | 0.613 |
|  | rs9369425 | A | G | -0.055 | 0.009 | 1.13E-10 | -0.003 | 0.012 | 0.791 |
|  | rs9894220 | A | G | 0.059 | 0.008 | 1.52E-13 | 0.006 | 0.011 | 0.566 |
|  | rs9928094 | A | G | -0.105 | 0.007 | 3.59E-47 | -0.022 | 0.011 | 0.042 |
|  | rs993380 | A | G | 0.051 | 0.008 | 4.59E-10 | 0.026 | 0.011 | 0.020 |
|  | rs9940149 | A | G | -0.058 | 0.010 | 9.29E-10 | 0.020 | 0.014 | 0.123 |
| *Defluviitaleaceae* | rs10077431 | A | C | -0.049 | 0.009 | 4.75E-08 | 0.017 | 0.019 | 0.442 |
|  | rs10087241 | A | G | -0.048 | 0.008 | 2.80E-09 | 0.009 | 0.016 | 0.582 |
|  | rs10100265 | A | C | 0.049 | 0.008 | 6.29E-10 | -0.013 | 0.016 | 0.422 |
|  | rs10114341 | C | T | -0.041 | 0.007 | 1.15E-08 | 0.002 | 0.016 | 0.879 |
|  | rs10401969 | C | T | 0.092 | 0.013 | 4.13E-12 | -0.017 | 0.030 | 0.504 |
|  | rs1050226 | G | A | -0.049 | 0.007 | 3.34E-11 | 0.033 | 0.016 | 0.039 |
|  | rs1061813 | A | G | -0.043 | 0.007 | 3.37E-09 | -0.013 | 0.016 | 0.402 |
|  | rs1063355 | T | G | -0.071 | 0.008 | 3.72E-19 | -0.014 | 0.016 | 0.393 |
|  | rs10740322 | A | G | 0.048 | 0.009 | 2.11E-08 | -0.006 | 0.017 | 0.689 |
|  | rs10811661 | C | T | -0.157 | 0.010 | 4.13E-58 | 0.036 | 0.021 | 0.071 |
|  | rs10842994 | C | T | 0.076 | 0.009 | 1.02E-16 | -0.008 | 0.019 | 0.718 |
|  | rs10974438 | A | C | -0.059 | 0.008 | 3.01E-15 | -0.014 | 0.016 | 0.397 |
|  | rs11098676 | C | T | 0.054 | 0.010 | 2.03E-08 | 0.008 | 0.019 | 0.761 |
|  | rs11107116 | G | T | -0.047 | 0.009 | 3.75E-08 | 0.020 | 0.019 | 0.260 |
|  | rs1111875 | T | C | -0.095 | 0.007 | 3.61E-39 | 0.006 | 0.016 | 0.704 |
|  | rs11257655 | C | T | -0.074 | 0.009 | 1.97E-17 | 0.003 | 0.019 | 0.857 |
|  | rs1127655 | C | T | 0.044 | 0.008 | 2.47E-08 | -0.015 | 0.016 | 0.334 |
|  | rs11708067 | A | G | 0.097 | 0.009 | 5.93E-29 | -0.014 | 0.019 | 0.483 |
|  | rs11925227 | A | G | -0.053 | 0.010 | 2.25E-08 | 0.029 | 0.020 | 0.165 |
|  | rs11926707 | C | T | 0.046 | 0.008 | 1.69E-08 | -0.031 | 0.016 | 0.062 |
|  | rs12088739 | A | G | 0.088 | 0.013 | 9.79E-12 | 0.009 | 0.027 | 0.717 |
|  | rs12299509 | A | G | -0.047 | 0.007 | 2.09E-10 | 0.010 | 0.016 | 0.534 |
|  | rs12617659 | C | T | 0.069 | 0.010 | 2.83E-11 | 0.005 | 0.022 | 0.856 |
|  | rs12910825 | A | G | -0.052 | 0.007 | 2.16E-12 | -0.015 | 0.016 | 0.350 |
|  | rs12945601 | C | T | -0.048 | 0.008 | 1.72E-09 | -0.003 | 0.016 | 0.854 |
|  | rs12970134 | A | G | 0.056 | 0.008 | 5.31E-12 | 0.019 | 0.018 | 0.278 |
|  | rs13239186 | C | T | -0.054 | 0.009 | 2.70E-10 | -0.022 | 0.017 | 0.201 |
|  | rs13330951 | A | G | 0.046 | 0.008 | 1.54E-08 | 0.010 | 0.016 | 0.557 |
|  | rs13389219 | C | T | 0.072 | 0.007 | 2.11E-22 | -0.013 | 0.016 | 0.488 |
|  | rs1359790 | G | A | 0.080 | 0.008 | 2.80E-23 | -0.019 | 0.018 | 0.291 |
|  | rs1496653 | G | A | -0.077 | 0.009 | 2.57E-18 | 0.012 | 0.019 | 0.550 |
|  | rs1552224 | C | A | -0.103 | 0.010 | 8.64E-25 | -0.016 | 0.021 | 0.420 |
|  | rs16988333 | A | G | 0.075 | 0.013 | 9.17E-09 | -0.013 | 0.029 | 0.680 |
|  | rs17086692 | G | T | 0.047 | 0.008 | 2.48E-08 | 0.009 | 0.017 | 0.588 |
|  | rs17168486 | C | T | -0.074 | 0.009 | 2.18E-15 | -0.023 | 0.021 | 0.289 |
|  | rs17405722 | A | G | 0.087 | 0.015 | 2.28E-09 | -0.009 | 0.030 | 0.798 |
|  | rs17631783 | C | T | 0.049 | 0.009 | 3.95E-08 | -0.012 | 0.018 | 0.513 |
|  | rs17791513 | A | G | 0.103 | 0.015 | 4.61E-12 | 0.014 | 0.032 | 0.699 |
|  | rs1801214 | C | T | 0.090 | 0.007 | 5.52E-34 | 0.023 | 0.016 | 0.183 |
|  | rs1899951 | T | C | -0.112 | 0.011 | 1.64E-24 | 0.009 | 0.023 | 0.728 |
|  | rs2237892 | C | T | 0.096 | 0.016 | 8.75E-10 | 0.012 | 0.035 | 0.607 |
|  | rs2246618 | C | T | -0.051 | 0.008 | 1.20E-09 | -0.003 | 0.017 | 0.859 |
|  | rs2261181 | C | T | -0.099 | 0.012 | 9.18E-17 | -0.034 | 0.027 | 0.208 |
|  | rs2294120 | G | A | -0.044 | 0.008 | 1.62E-08 | 0.023 | 0.016 | 0.135 |
|  | rs2296173 | A | G | -0.065 | 0.009 | 7.66E-14 | 0.002 | 0.019 | 0.890 |
|  | rs2299383 | C | T | -0.041 | 0.007 | 1.49E-08 | 0.041 | 0.016 | 0.010 |
|  | rs243019 | T | C | -0.057 | 0.007 | 2.29E-15 | -0.004 | 0.016 | 0.797 |
|  | rs2493394 | G | A | 0.073 | 0.011 | 1.15E-10 | 0.053 | 0.025 | 0.036 |
|  | rs2796441 | G | A | 0.072 | 0.007 | 1.96E-22 | -0.004 | 0.016 | 0.790 |
|  | rs2820426 | G | A | 0.052 | 0.007 | 1.30E-12 | 0.014 | 0.016 | 0.403 |
|  | rs2867125 | T | C | -0.060 | 0.010 | 4.33E-10 | -0.010 | 0.021 | 0.678 |
|  | rs2908282 | G | A | -0.055 | 0.009 | 4.25E-09 | 0.008 | 0.021 | 0.704 |
|  | rs2925979 | T | C | 0.053 | 0.008 | 9.06E-12 | 0.000 | 0.017 | 0.994 |
|  | rs2943656 | A | G | -0.090 | 0.007 | 6.70E-34 | -0.026 | 0.016 | 0.103 |
|  | rs3217992 | T | C | 0.053 | 0.007 | 7.23E-13 | 0.008 | 0.016 | 0.593 |
|  | rs340874 | T | C | -0.063 | 0.007 | 8.41E-18 | -0.001 | 0.016 | 0.992 |
|  | rs348330 | G | A | 0.049 | 0.008 | 1.86E-09 | -0.019 | 0.017 | 0.251 |
|  | rs3756784 | G | T | 0.051 | 0.009 | 2.59E-08 | 0.003 | 0.019 | 0.846 |
|  | rs3802177 | G | A | 0.122 | 0.008 | 2.32E-52 | 0.007 | 0.017 | 0.624 |
|  | rs459193 | G | A | 0.071 | 0.008 | 8.81E-18 | -0.009 | 0.018 | 0.608 |
|  | rs4622883 | A | G | 0.044 | 0.008 | 3.02E-08 | -0.005 | 0.016 | 0.759 |
|  | rs4686471 | C | T | 0.053 | 0.008 | 4.28E-11 | -0.013 | 0.016 | 0.372 |
|  | rs4812829 | A | G | 0.053 | 0.010 | 2.44E-08 | -0.021 | 0.021 | 0.308 |
|  | rs4823182 | A | G | -0.048 | 0.008 | 3.36E-10 | 0.000 | 0.016 | 0.993 |
|  | rs4865796 | A | G | 0.053 | 0.008 | 1.33E-11 | -0.021 | 0.017 | 0.262 |
|  | rs516946 | T | C | -0.082 | 0.009 | 3.16E-22 | -0.005 | 0.018 | 0.797 |
|  | rs5215 | C | T | 0.068 | 0.007 | 2.09E-20 | 0.008 | 0.016 | 0.613 |
|  | rs576674 | A | G | -0.065 | 0.010 | 1.79E-11 | 0.064 | 0.021 | 0.002 |
|  | rs6059662 | A | G | -0.045 | 0.008 | 1.51E-08 | 0.010 | 0.017 | 0.596 |
|  | rs61953351 | G | T | 0.070 | 0.009 | 1.98E-14 | 0.006 | 0.018 | 0.791 |
|  | rs622217 | C | T | -0.049 | 0.008 | 3.13E-10 | -0.009 | 0.016 | 0.560 |
|  | rs6515236 | A | C | 0.050 | 0.009 | 3.34E-08 | 0.010 | 0.018 | 0.571 |
|  | rs67232546 | C | T | -0.060 | 0.010 | 4.66E-10 | -0.006 | 0.020 | 0.788 |
|  | rs6767484 | A | G | -0.121 | 0.008 | 2.70E-56 | 0.027 | 0.017 | 0.112 |
|  | rs6785040 | C | T | -0.063 | 0.011 | 1.26E-08 | 0.004 | 0.021 | 0.880 |
|  | rs6795735 | C | T | 0.056 | 0.007 | 1.63E-14 | 0.014 | 0.016 | 0.386 |
|  | rs6878122 | A | G | -0.056 | 0.008 | 1.19E-12 | 0.017 | 0.017 | 0.337 |
|  | rs6960043 | C | T | 0.064 | 0.007 | 3.61E-19 | -0.012 | 0.016 | 0.464 |
|  | rs7144011 | G | T | -0.048 | 0.009 | 1.64E-08 | -0.001 | 0.019 | 0.938 |
|  | rs7177055 | A | G | 0.065 | 0.008 | 2.75E-16 | 0.022 | 0.017 | 0.203 |
|  | rs7240767 | C | T | 0.045 | 0.008 | 2.16E-08 | 0.014 | 0.016 | 0.372 |
|  | rs72892910 | G | T | -0.065 | 0.010 | 6.43E-11 | 0.011 | 0.021 | 0.564 |
|  | rs735949 | C | T | -0.071 | 0.011 | 1.95E-11 | 0.012 | 0.023 | 0.625 |
|  | rs753270 | C | T | 0.053 | 0.008 | 2.70E-11 | 0.007 | 0.016 | 0.647 |
|  | rs7561798 | A | G | -0.040 | 0.007 | 2.79E-08 | -0.002 | 0.016 | 0.921 |
|  | rs7572970 | A | G | -0.059 | 0.009 | 1.39E-11 | 0.005 | 0.018 | 0.794 |
|  | rs7607777 | G | T | 0.137 | 0.013 | 9.40E-28 | 0.010 | 0.025 | 0.722 |
|  | rs7674212 | G | T | 0.047 | 0.008 | 6.18E-10 | 0.024 | 0.016 | 0.133 |
|  | rs7685296 | C | T | 0.051 | 0.008 | 2.32E-10 | 0.009 | 0.017 | 0.607 |
|  | rs7756992 | A | G | -0.130 | 0.008 | 6.00E-62 | -0.001 | 0.017 | 0.960 |
|  | rs7786095 | A | G | 0.074 | 0.013 | 9.64E-09 | 0.025 | 0.026 | 0.321 |
|  | rs780094 | T | C | -0.069 | 0.007 | 5.16E-21 | 0.009 | 0.016 | 0.599 |
|  | rs7845219 | C | T | -0.042 | 0.007 | 4.54E-09 | -0.017 | 0.016 | 0.297 |
|  | rs7903146 | C | T | -0.306 | 0.008 | 1.00E-200 | -0.011 | 0.018 | 0.547 |
|  | rs7929543 | A | C | -0.083 | 0.014 | 2.20E-09 | -0.047 | 0.028 | 0.102 |
|  | rs7955901 | C | T | 0.044 | 0.007 | 7.16E-10 | -0.012 | 0.016 | 0.434 |
|  | rs8068804 | A | G | 0.059 | 0.008 | 4.41E-14 | -0.007 | 0.017 | 0.675 |
|  | rs8108269 | G | T | 0.064 | 0.008 | 3.11E-16 | 0.004 | 0.017 | 0.783 |
|  | rs825476 | C | T | -0.052 | 0.007 | 6.80E-13 | -0.008 | 0.016 | 0.627 |
|  | rs840967 | A | C | -0.050 | 0.008 | 5.44E-10 | 0.008 | 0.016 | 0.642 |
|  | rs849135 | A | G | -0.100 | 0.007 | 1.04E-43 | 0.018 | 0.016 | 0.259 |
|  | rs853974 | C | T | -0.060 | 0.009 | 7.86E-12 | -0.026 | 0.018 | 0.138 |
|  | rs9369425 | A | G | -0.055 | 0.009 | 1.13E-10 | -0.036 | 0.018 | 0.046 |
|  | rs9894220 | A | G | 0.059 | 0.008 | 1.52E-13 | 0.028 | 0.016 | 0.072 |
|  | rs9928094 | A | G | -0.105 | 0.007 | 3.59E-47 | 0.001 | 0.016 | 0.968 |
|  | rs993380 | A | G | 0.051 | 0.008 | 4.59E-10 | 0.000 | 0.016 | 0.982 |
|  | rs9940149 | A | G | -0.058 | 0.010 | 9.29E-10 | -0.021 | 0.020 | 0.327 |
| *Desulfovibrionaceae* | rs10077431 | A | C | -0.049 | 0.009 | 4.75E-08 | -0.019 | 0.014 | 0.187 |
|  | rs10087241 | A | G | -0.048 | 0.008 | 2.80E-09 | -0.008 | 0.012 | 0.456 |
|  | rs10100265 | A | C | 0.049 | 0.008 | 6.29E-10 | 0.003 | 0.012 | 0.848 |
|  | rs10114341 | C | T | -0.041 | 0.007 | 1.15E-08 | 0.023 | 0.012 | 0.081 |
|  | rs10401969 | C | T | 0.092 | 0.013 | 4.13E-12 | -0.010 | 0.022 | 0.616 |
|  | rs1050226 | G | A | -0.049 | 0.007 | 3.34E-11 | -0.018 | 0.012 | 0.139 |
|  | rs1061813 | A | G | -0.043 | 0.007 | 3.37E-09 | -0.009 | 0.012 | 0.368 |
|  | rs1063355 | T | G | -0.071 | 0.008 | 3.72E-19 | -0.001 | 0.012 | 0.924 |
|  | rs10740322 | A | G | 0.048 | 0.009 | 2.11E-08 | 0.011 | 0.012 | 0.382 |
|  | rs10811661 | C | T | -0.157 | 0.010 | 4.13E-58 | -0.015 | 0.015 | 0.354 |
|  | rs10842994 | C | T | 0.076 | 0.009 | 1.02E-16 | -0.015 | 0.014 | 0.321 |
|  | rs10974438 | A | C | -0.059 | 0.008 | 3.01E-15 | -0.010 | 0.012 | 0.395 |
|  | rs11098676 | C | T | 0.054 | 0.010 | 2.03E-08 | -0.019 | 0.014 | 0.205 |
|  | rs11107116 | G | T | -0.047 | 0.009 | 3.75E-08 | 0.003 | 0.014 | 0.769 |
|  | rs1111875 | T | C | -0.095 | 0.007 | 3.61E-39 | -0.009 | 0.012 | 0.446 |
|  | rs11257655 | C | T | -0.074 | 0.009 | 1.97E-17 | 0.006 | 0.014 | 0.675 |
|  | rs1127655 | C | T | 0.044 | 0.008 | 2.47E-08 | -0.003 | 0.012 | 0.808 |
|  | rs11708067 | A | G | 0.097 | 0.009 | 5.93E-29 | -0.004 | 0.014 | 0.770 |
|  | rs11925227 | A | G | -0.053 | 0.010 | 2.25E-08 | 0.003 | 0.015 | 0.846 |
|  | rs11926707 | C | T | 0.046 | 0.008 | 1.69E-08 | 0.011 | 0.012 | 0.382 |
|  | rs12088739 | A | G | 0.088 | 0.013 | 9.79E-12 | -0.005 | 0.020 | 0.839 |
|  | rs12299509 | A | G | -0.047 | 0.007 | 2.09E-10 | 0.007 | 0.012 | 0.525 |
|  | rs12617659 | C | T | 0.069 | 0.010 | 2.83E-11 | -0.005 | 0.016 | 0.768 |
|  | rs12910825 | A | G | -0.052 | 0.007 | 2.16E-12 | -0.009 | 0.012 | 0.431 |
|  | rs12945601 | C | T | -0.048 | 0.008 | 1.72E-09 | -0.018 | 0.012 | 0.127 |
|  | rs12970134 | A | G | 0.056 | 0.008 | 5.31E-12 | 0.018 | 0.013 | 0.170 |
|  | rs13239186 | C | T | -0.054 | 0.009 | 2.70E-10 | -0.006 | 0.013 | 0.603 |
|  | rs13330951 | A | G | 0.046 | 0.008 | 1.54E-08 | 0.022 | 0.012 | 0.057 |
|  | rs13389219 | C | T | 0.072 | 0.007 | 2.11E-22 | 0.018 | 0.012 | 0.164 |
|  | rs1359790 | G | A | 0.080 | 0.008 | 2.80E-23 | -0.003 | 0.013 | 0.851 |
|  | rs1496653 | G | A | -0.077 | 0.009 | 2.57E-18 | -0.004 | 0.014 | 0.769 |
|  | rs1552224 | C | A | -0.103 | 0.010 | 8.64E-25 | -0.007 | 0.016 | 0.579 |
|  | rs16988333 | A | G | 0.075 | 0.013 | 9.17E-09 | 0.029 | 0.023 | 0.192 |
|  | rs17086692 | G | T | 0.047 | 0.008 | 2.48E-08 | -0.035 | 0.012 | 0.004 |
|  | rs17168486 | C | T | -0.074 | 0.009 | 2.18E-15 | -0.009 | 0.015 | 0.549 |
|  | rs17405722 | A | G | 0.087 | 0.015 | 2.28E-09 | -0.045 | 0.023 | 0.054 |
|  | rs17631783 | C | T | 0.049 | 0.009 | 3.95E-08 | 0.011 | 0.013 | 0.374 |
|  | rs17791513 | A | G | 0.103 | 0.015 | 4.61E-12 | 0.021 | 0.023 | 0.321 |
|  | rs1801214 | C | T | 0.090 | 0.007 | 5.52E-34 | -0.008 | 0.012 | 0.479 |
|  | rs1899951 | T | C | -0.112 | 0.011 | 1.64E-24 | 0.013 | 0.017 | 0.396 |
|  | rs2237892 | C | T | 0.096 | 0.016 | 8.75E-10 | 0.008 | 0.023 | 0.520 |
|  | rs2246618 | C | T | -0.051 | 0.008 | 1.20E-09 | -0.001 | 0.012 | 0.934 |
|  | rs2261181 | C | T | -0.099 | 0.012 | 9.18E-17 | -0.022 | 0.019 | 0.234 |
|  | rs2294120 | G | A | -0.044 | 0.008 | 1.62E-08 | -0.003 | 0.012 | 0.811 |
|  | rs2296173 | A | G | -0.065 | 0.009 | 7.66E-14 | 0.011 | 0.014 | 0.433 |
|  | rs2299383 | C | T | -0.041 | 0.007 | 1.49E-08 | 0.010 | 0.012 | 0.405 |
|  | rs243019 | T | C | -0.057 | 0.007 | 2.29E-15 | 0.013 | 0.012 | 0.284 |
|  | rs2493394 | G | A | 0.073 | 0.011 | 1.15E-10 | 0.043 | 0.019 | 0.031 |
|  | rs2796441 | G | A | 0.072 | 0.007 | 1.96E-22 | -0.006 | 0.012 | 0.630 |
|  | rs2820426 | G | A | 0.052 | 0.007 | 1.30E-12 | -0.003 | 0.012 | 0.783 |
|  | rs2867125 | T | C | -0.060 | 0.010 | 4.33E-10 | -0.007 | 0.015 | 0.697 |
|  | rs2908282 | G | A | -0.055 | 0.009 | 4.25E-09 | 0.016 | 0.015 | 0.284 |
|  | rs2925979 | T | C | 0.053 | 0.008 | 9.06E-12 | -0.002 | 0.013 | 0.824 |
|  | rs2943656 | A | G | -0.090 | 0.007 | 6.70E-34 | 0.006 | 0.012 | 0.611 |
|  | rs3217992 | T | C | 0.053 | 0.007 | 7.23E-13 | 0.013 | 0.012 | 0.287 |
|  | rs340874 | T | C | -0.063 | 0.007 | 8.41E-18 | -0.011 | 0.012 | 0.346 |
|  | rs348330 | G | A | 0.049 | 0.008 | 1.86E-09 | -0.005 | 0.012 | 0.717 |
|  | rs3756784 | G | T | 0.051 | 0.009 | 2.59E-08 | -0.016 | 0.014 | 0.264 |
|  | rs3802177 | G | A | 0.122 | 0.008 | 2.32E-52 | -0.004 | 0.012 | 0.775 |
|  | rs459193 | G | A | 0.071 | 0.008 | 8.81E-18 | -0.001 | 0.013 | 0.968 |
|  | rs4622883 | A | G | 0.044 | 0.008 | 3.02E-08 | -0.008 | 0.012 | 0.475 |
|  | rs4686471 | C | T | 0.053 | 0.008 | 4.28E-11 | -0.007 | 0.012 | 0.551 |
|  | rs4812829 | A | G | 0.053 | 0.010 | 2.44E-08 | 0.015 | 0.015 | 0.346 |
|  | rs4823182 | A | G | -0.048 | 0.008 | 3.36E-10 | -0.002 | 0.012 | 0.865 |
|  | rs4865796 | A | G | 0.053 | 0.008 | 1.33E-11 | -0.005 | 0.013 | 0.748 |
|  | rs516946 | T | C | -0.082 | 0.009 | 3.16E-22 | 0.012 | 0.014 | 0.394 |
|  | rs5215 | C | T | 0.068 | 0.007 | 2.09E-20 | -0.014 | 0.012 | 0.220 |
|  | rs576674 | A | G | -0.065 | 0.010 | 1.79E-11 | 0.045 | 0.015 | 0.003 |
|  | rs6059662 | A | G | -0.045 | 0.008 | 1.51E-08 | -0.002 | 0.013 | 0.831 |
|  | rs61953351 | G | T | 0.070 | 0.009 | 1.98E-14 | 0.003 | 0.013 | 0.777 |
|  | rs622217 | C | T | -0.049 | 0.008 | 3.13E-10 | -0.003 | 0.012 | 0.817 |
|  | rs6515236 | A | C | 0.050 | 0.009 | 3.34E-08 | 0.009 | 0.013 | 0.494 |
|  | rs67232546 | C | T | -0.060 | 0.010 | 4.66E-10 | -0.002 | 0.015 | 0.946 |
|  | rs6767484 | A | G | -0.121 | 0.008 | 2.70E-56 | -0.004 | 0.012 | 0.758 |
|  | rs6785040 | C | T | -0.063 | 0.011 | 1.26E-08 | -0.003 | 0.016 | 0.878 |
|  | rs6795735 | C | T | 0.056 | 0.007 | 1.63E-14 | -0.006 | 0.012 | 0.643 |
|  | rs6878122 | A | G | -0.056 | 0.008 | 1.19E-12 | -0.007 | 0.013 | 0.498 |
|  | rs6960043 | C | T | 0.064 | 0.007 | 3.61E-19 | -0.005 | 0.012 | 0.650 |
|  | rs7144011 | G | T | -0.048 | 0.009 | 1.64E-08 | 0.001 | 0.014 | 0.948 |
|  | rs7177055 | A | G | 0.065 | 0.008 | 2.75E-16 | -0.002 | 0.013 | 0.919 |
|  | rs7240767 | C | T | 0.045 | 0.008 | 2.16E-08 | -0.004 | 0.012 | 0.759 |
|  | rs72892910 | G | T | -0.065 | 0.010 | 6.43E-11 | -0.025 | 0.015 | 0.085 |
|  | rs735949 | C | T | -0.071 | 0.011 | 1.95E-11 | -0.017 | 0.017 | 0.343 |
|  | rs753270 | C | T | 0.053 | 0.008 | 2.70E-11 | 0.002 | 0.012 | 0.900 |
|  | rs7561798 | A | G | -0.040 | 0.007 | 2.79E-08 | 0.015 | 0.012 | 0.198 |
|  | rs7572970 | A | G | -0.059 | 0.009 | 1.39E-11 | -0.001 | 0.013 | 0.936 |
|  | rs7607777 | G | T | 0.137 | 0.013 | 9.40E-28 | -0.002 | 0.019 | 0.923 |
|  | rs7674212 | G | T | 0.047 | 0.008 | 6.18E-10 | -0.008 | 0.012 | 0.501 |
|  | rs7685296 | C | T | 0.051 | 0.008 | 2.32E-10 | -0.006 | 0.013 | 0.612 |
|  | rs7756992 | A | G | -0.130 | 0.008 | 6.00E-62 | 0.018 | 0.013 | 0.168 |
|  | rs7786095 | A | G | 0.074 | 0.013 | 9.64E-09 | -0.010 | 0.020 | 0.628 |
|  | rs780094 | T | C | -0.069 | 0.007 | 5.16E-21 | -0.006 | 0.012 | 0.624 |
|  | rs7845219 | C | T | -0.042 | 0.007 | 4.54E-09 | -0.010 | 0.012 | 0.412 |
|  | rs7903146 | C | T | -0.306 | 0.008 | 1.00E-200 | -0.011 | 0.013 | 0.390 |
|  | rs7929543 | A | C | -0.083 | 0.014 | 2.20E-09 | -0.011 | 0.020 | 0.507 |
|  | rs7955901 | C | T | 0.044 | 0.007 | 7.16E-10 | 0.017 | 0.012 | 0.152 |
|  | rs8068804 | A | G | 0.059 | 0.008 | 4.41E-14 | 0.000 | 0.012 | 0.975 |
|  | rs8108269 | G | T | 0.064 | 0.008 | 3.11E-16 | 0.002 | 0.012 | 0.879 |
|  | rs825476 | C | T | -0.052 | 0.007 | 6.80E-13 | -0.025 | 0.012 | 0.028 |
|  | rs840967 | A | C | -0.050 | 0.008 | 5.44E-10 | -0.012 | 0.012 | 0.353 |
|  | rs849135 | A | G | -0.100 | 0.007 | 1.04E-43 | -0.008 | 0.012 | 0.469 |
|  | rs853974 | C | T | -0.060 | 0.009 | 7.86E-12 | -0.015 | 0.013 | 0.236 |
|  | rs9369425 | A | G | -0.055 | 0.009 | 1.13E-10 | 0.002 | 0.013 | 0.846 |
|  | rs9894220 | A | G | 0.059 | 0.008 | 1.52E-13 | 0.002 | 0.012 | 0.895 |
|  | rs9928094 | A | G | -0.105 | 0.007 | 3.59E-47 | -0.020 | 0.012 | 0.091 |
|  | rs993380 | A | G | 0.051 | 0.008 | 4.59E-10 | -0.007 | 0.012 | 0.556 |
|  | rs9940149 | A | G | -0.058 | 0.010 | 9.29E-10 | -0.018 | 0.015 | 0.276 |
| *Enterobacteriaceae* | rs10077431 | A | C | -0.049 | 0.009 | 4.75E-08 | -0.003 | 0.015 | 0.839 |
|  | rs10087241 | A | G | -0.048 | 0.008 | 2.80E-09 | 0.010 | 0.013 | 0.418 |
|  | rs10100265 | A | C | 0.049 | 0.008 | 6.29E-10 | -0.009 | 0.012 | 0.473 |
|  | rs10114341 | C | T | -0.041 | 0.007 | 1.15E-08 | 0.009 | 0.012 | 0.465 |
|  | rs10401969 | C | T | 0.092 | 0.013 | 4.13E-12 | -0.031 | 0.023 | 0.213 |
|  | rs1050226 | G | A | -0.049 | 0.007 | 3.34E-11 | 0.007 | 0.012 | 0.573 |
|  | rs1061813 | A | G | -0.043 | 0.007 | 3.37E-09 | 0.006 | 0.012 | 0.680 |
|  | rs1063355 | T | G | -0.071 | 0.008 | 3.72E-19 | 0.014 | 0.012 | 0.267 |
|  | rs10740322 | A | G | 0.048 | 0.009 | 2.11E-08 | -0.008 | 0.013 | 0.557 |
|  | rs10811661 | C | T | -0.157 | 0.010 | 4.13E-58 | -0.001 | 0.016 | 0.989 |
|  | rs10842994 | C | T | 0.076 | 0.009 | 1.02E-16 | -0.026 | 0.015 | 0.098 |
|  | rs10974438 | A | C | -0.059 | 0.008 | 3.01E-15 | 0.002 | 0.013 | 0.891 |
|  | rs11098676 | C | T | 0.054 | 0.010 | 2.03E-08 | 0.001 | 0.015 | 0.919 |
|  | rs11107116 | G | T | -0.047 | 0.009 | 3.75E-08 | 0.006 | 0.015 | 0.648 |
|  | rs1111875 | T | C | -0.095 | 0.007 | 3.61E-39 | 0.004 | 0.012 | 0.749 |
|  | rs11257655 | C | T | -0.074 | 0.009 | 1.97E-17 | 0.004 | 0.015 | 0.817 |
|  | rs1127655 | C | T | 0.044 | 0.008 | 2.47E-08 | 0.000 | 0.012 | 0.978 |
|  | rs11708067 | A | G | 0.097 | 0.009 | 5.93E-29 | 0.021 | 0.015 | 0.150 |
|  | rs11925227 | A | G | -0.053 | 0.010 | 2.25E-08 | 0.005 | 0.016 | 0.734 |
|  | rs11926707 | C | T | 0.046 | 0.008 | 1.69E-08 | 0.007 | 0.013 | 0.595 |
|  | rs12088739 | A | G | 0.088 | 0.013 | 9.79E-12 | 0.003 | 0.021 | 0.901 |
|  | rs12299509 | A | G | -0.047 | 0.007 | 2.09E-10 | 0.001 | 0.012 | 0.914 |
|  | rs12617659 | C | T | 0.069 | 0.010 | 2.83E-11 | 0.019 | 0.017 | 0.278 |
|  | rs12910825 | A | G | -0.052 | 0.007 | 2.16E-12 | 0.021 | 0.013 | 0.097 |
|  | rs12945601 | C | T | -0.048 | 0.008 | 1.72E-09 | 0.005 | 0.013 | 0.648 |
|  | rs12970134 | A | G | 0.056 | 0.008 | 5.31E-12 | 0.009 | 0.014 | 0.588 |
|  | rs13239186 | C | T | -0.054 | 0.009 | 2.70E-10 | -0.029 | 0.013 | 0.029 |
|  | rs13330951 | A | G | 0.046 | 0.008 | 1.54E-08 | 0.000 | 0.012 | 0.976 |
|  | rs13389219 | C | T | 0.072 | 0.007 | 2.11E-22 | -0.002 | 0.013 | 0.825 |
|  | rs1359790 | G | A | 0.080 | 0.008 | 2.80E-23 | 0.005 | 0.014 | 0.682 |
|  | rs1496653 | G | A | -0.077 | 0.009 | 2.57E-18 | -0.007 | 0.015 | 0.579 |
|  | rs1552224 | C | A | -0.103 | 0.010 | 8.64E-25 | -0.004 | 0.017 | 0.864 |
|  | rs16988333 | A | G | 0.075 | 0.013 | 9.17E-09 | 0.016 | 0.024 | 0.444 |
|  | rs17086692 | G | T | 0.047 | 0.008 | 2.48E-08 | -0.004 | 0.013 | 0.774 |
|  | rs17168486 | C | T | -0.074 | 0.009 | 2.18E-15 | 0.018 | 0.016 | 0.230 |
|  | rs17405722 | A | G | 0.087 | 0.015 | 2.28E-09 | -0.009 | 0.024 | 0.680 |
|  | rs17631783 | C | T | 0.049 | 0.009 | 3.95E-08 | -0.007 | 0.014 | 0.789 |
|  | rs17791513 | A | G | 0.103 | 0.015 | 4.61E-12 | 0.032 | 0.025 | 0.232 |
|  | rs1801214 | C | T | 0.090 | 0.007 | 5.52E-34 | -0.011 | 0.013 | 0.342 |
|  | rs1899951 | T | C | -0.112 | 0.011 | 1.64E-24 | 0.017 | 0.018 | 0.337 |
|  | rs2237892 | C | T | 0.096 | 0.016 | 8.75E-10 | -0.012 | 0.024 | 0.769 |
|  | rs2246618 | C | T | -0.051 | 0.008 | 1.20E-09 | 0.002 | 0.013 | 0.908 |
|  | rs2261181 | C | T | -0.099 | 0.012 | 9.18E-17 | 0.001 | 0.020 | 0.967 |
|  | rs2294120 | G | A | -0.044 | 0.008 | 1.62E-08 | 0.008 | 0.012 | 0.507 |
|  | rs2296173 | A | G | -0.065 | 0.009 | 7.66E-14 | -0.055 | 0.015 | 0.000 |
|  | rs2299383 | C | T | -0.041 | 0.007 | 1.49E-08 | -0.009 | 0.012 | 0.456 |
|  | rs243019 | T | C | -0.057 | 0.007 | 2.29E-15 | 0.009 | 0.012 | 0.452 |
|  | rs2493394 | G | A | 0.073 | 0.011 | 1.15E-10 | -0.001 | 0.020 | 0.979 |
|  | rs2796441 | G | A | 0.072 | 0.007 | 1.96E-22 | 0.007 | 0.012 | 0.584 |
|  | rs2820426 | G | A | 0.052 | 0.007 | 1.30E-12 | -0.007 | 0.013 | 0.590 |
|  | rs2867125 | T | C | -0.060 | 0.010 | 4.33E-10 | -0.022 | 0.016 | 0.218 |
|  | rs2908282 | G | A | -0.055 | 0.009 | 4.25E-09 | 0.005 | 0.016 | 0.809 |
|  | rs2925979 | T | C | 0.053 | 0.008 | 9.06E-12 | -0.009 | 0.013 | 0.508 |
|  | rs2943656 | A | G | -0.090 | 0.007 | 6.70E-34 | 0.010 | 0.013 | 0.502 |
|  | rs3217992 | T | C | 0.053 | 0.007 | 7.23E-13 | -0.028 | 0.013 | 0.025 |
|  | rs340874 | T | C | -0.063 | 0.007 | 8.41E-18 | -0.003 | 0.012 | 0.812 |
|  | rs348330 | G | A | 0.049 | 0.008 | 1.86E-09 | -0.004 | 0.013 | 0.763 |
|  | rs3756784 | G | T | 0.051 | 0.009 | 2.59E-08 | 0.004 | 0.015 | 0.772 |
|  | rs3802177 | G | A | 0.122 | 0.008 | 2.32E-52 | -0.015 | 0.013 | 0.251 |
|  | rs459193 | G | A | 0.071 | 0.008 | 8.81E-18 | 0.005 | 0.014 | 0.730 |
|  | rs4622883 | A | G | 0.044 | 0.008 | 3.02E-08 | 0.001 | 0.012 | 0.914 |
|  | rs4686471 | C | T | 0.053 | 0.008 | 4.28E-11 | 0.003 | 0.013 | 0.871 |
|  | rs4812829 | A | G | 0.053 | 0.010 | 2.44E-08 | 0.006 | 0.016 | 0.668 |
|  | rs4823182 | A | G | -0.048 | 0.008 | 3.36E-10 | -0.017 | 0.013 | 0.182 |
|  | rs4865796 | A | G | 0.053 | 0.008 | 1.33E-11 | -0.008 | 0.013 | 0.637 |
|  | rs516946 | T | C | -0.082 | 0.009 | 3.16E-22 | -0.013 | 0.014 | 0.416 |
|  | rs5215 | C | T | 0.068 | 0.007 | 2.09E-20 | -0.027 | 0.013 | 0.034 |
|  | rs576674 | A | G | -0.065 | 0.010 | 1.79E-11 | 0.024 | 0.016 | 0.127 |
|  | rs6059662 | A | G | -0.045 | 0.008 | 1.51E-08 | 0.000 | 0.013 | 0.937 |
|  | rs61953351 | G | T | 0.070 | 0.009 | 1.98E-14 | 0.008 | 0.014 | 0.534 |
|  | rs622217 | C | T | -0.049 | 0.008 | 3.13E-10 | 0.005 | 0.012 | 0.652 |
|  | rs6515236 | A | C | 0.050 | 0.009 | 3.34E-08 | 0.005 | 0.014 | 0.724 |
|  | rs67232546 | C | T | -0.060 | 0.010 | 4.66E-10 | -0.026 | 0.015 | 0.089 |
|  | rs6767484 | A | G | -0.121 | 0.008 | 2.70E-56 | -0.010 | 0.013 | 0.425 |
|  | rs6785040 | C | T | -0.063 | 0.011 | 1.26E-08 | -0.024 | 0.016 | 0.114 |
|  | rs6795735 | C | T | 0.056 | 0.007 | 1.63E-14 | 0.009 | 0.012 | 0.464 |
|  | rs6878122 | A | G | -0.056 | 0.008 | 1.19E-12 | -0.005 | 0.014 | 0.687 |
|  | rs6960043 | C | T | 0.064 | 0.007 | 3.61E-19 | 0.004 | 0.012 | 0.777 |
|  | rs7144011 | G | T | -0.048 | 0.009 | 1.64E-08 | -0.008 | 0.015 | 0.545 |
|  | rs7177055 | A | G | 0.065 | 0.008 | 2.75E-16 | 0.018 | 0.013 | 0.181 |
|  | rs7240767 | C | T | 0.045 | 0.008 | 2.16E-08 | 0.017 | 0.013 | 0.176 |
|  | rs72892910 | G | T | -0.065 | 0.010 | 6.43E-11 | -0.026 | 0.016 | 0.077 |
|  | rs735949 | C | T | -0.071 | 0.011 | 1.95E-11 | 0.020 | 0.018 | 0.289 |
|  | rs753270 | C | T | 0.053 | 0.008 | 2.70E-11 | 0.011 | 0.012 | 0.357 |
|  | rs7561798 | A | G | -0.040 | 0.007 | 2.79E-08 | -0.006 | 0.012 | 0.614 |
|  | rs7572970 | A | G | -0.059 | 0.009 | 1.39E-11 | 0.016 | 0.014 | 0.274 |
|  | rs7607777 | G | T | 0.137 | 0.013 | 9.40E-28 | -0.023 | 0.020 | 0.291 |
|  | rs7674212 | G | T | 0.047 | 0.008 | 6.18E-10 | -0.005 | 0.012 | 0.691 |
|  | rs7685296 | C | T | 0.051 | 0.008 | 2.32E-10 | 0.007 | 0.013 | 0.638 |
|  | rs7756992 | A | G | -0.130 | 0.008 | 6.00E-62 | 0.018 | 0.013 | 0.163 |
|  | rs7786095 | A | G | 0.074 | 0.013 | 9.64E-09 | 0.029 | 0.021 | 0.154 |
|  | rs780094 | T | C | -0.069 | 0.007 | 5.16E-21 | -0.013 | 0.013 | 0.325 |
|  | rs7845219 | C | T | -0.042 | 0.007 | 4.54E-09 | 0.001 | 0.012 | 0.920 |
|  | rs7903146 | C | T | -0.306 | 0.008 | 1.00E-200 | -0.010 | 0.014 | 0.477 |
|  | rs7929543 | A | C | -0.083 | 0.014 | 2.20E-09 | -0.014 | 0.021 | 0.554 |
|  | rs7955901 | C | T | 0.044 | 0.007 | 7.16E-10 | -0.039 | 0.012 | 0.001 |
|  | rs8068804 | A | G | 0.059 | 0.008 | 4.41E-14 | -0.012 | 0.013 | 0.358 |
|  | rs8108269 | G | T | 0.064 | 0.008 | 3.11E-16 | -0.009 | 0.013 | 0.528 |
|  | rs825476 | C | T | -0.052 | 0.007 | 6.80E-13 | -0.034 | 0.012 | 0.006 |
|  | rs840967 | A | C | -0.050 | 0.008 | 5.44E-10 | 0.017 | 0.012 | 0.141 |
|  | rs849135 | A | G | -0.100 | 0.007 | 1.04E-43 | 0.007 | 0.012 | 0.563 |
|  | rs853974 | C | T | -0.060 | 0.009 | 7.86E-12 | -0.006 | 0.014 | 0.690 |
|  | rs9369425 | A | G | -0.055 | 0.009 | 1.13E-10 | -0.001 | 0.014 | 0.964 |
|  | rs9894220 | A | G | 0.059 | 0.008 | 1.52E-13 | 0.006 | 0.012 | 0.645 |
|  | rs9928094 | A | G | -0.105 | 0.007 | 3.59E-47 | 0.006 | 0.012 | 0.624 |
|  | rs993380 | A | G | 0.051 | 0.008 | 4.59E-10 | 0.007 | 0.013 | 0.600 |
|  | rs9940149 | A | G | -0.058 | 0.010 | 9.29E-10 | 0.037 | 0.015 | 0.019 |
| *Erysipelotrichaceae* | rs10077431 | A | C | -0.049 | 0.009 | 4.75E-08 | -0.012 | 0.013 | 0.429 |
|  | rs10087241 | A | G | -0.048 | 0.008 | 2.80E-09 | -0.016 | 0.011 | 0.187 |
|  | rs10100265 | A | C | 0.049 | 0.008 | 6.29E-10 | -0.003 | 0.011 | 0.747 |
|  | rs10114341 | C | T | -0.041 | 0.007 | 1.15E-08 | 0.016 | 0.011 | 0.142 |
|  | rs10401969 | C | T | 0.092 | 0.013 | 4.13E-12 | -0.026 | 0.020 | 0.154 |
|  | rs1050226 | G | A | -0.049 | 0.007 | 3.34E-11 | 0.009 | 0.011 | 0.413 |
|  | rs1061813 | A | G | -0.043 | 0.007 | 3.37E-09 | -0.006 | 0.011 | 0.597 |
|  | rs1063355 | T | G | -0.071 | 0.008 | 3.72E-19 | 0.010 | 0.011 | 0.331 |
|  | rs10740322 | A | G | 0.048 | 0.009 | 2.11E-08 | 0.006 | 0.011 | 0.626 |
|  | rs10811661 | C | T | -0.157 | 0.010 | 4.13E-58 | 0.008 | 0.014 | 0.580 |
|  | rs10842994 | C | T | 0.076 | 0.009 | 1.02E-16 | -0.015 | 0.013 | 0.234 |
|  | rs10974438 | A | C | -0.059 | 0.008 | 3.01E-15 | 0.014 | 0.011 | 0.210 |
|  | rs11098676 | C | T | 0.054 | 0.010 | 2.03E-08 | 0.003 | 0.013 | 0.759 |
|  | rs11107116 | G | T | -0.047 | 0.009 | 3.75E-08 | -0.007 | 0.013 | 0.576 |
|  | rs1111875 | T | C | -0.095 | 0.007 | 3.61E-39 | -0.002 | 0.011 | 0.853 |
|  | rs11257655 | C | T | -0.074 | 0.009 | 1.97E-17 | -0.006 | 0.013 | 0.666 |
|  | rs1127655 | C | T | 0.044 | 0.008 | 2.47E-08 | 0.002 | 0.011 | 0.821 |
|  | rs11708067 | A | G | 0.097 | 0.009 | 5.93E-29 | 0.008 | 0.013 | 0.551 |
|  | rs11925227 | A | G | -0.053 | 0.010 | 2.25E-08 | 0.014 | 0.014 | 0.325 |
|  | rs11926707 | C | T | 0.046 | 0.008 | 1.69E-08 | -0.016 | 0.011 | 0.137 |
|  | rs12088739 | A | G | 0.088 | 0.013 | 9.79E-12 | -0.011 | 0.018 | 0.516 |
|  | rs12299509 | A | G | -0.047 | 0.007 | 2.09E-10 | -0.012 | 0.011 | 0.259 |
|  | rs12617659 | C | T | 0.069 | 0.010 | 2.83E-11 | 0.007 | 0.015 | 0.745 |
|  | rs12910825 | A | G | -0.052 | 0.007 | 2.16E-12 | 0.003 | 0.011 | 0.757 |
|  | rs12945601 | C | T | -0.048 | 0.008 | 1.72E-09 | -0.014 | 0.011 | 0.204 |
|  | rs12970134 | A | G | 0.056 | 0.008 | 5.31E-12 | -0.006 | 0.012 | 0.562 |
|  | rs13239186 | C | T | -0.054 | 0.009 | 2.70E-10 | 0.007 | 0.011 | 0.556 |
|  | rs13330951 | A | G | 0.046 | 0.008 | 1.54E-08 | -0.001 | 0.011 | 0.923 |
|  | rs13389219 | C | T | 0.072 | 0.007 | 2.11E-22 | 0.004 | 0.011 | 0.782 |
|  | rs1359790 | G | A | 0.080 | 0.008 | 2.80E-23 | 0.008 | 0.012 | 0.515 |
|  | rs1496653 | G | A | -0.077 | 0.009 | 2.57E-18 | -0.018 | 0.013 | 0.184 |
|  | rs1552224 | C | A | -0.103 | 0.010 | 8.64E-25 | 0.006 | 0.015 | 0.716 |
|  | rs16988333 | A | G | 0.075 | 0.013 | 9.17E-09 | 0.014 | 0.021 | 0.544 |
|  | rs17086692 | G | T | 0.047 | 0.008 | 2.48E-08 | 0.013 | 0.011 | 0.245 |
|  | rs17168486 | C | T | -0.074 | 0.009 | 2.18E-15 | -0.010 | 0.013 | 0.487 |
|  | rs17405722 | A | G | 0.087 | 0.015 | 2.28E-09 | -0.010 | 0.021 | 0.621 |
|  | rs17631783 | C | T | 0.049 | 0.009 | 3.95E-08 | 0.006 | 0.012 | 0.745 |
|  | rs17791513 | A | G | 0.103 | 0.015 | 4.61E-12 | -0.061 | 0.021 | 0.004 |
|  | rs1801214 | C | T | 0.090 | 0.007 | 5.52E-34 | -0.002 | 0.011 | 0.764 |
|  | rs1899951 | T | C | -0.112 | 0.011 | 1.64E-24 | -0.009 | 0.015 | 0.574 |
|  | rs2237892 | C | T | 0.096 | 0.016 | 8.75E-10 | 0.036 | 0.021 | 0.106 |
|  | rs2246618 | C | T | -0.051 | 0.008 | 1.20E-09 | 0.017 | 0.011 | 0.158 |
|  | rs2261181 | C | T | -0.099 | 0.012 | 9.18E-17 | 0.012 | 0.018 | 0.523 |
|  | rs2294120 | G | A | -0.044 | 0.008 | 1.62E-08 | -0.007 | 0.011 | 0.524 |
|  | rs2296173 | A | G | -0.065 | 0.009 | 7.66E-14 | 0.017 | 0.013 | 0.221 |
|  | rs2299383 | C | T | -0.041 | 0.007 | 1.49E-08 | 0.010 | 0.011 | 0.325 |
|  | rs243019 | T | C | -0.057 | 0.007 | 2.29E-15 | -0.001 | 0.011 | 0.960 |
|  | rs2493394 | G | A | 0.073 | 0.011 | 1.15E-10 | 0.007 | 0.017 | 0.603 |
|  | rs2796441 | G | A | 0.072 | 0.007 | 1.96E-22 | 0.011 | 0.011 | 0.285 |
|  | rs2820426 | G | A | 0.052 | 0.007 | 1.30E-12 | -0.003 | 0.011 | 0.767 |
|  | rs2867125 | T | C | -0.060 | 0.010 | 4.33E-10 | 0.010 | 0.014 | 0.442 |
|  | rs2908282 | G | A | -0.055 | 0.009 | 4.25E-09 | -0.014 | 0.014 | 0.283 |
|  | rs2925979 | T | C | 0.053 | 0.008 | 9.06E-12 | 0.005 | 0.011 | 0.640 |
|  | rs2943656 | A | G | -0.090 | 0.007 | 6.70E-34 | -0.004 | 0.011 | 0.552 |
|  | rs3217992 | T | C | 0.053 | 0.007 | 7.23E-13 | -0.002 | 0.011 | 0.819 |
|  | rs340874 | T | C | -0.063 | 0.007 | 8.41E-18 | -0.004 | 0.011 | 0.731 |
|  | rs348330 | G | A | 0.049 | 0.008 | 1.86E-09 | -0.026 | 0.011 | 0.019 |
|  | rs3756784 | G | T | 0.051 | 0.009 | 2.59E-08 | -0.013 | 0.013 | 0.306 |
|  | rs3802177 | G | A | 0.122 | 0.008 | 2.32E-52 | -0.002 | 0.011 | 0.886 |
|  | rs459193 | G | A | 0.071 | 0.008 | 8.81E-18 | -0.022 | 0.012 | 0.080 |
|  | rs4622883 | A | G | 0.044 | 0.008 | 3.02E-08 | -0.019 | 0.011 | 0.061 |
|  | rs4686471 | C | T | 0.053 | 0.008 | 4.28E-11 | -0.002 | 0.011 | 0.787 |
|  | rs4812829 | A | G | 0.053 | 0.010 | 2.44E-08 | -0.004 | 0.014 | 0.770 |
|  | rs4823182 | A | G | -0.048 | 0.008 | 3.36E-10 | 0.002 | 0.011 | 0.854 |
|  | rs4865796 | A | G | 0.053 | 0.008 | 1.33E-11 | -0.020 | 0.012 | 0.075 |
|  | rs516946 | T | C | -0.082 | 0.009 | 3.16E-22 | 0.016 | 0.012 | 0.192 |
|  | rs5215 | C | T | 0.068 | 0.007 | 2.09E-20 | -0.015 | 0.011 | 0.158 |
|  | rs576674 | A | G | -0.065 | 0.010 | 1.79E-11 | 0.006 | 0.014 | 0.619 |
|  | rs6059662 | A | G | -0.045 | 0.008 | 1.51E-08 | -0.027 | 0.012 | 0.019 |
|  | rs61953351 | G | T | 0.070 | 0.009 | 1.98E-14 | 0.019 | 0.012 | 0.084 |
|  | rs622217 | C | T | -0.049 | 0.008 | 3.13E-10 | 0.016 | 0.011 | 0.123 |
|  | rs6515236 | A | C | 0.050 | 0.009 | 3.34E-08 | -0.005 | 0.012 | 0.653 |
|  | rs67232546 | C | T | -0.060 | 0.010 | 4.66E-10 | -0.005 | 0.013 | 0.686 |
|  | rs6767484 | A | G | -0.121 | 0.008 | 2.70E-56 | 0.013 | 0.011 | 0.264 |
|  | rs6785040 | C | T | -0.063 | 0.011 | 1.26E-08 | 0.007 | 0.014 | 0.647 |
|  | rs6795735 | C | T | 0.056 | 0.007 | 1.63E-14 | 0.012 | 0.011 | 0.222 |
|  | rs6878122 | A | G | -0.056 | 0.008 | 1.19E-12 | 0.012 | 0.012 | 0.359 |
|  | rs6960043 | C | T | 0.064 | 0.007 | 3.61E-19 | -0.004 | 0.011 | 0.677 |
|  | rs7144011 | G | T | -0.048 | 0.009 | 1.64E-08 | -0.007 | 0.013 | 0.595 |
|  | rs7177055 | A | G | 0.065 | 0.008 | 2.75E-16 | -0.009 | 0.011 | 0.447 |
|  | rs7240767 | C | T | 0.045 | 0.008 | 2.16E-08 | 0.006 | 0.011 | 0.601 |
|  | rs72892910 | G | T | -0.065 | 0.010 | 6.43E-11 | -0.028 | 0.014 | 0.037 |
|  | rs735949 | C | T | -0.071 | 0.011 | 1.95E-11 | -0.030 | 0.016 | 0.058 |
|  | rs753270 | C | T | 0.053 | 0.008 | 2.70E-11 | -0.006 | 0.011 | 0.608 |
|  | rs7561798 | A | G | -0.040 | 0.007 | 2.79E-08 | -0.009 | 0.011 | 0.382 |
|  | rs7572970 | A | G | -0.059 | 0.009 | 1.39E-11 | 0.004 | 0.012 | 0.718 |
|  | rs7607777 | G | T | 0.137 | 0.013 | 9.40E-28 | -0.046 | 0.017 | 0.007 |
|  | rs7674212 | G | T | 0.047 | 0.008 | 6.18E-10 | 0.002 | 0.011 | 0.813 |
|  | rs7685296 | C | T | 0.051 | 0.008 | 2.32E-10 | -0.017 | 0.012 | 0.166 |
|  | rs7729395 | C | T | -0.137 | 0.016 | 1.10E-17 | -0.088 | 0.056 | 0.114 |
|  | rs7756992 | A | G | -0.130 | 0.008 | 6.00E-62 | 0.009 | 0.012 | 0.428 |
|  | rs7786095 | A | G | 0.074 | 0.013 | 9.64E-09 | 0.032 | 0.018 | 0.076 |
|  | rs780094 | T | C | -0.069 | 0.007 | 5.16E-21 | 0.001 | 0.011 | 0.995 |
|  | rs7845219 | C | T | -0.042 | 0.007 | 4.54E-09 | -0.024 | 0.011 | 0.021 |
|  | rs7903146 | C | T | -0.306 | 0.008 | 1.00E-200 | -0.012 | 0.012 | 0.319 |
|  | rs7929543 | A | C | -0.083 | 0.014 | 2.20E-09 | -0.009 | 0.018 | 0.660 |
|  | rs7955901 | C | T | 0.044 | 0.007 | 7.16E-10 | -0.002 | 0.011 | 0.827 |
|  | rs8068804 | A | G | 0.059 | 0.008 | 4.41E-14 | -0.004 | 0.011 | 0.691 |
|  | rs8108269 | G | T | 0.064 | 0.008 | 3.11E-16 | 0.003 | 0.011 | 0.773 |
|  | rs825476 | C | T | -0.052 | 0.007 | 6.80E-13 | -0.005 | 0.011 | 0.656 |
|  | rs840967 | A | C | -0.050 | 0.008 | 5.44E-10 | 0.002 | 0.011 | 0.837 |
|  | rs849135 | A | G | -0.100 | 0.007 | 1.04E-43 | 0.013 | 0.011 | 0.211 |
|  | rs853974 | C | T | -0.060 | 0.009 | 7.86E-12 | 0.001 | 0.012 | 0.921 |
|  | rs9369425 | A | G | -0.055 | 0.009 | 1.13E-10 | -0.001 | 0.012 | 0.905 |
|  | rs9894220 | A | G | 0.059 | 0.008 | 1.52E-13 | 0.011 | 0.011 | 0.314 |
|  | rs9928094 | A | G | -0.105 | 0.007 | 3.59E-47 | 0.003 | 0.011 | 0.788 |
|  | rs993380 | A | G | 0.051 | 0.008 | 4.59E-10 | 0.019 | 0.011 | 0.087 |
|  | rs9940149 | A | G | -0.058 | 0.010 | 9.29E-10 | 0.020 | 0.013 | 0.146 |
| *Lachnospiraceae* | rs10077431 | A | C | -0.049 | 0.009 | 4.75E-08 | 0.017 | 0.013 | 0.266 |
|  | rs10087241 | A | G | -0.048 | 0.008 | 2.80E-09 | 0.007 | 0.011 | 0.583 |
|  | rs10100265 | A | C | 0.049 | 0.008 | 6.29E-10 | 0.002 | 0.011 | 0.841 |
|  | rs10114341 | C | T | -0.041 | 0.007 | 1.15E-08 | -0.006 | 0.011 | 0.669 |
|  | rs10401969 | C | T | 0.092 | 0.013 | 4.13E-12 | -0.016 | 0.020 | 0.405 |
|  | rs1050226 | G | A | -0.049 | 0.007 | 3.34E-11 | 0.008 | 0.011 | 0.463 |
|  | rs1061813 | A | G | -0.043 | 0.007 | 3.37E-09 | -0.005 | 0.011 | 0.739 |
|  | rs1063355 | T | G | -0.071 | 0.008 | 3.72E-19 | -0.012 | 0.011 | 0.259 |
|  | rs10740322 | A | G | 0.048 | 0.009 | 2.11E-08 | 0.008 | 0.011 | 0.476 |
|  | rs10811661 | C | T | -0.157 | 0.010 | 4.13E-58 | 0.009 | 0.014 | 0.617 |
|  | rs10842994 | C | T | 0.076 | 0.009 | 1.02E-16 | 0.015 | 0.013 | 0.249 |
|  | rs10974438 | A | C | -0.059 | 0.008 | 3.01E-15 | 0.008 | 0.011 | 0.464 |
|  | rs11098676 | C | T | 0.054 | 0.010 | 2.03E-08 | 0.013 | 0.013 | 0.356 |
|  | rs11107116 | G | T | -0.047 | 0.009 | 3.75E-08 | -0.001 | 0.013 | 0.927 |
|  | rs1111875 | T | C | -0.095 | 0.007 | 3.61E-39 | -0.018 | 0.011 | 0.097 |
|  | rs11257655 | C | T | -0.074 | 0.009 | 1.97E-17 | -0.017 | 0.013 | 0.194 |
|  | rs1127655 | C | T | 0.044 | 0.008 | 2.47E-08 | 0.012 | 0.010 | 0.251 |
|  | rs11708067 | A | G | 0.097 | 0.009 | 5.93E-29 | 0.000 | 0.013 | 0.938 |
|  | rs11925227 | A | G | -0.053 | 0.010 | 2.25E-08 | 0.013 | 0.014 | 0.341 |
|  | rs11926707 | C | T | 0.046 | 0.008 | 1.69E-08 | -0.019 | 0.011 | 0.093 |
|  | rs12088739 | A | G | 0.088 | 0.013 | 9.79E-12 | 0.031 | 0.018 | 0.108 |
|  | rs12299509 | A | G | -0.047 | 0.007 | 2.09E-10 | -0.025 | 0.010 | 0.017 |
|  | rs12617659 | C | T | 0.069 | 0.010 | 2.83E-11 | -0.017 | 0.015 | 0.242 |
|  | rs12910825 | A | G | -0.052 | 0.007 | 2.16E-12 | 0.003 | 0.011 | 0.794 |
|  | rs12945601 | C | T | -0.048 | 0.008 | 1.72E-09 | -0.001 | 0.011 | 0.913 |
|  | rs12970134 | A | G | 0.056 | 0.008 | 5.31E-12 | -0.008 | 0.012 | 0.461 |
|  | rs13239186 | C | T | -0.054 | 0.009 | 2.70E-10 | -0.003 | 0.011 | 0.832 |
|  | rs13330951 | A | G | 0.046 | 0.008 | 1.54E-08 | 0.010 | 0.011 | 0.365 |
|  | rs13389219 | C | T | 0.072 | 0.007 | 2.11E-22 | 0.009 | 0.011 | 0.352 |
|  | rs1359790 | G | A | 0.080 | 0.008 | 2.80E-23 | -0.006 | 0.012 | 0.603 |
|  | rs1496653 | G | A | -0.077 | 0.009 | 2.57E-18 | 0.030 | 0.013 | 0.016 |
|  | rs1552224 | C | A | -0.103 | 0.010 | 8.64E-25 | -0.006 | 0.015 | 0.750 |
|  | rs16988333 | A | G | 0.075 | 0.013 | 9.17E-09 | 0.038 | 0.021 | 0.071 |
|  | rs17086692 | G | T | 0.047 | 0.008 | 2.48E-08 | -0.005 | 0.011 | 0.649 |
|  | rs17168486 | C | T | -0.074 | 0.009 | 2.18E-15 | 0.007 | 0.013 | 0.559 |
|  | rs17405722 | A | G | 0.087 | 0.015 | 2.28E-09 | 0.028 | 0.021 | 0.191 |
|  | rs17631783 | C | T | 0.049 | 0.009 | 3.95E-08 | 0.012 | 0.012 | 0.436 |
|  | rs17791513 | A | G | 0.103 | 0.015 | 4.61E-12 | -0.022 | 0.021 | 0.276 |
|  | rs1801214 | C | T | 0.090 | 0.007 | 5.52E-34 | 0.000 | 0.011 | 0.936 |
|  | rs1899951 | T | C | -0.112 | 0.011 | 1.64E-24 | -0.007 | 0.015 | 0.641 |
|  | rs2237892 | C | T | 0.096 | 0.016 | 8.75E-10 | -0.010 | 0.021 | 0.421 |
|  | rs2246618 | C | T | -0.051 | 0.008 | 1.20E-09 | 0.016 | 0.011 | 0.155 |
|  | rs2261181 | C | T | -0.099 | 0.012 | 9.18E-17 | 0.000 | 0.018 | 0.976 |
|  | rs2294120 | G | A | -0.044 | 0.008 | 1.62E-08 | 0.007 | 0.010 | 0.486 |
|  | rs2296173 | A | G | -0.065 | 0.009 | 7.66E-14 | 0.009 | 0.013 | 0.455 |
|  | rs2299383 | C | T | -0.041 | 0.007 | 1.49E-08 | -0.014 | 0.011 | 0.174 |
|  | rs243019 | T | C | -0.057 | 0.007 | 2.29E-15 | 0.013 | 0.011 | 0.219 |
|  | rs2493394 | G | A | 0.073 | 0.011 | 1.15E-10 | -0.020 | 0.017 | 0.277 |
|  | rs2796441 | G | A | 0.072 | 0.007 | 1.96E-22 | -0.009 | 0.011 | 0.397 |
|  | rs2820426 | G | A | 0.052 | 0.007 | 1.30E-12 | 0.010 | 0.011 | 0.390 |
|  | rs2867125 | T | C | -0.060 | 0.010 | 4.33E-10 | 0.014 | 0.014 | 0.347 |
|  | rs2908282 | G | A | -0.055 | 0.009 | 4.25E-09 | 0.002 | 0.014 | 0.931 |
|  | rs2925979 | T | C | 0.053 | 0.008 | 9.06E-12 | 0.007 | 0.011 | 0.559 |
|  | rs2943656 | A | G | -0.090 | 0.007 | 6.70E-34 | -0.021 | 0.011 | 0.044 |
|  | rs3217992 | T | C | 0.053 | 0.007 | 7.23E-13 | 0.007 | 0.011 | 0.515 |
|  | rs340874 | T | C | -0.063 | 0.007 | 8.41E-18 | -0.005 | 0.011 | 0.617 |
|  | rs348330 | G | A | 0.049 | 0.008 | 1.86E-09 | -0.003 | 0.011 | 0.783 |
|  | rs3756784 | G | T | 0.051 | 0.009 | 2.59E-08 | 0.021 | 0.013 | 0.096 |
|  | rs3802177 | G | A | 0.122 | 0.008 | 2.32E-52 | 0.006 | 0.011 | 0.635 |
|  | rs459193 | G | A | 0.071 | 0.008 | 8.81E-18 | -0.012 | 0.012 | 0.345 |
|  | rs4622883 | A | G | 0.044 | 0.008 | 3.02E-08 | -0.014 | 0.011 | 0.201 |
|  | rs4686471 | C | T | 0.053 | 0.008 | 4.28E-11 | -0.004 | 0.011 | 0.847 |
|  | rs4812829 | A | G | 0.053 | 0.010 | 2.44E-08 | 0.020 | 0.014 | 0.168 |
|  | rs4823182 | A | G | -0.048 | 0.008 | 3.36E-10 | 0.006 | 0.011 | 0.616 |
|  | rs4865796 | A | G | 0.053 | 0.008 | 1.33E-11 | 0.013 | 0.011 | 0.218 |
|  | rs516946 | T | C | -0.082 | 0.009 | 3.16E-22 | 0.016 | 0.012 | 0.195 |
|  | rs5215 | C | T | 0.068 | 0.007 | 2.09E-20 | 0.003 | 0.011 | 0.808 |
|  | rs576674 | A | G | -0.065 | 0.010 | 1.79E-11 | 0.003 | 0.014 | 0.802 |
|  | rs6059662 | A | G | -0.045 | 0.008 | 1.51E-08 | 0.002 | 0.012 | 0.851 |
|  | rs61953351 | G | T | 0.070 | 0.009 | 1.98E-14 | 0.002 | 0.012 | 0.876 |
|  | rs622217 | C | T | -0.049 | 0.008 | 3.13E-10 | 0.016 | 0.010 | 0.121 |
|  | rs6515236 | A | C | 0.050 | 0.009 | 3.34E-08 | 0.027 | 0.012 | 0.021 |
|  | rs67232546 | C | T | -0.060 | 0.010 | 4.66E-10 | 0.035 | 0.013 | 0.007 |
|  | rs6767484 | A | G | -0.121 | 0.008 | 2.70E-56 | 0.010 | 0.011 | 0.376 |
|  | rs6785040 | C | T | -0.063 | 0.011 | 1.26E-08 | 0.008 | 0.014 | 0.534 |
|  | rs6795735 | C | T | 0.056 | 0.007 | 1.63E-14 | 0.003 | 0.011 | 0.800 |
|  | rs6878122 | A | G | -0.056 | 0.008 | 1.19E-12 | 0.008 | 0.012 | 0.516 |
|  | rs6960043 | C | T | 0.064 | 0.007 | 3.61E-19 | 0.007 | 0.010 | 0.535 |
|  | rs7144011 | G | T | -0.048 | 0.009 | 1.64E-08 | -0.004 | 0.013 | 0.749 |
|  | rs7177055 | A | G | 0.065 | 0.008 | 2.75E-16 | -0.016 | 0.011 | 0.153 |
|  | rs7240767 | C | T | 0.045 | 0.008 | 2.16E-08 | 0.000 | 0.011 | 0.992 |
|  | rs72892910 | G | T | -0.065 | 0.010 | 6.43E-11 | -0.018 | 0.014 | 0.183 |
|  | rs735949 | C | T | -0.071 | 0.011 | 1.95E-11 | -0.011 | 0.016 | 0.522 |
|  | rs753270 | C | T | 0.053 | 0.008 | 2.70E-11 | -0.009 | 0.011 | 0.396 |
|  | rs7561798 | A | G | -0.040 | 0.007 | 2.79E-08 | 0.003 | 0.011 | 0.749 |
|  | rs7572970 | A | G | -0.059 | 0.009 | 1.39E-11 | 0.006 | 0.012 | 0.628 |
|  | rs7607777 | G | T | 0.137 | 0.013 | 9.40E-28 | -0.019 | 0.017 | 0.293 |
|  | rs7674212 | G | T | 0.047 | 0.008 | 6.18E-10 | -0.001 | 0.011 | 0.967 |
|  | rs7685296 | C | T | 0.051 | 0.008 | 2.32E-10 | 0.001 | 0.012 | 0.907 |
|  | rs7729395 | C | T | -0.137 | 0.016 | 1.10E-17 | 0.019 | 0.056 | 0.751 |
|  | rs7756992 | A | G | -0.130 | 0.008 | 6.00E-62 | -0.018 | 0.011 | 0.117 |
|  | rs7786095 | A | G | 0.074 | 0.013 | 9.64E-09 | -0.015 | 0.018 | 0.396 |
|  | rs780094 | T | C | -0.069 | 0.007 | 5.16E-21 | -0.003 | 0.011 | 0.767 |
|  | rs7845219 | C | T | -0.042 | 0.007 | 4.54E-09 | 0.008 | 0.011 | 0.476 |
|  | rs7903146 | C | T | -0.306 | 0.008 | 1.00E-200 | -0.002 | 0.012 | 0.929 |
|  | rs7929543 | A | C | -0.083 | 0.014 | 2.20E-09 | -0.020 | 0.018 | 0.360 |
|  | rs7955901 | C | T | 0.044 | 0.007 | 7.16E-10 | 0.026 | 0.011 | 0.011 |
|  | rs8068804 | A | G | 0.059 | 0.008 | 4.41E-14 | 0.002 | 0.011 | 0.860 |
|  | rs8108269 | G | T | 0.064 | 0.008 | 3.11E-16 | -0.014 | 0.011 | 0.207 |
|  | rs825476 | C | T | -0.052 | 0.007 | 6.80E-13 | -0.019 | 0.011 | 0.080 |
|  | rs840967 | A | C | -0.050 | 0.008 | 5.44E-10 | 0.016 | 0.011 | 0.146 |
|  | rs849135 | A | G | -0.100 | 0.007 | 1.04E-43 | 0.010 | 0.011 | 0.322 |
|  | rs853974 | C | T | -0.060 | 0.009 | 7.86E-12 | 0.021 | 0.012 | 0.079 |
|  | rs9369425 | A | G | -0.055 | 0.009 | 1.13E-10 | -0.010 | 0.012 | 0.490 |
|  | rs9894220 | A | G | 0.059 | 0.008 | 1.52E-13 | 0.000 | 0.011 | 0.980 |
|  | rs9928094 | A | G | -0.105 | 0.007 | 3.59E-47 | 0.007 | 0.011 | 0.499 |
|  | rs993380 | A | G | 0.051 | 0.008 | 4.59E-10 | -0.008 | 0.011 | 0.474 |
|  | rs9940149 | A | G | -0.058 | 0.010 | 9.29E-10 | 0.026 | 0.013 | 0.059 |
| *Lactobacillaceae* | rs10077431 | A | C | -0.049 | 0.009 | 4.75E-08 | -0.006 | 0.021 | 0.770 |
|  | rs10087241 | A | G | -0.048 | 0.008 | 2.80E-09 | 0.005 | 0.017 | 0.661 |
|  | rs10100265 | A | C | 0.049 | 0.008 | 6.29E-10 | -0.007 | 0.017 | 0.649 |
|  | rs10114341 | C | T | -0.041 | 0.007 | 1.15E-08 | -0.035 | 0.017 | 0.034 |
|  | rs10401969 | C | T | 0.092 | 0.013 | 4.13E-12 | 0.002 | 0.032 | 0.893 |
|  | rs1050226 | G | A | -0.049 | 0.007 | 3.34E-11 | 0.002 | 0.017 | 0.932 |
|  | rs1061813 | A | G | -0.043 | 0.007 | 3.37E-09 | -0.018 | 0.017 | 0.283 |
|  | rs1063355 | T | G | -0.071 | 0.008 | 3.72E-19 | 0.022 | 0.017 | 0.193 |
|  | rs10740322 | A | G | 0.048 | 0.009 | 2.11E-08 | 0.007 | 0.018 | 0.690 |
|  | rs10811661 | C | T | -0.157 | 0.010 | 4.13E-58 | 0.036 | 0.022 | 0.099 |
|  | rs10842994 | C | T | 0.076 | 0.009 | 1.02E-16 | 0.012 | 0.021 | 0.568 |
|  | rs10974438 | A | C | -0.059 | 0.008 | 3.01E-15 | 0.007 | 0.017 | 0.678 |
|  | rs11098676 | C | T | 0.054 | 0.010 | 2.03E-08 | -0.020 | 0.021 | 0.328 |
|  | rs11107116 | G | T | -0.047 | 0.009 | 3.75E-08 | -0.011 | 0.020 | 0.571 |
|  | rs1111875 | T | C | -0.095 | 0.007 | 3.61E-39 | 0.004 | 0.018 | 0.821 |
|  | rs11257655 | C | T | -0.074 | 0.009 | 1.97E-17 | 0.001 | 0.020 | 0.941 |
|  | rs1127655 | C | T | 0.044 | 0.008 | 2.47E-08 | -0.018 | 0.017 | 0.300 |
|  | rs11708067 | A | G | 0.097 | 0.009 | 5.93E-29 | -0.008 | 0.021 | 0.657 |
|  | rs11925227 | A | G | -0.053 | 0.010 | 2.25E-08 | 0.029 | 0.022 | 0.182 |
|  | rs11926707 | C | T | 0.046 | 0.008 | 1.69E-08 | 0.023 | 0.018 | 0.191 |
|  | rs12088739 | A | G | 0.088 | 0.013 | 9.79E-12 | -0.007 | 0.029 | 0.732 |
|  | rs12299509 | A | G | -0.047 | 0.007 | 2.09E-10 | -0.023 | 0.017 | 0.178 |
|  | rs12617659 | C | T | 0.069 | 0.010 | 2.83E-11 | 0.055 | 0.024 | 0.021 |
|  | rs12910825 | A | G | -0.052 | 0.007 | 2.16E-12 | 0.022 | 0.017 | 0.223 |
|  | rs12945601 | C | T | -0.048 | 0.008 | 1.72E-09 | 0.012 | 0.017 | 0.530 |
|  | rs12970134 | A | G | 0.056 | 0.008 | 5.31E-12 | -0.011 | 0.019 | 0.584 |
|  | rs13239186 | C | T | -0.054 | 0.009 | 2.70E-10 | -0.027 | 0.018 | 0.171 |
|  | rs13330951 | A | G | 0.046 | 0.008 | 1.54E-08 | -0.031 | 0.017 | 0.071 |
|  | rs13389219 | C | T | 0.072 | 0.007 | 2.11E-22 | -0.021 | 0.018 | 0.201 |
|  | rs1359790 | G | A | 0.080 | 0.008 | 2.80E-23 | 0.017 | 0.019 | 0.399 |
|  | rs1496653 | G | A | -0.077 | 0.009 | 2.57E-18 | -0.034 | 0.021 | 0.111 |
|  | rs1552224 | C | A | -0.103 | 0.010 | 8.64E-25 | 0.006 | 0.023 | 0.769 |
|  | rs16988333 | A | G | 0.075 | 0.013 | 9.17E-09 | 0.014 | 0.033 | 0.667 |
|  | rs17086692 | G | T | 0.047 | 0.008 | 2.48E-08 | 0.006 | 0.018 | 0.780 |
|  | rs17168486 | C | T | -0.074 | 0.009 | 2.18E-15 | 0.043 | 0.022 | 0.048 |
|  | rs17405722 | A | G | 0.087 | 0.015 | 2.28E-09 | -0.032 | 0.034 | 0.307 |
|  | rs17631783 | C | T | 0.049 | 0.009 | 3.95E-08 | 0.000 | 0.019 | 0.998 |
|  | rs17791513 | A | G | 0.103 | 0.015 | 4.61E-12 | -0.083 | 0.035 | 0.016 |
|  | rs1801214 | C | T | 0.090 | 0.007 | 5.52E-34 | -0.018 | 0.017 | 0.294 |
|  | rs1899951 | T | C | -0.112 | 0.011 | 1.64E-24 | -0.010 | 0.025 | 0.664 |
|  | rs2237892 | C | T | 0.096 | 0.016 | 8.75E-10 | 0.013 | 0.036 | 0.857 |
|  | rs2246618 | C | T | -0.051 | 0.008 | 1.20E-09 | 0.009 | 0.018 | 0.665 |
|  | rs2261181 | C | T | -0.099 | 0.012 | 9.18E-17 | -0.015 | 0.028 | 0.631 |
|  | rs2294120 | G | A | -0.044 | 0.008 | 1.62E-08 | 0.018 | 0.017 | 0.319 |
|  | rs2296173 | A | G | -0.065 | 0.009 | 7.66E-14 | -0.016 | 0.021 | 0.458 |
|  | rs2299383 | C | T | -0.041 | 0.007 | 1.49E-08 | -0.009 | 0.017 | 0.604 |
|  | rs243019 | T | C | -0.057 | 0.007 | 2.29E-15 | 0.002 | 0.017 | 0.912 |
|  | rs2493394 | G | A | 0.073 | 0.011 | 1.15E-10 | 0.018 | 0.028 | 0.456 |
|  | rs2796441 | G | A | 0.072 | 0.007 | 1.96E-22 | -0.003 | 0.017 | 0.843 |
|  | rs2820426 | G | A | 0.052 | 0.007 | 1.30E-12 | -0.006 | 0.017 | 0.751 |
|  | rs2867125 | T | C | -0.060 | 0.010 | 4.33E-10 | 0.051 | 0.023 | 0.025 |
|  | rs2908282 | G | A | -0.055 | 0.009 | 4.25E-09 | 0.028 | 0.022 | 0.222 |
|  | rs2925979 | T | C | 0.053 | 0.008 | 9.06E-12 | 0.011 | 0.018 | 0.547 |
|  | rs2943656 | A | G | -0.090 | 0.007 | 6.70E-34 | 0.021 | 0.018 | 0.208 |
|  | rs3217992 | T | C | 0.053 | 0.007 | 7.23E-13 | 0.014 | 0.017 | 0.411 |
|  | rs340874 | T | C | -0.063 | 0.007 | 8.41E-18 | -0.012 | 0.017 | 0.481 |
|  | rs348330 | G | A | 0.049 | 0.008 | 1.86E-09 | -0.006 | 0.018 | 0.720 |
|  | rs3756784 | G | T | 0.051 | 0.009 | 2.59E-08 | -0.042 | 0.021 | 0.047 |
|  | rs3802177 | G | A | 0.122 | 0.008 | 2.32E-52 | -0.026 | 0.018 | 0.174 |
|  | rs459193 | G | A | 0.071 | 0.008 | 8.81E-18 | 0.000 | 0.020 | 0.973 |
|  | rs4622883 | A | G | 0.044 | 0.008 | 3.02E-08 | -0.004 | 0.017 | 0.833 |
|  | rs4686471 | C | T | 0.053 | 0.008 | 4.28E-11 | 0.023 | 0.018 | 0.230 |
|  | rs4812829 | A | G | 0.053 | 0.010 | 2.44E-08 | 0.015 | 0.022 | 0.480 |
|  | rs4823182 | A | G | -0.048 | 0.008 | 3.36E-10 | -0.003 | 0.018 | 0.886 |
|  | rs4865796 | A | G | 0.053 | 0.008 | 1.33E-11 | -0.029 | 0.018 | 0.084 |
|  | rs516946 | T | C | -0.082 | 0.009 | 3.16E-22 | 0.023 | 0.020 | 0.253 |
|  | rs5215 | C | T | 0.068 | 0.007 | 2.09E-20 | 0.000 | 0.017 | 1.000 |
|  | rs576674 | A | G | -0.065 | 0.010 | 1.79E-11 | 0.024 | 0.023 | 0.276 |
|  | rs6059662 | A | G | -0.045 | 0.008 | 1.51E-08 | 0.003 | 0.019 | 0.864 |
|  | rs61953351 | G | T | 0.070 | 0.009 | 1.98E-14 | 0.005 | 0.020 | 0.777 |
|  | rs622217 | C | T | -0.049 | 0.008 | 3.13E-10 | -0.007 | 0.017 | 0.688 |
|  | rs6515236 | A | C | 0.050 | 0.009 | 3.34E-08 | -0.010 | 0.019 | 0.654 |
|  | rs67232546 | C | T | -0.060 | 0.010 | 4.66E-10 | 0.018 | 0.021 | 0.376 |
|  | rs6767484 | A | G | -0.121 | 0.008 | 2.70E-56 | 0.007 | 0.018 | 0.729 |
|  | rs6785040 | C | T | -0.063 | 0.011 | 1.26E-08 | -0.002 | 0.022 | 0.892 |
|  | rs6795735 | C | T | 0.056 | 0.007 | 1.63E-14 | -0.001 | 0.017 | 0.953 |
|  | rs6878122 | A | G | -0.056 | 0.008 | 1.19E-12 | 0.008 | 0.019 | 0.745 |
|  | rs6960043 | C | T | 0.064 | 0.007 | 3.61E-19 | -0.014 | 0.017 | 0.390 |
|  | rs7144011 | G | T | -0.048 | 0.009 | 1.64E-08 | -0.017 | 0.021 | 0.416 |
|  | rs7177055 | A | G | 0.065 | 0.008 | 2.75E-16 | 0.012 | 0.018 | 0.506 |
|  | rs7240767 | C | T | 0.045 | 0.008 | 2.16E-08 | -0.023 | 0.017 | 0.183 |
|  | rs72892910 | G | T | -0.065 | 0.010 | 6.43E-11 | 0.001 | 0.022 | 0.953 |
|  | rs735949 | C | T | -0.071 | 0.011 | 1.95E-11 | 0.028 | 0.025 | 0.255 |
|  | rs753270 | C | T | 0.053 | 0.008 | 2.70E-11 | 0.021 | 0.017 | 0.224 |
|  | rs7561798 | A | G | -0.040 | 0.007 | 2.79E-08 | 0.011 | 0.017 | 0.502 |
|  | rs7572970 | A | G | -0.059 | 0.009 | 1.39E-11 | -0.003 | 0.019 | 0.830 |
|  | rs7607777 | G | T | 0.137 | 0.013 | 9.40E-28 | 0.005 | 0.028 | 0.931 |
|  | rs7674212 | G | T | 0.047 | 0.008 | 6.18E-10 | -0.032 | 0.017 | 0.054 |
|  | rs7685296 | C | T | 0.051 | 0.008 | 2.32E-10 | -0.001 | 0.019 | 0.961 |
|  | rs7756992 | A | G | -0.130 | 0.008 | 6.00E-62 | 0.021 | 0.018 | 0.251 |
|  | rs7786095 | A | G | 0.074 | 0.013 | 9.64E-09 | 0.037 | 0.029 | 0.211 |
|  | rs780094 | T | C | -0.069 | 0.007 | 5.16E-21 | -0.002 | 0.017 | 0.923 |
|  | rs7845219 | C | T | -0.042 | 0.007 | 4.54E-09 | -0.003 | 0.017 | 0.845 |
|  | rs7903146 | C | T | -0.306 | 0.008 | 1.00E-200 | -0.045 | 0.019 | 0.017 |
|  | rs7929543 | A | C | -0.083 | 0.014 | 2.20E-09 | -0.035 | 0.029 | 0.232 |
|  | rs7955901 | C | T | 0.044 | 0.007 | 7.16E-10 | 0.016 | 0.017 | 0.339 |
|  | rs8068804 | A | G | 0.059 | 0.008 | 4.41E-14 | 0.010 | 0.018 | 0.606 |
|  | rs8108269 | G | T | 0.064 | 0.008 | 3.11E-16 | 0.015 | 0.018 | 0.400 |
|  | rs825476 | C | T | -0.052 | 0.007 | 6.80E-13 | 0.000 | 0.017 | 0.982 |
|  | rs840967 | A | C | -0.050 | 0.008 | 5.44E-10 | -0.020 | 0.017 | 0.251 |
|  | rs849135 | A | G | -0.100 | 0.007 | 1.04E-43 | -0.014 | 0.017 | 0.415 |
|  | rs853974 | C | T | -0.060 | 0.009 | 7.86E-12 | 0.031 | 0.019 | 0.096 |
|  | rs9369425 | A | G | -0.055 | 0.009 | 1.13E-10 | 0.016 | 0.019 | 0.464 |
|  | rs9894220 | A | G | 0.059 | 0.008 | 1.52E-13 | -0.030 | 0.017 | 0.083 |
|  | rs9928094 | A | G | -0.105 | 0.007 | 3.59E-47 | -0.006 | 0.017 | 0.721 |
|  | rs993380 | A | G | 0.051 | 0.008 | 4.59E-10 | 0.013 | 0.018 | 0.449 |
|  | rs9940149 | A | G | -0.058 | 0.010 | 9.29E-10 | -0.011 | 0.022 | 0.573 |
| *Methanobacteriaceae* | rs10077431 | A | C | -0.049 | 0.009 | 4.75E-08 | -0.036 | 0.029 | 0.212 |
|  | rs10087241 | A | G | -0.048 | 0.008 | 2.80E-09 | 0.005 | 0.024 | 0.862 |
|  | rs10100265 | A | C | 0.049 | 0.008 | 6.29E-10 | 0.019 | 0.024 | 0.420 |
|  | rs10114341 | C | T | -0.041 | 0.007 | 1.15E-08 | 0.004 | 0.024 | 0.920 |
|  | rs10401969 | C | T | 0.092 | 0.013 | 4.13E-12 | 0.076 | 0.047 | 0.072 |
|  | rs1050226 | G | A | -0.049 | 0.007 | 3.34E-11 | 0.016 | 0.024 | 0.533 |
|  | rs1061813 | A | G | -0.043 | 0.007 | 3.37E-09 | 0.022 | 0.023 | 0.368 |
|  | rs1063355 | T | G | -0.071 | 0.008 | 3.72E-19 | -0.029 | 0.024 | 0.218 |
|  | rs10740322 | A | G | 0.048 | 0.009 | 2.11E-08 | 0.019 | 0.025 | 0.419 |
|  | rs10811661 | C | T | -0.157 | 0.010 | 4.13E-58 | 0.040 | 0.032 | 0.235 |
|  | rs10842994 | C | T | 0.076 | 0.009 | 1.02E-16 | -0.016 | 0.030 | 0.610 |
|  | rs10974438 | A | C | -0.059 | 0.008 | 3.01E-15 | 0.014 | 0.024 | 0.561 |
|  | rs11098676 | C | T | 0.054 | 0.010 | 2.03E-08 | -0.001 | 0.028 | 0.986 |
|  | rs11107116 | G | T | -0.047 | 0.009 | 3.75E-08 | 0.053 | 0.028 | 0.048 |
|  | rs1111875 | T | C | -0.095 | 0.007 | 3.61E-39 | 0.022 | 0.025 | 0.384 |
|  | rs11257655 | C | T | -0.074 | 0.009 | 1.97E-17 | -0.017 | 0.028 | 0.476 |
|  | rs1127655 | C | T | 0.044 | 0.008 | 2.47E-08 | -0.014 | 0.023 | 0.539 |
|  | rs11708067 | A | G | 0.097 | 0.009 | 5.93E-29 | 0.018 | 0.028 | 0.553 |
|  | rs11925227 | A | G | -0.053 | 0.010 | 2.25E-08 | 0.070 | 0.032 | 0.029 |
|  | rs11926707 | C | T | 0.046 | 0.008 | 1.69E-08 | -0.045 | 0.025 | 0.076 |
|  | rs12088739 | A | G | 0.088 | 0.013 | 9.79E-12 | -0.070 | 0.041 | 0.103 |
|  | rs12299509 | A | G | -0.047 | 0.007 | 2.09E-10 | 0.001 | 0.023 | 0.983 |
|  | rs12617659 | C | T | 0.069 | 0.010 | 2.83E-11 | 0.010 | 0.033 | 0.787 |
|  | rs12910825 | A | G | -0.052 | 0.007 | 2.16E-12 | 0.010 | 0.024 | 0.659 |
|  | rs12945601 | C | T | -0.048 | 0.008 | 1.72E-09 | 0.027 | 0.024 | 0.261 |
|  | rs12970134 | A | G | 0.056 | 0.008 | 5.31E-12 | 0.021 | 0.027 | 0.466 |
|  | rs13239186 | C | T | -0.054 | 0.009 | 2.70E-10 | -0.021 | 0.026 | 0.363 |
|  | rs13330951 | A | G | 0.046 | 0.008 | 1.54E-08 | -0.010 | 0.023 | 0.701 |
|  | rs13389219 | C | T | 0.072 | 0.007 | 2.11E-22 | 0.020 | 0.025 | 0.444 |
|  | rs1359790 | G | A | 0.080 | 0.008 | 2.80E-23 | 0.005 | 0.027 | 0.878 |
|  | rs1496653 | G | A | -0.077 | 0.009 | 2.57E-18 | 0.011 | 0.029 | 0.719 |
|  | rs1552224 | C | A | -0.103 | 0.010 | 8.64E-25 | 0.022 | 0.032 | 0.420 |
|  | rs16988333 | A | G | 0.075 | 0.013 | 9.17E-09 | 0.013 | 0.045 | 0.846 |
|  | rs17086692 | G | T | 0.047 | 0.008 | 2.48E-08 | 0.004 | 0.025 | 0.861 |
|  | rs17168486 | C | T | -0.074 | 0.009 | 2.18E-15 | 0.006 | 0.031 | 0.911 |
|  | rs17405722 | A | G | 0.087 | 0.015 | 2.28E-09 | -0.006 | 0.047 | 0.875 |
|  | rs17631783 | C | T | 0.049 | 0.009 | 3.95E-08 | -0.019 | 0.027 | 0.469 |
|  | rs17791513 | A | G | 0.103 | 0.015 | 4.61E-12 | -0.009 | 0.050 | 0.792 |
|  | rs1801214 | C | T | 0.090 | 0.007 | 5.52E-34 | -0.018 | 0.024 | 0.416 |
|  | rs1899951 | T | C | -0.112 | 0.011 | 1.64E-24 | 0.013 | 0.036 | 0.978 |
|  | rs2246618 | C | T | -0.051 | 0.008 | 1.20E-09 | 0.012 | 0.025 | 0.658 |
|  | rs2261181 | C | T | -0.099 | 0.012 | 9.18E-17 | -0.002 | 0.038 | 0.943 |
|  | rs2294120 | G | A | -0.044 | 0.008 | 1.62E-08 | 0.025 | 0.023 | 0.296 |
|  | rs2296173 | A | G | -0.065 | 0.009 | 7.66E-14 | -0.051 | 0.029 | 0.070 |
|  | rs2299383 | C | T | -0.041 | 0.007 | 1.49E-08 | 0.029 | 0.023 | 0.221 |
|  | rs243019 | T | C | -0.057 | 0.007 | 2.29E-15 | -0.012 | 0.023 | 0.611 |
|  | rs2493394 | G | A | 0.073 | 0.011 | 1.15E-10 | -0.025 | 0.038 | 0.465 |
|  | rs2796441 | G | A | 0.072 | 0.007 | 1.96E-22 | 0.004 | 0.024 | 0.914 |
|  | rs2820426 | G | A | 0.052 | 0.007 | 1.30E-12 | 0.048 | 0.024 | 0.044 |
|  | rs2867125 | T | C | -0.060 | 0.010 | 4.33E-10 | 0.023 | 0.031 | 0.483 |
|  | rs2908282 | G | A | -0.055 | 0.009 | 4.25E-09 | -0.016 | 0.031 | 0.565 |
|  | rs2925979 | T | C | 0.053 | 0.008 | 9.06E-12 | -0.001 | 0.025 | 0.965 |
|  | rs2943656 | A | G | -0.090 | 0.007 | 6.70E-34 | 0.020 | 0.024 | 0.492 |
|  | rs3217992 | T | C | 0.053 | 0.007 | 7.23E-13 | 0.044 | 0.024 | 0.069 |
|  | rs340874 | T | C | -0.063 | 0.007 | 8.41E-18 | 0.000 | 0.025 | 0.999 |
|  | rs348330 | G | A | 0.049 | 0.008 | 1.86E-09 | 0.007 | 0.026 | 0.792 |
|  | rs3756784 | G | T | 0.051 | 0.009 | 2.59E-08 | -0.047 | 0.029 | 0.106 |
|  | rs3802177 | G | A | 0.122 | 0.008 | 2.32E-52 | 0.005 | 0.025 | 0.858 |
|  | rs459193 | G | A | 0.071 | 0.008 | 8.81E-18 | 0.055 | 0.028 | 0.052 |
|  | rs4622883 | A | G | 0.044 | 0.008 | 3.02E-08 | 0.001 | 0.023 | 0.961 |
|  | rs4686471 | C | T | 0.053 | 0.008 | 4.28E-11 | 0.052 | 0.024 | 0.027 |
|  | rs4812829 | A | G | 0.053 | 0.010 | 2.44E-08 | -0.013 | 0.032 | 0.650 |
|  | rs4823182 | A | G | -0.048 | 0.008 | 3.36E-10 | -0.026 | 0.024 | 0.292 |
|  | rs4865796 | A | G | 0.053 | 0.008 | 1.33E-11 | -0.001 | 0.025 | 0.916 |
|  | rs516946 | T | C | -0.082 | 0.009 | 3.16E-22 | 0.017 | 0.027 | 0.523 |
|  | rs5215 | C | T | 0.068 | 0.007 | 2.09E-20 | -0.003 | 0.024 | 0.912 |
|  | rs576674 | A | G | -0.065 | 0.010 | 1.79E-11 | -0.017 | 0.032 | 0.633 |
|  | rs6059662 | A | G | -0.045 | 0.008 | 1.51E-08 | 0.012 | 0.027 | 0.689 |
|  | rs61953351 | G | T | 0.070 | 0.009 | 1.98E-14 | 0.038 | 0.027 | 0.164 |
|  | rs622217 | C | T | -0.049 | 0.008 | 3.13E-10 | -0.001 | 0.023 | 0.959 |
|  | rs6515236 | A | C | 0.050 | 0.009 | 3.34E-08 | 0.001 | 0.027 | 0.983 |
|  | rs67232546 | C | T | -0.060 | 0.010 | 4.66E-10 | -0.018 | 0.030 | 0.522 |
|  | rs6767484 | A | G | -0.121 | 0.008 | 2.70E-56 | -0.014 | 0.025 | 0.581 |
|  | rs6785040 | C | T | -0.063 | 0.011 | 1.26E-08 | 0.010 | 0.031 | 0.686 |
|  | rs6795735 | C | T | 0.056 | 0.007 | 1.63E-14 | 0.012 | 0.024 | 0.611 |
|  | rs6878122 | A | G | -0.056 | 0.008 | 1.19E-12 | -0.008 | 0.026 | 0.703 |
|  | rs6960043 | C | T | 0.064 | 0.007 | 3.61E-19 | -0.036 | 0.023 | 0.125 |
|  | rs7144011 | G | T | -0.048 | 0.009 | 1.64E-08 | -0.014 | 0.029 | 0.601 |
|  | rs7177055 | A | G | 0.065 | 0.008 | 2.75E-16 | 0.023 | 0.026 | 0.374 |
|  | rs7240767 | C | T | 0.045 | 0.008 | 2.16E-08 | 0.011 | 0.024 | 0.634 |
|  | rs72892910 | G | T | -0.065 | 0.010 | 6.43E-11 | -0.056 | 0.031 | 0.073 |
|  | rs735949 | C | T | -0.071 | 0.011 | 1.95E-11 | 0.072 | 0.034 | 0.043 |
|  | rs753270 | C | T | 0.053 | 0.008 | 2.70E-11 | 0.051 | 0.024 | 0.030 |
|  | rs7561798 | A | G | -0.040 | 0.007 | 2.79E-08 | -0.031 | 0.023 | 0.187 |
|  | rs7572970 | A | G | -0.059 | 0.009 | 1.39E-11 | -0.036 | 0.026 | 0.141 |
|  | rs7607777 | G | T | 0.137 | 0.013 | 9.40E-28 | 0.001 | 0.038 | 0.957 |
|  | rs7674212 | G | T | 0.047 | 0.008 | 6.18E-10 | 0.015 | 0.024 | 0.533 |
|  | rs7685296 | C | T | 0.051 | 0.008 | 2.32E-10 | 0.006 | 0.026 | 0.828 |
|  | rs7756992 | A | G | -0.130 | 0.008 | 6.00E-62 | 0.023 | 0.026 | 0.353 |
|  | rs7786095 | A | G | 0.074 | 0.013 | 9.64E-09 | -0.026 | 0.039 | 0.520 |
|  | rs780094 | T | C | -0.069 | 0.007 | 5.16E-21 | 0.010 | 0.024 | 0.682 |
|  | rs7845219 | C | T | -0.042 | 0.007 | 4.54E-09 | -0.001 | 0.023 | 0.998 |
|  | rs7903146 | C | T | -0.306 | 0.008 | 1.00E-200 | 0.020 | 0.026 | 0.450 |
|  | rs7929543 | A | C | -0.083 | 0.014 | 2.20E-09 | 0.099 | 0.040 | 0.017 |
|  | rs7955901 | C | T | 0.044 | 0.007 | 7.16E-10 | 0.012 | 0.023 | 0.625 |
|  | rs8068804 | A | G | 0.059 | 0.008 | 4.41E-14 | 0.042 | 0.025 | 0.103 |
|  | rs8108269 | G | T | 0.064 | 0.008 | 3.11E-16 | -0.036 | 0.025 | 0.170 |
|  | rs825476 | C | T | -0.052 | 0.007 | 6.80E-13 | -0.009 | 0.024 | 0.713 |
|  | rs840967 | A | C | -0.050 | 0.008 | 5.44E-10 | 0.011 | 0.024 | 0.631 |
|  | rs849135 | A | G | -0.100 | 0.007 | 1.04E-43 | 0.012 | 0.023 | 0.607 |
|  | rs853974 | C | T | -0.060 | 0.009 | 7.86E-12 | -0.006 | 0.026 | 0.810 |
|  | rs9369425 | A | G | -0.055 | 0.009 | 1.13E-10 | -0.010 | 0.026 | 0.716 |
|  | rs9894220 | A | G | 0.059 | 0.008 | 1.52E-13 | -0.041 | 0.024 | 0.082 |
|  | rs9928094 | A | G | -0.105 | 0.007 | 3.59E-47 | -0.046 | 0.023 | 0.050 |
|  | rs993380 | A | G | 0.051 | 0.008 | 4.59E-10 | 0.006 | 0.024 | 0.800 |
|  | rs9940149 | A | G | -0.058 | 0.010 | 9.29E-10 | 0.032 | 0.030 | 0.251 |
| *Oxalobacteraceae* | rs10077431 | A | C | -0.049 | 0.009 | 4.75E-08 | -0.010 | 0.024 | 0.672 |
|  | rs10087241 | A | G | -0.048 | 0.008 | 2.80E-09 | 0.017 | 0.020 | 0.421 |
|  | rs10100265 | A | C | 0.049 | 0.008 | 6.29E-10 | -0.018 | 0.020 | 0.367 |
|  | rs10114341 | C | T | -0.041 | 0.007 | 1.15E-08 | -0.025 | 0.020 | 0.233 |
|  | rs10401969 | C | T | 0.092 | 0.013 | 4.13E-12 | -0.070 | 0.037 | 0.056 |
|  | rs1050226 | G | A | -0.049 | 0.007 | 3.34E-11 | 0.025 | 0.020 | 0.217 |
|  | rs1061813 | A | G | -0.043 | 0.007 | 3.37E-09 | -0.013 | 0.020 | 0.548 |
|  | rs1063355 | T | G | -0.071 | 0.008 | 3.72E-19 | 0.006 | 0.020 | 0.748 |
|  | rs10740322 | A | G | 0.048 | 0.009 | 2.11E-08 | 0.018 | 0.021 | 0.415 |
|  | rs10811661 | C | T | -0.157 | 0.010 | 4.13E-58 | 0.049 | 0.026 | 0.060 |
|  | rs10842994 | C | T | 0.076 | 0.009 | 1.02E-16 | 0.043 | 0.024 | 0.079 |
|  | rs10974438 | A | C | -0.059 | 0.008 | 3.01E-15 | -0.022 | 0.020 | 0.287 |
|  | rs11098676 | C | T | 0.054 | 0.010 | 2.03E-08 | -0.001 | 0.024 | 0.969 |
|  | rs11107116 | G | T | -0.047 | 0.009 | 3.75E-08 | 0.022 | 0.024 | 0.353 |
|  | rs1111875 | T | C | -0.095 | 0.007 | 3.61E-39 | 0.012 | 0.020 | 0.569 |
|  | rs11257655 | C | T | -0.074 | 0.009 | 1.97E-17 | 0.034 | 0.024 | 0.167 |
|  | rs1127655 | C | T | 0.044 | 0.008 | 2.47E-08 | 0.024 | 0.020 | 0.223 |
|  | rs11708067 | A | G | 0.097 | 0.009 | 5.93E-29 | -0.009 | 0.024 | 0.726 |
|  | rs11925227 | A | G | -0.053 | 0.010 | 2.25E-08 | -0.010 | 0.026 | 0.676 |
|  | rs11926707 | C | T | 0.046 | 0.008 | 1.69E-08 | -0.001 | 0.021 | 0.982 |
|  | rs12088739 | A | G | 0.088 | 0.013 | 9.79E-12 | -0.040 | 0.034 | 0.281 |
|  | rs12299509 | A | G | -0.047 | 0.007 | 2.09E-10 | 0.003 | 0.020 | 0.849 |
|  | rs12617659 | C | T | 0.069 | 0.010 | 2.83E-11 | -0.005 | 0.028 | 0.828 |
|  | rs12910825 | A | G | -0.052 | 0.007 | 2.16E-12 | -0.033 | 0.020 | 0.127 |
|  | rs12945601 | C | T | -0.048 | 0.008 | 1.72E-09 | 0.014 | 0.020 | 0.561 |
|  | rs12970134 | A | G | 0.056 | 0.008 | 5.31E-12 | -0.010 | 0.022 | 0.644 |
|  | rs13239186 | C | T | -0.054 | 0.009 | 2.70E-10 | -0.008 | 0.021 | 0.670 |
|  | rs13330951 | A | G | 0.046 | 0.008 | 1.54E-08 | -0.009 | 0.020 | 0.646 |
|  | rs13389219 | C | T | 0.072 | 0.007 | 2.11E-22 | -0.018 | 0.021 | 0.346 |
|  | rs1359790 | G | A | 0.080 | 0.008 | 2.80E-23 | -0.011 | 0.022 | 0.647 |
|  | rs1496653 | G | A | -0.077 | 0.009 | 2.57E-18 | 0.016 | 0.024 | 0.561 |
|  | rs1552224 | C | A | -0.103 | 0.010 | 8.64E-25 | 0.047 | 0.027 | 0.068 |
|  | rs16988333 | A | G | 0.075 | 0.013 | 9.17E-09 | -0.049 | 0.037 | 0.181 |
|  | rs17086692 | G | T | 0.047 | 0.008 | 2.48E-08 | -0.038 | 0.021 | 0.076 |
|  | rs17168486 | C | T | -0.074 | 0.009 | 2.18E-15 | -0.032 | 0.026 | 0.210 |
|  | rs17405722 | A | G | 0.087 | 0.015 | 2.28E-09 | -0.007 | 0.038 | 0.831 |
|  | rs17631783 | C | T | 0.049 | 0.009 | 3.95E-08 | -0.031 | 0.022 | 0.151 |
|  | rs17791513 | A | G | 0.103 | 0.015 | 4.61E-12 | 0.067 | 0.041 | 0.081 |
|  | rs1801214 | C | T | 0.090 | 0.007 | 5.52E-34 | -0.008 | 0.020 | 0.679 |
|  | rs1899951 | T | C | -0.112 | 0.011 | 1.64E-24 | 0.077 | 0.029 | 0.009 |
|  | rs2237892 | C | T | 0.096 | 0.016 | 8.75E-10 | 0.029 | 0.044 | 0.912 |
|  | rs2246618 | C | T | -0.051 | 0.008 | 1.20E-09 | -0.013 | 0.021 | 0.571 |
|  | rs2261181 | C | T | -0.099 | 0.012 | 9.18E-17 | 0.054 | 0.033 | 0.112 |
|  | rs2294120 | G | A | -0.044 | 0.008 | 1.62E-08 | 0.018 | 0.020 | 0.339 |
|  | rs2296173 | A | G | -0.065 | 0.009 | 7.66E-14 | 0.004 | 0.024 | 0.926 |
|  | rs2299383 | C | T | -0.041 | 0.007 | 1.49E-08 | 0.006 | 0.020 | 0.752 |
|  | rs243019 | T | C | -0.057 | 0.007 | 2.29E-15 | 0.001 | 0.020 | 0.971 |
|  | rs2493394 | G | A | 0.073 | 0.011 | 1.15E-10 | -0.005 | 0.032 | 0.903 |
|  | rs2796441 | G | A | 0.072 | 0.007 | 1.96E-22 | -0.017 | 0.020 | 0.419 |
|  | rs2820426 | G | A | 0.052 | 0.007 | 1.30E-12 | 0.024 | 0.020 | 0.259 |
|  | rs2867125 | T | C | -0.060 | 0.010 | 4.33E-10 | -0.015 | 0.026 | 0.590 |
|  | rs2908282 | G | A | -0.055 | 0.009 | 4.25E-09 | 0.001 | 0.026 | 0.960 |
|  | rs2925979 | T | C | 0.053 | 0.008 | 9.06E-12 | -0.008 | 0.021 | 0.714 |
|  | rs2943656 | A | G | -0.090 | 0.007 | 6.70E-34 | -0.015 | 0.021 | 0.488 |
|  | rs3217992 | T | C | 0.053 | 0.007 | 7.23E-13 | 0.010 | 0.020 | 0.658 |
|  | rs340874 | T | C | -0.063 | 0.007 | 8.41E-18 | -0.013 | 0.020 | 0.508 |
|  | rs348330 | G | A | 0.049 | 0.008 | 1.86E-09 | -0.031 | 0.021 | 0.137 |
|  | rs3756784 | G | T | 0.051 | 0.009 | 2.59E-08 | 0.013 | 0.024 | 0.534 |
|  | rs3802177 | G | A | 0.122 | 0.008 | 2.32E-52 | -0.039 | 0.021 | 0.070 |
|  | rs459193 | G | A | 0.071 | 0.008 | 8.81E-18 | 0.008 | 0.023 | 0.759 |
|  | rs4622883 | A | G | 0.044 | 0.008 | 3.02E-08 | -0.024 | 0.020 | 0.219 |
|  | rs4686471 | C | T | 0.053 | 0.008 | 4.28E-11 | -0.024 | 0.020 | 0.309 |
|  | rs4812829 | A | G | 0.053 | 0.010 | 2.44E-08 | -0.056 | 0.026 | 0.026 |
|  | rs4823182 | A | G | -0.048 | 0.008 | 3.36E-10 | 0.003 | 0.021 | 0.841 |
|  | rs4865796 | A | G | 0.053 | 0.008 | 1.33E-11 | -0.026 | 0.021 | 0.189 |
|  | rs516946 | T | C | -0.082 | 0.009 | 3.16E-22 | -0.012 | 0.023 | 0.542 |
|  | rs5215 | C | T | 0.068 | 0.007 | 2.09E-20 | -0.017 | 0.020 | 0.435 |
|  | rs576674 | A | G | -0.065 | 0.010 | 1.79E-11 | 0.029 | 0.026 | 0.314 |
|  | rs6059662 | A | G | -0.045 | 0.008 | 1.51E-08 | -0.008 | 0.022 | 0.697 |
|  | rs61953351 | G | T | 0.070 | 0.009 | 1.98E-14 | 0.011 | 0.023 | 0.550 |
|  | rs622217 | C | T | -0.049 | 0.008 | 3.13E-10 | -0.035 | 0.020 | 0.076 |
|  | rs6515236 | A | C | 0.050 | 0.009 | 3.34E-08 | -0.007 | 0.023 | 0.759 |
|  | rs67232546 | C | T | -0.060 | 0.010 | 4.66E-10 | 0.005 | 0.025 | 0.816 |
|  | rs6767484 | A | G | -0.121 | 0.008 | 2.70E-56 | 0.004 | 0.021 | 0.832 |
|  | rs6785040 | C | T | -0.063 | 0.011 | 1.26E-08 | -0.032 | 0.026 | 0.216 |
|  | rs6795735 | C | T | 0.056 | 0.007 | 1.63E-14 | -0.011 | 0.020 | 0.594 |
|  | rs6878122 | A | G | -0.056 | 0.008 | 1.19E-12 | 0.026 | 0.022 | 0.242 |
|  | rs6960043 | C | T | 0.064 | 0.007 | 3.61E-19 | -0.002 | 0.020 | 0.902 |
|  | rs7144011 | G | T | -0.048 | 0.009 | 1.64E-08 | 0.027 | 0.025 | 0.266 |
|  | rs7177055 | A | G | 0.065 | 0.008 | 2.75E-16 | 0.002 | 0.022 | 0.897 |
|  | rs7240767 | C | T | 0.045 | 0.008 | 2.16E-08 | -0.007 | 0.020 | 0.754 |
|  | rs72892910 | G | T | -0.065 | 0.010 | 6.43E-11 | 0.028 | 0.026 | 0.273 |
|  | rs735949 | C | T | -0.071 | 0.011 | 1.95E-11 | 0.040 | 0.029 | 0.159 |
|  | rs753270 | C | T | 0.053 | 0.008 | 2.70E-11 | 0.031 | 0.020 | 0.122 |
|  | rs7561798 | A | G | -0.040 | 0.007 | 2.79E-08 | -0.011 | 0.020 | 0.578 |
|  | rs7572970 | A | G | -0.059 | 0.009 | 1.39E-11 | -0.029 | 0.022 | 0.185 |
|  | rs7607777 | G | T | 0.137 | 0.013 | 9.40E-28 | -0.025 | 0.032 | 0.375 |
|  | rs7674212 | G | T | 0.047 | 0.008 | 6.18E-10 | -0.026 | 0.020 | 0.188 |
|  | rs7685296 | C | T | 0.051 | 0.008 | 2.32E-10 | -0.014 | 0.022 | 0.520 |
|  | rs7756992 | A | G | -0.130 | 0.008 | 6.00E-62 | 0.031 | 0.022 | 0.151 |
|  | rs7786095 | A | G | 0.074 | 0.013 | 9.64E-09 | -0.018 | 0.033 | 0.590 |
|  | rs780094 | T | C | -0.069 | 0.007 | 5.16E-21 | 0.014 | 0.020 | 0.499 |
|  | rs7845219 | C | T | -0.042 | 0.007 | 4.54E-09 | -0.003 | 0.020 | 0.863 |
|  | rs7903146 | C | T | -0.306 | 0.008 | 1.00E-200 | 0.004 | 0.022 | 0.838 |
|  | rs7929543 | A | C | -0.083 | 0.014 | 2.20E-09 | 0.013 | 0.034 | 0.680 |
|  | rs7955901 | C | T | 0.044 | 0.007 | 7.16E-10 | 0.014 | 0.020 | 0.475 |
|  | rs8068804 | A | G | 0.059 | 0.008 | 4.41E-14 | -0.017 | 0.021 | 0.427 |
|  | rs8108269 | G | T | 0.064 | 0.008 | 3.11E-16 | 0.026 | 0.021 | 0.258 |
|  | rs825476 | C | T | -0.052 | 0.007 | 6.80E-13 | -0.023 | 0.020 | 0.245 |
|  | rs840967 | A | C | -0.050 | 0.008 | 5.44E-10 | 0.001 | 0.020 | 0.949 |
|  | rs849135 | A | G | -0.100 | 0.007 | 1.04E-43 | -0.042 | 0.020 | 0.034 |
|  | rs853974 | C | T | -0.060 | 0.009 | 7.86E-12 | -0.058 | 0.022 | 0.007 |
|  | rs9369425 | A | G | -0.055 | 0.009 | 1.13E-10 | 0.018 | 0.022 | 0.452 |
|  | rs9894220 | A | G | 0.059 | 0.008 | 1.52E-13 | 0.009 | 0.020 | 0.634 |
|  | rs9928094 | A | G | -0.105 | 0.007 | 3.59E-47 | 0.026 | 0.020 | 0.191 |
|  | rs993380 | A | G | 0.051 | 0.008 | 4.59E-10 | 0.023 | 0.021 | 0.265 |
|  | rs9940149 | A | G | -0.058 | 0.010 | 9.29E-10 | -0.017 | 0.025 | 0.468 |
| *Pasteurellaceae* | rs10077431 | A | C | -0.049 | 0.009 | 4.75E-08 | -0.008 | 0.018 | 0.744 |
|  | rs10087241 | A | G | -0.048 | 0.008 | 2.80E-09 | -0.007 | 0.015 | 0.668 |
|  | rs10100265 | A | C | 0.049 | 0.008 | 6.29E-10 | -0.022 | 0.015 | 0.146 |
|  | rs10114341 | C | T | -0.041 | 0.007 | 1.15E-08 | 0.003 | 0.015 | 0.880 |
|  | rs10401969 | C | T | 0.092 | 0.013 | 4.13E-12 | -0.011 | 0.028 | 0.753 |
|  | rs1050226 | G | A | -0.049 | 0.007 | 3.34E-11 | 0.014 | 0.015 | 0.338 |
|  | rs1061813 | A | G | -0.043 | 0.007 | 3.37E-09 | 0.007 | 0.015 | 0.648 |
|  | rs1063355 | T | G | -0.071 | 0.008 | 3.72E-19 | 0.026 | 0.015 | 0.084 |
|  | rs10740322 | A | G | 0.048 | 0.009 | 2.11E-08 | -0.002 | 0.016 | 0.891 |
|  | rs10811661 | C | T | -0.157 | 0.010 | 4.13E-58 | 0.019 | 0.019 | 0.369 |
|  | rs10842994 | C | T | 0.076 | 0.009 | 1.02E-16 | 0.000 | 0.018 | 0.948 |
|  | rs10974438 | A | C | -0.059 | 0.008 | 3.01E-15 | 0.000 | 0.015 | 0.971 |
|  | rs11098676 | C | T | 0.054 | 0.010 | 2.03E-08 | -0.006 | 0.018 | 0.871 |
|  | rs11107116 | G | T | -0.047 | 0.009 | 3.75E-08 | 0.025 | 0.018 | 0.134 |
|  | rs1111875 | T | C | -0.095 | 0.007 | 3.61E-39 | -0.008 | 0.015 | 0.567 |
|  | rs11257655 | C | T | -0.074 | 0.009 | 1.97E-17 | 0.006 | 0.018 | 0.695 |
|  | rs1127655 | C | T | 0.044 | 0.008 | 2.47E-08 | -0.015 | 0.015 | 0.304 |
|  | rs11708067 | A | G | 0.097 | 0.009 | 5.93E-29 | -0.003 | 0.018 | 0.828 |
|  | rs11925227 | A | G | -0.053 | 0.010 | 2.25E-08 | 0.042 | 0.019 | 0.032 |
|  | rs11926707 | C | T | 0.046 | 0.008 | 1.69E-08 | 0.012 | 0.015 | 0.421 |
|  | rs12088739 | A | G | 0.088 | 0.013 | 9.79E-12 | 0.011 | 0.026 | 0.603 |
|  | rs12299509 | A | G | -0.047 | 0.007 | 2.09E-10 | 0.013 | 0.015 | 0.394 |
|  | rs12617659 | C | T | 0.069 | 0.010 | 2.83E-11 | -0.001 | 0.021 | 0.912 |
|  | rs12910825 | A | G | -0.052 | 0.007 | 2.16E-12 | 0.041 | 0.015 | 0.006 |
|  | rs12945601 | C | T | -0.048 | 0.008 | 1.72E-09 | 0.025 | 0.015 | 0.109 |
|  | rs12970134 | A | G | 0.056 | 0.008 | 5.31E-12 | -0.024 | 0.017 | 0.139 |
|  | rs13239186 | C | T | -0.054 | 0.009 | 2.70E-10 | 0.009 | 0.016 | 0.590 |
|  | rs13330951 | A | G | 0.046 | 0.008 | 1.54E-08 | 0.027 | 0.015 | 0.071 |
|  | rs13389219 | C | T | 0.072 | 0.007 | 2.11E-22 | -0.009 | 0.015 | 0.570 |
|  | rs1359790 | G | A | 0.080 | 0.008 | 2.80E-23 | 0.000 | 0.016 | 0.956 |
|  | rs1496653 | G | A | -0.077 | 0.009 | 2.57E-18 | 0.011 | 0.018 | 0.560 |
|  | rs1552224 | C | A | -0.103 | 0.010 | 8.64E-25 | 0.027 | 0.021 | 0.182 |
|  | rs16988333 | A | G | 0.075 | 0.013 | 9.17E-09 | -0.011 | 0.029 | 0.754 |
|  | rs17086692 | G | T | 0.047 | 0.008 | 2.48E-08 | -0.013 | 0.016 | 0.388 |
|  | rs17168486 | C | T | -0.074 | 0.009 | 2.18E-15 | -0.007 | 0.019 | 0.736 |
|  | rs17405722 | A | G | 0.087 | 0.015 | 2.28E-09 | 0.048 | 0.029 | 0.112 |
|  | rs17631783 | C | T | 0.049 | 0.009 | 3.95E-08 | 0.011 | 0.017 | 0.482 |
|  | rs17791513 | A | G | 0.103 | 0.015 | 4.61E-12 | 0.013 | 0.030 | 0.608 |
|  | rs1801214 | C | T | 0.090 | 0.007 | 5.52E-34 | 0.019 | 0.015 | 0.174 |
|  | rs1899951 | T | C | -0.112 | 0.011 | 1.64E-24 | -0.004 | 0.021 | 0.978 |
|  | rs2237892 | C | T | 0.096 | 0.016 | 8.75E-10 | -0.004 | 0.028 | 0.913 |
|  | rs2246618 | C | T | -0.051 | 0.008 | 1.20E-09 | -0.004 | 0.016 | 0.871 |
|  | rs2261181 | C | T | -0.099 | 0.012 | 9.18E-17 | 0.017 | 0.024 | 0.395 |
|  | rs2294120 | G | A | -0.044 | 0.008 | 1.62E-08 | -0.013 | 0.015 | 0.394 |
|  | rs2296173 | A | G | -0.065 | 0.009 | 7.66E-14 | -0.022 | 0.018 | 0.229 |
|  | rs2299383 | C | T | -0.041 | 0.007 | 1.49E-08 | 0.001 | 0.015 | 0.942 |
|  | rs243019 | T | C | -0.057 | 0.007 | 2.29E-15 | -0.001 | 0.015 | 0.910 |
|  | rs2493394 | G | A | 0.073 | 0.011 | 1.15E-10 | -0.004 | 0.024 | 0.911 |
|  | rs2796441 | G | A | 0.072 | 0.007 | 1.96E-22 | 0.036 | 0.015 | 0.015 |
|  | rs2820426 | G | A | 0.052 | 0.007 | 1.30E-12 | 0.017 | 0.015 | 0.239 |
|  | rs2867125 | T | C | -0.060 | 0.010 | 4.33E-10 | 0.014 | 0.020 | 0.487 |
|  | rs2908282 | G | A | -0.055 | 0.009 | 4.25E-09 | -0.016 | 0.019 | 0.398 |
|  | rs2925979 | T | C | 0.053 | 0.008 | 9.06E-12 | -0.039 | 0.016 | 0.017 |
|  | rs2943656 | A | G | -0.090 | 0.007 | 6.70E-34 | -0.002 | 0.015 | 0.911 |
|  | rs3217992 | T | C | 0.053 | 0.007 | 7.23E-13 | -0.008 | 0.015 | 0.638 |
|  | rs340874 | T | C | -0.063 | 0.007 | 8.41E-18 | 0.005 | 0.015 | 0.727 |
|  | rs348330 | G | A | 0.049 | 0.008 | 1.86E-09 | 0.006 | 0.015 | 0.718 |
|  | rs3756784 | G | T | 0.051 | 0.009 | 2.59E-08 | 0.011 | 0.018 | 0.490 |
|  | rs3802177 | G | A | 0.122 | 0.008 | 2.32E-52 | 0.015 | 0.016 | 0.339 |
|  | rs459193 | G | A | 0.071 | 0.008 | 8.81E-18 | 0.029 | 0.017 | 0.084 |
|  | rs4622883 | A | G | 0.044 | 0.008 | 3.02E-08 | 0.009 | 0.015 | 0.529 |
|  | rs4686471 | C | T | 0.053 | 0.008 | 4.28E-11 | -0.001 | 0.015 | 0.980 |
|  | rs4812829 | A | G | 0.053 | 0.010 | 2.44E-08 | 0.025 | 0.019 | 0.124 |
|  | rs4823182 | A | G | -0.048 | 0.008 | 3.36E-10 | -0.028 | 0.015 | 0.063 |
|  | rs4865796 | A | G | 0.053 | 0.008 | 1.33E-11 | 0.022 | 0.016 | 0.223 |
|  | rs516946 | T | C | -0.082 | 0.009 | 3.16E-22 | -0.031 | 0.017 | 0.090 |
|  | rs5215 | C | T | 0.068 | 0.007 | 2.09E-20 | 0.000 | 0.015 | 0.949 |
|  | rs576674 | A | G | -0.065 | 0.010 | 1.79E-11 | -0.004 | 0.019 | 0.768 |
|  | rs6059662 | A | G | -0.045 | 0.008 | 1.51E-08 | -0.015 | 0.016 | 0.388 |
|  | rs61953351 | G | T | 0.070 | 0.009 | 1.98E-14 | -0.018 | 0.017 | 0.286 |
|  | rs622217 | C | T | -0.049 | 0.008 | 3.13E-10 | -0.005 | 0.015 | 0.748 |
|  | rs6515236 | A | C | 0.050 | 0.009 | 3.34E-08 | 0.012 | 0.017 | 0.413 |
|  | rs67232546 | C | T | -0.060 | 0.010 | 4.66E-10 | 0.005 | 0.019 | 0.754 |
|  | rs6767484 | A | G | -0.121 | 0.008 | 2.70E-56 | -0.019 | 0.016 | 0.244 |
|  | rs6785040 | C | T | -0.063 | 0.011 | 1.26E-08 | -0.022 | 0.020 | 0.208 |
|  | rs6795735 | C | T | 0.056 | 0.007 | 1.63E-14 | -0.001 | 0.015 | 0.955 |
|  | rs6878122 | A | G | -0.056 | 0.008 | 1.19E-12 | 0.002 | 0.016 | 0.842 |
|  | rs6960043 | C | T | 0.064 | 0.007 | 3.61E-19 | 0.010 | 0.015 | 0.500 |
|  | rs7144011 | G | T | -0.048 | 0.009 | 1.64E-08 | -0.014 | 0.018 | 0.440 |
|  | rs7177055 | A | G | 0.065 | 0.008 | 2.75E-16 | 0.018 | 0.016 | 0.262 |
|  | rs7240767 | C | T | 0.045 | 0.008 | 2.16E-08 | -0.003 | 0.015 | 0.836 |
|  | rs72892910 | G | T | -0.065 | 0.010 | 6.43E-11 | 0.009 | 0.019 | 0.598 |
|  | rs735949 | C | T | -0.071 | 0.011 | 1.95E-11 | 0.013 | 0.022 | 0.574 |
|  | rs753270 | C | T | 0.053 | 0.008 | 2.70E-11 | 0.004 | 0.015 | 0.795 |
|  | rs7561798 | A | G | -0.040 | 0.007 | 2.79E-08 | -0.012 | 0.015 | 0.421 |
|  | rs7572970 | A | G | -0.059 | 0.009 | 1.39E-11 | 0.002 | 0.017 | 0.886 |
|  | rs7607777 | G | T | 0.137 | 0.013 | 9.40E-28 | 0.009 | 0.024 | 0.657 |
|  | rs7674212 | G | T | 0.047 | 0.008 | 6.18E-10 | -0.009 | 0.015 | 0.543 |
|  | rs7685296 | C | T | 0.051 | 0.008 | 2.32E-10 | -0.028 | 0.016 | 0.081 |
|  | rs7756992 | A | G | -0.130 | 0.008 | 6.00E-62 | 0.002 | 0.016 | 0.917 |
|  | rs7786095 | A | G | 0.074 | 0.013 | 9.64E-09 | 0.006 | 0.025 | 0.879 |
|  | rs780094 | T | C | -0.069 | 0.007 | 5.16E-21 | -0.013 | 0.015 | 0.359 |
|  | rs7845219 | C | T | -0.042 | 0.007 | 4.54E-09 | -0.031 | 0.015 | 0.037 |
|  | rs7903146 | C | T | -0.306 | 0.008 | 1.00E-200 | 0.004 | 0.016 | 0.804 |
|  | rs7929543 | A | C | -0.083 | 0.014 | 2.20E-09 | 0.005 | 0.025 | 0.745 |
|  | rs7955901 | C | T | 0.044 | 0.007 | 7.16E-10 | 0.015 | 0.015 | 0.311 |
|  | rs8068804 | A | G | 0.059 | 0.008 | 4.41E-14 | -0.026 | 0.016 | 0.102 |
|  | rs8108269 | G | T | 0.064 | 0.008 | 3.11E-16 | 0.003 | 0.016 | 0.846 |
|  | rs825476 | C | T | -0.052 | 0.007 | 6.80E-13 | -0.002 | 0.015 | 0.907 |
|  | rs840967 | A | C | -0.050 | 0.008 | 5.44E-10 | -0.005 | 0.015 | 0.829 |
|  | rs849135 | A | G | -0.100 | 0.007 | 1.04E-43 | -0.013 | 0.015 | 0.391 |
|  | rs853974 | C | T | -0.060 | 0.009 | 7.86E-12 | 0.009 | 0.016 | 0.589 |
|  | rs9369425 | A | G | -0.055 | 0.009 | 1.13E-10 | 0.007 | 0.017 | 0.708 |
|  | rs9894220 | A | G | 0.059 | 0.008 | 1.52E-13 | -0.003 | 0.015 | 0.868 |
|  | rs9928094 | A | G | -0.105 | 0.007 | 3.59E-47 | 0.018 | 0.015 | 0.224 |
|  | rs993380 | A | G | 0.051 | 0.008 | 4.59E-10 | 0.018 | 0.015 | 0.228 |
|  | rs9940149 | A | G | -0.058 | 0.010 | 9.29E-10 | -0.011 | 0.018 | 0.548 |
| *Peptococcaceae* | rs10077431 | A | C | -0.049 | 0.009 | 4.75E-08 | -0.024 | 0.017 | 0.191 |
|  | rs10087241 | A | G | -0.048 | 0.008 | 2.80E-09 | -0.007 | 0.014 | 0.652 |
|  | rs10100265 | A | C | 0.049 | 0.008 | 6.29E-10 | 0.017 | 0.014 | 0.213 |
|  | rs10114341 | C | T | -0.041 | 0.007 | 1.15E-08 | 0.009 | 0.014 | 0.528 |
|  | rs10401969 | C | T | 0.092 | 0.013 | 4.13E-12 | 0.044 | 0.026 | 0.074 |
|  | rs1050226 | G | A | -0.049 | 0.007 | 3.34E-11 | -0.021 | 0.014 | 0.131 |
|  | rs1061813 | A | G | -0.043 | 0.007 | 3.37E-09 | 0.029 | 0.014 | 0.038 |
|  | rs1063355 | T | G | -0.071 | 0.008 | 3.72E-19 | 0.019 | 0.014 | 0.173 |
|  | rs10740322 | A | G | 0.048 | 0.009 | 2.11E-08 | -0.007 | 0.015 | 0.659 |
|  | rs10811661 | C | T | -0.157 | 0.010 | 4.13E-58 | 0.026 | 0.018 | 0.134 |
|  | rs10842994 | C | T | 0.076 | 0.009 | 1.02E-16 | -0.018 | 0.017 | 0.307 |
|  | rs10974438 | A | C | -0.059 | 0.008 | 3.01E-15 | -0.028 | 0.014 | 0.061 |
|  | rs11098676 | C | T | 0.054 | 0.010 | 2.03E-08 | -0.021 | 0.017 | 0.189 |
|  | rs11107116 | G | T | -0.047 | 0.009 | 3.75E-08 | 0.005 | 0.016 | 0.818 |
|  | rs1111875 | T | C | -0.095 | 0.007 | 3.61E-39 | -0.001 | 0.014 | 0.930 |
|  | rs11257655 | C | T | -0.074 | 0.009 | 1.97E-17 | -0.026 | 0.017 | 0.116 |
|  | rs1127655 | C | T | 0.044 | 0.008 | 2.47E-08 | -0.001 | 0.014 | 0.939 |
|  | rs11708067 | A | G | 0.097 | 0.009 | 5.93E-29 | -0.010 | 0.016 | 0.539 |
|  | rs11925227 | A | G | -0.053 | 0.010 | 2.25E-08 | 0.036 | 0.018 | 0.047 |
|  | rs11926707 | C | T | 0.046 | 0.008 | 1.69E-08 | -0.018 | 0.014 | 0.208 |
|  | rs12088739 | A | G | 0.088 | 0.013 | 9.79E-12 | -0.009 | 0.024 | 0.699 |
|  | rs12299509 | A | G | -0.047 | 0.007 | 2.09E-10 | -0.003 | 0.014 | 0.858 |
|  | rs12617659 | C | T | 0.069 | 0.010 | 2.83E-11 | 0.018 | 0.019 | 0.366 |
|  | rs12910825 | A | G | -0.052 | 0.007 | 2.16E-12 | -0.025 | 0.014 | 0.066 |
|  | rs12945601 | C | T | -0.048 | 0.008 | 1.72E-09 | 0.011 | 0.014 | 0.435 |
|  | rs12970134 | A | G | 0.056 | 0.008 | 5.31E-12 | -0.003 | 0.016 | 0.868 |
|  | rs13239186 | C | T | -0.054 | 0.009 | 2.70E-10 | 0.010 | 0.015 | 0.502 |
|  | rs13330951 | A | G | 0.046 | 0.008 | 1.54E-08 | -0.016 | 0.014 | 0.286 |
|  | rs13389219 | C | T | 0.072 | 0.007 | 2.11E-22 | -0.003 | 0.014 | 0.936 |
|  | rs1359790 | G | A | 0.080 | 0.008 | 2.80E-23 | -0.003 | 0.015 | 0.818 |
|  | rs1496653 | G | A | -0.077 | 0.009 | 2.57E-18 | -0.015 | 0.017 | 0.355 |
|  | rs1552224 | C | A | -0.103 | 0.010 | 8.64E-25 | -0.021 | 0.019 | 0.347 |
|  | rs16988333 | A | G | 0.075 | 0.013 | 9.17E-09 | -0.015 | 0.026 | 0.646 |
|  | rs17086692 | G | T | 0.047 | 0.008 | 2.48E-08 | -0.007 | 0.015 | 0.636 |
|  | rs17168486 | C | T | -0.074 | 0.009 | 2.18E-15 | 0.002 | 0.018 | 0.894 |
|  | rs17405722 | A | G | 0.087 | 0.015 | 2.28E-09 | -0.062 | 0.026 | 0.018 |
|  | rs17631783 | C | T | 0.049 | 0.009 | 3.95E-08 | 0.006 | 0.015 | 0.662 |
|  | rs17791513 | A | G | 0.103 | 0.015 | 4.61E-12 | -0.010 | 0.028 | 0.702 |
|  | rs1801214 | C | T | 0.090 | 0.007 | 5.52E-34 | -0.005 | 0.014 | 0.730 |
|  | rs1899951 | T | C | -0.112 | 0.011 | 1.64E-24 | -0.042 | 0.020 | 0.051 |
|  | rs2237892 | C | T | 0.096 | 0.016 | 8.75E-10 | -0.028 | 0.030 | 0.538 |
|  | rs2246618 | C | T | -0.051 | 0.008 | 1.20E-09 | -0.003 | 0.015 | 0.862 |
|  | rs2261181 | C | T | -0.099 | 0.012 | 9.18E-17 | 0.024 | 0.023 | 0.302 |
|  | rs2294120 | G | A | -0.044 | 0.008 | 1.62E-08 | -0.024 | 0.014 | 0.083 |
|  | rs2296173 | A | G | -0.065 | 0.009 | 7.66E-14 | -0.018 | 0.017 | 0.287 |
|  | rs2299383 | C | T | -0.041 | 0.007 | 1.49E-08 | 0.006 | 0.014 | 0.688 |
|  | rs243019 | T | C | -0.057 | 0.007 | 2.29E-15 | -0.017 | 0.014 | 0.213 |
|  | rs2493394 | G | A | 0.073 | 0.011 | 1.15E-10 | -0.013 | 0.022 | 0.526 |
|  | rs2796441 | G | A | 0.072 | 0.007 | 1.96E-22 | 0.011 | 0.014 | 0.451 |
|  | rs2820426 | G | A | 0.052 | 0.007 | 1.30E-12 | -0.007 | 0.014 | 0.637 |
|  | rs2867125 | T | C | -0.060 | 0.010 | 4.33E-10 | 0.018 | 0.018 | 0.311 |
|  | rs2908282 | G | A | -0.055 | 0.009 | 4.25E-09 | -0.021 | 0.018 | 0.247 |
|  | rs2925979 | T | C | 0.053 | 0.008 | 9.06E-12 | -0.004 | 0.015 | 0.823 |
|  | rs2943656 | A | G | -0.090 | 0.007 | 6.70E-34 | -0.013 | 0.014 | 0.406 |
|  | rs3217992 | T | C | 0.053 | 0.007 | 7.23E-13 | 0.001 | 0.014 | 0.924 |
|  | rs340874 | T | C | -0.063 | 0.007 | 8.41E-18 | 0.001 | 0.014 | 0.922 |
|  | rs348330 | G | A | 0.049 | 0.008 | 1.86E-09 | -0.019 | 0.014 | 0.189 |
|  | rs3756784 | G | T | 0.051 | 0.009 | 2.59E-08 | -0.009 | 0.017 | 0.613 |
|  | rs3802177 | G | A | 0.122 | 0.008 | 2.32E-52 | 0.017 | 0.015 | 0.257 |
|  | rs459193 | G | A | 0.071 | 0.008 | 8.81E-18 | -0.020 | 0.016 | 0.198 |
|  | rs4622883 | A | G | 0.044 | 0.008 | 3.02E-08 | 0.013 | 0.014 | 0.339 |
|  | rs4686471 | C | T | 0.053 | 0.008 | 4.28E-11 | -0.011 | 0.014 | 0.346 |
|  | rs4812829 | A | G | 0.053 | 0.010 | 2.44E-08 | -0.022 | 0.018 | 0.205 |
|  | rs4823182 | A | G | -0.048 | 0.008 | 3.36E-10 | 0.019 | 0.014 | 0.174 |
|  | rs4865796 | A | G | 0.053 | 0.008 | 1.33E-11 | -0.029 | 0.015 | 0.071 |
|  | rs516946 | T | C | -0.082 | 0.009 | 3.16E-22 | 0.001 | 0.016 | 0.987 |
|  | rs5215 | C | T | 0.068 | 0.007 | 2.09E-20 | -0.013 | 0.014 | 0.342 |
|  | rs576674 | A | G | -0.065 | 0.010 | 1.79E-11 | -0.005 | 0.018 | 0.797 |
|  | rs6059662 | A | G | -0.045 | 0.008 | 1.51E-08 | 0.005 | 0.015 | 0.739 |
|  | rs61953351 | G | T | 0.070 | 0.009 | 1.98E-14 | 0.004 | 0.016 | 0.809 |
|  | rs622217 | C | T | -0.049 | 0.008 | 3.13E-10 | 0.001 | 0.014 | 0.973 |
|  | rs6515236 | A | C | 0.050 | 0.009 | 3.34E-08 | 0.029 | 0.016 | 0.063 |
|  | rs67232546 | C | T | -0.060 | 0.010 | 4.66E-10 | -0.028 | 0.017 | 0.097 |
|  | rs6767484 | A | G | -0.121 | 0.008 | 2.70E-56 | 0.029 | 0.015 | 0.046 |
|  | rs6785040 | C | T | -0.063 | 0.011 | 1.26E-08 | 0.002 | 0.018 | 0.877 |
|  | rs6795735 | C | T | 0.056 | 0.007 | 1.63E-14 | -0.008 | 0.014 | 0.538 |
|  | rs6878122 | A | G | -0.056 | 0.008 | 1.19E-12 | 0.016 | 0.015 | 0.261 |
|  | rs6960043 | C | T | 0.064 | 0.007 | 3.61E-19 | 0.016 | 0.014 | 0.254 |
|  | rs7144011 | G | T | -0.048 | 0.009 | 1.64E-08 | -0.004 | 0.017 | 0.741 |
|  | rs7177055 | A | G | 0.065 | 0.008 | 2.75E-16 | 0.012 | 0.015 | 0.441 |
|  | rs7240767 | C | T | 0.045 | 0.008 | 2.16E-08 | -0.019 | 0.014 | 0.174 |
|  | rs72892910 | G | T | -0.065 | 0.010 | 6.43E-11 | -0.001 | 0.018 | 0.948 |
|  | rs735949 | C | T | -0.071 | 0.011 | 1.95E-11 | 0.008 | 0.020 | 0.667 |
|  | rs753270 | C | T | 0.053 | 0.008 | 2.70E-11 | 0.012 | 0.014 | 0.392 |
|  | rs7561798 | A | G | -0.040 | 0.007 | 2.79E-08 | -0.006 | 0.014 | 0.699 |
|  | rs7572970 | A | G | -0.059 | 0.009 | 1.39E-11 | -0.005 | 0.015 | 0.744 |
|  | rs7607777 | G | T | 0.137 | 0.013 | 9.40E-28 | 0.010 | 0.022 | 0.709 |
|  | rs7674212 | G | T | 0.047 | 0.008 | 6.18E-10 | -0.005 | 0.014 | 0.723 |
|  | rs7685296 | C | T | 0.051 | 0.008 | 2.32E-10 | -0.023 | 0.015 | 0.130 |
|  | rs7756992 | A | G | -0.130 | 0.008 | 6.00E-62 | 0.004 | 0.015 | 0.735 |
|  | rs7786095 | A | G | 0.074 | 0.013 | 9.64E-09 | -0.036 | 0.023 | 0.122 |
|  | rs780094 | T | C | -0.069 | 0.007 | 5.16E-21 | -0.018 | 0.014 | 0.188 |
|  | rs7845219 | C | T | -0.042 | 0.007 | 4.54E-09 | 0.006 | 0.014 | 0.676 |
|  | rs7903146 | C | T | -0.306 | 0.008 | 1.00E-200 | 0.005 | 0.015 | 0.716 |
|  | rs7929543 | A | C | -0.083 | 0.014 | 2.20E-09 | 0.042 | 0.024 | 0.078 |
|  | rs7955901 | C | T | 0.044 | 0.007 | 7.16E-10 | 0.000 | 0.014 | 0.989 |
|  | rs8068804 | A | G | 0.059 | 0.008 | 4.41E-14 | -0.007 | 0.015 | 0.613 |
|  | rs8108269 | G | T | 0.064 | 0.008 | 3.11E-16 | 0.006 | 0.015 | 0.681 |
|  | rs825476 | C | T | -0.052 | 0.007 | 6.80E-13 | -0.018 | 0.014 | 0.210 |
|  | rs840967 | A | C | -0.050 | 0.008 | 5.44E-10 | -0.002 | 0.014 | 0.876 |
|  | rs849135 | A | G | -0.100 | 0.007 | 1.04E-43 | 0.009 | 0.014 | 0.496 |
|  | rs853974 | C | T | -0.060 | 0.009 | 7.86E-12 | -0.035 | 0.015 | 0.021 |
|  | rs9369425 | A | G | -0.055 | 0.009 | 1.13E-10 | 0.001 | 0.015 | 0.931 |
|  | rs9894220 | A | G | 0.059 | 0.008 | 1.52E-13 | -0.013 | 0.014 | 0.343 |
|  | rs9928094 | A | G | -0.105 | 0.007 | 3.59E-47 | -0.005 | 0.014 | 0.724 |
|  | rs993380 | A | G | 0.051 | 0.008 | 4.59E-10 | 0.004 | 0.014 | 0.771 |
|  | rs9940149 | A | G | -0.058 | 0.010 | 9.29E-10 | 0.029 | 0.018 | 0.132 |
| *Peptostreptococcaceae* | rs10077431 | A | C | -0.049 | 0.009 | 4.75E-08 | -0.002 | 0.014 | 0.981 |
|  | rs10087241 | A | G | -0.048 | 0.008 | 2.80E-09 | 0.004 | 0.012 | 0.624 |
|  | rs10100265 | A | C | 0.049 | 0.008 | 6.29E-10 | -0.001 | 0.011 | 0.923 |
|  | rs10114341 | C | T | -0.041 | 0.007 | 1.15E-08 | 0.009 | 0.011 | 0.494 |
|  | rs10401969 | C | T | 0.092 | 0.013 | 4.13E-12 | 0.026 | 0.021 | 0.223 |
|  | rs1050226 | G | A | -0.049 | 0.007 | 3.34E-11 | -0.014 | 0.011 | 0.235 |
|  | rs1061813 | A | G | -0.043 | 0.007 | 3.37E-09 | 0.002 | 0.011 | 0.969 |
|  | rs1063355 | T | G | -0.071 | 0.008 | 3.72E-19 | 0.000 | 0.011 | 0.997 |
|  | rs10740322 | A | G | 0.048 | 0.009 | 2.11E-08 | 0.012 | 0.012 | 0.273 |
|  | rs10811661 | C | T | -0.157 | 0.010 | 4.13E-58 | 0.017 | 0.015 | 0.245 |
|  | rs10842994 | C | T | 0.076 | 0.009 | 1.02E-16 | 0.006 | 0.014 | 0.765 |
|  | rs10974438 | A | C | -0.059 | 0.008 | 3.01E-15 | 0.002 | 0.012 | 0.913 |
|  | rs11098676 | C | T | 0.054 | 0.010 | 2.03E-08 | 0.005 | 0.014 | 0.661 |
|  | rs11107116 | G | T | -0.047 | 0.009 | 3.75E-08 | 0.015 | 0.013 | 0.238 |
|  | rs1111875 | T | C | -0.095 | 0.007 | 3.61E-39 | -0.007 | 0.012 | 0.557 |
|  | rs11257655 | C | T | -0.074 | 0.009 | 1.97E-17 | -0.009 | 0.014 | 0.525 |
|  | rs1127655 | C | T | 0.044 | 0.008 | 2.47E-08 | -0.008 | 0.011 | 0.441 |
|  | rs11708067 | A | G | 0.097 | 0.009 | 5.93E-29 | 0.012 | 0.014 | 0.409 |
|  | rs11925227 | A | G | -0.053 | 0.010 | 2.25E-08 | -0.001 | 0.015 | 0.933 |
|  | rs11926707 | C | T | 0.046 | 0.008 | 1.69E-08 | 0.003 | 0.012 | 0.832 |
|  | rs12088739 | A | G | 0.088 | 0.013 | 9.79E-12 | 0.014 | 0.020 | 0.443 |
|  | rs12299509 | A | G | -0.047 | 0.007 | 2.09E-10 | -0.004 | 0.011 | 0.747 |
|  | rs12617659 | C | T | 0.069 | 0.010 | 2.83E-11 | 0.012 | 0.016 | 0.444 |
|  | rs12910825 | A | G | -0.052 | 0.007 | 2.16E-12 | 0.002 | 0.012 | 0.860 |
|  | rs12945601 | C | T | -0.048 | 0.008 | 1.72E-09 | -0.014 | 0.011 | 0.202 |
|  | rs12970134 | A | G | 0.056 | 0.008 | 5.31E-12 | -0.004 | 0.013 | 0.697 |
|  | rs13239186 | C | T | -0.054 | 0.009 | 2.70E-10 | -0.006 | 0.012 | 0.594 |
|  | rs13330951 | A | G | 0.046 | 0.008 | 1.54E-08 | -0.007 | 0.011 | 0.491 |
|  | rs13389219 | C | T | 0.072 | 0.007 | 2.11E-22 | -0.013 | 0.012 | 0.321 |
|  | rs1359790 | G | A | 0.080 | 0.008 | 2.80E-23 | 0.001 | 0.013 | 0.954 |
|  | rs1496653 | G | A | -0.077 | 0.009 | 2.57E-18 | 0.003 | 0.014 | 0.800 |
|  | rs1552224 | C | A | -0.103 | 0.010 | 8.64E-25 | 0.009 | 0.016 | 0.630 |
|  | rs16988333 | A | G | 0.075 | 0.013 | 9.17E-09 | -0.022 | 0.022 | 0.310 |
|  | rs17086692 | G | T | 0.047 | 0.008 | 2.48E-08 | 0.003 | 0.012 | 0.856 |
|  | rs17168486 | C | T | -0.074 | 0.009 | 2.18E-15 | 0.014 | 0.014 | 0.381 |
|  | rs17405722 | A | G | 0.087 | 0.015 | 2.28E-09 | 0.004 | 0.022 | 0.877 |
|  | rs17631783 | C | T | 0.049 | 0.009 | 3.95E-08 | -0.023 | 0.013 | 0.074 |
|  | rs17791513 | A | G | 0.103 | 0.015 | 4.61E-12 | -0.026 | 0.023 | 0.220 |
|  | rs1801214 | C | T | 0.090 | 0.007 | 5.52E-34 | -0.012 | 0.012 | 0.316 |
|  | rs1899951 | T | C | -0.112 | 0.011 | 1.64E-24 | -0.011 | 0.016 | 0.473 |
|  | rs2237892 | C | T | 0.096 | 0.016 | 8.75E-10 | -0.015 | 0.023 | 0.957 |
|  | rs2246618 | C | T | -0.051 | 0.008 | 1.20E-09 | 0.020 | 0.012 | 0.088 |
|  | rs2261181 | C | T | -0.099 | 0.012 | 9.18E-17 | 0.017 | 0.019 | 0.416 |
|  | rs2294120 | G | A | -0.044 | 0.008 | 1.62E-08 | -0.002 | 0.011 | 0.852 |
|  | rs2296173 | A | G | -0.065 | 0.009 | 7.66E-14 | -0.034 | 0.014 | 0.013 |
|  | rs2299383 | C | T | -0.041 | 0.007 | 1.49E-08 | -0.006 | 0.011 | 0.602 |
|  | rs243019 | T | C | -0.057 | 0.007 | 2.29E-15 | -0.009 | 0.011 | 0.432 |
|  | rs2493394 | G | A | 0.073 | 0.011 | 1.15E-10 | -0.024 | 0.018 | 0.210 |
|  | rs2796441 | G | A | 0.072 | 0.007 | 1.96E-22 | 0.008 | 0.011 | 0.509 |
|  | rs2820426 | G | A | 0.052 | 0.007 | 1.30E-12 | 0.016 | 0.011 | 0.174 |
|  | rs2867125 | T | C | -0.060 | 0.010 | 4.33E-10 | -0.006 | 0.015 | 0.637 |
|  | rs2908282 | G | A | -0.055 | 0.009 | 4.25E-09 | -0.018 | 0.015 | 0.219 |
|  | rs2925979 | T | C | 0.053 | 0.008 | 9.06E-12 | 0.001 | 0.012 | 0.967 |
|  | rs2943656 | A | G | -0.090 | 0.007 | 6.70E-34 | -0.014 | 0.012 | 0.186 |
|  | rs3217992 | T | C | 0.053 | 0.007 | 7.23E-13 | 0.018 | 0.012 | 0.121 |
|  | rs340874 | T | C | -0.063 | 0.007 | 8.41E-18 | 0.006 | 0.011 | 0.612 |
|  | rs348330 | G | A | 0.049 | 0.008 | 1.86E-09 | -0.008 | 0.012 | 0.491 |
|  | rs3756784 | G | T | 0.051 | 0.009 | 2.59E-08 | 0.004 | 0.014 | 0.803 |
|  | rs3802177 | G | A | 0.122 | 0.008 | 2.32E-52 | -0.002 | 0.012 | 0.835 |
|  | rs459193 | G | A | 0.071 | 0.008 | 8.81E-18 | 0.011 | 0.013 | 0.344 |
|  | rs4622883 | A | G | 0.044 | 0.008 | 3.02E-08 | 0.006 | 0.011 | 0.598 |
|  | rs4686471 | C | T | 0.053 | 0.008 | 4.28E-11 | -0.014 | 0.012 | 0.205 |
|  | rs4812829 | A | G | 0.053 | 0.010 | 2.44E-08 | 0.038 | 0.015 | 0.010 |
|  | rs4823182 | A | G | -0.048 | 0.008 | 3.36E-10 | 0.016 | 0.012 | 0.176 |
|  | rs4865796 | A | G | 0.053 | 0.008 | 1.33E-11 | -0.008 | 0.012 | 0.530 |
|  | rs516946 | T | C | -0.082 | 0.009 | 3.16E-22 | -0.003 | 0.013 | 0.873 |
|  | rs5215 | C | T | 0.068 | 0.007 | 2.09E-20 | -0.004 | 0.012 | 0.706 |
|  | rs576674 | A | G | -0.065 | 0.010 | 1.79E-11 | -0.018 | 0.015 | 0.243 |
|  | rs6059662 | A | G | -0.045 | 0.008 | 1.51E-08 | -0.008 | 0.012 | 0.543 |
|  | rs61953351 | G | T | 0.070 | 0.009 | 1.98E-14 | -0.001 | 0.013 | 0.967 |
|  | rs622217 | C | T | -0.049 | 0.008 | 3.13E-10 | -0.006 | 0.011 | 0.591 |
|  | rs6515236 | A | C | 0.050 | 0.009 | 3.34E-08 | 0.011 | 0.013 | 0.420 |
|  | rs67232546 | C | T | -0.060 | 0.010 | 4.66E-10 | -0.012 | 0.014 | 0.406 |
|  | rs6767484 | A | G | -0.121 | 0.008 | 2.70E-56 | 0.036 | 0.012 | 0.003 |
|  | rs6785040 | C | T | -0.063 | 0.011 | 1.26E-08 | 0.017 | 0.015 | 0.225 |
|  | rs6795735 | C | T | 0.056 | 0.007 | 1.63E-14 | -0.007 | 0.011 | 0.538 |
|  | rs6878122 | A | G | -0.056 | 0.008 | 1.19E-12 | -0.008 | 0.012 | 0.455 |
|  | rs6960043 | C | T | 0.064 | 0.007 | 3.61E-19 | 0.020 | 0.011 | 0.078 |
|  | rs7144011 | G | T | -0.048 | 0.009 | 1.64E-08 | 0.003 | 0.014 | 0.833 |
|  | rs7177055 | A | G | 0.065 | 0.008 | 2.75E-16 | -0.024 | 0.012 | 0.051 |
|  | rs7240767 | C | T | 0.045 | 0.008 | 2.16E-08 | -0.006 | 0.012 | 0.570 |
|  | rs72892910 | G | T | -0.065 | 0.010 | 6.43E-11 | 0.004 | 0.015 | 0.703 |
|  | rs735949 | C | T | -0.071 | 0.011 | 1.95E-11 | -0.011 | 0.017 | 0.540 |
|  | rs753270 | C | T | 0.053 | 0.008 | 2.70E-11 | 0.000 | 0.011 | 0.989 |
|  | rs7561798 | A | G | -0.040 | 0.007 | 2.79E-08 | 0.004 | 0.011 | 0.741 |
|  | rs7572970 | A | G | -0.059 | 0.009 | 1.39E-11 | -0.003 | 0.013 | 0.758 |
|  | rs7607777 | G | T | 0.137 | 0.013 | 9.40E-28 | -0.007 | 0.019 | 0.602 |
|  | rs7674212 | G | T | 0.047 | 0.008 | 6.18E-10 | 0.001 | 0.011 | 0.933 |
|  | rs7685296 | C | T | 0.051 | 0.008 | 2.32E-10 | -0.025 | 0.012 | 0.041 |
|  | rs7729395 | C | T | -0.137 | 0.016 | 1.10E-17 | -0.015 | 0.057 | 0.878 |
|  | rs7756992 | A | G | -0.130 | 0.008 | 6.00E-62 | 0.016 | 0.012 | 0.213 |
|  | rs7786095 | A | G | 0.074 | 0.013 | 9.64E-09 | -0.028 | 0.019 | 0.157 |
|  | rs780094 | T | C | -0.069 | 0.007 | 5.16E-21 | -0.009 | 0.012 | 0.446 |
|  | rs7845219 | C | T | -0.042 | 0.007 | 4.54E-09 | 0.014 | 0.011 | 0.234 |
|  | rs7903146 | C | T | -0.306 | 0.008 | 1.00E-200 | -0.018 | 0.013 | 0.164 |
|  | rs7929543 | A | C | -0.083 | 0.014 | 2.20E-09 | -0.023 | 0.019 | 0.239 |
|  | rs7955901 | C | T | 0.044 | 0.007 | 7.16E-10 | -0.013 | 0.011 | 0.235 |
|  | rs8068804 | A | G | 0.059 | 0.008 | 4.41E-14 | 0.001 | 0.012 | 0.935 |
|  | rs8108269 | G | T | 0.064 | 0.008 | 3.11E-16 | -0.020 | 0.012 | 0.093 |
|  | rs825476 | C | T | -0.052 | 0.007 | 6.80E-13 | 0.011 | 0.011 | 0.297 |
|  | rs840967 | A | C | -0.050 | 0.008 | 5.44E-10 | 0.008 | 0.011 | 0.459 |
|  | rs849135 | A | G | -0.100 | 0.007 | 1.04E-43 | 0.013 | 0.011 | 0.252 |
|  | rs853974 | C | T | -0.060 | 0.009 | 7.86E-12 | 0.008 | 0.013 | 0.524 |
|  | rs9369425 | A | G | -0.055 | 0.009 | 1.13E-10 | 0.012 | 0.013 | 0.297 |
|  | rs9894220 | A | G | 0.059 | 0.008 | 1.52E-13 | -0.005 | 0.011 | 0.652 |
|  | rs9928094 | A | G | -0.105 | 0.007 | 3.59E-47 | -0.016 | 0.011 | 0.154 |
|  | rs993380 | A | G | 0.051 | 0.008 | 4.59E-10 | -0.003 | 0.012 | 0.768 |
|  | rs9940149 | A | G | -0.058 | 0.010 | 9.29E-10 | -0.002 | 0.014 | 0.771 |
| *Porphyromonadaceae* | rs10077431 | A | C | -0.049 | 0.009 | 4.75E-08 | -0.003 | 0.013 | 0.881 |
|  | rs10087241 | A | G | -0.048 | 0.008 | 2.80E-09 | 0.005 | 0.011 | 0.647 |
|  | rs10100265 | A | C | 0.049 | 0.008 | 6.29E-10 | -0.004 | 0.011 | 0.736 |
|  | rs10114341 | C | T | -0.041 | 0.007 | 1.15E-08 | 0.006 | 0.011 | 0.557 |
|  | rs10401969 | C | T | 0.092 | 0.013 | 4.13E-12 | 0.004 | 0.020 | 0.871 |
|  | rs1050226 | G | A | -0.049 | 0.007 | 3.34E-11 | -0.013 | 0.011 | 0.256 |
|  | rs1061813 | A | G | -0.043 | 0.007 | 3.37E-09 | -0.001 | 0.011 | 0.903 |
|  | rs1063355 | T | G | -0.071 | 0.008 | 3.72E-19 | -0.005 | 0.011 | 0.626 |
|  | rs10740322 | A | G | 0.048 | 0.009 | 2.11E-08 | 0.001 | 0.011 | 0.965 |
|  | rs10811661 | C | T | -0.157 | 0.010 | 4.13E-58 | -0.008 | 0.014 | 0.636 |
|  | rs10842994 | C | T | 0.076 | 0.009 | 1.02E-16 | -0.001 | 0.013 | 0.906 |
|  | rs10974438 | A | C | -0.059 | 0.008 | 3.01E-15 | 0.004 | 0.011 | 0.755 |
|  | rs11098676 | C | T | 0.054 | 0.010 | 2.03E-08 | -0.017 | 0.013 | 0.161 |
|  | rs11107116 | G | T | -0.047 | 0.009 | 3.75E-08 | 0.006 | 0.013 | 0.705 |
|  | rs1111875 | T | C | -0.095 | 0.007 | 3.61E-39 | 0.011 | 0.011 | 0.286 |
|  | rs11257655 | C | T | -0.074 | 0.009 | 1.97E-17 | 0.011 | 0.013 | 0.390 |
|  | rs1127655 | C | T | 0.044 | 0.008 | 2.47E-08 | -0.002 | 0.011 | 0.847 |
|  | rs11708067 | A | G | 0.097 | 0.009 | 5.93E-29 | -0.016 | 0.013 | 0.252 |
|  | rs11925227 | A | G | -0.053 | 0.010 | 2.25E-08 | 0.020 | 0.014 | 0.145 |
|  | rs11926707 | C | T | 0.046 | 0.008 | 1.69E-08 | 0.004 | 0.011 | 0.724 |
|  | rs12088739 | A | G | 0.088 | 0.013 | 9.79E-12 | -0.009 | 0.018 | 0.666 |
|  | rs12299509 | A | G | -0.047 | 0.007 | 2.09E-10 | 0.002 | 0.011 | 0.802 |
|  | rs12617659 | C | T | 0.069 | 0.010 | 2.83E-11 | -0.013 | 0.015 | 0.412 |
|  | rs12910825 | A | G | -0.052 | 0.007 | 2.16E-12 | -0.004 | 0.011 | 0.761 |
|  | rs12945601 | C | T | -0.048 | 0.008 | 1.72E-09 | -0.004 | 0.011 | 0.681 |
|  | rs12970134 | A | G | 0.056 | 0.008 | 5.31E-12 | 0.020 | 0.012 | 0.082 |
|  | rs13239186 | C | T | -0.054 | 0.009 | 2.70E-10 | 0.000 | 0.012 | 0.965 |
|  | rs13330951 | A | G | 0.046 | 0.008 | 1.54E-08 | -0.016 | 0.011 | 0.155 |
|  | rs13389219 | C | T | 0.072 | 0.007 | 2.11E-22 | -0.013 | 0.011 | 0.243 |
|  | rs1359790 | G | A | 0.080 | 0.008 | 2.80E-23 | -0.001 | 0.012 | 0.935 |
|  | rs1496653 | G | A | -0.077 | 0.009 | 2.57E-18 | 0.022 | 0.013 | 0.082 |
|  | rs1552224 | C | A | -0.103 | 0.010 | 8.64E-25 | 0.013 | 0.015 | 0.449 |
|  | rs16988333 | A | G | 0.075 | 0.013 | 9.17E-09 | -0.022 | 0.021 | 0.348 |
|  | rs17086692 | G | T | 0.047 | 0.008 | 2.48E-08 | -0.001 | 0.011 | 0.900 |
|  | rs17168486 | C | T | -0.074 | 0.009 | 2.18E-15 | -0.022 | 0.014 | 0.115 |
|  | rs17405722 | A | G | 0.087 | 0.015 | 2.28E-09 | -0.032 | 0.021 | 0.130 |
|  | rs17631783 | C | T | 0.049 | 0.009 | 3.95E-08 | 0.001 | 0.012 | 0.940 |
|  | rs17791513 | A | G | 0.103 | 0.015 | 4.61E-12 | 0.042 | 0.021 | 0.066 |
|  | rs1801214 | C | T | 0.090 | 0.007 | 5.52E-34 | -0.001 | 0.011 | 0.932 |
|  | rs1899951 | T | C | -0.112 | 0.011 | 1.64E-24 | 0.021 | 0.016 | 0.182 |
|  | rs2237892 | C | T | 0.096 | 0.016 | 8.75E-10 | -0.020 | 0.021 | 0.345 |
|  | rs2246618 | C | T | -0.051 | 0.008 | 1.20E-09 | -0.027 | 0.011 | 0.014 |
|  | rs2261181 | C | T | -0.099 | 0.012 | 9.18E-17 | 0.009 | 0.018 | 0.567 |
|  | rs2294120 | G | A | -0.044 | 0.008 | 1.62E-08 | 0.001 | 0.011 | 0.889 |
|  | rs2296173 | A | G | -0.065 | 0.009 | 7.66E-14 | 0.004 | 0.013 | 0.789 |
|  | rs2299383 | C | T | -0.041 | 0.007 | 1.49E-08 | 0.003 | 0.011 | 0.797 |
|  | rs243019 | T | C | -0.057 | 0.007 | 2.29E-15 | 0.004 | 0.011 | 0.712 |
|  | rs2493394 | G | A | 0.073 | 0.011 | 1.15E-10 | -0.006 | 0.017 | 0.587 |
|  | rs2796441 | G | A | 0.072 | 0.007 | 1.96E-22 | -0.006 | 0.011 | 0.566 |
|  | rs2820426 | G | A | 0.052 | 0.007 | 1.30E-12 | 0.001 | 0.011 | 0.913 |
|  | rs2867125 | T | C | -0.060 | 0.010 | 4.33E-10 | -0.017 | 0.014 | 0.244 |
|  | rs2908282 | G | A | -0.055 | 0.009 | 4.25E-09 | 0.005 | 0.014 | 0.743 |
|  | rs2925979 | T | C | 0.053 | 0.008 | 9.06E-12 | -0.016 | 0.012 | 0.170 |
|  | rs2943656 | A | G | -0.090 | 0.007 | 6.70E-34 | 0.012 | 0.011 | 0.215 |
|  | rs3217992 | T | C | 0.053 | 0.007 | 7.23E-13 | 0.007 | 0.011 | 0.508 |
|  | rs340874 | T | C | -0.063 | 0.007 | 8.41E-18 | 0.004 | 0.011 | 0.708 |
|  | rs348330 | G | A | 0.049 | 0.008 | 1.86E-09 | 0.011 | 0.011 | 0.323 |
|  | rs3756784 | G | T | 0.051 | 0.009 | 2.59E-08 | -0.002 | 0.013 | 0.848 |
|  | rs3802177 | G | A | 0.122 | 0.008 | 2.32E-52 | -0.009 | 0.011 | 0.465 |
|  | rs459193 | G | A | 0.071 | 0.008 | 8.81E-18 | 0.002 | 0.012 | 0.906 |
|  | rs4622883 | A | G | 0.044 | 0.008 | 3.02E-08 | 0.004 | 0.011 | 0.721 |
|  | rs4686471 | C | T | 0.053 | 0.008 | 4.28E-11 | 0.001 | 0.011 | 0.940 |
|  | rs4812829 | A | G | 0.053 | 0.010 | 2.44E-08 | -0.024 | 0.014 | 0.069 |
|  | rs4823182 | A | G | -0.048 | 0.008 | 3.36E-10 | -0.003 | 0.011 | 0.726 |
|  | rs4865796 | A | G | 0.053 | 0.008 | 1.33E-11 | -0.010 | 0.012 | 0.394 |
|  | rs516946 | T | C | -0.082 | 0.009 | 3.16E-22 | 0.000 | 0.012 | 0.997 |
|  | rs5215 | C | T | 0.068 | 0.007 | 2.09E-20 | 0.000 | 0.011 | 0.964 |
|  | rs576674 | A | G | -0.065 | 0.010 | 1.79E-11 | 0.008 | 0.014 | 0.435 |
|  | rs6059662 | A | G | -0.045 | 0.008 | 1.51E-08 | 0.001 | 0.012 | 0.954 |
|  | rs61953351 | G | T | 0.070 | 0.009 | 1.98E-14 | -0.007 | 0.012 | 0.524 |
|  | rs622217 | C | T | -0.049 | 0.008 | 3.13E-10 | 0.007 | 0.011 | 0.529 |
|  | rs6515236 | A | C | 0.050 | 0.009 | 3.34E-08 | 0.005 | 0.012 | 0.654 |
|  | rs67232546 | C | T | -0.060 | 0.010 | 4.66E-10 | -0.005 | 0.013 | 0.687 |
|  | rs6767484 | A | G | -0.121 | 0.008 | 2.70E-56 | -0.008 | 0.011 | 0.475 |
|  | rs6785040 | C | T | -0.063 | 0.011 | 1.26E-08 | -0.015 | 0.014 | 0.328 |
|  | rs6795735 | C | T | 0.056 | 0.007 | 1.63E-14 | 0.008 | 0.011 | 0.461 |
|  | rs6878122 | A | G | -0.056 | 0.008 | 1.19E-12 | 0.020 | 0.012 | 0.103 |
|  | rs6960043 | C | T | 0.064 | 0.007 | 3.61E-19 | -0.010 | 0.011 | 0.346 |
|  | rs7144011 | G | T | -0.048 | 0.009 | 1.64E-08 | 0.005 | 0.013 | 0.714 |
|  | rs7177055 | A | G | 0.065 | 0.008 | 2.75E-16 | -0.002 | 0.012 | 0.856 |
|  | rs7240767 | C | T | 0.045 | 0.008 | 2.16E-08 | 0.005 | 0.011 | 0.631 |
|  | rs72892910 | G | T | -0.065 | 0.010 | 6.43E-11 | -0.016 | 0.014 | 0.234 |
|  | rs735949 | C | T | -0.071 | 0.011 | 1.95E-11 | 0.001 | 0.016 | 0.862 |
|  | rs753270 | C | T | 0.053 | 0.008 | 2.70E-11 | 0.000 | 0.011 | 0.984 |
|  | rs7561798 | A | G | -0.040 | 0.007 | 2.79E-08 | 0.005 | 0.011 | 0.630 |
|  | rs7572970 | A | G | -0.059 | 0.009 | 1.39E-11 | -0.019 | 0.012 | 0.104 |
|  | rs7607777 | G | T | 0.137 | 0.013 | 9.40E-28 | 0.024 | 0.018 | 0.175 |
|  | rs7674212 | G | T | 0.047 | 0.008 | 6.18E-10 | -0.014 | 0.011 | 0.174 |
|  | rs7685296 | C | T | 0.051 | 0.008 | 2.32E-10 | 0.019 | 0.012 | 0.097 |
|  | rs7729395 | C | T | -0.137 | 0.016 | 1.10E-17 | 0.030 | 0.056 | 0.629 |
|  | rs7756992 | A | G | -0.130 | 0.008 | 6.00E-62 | 0.002 | 0.012 | 0.842 |
|  | rs7786095 | A | G | 0.074 | 0.013 | 9.64E-09 | -0.010 | 0.018 | 0.590 |
|  | rs780094 | T | C | -0.069 | 0.007 | 5.16E-21 | -0.007 | 0.011 | 0.516 |
|  | rs7845219 | C | T | -0.042 | 0.007 | 4.54E-09 | -0.007 | 0.011 | 0.521 |
|  | rs7903146 | C | T | -0.306 | 0.008 | 1.00E-200 | 0.012 | 0.012 | 0.321 |
|  | rs7929543 | A | C | -0.083 | 0.014 | 2.20E-09 | -0.007 | 0.018 | 0.754 |
|  | rs7955901 | C | T | 0.044 | 0.007 | 7.16E-10 | 0.012 | 0.011 | 0.249 |
|  | rs8068804 | A | G | 0.059 | 0.008 | 4.41E-14 | -0.009 | 0.011 | 0.432 |
|  | rs8108269 | G | T | 0.064 | 0.008 | 3.11E-16 | 0.012 | 0.011 | 0.302 |
|  | rs825476 | C | T | -0.052 | 0.007 | 6.80E-13 | -0.015 | 0.011 | 0.169 |
|  | rs840967 | A | C | -0.050 | 0.008 | 5.44E-10 | -0.006 | 0.011 | 0.584 |
|  | rs849135 | A | G | -0.100 | 0.007 | 1.04E-43 | -0.005 | 0.011 | 0.607 |
|  | rs853974 | C | T | -0.060 | 0.009 | 7.86E-12 | -0.023 | 0.012 | 0.053 |
|  | rs9369425 | A | G | -0.055 | 0.009 | 1.13E-10 | 0.004 | 0.012 | 0.733 |
|  | rs9894220 | A | G | 0.059 | 0.008 | 1.52E-13 | 0.001 | 0.011 | 0.900 |
|  | rs9928094 | A | G | -0.105 | 0.007 | 3.59E-47 | -0.001 | 0.011 | 0.903 |
|  | rs993380 | A | G | 0.051 | 0.008 | 4.59E-10 | -0.016 | 0.011 | 0.155 |
|  | rs9940149 | A | G | -0.058 | 0.010 | 9.29E-10 | -0.016 | 0.013 | 0.297 |
| *Prevotellaceae* | rs10077431 | A | C | -0.049 | 0.009 | 4.75E-08 | 0.002 | 0.015 | 0.841 |
|  | rs10087241 | A | G | -0.048 | 0.008 | 2.80E-09 | 0.008 | 0.012 | 0.505 |
|  | rs10100265 | A | C | 0.049 | 0.008 | 6.29E-10 | -0.006 | 0.012 | 0.582 |
|  | rs10114341 | C | T | -0.041 | 0.007 | 1.15E-08 | 0.002 | 0.012 | 0.869 |
|  | rs10401969 | C | T | 0.092 | 0.013 | 4.13E-12 | 0.021 | 0.022 | 0.321 |
|  | rs1050226 | G | A | -0.049 | 0.007 | 3.34E-11 | -0.003 | 0.012 | 0.775 |
|  | rs1061813 | A | G | -0.043 | 0.007 | 3.37E-09 | 0.030 | 0.012 | 0.009 |
|  | rs1063355 | T | G | -0.071 | 0.008 | 3.72E-19 | 0.003 | 0.012 | 0.789 |
|  | rs10740322 | A | G | 0.048 | 0.009 | 2.11E-08 | 0.014 | 0.012 | 0.271 |
|  | rs10811661 | C | T | -0.157 | 0.010 | 4.13E-58 | 0.001 | 0.015 | 0.943 |
|  | rs10842994 | C | T | 0.076 | 0.009 | 1.02E-16 | -0.006 | 0.015 | 0.656 |
|  | rs10974438 | A | C | -0.059 | 0.008 | 3.01E-15 | -0.002 | 0.012 | 0.853 |
|  | rs11098676 | C | T | 0.054 | 0.010 | 2.03E-08 | 0.014 | 0.014 | 0.301 |
|  | rs11107116 | G | T | -0.047 | 0.009 | 3.75E-08 | -0.026 | 0.014 | 0.064 |
|  | rs1111875 | T | C | -0.095 | 0.007 | 3.61E-39 | -0.011 | 0.012 | 0.377 |
|  | rs11257655 | C | T | -0.074 | 0.009 | 1.97E-17 | -0.009 | 0.014 | 0.502 |
|  | rs1127655 | C | T | 0.044 | 0.008 | 2.47E-08 | 0.002 | 0.012 | 0.832 |
|  | rs11708067 | A | G | 0.097 | 0.009 | 5.93E-29 | 0.003 | 0.014 | 0.870 |
|  | rs11925227 | A | G | -0.053 | 0.010 | 2.25E-08 | -0.019 | 0.015 | 0.216 |
|  | rs11926707 | C | T | 0.046 | 0.008 | 1.69E-08 | 0.007 | 0.012 | 0.569 |
|  | rs12088739 | A | G | 0.088 | 0.013 | 9.79E-12 | -0.007 | 0.020 | 0.827 |
|  | rs12299509 | A | G | -0.047 | 0.007 | 2.09E-10 | -0.002 | 0.012 | 0.865 |
|  | rs12617659 | C | T | 0.069 | 0.010 | 2.83E-11 | -0.005 | 0.017 | 0.828 |
|  | rs12910825 | A | G | -0.052 | 0.007 | 2.16E-12 | -0.019 | 0.012 | 0.121 |
|  | rs12945601 | C | T | -0.048 | 0.008 | 1.72E-09 | 0.007 | 0.012 | 0.532 |
|  | rs12970134 | A | G | 0.056 | 0.008 | 5.31E-12 | 0.001 | 0.013 | 0.851 |
|  | rs13239186 | C | T | -0.054 | 0.009 | 2.70E-10 | 0.028 | 0.013 | 0.025 |
|  | rs13330951 | A | G | 0.046 | 0.008 | 1.54E-08 | -0.005 | 0.012 | 0.654 |
|  | rs13389219 | C | T | 0.072 | 0.007 | 2.11E-22 | -0.033 | 0.012 | 0.012 |
|  | rs1359790 | G | A | 0.080 | 0.008 | 2.80E-23 | 0.011 | 0.013 | 0.418 |
|  | rs1496653 | G | A | -0.077 | 0.009 | 2.57E-18 | -0.011 | 0.014 | 0.422 |
|  | rs1552224 | C | A | -0.103 | 0.010 | 8.64E-25 | 0.005 | 0.016 | 0.751 |
|  | rs16988333 | A | G | 0.075 | 0.013 | 9.17E-09 | -0.019 | 0.023 | 0.467 |
|  | rs17086692 | G | T | 0.047 | 0.008 | 2.48E-08 | 0.017 | 0.012 | 0.189 |
|  | rs17168486 | C | T | -0.074 | 0.009 | 2.18E-15 | 0.013 | 0.015 | 0.438 |
|  | rs17405722 | A | G | 0.087 | 0.015 | 2.28E-09 | 0.017 | 0.024 | 0.424 |
|  | rs17631783 | C | T | 0.049 | 0.009 | 3.95E-08 | 0.003 | 0.013 | 0.782 |
|  | rs17791513 | A | G | 0.103 | 0.015 | 4.61E-12 | -0.006 | 0.024 | 0.739 |
|  | rs1801214 | C | T | 0.090 | 0.007 | 5.52E-34 | 0.013 | 0.012 | 0.294 |
|  | rs1899951 | T | C | -0.112 | 0.011 | 1.64E-24 | -0.005 | 0.017 | 0.822 |
|  | rs2237892 | C | T | 0.096 | 0.016 | 8.75E-10 | -0.026 | 0.023 | 0.357 |
|  | rs2246618 | C | T | -0.051 | 0.008 | 1.20E-09 | -0.018 | 0.013 | 0.169 |
|  | rs2261181 | C | T | -0.099 | 0.012 | 9.18E-17 | -0.008 | 0.019 | 0.618 |
|  | rs2294120 | G | A | -0.044 | 0.008 | 1.62E-08 | 0.007 | 0.012 | 0.588 |
|  | rs2296173 | A | G | -0.065 | 0.009 | 7.66E-14 | -0.006 | 0.014 | 0.712 |
|  | rs2299383 | C | T | -0.041 | 0.007 | 1.49E-08 | 0.017 | 0.012 | 0.156 |
|  | rs243019 | T | C | -0.057 | 0.007 | 2.29E-15 | -0.007 | 0.012 | 0.547 |
|  | rs2493394 | G | A | 0.073 | 0.011 | 1.15E-10 | -0.030 | 0.019 | 0.104 |
|  | rs2796441 | G | A | 0.072 | 0.007 | 1.96E-22 | 0.006 | 0.012 | 0.591 |
|  | rs2820426 | G | A | 0.052 | 0.007 | 1.30E-12 | -0.029 | 0.012 | 0.016 |
|  | rs2867125 | T | C | -0.060 | 0.010 | 4.33E-10 | -0.006 | 0.016 | 0.641 |
|  | rs2908282 | G | A | -0.055 | 0.009 | 4.25E-09 | -0.016 | 0.015 | 0.337 |
|  | rs2925979 | T | C | 0.053 | 0.008 | 9.06E-12 | -0.011 | 0.013 | 0.407 |
|  | rs2943656 | A | G | -0.090 | 0.007 | 6.70E-34 | -0.018 | 0.012 | 0.168 |
|  | rs3217992 | T | C | 0.053 | 0.007 | 7.23E-13 | 0.006 | 0.012 | 0.652 |
|  | rs340874 | T | C | -0.063 | 0.007 | 8.41E-18 | -0.018 | 0.012 | 0.142 |
|  | rs348330 | G | A | 0.049 | 0.008 | 1.86E-09 | -0.014 | 0.012 | 0.254 |
|  | rs3756784 | G | T | 0.051 | 0.009 | 2.59E-08 | 0.007 | 0.014 | 0.660 |
|  | rs3802177 | G | A | 0.122 | 0.008 | 2.32E-52 | -0.006 | 0.013 | 0.633 |
|  | rs459193 | G | A | 0.071 | 0.008 | 8.81E-18 | 0.009 | 0.013 | 0.512 |
|  | rs4622883 | A | G | 0.044 | 0.008 | 3.02E-08 | -0.006 | 0.012 | 0.637 |
|  | rs4686471 | C | T | 0.053 | 0.008 | 4.28E-11 | 0.013 | 0.012 | 0.234 |
|  | rs4812829 | A | G | 0.053 | 0.010 | 2.44E-08 | -0.003 | 0.015 | 0.900 |
|  | rs4823182 | A | G | -0.048 | 0.008 | 3.36E-10 | -0.003 | 0.012 | 0.797 |
|  | rs4865796 | A | G | 0.053 | 0.008 | 1.33E-11 | -0.010 | 0.013 | 0.413 |
|  | rs516946 | T | C | -0.082 | 0.009 | 3.16E-22 | -0.003 | 0.014 | 0.828 |
|  | rs5215 | C | T | 0.068 | 0.007 | 2.09E-20 | -0.002 | 0.012 | 0.867 |
|  | rs576674 | A | G | -0.065 | 0.010 | 1.79E-11 | -0.008 | 0.015 | 0.673 |
|  | rs6059662 | A | G | -0.045 | 0.008 | 1.51E-08 | 0.004 | 0.013 | 0.761 |
|  | rs61953351 | G | T | 0.070 | 0.009 | 1.98E-14 | -0.004 | 0.014 | 0.760 |
|  | rs622217 | C | T | -0.049 | 0.008 | 3.13E-10 | 0.003 | 0.012 | 0.814 |
|  | rs6515236 | A | C | 0.050 | 0.009 | 3.34E-08 | -0.020 | 0.013 | 0.152 |
|  | rs67232546 | C | T | -0.060 | 0.010 | 4.66E-10 | 0.030 | 0.015 | 0.042 |
|  | rs6767484 | A | G | -0.121 | 0.008 | 2.70E-56 | 0.011 | 0.013 | 0.393 |
|  | rs6785040 | C | T | -0.063 | 0.011 | 1.26E-08 | -0.029 | 0.016 | 0.039 |
|  | rs6795735 | C | T | 0.056 | 0.007 | 1.63E-14 | -0.019 | 0.012 | 0.103 |
|  | rs6878122 | A | G | -0.056 | 0.008 | 1.19E-12 | -0.025 | 0.013 | 0.045 |
|  | rs6960043 | C | T | 0.064 | 0.007 | 3.61E-19 | -0.019 | 0.012 | 0.111 |
|  | rs7144011 | G | T | -0.048 | 0.009 | 1.64E-08 | 0.002 | 0.015 | 0.948 |
|  | rs7177055 | A | G | 0.065 | 0.008 | 2.75E-16 | 0.006 | 0.013 | 0.606 |
|  | rs7240767 | C | T | 0.045 | 0.008 | 2.16E-08 | 0.009 | 0.012 | 0.488 |
|  | rs72892910 | G | T | -0.065 | 0.010 | 6.43E-11 | 0.019 | 0.015 | 0.235 |
|  | rs735949 | C | T | -0.071 | 0.011 | 1.95E-11 | 0.011 | 0.017 | 0.485 |
|  | rs753270 | C | T | 0.053 | 0.008 | 2.70E-11 | -0.003 | 0.012 | 0.816 |
|  | rs7561798 | A | G | -0.040 | 0.007 | 2.79E-08 | -0.008 | 0.012 | 0.490 |
|  | rs7572970 | A | G | -0.059 | 0.009 | 1.39E-11 | 0.006 | 0.013 | 0.641 |
|  | rs7607777 | G | T | 0.137 | 0.013 | 9.40E-28 | 0.037 | 0.019 | 0.057 |
|  | rs7674212 | G | T | 0.047 | 0.008 | 6.18E-10 | -0.001 | 0.012 | 0.908 |
|  | rs7685296 | C | T | 0.051 | 0.008 | 2.32E-10 | -0.009 | 0.013 | 0.479 |
|  | rs7756992 | A | G | -0.130 | 0.008 | 6.00E-62 | -0.010 | 0.013 | 0.489 |
|  | rs7786095 | A | G | 0.074 | 0.013 | 9.64E-09 | 0.007 | 0.020 | 0.711 |
|  | rs780094 | T | C | -0.069 | 0.007 | 5.16E-21 | 0.011 | 0.012 | 0.354 |
|  | rs7845219 | C | T | -0.042 | 0.007 | 4.54E-09 | -0.011 | 0.012 | 0.366 |
|  | rs7903146 | C | T | -0.306 | 0.008 | 1.00E-200 | -0.001 | 0.013 | 0.899 |
|  | rs7929543 | A | C | -0.083 | 0.014 | 2.20E-09 | 0.005 | 0.020 | 0.834 |
|  | rs7955901 | C | T | 0.044 | 0.007 | 7.16E-10 | -0.017 | 0.012 | 0.144 |
|  | rs8068804 | A | G | 0.059 | 0.008 | 4.41E-14 | 0.020 | 0.013 | 0.110 |
|  | rs8108269 | G | T | 0.064 | 0.008 | 3.11E-16 | 0.017 | 0.013 | 0.172 |
|  | rs825476 | C | T | -0.052 | 0.007 | 6.80E-13 | 0.018 | 0.012 | 0.141 |
|  | rs840967 | A | C | -0.050 | 0.008 | 5.44E-10 | -0.005 | 0.012 | 0.727 |
|  | rs849135 | A | G | -0.100 | 0.007 | 1.04E-43 | 0.006 | 0.012 | 0.579 |
|  | rs853974 | C | T | -0.060 | 0.009 | 7.86E-12 | -0.009 | 0.013 | 0.548 |
|  | rs9369425 | A | G | -0.055 | 0.009 | 1.13E-10 | 0.004 | 0.013 | 0.712 |
|  | rs9894220 | A | G | 0.059 | 0.008 | 1.52E-13 | -0.004 | 0.012 | 0.687 |
|  | rs9928094 | A | G | -0.105 | 0.007 | 3.59E-47 | 0.011 | 0.012 | 0.376 |
|  | rs993380 | A | G | 0.051 | 0.008 | 4.59E-10 | -0.001 | 0.012 | 0.909 |
|  | rs9940149 | A | G | -0.058 | 0.010 | 9.29E-10 | -0.018 | 0.015 | 0.184 |
| *Rhodospirillaceae* | rs10077431 | A | C | -0.049 | 0.009 | 4.75E-08 | 0.000 | 0.019 | 0.928 |
|  | rs10087241 | A | G | -0.048 | 0.008 | 2.80E-09 | 0.017 | 0.016 | 0.280 |
|  | rs10100265 | A | C | 0.049 | 0.008 | 6.29E-10 | -0.015 | 0.016 | 0.315 |
|  | rs10114341 | C | T | -0.041 | 0.007 | 1.15E-08 | 0.010 | 0.015 | 0.524 |
|  | rs10401969 | C | T | 0.092 | 0.013 | 4.13E-12 | 0.001 | 0.029 | 0.933 |
|  | rs1050226 | G | A | -0.049 | 0.007 | 3.34E-11 | 0.018 | 0.016 | 0.245 |
|  | rs1061813 | A | G | -0.043 | 0.007 | 3.37E-09 | -0.013 | 0.015 | 0.394 |
|  | rs1063355 | T | G | -0.071 | 0.008 | 3.72E-19 | -0.008 | 0.016 | 0.642 |
|  | rs10740322 | A | G | 0.048 | 0.009 | 2.11E-08 | -0.012 | 0.016 | 0.439 |
|  | rs10811661 | C | T | -0.157 | 0.010 | 4.13E-58 | -0.025 | 0.021 | 0.258 |
|  | rs10842994 | C | T | 0.076 | 0.009 | 1.02E-16 | -0.022 | 0.019 | 0.242 |
|  | rs10974438 | A | C | -0.059 | 0.008 | 3.01E-15 | -0.022 | 0.016 | 0.173 |
|  | rs11098676 | C | T | 0.054 | 0.010 | 2.03E-08 | -0.027 | 0.018 | 0.164 |
|  | rs11107116 | G | T | -0.047 | 0.009 | 3.75E-08 | -0.008 | 0.019 | 0.645 |
|  | rs1111875 | T | C | -0.095 | 0.007 | 3.61E-39 | 0.005 | 0.016 | 0.771 |
|  | rs11257655 | C | T | -0.074 | 0.009 | 1.97E-17 | 0.048 | 0.019 | 0.013 |
|  | rs1127655 | C | T | 0.044 | 0.008 | 2.47E-08 | -0.028 | 0.015 | 0.064 |
|  | rs11708067 | A | G | 0.097 | 0.009 | 5.93E-29 | -0.009 | 0.018 | 0.680 |
|  | rs11925227 | A | G | -0.053 | 0.010 | 2.25E-08 | 0.010 | 0.020 | 0.598 |
|  | rs11926707 | C | T | 0.046 | 0.008 | 1.69E-08 | 0.053 | 0.016 | 0.001 |
|  | rs12088739 | A | G | 0.088 | 0.013 | 9.79E-12 | -0.018 | 0.027 | 0.547 |
|  | rs12299509 | A | G | -0.047 | 0.007 | 2.09E-10 | -0.004 | 0.015 | 0.773 |
|  | rs12617659 | C | T | 0.069 | 0.010 | 2.83E-11 | 0.002 | 0.022 | 0.964 |
|  | rs12910825 | A | G | -0.052 | 0.007 | 2.16E-12 | -0.032 | 0.016 | 0.047 |
|  | rs12945601 | C | T | -0.048 | 0.008 | 1.72E-09 | -0.005 | 0.016 | 0.747 |
|  | rs12970134 | A | G | 0.056 | 0.008 | 5.31E-12 | -0.010 | 0.017 | 0.580 |
|  | rs13239186 | C | T | -0.054 | 0.009 | 2.70E-10 | 0.000 | 0.017 | 0.994 |
|  | rs13330951 | A | G | 0.046 | 0.008 | 1.54E-08 | 0.032 | 0.015 | 0.036 |
|  | rs13389219 | C | T | 0.072 | 0.007 | 2.11E-22 | 0.023 | 0.016 | 0.144 |
|  | rs1359790 | G | A | 0.080 | 0.008 | 2.80E-23 | -0.004 | 0.018 | 0.873 |
|  | rs1496653 | G | A | -0.077 | 0.009 | 2.57E-18 | 0.025 | 0.019 | 0.170 |
|  | rs1552224 | C | A | -0.103 | 0.010 | 8.64E-25 | -0.017 | 0.021 | 0.411 |
|  | rs16988333 | A | G | 0.075 | 0.013 | 9.17E-09 | 0.011 | 0.030 | 0.737 |
|  | rs17086692 | G | T | 0.047 | 0.008 | 2.48E-08 | 0.001 | 0.016 | 0.986 |
|  | rs17168486 | C | T | -0.074 | 0.009 | 2.18E-15 | -0.019 | 0.020 | 0.332 |
|  | rs17405722 | A | G | 0.087 | 0.015 | 2.28E-09 | -0.013 | 0.030 | 0.637 |
|  | rs17631783 | C | T | 0.049 | 0.009 | 3.95E-08 | -0.023 | 0.017 | 0.178 |
|  | rs17791513 | A | G | 0.103 | 0.015 | 4.61E-12 | 0.033 | 0.031 | 0.309 |
|  | rs1801214 | C | T | 0.090 | 0.007 | 5.52E-34 | -0.019 | 0.016 | 0.239 |
|  | rs1899951 | T | C | -0.112 | 0.011 | 1.64E-24 | -0.003 | 0.022 | 0.949 |
|  | rs2237892 | C | T | 0.096 | 0.016 | 8.75E-10 | -0.025 | 0.035 | 0.456 |
|  | rs2246618 | C | T | -0.051 | 0.008 | 1.20E-09 | 0.052 | 0.016 | 0.002 |
|  | rs2261181 | C | T | -0.099 | 0.012 | 9.18E-17 | -0.029 | 0.026 | 0.423 |
|  | rs2294120 | G | A | -0.044 | 0.008 | 1.62E-08 | 0.025 | 0.015 | 0.108 |
|  | rs2296173 | A | G | -0.065 | 0.009 | 7.66E-14 | 0.010 | 0.019 | 0.601 |
|  | rs2299383 | C | T | -0.041 | 0.007 | 1.49E-08 | -0.017 | 0.015 | 0.274 |
|  | rs243019 | T | C | -0.057 | 0.007 | 2.29E-15 | 0.003 | 0.015 | 0.863 |
|  | rs2493394 | G | A | 0.073 | 0.011 | 1.15E-10 | 0.028 | 0.024 | 0.243 |
|  | rs2796441 | G | A | 0.072 | 0.007 | 1.96E-22 | -0.010 | 0.016 | 0.528 |
|  | rs2820426 | G | A | 0.052 | 0.007 | 1.30E-12 | 0.005 | 0.016 | 0.754 |
|  | rs2867125 | T | C | -0.060 | 0.010 | 4.33E-10 | -0.013 | 0.020 | 0.556 |
|  | rs2908282 | G | A | -0.055 | 0.009 | 4.25E-09 | 0.015 | 0.020 | 0.488 |
|  | rs2925979 | T | C | 0.053 | 0.008 | 9.06E-12 | 0.009 | 0.017 | 0.560 |
|  | rs2943656 | A | G | -0.090 | 0.007 | 6.70E-34 | -0.013 | 0.016 | 0.430 |
|  | rs3217992 | T | C | 0.053 | 0.007 | 7.23E-13 | 0.012 | 0.016 | 0.428 |
|  | rs340874 | T | C | -0.063 | 0.007 | 8.41E-18 | -0.028 | 0.016 | 0.072 |
|  | rs348330 | G | A | 0.049 | 0.008 | 1.86E-09 | -0.042 | 0.016 | 0.010 |
|  | rs3756784 | G | T | 0.051 | 0.009 | 2.59E-08 | -0.011 | 0.019 | 0.539 |
|  | rs3802177 | G | A | 0.122 | 0.008 | 2.32E-52 | 0.014 | 0.017 | 0.442 |
|  | rs459193 | G | A | 0.071 | 0.008 | 8.81E-18 | -0.002 | 0.018 | 0.928 |
|  | rs4622883 | A | G | 0.044 | 0.008 | 3.02E-08 | -0.007 | 0.015 | 0.654 |
|  | rs4686471 | C | T | 0.053 | 0.008 | 4.28E-11 | 0.019 | 0.016 | 0.237 |
|  | rs4812829 | A | G | 0.053 | 0.010 | 2.44E-08 | -0.041 | 0.021 | 0.043 |
|  | rs4823182 | A | G | -0.048 | 0.008 | 3.36E-10 | -0.021 | 0.016 | 0.190 |
|  | rs4865796 | A | G | 0.053 | 0.008 | 1.33E-11 | -0.006 | 0.017 | 0.646 |
|  | rs516946 | T | C | -0.082 | 0.009 | 3.16E-22 | 0.006 | 0.018 | 0.712 |
|  | rs5215 | C | T | 0.068 | 0.007 | 2.09E-20 | -0.006 | 0.016 | 0.744 |
|  | rs576674 | A | G | -0.065 | 0.010 | 1.79E-11 | 0.038 | 0.021 | 0.056 |
|  | rs6059662 | A | G | -0.045 | 0.008 | 1.51E-08 | -0.002 | 0.017 | 0.923 |
|  | rs61953351 | G | T | 0.070 | 0.009 | 1.98E-14 | 0.013 | 0.018 | 0.461 |
|  | rs622217 | C | T | -0.049 | 0.008 | 3.13E-10 | -0.021 | 0.015 | 0.168 |
|  | rs6515236 | A | C | 0.050 | 0.009 | 3.34E-08 | -0.027 | 0.018 | 0.127 |
|  | rs67232546 | C | T | -0.060 | 0.010 | 4.66E-10 | 0.009 | 0.020 | 0.612 |
|  | rs6767484 | A | G | -0.121 | 0.008 | 2.70E-56 | 0.003 | 0.017 | 0.856 |
|  | rs6785040 | C | T | -0.063 | 0.011 | 1.26E-08 | 0.010 | 0.021 | 0.640 |
|  | rs6795735 | C | T | 0.056 | 0.007 | 1.63E-14 | 0.010 | 0.016 | 0.531 |
|  | rs6878122 | A | G | -0.056 | 0.008 | 1.19E-12 | 0.014 | 0.017 | 0.392 |
|  | rs6960043 | C | T | 0.064 | 0.007 | 3.61E-19 | -0.003 | 0.015 | 0.810 |
|  | rs7144011 | G | T | -0.048 | 0.009 | 1.64E-08 | -0.004 | 0.019 | 0.850 |
|  | rs7177055 | A | G | 0.065 | 0.008 | 2.75E-16 | -0.015 | 0.017 | 0.392 |
|  | rs7240767 | C | T | 0.045 | 0.008 | 2.16E-08 | 0.015 | 0.016 | 0.347 |
|  | rs72892910 | G | T | -0.065 | 0.010 | 6.43E-11 | 0.010 | 0.020 | 0.593 |
|  | rs735949 | C | T | -0.071 | 0.011 | 1.95E-11 | -0.022 | 0.022 | 0.312 |
|  | rs753270 | C | T | 0.053 | 0.008 | 2.70E-11 | 0.008 | 0.016 | 0.601 |
|  | rs7561798 | A | G | -0.040 | 0.007 | 2.79E-08 | 0.001 | 0.015 | 0.947 |
|  | rs7572970 | A | G | -0.059 | 0.009 | 1.39E-11 | -0.020 | 0.017 | 0.288 |
|  | rs7607777 | G | T | 0.137 | 0.013 | 9.40E-28 | 0.029 | 0.025 | 0.252 |
|  | rs7674212 | G | T | 0.047 | 0.008 | 6.18E-10 | -0.013 | 0.016 | 0.406 |
|  | rs7685296 | C | T | 0.051 | 0.008 | 2.32E-10 | 0.027 | 0.017 | 0.127 |
|  | rs7756992 | A | G | -0.130 | 0.008 | 6.00E-62 | -0.001 | 0.017 | 0.918 |
|  | rs7786095 | A | G | 0.074 | 0.013 | 9.64E-09 | -0.013 | 0.026 | 0.634 |
|  | rs780094 | T | C | -0.069 | 0.007 | 5.16E-21 | 0.044 | 0.016 | 0.005 |
|  | rs7845219 | C | T | -0.042 | 0.007 | 4.54E-09 | -0.017 | 0.015 | 0.263 |
|  | rs7903146 | C | T | -0.306 | 0.008 | 1.00E-200 | 0.018 | 0.017 | 0.296 |
|  | rs7929543 | A | C | -0.083 | 0.014 | 2.20E-09 | -0.027 | 0.027 | 0.356 |
|  | rs7955901 | C | T | 0.044 | 0.007 | 7.16E-10 | -0.004 | 0.015 | 0.816 |
|  | rs8068804 | A | G | 0.059 | 0.008 | 4.41E-14 | -0.003 | 0.017 | 0.892 |
|  | rs8108269 | G | T | 0.064 | 0.008 | 3.11E-16 | 0.022 | 0.017 | 0.181 |
|  | rs825476 | C | T | -0.052 | 0.007 | 6.80E-13 | -0.006 | 0.016 | 0.716 |
|  | rs840967 | A | C | -0.050 | 0.008 | 5.44E-10 | 0.030 | 0.016 | 0.053 |
|  | rs849135 | A | G | -0.100 | 0.007 | 1.04E-43 | -0.048 | 0.015 | 0.002 |
|  | rs853974 | C | T | -0.060 | 0.009 | 7.86E-12 | -0.032 | 0.017 | 0.062 |
|  | rs9369425 | A | G | -0.055 | 0.009 | 1.13E-10 | -0.004 | 0.017 | 0.779 |
|  | rs9894220 | A | G | 0.059 | 0.008 | 1.52E-13 | 0.018 | 0.016 | 0.254 |
|  | rs9928094 | A | G | -0.105 | 0.007 | 3.59E-47 | -0.012 | 0.015 | 0.447 |
|  | rs993380 | A | G | 0.051 | 0.008 | 4.59E-10 | -0.012 | 0.016 | 0.456 |
|  | rs9940149 | A | G | -0.058 | 0.010 | 9.29E-10 | -0.007 | 0.020 | 0.665 |
| *Rikenellaceae* | rs10077431 | A | C | -0.049 | 0.009 | 4.75E-08 | -0.010 | 0.013 | 0.463 |
|  | rs10087241 | A | G | -0.048 | 0.008 | 2.80E-09 | 0.019 | 0.011 | 0.106 |
|  | rs10100265 | A | C | 0.049 | 0.008 | 6.29E-10 | -0.010 | 0.011 | 0.340 |
|  | rs10114341 | C | T | -0.041 | 0.007 | 1.15E-08 | 0.008 | 0.011 | 0.472 |
|  | rs10401969 | C | T | 0.092 | 0.013 | 4.13E-12 | -0.025 | 0.020 | 0.200 |
|  | rs1050226 | G | A | -0.049 | 0.007 | 3.34E-11 | -0.011 | 0.011 | 0.323 |
|  | rs1061813 | A | G | -0.043 | 0.007 | 3.37E-09 | 0.007 | 0.011 | 0.521 |
|  | rs1063355 | T | G | -0.071 | 0.008 | 3.72E-19 | 0.006 | 0.011 | 0.552 |
|  | rs10740322 | A | G | 0.048 | 0.009 | 2.11E-08 | 0.005 | 0.011 | 0.710 |
|  | rs10811661 | C | T | -0.157 | 0.010 | 4.13E-58 | 0.000 | 0.014 | 0.873 |
|  | rs10842994 | C | T | 0.076 | 0.009 | 1.02E-16 | 0.007 | 0.013 | 0.621 |
|  | rs10974438 | A | C | -0.059 | 0.008 | 3.01E-15 | 0.010 | 0.011 | 0.341 |
|  | rs11098676 | C | T | 0.054 | 0.010 | 2.03E-08 | 0.004 | 0.013 | 0.715 |
|  | rs11107116 | G | T | -0.047 | 0.009 | 3.75E-08 | -0.012 | 0.013 | 0.340 |
|  | rs1111875 | T | C | -0.095 | 0.007 | 3.61E-39 | 0.013 | 0.011 | 0.226 |
|  | rs11257655 | C | T | -0.074 | 0.009 | 1.97E-17 | 0.027 | 0.013 | 0.041 |
|  | rs1127655 | C | T | 0.044 | 0.008 | 2.47E-08 | 0.006 | 0.011 | 0.585 |
|  | rs11708067 | A | G | 0.097 | 0.009 | 5.93E-29 | -0.010 | 0.013 | 0.471 |
|  | rs11925227 | A | G | -0.053 | 0.010 | 2.25E-08 | -0.003 | 0.014 | 0.808 |
|  | rs11926707 | C | T | 0.046 | 0.008 | 1.69E-08 | 0.019 | 0.011 | 0.084 |
|  | rs12088739 | A | G | 0.088 | 0.013 | 9.79E-12 | 0.005 | 0.019 | 0.766 |
|  | rs12299509 | A | G | -0.047 | 0.007 | 2.09E-10 | 0.019 | 0.011 | 0.071 |
|  | rs12617659 | C | T | 0.069 | 0.010 | 2.83E-11 | -0.009 | 0.015 | 0.560 |
|  | rs12910825 | A | G | -0.052 | 0.007 | 2.16E-12 | -0.001 | 0.011 | 0.911 |
|  | rs12945601 | C | T | -0.048 | 0.008 | 1.72E-09 | 0.003 | 0.011 | 0.754 |
|  | rs12970134 | A | G | 0.056 | 0.008 | 5.31E-12 | -0.003 | 0.012 | 0.819 |
|  | rs13239186 | C | T | -0.054 | 0.009 | 2.70E-10 | -0.005 | 0.012 | 0.698 |
|  | rs13330951 | A | G | 0.046 | 0.008 | 1.54E-08 | -0.009 | 0.011 | 0.437 |
|  | rs13389219 | C | T | 0.072 | 0.007 | 2.11E-22 | -0.004 | 0.011 | 0.555 |
|  | rs1359790 | G | A | 0.080 | 0.008 | 2.80E-23 | -0.015 | 0.012 | 0.198 |
|  | rs1496653 | G | A | -0.077 | 0.009 | 2.57E-18 | 0.005 | 0.013 | 0.666 |
|  | rs1552224 | C | A | -0.103 | 0.010 | 8.64E-25 | 0.011 | 0.015 | 0.456 |
|  | rs16988333 | A | G | 0.075 | 0.013 | 9.17E-09 | 0.008 | 0.021 | 0.609 |
|  | rs17086692 | G | T | 0.047 | 0.008 | 2.48E-08 | 0.006 | 0.011 | 0.570 |
|  | rs17168486 | C | T | -0.074 | 0.009 | 2.18E-15 | 0.000 | 0.014 | 0.999 |
|  | rs17405722 | A | G | 0.087 | 0.015 | 2.28E-09 | -0.043 | 0.021 | 0.049 |
|  | rs17631783 | C | T | 0.049 | 0.009 | 3.95E-08 | 0.003 | 0.012 | 0.886 |
|  | rs17791513 | A | G | 0.103 | 0.015 | 4.61E-12 | 0.032 | 0.022 | 0.136 |
|  | rs1801214 | C | T | 0.090 | 0.007 | 5.52E-34 | 0.009 | 0.011 | 0.496 |
|  | rs1899951 | T | C | -0.112 | 0.011 | 1.64E-24 | 0.007 | 0.016 | 0.767 |
|  | rs2237892 | C | T | 0.096 | 0.016 | 8.75E-10 | -0.051 | 0.022 | 0.055 |
|  | rs2246618 | C | T | -0.051 | 0.008 | 1.20E-09 | -0.012 | 0.011 | 0.265 |
|  | rs2261181 | C | T | -0.099 | 0.012 | 9.18E-17 | 0.002 | 0.018 | 0.869 |
|  | rs2294120 | G | A | -0.044 | 0.008 | 1.62E-08 | -0.003 | 0.011 | 0.797 |
|  | rs2296173 | A | G | -0.065 | 0.009 | 7.66E-14 | -0.011 | 0.013 | 0.397 |
|  | rs2299383 | C | T | -0.041 | 0.007 | 1.49E-08 | -0.007 | 0.011 | 0.505 |
|  | rs243019 | T | C | -0.057 | 0.007 | 2.29E-15 | -0.003 | 0.011 | 0.761 |
|  | rs2493394 | G | A | 0.073 | 0.011 | 1.15E-10 | 0.025 | 0.017 | 0.202 |
|  | rs2796441 | G | A | 0.072 | 0.007 | 1.96E-22 | 0.012 | 0.011 | 0.277 |
|  | rs2820426 | G | A | 0.052 | 0.007 | 1.30E-12 | 0.003 | 0.011 | 0.808 |
|  | rs2867125 | T | C | -0.060 | 0.010 | 4.33E-10 | -0.013 | 0.014 | 0.421 |
|  | rs2908282 | G | A | -0.055 | 0.009 | 4.25E-09 | 0.011 | 0.014 | 0.440 |
|  | rs2925979 | T | C | 0.053 | 0.008 | 9.06E-12 | 0.008 | 0.012 | 0.454 |
|  | rs2943656 | A | G | -0.090 | 0.007 | 6.70E-34 | 0.028 | 0.011 | 0.010 |
|  | rs3217992 | T | C | 0.053 | 0.007 | 7.23E-13 | -0.002 | 0.011 | 0.829 |
|  | rs340874 | T | C | -0.063 | 0.007 | 8.41E-18 | -0.013 | 0.011 | 0.222 |
|  | rs348330 | G | A | 0.049 | 0.008 | 1.86E-09 | 0.000 | 0.011 | 0.994 |
|  | rs3756784 | G | T | 0.051 | 0.009 | 2.59E-08 | -0.017 | 0.013 | 0.213 |
|  | rs3802177 | G | A | 0.122 | 0.008 | 2.32E-52 | -0.010 | 0.012 | 0.413 |
|  | rs459193 | G | A | 0.071 | 0.008 | 8.81E-18 | -0.021 | 0.012 | 0.087 |
|  | rs4622883 | A | G | 0.044 | 0.008 | 3.02E-08 | 0.014 | 0.011 | 0.200 |
|  | rs4686471 | C | T | 0.053 | 0.008 | 4.28E-11 | -0.015 | 0.011 | 0.127 |
|  | rs4812829 | A | G | 0.053 | 0.010 | 2.44E-08 | -0.035 | 0.014 | 0.012 |
|  | rs4823182 | A | G | -0.048 | 0.008 | 3.36E-10 | -0.007 | 0.011 | 0.498 |
|  | rs4865796 | A | G | 0.053 | 0.008 | 1.33E-11 | -0.002 | 0.012 | 0.926 |
|  | rs516946 | T | C | -0.082 | 0.009 | 3.16E-22 | 0.007 | 0.013 | 0.581 |
|  | rs5215 | C | T | 0.068 | 0.007 | 2.09E-20 | -0.009 | 0.011 | 0.433 |
|  | rs576674 | A | G | -0.065 | 0.010 | 1.79E-11 | 0.014 | 0.014 | 0.335 |
|  | rs6059662 | A | G | -0.045 | 0.008 | 1.51E-08 | 0.026 | 0.012 | 0.027 |
|  | rs61953351 | G | T | 0.070 | 0.009 | 1.98E-14 | -0.018 | 0.012 | 0.120 |
|  | rs622217 | C | T | -0.049 | 0.008 | 3.13E-10 | -0.007 | 0.011 | 0.517 |
|  | rs6515236 | A | C | 0.050 | 0.009 | 3.34E-08 | -0.014 | 0.012 | 0.226 |
|  | rs67232546 | C | T | -0.060 | 0.010 | 4.66E-10 | -0.013 | 0.013 | 0.335 |
|  | rs6767484 | A | G | -0.121 | 0.008 | 2.70E-56 | -0.006 | 0.011 | 0.590 |
|  | rs6785040 | C | T | -0.063 | 0.011 | 1.26E-08 | 0.005 | 0.014 | 0.740 |
|  | rs6795735 | C | T | 0.056 | 0.007 | 1.63E-14 | 0.022 | 0.011 | 0.040 |
|  | rs6878122 | A | G | -0.056 | 0.008 | 1.19E-12 | 0.011 | 0.012 | 0.337 |
|  | rs6960043 | C | T | 0.064 | 0.007 | 3.61E-19 | 0.006 | 0.011 | 0.564 |
|  | rs7144011 | G | T | -0.048 | 0.009 | 1.64E-08 | -0.014 | 0.013 | 0.275 |
|  | rs7177055 | A | G | 0.065 | 0.008 | 2.75E-16 | -0.006 | 0.012 | 0.558 |
|  | rs7240767 | C | T | 0.045 | 0.008 | 2.16E-08 | -0.001 | 0.011 | 0.904 |
|  | rs72892910 | G | T | -0.065 | 0.010 | 6.43E-11 | -0.012 | 0.014 | 0.406 |
|  | rs735949 | C | T | -0.071 | 0.011 | 1.95E-11 | 0.001 | 0.016 | 0.900 |
|  | rs753270 | C | T | 0.053 | 0.008 | 2.70E-11 | 0.008 | 0.011 | 0.432 |
|  | rs7561798 | A | G | -0.040 | 0.007 | 2.79E-08 | -0.002 | 0.011 | 0.857 |
|  | rs7572970 | A | G | -0.059 | 0.009 | 1.39E-11 | -0.011 | 0.012 | 0.332 |
|  | rs7607777 | G | T | 0.137 | 0.013 | 9.40E-28 | -0.007 | 0.018 | 0.714 |
|  | rs7674212 | G | T | 0.047 | 0.008 | 6.18E-10 | -0.001 | 0.011 | 0.952 |
|  | rs7685296 | C | T | 0.051 | 0.008 | 2.32E-10 | 0.025 | 0.012 | 0.035 |
|  | rs7729395 | C | T | -0.137 | 0.016 | 1.10E-17 | 0.028 | 0.056 | 0.601 |
|  | rs7756992 | A | G | -0.130 | 0.008 | 6.00E-62 | -0.012 | 0.012 | 0.271 |
|  | rs7786095 | A | G | 0.074 | 0.013 | 9.64E-09 | 0.002 | 0.018 | 0.913 |
|  | rs780094 | T | C | -0.069 | 0.007 | 5.16E-21 | 0.018 | 0.011 | 0.100 |
|  | rs7845219 | C | T | -0.042 | 0.007 | 4.54E-09 | 0.017 | 0.011 | 0.116 |
|  | rs7903146 | C | T | -0.306 | 0.008 | 1.00E-200 | 0.008 | 0.012 | 0.530 |
|  | rs7929543 | A | C | -0.083 | 0.014 | 2.20E-09 | -0.008 | 0.018 | 0.623 |
|  | rs7955901 | C | T | 0.044 | 0.007 | 7.16E-10 | 0.004 | 0.011 | 0.671 |
|  | rs8068804 | A | G | 0.059 | 0.008 | 4.41E-14 | -0.010 | 0.012 | 0.417 |
|  | rs8108269 | G | T | 0.064 | 0.008 | 3.11E-16 | -0.002 | 0.012 | 0.876 |
|  | rs825476 | C | T | -0.052 | 0.007 | 6.80E-13 | -0.004 | 0.011 | 0.688 |
|  | rs840967 | A | C | -0.050 | 0.008 | 5.44E-10 | 0.003 | 0.011 | 0.834 |
|  | rs849135 | A | G | -0.100 | 0.007 | 1.04E-43 | -0.001 | 0.011 | 0.942 |
|  | rs853974 | C | T | -0.060 | 0.009 | 7.86E-12 | -0.009 | 0.012 | 0.431 |
|  | rs9369425 | A | G | -0.055 | 0.009 | 1.13E-10 | -0.003 | 0.012 | 0.839 |
|  | rs9894220 | A | G | 0.059 | 0.008 | 1.52E-13 | 0.014 | 0.011 | 0.193 |
|  | rs9928094 | A | G | -0.105 | 0.007 | 3.59E-47 | -0.010 | 0.011 | 0.365 |
|  | rs993380 | A | G | 0.051 | 0.008 | 4.59E-10 | -0.003 | 0.011 | 0.788 |
|  | rs9940149 | A | G | -0.058 | 0.010 | 9.29E-10 | 0.004 | 0.014 | 0.687 |
| *Ruminococcaceae* | rs10077431 | A | C | -0.049 | 0.009 | 4.75E-08 | -0.013 | 0.013 | 0.359 |
|  | rs10087241 | A | G | -0.048 | 0.008 | 2.80E-09 | -0.015 | 0.011 | 0.152 |
|  | rs10100265 | A | C | 0.049 | 0.008 | 6.29E-10 | -0.006 | 0.011 | 0.541 |
|  | rs10114341 | C | T | -0.041 | 0.007 | 1.15E-08 | -0.001 | 0.011 | 0.968 |
|  | rs10401969 | C | T | 0.092 | 0.013 | 4.13E-12 | 0.014 | 0.020 | 0.489 |
|  | rs1050226 | G | A | -0.049 | 0.007 | 3.34E-11 | -0.003 | 0.011 | 0.788 |
|  | rs1061813 | A | G | -0.043 | 0.007 | 3.37E-09 | -0.011 | 0.011 | 0.341 |
|  | rs1063355 | T | G | -0.071 | 0.008 | 3.72E-19 | 0.003 | 0.011 | 0.789 |
|  | rs10740322 | A | G | 0.048 | 0.009 | 2.11E-08 | -0.007 | 0.011 | 0.529 |
|  | rs10811661 | C | T | -0.157 | 0.010 | 4.13E-58 | -0.003 | 0.014 | 0.877 |
|  | rs10842994 | C | T | 0.076 | 0.009 | 1.02E-16 | -0.017 | 0.013 | 0.185 |
|  | rs10974438 | A | C | -0.059 | 0.008 | 3.01E-15 | -0.012 | 0.011 | 0.285 |
|  | rs11098676 | C | T | 0.054 | 0.010 | 2.03E-08 | -0.011 | 0.013 | 0.467 |
|  | rs11107116 | G | T | -0.047 | 0.009 | 3.75E-08 | 0.015 | 0.013 | 0.241 |
|  | rs1111875 | T | C | -0.095 | 0.007 | 3.61E-39 | -0.017 | 0.011 | 0.115 |
|  | rs11257655 | C | T | -0.074 | 0.009 | 1.97E-17 | -0.003 | 0.013 | 0.804 |
|  | rs1127655 | C | T | 0.044 | 0.008 | 2.47E-08 | 0.000 | 0.010 | 0.987 |
|  | rs11708067 | A | G | 0.097 | 0.009 | 5.93E-29 | -0.014 | 0.013 | 0.276 |
|  | rs11925227 | A | G | -0.053 | 0.010 | 2.25E-08 | -0.009 | 0.014 | 0.486 |
|  | rs11926707 | C | T | 0.046 | 0.008 | 1.69E-08 | 0.016 | 0.011 | 0.145 |
|  | rs12088739 | A | G | 0.088 | 0.013 | 9.79E-12 | 0.033 | 0.018 | 0.073 |
|  | rs12299509 | A | G | -0.047 | 0.007 | 2.09E-10 | 0.008 | 0.010 | 0.403 |
|  | rs12617659 | C | T | 0.069 | 0.010 | 2.83E-11 | -0.008 | 0.015 | 0.571 |
|  | rs12910825 | A | G | -0.052 | 0.007 | 2.16E-12 | -0.005 | 0.011 | 0.646 |
|  | rs12945601 | C | T | -0.048 | 0.008 | 1.72E-09 | 0.016 | 0.011 | 0.124 |
|  | rs12970134 | A | G | 0.056 | 0.008 | 5.31E-12 | -0.006 | 0.012 | 0.580 |
|  | rs13239186 | C | T | -0.054 | 0.009 | 2.70E-10 | -0.002 | 0.011 | 0.826 |
|  | rs13330951 | A | G | 0.046 | 0.008 | 1.54E-08 | -0.008 | 0.011 | 0.431 |
|  | rs13389219 | C | T | 0.072 | 0.007 | 2.11E-22 | 0.000 | 0.011 | 1.000 |
|  | rs1359790 | G | A | 0.080 | 0.008 | 2.80E-23 | -0.005 | 0.012 | 0.611 |
|  | rs1496653 | G | A | -0.077 | 0.009 | 2.57E-18 | 0.011 | 0.013 | 0.342 |
|  | rs1552224 | C | A | -0.103 | 0.010 | 8.64E-25 | 0.000 | 0.015 | 0.990 |
|  | rs16988333 | A | G | 0.075 | 0.013 | 9.17E-09 | -0.018 | 0.021 | 0.345 |
|  | rs17086692 | G | T | 0.047 | 0.008 | 2.48E-08 | -0.008 | 0.011 | 0.498 |
|  | rs17168486 | C | T | -0.074 | 0.009 | 2.18E-15 | -0.002 | 0.013 | 0.856 |
|  | rs17405722 | A | G | 0.087 | 0.015 | 2.28E-09 | -0.002 | 0.021 | 0.957 |
|  | rs17631783 | C | T | 0.049 | 0.009 | 3.95E-08 | -0.002 | 0.012 | 0.693 |
|  | rs17791513 | A | G | 0.103 | 0.015 | 4.61E-12 | 0.029 | 0.021 | 0.177 |
|  | rs1801214 | C | T | 0.090 | 0.007 | 5.52E-34 | 0.012 | 0.011 | 0.243 |
|  | rs1899951 | T | C | -0.112 | 0.011 | 1.64E-24 | 0.018 | 0.015 | 0.301 |
|  | rs2237892 | C | T | 0.096 | 0.016 | 8.75E-10 | 0.009 | 0.021 | 0.469 |
|  | rs2246618 | C | T | -0.051 | 0.008 | 1.20E-09 | -0.008 | 0.011 | 0.454 |
|  | rs2261181 | C | T | -0.099 | 0.012 | 9.18E-17 | -0.029 | 0.018 | 0.114 |
|  | rs2294120 | G | A | -0.044 | 0.008 | 1.62E-08 | 0.000 | 0.010 | 0.960 |
|  | rs2296173 | A | G | -0.065 | 0.009 | 7.66E-14 | -0.013 | 0.013 | 0.306 |
|  | rs2299383 | C | T | -0.041 | 0.007 | 1.49E-08 | -0.016 | 0.011 | 0.135 |
|  | rs243019 | T | C | -0.057 | 0.007 | 2.29E-15 | -0.014 | 0.011 | 0.184 |
|  | rs2493394 | G | A | 0.073 | 0.011 | 1.15E-10 | 0.039 | 0.017 | 0.022 |
|  | rs2796441 | G | A | 0.072 | 0.007 | 1.96E-22 | 0.005 | 0.011 | 0.665 |
|  | rs2820426 | G | A | 0.052 | 0.007 | 1.30E-12 | 0.000 | 0.011 | 0.976 |
|  | rs2867125 | T | C | -0.060 | 0.010 | 4.33E-10 | -0.008 | 0.014 | 0.671 |
|  | rs2908282 | G | A | -0.055 | 0.009 | 4.25E-09 | 0.003 | 0.014 | 0.854 |
|  | rs2925979 | T | C | 0.053 | 0.008 | 9.06E-12 | 0.012 | 0.011 | 0.305 |
|  | rs2943656 | A | G | -0.090 | 0.007 | 6.70E-34 | -0.006 | 0.011 | 0.542 |
|  | rs3217992 | T | C | 0.053 | 0.007 | 7.23E-13 | 0.001 | 0.011 | 0.958 |
|  | rs340874 | T | C | -0.063 | 0.007 | 8.41E-18 | -0.006 | 0.011 | 0.566 |
|  | rs348330 | G | A | 0.049 | 0.008 | 1.86E-09 | 0.020 | 0.011 | 0.077 |
|  | rs3756784 | G | T | 0.051 | 0.009 | 2.59E-08 | 0.015 | 0.013 | 0.256 |
|  | rs3802177 | G | A | 0.122 | 0.008 | 2.32E-52 | 0.003 | 0.011 | 0.847 |
|  | rs459193 | G | A | 0.071 | 0.008 | 8.81E-18 | 0.014 | 0.012 | 0.274 |
|  | rs4622883 | A | G | 0.044 | 0.008 | 3.02E-08 | 0.007 | 0.011 | 0.523 |
|  | rs4686471 | C | T | 0.053 | 0.008 | 4.28E-11 | -0.008 | 0.011 | 0.562 |
|  | rs4812829 | A | G | 0.053 | 0.010 | 2.44E-08 | 0.032 | 0.014 | 0.019 |
|  | rs4823182 | A | G | -0.048 | 0.008 | 3.36E-10 | -0.013 | 0.011 | 0.239 |
|  | rs4865796 | A | G | 0.053 | 0.008 | 1.33E-11 | -0.003 | 0.011 | 0.859 |
|  | rs516946 | T | C | -0.082 | 0.009 | 3.16E-22 | -0.005 | 0.012 | 0.612 |
|  | rs5215 | C | T | 0.068 | 0.007 | 2.09E-20 | 0.006 | 0.011 | 0.556 |
|  | rs576674 | A | G | -0.065 | 0.010 | 1.79E-11 | -0.003 | 0.014 | 0.694 |
|  | rs6059662 | A | G | -0.045 | 0.008 | 1.51E-08 | -0.002 | 0.012 | 0.862 |
|  | rs61953351 | G | T | 0.070 | 0.009 | 1.98E-14 | -0.018 | 0.012 | 0.139 |
|  | rs622217 | C | T | -0.049 | 0.008 | 3.13E-10 | -0.021 | 0.010 | 0.042 |
|  | rs6515236 | A | C | 0.050 | 0.009 | 3.34E-08 | -0.011 | 0.012 | 0.322 |
|  | rs67232546 | C | T | -0.060 | 0.010 | 4.66E-10 | -0.019 | 0.013 | 0.152 |
|  | rs6767484 | A | G | -0.121 | 0.008 | 2.70E-56 | 0.007 | 0.011 | 0.515 |
|  | rs6785040 | C | T | -0.063 | 0.011 | 1.26E-08 | 0.011 | 0.014 | 0.360 |
|  | rs6795735 | C | T | 0.056 | 0.007 | 1.63E-14 | 0.005 | 0.011 | 0.606 |
|  | rs6878122 | A | G | -0.056 | 0.008 | 1.19E-12 | 0.016 | 0.012 | 0.159 |
|  | rs6960043 | C | T | 0.064 | 0.007 | 3.61E-19 | -0.018 | 0.010 | 0.087 |
|  | rs7144011 | G | T | -0.048 | 0.009 | 1.64E-08 | 0.000 | 0.013 | 0.998 |
|  | rs7177055 | A | G | 0.065 | 0.008 | 2.75E-16 | 0.007 | 0.011 | 0.552 |
|  | rs7240767 | C | T | 0.045 | 0.008 | 2.16E-08 | 0.007 | 0.011 | 0.552 |
|  | rs72892910 | G | T | -0.065 | 0.010 | 6.43E-11 | 0.016 | 0.014 | 0.220 |
|  | rs735949 | C | T | -0.071 | 0.011 | 1.95E-11 | 0.016 | 0.016 | 0.326 |
|  | rs753270 | C | T | 0.053 | 0.008 | 2.70E-11 | -0.014 | 0.011 | 0.182 |
|  | rs7561798 | A | G | -0.040 | 0.007 | 2.79E-08 | -0.019 | 0.010 | 0.070 |
|  | rs7572970 | A | G | -0.059 | 0.009 | 1.39E-11 | -0.014 | 0.012 | 0.230 |
|  | rs7607777 | G | T | 0.137 | 0.013 | 9.40E-28 | 0.032 | 0.017 | 0.064 |
|  | rs7674212 | G | T | 0.047 | 0.008 | 6.18E-10 | 0.010 | 0.011 | 0.382 |
|  | rs7685296 | C | T | 0.051 | 0.008 | 2.32E-10 | -0.015 | 0.012 | 0.185 |
|  | rs7729395 | C | T | -0.137 | 0.016 | 1.10E-17 | -0.021 | 0.056 | 0.654 |
|  | rs7756992 | A | G | -0.130 | 0.008 | 6.00E-62 | -0.012 | 0.011 | 0.293 |
|  | rs7786095 | A | G | 0.074 | 0.013 | 9.64E-09 | -0.017 | 0.018 | 0.401 |
|  | rs780094 | T | C | -0.069 | 0.007 | 5.16E-21 | 0.020 | 0.011 | 0.066 |
|  | rs7845219 | C | T | -0.042 | 0.007 | 4.54E-09 | -0.017 | 0.011 | 0.111 |
|  | rs7903146 | C | T | -0.306 | 0.008 | 1.00E-200 | 0.006 | 0.012 | 0.608 |
|  | rs7929543 | A | C | -0.083 | 0.014 | 2.20E-09 | 0.001 | 0.018 | 0.969 |
|  | rs7955901 | C | T | 0.044 | 0.007 | 7.16E-10 | -0.008 | 0.011 | 0.443 |
|  | rs8068804 | A | G | 0.059 | 0.008 | 4.41E-14 | -0.013 | 0.011 | 0.263 |
|  | rs8108269 | G | T | 0.064 | 0.008 | 3.11E-16 | -0.009 | 0.011 | 0.443 |
|  | rs825476 | C | T | -0.052 | 0.007 | 6.80E-13 | 0.000 | 0.011 | 0.996 |
|  | rs840967 | A | C | -0.050 | 0.008 | 5.44E-10 | -0.003 | 0.011 | 0.763 |
|  | rs849135 | A | G | -0.100 | 0.007 | 1.04E-43 | -0.020 | 0.011 | 0.057 |
|  | rs853974 | C | T | -0.060 | 0.009 | 7.86E-12 | -0.022 | 0.012 | 0.049 |
|  | rs9369425 | A | G | -0.055 | 0.009 | 1.13E-10 | -0.001 | 0.012 | 0.878 |
|  | rs9894220 | A | G | 0.059 | 0.008 | 1.52E-13 | -0.002 | 0.011 | 0.836 |
|  | rs9928094 | A | G | -0.105 | 0.007 | 3.59E-47 | 0.006 | 0.011 | 0.530 |
|  | rs993380 | A | G | 0.051 | 0.008 | 4.59E-10 | -0.002 | 0.011 | 0.855 |
|  | rs9940149 | A | G | -0.058 | 0.010 | 9.29E-10 | 0.014 | 0.013 | 0.257 |
| *Streptococcaceae* | rs10077431 | A | C | -0.049 | 0.009 | 4.75E-08 | 0.001 | 0.014 | 0.990 |
|  | rs10087241 | A | G | -0.048 | 0.008 | 2.80E-09 | -0.022 | 0.011 | 0.052 |
|  | rs10100265 | A | C | 0.049 | 0.008 | 6.29E-10 | -0.011 | 0.011 | 0.346 |
|  | rs10114341 | C | T | -0.041 | 0.007 | 1.15E-08 | -0.003 | 0.011 | 0.681 |
|  | rs10401969 | C | T | 0.092 | 0.013 | 4.13E-12 | 0.000 | 0.021 | 0.926 |
|  | rs1050226 | G | A | -0.049 | 0.007 | 3.34E-11 | 0.008 | 0.011 | 0.470 |
|  | rs1061813 | A | G | -0.043 | 0.007 | 3.37E-09 | 0.006 | 0.011 | 0.601 |
|  | rs1063355 | T | G | -0.071 | 0.008 | 3.72E-19 | 0.018 | 0.011 | 0.113 |
|  | rs10740322 | A | G | 0.048 | 0.009 | 2.11E-08 | 0.000 | 0.012 | 0.973 |
|  | rs10811661 | C | T | -0.157 | 0.010 | 4.13E-58 | 0.018 | 0.015 | 0.212 |
|  | rs10842994 | C | T | 0.076 | 0.009 | 1.02E-16 | 0.028 | 0.014 | 0.041 |
|  | rs10974438 | A | C | -0.059 | 0.008 | 3.01E-15 | -0.022 | 0.011 | 0.056 |
|  | rs11098676 | C | T | 0.054 | 0.010 | 2.03E-08 | 0.001 | 0.013 | 0.877 |
|  | rs11107116 | G | T | -0.047 | 0.009 | 3.75E-08 | -0.002 | 0.013 | 0.896 |
|  | rs1111875 | T | C | -0.095 | 0.007 | 3.61E-39 | -0.013 | 0.011 | 0.258 |
|  | rs11257655 | C | T | -0.074 | 0.009 | 1.97E-17 | -0.010 | 0.013 | 0.425 |
|  | rs1127655 | C | T | 0.044 | 0.008 | 2.47E-08 | -0.001 | 0.011 | 0.959 |
|  | rs11708067 | A | G | 0.097 | 0.009 | 5.93E-29 | 0.025 | 0.013 | 0.068 |
|  | rs11925227 | A | G | -0.053 | 0.010 | 2.25E-08 | 0.015 | 0.014 | 0.293 |
|  | rs11926707 | C | T | 0.046 | 0.008 | 1.69E-08 | 0.005 | 0.011 | 0.659 |
|  | rs12088739 | A | G | 0.088 | 0.013 | 9.79E-12 | 0.022 | 0.019 | 0.266 |
|  | rs12299509 | A | G | -0.047 | 0.007 | 2.09E-10 | 0.007 | 0.011 | 0.509 |
|  | rs12617659 | C | T | 0.069 | 0.010 | 2.83E-11 | 0.010 | 0.016 | 0.535 |
|  | rs12910825 | A | G | -0.052 | 0.007 | 2.16E-12 | -0.002 | 0.011 | 0.875 |
|  | rs12945601 | C | T | -0.048 | 0.008 | 1.72E-09 | 0.004 | 0.011 | 0.720 |
|  | rs12970134 | A | G | 0.056 | 0.008 | 5.31E-12 | -0.013 | 0.013 | 0.246 |
|  | rs13239186 | C | T | -0.054 | 0.009 | 2.70E-10 | -0.024 | 0.012 | 0.051 |
|  | rs13330951 | A | G | 0.046 | 0.008 | 1.54E-08 | -0.007 | 0.011 | 0.548 |
|  | rs13389219 | C | T | 0.072 | 0.007 | 2.11E-22 | 0.008 | 0.012 | 0.577 |
|  | rs1359790 | G | A | 0.080 | 0.008 | 2.80E-23 | -0.012 | 0.012 | 0.329 |
|  | rs1496653 | G | A | -0.077 | 0.009 | 2.57E-18 | -0.002 | 0.014 | 0.925 |
|  | rs1552224 | C | A | -0.103 | 0.010 | 8.64E-25 | -0.021 | 0.015 | 0.164 |
|  | rs16988333 | A | G | 0.075 | 0.013 | 9.17E-09 | -0.007 | 0.021 | 0.688 |
|  | rs17086692 | G | T | 0.047 | 0.008 | 2.48E-08 | 0.012 | 0.012 | 0.306 |
|  | rs17168486 | C | T | -0.074 | 0.009 | 2.18E-15 | -0.008 | 0.014 | 0.501 |
|  | rs17405722 | A | G | 0.087 | 0.015 | 2.28E-09 | 0.002 | 0.022 | 0.967 |
|  | rs17631783 | C | T | 0.049 | 0.009 | 3.95E-08 | 0.010 | 0.013 | 0.470 |
|  | rs17791513 | A | G | 0.103 | 0.015 | 4.61E-12 | -0.008 | 0.022 | 0.731 |
|  | rs1801214 | C | T | 0.090 | 0.007 | 5.52E-34 | -0.006 | 0.011 | 0.658 |
|  | rs1899951 | T | C | -0.112 | 0.011 | 1.64E-24 | -0.022 | 0.016 | 0.182 |
|  | rs2237892 | C | T | 0.096 | 0.016 | 8.75E-10 | 0.037 | 0.023 | 0.084 |
|  | rs2246618 | C | T | -0.051 | 0.008 | 1.20E-09 | 0.025 | 0.012 | 0.033 |
|  | rs2261181 | C | T | -0.099 | 0.012 | 9.18E-17 | -0.002 | 0.018 | 0.932 |
|  | rs2294120 | G | A | -0.044 | 0.008 | 1.62E-08 | 0.008 | 0.011 | 0.504 |
|  | rs2296173 | A | G | -0.065 | 0.009 | 7.66E-14 | -0.026 | 0.014 | 0.054 |
|  | rs2299383 | C | T | -0.041 | 0.007 | 1.49E-08 | 0.010 | 0.011 | 0.380 |
|  | rs243019 | T | C | -0.057 | 0.007 | 2.29E-15 | 0.010 | 0.011 | 0.360 |
|  | rs2493394 | G | A | 0.073 | 0.011 | 1.15E-10 | 0.010 | 0.018 | 0.505 |
|  | rs2796441 | G | A | 0.072 | 0.007 | 1.96E-22 | -0.012 | 0.011 | 0.291 |
|  | rs2820426 | G | A | 0.052 | 0.007 | 1.30E-12 | 0.010 | 0.011 | 0.396 |
|  | rs2867125 | T | C | -0.060 | 0.010 | 4.33E-10 | 0.015 | 0.015 | 0.314 |
|  | rs2908282 | G | A | -0.055 | 0.009 | 4.25E-09 | 0.000 | 0.015 | 0.971 |
|  | rs2925979 | T | C | 0.053 | 0.008 | 9.06E-12 | 0.006 | 0.012 | 0.584 |
|  | rs2943656 | A | G | -0.090 | 0.007 | 6.70E-34 | 0.006 | 0.012 | 0.657 |
|  | rs3217992 | T | C | 0.053 | 0.007 | 7.23E-13 | -0.001 | 0.011 | 0.927 |
|  | rs340874 | T | C | -0.063 | 0.007 | 8.41E-18 | 0.003 | 0.011 | 0.781 |
|  | rs348330 | G | A | 0.049 | 0.008 | 1.86E-09 | 0.005 | 0.012 | 0.640 |
|  | rs3756784 | G | T | 0.051 | 0.009 | 2.59E-08 | 0.004 | 0.014 | 0.759 |
|  | rs3802177 | G | A | 0.122 | 0.008 | 2.32E-52 | 0.008 | 0.012 | 0.531 |
|  | rs459193 | G | A | 0.071 | 0.008 | 8.81E-18 | -0.010 | 0.013 | 0.449 |
|  | rs4622883 | A | G | 0.044 | 0.008 | 3.02E-08 | 0.006 | 0.011 | 0.568 |
|  | rs4686471 | C | T | 0.053 | 0.008 | 4.28E-11 | 0.004 | 0.012 | 0.699 |
|  | rs4812829 | A | G | 0.053 | 0.010 | 2.44E-08 | 0.004 | 0.014 | 0.756 |
|  | rs4823182 | A | G | -0.048 | 0.008 | 3.36E-10 | -0.018 | 0.011 | 0.114 |
|  | rs4865796 | A | G | 0.053 | 0.008 | 1.33E-11 | -0.007 | 0.012 | 0.624 |
|  | rs516946 | T | C | -0.082 | 0.009 | 3.16E-22 | -0.001 | 0.013 | 0.871 |
|  | rs5215 | C | T | 0.068 | 0.007 | 2.09E-20 | 0.012 | 0.011 | 0.260 |
|  | rs576674 | A | G | -0.065 | 0.010 | 1.79E-11 | 0.015 | 0.015 | 0.262 |
|  | rs6059662 | A | G | -0.045 | 0.008 | 1.51E-08 | -0.009 | 0.012 | 0.478 |
|  | rs61953351 | G | T | 0.070 | 0.009 | 1.98E-14 | -0.016 | 0.013 | 0.189 |
|  | rs622217 | C | T | -0.049 | 0.008 | 3.13E-10 | -0.005 | 0.011 | 0.636 |
|  | rs6515236 | A | C | 0.050 | 0.009 | 3.34E-08 | 0.013 | 0.013 | 0.275 |
|  | rs67232546 | C | T | -0.060 | 0.010 | 4.66E-10 | 0.008 | 0.014 | 0.550 |
|  | rs6767484 | A | G | -0.121 | 0.008 | 2.70E-56 | 0.003 | 0.012 | 0.817 |
|  | rs6785040 | C | T | -0.063 | 0.011 | 1.26E-08 | 0.004 | 0.015 | 0.754 |
|  | rs6795735 | C | T | 0.056 | 0.007 | 1.63E-14 | -0.005 | 0.011 | 0.659 |
|  | rs6878122 | A | G | -0.056 | 0.008 | 1.19E-12 | 0.006 | 0.012 | 0.549 |
|  | rs6960043 | C | T | 0.064 | 0.007 | 3.61E-19 | -0.006 | 0.011 | 0.603 |
|  | rs7144011 | G | T | -0.048 | 0.009 | 1.64E-08 | 0.010 | 0.014 | 0.457 |
|  | rs7177055 | A | G | 0.065 | 0.008 | 2.75E-16 | -0.005 | 0.012 | 0.679 |
|  | rs7240767 | C | T | 0.045 | 0.008 | 2.16E-08 | -0.009 | 0.011 | 0.433 |
|  | rs72892910 | G | T | -0.065 | 0.010 | 6.43E-11 | 0.022 | 0.014 | 0.137 |
|  | rs735949 | C | T | -0.071 | 0.011 | 1.95E-11 | -0.007 | 0.016 | 0.678 |
|  | rs753270 | C | T | 0.053 | 0.008 | 2.70E-11 | -0.004 | 0.011 | 0.695 |
|  | rs7561798 | A | G | -0.040 | 0.007 | 2.79E-08 | -0.002 | 0.011 | 0.886 |
|  | rs7572970 | A | G | -0.059 | 0.009 | 1.39E-11 | 0.010 | 0.012 | 0.440 |
|  | rs7607777 | G | T | 0.137 | 0.013 | 9.40E-28 | 0.000 | 0.018 | 0.916 |
|  | rs7674212 | G | T | 0.047 | 0.008 | 6.18E-10 | 0.001 | 0.011 | 0.967 |
|  | rs7685296 | C | T | 0.051 | 0.008 | 2.32E-10 | -0.006 | 0.012 | 0.628 |
|  | rs7729395 | C | T | -0.137 | 0.016 | 1.10E-17 | -0.051 | 0.057 | 0.360 |
|  | rs7756992 | A | G | -0.130 | 0.008 | 6.00E-62 | 0.014 | 0.012 | 0.234 |
|  | rs7786095 | A | G | 0.074 | 0.013 | 9.64E-09 | -0.014 | 0.019 | 0.463 |
|  | rs780094 | T | C | -0.069 | 0.007 | 5.16E-21 | 0.009 | 0.011 | 0.399 |
|  | rs7845219 | C | T | -0.042 | 0.007 | 4.54E-09 | -0.004 | 0.011 | 0.734 |
|  | rs7903146 | C | T | -0.306 | 0.008 | 1.00E-200 | -0.013 | 0.012 | 0.277 |
|  | rs7929543 | A | C | -0.083 | 0.014 | 2.20E-09 | -0.014 | 0.019 | 0.443 |
|  | rs7955901 | C | T | 0.044 | 0.007 | 7.16E-10 | -0.003 | 0.011 | 0.785 |
|  | rs8068804 | A | G | 0.059 | 0.008 | 4.41E-14 | 0.021 | 0.012 | 0.080 |
|  | rs8108269 | G | T | 0.064 | 0.008 | 3.11E-16 | 0.004 | 0.012 | 0.724 |
|  | rs825476 | C | T | -0.052 | 0.007 | 6.80E-13 | -0.016 | 0.011 | 0.151 |
|  | rs840967 | A | C | -0.050 | 0.008 | 5.44E-10 | 0.012 | 0.011 | 0.268 |
|  | rs849135 | A | G | -0.100 | 0.007 | 1.04E-43 | -0.003 | 0.011 | 0.742 |
|  | rs853974 | C | T | -0.060 | 0.009 | 7.86E-12 | -0.012 | 0.012 | 0.413 |
|  | rs9369425 | A | G | -0.055 | 0.009 | 1.13E-10 | 0.005 | 0.012 | 0.716 |
|  | rs9894220 | A | G | 0.059 | 0.008 | 1.52E-13 | -0.007 | 0.011 | 0.541 |
|  | rs9928094 | A | G | -0.105 | 0.007 | 3.59E-47 | -0.005 | 0.011 | 0.668 |
|  | rs993380 | A | G | 0.051 | 0.008 | 4.59E-10 | -0.003 | 0.011 | 0.765 |
|  | rs9940149 | A | G | -0.058 | 0.010 | 9.29E-10 | 0.001 | 0.014 | 0.952 |
| *Veillonellaceae* | rs10077431 | A | C | -0.049 | 0.009 | 4.75E-08 | 0.006 | 0.014 | 0.787 |
|  | rs10087241 | A | G | -0.048 | 0.008 | 2.80E-09 | -0.002 | 0.012 | 0.936 |
|  | rs10100265 | A | C | 0.049 | 0.008 | 6.29E-10 | -0.006 | 0.011 | 0.569 |
|  | rs10114341 | C | T | -0.041 | 0.007 | 1.15E-08 | 0.006 | 0.011 | 0.643 |
|  | rs10401969 | C | T | 0.092 | 0.013 | 4.13E-12 | -0.011 | 0.021 | 0.663 |
|  | rs1050226 | G | A | -0.049 | 0.007 | 3.34E-11 | -0.007 | 0.011 | 0.522 |
|  | rs1061813 | A | G | -0.043 | 0.007 | 3.37E-09 | -0.003 | 0.011 | 0.755 |
|  | rs1063355 | T | G | -0.071 | 0.008 | 3.72E-19 | 0.014 | 0.011 | 0.207 |
|  | rs10740322 | A | G | 0.048 | 0.009 | 2.11E-08 | -0.017 | 0.012 | 0.163 |
|  | rs10811661 | C | T | -0.157 | 0.010 | 4.13E-58 | 0.005 | 0.015 | 0.681 |
|  | rs10842994 | C | T | 0.076 | 0.009 | 1.02E-16 | 0.015 | 0.014 | 0.306 |
|  | rs10974438 | A | C | -0.059 | 0.008 | 3.01E-15 | -0.010 | 0.012 | 0.357 |
|  | rs11098676 | C | T | 0.054 | 0.010 | 2.03E-08 | -0.020 | 0.014 | 0.134 |
|  | rs11107116 | G | T | -0.047 | 0.009 | 3.75E-08 | -0.025 | 0.013 | 0.053 |
|  | rs1111875 | T | C | -0.095 | 0.007 | 3.61E-39 | -0.009 | 0.012 | 0.412 |
|  | rs11257655 | C | T | -0.074 | 0.009 | 1.97E-17 | -0.001 | 0.014 | 0.787 |
|  | rs1127655 | C | T | 0.044 | 0.008 | 2.47E-08 | -0.022 | 0.011 | 0.047 |
|  | rs11708067 | A | G | 0.097 | 0.009 | 5.93E-29 | -0.014 | 0.014 | 0.300 |
|  | rs11925227 | A | G | -0.053 | 0.010 | 2.25E-08 | 0.019 | 0.015 | 0.176 |
|  | rs11926707 | C | T | 0.046 | 0.008 | 1.69E-08 | 0.000 | 0.012 | 0.984 |
|  | rs12088739 | A | G | 0.088 | 0.013 | 9.79E-12 | -0.023 | 0.020 | 0.251 |
|  | rs12299509 | A | G | -0.047 | 0.007 | 2.09E-10 | -0.012 | 0.011 | 0.277 |
|  | rs12617659 | C | T | 0.069 | 0.010 | 2.83E-11 | 0.012 | 0.016 | 0.479 |
|  | rs12910825 | A | G | -0.052 | 0.007 | 2.16E-12 | -0.003 | 0.012 | 0.805 |
|  | rs12945601 | C | T | -0.048 | 0.008 | 1.72E-09 | -0.006 | 0.012 | 0.615 |
|  | rs12970134 | A | G | 0.056 | 0.008 | 5.31E-12 | -0.006 | 0.013 | 0.680 |
|  | rs13239186 | C | T | -0.054 | 0.009 | 2.70E-10 | 0.012 | 0.012 | 0.305 |
|  | rs13330951 | A | G | 0.046 | 0.008 | 1.54E-08 | 0.001 | 0.011 | 0.975 |
|  | rs13389219 | C | T | 0.072 | 0.007 | 2.11E-22 | -0.019 | 0.012 | 0.103 |
|  | rs1359790 | G | A | 0.080 | 0.008 | 2.80E-23 | -0.021 | 0.013 | 0.087 |
|  | rs1496653 | G | A | -0.077 | 0.009 | 2.57E-18 | 0.002 | 0.014 | 0.935 |
|  | rs1552224 | C | A | -0.103 | 0.010 | 8.64E-25 | 0.013 | 0.016 | 0.531 |
|  | rs16988333 | A | G | 0.075 | 0.013 | 9.17E-09 | 0.015 | 0.022 | 0.503 |
|  | rs17086692 | G | T | 0.047 | 0.008 | 2.48E-08 | -0.017 | 0.012 | 0.154 |
|  | rs17168486 | C | T | -0.074 | 0.009 | 2.18E-15 | -0.013 | 0.014 | 0.321 |
|  | rs17405722 | A | G | 0.087 | 0.015 | 2.28E-09 | -0.006 | 0.022 | 0.894 |
|  | rs17631783 | C | T | 0.049 | 0.009 | 3.95E-08 | 0.016 | 0.013 | 0.223 |
|  | rs17791513 | A | G | 0.103 | 0.015 | 4.61E-12 | 0.003 | 0.023 | 0.998 |
|  | rs1801214 | C | T | 0.090 | 0.007 | 5.52E-34 | -0.029 | 0.012 | 0.010 |
|  | rs1899951 | T | C | -0.112 | 0.011 | 1.64E-24 | 0.001 | 0.016 | 0.982 |
|  | rs2237892 | C | T | 0.096 | 0.016 | 8.75E-10 | -0.011 | 0.022 | 0.543 |
|  | rs2246618 | C | T | -0.051 | 0.008 | 1.20E-09 | 0.000 | 0.012 | 0.984 |
|  | rs2261181 | C | T | -0.099 | 0.012 | 9.18E-17 | -0.021 | 0.019 | 0.233 |
|  | rs2294120 | G | A | -0.044 | 0.008 | 1.62E-08 | 0.006 | 0.011 | 0.608 |
|  | rs2296173 | A | G | -0.065 | 0.009 | 7.66E-14 | -0.027 | 0.014 | 0.050 |
|  | rs2299383 | C | T | -0.041 | 0.007 | 1.49E-08 | -0.009 | 0.011 | 0.430 |
|  | rs243019 | T | C | -0.057 | 0.007 | 2.29E-15 | -0.002 | 0.011 | 0.875 |
|  | rs2493394 | G | A | 0.073 | 0.011 | 1.15E-10 | 0.028 | 0.018 | 0.116 |
|  | rs2796441 | G | A | 0.072 | 0.007 | 1.96E-22 | -0.002 | 0.011 | 0.813 |
|  | rs2820426 | G | A | 0.052 | 0.007 | 1.30E-12 | -0.007 | 0.012 | 0.555 |
|  | rs2867125 | T | C | -0.060 | 0.010 | 4.33E-10 | -0.002 | 0.015 | 0.986 |
|  | rs2908282 | G | A | -0.055 | 0.009 | 4.25E-09 | -0.023 | 0.015 | 0.110 |
|  | rs2925979 | T | C | 0.053 | 0.008 | 9.06E-12 | -0.013 | 0.012 | 0.324 |
|  | rs2943656 | A | G | -0.090 | 0.007 | 6.70E-34 | 0.001 | 0.012 | 0.909 |
|  | rs3217992 | T | C | 0.053 | 0.007 | 7.23E-13 | 0.005 | 0.012 | 0.705 |
|  | rs340874 | T | C | -0.063 | 0.007 | 8.41E-18 | 0.007 | 0.011 | 0.514 |
|  | rs348330 | G | A | 0.049 | 0.008 | 1.86E-09 | -0.007 | 0.012 | 0.555 |
|  | rs3756784 | G | T | 0.051 | 0.009 | 2.59E-08 | -0.026 | 0.014 | 0.052 |
|  | rs3802177 | G | A | 0.122 | 0.008 | 2.32E-52 | 0.016 | 0.012 | 0.163 |
|  | rs459193 | G | A | 0.071 | 0.008 | 8.81E-18 | 0.020 | 0.013 | 0.118 |
|  | rs4622883 | A | G | 0.044 | 0.008 | 3.02E-08 | -0.021 | 0.011 | 0.071 |
|  | rs4686471 | C | T | 0.053 | 0.008 | 4.28E-11 | 0.009 | 0.012 | 0.431 |
|  | rs4812829 | A | G | 0.053 | 0.010 | 2.44E-08 | 0.008 | 0.014 | 0.390 |
|  | rs4823182 | A | G | -0.048 | 0.008 | 3.36E-10 | -0.003 | 0.012 | 0.813 |
|  | rs4865796 | A | G | 0.053 | 0.008 | 1.33E-11 | -0.020 | 0.012 | 0.080 |
|  | rs516946 | T | C | -0.082 | 0.009 | 3.16E-22 | 0.020 | 0.013 | 0.145 |
|  | rs5215 | C | T | 0.068 | 0.007 | 2.09E-20 | -0.005 | 0.012 | 0.635 |
|  | rs576674 | A | G | -0.065 | 0.010 | 1.79E-11 | -0.020 | 0.015 | 0.154 |
|  | rs6059662 | A | G | -0.045 | 0.008 | 1.51E-08 | -0.002 | 0.012 | 0.888 |
|  | rs61953351 | G | T | 0.070 | 0.009 | 1.98E-14 | -0.019 | 0.013 | 0.179 |
|  | rs622217 | C | T | -0.049 | 0.008 | 3.13E-10 | -0.021 | 0.011 | 0.054 |
|  | rs6515236 | A | C | 0.050 | 0.009 | 3.34E-08 | 0.000 | 0.013 | 0.994 |
|  | rs67232546 | C | T | -0.060 | 0.010 | 4.66E-10 | -0.014 | 0.014 | 0.360 |
|  | rs6767484 | A | G | -0.121 | 0.008 | 2.70E-56 | -0.002 | 0.012 | 0.865 |
|  | rs6785040 | C | T | -0.063 | 0.011 | 1.26E-08 | -0.017 | 0.015 | 0.229 |
|  | rs6795735 | C | T | 0.056 | 0.007 | 1.63E-14 | 0.008 | 0.011 | 0.504 |
|  | rs6878122 | A | G | -0.056 | 0.008 | 1.19E-12 | -0.009 | 0.012 | 0.463 |
|  | rs6960043 | C | T | 0.064 | 0.007 | 3.61E-19 | -0.015 | 0.011 | 0.186 |
|  | rs7144011 | G | T | -0.048 | 0.009 | 1.64E-08 | -0.001 | 0.014 | 0.925 |
|  | rs7177055 | A | G | 0.065 | 0.008 | 2.75E-16 | 0.013 | 0.012 | 0.259 |
|  | rs7240767 | C | T | 0.045 | 0.008 | 2.16E-08 | -0.023 | 0.012 | 0.051 |
|  | rs72892910 | G | T | -0.065 | 0.010 | 6.43E-11 | 0.011 | 0.015 | 0.442 |
|  | rs735949 | C | T | -0.071 | 0.011 | 1.95E-11 | 0.000 | 0.017 | 0.969 |
|  | rs753270 | C | T | 0.053 | 0.008 | 2.70E-11 | -0.013 | 0.011 | 0.267 |
|  | rs7561798 | A | G | -0.040 | 0.007 | 2.79E-08 | 0.027 | 0.011 | 0.016 |
|  | rs7572970 | A | G | -0.059 | 0.009 | 1.39E-11 | 0.028 | 0.013 | 0.031 |
|  | rs7607777 | G | T | 0.137 | 0.013 | 9.40E-28 | 0.013 | 0.019 | 0.606 |
|  | rs7674212 | G | T | 0.047 | 0.008 | 6.18E-10 | -0.021 | 0.011 | 0.060 |
|  | rs7685296 | C | T | 0.051 | 0.008 | 2.32E-10 | 0.016 | 0.012 | 0.190 |
|  | rs7756992 | A | G | -0.130 | 0.008 | 6.00E-62 | 0.010 | 0.012 | 0.399 |
|  | rs7786095 | A | G | 0.074 | 0.013 | 9.64E-09 | 0.008 | 0.019 | 0.678 |
|  | rs780094 | T | C | -0.069 | 0.007 | 5.16E-21 | 0.010 | 0.012 | 0.394 |
|  | rs7845219 | C | T | -0.042 | 0.007 | 4.54E-09 | -0.007 | 0.011 | 0.514 |
|  | rs7903146 | C | T | -0.306 | 0.008 | 1.00E-200 | -0.007 | 0.013 | 0.549 |
|  | rs7929543 | A | C | -0.083 | 0.014 | 2.20E-09 | -0.001 | 0.019 | 0.829 |
|  | rs7955901 | C | T | 0.044 | 0.007 | 7.16E-10 | 0.000 | 0.011 | 0.969 |
|  | rs8068804 | A | G | 0.059 | 0.008 | 4.41E-14 | 0.029 | 0.012 | 0.018 |
|  | rs8108269 | G | T | 0.064 | 0.008 | 3.11E-16 | -0.001 | 0.012 | 0.966 |
|  | rs825476 | C | T | -0.052 | 0.007 | 6.80E-13 | -0.005 | 0.011 | 0.625 |
|  | rs840967 | A | C | -0.050 | 0.008 | 5.44E-10 | -0.001 | 0.012 | 0.966 |
|  | rs849135 | A | G | -0.100 | 0.007 | 1.04E-43 | 0.014 | 0.011 | 0.216 |
|  | rs853974 | C | T | -0.060 | 0.009 | 7.86E-12 | -0.006 | 0.013 | 0.591 |
|  | rs9369425 | A | G | -0.055 | 0.009 | 1.13E-10 | -0.011 | 0.013 | 0.435 |
|  | rs9894220 | A | G | 0.059 | 0.008 | 1.52E-13 | -0.006 | 0.011 | 0.585 |
|  | rs9928094 | A | G | -0.105 | 0.007 | 3.59E-47 | 0.001 | 0.011 | 0.893 |
|  | rs993380 | A | G | 0.051 | 0.008 | 4.59E-10 | -0.010 | 0.012 | 0.365 |
|  | rs9940149 | A | G | -0.058 | 0.010 | 9.29E-10 | -0.036 | 0.014 | 0.013 |
| *Verrucomicrobiaceae* | rs10077431 | A | C | -0.049 | 0.009 | 4.75E-08 | -0.002 | 0.016 | 0.760 |
|  | rs10087241 | A | G | -0.048 | 0.008 | 2.80E-09 | 0.004 | 0.013 | 0.767 |
|  | rs10100265 | A | C | 0.049 | 0.008 | 6.29E-10 | 0.010 | 0.013 | 0.445 |
|  | rs10114341 | C | T | -0.041 | 0.007 | 1.15E-08 | 0.001 | 0.013 | 0.975 |
|  | rs10401969 | C | T | 0.092 | 0.013 | 4.13E-12 | -0.017 | 0.024 | 0.446 |
|  | rs1050226 | G | A | -0.049 | 0.007 | 3.34E-11 | 0.011 | 0.013 | 0.406 |
|  | rs1061813 | A | G | -0.043 | 0.007 | 3.37E-09 | -0.015 | 0.013 | 0.242 |
|  | rs1063355 | T | G | -0.071 | 0.008 | 3.72E-19 | -0.005 | 0.013 | 0.729 |
|  | rs10740322 | A | G | 0.048 | 0.009 | 2.11E-08 | -0.014 | 0.014 | 0.323 |
|  | rs10811661 | C | T | -0.157 | 0.010 | 4.13E-58 | -0.027 | 0.017 | 0.122 |
|  | rs10842994 | C | T | 0.076 | 0.009 | 1.02E-16 | 0.010 | 0.016 | 0.568 |
|  | rs10974438 | A | C | -0.059 | 0.008 | 3.01E-15 | 0.006 | 0.014 | 0.683 |
|  | rs11098676 | C | T | 0.054 | 0.010 | 2.03E-08 | -0.043 | 0.016 | 0.009 |
|  | rs11107116 | G | T | -0.047 | 0.009 | 3.75E-08 | -0.005 | 0.016 | 0.732 |
|  | rs1111875 | T | C | -0.095 | 0.007 | 3.61E-39 | 0.008 | 0.013 | 0.521 |
|  | rs11257655 | C | T | -0.074 | 0.009 | 1.97E-17 | 0.024 | 0.016 | 0.133 |
|  | rs1127655 | C | T | 0.044 | 0.008 | 2.47E-08 | -0.009 | 0.013 | 0.504 |
|  | rs11708067 | A | G | 0.097 | 0.009 | 5.93E-29 | -0.023 | 0.016 | 0.158 |
|  | rs11925227 | A | G | -0.053 | 0.010 | 2.25E-08 | 0.005 | 0.017 | 0.750 |
|  | rs11926707 | C | T | 0.046 | 0.008 | 1.69E-08 | 0.004 | 0.014 | 0.796 |
|  | rs12088739 | A | G | 0.088 | 0.013 | 9.79E-12 | 0.022 | 0.023 | 0.356 |
|  | rs12299509 | A | G | -0.047 | 0.007 | 2.09E-10 | 0.005 | 0.013 | 0.736 |
|  | rs12617659 | C | T | 0.069 | 0.010 | 2.83E-11 | 0.017 | 0.018 | 0.359 |
|  | rs12910825 | A | G | -0.052 | 0.007 | 2.16E-12 | -0.009 | 0.013 | 0.507 |
|  | rs12945601 | C | T | -0.048 | 0.008 | 1.72E-09 | 0.004 | 0.013 | 0.777 |
|  | rs12970134 | A | G | 0.056 | 0.008 | 5.31E-12 | 0.030 | 0.015 | 0.038 |
|  | rs13239186 | C | T | -0.054 | 0.009 | 2.70E-10 | -0.021 | 0.014 | 0.117 |
|  | rs13330951 | A | G | 0.046 | 0.008 | 1.54E-08 | -0.025 | 0.013 | 0.057 |
|  | rs13389219 | C | T | 0.072 | 0.007 | 2.11E-22 | 0.005 | 0.013 | 0.762 |
|  | rs1359790 | G | A | 0.080 | 0.008 | 2.80E-23 | 0.007 | 0.015 | 0.606 |
|  | rs1496653 | G | A | -0.077 | 0.009 | 2.57E-18 | 0.012 | 0.016 | 0.492 |
|  | rs1552224 | C | A | -0.103 | 0.010 | 8.64E-25 | -0.015 | 0.018 | 0.313 |
|  | rs16988333 | A | G | 0.075 | 0.013 | 9.17E-09 | 0.021 | 0.025 | 0.373 |
|  | rs17086692 | G | T | 0.047 | 0.008 | 2.48E-08 | -0.010 | 0.014 | 0.451 |
|  | rs17168486 | C | T | -0.074 | 0.009 | 2.18E-15 | 0.010 | 0.017 | 0.572 |
|  | rs17405722 | A | G | 0.087 | 0.015 | 2.28E-09 | -0.035 | 0.025 | 0.153 |
|  | rs17631783 | C | T | 0.049 | 0.009 | 3.95E-08 | -0.020 | 0.015 | 0.171 |
|  | rs17791513 | A | G | 0.103 | 0.015 | 4.61E-12 | 0.036 | 0.026 | 0.164 |
|  | rs1801214 | C | T | 0.090 | 0.007 | 5.52E-34 | -0.002 | 0.013 | 0.924 |
|  | rs1899951 | T | C | -0.112 | 0.011 | 1.64E-24 | 0.043 | 0.019 | 0.023 |
|  | rs2237892 | C | T | 0.096 | 0.016 | 8.75E-10 | -0.002 | 0.027 | 0.904 |
|  | rs2246618 | C | T | -0.051 | 0.008 | 1.20E-09 | -0.010 | 0.014 | 0.476 |
|  | rs2261181 | C | T | -0.099 | 0.012 | 9.18E-17 | 0.055 | 0.022 | 0.013 |
|  | rs2294120 | G | A | -0.044 | 0.008 | 1.62E-08 | -0.008 | 0.013 | 0.546 |
|  | rs2296173 | A | G | -0.065 | 0.009 | 7.66E-14 | 0.007 | 0.016 | 0.627 |
|  | rs2299383 | C | T | -0.041 | 0.007 | 1.49E-08 | -0.023 | 0.013 | 0.085 |
|  | rs243019 | T | C | -0.057 | 0.007 | 2.29E-15 | 0.010 | 0.013 | 0.426 |
|  | rs2493394 | G | A | 0.073 | 0.011 | 1.15E-10 | 0.020 | 0.021 | 0.347 |
|  | rs2796441 | G | A | 0.072 | 0.007 | 1.96E-22 | -0.009 | 0.013 | 0.471 |
|  | rs2820426 | G | A | 0.052 | 0.007 | 1.30E-12 | 0.005 | 0.013 | 0.696 |
|  | rs2867125 | T | C | -0.060 | 0.010 | 4.33E-10 | 0.045 | 0.017 | 0.006 |
|  | rs2908282 | G | A | -0.055 | 0.009 | 4.25E-09 | -0.008 | 0.017 | 0.589 |
|  | rs2925979 | T | C | 0.053 | 0.008 | 9.06E-12 | -0.003 | 0.014 | 0.843 |
|  | rs2943656 | A | G | -0.090 | 0.007 | 6.70E-34 | 0.029 | 0.014 | 0.035 |
|  | rs3217992 | T | C | 0.053 | 0.007 | 7.23E-13 | 0.002 | 0.013 | 0.935 |
|  | rs340874 | T | C | -0.063 | 0.007 | 8.41E-18 | 0.000 | 0.013 | 0.955 |
|  | rs348330 | G | A | 0.049 | 0.008 | 1.86E-09 | 0.002 | 0.014 | 0.879 |
|  | rs3756784 | G | T | 0.051 | 0.009 | 2.59E-08 | -0.026 | 0.016 | 0.124 |
|  | rs3802177 | G | A | 0.122 | 0.008 | 2.32E-52 | 0.033 | 0.014 | 0.016 |
|  | rs459193 | G | A | 0.071 | 0.008 | 8.81E-18 | -0.010 | 0.015 | 0.534 |
|  | rs4622883 | A | G | 0.044 | 0.008 | 3.02E-08 | 0.012 | 0.013 | 0.355 |
|  | rs4686471 | C | T | 0.053 | 0.008 | 4.28E-11 | -0.009 | 0.014 | 0.445 |
|  | rs4812829 | A | G | 0.053 | 0.010 | 2.44E-08 | -0.018 | 0.017 | 0.218 |
|  | rs4823182 | A | G | -0.048 | 0.008 | 3.36E-10 | -0.010 | 0.014 | 0.473 |
|  | rs4865796 | A | G | 0.053 | 0.008 | 1.33E-11 | -0.006 | 0.014 | 0.634 |
|  | rs516946 | T | C | -0.082 | 0.009 | 3.16E-22 | 0.013 | 0.015 | 0.428 |
|  | rs5215 | C | T | 0.068 | 0.007 | 2.09E-20 | -0.007 | 0.014 | 0.604 |
|  | rs576674 | A | G | -0.065 | 0.010 | 1.79E-11 | 0.030 | 0.017 | 0.072 |
|  | rs6059662 | A | G | -0.045 | 0.008 | 1.51E-08 | -0.004 | 0.014 | 0.767 |
|  | rs61953351 | G | T | 0.070 | 0.009 | 1.98E-14 | 0.007 | 0.015 | 0.568 |
|  | rs622217 | C | T | -0.049 | 0.008 | 3.13E-10 | 0.002 | 0.013 | 0.890 |
|  | rs6515236 | A | C | 0.050 | 0.009 | 3.34E-08 | -0.010 | 0.015 | 0.460 |
|  | rs67232546 | C | T | -0.060 | 0.010 | 4.66E-10 | -0.002 | 0.016 | 0.942 |
|  | rs6767484 | A | G | -0.121 | 0.008 | 2.70E-56 | -0.019 | 0.014 | 0.180 |
|  | rs6785040 | C | T | -0.063 | 0.011 | 1.26E-08 | 0.026 | 0.018 | 0.158 |
|  | rs6795735 | C | T | 0.056 | 0.007 | 1.63E-14 | -0.014 | 0.013 | 0.281 |
|  | rs6878122 | A | G | -0.056 | 0.008 | 1.19E-12 | 0.013 | 0.014 | 0.393 |
|  | rs6960043 | C | T | 0.064 | 0.007 | 3.61E-19 | -0.001 | 0.013 | 0.968 |
|  | rs7144011 | G | T | -0.048 | 0.009 | 1.64E-08 | -0.010 | 0.016 | 0.518 |
|  | rs7177055 | A | G | 0.065 | 0.008 | 2.75E-16 | 0.002 | 0.014 | 0.871 |
|  | rs7240767 | C | T | 0.045 | 0.008 | 2.16E-08 | 0.006 | 0.013 | 0.671 |
|  | rs72892910 | G | T | -0.065 | 0.010 | 6.43E-11 | -0.019 | 0.017 | 0.338 |
|  | rs735949 | C | T | -0.071 | 0.011 | 1.95E-11 | 0.005 | 0.019 | 0.807 |
|  | rs753270 | C | T | 0.053 | 0.008 | 2.70E-11 | 0.014 | 0.013 | 0.300 |
|  | rs7561798 | A | G | -0.040 | 0.007 | 2.79E-08 | 0.014 | 0.013 | 0.281 |
|  | rs7572970 | A | G | -0.059 | 0.009 | 1.39E-11 | -0.013 | 0.015 | 0.385 |
|  | rs7607777 | G | T | 0.137 | 0.013 | 9.40E-28 | 0.022 | 0.021 | 0.353 |
|  | rs7674212 | G | T | 0.047 | 0.008 | 6.18E-10 | -0.001 | 0.013 | 0.929 |
|  | rs7685296 | C | T | 0.051 | 0.008 | 2.32E-10 | -0.009 | 0.014 | 0.556 |
|  | rs7756992 | A | G | -0.130 | 0.008 | 6.00E-62 | -0.002 | 0.014 | 0.884 |
|  | rs7786095 | A | G | 0.074 | 0.013 | 9.64E-09 | 0.017 | 0.022 | 0.455 |
|  | rs780094 | T | C | -0.069 | 0.007 | 5.16E-21 | -0.001 | 0.013 | 0.941 |
|  | rs7845219 | C | T | -0.042 | 0.007 | 4.54E-09 | -0.004 | 0.013 | 0.780 |
|  | rs7903146 | C | T | -0.306 | 0.008 | 1.00E-200 | 0.008 | 0.015 | 0.574 |
|  | rs7929543 | A | C | -0.083 | 0.014 | 2.20E-09 | 0.000 | 0.023 | 0.972 |
|  | rs7955901 | C | T | 0.044 | 0.007 | 7.16E-10 | 0.003 | 0.013 | 0.820 |
|  | rs8068804 | A | G | 0.059 | 0.008 | 4.41E-14 | -0.015 | 0.014 | 0.305 |
|  | rs8108269 | G | T | 0.064 | 0.008 | 3.11E-16 | -0.033 | 0.014 | 0.021 |
|  | rs825476 | C | T | -0.052 | 0.007 | 6.80E-13 | -0.019 | 0.013 | 0.157 |
|  | rs840967 | A | C | -0.050 | 0.008 | 5.44E-10 | 0.003 | 0.013 | 0.800 |
|  | rs849135 | A | G | -0.100 | 0.007 | 1.04E-43 | -0.008 | 0.013 | 0.566 |
|  | rs853974 | C | T | -0.060 | 0.009 | 7.86E-12 | 0.018 | 0.015 | 0.231 |
|  | rs9369425 | A | G | -0.055 | 0.009 | 1.13E-10 | 0.008 | 0.015 | 0.493 |
|  | rs9894220 | A | G | 0.059 | 0.008 | 1.52E-13 | -0.011 | 0.013 | 0.405 |
|  | rs9928094 | A | G | -0.105 | 0.007 | 3.59E-47 | -0.008 | 0.013 | 0.576 |
|  | rs993380 | A | G | 0.051 | 0.008 | 4.59E-10 | 0.017 | 0.014 | 0.207 |
|  | rs9940149 | A | G | -0.058 | 0.010 | 9.29E-10 | 0.011 | 0.017 | 0.583 |

Abbreviations: SNP, single nucleotide polymorphism; IVs, instrumental variables; T2DM, type 2 diabetes mellitus; GWAS, genome-wide association study.
