# Supplementary material for: Genetically Predicted Causality of 28 Gut Microbiome Families and Type 2 Diabetes Mellitus Risk
Source: Front Endocrinol (Lausanne). 2022 Feb 3;13:780133. doi: 10.3389/fendo.2022.780133 (PMC8851667; doi:10.3389/fendo.2022.780133)
Supplement: Supplementary file 8 [file Table_6.docx]

| **Supplementary Table 6. SNPs used as IVs from T2DM and gut microbiome GWAS (Asian)** | | | | | | | | | |
| --- | --- | --- | --- | --- | --- | --- | --- | --- | --- |
| **Bacterial traits** | **SNP** | **Effect allele** | **Other allele** | **T2DM** | | | **Gut microbiome** | | |
|  |  |  |  | **Beta** | **SE** | ***P* value** | **Beta** | **SE** | ***P* value** |
| *Acidaminococcaceae* | rs10011838 | G | A | 0.073 | 0.007 | 1.43E-27 | 0.000 | 0.014 | 0.991 |
|  | rs1016565 | G | A | -0.038 | 0.007 | 2.18E-08 | -0.024 | 0.013 | 0.082 |
|  | rs10507349 | G | A | 0.064 | 0.007 | 1.69E-21 | 0.009 | 0.015 | 0.530 |
|  | rs10852123 | A | C | 0.060 | 0.008 | 8.38E-13 | 0.016 | 0.015 | 0.282 |
|  | rs10860209 | C | A | 0.040 | 0.007 | 5.67E-09 | 0.000 | 0.017 | 0.997 |
|  | rs10938398 | G | A | -0.046 | 0.007 | 3.84E-10 | -0.018 | 0.013 | 0.175 |
|  | rs10950550 | T | G | 0.065 | 0.007 | 1.75E-19 | -0.013 | 0.013 | 0.284 |
|  | rs10965248 | T | C | 0.183 | 0.007 | 4.42E-164 | -0.004 | 0.016 | 0.802 |
|  | rs111246699 | G | A | -0.061 | 0.008 | 1.54E-15 | 0.022 | 0.015 | 0.150 |
|  | rs113154802 | C | T | 0.060 | 0.011 | 3.51E-08 | 0.022 | 0.022 | 0.331 |
|  | rs1182444 | A | G | -0.047 | 0.007 | 1.67E-12 | 0.016 | 0.013 | 0.223 |
|  | rs11926494 | G | A | 0.112 | 0.009 | 2.69E-37 | -0.019 | 0.022 | 0.328 |
|  | rs1206684 | G | A | 0.040 | 0.007 | 4.42E-09 | 0.022 | 0.013 | 0.111 |
|  | rs123378 | G | A | 0.053 | 0.008 | 2.22E-10 | 0.002 | 0.014 | 0.793 |
|  | rs1260326 | T | C | -0.063 | 0.007 | 1.01E-21 | -0.018 | 0.013 | 0.167 |
|  | rs12625671 | T | C | -0.066 | 0.007 | 2.25E-21 | 0.025 | 0.018 | 0.129 |
|  | rs1266488 | T | C | 0.044 | 0.007 | 6.29E-10 | 0.014 | 0.039 | 0.832 |
|  | rs12698877 | A | G | -0.067 | 0.007 | 6.96E-22 | 0.012 | 0.014 | 0.423 |
|  | rs12818766 | G | A | -0.054 | 0.009 | 2.46E-10 | 0.007 | 0.017 | 0.607 |
|  | rs13092876 | G | A | -0.126 | 0.007 | 1.91E-66 | -0.003 | 0.014 | 0.854 |
|  | rs13266634 | C | T | 0.116 | 0.007 | 3.72E-67 | -0.019 | 0.014 | 0.145 |
|  | rs1328412 | T | C | 0.097 | 0.015 | 6.41E-11 | -0.003 | 0.025 | 0.808 |
|  | rs1421085 | T | C | -0.130 | 0.009 | 1.55E-48 | 0.012 | 0.013 | 0.315 |
|  | rs1426371 | G | A | 0.048 | 0.007 | 7.76E-12 | -0.004 | 0.015 | 0.742 |
|  | rs1459513 | A | C | -0.046 | 0.007 | 3.73E-11 | 0.026 | 0.028 | 0.409 |
|  | rs147707338 | C | T | -0.087 | 0.014 | 6.47E-10 | 0.011 | 0.022 | 0.630 |
|  | rs1513275 | T | C | -0.057 | 0.009 | 2.76E-11 | -0.028 | 0.015 | 0.067 |
|  | rs16884229 | T | C | -0.097 | 0.007 | 1.15E-43 | 0.013 | 0.016 | 0.414 |
|  | rs17168486 | C | T | -0.064 | 0.007 | 8.23E-22 | -0.005 | 0.016 | 0.904 |
|  | rs1850421 | A | C | 0.044 | 0.007 | 1.41E-09 | 0.000 | 0.014 | 0.983 |
|  | rs2074120 | A | C | 0.041 | 0.007 | 8.38E-09 | -0.009 | 0.013 | 0.450 |
|  | rs2092518 | G | T | 0.046 | 0.007 | 1.39E-10 | -0.022 | 0.013 | 0.079 |
|  | rs2126736 | A | G | 0.038 | 0.007 | 1.84E-08 | -0.003 | 0.013 | 0.819 |
|  | rs2240885 | G | A | -0.042 | 0.007 | 2.79E-09 | 0.000 | 0.015 | 0.992 |
|  | rs2269245 | G | A | 0.054 | 0.009 | 5.40E-10 | -0.011 | 0.016 | 0.430 |
|  | rs2327777 | T | C | 0.050 | 0.007 | 1.06E-13 | 0.005 | 0.013 | 0.717 |
|  | rs2583934 | G | T | -0.058 | 0.007 | 4.95E-16 | 0.005 | 0.017 | 0.758 |
|  | rs261982 | C | T | -0.040 | 0.007 | 3.12E-09 | -0.021 | 0.013 | 0.095 |
|  | rs2706710 | C | T | -0.071 | 0.013 | 1.67E-08 | 0.010 | 0.017 | 0.655 |
|  | rs2796441 | G | A | 0.075 | 0.007 | 1.43E-28 | -0.010 | 0.013 | 0.434 |
|  | rs28599782 | G | A | -0.067 | 0.008 | 4.64E-16 | 0.026 | 0.035 | 0.247 |
|  | rs28691713 | C | T | 0.066 | 0.008 | 1.79E-17 | -0.015 | 0.013 | 0.251 |
|  | rs2908279 | T | G | -0.046 | 0.007 | 8.42E-11 | -0.017 | 0.013 | 0.172 |
|  | rs2980883 | T | G | 0.042 | 0.007 | 3.93E-09 | -0.003 | 0.018 | 0.890 |
|  | rs3094508 | T | C | -0.059 | 0.008 | 1.31E-13 | -0.001 | 0.013 | 0.947 |
|  | rs329122 | G | A | -0.039 | 0.007 | 2.22E-08 | -0.011 | 0.013 | 0.393 |
|  | rs34811727 | G | T | -0.076 | 0.013 | 3.32E-09 | -0.005 | 0.026 | 0.834 |
|  | rs349359 | A | C | -0.043 | 0.008 | 3.05E-08 | -0.011 | 0.019 | 0.617 |
|  | rs35589574 | C | T | -0.171 | 0.011 | 1.58E-55 | -0.023 | 0.015 | 0.184 |
|  | rs3735641 | A | G | -0.069 | 0.008 | 3.62E-19 | 0.010 | 0.013 | 0.450 |
|  | rs3751236 | G | A | 0.067 | 0.007 | 6.58E-21 | 0.021 | 0.016 | 0.147 |
|  | rs3852529 | C | T | -0.099 | 0.010 | 2.07E-24 | 0.015 | 0.022 | 0.480 |
|  | rs3887925 | C | T | -0.040 | 0.007 | 3.12E-09 | 0.013 | 0.013 | 0.284 |
|  | rs391933 | G | A | 0.037 | 0.007 | 1.46E-08 | -0.024 | 0.013 | 0.067 |
|  | rs4273712 | A | G | -0.047 | 0.007 | 2.56E-12 | 0.016 | 0.014 | 0.253 |
|  | rs4499362 | C | T | 0.044 | 0.007 | 1.53E-09 | 0.029 | 0.016 | 0.073 |
|  | rs476828 | T | C | -0.084 | 0.008 | 4.81E-27 | -0.013 | 0.015 | 0.428 |
|  | rs4930974 | C | T | -0.069 | 0.008 | 4.07E-19 | -0.025 | 0.015 | 0.124 |
|  | rs532504 | G | A | -0.055 | 0.008 | 7.39E-12 | 0.002 | 0.016 | 0.885 |
|  | rs55700915 | G | A | -0.040 | 0.007 | 1.50E-08 | -0.009 | 0.016 | 0.530 |
|  | rs58524310 | A | G | -0.047 | 0.007 | 8.41E-11 | 0.004 | 0.018 | 0.818 |
|  | rs58718028 | C | T | -0.073 | 0.007 | 3.35E-25 | -0.013 | 0.015 | 0.358 |
|  | rs6012878 | G | A | 0.041 | 0.007 | 4.32E-09 | 0.011 | 0.013 | 0.420 |
|  | rs60573766 | C | T | 0.044 | 0.007 | 4.30E-10 | 0.007 | 0.018 | 0.767 |
|  | rs61779313 | T | C | -0.060 | 0.009 | 5.59E-11 | 0.010 | 0.015 | 0.459 |
|  | rs61975988 | A | G | 0.040 | 0.007 | 1.97E-09 | 0.007 | 0.014 | 0.685 |
|  | rs62173901 | A | G | 0.123 | 0.020 | 6.58E-10 | -0.013 | 0.019 | 0.424 |
|  | rs62405419 | G | T | -0.044 | 0.008 | 3.79E-09 | 0.028 | 0.019 | 0.109 |
|  | rs633862 | C | T | -0.039 | 0.007 | 1.26E-08 | 0.002 | 0.013 | 0.881 |
|  | rs6416749 | C | T | 0.052 | 0.008 | 3.40E-12 | 0.027 | 0.014 | 0.054 |
|  | rs6731688 | A | C | -0.098 | 0.012 | 2.55E-17 | 0.001 | 0.017 | 0.985 |
|  | rs703980 | G | A | 0.060 | 0.007 | 6.55E-19 | -0.010 | 0.013 | 0.405 |
|  | rs7107784 | G | A | 0.086 | 0.012 | 2.60E-13 | -0.016 | 0.014 | 0.239 |
|  | rs7109575 | G | A | 0.141 | 0.015 | 5.46E-21 | 0.017 | 0.018 | 0.301 |
|  | rs7250869 | T | C | 0.056 | 0.007 | 2.29E-16 | 0.006 | 0.014 | 0.656 |
|  | rs73085586 | G | A | 0.043 | 0.007 | 1.66E-09 | -0.024 | 0.016 | 0.130 |
|  | rs7313668 | G | T | -0.045 | 0.007 | 4.91E-11 | 0.009 | 0.013 | 0.493 |
|  | rs73347525 | A | G | 0.059 | 0.009 | 7.46E-11 | 0.026 | 0.017 | 0.110 |
|  | rs742762 | A | C | 0.075 | 0.008 | 1.79E-22 | -0.014 | 0.020 | 0.437 |
|  | rs7501939 | T | C | 0.120 | 0.007 | 1.60E-60 | 0.023 | 0.013 | 0.076 |
|  | rs75990271 | T | C | 0.066 | 0.010 | 3.22E-11 | 0.004 | 0.029 | 0.851 |
|  | rs7656416 | C | T | 0.100 | 0.007 | 9.01E-42 | -0.015 | 0.036 | 0.602 |
|  | rs76704029 | T | C | 0.055 | 0.010 | 3.39E-08 | -0.008 | 0.028 | 0.949 |
|  | rs7787720 | C | T | -0.054 | 0.007 | 2.25E-15 | 0.024 | 0.013 | 0.063 |
|  | rs7901695 | T | C | -0.275 | 0.017 | 8.18E-62 | 0.008 | 0.014 | 0.545 |
|  | rs80196932 | T | C | 0.060 | 0.008 | 7.57E-13 | -0.005 | 0.018 | 0.767 |
|  | rs8026714 | G | A | -0.066 | 0.007 | 1.06E-22 | -0.015 | 0.018 | 0.449 |
|  | rs8043085 | G | T | -0.052 | 0.007 | 2.06E-14 | 0.010 | 0.015 | 0.482 |
|  | rs896852 | G | T | 0.042 | 0.007 | 6.42E-09 | -0.006 | 0.013 | 0.659 |
|  | rs9316706 | A | G | 0.041 | 0.007 | 3.33E-09 | -0.042 | 0.017 | 0.012 |
|  | rs9350271 | G | A | -0.193 | 0.007 | 4.95E-183 | 0.011 | 0.013 | 0.408 |
|  | rs9379084 | G | A | 0.072 | 0.009 | 2.20E-14 | 0.009 | 0.021 | 0.607 |
|  | rs9390022 | T | C | 0.048 | 0.008 | 6.35E-09 | -0.009 | 0.013 | 0.528 |
|  | rs9461650 | G | A | 0.060 | 0.009 | 1.99E-12 | -0.013 | 0.017 | 0.489 |
|  | rs9523295 | G | A | 0.078 | 0.009 | 7.24E-18 | -0.017 | 0.015 | 0.208 |
|  | rs9788635 | C | T | 0.058 | 0.008 | 9.43E-14 | 0.004 | 0.019 | 0.890 |
|  | rs9948462 | C | T | -0.047 | 0.008 | 8.70E-10 | 0.011 | 0.013 | 0.440 |
| *Actinomycetaceae* | rs10011838 | G | A | 0.073 | 0.007 | 1.43E-27 | -0.006 | 0.018 | 0.729 |
|  | rs1016565 | G | A | -0.038 | 0.007 | 2.18E-08 | -0.009 | 0.017 | 0.584 |
|  | rs10507349 | G | A | 0.064 | 0.007 | 1.69E-21 | -0.026 | 0.019 | 0.140 |
|  | rs10852123 | A | C | 0.060 | 0.008 | 8.38E-13 | -0.024 | 0.018 | 0.182 |
|  | rs10860209 | C | A | 0.040 | 0.007 | 5.67E-09 | -0.034 | 0.022 | 0.151 |
|  | rs10938398 | G | A | -0.046 | 0.007 | 3.84E-10 | -0.038 | 0.016 | 0.019 |
|  | rs10950550 | T | G | 0.065 | 0.007 | 1.75E-19 | 0.002 | 0.016 | 0.907 |
|  | rs10965248 | T | C | 0.183 | 0.007 | 4.42E-164 | -0.024 | 0.021 | 0.226 |
|  | rs111246699 | G | A | -0.061 | 0.008 | 1.54E-15 | -0.001 | 0.019 | 0.913 |
|  | rs113154802 | C | T | 0.060 | 0.011 | 3.51E-08 | 0.000 | 0.027 | 0.995 |
|  | rs1182444 | A | G | -0.047 | 0.007 | 1.67E-12 | 0.008 | 0.017 | 0.623 |
|  | rs11926494 | G | A | 0.112 | 0.009 | 2.69E-37 | 0.013 | 0.028 | 0.630 |
|  | rs1206684 | G | A | 0.040 | 0.007 | 4.42E-09 | -0.006 | 0.017 | 0.715 |
|  | rs123378 | G | A | 0.053 | 0.008 | 2.22E-10 | -0.007 | 0.017 | 0.675 |
|  | rs1260326 | T | C | -0.063 | 0.007 | 1.01E-21 | -0.017 | 0.017 | 0.299 |
|  | rs12625671 | T | C | -0.066 | 0.007 | 2.25E-21 | 0.001 | 0.025 | 0.949 |
|  | rs12698877 | A | G | -0.067 | 0.007 | 6.96E-22 | 0.000 | 0.018 | 0.971 |
|  | rs12818766 | G | A | -0.054 | 0.009 | 2.46E-10 | 0.017 | 0.022 | 0.431 |
|  | rs13092876 | G | A | -0.126 | 0.007 | 1.91E-66 | -0.022 | 0.017 | 0.196 |
|  | rs13266634 | C | T | 0.116 | 0.007 | 3.72E-67 | 0.016 | 0.017 | 0.391 |
|  | rs1328412 | T | C | 0.097 | 0.015 | 6.41E-11 | 0.010 | 0.032 | 0.737 |
|  | rs1421085 | T | C | -0.130 | 0.009 | 1.55E-48 | -0.007 | 0.016 | 0.706 |
|  | rs1426371 | G | A | 0.048 | 0.007 | 7.76E-12 | 0.024 | 0.018 | 0.206 |
|  | rs1459513 | A | C | -0.046 | 0.007 | 3.73E-11 | 0.009 | 0.037 | 0.895 |
|  | rs147707338 | C | T | -0.087 | 0.014 | 6.47E-10 | -0.034 | 0.026 | 0.234 |
|  | rs1513275 | T | C | -0.057 | 0.009 | 2.76E-11 | 0.009 | 0.020 | 0.676 |
|  | rs16884229 | T | C | -0.097 | 0.007 | 1.15E-43 | 0.016 | 0.020 | 0.460 |
|  | rs17168486 | C | T | -0.064 | 0.007 | 8.23E-22 | -0.020 | 0.021 | 0.377 |
|  | rs1850421 | A | C | 0.044 | 0.007 | 1.41E-09 | -0.011 | 0.018 | 0.539 |
|  | rs2074120 | A | C | 0.041 | 0.007 | 8.38E-09 | 0.004 | 0.016 | 0.789 |
|  | rs2092518 | G | T | 0.046 | 0.007 | 1.39E-10 | -0.033 | 0.016 | 0.045 |
|  | rs2126736 | A | G | 0.038 | 0.007 | 1.84E-08 | 0.010 | 0.016 | 0.534 |
|  | rs2240885 | G | A | -0.042 | 0.007 | 2.79E-09 | -0.003 | 0.020 | 0.857 |
|  | rs2269245 | G | A | 0.054 | 0.009 | 5.40E-10 | 0.023 | 0.020 | 0.278 |
|  | rs2327777 | T | C | 0.050 | 0.007 | 1.06E-13 | 0.010 | 0.016 | 0.549 |
|  | rs2583934 | G | T | -0.058 | 0.007 | 4.95E-16 | 0.011 | 0.023 | 0.625 |
|  | rs261982 | C | T | -0.040 | 0.007 | 3.12E-09 | 0.009 | 0.016 | 0.586 |
|  | rs2706710 | C | T | -0.071 | 0.013 | 1.67E-08 | 0.008 | 0.021 | 0.792 |
|  | rs2796441 | G | A | 0.075 | 0.007 | 1.43E-28 | -0.015 | 0.016 | 0.364 |
|  | rs28691713 | C | T | 0.066 | 0.008 | 1.79E-17 | 0.008 | 0.016 | 0.625 |
|  | rs2908279 | T | G | -0.046 | 0.007 | 8.42E-11 | -0.001 | 0.016 | 0.938 |
|  | rs2980883 | T | G | 0.042 | 0.007 | 3.93E-09 | -0.015 | 0.023 | 0.597 |
|  | rs3094508 | T | C | -0.059 | 0.008 | 1.31E-13 | -0.010 | 0.016 | 0.515 |
|  | rs329122 | G | A | -0.039 | 0.007 | 2.22E-08 | -0.024 | 0.016 | 0.146 |
|  | rs34811727 | G | T | -0.076 | 0.013 | 3.32E-09 | 0.010 | 0.032 | 0.764 |
|  | rs349359 | A | C | -0.043 | 0.008 | 3.05E-08 | 0.034 | 0.025 | 0.170 |
|  | rs35589574 | C | T | -0.171 | 0.011 | 1.58E-55 | -0.025 | 0.019 | 0.189 |
|  | rs3735641 | A | G | -0.069 | 0.008 | 3.62E-19 | 0.000 | 0.017 | 0.974 |
|  | rs3751236 | G | A | 0.067 | 0.007 | 6.58E-21 | -0.025 | 0.020 | 0.260 |
|  | rs3852529 | C | T | -0.099 | 0.010 | 2.07E-24 | 0.004 | 0.028 | 0.889 |
|  | rs3887925 | C | T | -0.040 | 0.007 | 3.12E-09 | 0.000 | 0.016 | 0.977 |
|  | rs391933 | G | A | 0.037 | 0.007 | 1.46E-08 | 0.004 | 0.016 | 0.799 |
|  | rs4273712 | A | G | -0.047 | 0.007 | 2.56E-12 | 0.007 | 0.018 | 0.651 |
|  | rs4499362 | C | T | 0.044 | 0.007 | 1.53E-09 | 0.038 | 0.020 | 0.064 |
|  | rs476828 | T | C | -0.084 | 0.008 | 4.81E-27 | -0.009 | 0.019 | 0.665 |
|  | rs4930974 | C | T | -0.069 | 0.008 | 4.07E-19 | 0.012 | 0.019 | 0.556 |
|  | rs532504 | G | A | -0.055 | 0.008 | 7.39E-12 | 0.026 | 0.020 | 0.202 |
|  | rs55700915 | G | A | -0.040 | 0.007 | 1.50E-08 | -0.010 | 0.020 | 0.633 |
|  | rs58524310 | A | G | -0.047 | 0.007 | 8.41E-11 | -0.011 | 0.022 | 0.652 |
|  | rs58718028 | C | T | -0.073 | 0.007 | 3.35E-25 | -0.018 | 0.019 | 0.345 |
|  | rs6012878 | G | A | 0.041 | 0.007 | 4.32E-09 | 0.035 | 0.016 | 0.029 |
|  | rs60573766 | C | T | 0.044 | 0.007 | 4.30E-10 | -0.004 | 0.023 | 0.840 |
|  | rs61779313 | T | C | -0.060 | 0.009 | 5.59E-11 | -0.031 | 0.019 | 0.095 |
|  | rs61975988 | A | G | 0.040 | 0.007 | 1.97E-09 | -0.021 | 0.019 | 0.262 |
|  | rs62173901 | A | G | 0.123 | 0.020 | 6.58E-10 | 0.027 | 0.023 | 0.191 |
|  | rs62405419 | G | T | -0.044 | 0.008 | 3.79E-09 | -0.027 | 0.026 | 0.317 |
|  | rs633862 | C | T | -0.039 | 0.007 | 1.26E-08 | 0.010 | 0.016 | 0.555 |
|  | rs6416749 | C | T | 0.052 | 0.008 | 3.40E-12 | -0.021 | 0.017 | 0.222 |
|  | rs6731688 | A | C | -0.098 | 0.012 | 2.55E-17 | 0.000 | 0.022 | 0.971 |
|  | rs703980 | G | A | 0.060 | 0.007 | 6.55E-19 | -0.025 | 0.016 | 0.128 |
|  | rs7107784 | G | A | 0.086 | 0.012 | 2.60E-13 | -0.013 | 0.018 | 0.440 |
|  | rs7109575 | G | A | 0.141 | 0.015 | 5.46E-21 | 0.011 | 0.022 | 0.675 |
|  | rs7250869 | T | C | 0.056 | 0.007 | 2.29E-16 | 0.000 | 0.017 | 0.994 |
|  | rs73085586 | G | A | 0.043 | 0.007 | 1.66E-09 | -0.005 | 0.020 | 0.800 |
|  | rs7313668 | G | T | -0.045 | 0.007 | 4.91E-11 | 0.001 | 0.016 | 0.953 |
|  | rs73347525 | A | G | 0.059 | 0.009 | 7.46E-11 | 0.003 | 0.021 | 0.884 |
|  | rs742762 | A | C | 0.075 | 0.008 | 1.79E-22 | 0.009 | 0.026 | 0.777 |
|  | rs7501939 | T | C | 0.120 | 0.007 | 1.60E-60 | -0.027 | 0.016 | 0.111 |
|  | rs75990271 | T | C | 0.066 | 0.010 | 3.22E-11 | -0.034 | 0.036 | 0.393 |
|  | rs76704029 | T | C | 0.055 | 0.010 | 3.39E-08 | -0.023 | 0.041 | 0.576 |
|  | rs7787720 | C | T | -0.054 | 0.007 | 2.25E-15 | -0.006 | 0.016 | 0.712 |
|  | rs7901695 | T | C | -0.275 | 0.017 | 8.18E-62 | -0.007 | 0.018 | 0.659 |
|  | rs80196932 | T | C | 0.060 | 0.008 | 7.57E-13 | -0.001 | 0.023 | 0.993 |
|  | rs8026714 | G | A | -0.066 | 0.007 | 1.06E-22 | 0.026 | 0.024 | 0.253 |
|  | rs8043085 | G | T | -0.052 | 0.007 | 2.06E-14 | -0.028 | 0.019 | 0.141 |
|  | rs896852 | G | T | 0.042 | 0.007 | 6.42E-09 | -0.007 | 0.016 | 0.669 |
|  | rs9316706 | A | G | 0.041 | 0.007 | 3.33E-09 | 0.017 | 0.021 | 0.428 |
|  | rs9350271 | G | A | -0.193 | 0.007 | 4.95E-183 | 0.016 | 0.017 | 0.332 |
|  | rs9379084 | G | A | 0.072 | 0.009 | 2.20E-14 | 0.008 | 0.026 | 0.724 |
|  | rs9390022 | T | C | 0.048 | 0.008 | 6.35E-09 | -0.013 | 0.017 | 0.419 |
|  | rs9461650 | G | A | 0.060 | 0.009 | 1.99E-12 | 0.044 | 0.023 | 0.059 |
|  | rs9523295 | G | A | 0.078 | 0.009 | 7.24E-18 | 0.018 | 0.019 | 0.340 |
|  | rs9788635 | C | T | 0.058 | 0.008 | 9.43E-14 | -0.041 | 0.025 | 0.111 |
|  | rs9948462 | C | T | -0.047 | 0.008 | 8.70E-10 | 0.001 | 0.017 | 0.947 |
| *Alcaligenaceae* | rs10011838 | G | A | 0.073 | 0.007 | 1.43E-27 | -0.004 | 0.012 | 0.759 |
|  | rs1016565 | G | A | -0.038 | 0.007 | 2.18E-08 | 0.007 | 0.012 | 0.582 |
|  | rs10507349 | G | A | 0.064 | 0.007 | 1.69E-21 | 0.013 | 0.013 | 0.237 |
|  | rs10852123 | A | C | 0.060 | 0.008 | 8.38E-13 | -0.002 | 0.013 | 0.948 |
|  | rs10860209 | C | A | 0.040 | 0.007 | 5.67E-09 | 0.003 | 0.015 | 0.733 |
|  | rs10886863 | T | C | -0.060 | 0.007 | 5.28E-17 | -0.011 | 0.034 | 0.887 |
|  | rs10938398 | G | A | -0.046 | 0.007 | 3.84E-10 | 0.021 | 0.011 | 0.057 |
|  | rs10950550 | T | G | 0.065 | 0.007 | 1.75E-19 | -0.013 | 0.011 | 0.247 |
|  | rs10965248 | T | C | 0.183 | 0.007 | 4.42E-164 | 0.015 | 0.014 | 0.268 |
|  | rs111246699 | G | A | -0.061 | 0.008 | 1.54E-15 | -0.015 | 0.013 | 0.233 |
|  | rs113154802 | C | T | 0.060 | 0.011 | 3.51E-08 | 0.035 | 0.019 | 0.044 |
|  | rs1182444 | A | G | -0.047 | 0.007 | 1.67E-12 | -0.012 | 0.011 | 0.314 |
|  | rs11926494 | G | A | 0.112 | 0.009 | 2.69E-37 | -0.016 | 0.019 | 0.428 |
|  | rs1206684 | G | A | 0.040 | 0.007 | 4.42E-09 | 0.020 | 0.011 | 0.072 |
|  | rs123378 | G | A | 0.053 | 0.008 | 2.22E-10 | 0.003 | 0.012 | 0.855 |
|  | rs1260326 | T | C | -0.063 | 0.007 | 1.01E-21 | -0.007 | 0.011 | 0.539 |
|  | rs12610052 | T | C | -0.046 | 0.008 | 1.97E-09 | 0.030 | 0.041 | 0.336 |
|  | rs12625671 | T | C | -0.066 | 0.007 | 2.25E-21 | -0.023 | 0.016 | 0.160 |
|  | rs1266488 | T | C | 0.044 | 0.007 | 6.29E-10 | -0.015 | 0.034 | 0.863 |
|  | rs12698877 | A | G | -0.067 | 0.007 | 6.96E-22 | 0.005 | 0.012 | 0.677 |
|  | rs12818766 | G | A | -0.054 | 0.009 | 2.46E-10 | 0.041 | 0.015 | 0.004 |
|  | rs13092876 | G | A | -0.126 | 0.007 | 1.91E-66 | 0.002 | 0.012 | 0.833 |
|  | rs13266634 | C | T | 0.116 | 0.007 | 3.72E-67 | 0.018 | 0.012 | 0.121 |
|  | rs1328412 | T | C | 0.097 | 0.015 | 6.41E-11 | 0.002 | 0.022 | 0.995 |
|  | rs1421085 | T | C | -0.130 | 0.009 | 1.55E-48 | -0.006 | 0.011 | 0.564 |
|  | rs1426371 | G | A | 0.048 | 0.007 | 7.76E-12 | 0.006 | 0.013 | 0.709 |
|  | rs1459513 | A | C | -0.046 | 0.007 | 3.73E-11 | 0.012 | 0.024 | 0.710 |
|  | rs147707338 | C | T | -0.087 | 0.014 | 6.47E-10 | -0.007 | 0.019 | 0.594 |
|  | rs1513275 | T | C | -0.057 | 0.009 | 2.76E-11 | -0.005 | 0.013 | 0.685 |
|  | rs16884229 | T | C | -0.097 | 0.007 | 1.15E-43 | 0.014 | 0.014 | 0.316 |
|  | rs17168486 | C | T | -0.064 | 0.007 | 8.23E-22 | 0.001 | 0.014 | 0.952 |
|  | rs1850421 | A | C | 0.044 | 0.007 | 1.41E-09 | 0.000 | 0.012 | 0.973 |
|  | rs2074120 | A | C | 0.041 | 0.007 | 8.38E-09 | -0.007 | 0.011 | 0.561 |
|  | rs2092518 | G | T | 0.046 | 0.007 | 1.39E-10 | 0.004 | 0.011 | 0.693 |
|  | rs2126736 | A | G | 0.038 | 0.007 | 1.84E-08 | 0.003 | 0.011 | 0.824 |
|  | rs2240885 | G | A | -0.042 | 0.007 | 2.79E-09 | 0.017 | 0.013 | 0.207 |
|  | rs2269245 | G | A | 0.054 | 0.009 | 5.40E-10 | -0.015 | 0.014 | 0.328 |
|  | rs2327777 | T | C | 0.050 | 0.007 | 1.06E-13 | 0.002 | 0.011 | 0.821 |
|  | rs2583934 | G | T | -0.058 | 0.007 | 4.95E-16 | -0.032 | 0.015 | 0.020 |
|  | rs261982 | C | T | -0.040 | 0.007 | 3.12E-09 | -0.020 | 0.011 | 0.072 |
|  | rs2706710 | C | T | -0.071 | 0.013 | 1.67E-08 | 0.029 | 0.014 | 0.047 |
|  | rs2796441 | G | A | 0.075 | 0.007 | 1.43E-28 | -0.026 | 0.011 | 0.018 |
|  | rs28599782 | G | A | -0.067 | 0.008 | 4.64E-16 | 0.009 | 0.030 | 0.890 |
|  | rs28691713 | C | T | 0.066 | 0.008 | 1.79E-17 | -0.018 | 0.011 | 0.113 |
|  | rs2908279 | T | G | -0.046 | 0.007 | 8.42E-11 | 0.012 | 0.011 | 0.289 |
|  | rs2980883 | T | G | 0.042 | 0.007 | 3.93E-09 | -0.011 | 0.015 | 0.440 |
|  | rs3094508 | T | C | -0.059 | 0.008 | 1.31E-13 | 0.023 | 0.011 | 0.043 |
|  | rs329122 | G | A | -0.039 | 0.007 | 2.22E-08 | -0.003 | 0.011 | 0.816 |
|  | rs34811727 | G | T | -0.076 | 0.013 | 3.32E-09 | -0.027 | 0.022 | 0.257 |
|  | rs349359 | A | C | -0.043 | 0.008 | 3.05E-08 | 0.010 | 0.017 | 0.585 |
|  | rs35589574 | C | T | -0.171 | 0.011 | 1.58E-55 | -0.015 | 0.013 | 0.260 |
|  | rs3735641 | A | G | -0.069 | 0.008 | 3.62E-19 | 0.019 | 0.011 | 0.097 |
|  | rs3751236 | G | A | 0.067 | 0.007 | 6.58E-21 | -0.002 | 0.014 | 0.886 |
|  | rs3852529 | C | T | -0.099 | 0.010 | 2.07E-24 | -0.018 | 0.019 | 0.436 |
|  | rs3887925 | C | T | -0.040 | 0.007 | 3.12E-09 | 0.010 | 0.011 | 0.349 |
|  | rs391933 | G | A | 0.037 | 0.007 | 1.46E-08 | -0.010 | 0.011 | 0.355 |
|  | rs4273712 | A | G | -0.047 | 0.007 | 2.56E-12 | -0.028 | 0.012 | 0.021 |
|  | rs4499362 | C | T | 0.044 | 0.007 | 1.53E-09 | 0.012 | 0.014 | 0.386 |
|  | rs476828 | T | C | -0.084 | 0.008 | 4.81E-27 | -0.001 | 0.013 | 0.897 |
|  | rs4930974 | C | T | -0.069 | 0.008 | 4.07E-19 | -0.001 | 0.013 | 0.993 |
|  | rs532504 | G | A | -0.055 | 0.008 | 7.39E-12 | 0.007 | 0.014 | 0.526 |
|  | rs55700915 | G | A | -0.040 | 0.007 | 1.50E-08 | -0.028 | 0.013 | 0.030 |
|  | rs58524310 | A | G | -0.047 | 0.007 | 8.41E-11 | -0.011 | 0.015 | 0.516 |
|  | rs58718028 | C | T | -0.073 | 0.007 | 3.35E-25 | 0.029 | 0.013 | 0.026 |
|  | rs6012878 | G | A | 0.041 | 0.007 | 4.32E-09 | -0.018 | 0.011 | 0.097 |
|  | rs60573766 | C | T | 0.044 | 0.007 | 4.30E-10 | 0.010 | 0.016 | 0.535 |
|  | rs61779313 | T | C | -0.060 | 0.009 | 5.59E-11 | 0.006 | 0.013 | 0.506 |
|  | rs61975988 | A | G | 0.040 | 0.007 | 1.97E-09 | -0.007 | 0.012 | 0.567 |
|  | rs62173901 | A | G | 0.123 | 0.020 | 6.58E-10 | 0.016 | 0.016 | 0.318 |
|  | rs62405419 | G | T | -0.044 | 0.008 | 3.79E-09 | -0.014 | 0.017 | 0.426 |
|  | rs633862 | C | T | -0.039 | 0.007 | 1.26E-08 | -0.001 | 0.011 | 0.901 |
|  | rs6416749 | C | T | 0.052 | 0.008 | 3.40E-12 | -0.010 | 0.012 | 0.420 |
|  | rs6731688 | A | C | -0.098 | 0.012 | 2.55E-17 | -0.010 | 0.015 | 0.470 |
|  | rs703980 | G | A | 0.060 | 0.007 | 6.55E-19 | 0.014 | 0.011 | 0.189 |
|  | rs7107784 | G | A | 0.086 | 0.012 | 2.60E-13 | -0.008 | 0.012 | 0.439 |
|  | rs7109575 | G | A | 0.141 | 0.015 | 5.46E-21 | -0.039 | 0.015 | 0.013 |
|  | rs7250869 | T | C | 0.056 | 0.007 | 2.29E-16 | 0.004 | 0.012 | 0.734 |
|  | rs7304270 | C | T | 0.068 | 0.010 | 1.04E-12 | 0.033 | 0.058 | 0.581 |
|  | rs73085586 | G | A | 0.043 | 0.007 | 1.66E-09 | 0.010 | 0.014 | 0.534 |
|  | rs7313668 | G | T | -0.045 | 0.007 | 4.91E-11 | -0.009 | 0.011 | 0.422 |
|  | rs73347525 | A | G | 0.059 | 0.009 | 7.46E-11 | 0.017 | 0.014 | 0.247 |
|  | rs742762 | A | C | 0.075 | 0.008 | 1.79E-22 | -0.017 | 0.017 | 0.366 |
|  | rs7501939 | T | C | 0.120 | 0.007 | 1.60E-60 | 0.009 | 0.011 | 0.433 |
|  | rs75990271 | T | C | 0.066 | 0.010 | 3.22E-11 | -0.002 | 0.025 | 0.768 |
|  | rs7656416 | C | T | 0.100 | 0.007 | 9.01E-42 | 0.000 | 0.031 | 0.781 |
|  | rs76704029 | T | C | 0.055 | 0.010 | 3.39E-08 | -0.014 | 0.025 | 0.518 |
|  | rs76878791 | A | G | -0.053 | 0.007 | 2.02E-13 | 0.012 | 0.048 | 0.895 |
|  | rs77789961 | C | T | -0.047 | 0.008 | 1.92E-08 | 0.030 | 0.040 | 0.482 |
|  | rs7787720 | C | T | -0.054 | 0.007 | 2.25E-15 | 0.003 | 0.011 | 0.789 |
|  | rs7901695 | T | C | -0.275 | 0.017 | 8.18E-62 | 0.015 | 0.012 | 0.215 |
|  | rs80196932 | T | C | 0.060 | 0.008 | 7.57E-13 | 0.025 | 0.015 | 0.115 |
|  | rs8026714 | G | A | -0.066 | 0.007 | 1.06E-22 | -0.023 | 0.016 | 0.153 |
|  | rs8043085 | G | T | -0.052 | 0.007 | 2.06E-14 | 0.009 | 0.013 | 0.445 |
|  | rs896852 | G | T | 0.042 | 0.007 | 6.42E-09 | -0.012 | 0.011 | 0.319 |
|  | rs9316706 | A | G | 0.041 | 0.007 | 3.33E-09 | 0.008 | 0.014 | 0.562 |
|  | rs9350271 | G | A | -0.193 | 0.007 | 4.95E-183 | 0.005 | 0.012 | 0.652 |
|  | rs9379084 | G | A | 0.072 | 0.009 | 2.20E-14 | 0.014 | 0.018 | 0.423 |
|  | rs9390022 | T | C | 0.048 | 0.008 | 6.35E-09 | -0.008 | 0.012 | 0.423 |
|  | rs9461650 | G | A | 0.060 | 0.009 | 1.99E-12 | 0.000 | 0.015 | 0.932 |
|  | rs9523295 | G | A | 0.078 | 0.009 | 7.24E-18 | -0.002 | 0.013 | 0.823 |
|  | rs9788635 | C | T | 0.058 | 0.008 | 9.43E-14 | 0.023 | 0.017 | 0.225 |
|  | rs9948462 | C | T | -0.047 | 0.008 | 8.70E-10 | -0.004 | 0.011 | 0.726 |
| *Bacteroidaceae* | rs10011838 | G | A | 0.073 | 0.007 | 1.43E-27 | 0.007 | 0.011 | 0.536 |
|  | rs1016565 | G | A | -0.038 | 0.007 | 2.18E-08 | -0.012 | 0.011 | 0.267 |
|  | rs10507349 | G | A | 0.064 | 0.007 | 1.69E-21 | -0.019 | 0.012 | 0.116 |
|  | rs10852123 | A | C | 0.060 | 0.008 | 8.38E-13 | 0.011 | 0.012 | 0.326 |
|  | rs10860209 | C | A | 0.040 | 0.007 | 5.67E-09 | 0.010 | 0.014 | 0.562 |
|  | rs10886863 | T | C | -0.060 | 0.007 | 5.28E-17 | -0.038 | 0.033 | 0.172 |
|  | rs10938398 | G | A | -0.046 | 0.007 | 3.84E-10 | 0.022 | 0.011 | 0.037 |
|  | rs10950550 | T | G | 0.065 | 0.007 | 1.75E-19 | 0.021 | 0.011 | 0.048 |
|  | rs10965248 | T | C | 0.183 | 0.007 | 4.42E-164 | 0.017 | 0.014 | 0.238 |
|  | rs111246699 | G | A | -0.061 | 0.008 | 1.54E-15 | 0.008 | 0.012 | 0.571 |
|  | rs113154802 | C | T | 0.060 | 0.011 | 3.51E-08 | -0.008 | 0.018 | 0.595 |
|  | rs1182444 | A | G | -0.047 | 0.007 | 1.67E-12 | -0.007 | 0.011 | 0.535 |
|  | rs11926494 | G | A | 0.112 | 0.009 | 2.69E-37 | -0.005 | 0.018 | 0.756 |
|  | rs1206684 | G | A | 0.040 | 0.007 | 4.42E-09 | 0.011 | 0.011 | 0.287 |
|  | rs123378 | G | A | 0.053 | 0.008 | 2.22E-10 | -0.008 | 0.011 | 0.507 |
|  | rs1260326 | T | C | -0.063 | 0.007 | 1.01E-21 | 0.006 | 0.011 | 0.568 |
|  | rs12610052 | T | C | -0.046 | 0.008 | 1.97E-09 | 0.021 | 0.039 | 0.616 |
|  | rs12625671 | T | C | -0.066 | 0.007 | 2.25E-21 | -0.006 | 0.015 | 0.774 |
|  | rs1266488 | T | C | 0.044 | 0.007 | 6.29E-10 | 0.008 | 0.033 | 0.837 |
|  | rs12698877 | A | G | -0.067 | 0.007 | 6.96E-22 | 0.008 | 0.012 | 0.469 |
|  | rs12818766 | G | A | -0.054 | 0.009 | 2.46E-10 | -0.010 | 0.014 | 0.472 |
|  | rs13092876 | G | A | -0.126 | 0.007 | 1.91E-66 | 0.002 | 0.011 | 0.873 |
|  | rs13266634 | C | T | 0.116 | 0.007 | 3.72E-67 | 0.011 | 0.011 | 0.366 |
|  | rs1328412 | T | C | 0.097 | 0.015 | 6.41E-11 | 0.027 | 0.021 | 0.178 |
|  | rs1421085 | T | C | -0.130 | 0.009 | 1.55E-48 | -0.013 | 0.011 | 0.240 |
|  | rs1426371 | G | A | 0.048 | 0.007 | 7.76E-12 | -0.019 | 0.012 | 0.121 |
|  | rs1459513 | A | C | -0.046 | 0.007 | 3.73E-11 | -0.011 | 0.023 | 0.597 |
|  | rs147707338 | C | T | -0.087 | 0.014 | 6.47E-10 | 0.008 | 0.018 | 0.685 |
|  | rs1513275 | T | C | -0.057 | 0.009 | 2.76E-11 | -0.007 | 0.012 | 0.539 |
|  | rs16884229 | T | C | -0.097 | 0.007 | 1.15E-43 | 0.016 | 0.013 | 0.200 |
|  | rs17168486 | C | T | -0.064 | 0.007 | 8.23E-22 | -0.022 | 0.013 | 0.084 |
|  | rs1850421 | A | C | 0.044 | 0.007 | 1.41E-09 | 0.008 | 0.011 | 0.519 |
|  | rs2074120 | A | C | 0.041 | 0.007 | 8.38E-09 | 0.003 | 0.010 | 0.755 |
|  | rs2092518 | G | T | 0.046 | 0.007 | 1.39E-10 | -0.002 | 0.011 | 0.889 |
|  | rs2126736 | A | G | 0.038 | 0.007 | 1.84E-08 | 0.012 | 0.011 | 0.249 |
|  | rs2240885 | G | A | -0.042 | 0.007 | 2.79E-09 | -0.002 | 0.013 | 0.816 |
|  | rs2269245 | G | A | 0.054 | 0.009 | 5.40E-10 | -0.023 | 0.013 | 0.079 |
|  | rs2327777 | T | C | 0.050 | 0.007 | 1.06E-13 | 0.005 | 0.010 | 0.631 |
|  | rs2583934 | G | T | -0.058 | 0.007 | 4.95E-16 | 0.000 | 0.014 | 0.955 |
|  | rs261982 | C | T | -0.040 | 0.007 | 3.12E-09 | -0.018 | 0.011 | 0.088 |
|  | rs2706710 | C | T | -0.071 | 0.013 | 1.67E-08 | 0.004 | 0.014 | 0.850 |
|  | rs2796441 | G | A | 0.075 | 0.007 | 1.43E-28 | -0.026 | 0.011 | 0.017 |
|  | rs28599782 | G | A | -0.067 | 0.008 | 4.64E-16 | 0.014 | 0.029 | 0.577 |
|  | rs28691713 | C | T | 0.066 | 0.008 | 1.79E-17 | -0.003 | 0.011 | 0.752 |
|  | rs2908279 | T | G | -0.046 | 0.007 | 8.42E-11 | -0.005 | 0.010 | 0.680 |
|  | rs2980883 | T | G | 0.042 | 0.007 | 3.93E-09 | -0.007 | 0.015 | 0.691 |
|  | rs3094508 | T | C | -0.059 | 0.008 | 1.31E-13 | 0.002 | 0.011 | 0.896 |
|  | rs329122 | G | A | -0.039 | 0.007 | 2.22E-08 | 0.013 | 0.011 | 0.220 |
|  | rs34811727 | G | T | -0.076 | 0.013 | 3.32E-09 | -0.024 | 0.021 | 0.238 |
|  | rs349359 | A | C | -0.043 | 0.008 | 3.05E-08 | -0.003 | 0.016 | 0.937 |
|  | rs35589574 | C | T | -0.171 | 0.011 | 1.58E-55 | -0.016 | 0.012 | 0.150 |
|  | rs3735641 | A | G | -0.069 | 0.008 | 3.62E-19 | 0.007 | 0.011 | 0.493 |
|  | rs3751236 | G | A | 0.067 | 0.007 | 6.58E-21 | -0.003 | 0.013 | 0.794 |
|  | rs3852529 | C | T | -0.099 | 0.010 | 2.07E-24 | -0.008 | 0.018 | 0.839 |
|  | rs3887925 | C | T | -0.040 | 0.007 | 3.12E-09 | 0.005 | 0.011 | 0.624 |
|  | rs391933 | G | A | 0.037 | 0.007 | 1.46E-08 | 0.001 | 0.011 | 0.921 |
|  | rs4273712 | A | G | -0.047 | 0.007 | 2.56E-12 | 0.004 | 0.012 | 0.724 |
|  | rs4499362 | C | T | 0.044 | 0.007 | 1.53E-09 | 0.018 | 0.013 | 0.180 |
|  | rs476828 | T | C | -0.084 | 0.008 | 4.81E-27 | -0.020 | 0.012 | 0.104 |
|  | rs4930974 | C | T | -0.069 | 0.008 | 4.07E-19 | -0.015 | 0.013 | 0.263 |
|  | rs532504 | G | A | -0.055 | 0.008 | 7.39E-12 | -0.014 | 0.013 | 0.298 |
|  | rs55700915 | G | A | -0.040 | 0.007 | 1.50E-08 | -0.010 | 0.013 | 0.372 |
|  | rs58524310 | A | G | -0.047 | 0.007 | 8.41E-11 | -0.003 | 0.015 | 0.833 |
|  | rs58718028 | C | T | -0.073 | 0.007 | 3.35E-25 | 0.002 | 0.012 | 0.919 |
|  | rs6012878 | G | A | 0.041 | 0.007 | 4.32E-09 | -0.008 | 0.011 | 0.453 |
|  | rs60573766 | C | T | 0.044 | 0.007 | 4.30E-10 | 0.006 | 0.015 | 0.649 |
|  | rs61779313 | T | C | -0.060 | 0.009 | 5.59E-11 | 0.021 | 0.012 | 0.089 |
|  | rs61975988 | A | G | 0.040 | 0.007 | 1.97E-09 | -0.001 | 0.012 | 0.925 |
|  | rs62173901 | A | G | 0.123 | 0.020 | 6.58E-10 | 0.008 | 0.015 | 0.562 |
|  | rs62405419 | G | T | -0.044 | 0.008 | 3.79E-09 | -0.017 | 0.016 | 0.281 |
|  | rs633862 | C | T | -0.039 | 0.007 | 1.26E-08 | 0.021 | 0.011 | 0.047 |
|  | rs6416749 | C | T | 0.052 | 0.008 | 3.40E-12 | 0.004 | 0.011 | 0.728 |
|  | rs6731688 | A | C | -0.098 | 0.012 | 2.55E-17 | 0.007 | 0.014 | 0.671 |
|  | rs703980 | G | A | 0.060 | 0.007 | 6.55E-19 | -0.021 | 0.011 | 0.040 |
|  | rs7107784 | G | A | 0.086 | 0.012 | 2.60E-13 | -0.009 | 0.012 | 0.425 |
|  | rs7109575 | G | A | 0.141 | 0.015 | 5.46E-21 | -0.021 | 0.015 | 0.149 |
|  | rs7250869 | T | C | 0.056 | 0.007 | 2.29E-16 | 0.007 | 0.011 | 0.563 |
|  | rs7304270 | C | T | 0.068 | 0.010 | 1.04E-12 | 0.061 | 0.057 | 0.266 |
|  | rs73085586 | G | A | 0.043 | 0.007 | 1.66E-09 | 0.023 | 0.013 | 0.071 |
|  | rs7313668 | G | T | -0.045 | 0.007 | 4.91E-11 | 0.003 | 0.011 | 0.797 |
|  | rs73347525 | A | G | 0.059 | 0.009 | 7.46E-11 | 0.025 | 0.014 | 0.072 |
|  | rs742762 | A | C | 0.075 | 0.008 | 1.79E-22 | -0.005 | 0.017 | 0.873 |
|  | rs7501939 | T | C | 0.120 | 0.007 | 1.60E-60 | 0.007 | 0.011 | 0.543 |
|  | rs75990271 | T | C | 0.066 | 0.010 | 3.22E-11 | -0.017 | 0.024 | 0.434 |
|  | rs7656416 | C | T | 0.100 | 0.007 | 9.01E-42 | -0.032 | 0.030 | 0.211 |
|  | rs76704029 | T | C | 0.055 | 0.010 | 3.39E-08 | -0.047 | 0.024 | 0.062 |
|  | rs76878791 | A | G | -0.053 | 0.007 | 2.02E-13 | 0.015 | 0.046 | 0.619 |
|  | rs77789961 | C | T | -0.047 | 0.008 | 1.92E-08 | -0.048 | 0.039 | 0.301 |
|  | rs7787720 | C | T | -0.054 | 0.007 | 2.25E-15 | 0.002 | 0.011 | 0.845 |
|  | rs7901695 | T | C | -0.275 | 0.017 | 8.18E-62 | 0.015 | 0.011 | 0.186 |
|  | rs80196932 | T | C | 0.060 | 0.008 | 7.57E-13 | -0.010 | 0.015 | 0.457 |
|  | rs8026714 | G | A | -0.066 | 0.007 | 1.06E-22 | 0.007 | 0.015 | 0.674 |
|  | rs8043085 | G | T | -0.052 | 0.007 | 2.06E-14 | 0.023 | 0.012 | 0.057 |
|  | rs896852 | G | T | 0.042 | 0.007 | 6.42E-09 | -0.002 | 0.011 | 0.897 |
|  | rs9316706 | A | G | 0.041 | 0.007 | 3.33E-09 | 0.005 | 0.014 | 0.685 |
|  | rs9350271 | G | A | -0.193 | 0.007 | 4.95E-183 | 0.002 | 0.011 | 0.912 |
|  | rs9379084 | G | A | 0.072 | 0.009 | 2.20E-14 | 0.003 | 0.017 | 0.994 |
|  | rs9390022 | T | C | 0.048 | 0.008 | 6.35E-09 | -0.011 | 0.011 | 0.323 |
|  | rs9461650 | G | A | 0.060 | 0.009 | 1.99E-12 | 0.002 | 0.014 | 0.835 |
|  | rs9523295 | G | A | 0.078 | 0.009 | 7.24E-18 | -0.015 | 0.012 | 0.192 |
|  | rs9788635 | C | T | 0.058 | 0.008 | 9.43E-14 | -0.019 | 0.016 | 0.226 |
|  | rs9948462 | C | T | -0.047 | 0.008 | 8.70E-10 | -0.009 | 0.011 | 0.437 |
| *Bacteroidales_S24-7* | rs10011838 | G | A | 0.073 | 0.007 | 1.43E-27 | 0.020 | 0.017 | 0.251 |
|  | rs1016565 | G | A | -0.038 | 0.007 | 2.18E-08 | 0.006 | 0.017 | 0.722 |
|  | rs10507349 | G | A | 0.064 | 0.007 | 1.69E-21 | 0.007 | 0.018 | 0.606 |
|  | rs10852123 | A | C | 0.060 | 0.008 | 8.38E-13 | -0.015 | 0.019 | 0.468 |
|  | rs10860209 | C | A | 0.040 | 0.007 | 5.67E-09 | 0.009 | 0.022 | 0.687 |
|  | rs10938398 | G | A | -0.046 | 0.007 | 3.84E-10 | -0.021 | 0.016 | 0.185 |
|  | rs10950550 | T | G | 0.065 | 0.007 | 1.75E-19 | 0.021 | 0.016 | 0.199 |
|  | rs10965248 | T | C | 0.183 | 0.007 | 4.42E-164 | 0.007 | 0.021 | 0.643 |
|  | rs111246699 | G | A | -0.061 | 0.008 | 1.54E-15 | -0.015 | 0.019 | 0.418 |
|  | rs113154802 | C | T | 0.060 | 0.011 | 3.51E-08 | 0.013 | 0.027 | 0.579 |
|  | rs1182444 | A | G | -0.047 | 0.007 | 1.67E-12 | -0.014 | 0.017 | 0.421 |
|  | rs11926494 | G | A | 0.112 | 0.009 | 2.69E-37 | 0.009 | 0.027 | 0.720 |
|  | rs1206684 | G | A | 0.040 | 0.007 | 4.42E-09 | 0.018 | 0.016 | 0.286 |
|  | rs123378 | G | A | 0.053 | 0.008 | 2.22E-10 | 0.002 | 0.017 | 0.873 |
|  | rs1260326 | T | C | -0.063 | 0.007 | 1.01E-21 | -0.022 | 0.016 | 0.180 |
|  | rs12625671 | T | C | -0.066 | 0.007 | 2.25E-21 | 0.029 | 0.023 | 0.118 |
|  | rs12698877 | A | G | -0.067 | 0.007 | 6.96E-22 | -0.007 | 0.018 | 0.711 |
|  | rs12818766 | G | A | -0.054 | 0.009 | 2.46E-10 | 0.031 | 0.021 | 0.184 |
|  | rs13092876 | G | A | -0.126 | 0.007 | 1.91E-66 | -0.031 | 0.017 | 0.074 |
|  | rs13266634 | C | T | 0.116 | 0.007 | 3.72E-67 | -0.036 | 0.017 | 0.037 |
|  | rs1328412 | T | C | 0.097 | 0.015 | 6.41E-11 | 0.023 | 0.031 | 0.386 |
|  | rs1421085 | T | C | -0.130 | 0.009 | 1.55E-48 | -0.022 | 0.016 | 0.200 |
|  | rs1426371 | G | A | 0.048 | 0.007 | 7.76E-12 | 0.017 | 0.018 | 0.375 |
|  | rs1459513 | A | C | -0.046 | 0.007 | 3.73E-11 | 0.028 | 0.034 | 0.410 |
|  | rs147707338 | C | T | -0.087 | 0.014 | 6.47E-10 | 0.013 | 0.027 | 0.711 |
|  | rs1513275 | T | C | -0.057 | 0.009 | 2.76E-11 | 0.004 | 0.019 | 0.796 |
|  | rs16884229 | T | C | -0.097 | 0.007 | 1.15E-43 | -0.027 | 0.020 | 0.194 |
|  | rs17168486 | C | T | -0.064 | 0.007 | 8.23E-22 | 0.013 | 0.020 | 0.537 |
|  | rs1850421 | A | C | 0.044 | 0.007 | 1.41E-09 | -0.006 | 0.017 | 0.721 |
|  | rs2074120 | A | C | 0.041 | 0.007 | 8.38E-09 | 0.001 | 0.016 | 0.943 |
|  | rs2092518 | G | T | 0.046 | 0.007 | 1.39E-10 | -0.012 | 0.016 | 0.467 |
|  | rs2126736 | A | G | 0.038 | 0.007 | 1.84E-08 | 0.015 | 0.016 | 0.350 |
|  | rs2240885 | G | A | -0.042 | 0.007 | 2.79E-09 | -0.013 | 0.019 | 0.435 |
|  | rs2269245 | G | A | 0.054 | 0.009 | 5.40E-10 | 0.020 | 0.020 | 0.287 |
|  | rs2327777 | T | C | 0.050 | 0.007 | 1.06E-13 | -0.023 | 0.016 | 0.155 |
|  | rs2583934 | G | T | -0.058 | 0.007 | 4.95E-16 | -0.002 | 0.022 | 0.869 |
|  | rs261982 | C | T | -0.040 | 0.007 | 3.12E-09 | 0.015 | 0.016 | 0.376 |
|  | rs2706710 | C | T | -0.071 | 0.013 | 1.67E-08 | 0.049 | 0.021 | 0.024 |
|  | rs2796441 | G | A | 0.075 | 0.007 | 1.43E-28 | -0.013 | 0.016 | 0.429 |
|  | rs28691713 | C | T | 0.066 | 0.008 | 1.79E-17 | -0.043 | 0.016 | 0.008 |
|  | rs2908279 | T | G | -0.046 | 0.007 | 8.42E-11 | 0.012 | 0.016 | 0.427 |
|  | rs2980883 | T | G | 0.042 | 0.007 | 3.93E-09 | -0.003 | 0.022 | 0.850 |
|  | rs3094508 | T | C | -0.059 | 0.008 | 1.31E-13 | -0.007 | 0.016 | 0.677 |
|  | rs329122 | G | A | -0.039 | 0.007 | 2.22E-08 | -0.009 | 0.016 | 0.628 |
|  | rs34811727 | G | T | -0.076 | 0.013 | 3.32E-09 | -0.031 | 0.032 | 0.318 |
|  | rs349359 | A | C | -0.043 | 0.008 | 3.05E-08 | 0.011 | 0.024 | 0.641 |
|  | rs35589574 | C | T | -0.171 | 0.011 | 1.58E-55 | -0.013 | 0.019 | 0.428 |
|  | rs3735641 | A | G | -0.069 | 0.008 | 3.62E-19 | 0.021 | 0.016 | 0.200 |
|  | rs3751236 | G | A | 0.067 | 0.007 | 6.58E-21 | 0.007 | 0.020 | 0.820 |
|  | rs3852529 | C | T | -0.099 | 0.010 | 2.07E-24 | -0.014 | 0.027 | 0.552 |
|  | rs3887925 | C | T | -0.040 | 0.007 | 3.12E-09 | 0.001 | 0.016 | 0.971 |
|  | rs391933 | G | A | 0.037 | 0.007 | 1.46E-08 | -0.003 | 0.016 | 0.870 |
|  | rs4273712 | A | G | -0.047 | 0.007 | 2.56E-12 | -0.006 | 0.018 | 0.725 |
|  | rs4499362 | C | T | 0.044 | 0.007 | 1.53E-09 | 0.023 | 0.020 | 0.264 |
|  | rs476828 | T | C | -0.084 | 0.008 | 4.81E-27 | 0.025 | 0.019 | 0.188 |
|  | rs4930974 | C | T | -0.069 | 0.008 | 4.07E-19 | -0.019 | 0.019 | 0.306 |
|  | rs532504 | G | A | -0.055 | 0.008 | 7.39E-12 | -0.017 | 0.020 | 0.408 |
|  | rs55700915 | G | A | -0.040 | 0.007 | 1.50E-08 | -0.006 | 0.019 | 0.759 |
|  | rs58524310 | A | G | -0.047 | 0.007 | 8.41E-11 | -0.015 | 0.022 | 0.562 |
|  | rs58718028 | C | T | -0.073 | 0.007 | 3.35E-25 | 0.032 | 0.018 | 0.075 |
|  | rs6012878 | G | A | 0.041 | 0.007 | 4.32E-09 | -0.005 | 0.016 | 0.739 |
|  | rs60573766 | C | T | 0.044 | 0.007 | 4.30E-10 | 0.006 | 0.023 | 0.671 |
|  | rs61779313 | T | C | -0.060 | 0.009 | 5.59E-11 | 0.006 | 0.019 | 0.741 |
|  | rs61975988 | A | G | 0.040 | 0.007 | 1.97E-09 | -0.010 | 0.018 | 0.566 |
|  | rs62173901 | A | G | 0.123 | 0.020 | 6.58E-10 | -0.006 | 0.023 | 0.718 |
|  | rs62405419 | G | T | -0.044 | 0.008 | 3.79E-09 | -0.013 | 0.024 | 0.631 |
|  | rs633862 | C | T | -0.039 | 0.007 | 1.26E-08 | 0.005 | 0.016 | 0.746 |
|  | rs6416749 | C | T | 0.052 | 0.008 | 3.40E-12 | 0.036 | 0.017 | 0.034 |
|  | rs6731688 | A | C | -0.098 | 0.012 | 2.55E-17 | 0.013 | 0.021 | 0.575 |
|  | rs703980 | G | A | 0.060 | 0.007 | 6.55E-19 | 0.024 | 0.016 | 0.133 |
|  | rs7107784 | G | A | 0.086 | 0.012 | 2.60E-13 | 0.025 | 0.018 | 0.162 |
|  | rs7109575 | G | A | 0.141 | 0.015 | 5.46E-21 | 0.011 | 0.022 | 0.752 |
|  | rs7250869 | T | C | 0.056 | 0.007 | 2.29E-16 | 0.023 | 0.017 | 0.189 |
|  | rs73085586 | G | A | 0.043 | 0.007 | 1.66E-09 | -0.020 | 0.020 | 0.245 |
|  | rs7313668 | G | T | -0.045 | 0.007 | 4.91E-11 | 0.026 | 0.016 | 0.118 |
|  | rs73347525 | A | G | 0.059 | 0.009 | 7.46E-11 | -0.005 | 0.020 | 0.760 |
|  | rs742762 | A | C | 0.075 | 0.008 | 1.79E-22 | 0.023 | 0.025 | 0.382 |
|  | rs7501939 | T | C | 0.120 | 0.007 | 1.60E-60 | -0.030 | 0.016 | 0.065 |
|  | rs75990271 | T | C | 0.066 | 0.010 | 3.22E-11 | -0.048 | 0.034 | 0.189 |
|  | rs76704029 | T | C | 0.055 | 0.010 | 3.39E-08 | 0.030 | 0.038 | 0.429 |
|  | rs7787720 | C | T | -0.054 | 0.007 | 2.25E-15 | 0.013 | 0.016 | 0.399 |
|  | rs7901695 | T | C | -0.275 | 0.017 | 8.18E-62 | 0.003 | 0.018 | 0.858 |
|  | rs80196932 | T | C | 0.060 | 0.008 | 7.57E-13 | 0.020 | 0.022 | 0.438 |
|  | rs8026714 | G | A | -0.066 | 0.007 | 1.06E-22 | -0.008 | 0.023 | 0.857 |
|  | rs8043085 | G | T | -0.052 | 0.007 | 2.06E-14 | -0.013 | 0.018 | 0.459 |
|  | rs896852 | G | T | 0.042 | 0.007 | 6.42E-09 | 0.010 | 0.016 | 0.554 |
|  | rs9316706 | A | G | 0.041 | 0.007 | 3.33E-09 | 0.024 | 0.021 | 0.213 |
|  | rs9350271 | G | A | -0.193 | 0.007 | 4.95E-183 | 0.023 | 0.017 | 0.171 |
|  | rs9379084 | G | A | 0.072 | 0.009 | 2.20E-14 | -0.011 | 0.026 | 0.740 |
|  | rs9390022 | T | C | 0.048 | 0.008 | 6.35E-09 | 0.033 | 0.017 | 0.053 |
|  | rs9461650 | G | A | 0.060 | 0.009 | 1.99E-12 | -0.003 | 0.022 | 0.921 |
|  | rs9523295 | G | A | 0.078 | 0.009 | 7.24E-18 | 0.007 | 0.018 | 0.684 |
|  | rs9788635 | C | T | 0.058 | 0.008 | 9.43E-14 | 0.025 | 0.024 | 0.428 |
|  | rs9948462 | C | T | -0.047 | 0.008 | 8.70E-10 | -0.022 | 0.016 | 0.178 |
| *Bifidobacteriaceae* | rs10011838 | G | A | 0.073 | 0.007 | 1.43E-27 | -0.004 | 0.013 | 0.796 |
|  | rs1016565 | G | A | -0.038 | 0.007 | 2.18E-08 | -0.010 | 0.012 | 0.380 |
|  | rs10507349 | G | A | 0.064 | 0.007 | 1.69E-21 | 0.019 | 0.013 | 0.185 |
|  | rs10852123 | A | C | 0.060 | 0.008 | 8.38E-13 | -0.009 | 0.013 | 0.490 |
|  | rs10860209 | C | A | 0.040 | 0.007 | 5.67E-09 | -0.007 | 0.016 | 0.713 |
|  | rs10886863 | T | C | -0.060 | 0.007 | 5.28E-17 | -0.027 | 0.034 | 0.446 |
|  | rs10938398 | G | A | -0.046 | 0.007 | 3.84E-10 | -0.021 | 0.012 | 0.084 |
|  | rs10950550 | T | G | 0.065 | 0.007 | 1.75E-19 | -0.009 | 0.012 | 0.462 |
|  | rs10965248 | T | C | 0.183 | 0.007 | 4.42E-164 | -0.009 | 0.015 | 0.601 |
|  | rs111246699 | G | A | -0.061 | 0.008 | 1.54E-15 | 0.015 | 0.014 | 0.285 |
|  | rs113154802 | C | T | 0.060 | 0.011 | 3.51E-08 | 0.016 | 0.020 | 0.479 |
|  | rs1182444 | A | G | -0.047 | 0.007 | 1.67E-12 | -0.003 | 0.012 | 0.847 |
|  | rs11926494 | G | A | 0.112 | 0.009 | 2.69E-37 | 0.004 | 0.020 | 0.867 |
|  | rs1206684 | G | A | 0.040 | 0.007 | 4.42E-09 | -0.005 | 0.012 | 0.760 |
|  | rs123378 | G | A | 0.053 | 0.008 | 2.22E-10 | 0.022 | 0.013 | 0.077 |
|  | rs1260326 | T | C | -0.063 | 0.007 | 1.01E-21 | -0.001 | 0.012 | 0.896 |
|  | rs12610052 | T | C | -0.046 | 0.008 | 1.97E-09 | -0.097 | 0.041 | 0.020 |
|  | rs12625671 | T | C | -0.066 | 0.007 | 2.25E-21 | -0.014 | 0.017 | 0.392 |
|  | rs1266488 | T | C | 0.044 | 0.007 | 6.29E-10 | 0.008 | 0.036 | 0.876 |
|  | rs12698877 | A | G | -0.067 | 0.007 | 6.96E-22 | 0.021 | 0.013 | 0.102 |
|  | rs12818766 | G | A | -0.054 | 0.009 | 2.46E-10 | 0.002 | 0.015 | 0.921 |
|  | rs13092876 | G | A | -0.126 | 0.007 | 1.91E-66 | 0.014 | 0.012 | 0.255 |
|  | rs13266634 | C | T | 0.116 | 0.007 | 3.72E-67 | -0.013 | 0.013 | 0.314 |
|  | rs1328412 | T | C | 0.097 | 0.015 | 6.41E-11 | -0.037 | 0.023 | 0.105 |
|  | rs1421085 | T | C | -0.130 | 0.009 | 1.55E-48 | -0.010 | 0.012 | 0.413 |
|  | rs1426371 | G | A | 0.048 | 0.007 | 7.76E-12 | 0.014 | 0.013 | 0.315 |
|  | rs1459513 | A | C | -0.046 | 0.007 | 3.73E-11 | -0.010 | 0.025 | 0.810 |
|  | rs147707338 | C | T | -0.087 | 0.014 | 6.47E-10 | -0.004 | 0.020 | 0.869 |
|  | rs1513275 | T | C | -0.057 | 0.009 | 2.76E-11 | 0.021 | 0.014 | 0.124 |
|  | rs16884229 | T | C | -0.097 | 0.007 | 1.15E-43 | 0.016 | 0.015 | 0.266 |
|  | rs17168486 | C | T | -0.064 | 0.007 | 8.23E-22 | 0.039 | 0.015 | 0.007 |
|  | rs1850421 | A | C | 0.044 | 0.007 | 1.41E-09 | 0.002 | 0.013 | 0.839 |
|  | rs2074120 | A | C | 0.041 | 0.007 | 8.38E-09 | -0.004 | 0.012 | 0.708 |
|  | rs2092518 | G | T | 0.046 | 0.007 | 1.39E-10 | -0.002 | 0.012 | 0.856 |
|  | rs2126736 | A | G | 0.038 | 0.007 | 1.84E-08 | -0.022 | 0.012 | 0.059 |
|  | rs2240885 | G | A | -0.042 | 0.007 | 2.79E-09 | 0.014 | 0.014 | 0.313 |
|  | rs2269245 | G | A | 0.054 | 0.009 | 5.40E-10 | 0.014 | 0.014 | 0.372 |
|  | rs2327777 | T | C | 0.050 | 0.007 | 1.06E-13 | 0.017 | 0.012 | 0.138 |
|  | rs2583934 | G | T | -0.058 | 0.007 | 4.95E-16 | 0.011 | 0.016 | 0.468 |
|  | rs261982 | C | T | -0.040 | 0.007 | 3.12E-09 | 0.017 | 0.012 | 0.152 |
|  | rs2706710 | C | T | -0.071 | 0.013 | 1.67E-08 | -0.024 | 0.015 | 0.094 |
|  | rs2796441 | G | A | 0.075 | 0.007 | 1.43E-28 | -0.038 | 0.012 | 0.001 |
|  | rs28599782 | G | A | -0.067 | 0.008 | 4.64E-16 | -0.029 | 0.031 | 0.409 |
|  | rs28691713 | C | T | 0.066 | 0.008 | 1.79E-17 | 0.024 | 0.012 | 0.041 |
|  | rs2908279 | T | G | -0.046 | 0.007 | 8.42E-11 | -0.006 | 0.012 | 0.620 |
|  | rs2980883 | T | G | 0.042 | 0.007 | 3.93E-09 | 0.017 | 0.016 | 0.299 |
|  | rs3094508 | T | C | -0.059 | 0.008 | 1.31E-13 | -0.011 | 0.012 | 0.322 |
|  | rs329122 | G | A | -0.039 | 0.007 | 2.22E-08 | 0.007 | 0.012 | 0.551 |
|  | rs34811727 | G | T | -0.076 | 0.013 | 3.32E-09 | 0.042 | 0.024 | 0.084 |
|  | rs349359 | A | C | -0.043 | 0.008 | 3.05E-08 | 0.002 | 0.018 | 0.924 |
|  | rs35589574 | C | T | -0.171 | 0.011 | 1.58E-55 | -0.005 | 0.014 | 0.835 |
|  | rs3735641 | A | G | -0.069 | 0.008 | 3.62E-19 | -0.017 | 0.012 | 0.149 |
|  | rs3751236 | G | A | 0.067 | 0.007 | 6.58E-21 | -0.007 | 0.014 | 0.637 |
|  | rs3852529 | C | T | -0.099 | 0.010 | 2.07E-24 | -0.011 | 0.020 | 0.662 |
|  | rs3887925 | C | T | -0.040 | 0.007 | 3.12E-09 | -0.004 | 0.012 | 0.701 |
|  | rs391933 | G | A | 0.037 | 0.007 | 1.46E-08 | -0.002 | 0.012 | 0.871 |
|  | rs4273712 | A | G | -0.047 | 0.007 | 2.56E-12 | 0.009 | 0.013 | 0.469 |
|  | rs4499362 | C | T | 0.044 | 0.007 | 1.53E-09 | -0.012 | 0.015 | 0.387 |
|  | rs476828 | T | C | -0.084 | 0.008 | 4.81E-27 | 0.021 | 0.014 | 0.129 |
|  | rs4930974 | C | T | -0.069 | 0.008 | 4.07E-19 | 0.023 | 0.014 | 0.084 |
|  | rs532504 | G | A | -0.055 | 0.008 | 7.39E-12 | 0.001 | 0.014 | 0.984 |
|  | rs55700915 | G | A | -0.040 | 0.007 | 1.50E-08 | 0.028 | 0.014 | 0.029 |
|  | rs58524310 | A | G | -0.047 | 0.007 | 8.41E-11 | 0.018 | 0.016 | 0.257 |
|  | rs58718028 | C | T | -0.073 | 0.007 | 3.35E-25 | -0.002 | 0.013 | 0.858 |
|  | rs6012878 | G | A | 0.041 | 0.007 | 4.32E-09 | 0.001 | 0.012 | 0.962 |
|  | rs60573766 | C | T | 0.044 | 0.007 | 4.30E-10 | 0.029 | 0.017 | 0.092 |
|  | rs61779313 | T | C | -0.060 | 0.009 | 5.59E-11 | -0.011 | 0.014 | 0.468 |
|  | rs61975988 | A | G | 0.040 | 0.007 | 1.97E-09 | 0.007 | 0.013 | 0.610 |
|  | rs62173901 | A | G | 0.123 | 0.020 | 6.58E-10 | -0.004 | 0.017 | 0.638 |
|  | rs62405419 | G | T | -0.044 | 0.008 | 3.79E-09 | -0.008 | 0.018 | 0.815 |
|  | rs633862 | C | T | -0.039 | 0.007 | 1.26E-08 | 0.022 | 0.012 | 0.054 |
|  | rs6416749 | C | T | 0.052 | 0.008 | 3.40E-12 | 0.006 | 0.012 | 0.594 |
|  | rs6731688 | A | C | -0.098 | 0.012 | 2.55E-17 | 0.011 | 0.016 | 0.493 |
|  | rs703980 | G | A | 0.060 | 0.007 | 6.55E-19 | -0.015 | 0.012 | 0.202 |
|  | rs7107784 | G | A | 0.086 | 0.012 | 2.60E-13 | -0.001 | 0.013 | 0.875 |
|  | rs7109575 | G | A | 0.141 | 0.015 | 5.46E-21 | 0.032 | 0.016 | 0.044 |
|  | rs7250869 | T | C | 0.056 | 0.007 | 2.29E-16 | 0.023 | 0.012 | 0.062 |
|  | rs73085586 | G | A | 0.043 | 0.007 | 1.66E-09 | -0.017 | 0.014 | 0.236 |
|  | rs7313668 | G | T | -0.045 | 0.007 | 4.91E-11 | 0.013 | 0.012 | 0.314 |
|  | rs73347525 | A | G | 0.059 | 0.009 | 7.46E-11 | -0.017 | 0.015 | 0.246 |
|  | rs742762 | A | C | 0.075 | 0.008 | 1.79E-22 | 0.007 | 0.018 | 0.658 |
|  | rs7501939 | T | C | 0.120 | 0.007 | 1.60E-60 | 0.008 | 0.012 | 0.507 |
|  | rs75990271 | T | C | 0.066 | 0.010 | 3.22E-11 | -0.019 | 0.026 | 0.492 |
|  | rs7656416 | C | T | 0.100 | 0.007 | 9.01E-42 | -0.001 | 0.033 | 0.809 |
|  | rs76704029 | T | C | 0.055 | 0.010 | 3.39E-08 | -0.014 | 0.026 | 0.694 |
|  | rs76878791 | A | G | -0.053 | 0.007 | 2.02E-13 | 0.040 | 0.049 | 0.413 |
|  | rs77789961 | C | T | -0.047 | 0.008 | 1.92E-08 | 0.023 | 0.041 | 0.843 |
|  | rs7787720 | C | T | -0.054 | 0.007 | 2.25E-15 | -0.019 | 0.012 | 0.096 |
|  | rs7901695 | T | C | -0.275 | 0.017 | 8.18E-62 | -0.035 | 0.013 | 0.006 |
|  | rs80196932 | T | C | 0.060 | 0.008 | 7.57E-13 | -0.014 | 0.016 | 0.408 |
|  | rs8026714 | G | A | -0.066 | 0.007 | 1.06E-22 | 0.028 | 0.017 | 0.106 |
|  | rs8043085 | G | T | -0.052 | 0.007 | 2.06E-14 | 0.018 | 0.014 | 0.203 |
|  | rs896852 | G | T | 0.042 | 0.007 | 6.42E-09 | 0.006 | 0.012 | 0.629 |
|  | rs9316706 | A | G | 0.041 | 0.007 | 3.33E-09 | -0.014 | 0.015 | 0.374 |
|  | rs9350271 | G | A | -0.193 | 0.007 | 4.95E-183 | 0.005 | 0.012 | 0.688 |
|  | rs9379084 | G | A | 0.072 | 0.009 | 2.20E-14 | 0.003 | 0.019 | 0.895 |
|  | rs9390022 | T | C | 0.048 | 0.008 | 6.35E-09 | 0.006 | 0.012 | 0.591 |
|  | rs9461650 | G | A | 0.060 | 0.009 | 1.99E-12 | 0.014 | 0.016 | 0.350 |
|  | rs9523295 | G | A | 0.078 | 0.009 | 7.24E-18 | -0.002 | 0.013 | 0.896 |
|  | rs9788635 | C | T | 0.058 | 0.008 | 9.43E-14 | 0.009 | 0.018 | 0.584 |
|  | rs9948462 | C | T | -0.047 | 0.008 | 8.70E-10 | 0.013 | 0.012 | 0.275 |
| *Christensenellaceae* | rs10011838 | G | A | 0.073 | 0.007 | 1.43E-27 | -0.002 | 0.012 | 0.892 |
|  | rs1016565 | G | A | -0.038 | 0.007 | 2.18E-08 | 0.001 | 0.012 | 0.927 |
|  | rs10507349 | G | A | 0.064 | 0.007 | 1.69E-21 | -0.003 | 0.013 | 0.768 |
|  | rs10852123 | A | C | 0.060 | 0.008 | 8.38E-13 | -0.007 | 0.013 | 0.586 |
|  | rs10860209 | C | A | 0.040 | 0.007 | 5.67E-09 | 0.014 | 0.015 | 0.355 |
|  | rs10886863 | T | C | -0.060 | 0.007 | 5.28E-17 | 0.061 | 0.036 | 0.107 |
|  | rs10938398 | G | A | -0.046 | 0.007 | 3.84E-10 | -0.013 | 0.011 | 0.228 |
|  | rs10950550 | T | G | 0.065 | 0.007 | 1.75E-19 | -0.006 | 0.011 | 0.592 |
|  | rs10965248 | T | C | 0.183 | 0.007 | 4.42E-164 | 0.010 | 0.014 | 0.448 |
|  | rs111246699 | G | A | -0.061 | 0.008 | 1.54E-15 | 0.006 | 0.013 | 0.718 |
|  | rs113154802 | C | T | 0.060 | 0.011 | 3.51E-08 | 0.002 | 0.019 | 0.971 |
|  | rs1182444 | A | G | -0.047 | 0.007 | 1.67E-12 | -0.022 | 0.011 | 0.056 |
|  | rs11926494 | G | A | 0.112 | 0.009 | 2.69E-37 | -0.008 | 0.019 | 0.594 |
|  | rs1206684 | G | A | 0.040 | 0.007 | 4.42E-09 | -0.021 | 0.011 | 0.063 |
|  | rs123378 | G | A | 0.053 | 0.008 | 2.22E-10 | -0.015 | 0.012 | 0.217 |
|  | rs1260326 | T | C | -0.063 | 0.007 | 1.01E-21 | 0.008 | 0.011 | 0.505 |
|  | rs12610052 | T | C | -0.046 | 0.008 | 1.97E-09 | 0.031 | 0.042 | 0.600 |
|  | rs12625671 | T | C | -0.066 | 0.007 | 2.25E-21 | 0.006 | 0.016 | 0.615 |
|  | rs1266488 | T | C | 0.044 | 0.007 | 6.29E-10 | -0.010 | 0.036 | 0.444 |
|  | rs12698877 | A | G | -0.067 | 0.007 | 6.96E-22 | 0.006 | 0.012 | 0.635 |
|  | rs12818766 | G | A | -0.054 | 0.009 | 2.46E-10 | 0.003 | 0.015 | 0.833 |
|  | rs13092876 | G | A | -0.126 | 0.007 | 1.91E-66 | 0.005 | 0.012 | 0.654 |
|  | rs13266634 | C | T | 0.116 | 0.007 | 3.72E-67 | -0.005 | 0.012 | 0.749 |
|  | rs1328412 | T | C | 0.097 | 0.015 | 6.41E-11 | 0.009 | 0.022 | 0.651 |
|  | rs1421085 | T | C | -0.130 | 0.009 | 1.55E-48 | -0.011 | 0.011 | 0.318 |
|  | rs1426371 | G | A | 0.048 | 0.007 | 7.76E-12 | 0.003 | 0.012 | 0.804 |
|  | rs1459513 | A | C | -0.046 | 0.007 | 3.73E-11 | -0.032 | 0.024 | 0.191 |
|  | rs147707338 | C | T | -0.087 | 0.014 | 6.47E-10 | 0.018 | 0.019 | 0.325 |
|  | rs1513275 | T | C | -0.057 | 0.009 | 2.76E-11 | 0.012 | 0.013 | 0.325 |
|  | rs16884229 | T | C | -0.097 | 0.007 | 1.15E-43 | -0.012 | 0.014 | 0.350 |
|  | rs17168486 | C | T | -0.064 | 0.007 | 8.23E-22 | 0.012 | 0.014 | 0.293 |
|  | rs1850421 | A | C | 0.044 | 0.007 | 1.41E-09 | 0.007 | 0.012 | 0.571 |
|  | rs2074120 | A | C | 0.041 | 0.007 | 8.38E-09 | -0.010 | 0.011 | 0.375 |
|  | rs2092518 | G | T | 0.046 | 0.007 | 1.39E-10 | 0.009 | 0.011 | 0.419 |
|  | rs2126736 | A | G | 0.038 | 0.007 | 1.84E-08 | 0.002 | 0.011 | 0.839 |
|  | rs2240885 | G | A | -0.042 | 0.007 | 2.79E-09 | -0.021 | 0.013 | 0.143 |
|  | rs2269245 | G | A | 0.054 | 0.009 | 5.40E-10 | 0.005 | 0.014 | 0.645 |
|  | rs2327777 | T | C | 0.050 | 0.007 | 1.06E-13 | 0.007 | 0.011 | 0.519 |
|  | rs2583934 | G | T | -0.058 | 0.007 | 4.95E-16 | -0.004 | 0.015 | 0.870 |
|  | rs261982 | C | T | -0.040 | 0.007 | 3.12E-09 | -0.018 | 0.011 | 0.107 |
|  | rs2706710 | C | T | -0.071 | 0.013 | 1.67E-08 | 0.004 | 0.014 | 0.746 |
|  | rs2796441 | G | A | 0.075 | 0.007 | 1.43E-28 | 0.010 | 0.011 | 0.373 |
|  | rs28599782 | G | A | -0.067 | 0.008 | 4.64E-16 | -0.022 | 0.031 | 0.374 |
|  | rs28691713 | C | T | 0.066 | 0.008 | 1.79E-17 | -0.002 | 0.011 | 0.859 |
|  | rs2908279 | T | G | -0.046 | 0.007 | 8.42E-11 | -0.010 | 0.011 | 0.343 |
|  | rs2980883 | T | G | 0.042 | 0.007 | 3.93E-09 | 0.006 | 0.015 | 0.710 |
|  | rs3094508 | T | C | -0.059 | 0.008 | 1.31E-13 | -0.014 | 0.011 | 0.208 |
|  | rs329122 | G | A | -0.039 | 0.007 | 2.22E-08 | 0.025 | 0.011 | 0.024 |
|  | rs34811727 | G | T | -0.076 | 0.013 | 3.32E-09 | -0.012 | 0.022 | 0.628 |
|  | rs349359 | A | C | -0.043 | 0.008 | 3.05E-08 | -0.020 | 0.017 | 0.238 |
|  | rs35589574 | C | T | -0.171 | 0.011 | 1.58E-55 | 0.008 | 0.013 | 0.613 |
|  | rs3735641 | A | G | -0.069 | 0.008 | 3.62E-19 | -0.011 | 0.011 | 0.333 |
|  | rs3751236 | G | A | 0.067 | 0.007 | 6.58E-21 | -0.027 | 0.014 | 0.052 |
|  | rs3852529 | C | T | -0.099 | 0.010 | 2.07E-24 | 0.006 | 0.019 | 0.896 |
|  | rs3887925 | C | T | -0.040 | 0.007 | 3.12E-09 | -0.001 | 0.011 | 0.955 |
|  | rs391933 | G | A | 0.037 | 0.007 | 1.46E-08 | 0.014 | 0.011 | 0.196 |
|  | rs4273712 | A | G | -0.047 | 0.007 | 2.56E-12 | -0.012 | 0.012 | 0.312 |
|  | rs4499362 | C | T | 0.044 | 0.007 | 1.53E-09 | 0.006 | 0.014 | 0.643 |
|  | rs476828 | T | C | -0.084 | 0.008 | 4.81E-27 | -0.008 | 0.013 | 0.521 |
|  | rs4930974 | C | T | -0.069 | 0.008 | 4.07E-19 | 0.023 | 0.013 | 0.080 |
|  | rs532504 | G | A | -0.055 | 0.008 | 7.39E-12 | -0.003 | 0.014 | 0.836 |
|  | rs55700915 | G | A | -0.040 | 0.007 | 1.50E-08 | 0.012 | 0.013 | 0.422 |
|  | rs58524310 | A | G | -0.047 | 0.007 | 8.41E-11 | 0.021 | 0.015 | 0.165 |
|  | rs58718028 | C | T | -0.073 | 0.007 | 3.35E-25 | -0.021 | 0.013 | 0.105 |
|  | rs6012878 | G | A | 0.041 | 0.007 | 4.32E-09 | -0.018 | 0.011 | 0.108 |
|  | rs60573766 | C | T | 0.044 | 0.007 | 4.30E-10 | -0.002 | 0.016 | 0.883 |
|  | rs61779313 | T | C | -0.060 | 0.009 | 5.59E-11 | -0.019 | 0.013 | 0.125 |
|  | rs61975988 | A | G | 0.040 | 0.007 | 1.97E-09 | 0.003 | 0.012 | 0.841 |
|  | rs62173901 | A | G | 0.123 | 0.020 | 6.58E-10 | -0.020 | 0.016 | 0.251 |
|  | rs62405419 | G | T | -0.044 | 0.008 | 3.79E-09 | -0.002 | 0.017 | 0.972 |
|  | rs633862 | C | T | -0.039 | 0.007 | 1.26E-08 | 0.015 | 0.011 | 0.169 |
|  | rs6416749 | C | T | 0.052 | 0.008 | 3.40E-12 | -0.027 | 0.012 | 0.018 |
|  | rs6731688 | A | C | -0.098 | 0.012 | 2.55E-17 | -0.011 | 0.015 | 0.503 |
|  | rs703980 | G | A | 0.060 | 0.007 | 6.55E-19 | 0.012 | 0.011 | 0.278 |
|  | rs7107784 | G | A | 0.086 | 0.012 | 2.60E-13 | 0.005 | 0.012 | 0.731 |
|  | rs7109575 | G | A | 0.141 | 0.015 | 5.46E-21 | 0.023 | 0.015 | 0.157 |
|  | rs7250869 | T | C | 0.056 | 0.007 | 2.29E-16 | -0.001 | 0.012 | 0.960 |
|  | rs73085586 | G | A | 0.043 | 0.007 | 1.66E-09 | -0.003 | 0.014 | 0.824 |
|  | rs7313668 | G | T | -0.045 | 0.007 | 4.91E-11 | 0.012 | 0.011 | 0.266 |
|  | rs73347525 | A | G | 0.059 | 0.009 | 7.46E-11 | 0.019 | 0.014 | 0.200 |
|  | rs742762 | A | C | 0.075 | 0.008 | 1.79E-22 | 0.029 | 0.017 | 0.087 |
|  | rs7501939 | T | C | 0.120 | 0.007 | 1.60E-60 | 0.006 | 0.011 | 0.560 |
|  | rs75990271 | T | C | 0.066 | 0.010 | 3.22E-11 | 0.034 | 0.024 | 0.143 |
|  | rs7656416 | C | T | 0.100 | 0.007 | 9.01E-42 | 0.007 | 0.033 | 0.962 |
|  | rs76704029 | T | C | 0.055 | 0.010 | 3.39E-08 | 0.040 | 0.025 | 0.121 |
|  | rs76878791 | A | G | -0.053 | 0.007 | 2.02E-13 | -0.023 | 0.050 | 0.624 |
|  | rs77789961 | C | T | -0.047 | 0.008 | 1.92E-08 | 0.055 | 0.043 | 0.255 |
|  | rs7787720 | C | T | -0.054 | 0.007 | 2.25E-15 | -0.009 | 0.011 | 0.423 |
|  | rs7901695 | T | C | -0.275 | 0.017 | 8.18E-62 | -0.002 | 0.012 | 0.870 |
|  | rs80196932 | T | C | 0.060 | 0.008 | 7.57E-13 | -0.008 | 0.015 | 0.611 |
|  | rs8026714 | G | A | -0.066 | 0.007 | 1.06E-22 | 0.030 | 0.016 | 0.053 |
|  | rs8043085 | G | T | -0.052 | 0.007 | 2.06E-14 | 0.000 | 0.013 | 0.981 |
|  | rs896852 | G | T | 0.042 | 0.007 | 6.42E-09 | 0.020 | 0.011 | 0.065 |
|  | rs9316706 | A | G | 0.041 | 0.007 | 3.33E-09 | -0.011 | 0.014 | 0.450 |
|  | rs9350271 | G | A | -0.193 | 0.007 | 4.95E-183 | 0.003 | 0.012 | 0.769 |
|  | rs9379084 | G | A | 0.072 | 0.009 | 2.20E-14 | -0.003 | 0.018 | 0.784 |
|  | rs9390022 | T | C | 0.048 | 0.008 | 6.35E-09 | 0.006 | 0.012 | 0.660 |
|  | rs9461650 | G | A | 0.060 | 0.009 | 1.99E-12 | -0.002 | 0.015 | 0.957 |
|  | rs9523295 | G | A | 0.078 | 0.009 | 7.24E-18 | 0.011 | 0.013 | 0.424 |
|  | rs9788635 | C | T | 0.058 | 0.008 | 9.43E-14 | -0.009 | 0.017 | 0.568 |
|  | rs9948462 | C | T | -0.047 | 0.008 | 8.70E-10 | -0.008 | 0.011 | 0.489 |
| *Clostridiaceae_1* | rs10011838 | G | A | 0.073 | 0.007 | 1.43E-27 | -0.009 | 0.013 | 0.482 |
|  | rs1016565 | G | A | -0.038 | 0.007 | 2.18E-08 | 0.023 | 0.013 | 0.066 |
|  | rs10507349 | G | A | 0.064 | 0.007 | 1.69E-21 | 0.013 | 0.014 | 0.378 |
|  | rs10852123 | A | C | 0.060 | 0.008 | 8.38E-13 | 0.007 | 0.014 | 0.667 |
|  | rs10860209 | C | A | 0.040 | 0.007 | 5.67E-09 | 0.009 | 0.016 | 0.596 |
|  | rs10938398 | G | A | -0.046 | 0.007 | 3.84E-10 | 0.022 | 0.012 | 0.060 |
|  | rs10950550 | T | G | 0.065 | 0.007 | 1.75E-19 | 0.015 | 0.012 | 0.208 |
|  | rs10965248 | T | C | 0.183 | 0.007 | 4.42E-164 | -0.013 | 0.015 | 0.487 |
|  | rs111246699 | G | A | -0.061 | 0.008 | 1.54E-15 | 0.020 | 0.014 | 0.149 |
|  | rs113154802 | C | T | 0.060 | 0.011 | 3.51E-08 | 0.024 | 0.020 | 0.196 |
|  | rs1182444 | A | G | -0.047 | 0.007 | 1.67E-12 | 0.008 | 0.012 | 0.542 |
|  | rs11926494 | G | A | 0.112 | 0.009 | 2.69E-37 | 0.011 | 0.020 | 0.609 |
|  | rs1206684 | G | A | 0.040 | 0.007 | 4.42E-09 | -0.024 | 0.012 | 0.059 |
|  | rs123378 | G | A | 0.053 | 0.008 | 2.22E-10 | 0.000 | 0.013 | 0.945 |
|  | rs1260326 | T | C | -0.063 | 0.007 | 1.01E-21 | -0.007 | 0.012 | 0.572 |
|  | rs12610052 | T | C | -0.046 | 0.008 | 1.97E-09 | -0.042 | 0.043 | 0.280 |
|  | rs12625671 | T | C | -0.066 | 0.007 | 2.25E-21 | 0.004 | 0.017 | 0.812 |
|  | rs1266488 | T | C | 0.044 | 0.007 | 6.29E-10 | 0.010 | 0.040 | 0.472 |
|  | rs12698877 | A | G | -0.067 | 0.007 | 6.96E-22 | 0.003 | 0.013 | 0.808 |
|  | rs12818766 | G | A | -0.054 | 0.009 | 2.46E-10 | 0.025 | 0.016 | 0.138 |
|  | rs13092876 | G | A | -0.126 | 0.007 | 1.91E-66 | 0.026 | 0.013 | 0.036 |
|  | rs13266634 | C | T | 0.116 | 0.007 | 3.72E-67 | 0.007 | 0.013 | 0.603 |
|  | rs1328412 | T | C | 0.097 | 0.015 | 6.41E-11 | -0.061 | 0.024 | 0.012 |
|  | rs1421085 | T | C | -0.130 | 0.009 | 1.55E-48 | 0.000 | 0.012 | 0.999 |
|  | rs1426371 | G | A | 0.048 | 0.007 | 7.76E-12 | 0.012 | 0.014 | 0.330 |
|  | rs1459513 | A | C | -0.046 | 0.007 | 3.73E-11 | -0.006 | 0.026 | 0.961 |
|  | rs147707338 | C | T | -0.087 | 0.014 | 6.47E-10 | 0.015 | 0.020 | 0.456 |
|  | rs1513275 | T | C | -0.057 | 0.009 | 2.76E-11 | 0.005 | 0.014 | 0.764 |
|  | rs16884229 | T | C | -0.097 | 0.007 | 1.15E-43 | -0.004 | 0.015 | 0.758 |
|  | rs17168486 | C | T | -0.064 | 0.007 | 8.23E-22 | 0.011 | 0.015 | 0.427 |
|  | rs1850421 | A | C | 0.044 | 0.007 | 1.41E-09 | 0.003 | 0.013 | 0.830 |
|  | rs2074120 | A | C | 0.041 | 0.007 | 8.38E-09 | 0.000 | 0.012 | 0.971 |
|  | rs2092518 | G | T | 0.046 | 0.007 | 1.39E-10 | -0.017 | 0.012 | 0.164 |
|  | rs2126736 | A | G | 0.038 | 0.007 | 1.84E-08 | -0.009 | 0.012 | 0.448 |
|  | rs2240885 | G | A | -0.042 | 0.007 | 2.79E-09 | -0.006 | 0.014 | 0.650 |
|  | rs2269245 | G | A | 0.054 | 0.009 | 5.40E-10 | 0.008 | 0.015 | 0.599 |
|  | rs2327777 | T | C | 0.050 | 0.007 | 1.06E-13 | 0.004 | 0.012 | 0.729 |
|  | rs2583934 | G | T | -0.058 | 0.007 | 4.95E-16 | -0.010 | 0.016 | 0.628 |
|  | rs261982 | C | T | -0.040 | 0.007 | 3.12E-09 | 0.007 | 0.012 | 0.575 |
|  | rs2706710 | C | T | -0.071 | 0.013 | 1.67E-08 | -0.013 | 0.016 | 0.482 |
|  | rs2796441 | G | A | 0.075 | 0.007 | 1.43E-28 | -0.006 | 0.012 | 0.607 |
|  | rs28599782 | G | A | -0.067 | 0.008 | 4.64E-16 | -0.045 | 0.033 | 0.236 |
|  | rs28691713 | C | T | 0.066 | 0.008 | 1.79E-17 | -0.016 | 0.012 | 0.175 |
|  | rs2908279 | T | G | -0.046 | 0.007 | 8.42E-11 | -0.006 | 0.012 | 0.642 |
|  | rs2980883 | T | G | 0.042 | 0.007 | 3.93E-09 | 0.005 | 0.017 | 0.768 |
|  | rs3094508 | T | C | -0.059 | 0.008 | 1.31E-13 | -0.025 | 0.012 | 0.042 |
|  | rs329122 | G | A | -0.039 | 0.007 | 2.22E-08 | 0.001 | 0.012 | 0.896 |
|  | rs34811727 | G | T | -0.076 | 0.013 | 3.32E-09 | 0.043 | 0.024 | 0.087 |
|  | rs349359 | A | C | -0.043 | 0.008 | 3.05E-08 | -0.001 | 0.018 | 0.945 |
|  | rs35589574 | C | T | -0.171 | 0.011 | 1.58E-55 | -0.005 | 0.014 | 0.692 |
|  | rs3735641 | A | G | -0.069 | 0.008 | 3.62E-19 | -0.020 | 0.012 | 0.097 |
|  | rs3751236 | G | A | 0.067 | 0.007 | 6.58E-21 | -0.001 | 0.015 | 0.950 |
|  | rs3852529 | C | T | -0.099 | 0.010 | 2.07E-24 | 0.015 | 0.021 | 0.501 |
|  | rs3887925 | C | T | -0.040 | 0.007 | 3.12E-09 | -0.002 | 0.012 | 0.885 |
|  | rs391933 | G | A | 0.037 | 0.007 | 1.46E-08 | 0.017 | 0.012 | 0.149 |
|  | rs4273712 | A | G | -0.047 | 0.007 | 2.56E-12 | 0.018 | 0.013 | 0.184 |
|  | rs4499362 | C | T | 0.044 | 0.007 | 1.53E-09 | -0.015 | 0.015 | 0.350 |
|  | rs476828 | T | C | -0.084 | 0.008 | 4.81E-27 | -0.027 | 0.014 | 0.048 |
|  | rs4930974 | C | T | -0.069 | 0.008 | 4.07E-19 | -0.014 | 0.014 | 0.335 |
|  | rs532504 | G | A | -0.055 | 0.008 | 7.39E-12 | -0.025 | 0.015 | 0.101 |
|  | rs55700915 | G | A | -0.040 | 0.007 | 1.50E-08 | 0.010 | 0.015 | 0.511 |
|  | rs58524310 | A | G | -0.047 | 0.007 | 8.41E-11 | -0.012 | 0.016 | 0.428 |
|  | rs58718028 | C | T | -0.073 | 0.007 | 3.35E-25 | 0.012 | 0.014 | 0.363 |
|  | rs6012878 | G | A | 0.041 | 0.007 | 4.32E-09 | -0.008 | 0.012 | 0.536 |
|  | rs60573766 | C | T | 0.044 | 0.007 | 4.30E-10 | 0.020 | 0.017 | 0.195 |
|  | rs61779313 | T | C | -0.060 | 0.009 | 5.59E-11 | -0.033 | 0.014 | 0.018 |
|  | rs61975988 | A | G | 0.040 | 0.007 | 1.97E-09 | 0.003 | 0.014 | 0.795 |
|  | rs62173901 | A | G | 0.123 | 0.020 | 6.58E-10 | -0.006 | 0.017 | 0.697 |
|  | rs62405419 | G | T | -0.044 | 0.008 | 3.79E-09 | -0.002 | 0.018 | 0.928 |
|  | rs633862 | C | T | -0.039 | 0.007 | 1.26E-08 | 0.000 | 0.012 | 0.968 |
|  | rs6416749 | C | T | 0.052 | 0.008 | 3.40E-12 | 0.015 | 0.013 | 0.231 |
|  | rs6731688 | A | C | -0.098 | 0.012 | 2.55E-17 | 0.002 | 0.016 | 0.907 |
|  | rs703980 | G | A | 0.060 | 0.007 | 6.55E-19 | 0.013 | 0.012 | 0.283 |
|  | rs7107784 | G | A | 0.086 | 0.012 | 2.60E-13 | 0.000 | 0.013 | 0.995 |
|  | rs7109575 | G | A | 0.141 | 0.015 | 5.46E-21 | -0.010 | 0.016 | 0.555 |
|  | rs7250869 | T | C | 0.056 | 0.007 | 2.29E-16 | 0.011 | 0.013 | 0.383 |
|  | rs73085586 | G | A | 0.043 | 0.007 | 1.66E-09 | 0.010 | 0.015 | 0.476 |
|  | rs7313668 | G | T | -0.045 | 0.007 | 4.91E-11 | 0.019 | 0.012 | 0.105 |
|  | rs73347525 | A | G | 0.059 | 0.009 | 7.46E-11 | 0.001 | 0.015 | 0.972 |
|  | rs742762 | A | C | 0.075 | 0.008 | 1.79E-22 | 0.032 | 0.019 | 0.098 |
|  | rs7501939 | T | C | 0.120 | 0.007 | 1.60E-60 | 0.005 | 0.012 | 0.703 |
|  | rs75990271 | T | C | 0.066 | 0.010 | 3.22E-11 | 0.005 | 0.026 | 0.740 |
|  | rs7656416 | C | T | 0.100 | 0.007 | 9.01E-42 | 0.019 | 0.036 | 0.425 |
|  | rs76704029 | T | C | 0.055 | 0.010 | 3.39E-08 | 0.040 | 0.028 | 0.128 |
|  | rs7787720 | C | T | -0.054 | 0.007 | 2.25E-15 | -0.008 | 0.012 | 0.516 |
|  | rs7901695 | T | C | -0.275 | 0.017 | 8.18E-62 | -0.008 | 0.013 | 0.506 |
|  | rs80196932 | T | C | 0.060 | 0.008 | 7.57E-13 | 0.000 | 0.017 | 0.966 |
|  | rs8026714 | G | A | -0.066 | 0.007 | 1.06E-22 | -0.013 | 0.017 | 0.463 |
|  | rs8043085 | G | T | -0.052 | 0.007 | 2.06E-14 | -0.027 | 0.014 | 0.048 |
|  | rs896852 | G | T | 0.042 | 0.007 | 6.42E-09 | -0.030 | 0.012 | 0.013 |
|  | rs9316706 | A | G | 0.041 | 0.007 | 3.33E-09 | 0.003 | 0.016 | 0.937 |
|  | rs9350271 | G | A | -0.193 | 0.007 | 4.95E-183 | 0.003 | 0.013 | 0.788 |
|  | rs9379084 | G | A | 0.072 | 0.009 | 2.20E-14 | -0.027 | 0.019 | 0.208 |
|  | rs9390022 | T | C | 0.048 | 0.008 | 6.35E-09 | 0.006 | 0.013 | 0.655 |
|  | rs9461650 | G | A | 0.060 | 0.009 | 1.99E-12 | -0.023 | 0.016 | 0.145 |
|  | rs9523295 | G | A | 0.078 | 0.009 | 7.24E-18 | 0.009 | 0.014 | 0.529 |
|  | rs9788635 | C | T | 0.058 | 0.008 | 9.43E-14 | 0.016 | 0.018 | 0.401 |
|  | rs9948462 | C | T | -0.047 | 0.008 | 8.70E-10 | 0.002 | 0.012 | 0.896 |
| *Coriobacteriaceae* | rs10011838 | G | A | 0.073 | 0.007 | 1.43E-27 | -0.006 | 0.012 | 0.614 |
|  | rs1016565 | G | A | -0.038 | 0.007 | 2.18E-08 | 0.015 | 0.011 | 0.192 |
|  | rs10507349 | G | A | 0.064 | 0.007 | 1.69E-21 | 0.007 | 0.012 | 0.589 |
|  | rs10852123 | A | C | 0.060 | 0.008 | 8.38E-13 | -0.009 | 0.012 | 0.504 |
|  | rs10860209 | C | A | 0.040 | 0.007 | 5.67E-09 | 0.004 | 0.015 | 0.689 |
|  | rs10886863 | T | C | -0.060 | 0.007 | 5.28E-17 | 0.006 | 0.034 | 0.876 |
|  | rs10938398 | G | A | -0.046 | 0.007 | 3.84E-10 | -0.008 | 0.011 | 0.433 |
|  | rs10950550 | T | G | 0.065 | 0.007 | 1.75E-19 | 0.003 | 0.011 | 0.779 |
|  | rs10965248 | T | C | 0.183 | 0.007 | 4.42E-164 | 0.000 | 0.014 | 0.974 |
|  | rs111246699 | G | A | -0.061 | 0.008 | 1.54E-15 | -0.001 | 0.013 | 0.935 |
|  | rs113154802 | C | T | 0.060 | 0.011 | 3.51E-08 | 0.008 | 0.018 | 0.702 |
|  | rs1182444 | A | G | -0.047 | 0.007 | 1.67E-12 | 0.014 | 0.011 | 0.198 |
|  | rs11926494 | G | A | 0.112 | 0.009 | 2.69E-37 | 0.023 | 0.018 | 0.210 |
|  | rs1206684 | G | A | 0.040 | 0.007 | 4.42E-09 | -0.002 | 0.011 | 0.864 |
|  | rs123378 | G | A | 0.053 | 0.008 | 2.22E-10 | 0.019 | 0.012 | 0.107 |
|  | rs1260326 | T | C | -0.063 | 0.007 | 1.01E-21 | 0.001 | 0.011 | 0.982 |
|  | rs12610052 | T | C | -0.046 | 0.008 | 1.97E-09 | -0.099 | 0.040 | 0.007 |
|  | rs12625671 | T | C | -0.066 | 0.007 | 2.25E-21 | 0.007 | 0.016 | 0.520 |
|  | rs1266488 | T | C | 0.044 | 0.007 | 6.29E-10 | 0.081 | 0.035 | 0.025 |
|  | rs12698877 | A | G | -0.067 | 0.007 | 6.96E-22 | 0.004 | 0.012 | 0.739 |
|  | rs12818766 | G | A | -0.054 | 0.009 | 2.46E-10 | 0.018 | 0.014 | 0.209 |
|  | rs13092876 | G | A | -0.126 | 0.007 | 1.91E-66 | 0.007 | 0.011 | 0.541 |
|  | rs13266634 | C | T | 0.116 | 0.007 | 3.72E-67 | -0.010 | 0.012 | 0.411 |
|  | rs1328412 | T | C | 0.097 | 0.015 | 6.41E-11 | -0.051 | 0.021 | 0.019 |
|  | rs1421085 | T | C | -0.130 | 0.009 | 1.55E-48 | -0.019 | 0.011 | 0.072 |
|  | rs1426371 | G | A | 0.048 | 0.007 | 7.76E-12 | -0.008 | 0.012 | 0.483 |
|  | rs1459513 | A | C | -0.046 | 0.007 | 3.73E-11 | -0.005 | 0.023 | 0.923 |
|  | rs147707338 | C | T | -0.087 | 0.014 | 6.47E-10 | 0.032 | 0.018 | 0.060 |
|  | rs1513275 | T | C | -0.057 | 0.009 | 2.76E-11 | 0.020 | 0.013 | 0.128 |
|  | rs16884229 | T | C | -0.097 | 0.007 | 1.15E-43 | -0.006 | 0.013 | 0.587 |
|  | rs17168486 | C | T | -0.064 | 0.007 | 8.23E-22 | -0.002 | 0.014 | 0.974 |
|  | rs1850421 | A | C | 0.044 | 0.007 | 1.41E-09 | 0.003 | 0.012 | 0.741 |
|  | rs2074120 | A | C | 0.041 | 0.007 | 8.38E-09 | -0.003 | 0.011 | 0.791 |
|  | rs2092518 | G | T | 0.046 | 0.007 | 1.39E-10 | -0.012 | 0.011 | 0.262 |
|  | rs2126736 | A | G | 0.038 | 0.007 | 1.84E-08 | 0.003 | 0.011 | 0.786 |
|  | rs2240885 | G | A | -0.042 | 0.007 | 2.79E-09 | -0.010 | 0.013 | 0.365 |
|  | rs2269245 | G | A | 0.054 | 0.009 | 5.40E-10 | 0.010 | 0.013 | 0.479 |
|  | rs2327777 | T | C | 0.050 | 0.007 | 1.06E-13 | 0.013 | 0.011 | 0.214 |
|  | rs2583934 | G | T | -0.058 | 0.007 | 4.95E-16 | 0.007 | 0.015 | 0.721 |
|  | rs261982 | C | T | -0.040 | 0.007 | 3.12E-09 | 0.018 | 0.011 | 0.096 |
|  | rs2706710 | C | T | -0.071 | 0.013 | 1.67E-08 | -0.019 | 0.014 | 0.209 |
|  | rs2796441 | G | A | 0.075 | 0.007 | 1.43E-28 | -0.007 | 0.011 | 0.502 |
|  | rs28599782 | G | A | -0.067 | 0.008 | 4.64E-16 | -0.013 | 0.030 | 0.702 |
|  | rs28691713 | C | T | 0.066 | 0.008 | 1.79E-17 | -0.004 | 0.011 | 0.694 |
|  | rs2908279 | T | G | -0.046 | 0.007 | 8.42E-11 | -0.012 | 0.011 | 0.253 |
|  | rs2980883 | T | G | 0.042 | 0.007 | 3.93E-09 | 0.007 | 0.015 | 0.579 |
|  | rs3094508 | T | C | -0.059 | 0.008 | 1.31E-13 | -0.003 | 0.011 | 0.764 |
|  | rs329122 | G | A | -0.039 | 0.007 | 2.22E-08 | -0.009 | 0.011 | 0.408 |
|  | rs34811727 | G | T | -0.076 | 0.013 | 3.32E-09 | 0.013 | 0.022 | 0.538 |
|  | rs349359 | A | C | -0.043 | 0.008 | 3.05E-08 | 0.024 | 0.016 | 0.204 |
|  | rs35589574 | C | T | -0.171 | 0.011 | 1.58E-55 | -0.007 | 0.012 | 0.656 |
|  | rs3735641 | A | G | -0.069 | 0.008 | 3.62E-19 | -0.021 | 0.011 | 0.058 |
|  | rs3751236 | G | A | 0.067 | 0.007 | 6.58E-21 | -0.010 | 0.013 | 0.483 |
|  | rs3852529 | C | T | -0.099 | 0.010 | 2.07E-24 | -0.013 | 0.019 | 0.479 |
|  | rs3887925 | C | T | -0.040 | 0.007 | 3.12E-09 | -0.013 | 0.011 | 0.234 |
|  | rs391933 | G | A | 0.037 | 0.007 | 1.46E-08 | 0.001 | 0.011 | 0.915 |
|  | rs4273712 | A | G | -0.047 | 0.007 | 2.56E-12 | -0.011 | 0.012 | 0.391 |
|  | rs4499362 | C | T | 0.044 | 0.007 | 1.53E-09 | -0.034 | 0.013 | 0.011 |
|  | rs476828 | T | C | -0.084 | 0.008 | 4.81E-27 | 0.001 | 0.012 | 0.916 |
|  | rs4930974 | C | T | -0.069 | 0.008 | 4.07E-19 | 0.019 | 0.013 | 0.145 |
|  | rs532504 | G | A | -0.055 | 0.008 | 7.39E-12 | -0.010 | 0.013 | 0.436 |
|  | rs55700915 | G | A | -0.040 | 0.007 | 1.50E-08 | 0.013 | 0.013 | 0.324 |
|  | rs58524310 | A | G | -0.047 | 0.007 | 8.41E-11 | 0.020 | 0.015 | 0.205 |
|  | rs58718028 | C | T | -0.073 | 0.007 | 3.35E-25 | -0.012 | 0.012 | 0.317 |
|  | rs6012878 | G | A | 0.041 | 0.007 | 4.32E-09 | 0.010 | 0.011 | 0.372 |
|  | rs60573766 | C | T | 0.044 | 0.007 | 4.30E-10 | 0.024 | 0.015 | 0.134 |
|  | rs61779313 | T | C | -0.060 | 0.009 | 5.59E-11 | -0.012 | 0.013 | 0.351 |
|  | rs61975988 | A | G | 0.040 | 0.007 | 1.97E-09 | 0.005 | 0.012 | 0.681 |
|  | rs62173901 | A | G | 0.123 | 0.020 | 6.58E-10 | -0.011 | 0.016 | 0.380 |
|  | rs62405419 | G | T | -0.044 | 0.008 | 3.79E-09 | -0.010 | 0.016 | 0.636 |
|  | rs633862 | C | T | -0.039 | 0.007 | 1.26E-08 | 0.011 | 0.011 | 0.315 |
|  | rs6416749 | C | T | 0.052 | 0.008 | 3.40E-12 | -0.016 | 0.011 | 0.161 |
|  | rs6731688 | A | C | -0.098 | 0.012 | 2.55E-17 | -0.004 | 0.014 | 0.918 |
|  | rs703980 | G | A | 0.060 | 0.007 | 6.55E-19 | -0.006 | 0.011 | 0.560 |
|  | rs7107784 | G | A | 0.086 | 0.012 | 2.60E-13 | -0.011 | 0.012 | 0.385 |
|  | rs7109575 | G | A | 0.141 | 0.015 | 5.46E-21 | 0.014 | 0.015 | 0.342 |
|  | rs7250869 | T | C | 0.056 | 0.007 | 2.29E-16 | 0.011 | 0.011 | 0.332 |
|  | rs7304270 | C | T | 0.068 | 0.010 | 1.04E-12 | 0.027 | 0.058 | 0.579 |
|  | rs73085586 | G | A | 0.043 | 0.007 | 1.66E-09 | -0.011 | 0.013 | 0.409 |
|  | rs7313668 | G | T | -0.045 | 0.007 | 4.91E-11 | 0.008 | 0.011 | 0.470 |
|  | rs73347525 | A | G | 0.059 | 0.009 | 7.46E-11 | -0.014 | 0.014 | 0.335 |
|  | rs742762 | A | C | 0.075 | 0.008 | 1.79E-22 | -0.007 | 0.017 | 0.806 |
|  | rs7501939 | T | C | 0.120 | 0.007 | 1.60E-60 | 0.000 | 0.011 | 0.986 |
|  | rs75990271 | T | C | 0.066 | 0.010 | 3.22E-11 | -0.018 | 0.024 | 0.416 |
|  | rs7656416 | C | T | 0.100 | 0.007 | 9.01E-42 | -0.009 | 0.032 | 0.898 |
|  | rs76704029 | T | C | 0.055 | 0.010 | 3.39E-08 | 0.005 | 0.025 | 0.910 |
|  | rs76878791 | A | G | -0.053 | 0.007 | 2.02E-13 | 0.049 | 0.049 | 0.367 |
|  | rs77789961 | C | T | -0.047 | 0.008 | 1.92E-08 | -0.046 | 0.040 | 0.341 |
|  | rs7787720 | C | T | -0.054 | 0.007 | 2.25E-15 | -0.030 | 0.011 | 0.005 |
|  | rs7901695 | T | C | -0.275 | 0.017 | 8.18E-62 | -0.012 | 0.012 | 0.317 |
|  | rs80196932 | T | C | 0.060 | 0.008 | 7.57E-13 | -0.031 | 0.015 | 0.043 |
|  | rs8026714 | G | A | -0.066 | 0.007 | 1.06E-22 | -0.011 | 0.015 | 0.482 |
|  | rs8043085 | G | T | -0.052 | 0.007 | 2.06E-14 | 0.012 | 0.012 | 0.345 |
|  | rs896852 | G | T | 0.042 | 0.007 | 6.42E-09 | 0.002 | 0.011 | 0.914 |
|  | rs9316706 | A | G | 0.041 | 0.007 | 3.33E-09 | -0.034 | 0.014 | 0.015 |
|  | rs9350271 | G | A | -0.193 | 0.007 | 4.95E-183 | 0.009 | 0.011 | 0.436 |
|  | rs9379084 | G | A | 0.072 | 0.009 | 2.20E-14 | -0.009 | 0.017 | 0.564 |
|  | rs9390022 | T | C | 0.048 | 0.008 | 6.35E-09 | -0.003 | 0.011 | 0.805 |
|  | rs9461650 | G | A | 0.060 | 0.009 | 1.99E-12 | 0.001 | 0.015 | 0.899 |
|  | rs9523295 | G | A | 0.078 | 0.009 | 7.24E-18 | -0.012 | 0.012 | 0.372 |
|  | rs9788635 | C | T | 0.058 | 0.008 | 9.43E-14 | 0.029 | 0.016 | 0.066 |
|  | rs9948462 | C | T | -0.047 | 0.008 | 8.70E-10 | -0.002 | 0.011 | 0.906 |
| *Defluviitaleaceae* | rs10011838 | G | A | 0.073 | 0.007 | 1.43E-27 | 0.010 | 0.017 | 0.535 |
|  | rs1016565 | G | A | -0.038 | 0.007 | 2.18E-08 | 0.021 | 0.017 | 0.223 |
|  | rs10507349 | G | A | 0.064 | 0.007 | 1.69E-21 | 0.001 | 0.018 | 0.980 |
|  | rs10852123 | A | C | 0.060 | 0.008 | 8.38E-13 | -0.012 | 0.018 | 0.499 |
|  | rs10860209 | C | A | 0.040 | 0.007 | 5.67E-09 | 0.015 | 0.022 | 0.513 |
|  | rs10938398 | G | A | -0.046 | 0.007 | 3.84E-10 | 0.003 | 0.016 | 0.858 |
|  | rs10950550 | T | G | 0.065 | 0.007 | 1.75E-19 | -0.009 | 0.016 | 0.582 |
|  | rs10965248 | T | C | 0.183 | 0.007 | 4.42E-164 | -0.037 | 0.020 | 0.066 |
|  | rs111246699 | G | A | -0.061 | 0.008 | 1.54E-15 | 0.015 | 0.019 | 0.492 |
|  | rs113154802 | C | T | 0.060 | 0.011 | 3.51E-08 | 0.012 | 0.026 | 0.633 |
|  | rs1182444 | A | G | -0.047 | 0.007 | 1.67E-12 | -0.004 | 0.016 | 0.825 |
|  | rs11926494 | G | A | 0.112 | 0.009 | 2.69E-37 | 0.002 | 0.027 | 0.957 |
|  | rs1206684 | G | A | 0.040 | 0.007 | 4.42E-09 | 0.006 | 0.016 | 0.680 |
|  | rs123378 | G | A | 0.053 | 0.008 | 2.22E-10 | -0.001 | 0.017 | 0.895 |
|  | rs1260326 | T | C | -0.063 | 0.007 | 1.01E-21 | 0.004 | 0.016 | 0.823 |
|  | rs12625671 | T | C | -0.066 | 0.007 | 2.25E-21 | 0.029 | 0.024 | 0.237 |
|  | rs12698877 | A | G | -0.067 | 0.007 | 6.96E-22 | -0.003 | 0.018 | 0.878 |
|  | rs12818766 | G | A | -0.054 | 0.009 | 2.46E-10 | 0.019 | 0.021 | 0.374 |
|  | rs13092876 | G | A | -0.126 | 0.007 | 1.91E-66 | 0.024 | 0.017 | 0.156 |
|  | rs13266634 | C | T | 0.116 | 0.007 | 3.72E-67 | 0.008 | 0.017 | 0.594 |
|  | rs1328412 | T | C | 0.097 | 0.015 | 6.41E-11 | 0.019 | 0.031 | 0.549 |
|  | rs1421085 | T | C | -0.130 | 0.009 | 1.55E-48 | -0.002 | 0.016 | 0.860 |
|  | rs1426371 | G | A | 0.048 | 0.007 | 7.76E-12 | 0.002 | 0.018 | 0.908 |
|  | rs1459513 | A | C | -0.046 | 0.007 | 3.73E-11 | 0.064 | 0.036 | 0.055 |
|  | rs147707338 | C | T | -0.087 | 0.014 | 6.47E-10 | 0.057 | 0.026 | 0.022 |
|  | rs1513275 | T | C | -0.057 | 0.009 | 2.76E-11 | 0.009 | 0.019 | 0.599 |
|  | rs16884229 | T | C | -0.097 | 0.007 | 1.15E-43 | 0.035 | 0.020 | 0.073 |
|  | rs17168486 | C | T | -0.064 | 0.007 | 8.23E-22 | -0.023 | 0.021 | 0.289 |
|  | rs1850421 | A | C | 0.044 | 0.007 | 1.41E-09 | 0.008 | 0.017 | 0.665 |
|  | rs2074120 | A | C | 0.041 | 0.007 | 8.38E-09 | 0.014 | 0.016 | 0.363 |
|  | rs2092518 | G | T | 0.046 | 0.007 | 1.39E-10 | -0.021 | 0.016 | 0.175 |
|  | rs2126736 | A | G | 0.038 | 0.007 | 1.84E-08 | -0.008 | 0.016 | 0.609 |
|  | rs2240885 | G | A | -0.042 | 0.007 | 2.79E-09 | -0.014 | 0.019 | 0.450 |
|  | rs2269245 | G | A | 0.054 | 0.009 | 5.40E-10 | 0.009 | 0.020 | 0.590 |
|  | rs2327777 | T | C | 0.050 | 0.007 | 1.06E-13 | 0.011 | 0.016 | 0.467 |
|  | rs2583934 | G | T | -0.058 | 0.007 | 4.95E-16 | -0.009 | 0.022 | 0.816 |
|  | rs261982 | C | T | -0.040 | 0.007 | 3.12E-09 | -0.027 | 0.016 | 0.097 |
|  | rs2706710 | C | T | -0.071 | 0.013 | 1.67E-08 | -0.023 | 0.020 | 0.255 |
|  | rs2796441 | G | A | 0.075 | 0.007 | 1.43E-28 | -0.004 | 0.016 | 0.790 |
|  | rs28691713 | C | T | 0.066 | 0.008 | 1.79E-17 | 0.014 | 0.016 | 0.376 |
|  | rs2908279 | T | G | -0.046 | 0.007 | 8.42E-11 | -0.029 | 0.016 | 0.061 |
|  | rs2980883 | T | G | 0.042 | 0.007 | 3.93E-09 | -0.012 | 0.022 | 0.572 |
|  | rs3094508 | T | C | -0.059 | 0.008 | 1.31E-13 | 0.025 | 0.016 | 0.121 |
|  | rs329122 | G | A | -0.039 | 0.007 | 2.22E-08 | -0.017 | 0.016 | 0.269 |
|  | rs34811727 | G | T | -0.076 | 0.013 | 3.32E-09 | 0.023 | 0.031 | 0.430 |
|  | rs349359 | A | C | -0.043 | 0.008 | 3.05E-08 | -0.005 | 0.024 | 0.807 |
|  | rs35589574 | C | T | -0.171 | 0.011 | 1.58E-55 | -0.004 | 0.018 | 0.798 |
|  | rs3735641 | A | G | -0.069 | 0.008 | 3.62E-19 | 0.002 | 0.016 | 0.897 |
|  | rs3751236 | G | A | 0.067 | 0.007 | 6.58E-21 | -0.009 | 0.019 | 0.684 |
|  | rs3852529 | C | T | -0.099 | 0.010 | 2.07E-24 | -0.014 | 0.028 | 0.653 |
|  | rs3887925 | C | T | -0.040 | 0.007 | 3.12E-09 | -0.006 | 0.016 | 0.733 |
|  | rs391933 | G | A | 0.037 | 0.007 | 1.46E-08 | -0.008 | 0.016 | 0.598 |
|  | rs4273712 | A | G | -0.047 | 0.007 | 2.56E-12 | -0.029 | 0.018 | 0.093 |
|  | rs4499362 | C | T | 0.044 | 0.007 | 1.53E-09 | -0.005 | 0.020 | 0.801 |
|  | rs476828 | T | C | -0.084 | 0.008 | 4.81E-27 | -0.004 | 0.018 | 0.804 |
|  | rs4930974 | C | T | -0.069 | 0.008 | 4.07E-19 | -0.011 | 0.019 | 0.570 |
|  | rs532504 | G | A | -0.055 | 0.008 | 7.39E-12 | -0.040 | 0.020 | 0.039 |
|  | rs55700915 | G | A | -0.040 | 0.007 | 1.50E-08 | 0.010 | 0.020 | 0.645 |
|  | rs58524310 | A | G | -0.047 | 0.007 | 8.41E-11 | 0.010 | 0.022 | 0.624 |
|  | rs58718028 | C | T | -0.073 | 0.007 | 3.35E-25 | -0.039 | 0.018 | 0.032 |
|  | rs6012878 | G | A | 0.041 | 0.007 | 4.32E-09 | -0.036 | 0.016 | 0.023 |
|  | rs60573766 | C | T | 0.044 | 0.007 | 4.30E-10 | 0.015 | 0.023 | 0.593 |
|  | rs61779313 | T | C | -0.060 | 0.009 | 5.59E-11 | 0.001 | 0.018 | 0.944 |
|  | rs61975988 | A | G | 0.040 | 0.007 | 1.97E-09 | 0.001 | 0.018 | 0.931 |
|  | rs62173901 | A | G | 0.123 | 0.020 | 6.58E-10 | -0.005 | 0.022 | 0.778 |
|  | rs62405419 | G | T | -0.044 | 0.008 | 3.79E-09 | 0.007 | 0.025 | 0.837 |
|  | rs633862 | C | T | -0.039 | 0.007 | 1.26E-08 | -0.009 | 0.016 | 0.586 |
|  | rs6416749 | C | T | 0.052 | 0.008 | 3.40E-12 | -0.012 | 0.017 | 0.486 |
|  | rs6731688 | A | C | -0.098 | 0.012 | 2.55E-17 | -0.009 | 0.021 | 0.666 |
|  | rs703980 | G | A | 0.060 | 0.007 | 6.55E-19 | 0.007 | 0.016 | 0.640 |
|  | rs7107784 | G | A | 0.086 | 0.012 | 2.60E-13 | 0.019 | 0.017 | 0.240 |
|  | rs7109575 | G | A | 0.141 | 0.015 | 5.46E-21 | 0.016 | 0.021 | 0.438 |
|  | rs7250869 | T | C | 0.056 | 0.007 | 2.29E-16 | 0.022 | 0.017 | 0.198 |
|  | rs73085586 | G | A | 0.043 | 0.007 | 1.66E-09 | 0.014 | 0.020 | 0.457 |
|  | rs7313668 | G | T | -0.045 | 0.007 | 4.91E-11 | 0.017 | 0.016 | 0.256 |
|  | rs73347525 | A | G | 0.059 | 0.009 | 7.46E-11 | 0.019 | 0.020 | 0.377 |
|  | rs742762 | A | C | 0.075 | 0.008 | 1.79E-22 | 0.007 | 0.025 | 0.843 |
|  | rs7501939 | T | C | 0.120 | 0.007 | 1.60E-60 | -0.009 | 0.016 | 0.635 |
|  | rs75990271 | T | C | 0.066 | 0.010 | 3.22E-11 | 0.080 | 0.034 | 0.012 |
|  | rs76704029 | T | C | 0.055 | 0.010 | 3.39E-08 | -0.050 | 0.039 | 0.248 |
|  | rs7787720 | C | T | -0.054 | 0.007 | 2.25E-15 | -0.016 | 0.016 | 0.314 |
|  | rs7901695 | T | C | -0.275 | 0.017 | 8.18E-62 | -0.014 | 0.017 | 0.431 |
|  | rs80196932 | T | C | 0.060 | 0.008 | 7.57E-13 | -0.009 | 0.022 | 0.720 |
|  | rs8026714 | G | A | -0.066 | 0.007 | 1.06E-22 | -0.009 | 0.023 | 0.694 |
|  | rs8043085 | G | T | -0.052 | 0.007 | 2.06E-14 | -0.002 | 0.018 | 0.865 |
|  | rs896852 | G | T | 0.042 | 0.007 | 6.42E-09 | 0.007 | 0.016 | 0.659 |
|  | rs9316706 | A | G | 0.041 | 0.007 | 3.33E-09 | -0.038 | 0.021 | 0.063 |
|  | rs9350271 | G | A | -0.193 | 0.007 | 4.95E-183 | -0.009 | 0.017 | 0.594 |
|  | rs9379084 | G | A | 0.072 | 0.009 | 2.20E-14 | -0.037 | 0.025 | 0.154 |
|  | rs9390022 | T | C | 0.048 | 0.008 | 6.35E-09 | 0.017 | 0.016 | 0.302 |
|  | rs9461650 | G | A | 0.060 | 0.009 | 1.99E-12 | -0.027 | 0.022 | 0.200 |
|  | rs9523295 | G | A | 0.078 | 0.009 | 7.24E-18 | 0.009 | 0.018 | 0.592 |
|  | rs9788635 | C | T | 0.058 | 0.008 | 9.43E-14 | 0.001 | 0.024 | 0.982 |
|  | rs9948462 | C | T | -0.047 | 0.008 | 8.70E-10 | -0.013 | 0.016 | 0.437 |
| *Desulfovibrionaceae* | rs10011838 | G | A | 0.073 | 0.007 | 1.43E-27 | -0.002 | 0.013 | 0.860 |
|  | rs1016565 | G | A | -0.038 | 0.007 | 2.18E-08 | -0.013 | 0.012 | 0.307 |
|  | rs10507349 | G | A | 0.064 | 0.007 | 1.69E-21 | 0.001 | 0.013 | 0.962 |
|  | rs10852123 | A | C | 0.060 | 0.008 | 8.38E-13 | 0.010 | 0.013 | 0.488 |
|  | rs10860209 | C | A | 0.040 | 0.007 | 5.67E-09 | -0.002 | 0.016 | 0.900 |
|  | rs10886863 | T | C | -0.060 | 0.007 | 5.28E-17 | -0.014 | 0.037 | 0.680 |
|  | rs10938398 | G | A | -0.046 | 0.007 | 3.84E-10 | 0.004 | 0.012 | 0.765 |
|  | rs10950550 | T | G | 0.065 | 0.007 | 1.75E-19 | -0.002 | 0.012 | 0.831 |
|  | rs10965248 | T | C | 0.183 | 0.007 | 4.42E-164 | 0.021 | 0.015 | 0.184 |
|  | rs111246699 | G | A | -0.061 | 0.008 | 1.54E-15 | -0.009 | 0.014 | 0.504 |
|  | rs113154802 | C | T | 0.060 | 0.011 | 3.51E-08 | 0.017 | 0.020 | 0.356 |
|  | rs1182444 | A | G | -0.047 | 0.007 | 1.67E-12 | -0.008 | 0.012 | 0.524 |
|  | rs11926494 | G | A | 0.112 | 0.009 | 2.69E-37 | -0.002 | 0.020 | 0.876 |
|  | rs1206684 | G | A | 0.040 | 0.007 | 4.42E-09 | 0.000 | 0.012 | 0.975 |
|  | rs123378 | G | A | 0.053 | 0.008 | 2.22E-10 | -0.024 | 0.012 | 0.052 |
|  | rs1260326 | T | C | -0.063 | 0.007 | 1.01E-21 | -0.004 | 0.012 | 0.730 |
|  | rs12610052 | T | C | -0.046 | 0.008 | 1.97E-09 | 0.046 | 0.044 | 0.337 |
|  | rs12625671 | T | C | -0.066 | 0.007 | 2.25E-21 | -0.003 | 0.017 | 0.951 |
|  | rs1266488 | T | C | 0.044 | 0.007 | 6.29E-10 | 0.051 | 0.037 | 0.234 |
|  | rs12698877 | A | G | -0.067 | 0.007 | 6.96E-22 | -0.018 | 0.013 | 0.154 |
|  | rs12818766 | G | A | -0.054 | 0.009 | 2.46E-10 | 0.017 | 0.015 | 0.277 |
|  | rs13092876 | G | A | -0.126 | 0.007 | 1.91E-66 | -0.001 | 0.012 | 0.912 |
|  | rs13266634 | C | T | 0.116 | 0.007 | 3.72E-67 | -0.003 | 0.012 | 0.852 |
|  | rs1328412 | T | C | 0.097 | 0.015 | 6.41E-11 | 0.007 | 0.023 | 0.756 |
|  | rs1421085 | T | C | -0.130 | 0.009 | 1.55E-48 | -0.013 | 0.012 | 0.317 |
|  | rs1426371 | G | A | 0.048 | 0.007 | 7.76E-12 | 0.014 | 0.013 | 0.241 |
|  | rs1459513 | A | C | -0.046 | 0.007 | 3.73E-11 | -0.032 | 0.025 | 0.177 |
|  | rs147707338 | C | T | -0.087 | 0.014 | 6.47E-10 | 0.005 | 0.020 | 0.793 |
|  | rs1513275 | T | C | -0.057 | 0.009 | 2.76E-11 | -0.012 | 0.014 | 0.405 |
|  | rs16884229 | T | C | -0.097 | 0.007 | 1.15E-43 | -0.012 | 0.014 | 0.392 |
|  | rs17168486 | C | T | -0.064 | 0.007 | 8.23E-22 | -0.009 | 0.015 | 0.549 |
|  | rs1850421 | A | C | 0.044 | 0.007 | 1.41E-09 | -0.026 | 0.013 | 0.040 |
|  | rs2074120 | A | C | 0.041 | 0.007 | 8.38E-09 | 0.004 | 0.012 | 0.723 |
|  | rs2092518 | G | T | 0.046 | 0.007 | 1.39E-10 | 0.005 | 0.012 | 0.701 |
|  | rs2126736 | A | G | 0.038 | 0.007 | 1.84E-08 | 0.002 | 0.012 | 0.904 |
|  | rs2240885 | G | A | -0.042 | 0.007 | 2.79E-09 | 0.032 | 0.014 | 0.022 |
|  | rs2269245 | G | A | 0.054 | 0.009 | 5.40E-10 | 0.002 | 0.014 | 0.892 |
|  | rs2327777 | T | C | 0.050 | 0.007 | 1.06E-13 | 0.009 | 0.012 | 0.445 |
|  | rs2583934 | G | T | -0.058 | 0.007 | 4.95E-16 | -0.022 | 0.016 | 0.136 |
|  | rs261982 | C | T | -0.040 | 0.007 | 3.12E-09 | -0.025 | 0.012 | 0.038 |
|  | rs2706710 | C | T | -0.071 | 0.013 | 1.67E-08 | -0.004 | 0.015 | 0.762 |
|  | rs2796441 | G | A | 0.075 | 0.007 | 1.43E-28 | -0.006 | 0.012 | 0.630 |
|  | rs28599782 | G | A | -0.067 | 0.008 | 4.64E-16 | 0.012 | 0.033 | 0.733 |
|  | rs28691713 | C | T | 0.066 | 0.008 | 1.79E-17 | -0.008 | 0.012 | 0.486 |
|  | rs2908279 | T | G | -0.046 | 0.007 | 8.42E-11 | 0.000 | 0.012 | 0.960 |
|  | rs2980883 | T | G | 0.042 | 0.007 | 3.93E-09 | 0.004 | 0.016 | 0.795 |
|  | rs3094508 | T | C | -0.059 | 0.008 | 1.31E-13 | 0.006 | 0.012 | 0.559 |
|  | rs329122 | G | A | -0.039 | 0.007 | 2.22E-08 | 0.006 | 0.012 | 0.619 |
|  | rs34811727 | G | T | -0.076 | 0.013 | 3.32E-09 | 0.010 | 0.023 | 0.686 |
|  | rs349359 | A | C | -0.043 | 0.008 | 3.05E-08 | 0.015 | 0.018 | 0.474 |
|  | rs35589574 | C | T | -0.171 | 0.011 | 1.58E-55 | -0.028 | 0.014 | 0.052 |
|  | rs3735641 | A | G | -0.069 | 0.008 | 3.62E-19 | 0.013 | 0.012 | 0.256 |
|  | rs3751236 | G | A | 0.067 | 0.007 | 6.58E-21 | -0.015 | 0.014 | 0.331 |
|  | rs3852529 | C | T | -0.099 | 0.010 | 2.07E-24 | -0.019 | 0.020 | 0.246 |
|  | rs3887925 | C | T | -0.040 | 0.007 | 3.12E-09 | 0.002 | 0.012 | 0.861 |
|  | rs391933 | G | A | 0.037 | 0.007 | 1.46E-08 | -0.017 | 0.012 | 0.136 |
|  | rs4273712 | A | G | -0.047 | 0.007 | 2.56E-12 | -0.026 | 0.013 | 0.047 |
|  | rs4499362 | C | T | 0.044 | 0.007 | 1.53E-09 | -0.011 | 0.014 | 0.479 |
|  | rs476828 | T | C | -0.084 | 0.008 | 4.81E-27 | -0.024 | 0.013 | 0.074 |
|  | rs4930974 | C | T | -0.069 | 0.008 | 4.07E-19 | -0.026 | 0.014 | 0.059 |
|  | rs532504 | G | A | -0.055 | 0.008 | 7.39E-12 | 0.001 | 0.014 | 0.926 |
|  | rs55700915 | G | A | -0.040 | 0.007 | 1.50E-08 | -0.009 | 0.014 | 0.538 |
|  | rs58524310 | A | G | -0.047 | 0.007 | 8.41E-11 | -0.024 | 0.016 | 0.130 |
|  | rs58718028 | C | T | -0.073 | 0.007 | 3.35E-25 | -0.005 | 0.013 | 0.700 |
|  | rs6012878 | G | A | 0.041 | 0.007 | 4.32E-09 | 0.008 | 0.012 | 0.484 |
|  | rs60573766 | C | T | 0.044 | 0.007 | 4.30E-10 | -0.023 | 0.017 | 0.187 |
|  | rs61779313 | T | C | -0.060 | 0.009 | 5.59E-11 | 0.013 | 0.014 | 0.316 |
|  | rs61975988 | A | G | 0.040 | 0.007 | 1.97E-09 | -0.014 | 0.013 | 0.291 |
|  | rs62173901 | A | G | 0.123 | 0.020 | 6.58E-10 | 0.000 | 0.017 | 0.951 |
|  | rs62405419 | G | T | -0.044 | 0.008 | 3.79E-09 | -0.027 | 0.018 | 0.092 |
|  | rs633862 | C | T | -0.039 | 0.007 | 1.26E-08 | -0.002 | 0.012 | 0.863 |
|  | rs6416749 | C | T | 0.052 | 0.008 | 3.40E-12 | -0.020 | 0.012 | 0.103 |
|  | rs6731688 | A | C | -0.098 | 0.012 | 2.55E-17 | -0.004 | 0.015 | 0.834 |
|  | rs703980 | G | A | 0.060 | 0.007 | 6.55E-19 | 0.006 | 0.012 | 0.612 |
|  | rs7107784 | G | A | 0.086 | 0.012 | 2.60E-13 | 0.010 | 0.013 | 0.417 |
|  | rs7109575 | G | A | 0.141 | 0.015 | 5.46E-21 | 0.012 | 0.016 | 0.333 |
|  | rs7250869 | T | C | 0.056 | 0.007 | 2.29E-16 | 0.022 | 0.012 | 0.084 |
|  | rs73085586 | G | A | 0.043 | 0.007 | 1.66E-09 | 0.001 | 0.014 | 0.927 |
|  | rs7313668 | G | T | -0.045 | 0.007 | 4.91E-11 | -0.001 | 0.012 | 0.970 |
|  | rs73347525 | A | G | 0.059 | 0.009 | 7.46E-11 | -0.009 | 0.015 | 0.500 |
|  | rs742762 | A | C | 0.075 | 0.008 | 1.79E-22 | 0.009 | 0.018 | 0.484 |
|  | rs7501939 | T | C | 0.120 | 0.007 | 1.60E-60 | 0.004 | 0.012 | 0.722 |
|  | rs75990271 | T | C | 0.066 | 0.010 | 3.22E-11 | 0.024 | 0.026 | 0.413 |
|  | rs7656416 | C | T | 0.100 | 0.007 | 9.01E-42 | 0.028 | 0.034 | 0.526 |
|  | rs76704029 | T | C | 0.055 | 0.010 | 3.39E-08 | -0.007 | 0.026 | 0.776 |
|  | rs7787720 | C | T | -0.054 | 0.007 | 2.25E-15 | 0.009 | 0.012 | 0.447 |
|  | rs7901695 | T | C | -0.275 | 0.017 | 8.18E-62 | -0.002 | 0.013 | 0.886 |
|  | rs80196932 | T | C | 0.060 | 0.008 | 7.57E-13 | -0.011 | 0.016 | 0.486 |
|  | rs8026714 | G | A | -0.066 | 0.007 | 1.06E-22 | -0.029 | 0.017 | 0.091 |
|  | rs8043085 | G | T | -0.052 | 0.007 | 2.06E-14 | 0.009 | 0.013 | 0.496 |
|  | rs896852 | G | T | 0.042 | 0.007 | 6.42E-09 | 0.012 | 0.012 | 0.306 |
|  | rs9316706 | A | G | 0.041 | 0.007 | 3.33E-09 | -0.008 | 0.015 | 0.634 |
|  | rs9350271 | G | A | -0.193 | 0.007 | 4.95E-183 | 0.024 | 0.012 | 0.047 |
|  | rs9379084 | G | A | 0.072 | 0.009 | 2.20E-14 | -0.035 | 0.019 | 0.051 |
|  | rs9390022 | T | C | 0.048 | 0.008 | 6.35E-09 | -0.013 | 0.012 | 0.278 |
|  | rs9461650 | G | A | 0.060 | 0.009 | 1.99E-12 | -0.001 | 0.016 | 0.965 |
|  | rs9523295 | G | A | 0.078 | 0.009 | 7.24E-18 | -0.019 | 0.013 | 0.138 |
|  | rs9788635 | C | T | 0.058 | 0.008 | 9.43E-14 | -0.004 | 0.018 | 0.741 |
|  | rs9948462 | C | T | -0.047 | 0.008 | 8.70E-10 | 0.005 | 0.012 | 0.674 |
| *Enterobacteriaceae* | rs10011838 | G | A | 0.073 | 0.007 | 1.43E-27 | 0.013 | 0.013 | 0.348 |
|  | rs1016565 | G | A | -0.038 | 0.007 | 2.18E-08 | -0.002 | 0.013 | 0.911 |
|  | rs10507349 | G | A | 0.064 | 0.007 | 1.69E-21 | -0.001 | 0.014 | 0.877 |
|  | rs10852123 | A | C | 0.060 | 0.008 | 8.38E-13 | -0.018 | 0.014 | 0.168 |
|  | rs10860209 | C | A | 0.040 | 0.007 | 5.67E-09 | 0.018 | 0.017 | 0.312 |
|  | rs10886863 | T | C | -0.060 | 0.007 | 5.28E-17 | 0.012 | 0.037 | 0.480 |
|  | rs10938398 | G | A | -0.046 | 0.007 | 3.84E-10 | -0.014 | 0.012 | 0.260 |
|  | rs10950550 | T | G | 0.065 | 0.007 | 1.75E-19 | -0.001 | 0.012 | 0.919 |
|  | rs10965248 | T | C | 0.183 | 0.007 | 4.42E-164 | -0.003 | 0.016 | 0.821 |
|  | rs111246699 | G | A | -0.061 | 0.008 | 1.54E-15 | 0.025 | 0.014 | 0.086 |
|  | rs113154802 | C | T | 0.060 | 0.011 | 3.51E-08 | -0.036 | 0.021 | 0.082 |
|  | rs1182444 | A | G | -0.047 | 0.007 | 1.67E-12 | -0.011 | 0.013 | 0.416 |
|  | rs11926494 | G | A | 0.112 | 0.009 | 2.69E-37 | 0.018 | 0.021 | 0.468 |
|  | rs1206684 | G | A | 0.040 | 0.007 | 4.42E-09 | -0.007 | 0.013 | 0.590 |
|  | rs123378 | G | A | 0.053 | 0.008 | 2.22E-10 | -0.033 | 0.013 | 0.016 |
|  | rs1260326 | T | C | -0.063 | 0.007 | 1.01E-21 | -0.008 | 0.012 | 0.503 |
|  | rs12610052 | T | C | -0.046 | 0.008 | 1.97E-09 | 0.074 | 0.041 | 0.121 |
|  | rs12625671 | T | C | -0.066 | 0.007 | 2.25E-21 | 0.007 | 0.018 | 0.766 |
|  | rs1266488 | T | C | 0.044 | 0.007 | 6.29E-10 | 0.007 | 0.039 | 0.913 |
|  | rs12698877 | A | G | -0.067 | 0.007 | 6.96E-22 | 0.011 | 0.014 | 0.378 |
|  | rs12818766 | G | A | -0.054 | 0.009 | 2.46E-10 | 0.020 | 0.016 | 0.220 |
|  | rs13092876 | G | A | -0.126 | 0.007 | 1.91E-66 | -0.009 | 0.013 | 0.472 |
|  | rs13266634 | C | T | 0.116 | 0.007 | 3.72E-67 | -0.016 | 0.013 | 0.237 |
|  | rs1328412 | T | C | 0.097 | 0.015 | 6.41E-11 | 0.033 | 0.024 | 0.183 |
|  | rs1421085 | T | C | -0.130 | 0.009 | 1.55E-48 | -0.003 | 0.012 | 0.755 |
|  | rs1426371 | G | A | 0.048 | 0.007 | 7.76E-12 | -0.014 | 0.014 | 0.327 |
|  | rs1459513 | A | C | -0.046 | 0.007 | 3.73E-11 | -0.021 | 0.027 | 0.363 |
|  | rs147707338 | C | T | -0.087 | 0.014 | 6.47E-10 | -0.020 | 0.021 | 0.284 |
|  | rs1513275 | T | C | -0.057 | 0.009 | 2.76E-11 | 0.007 | 0.014 | 0.500 |
|  | rs16884229 | T | C | -0.097 | 0.007 | 1.15E-43 | -0.002 | 0.015 | 0.855 |
|  | rs17168486 | C | T | -0.064 | 0.007 | 8.23E-22 | 0.018 | 0.016 | 0.230 |
|  | rs1850421 | A | C | 0.044 | 0.007 | 1.41E-09 | 0.013 | 0.013 | 0.360 |
|  | rs2074120 | A | C | 0.041 | 0.007 | 8.38E-09 | 0.023 | 0.012 | 0.056 |
|  | rs2092518 | G | T | 0.046 | 0.007 | 1.39E-10 | 0.001 | 0.012 | 0.922 |
|  | rs2126736 | A | G | 0.038 | 0.007 | 1.84E-08 | -0.001 | 0.012 | 0.925 |
|  | rs2240885 | G | A | -0.042 | 0.007 | 2.79E-09 | -0.045 | 0.015 | 0.002 |
|  | rs2269245 | G | A | 0.054 | 0.009 | 5.40E-10 | 0.006 | 0.015 | 0.655 |
|  | rs2327777 | T | C | 0.050 | 0.007 | 1.06E-13 | -0.015 | 0.012 | 0.206 |
|  | rs2583934 | G | T | -0.058 | 0.007 | 4.95E-16 | -0.020 | 0.017 | 0.264 |
|  | rs261982 | C | T | -0.040 | 0.007 | 3.12E-09 | 0.027 | 0.012 | 0.028 |
|  | rs2706710 | C | T | -0.071 | 0.013 | 1.67E-08 | 0.005 | 0.016 | 0.751 |
|  | rs2796441 | G | A | 0.075 | 0.007 | 1.43E-28 | 0.007 | 0.012 | 0.584 |
|  | rs28599782 | G | A | -0.067 | 0.008 | 4.64E-16 | -0.046 | 0.034 | 0.119 |
|  | rs28691713 | C | T | 0.066 | 0.008 | 1.79E-17 | 0.003 | 0.012 | 0.790 |
|  | rs2908279 | T | G | -0.046 | 0.007 | 8.42E-11 | -0.002 | 0.012 | 0.882 |
|  | rs2980883 | T | G | 0.042 | 0.007 | 3.93E-09 | -0.002 | 0.017 | 0.906 |
|  | rs3094508 | T | C | -0.059 | 0.008 | 1.31E-13 | 0.013 | 0.013 | 0.335 |
|  | rs329122 | G | A | -0.039 | 0.007 | 2.22E-08 | 0.016 | 0.012 | 0.211 |
|  | rs34811727 | G | T | -0.076 | 0.013 | 3.32E-09 | -0.025 | 0.025 | 0.288 |
|  | rs349359 | A | C | -0.043 | 0.008 | 3.05E-08 | 0.021 | 0.019 | 0.206 |
|  | rs35589574 | C | T | -0.171 | 0.011 | 1.58E-55 | 0.011 | 0.014 | 0.426 |
|  | rs3735641 | A | G | -0.069 | 0.008 | 3.62E-19 | -0.014 | 0.013 | 0.282 |
|  | rs3751236 | G | A | 0.067 | 0.007 | 6.58E-21 | -0.022 | 0.015 | 0.135 |
|  | rs3852529 | C | T | -0.099 | 0.010 | 2.07E-24 | -0.027 | 0.021 | 0.219 |
|  | rs3887925 | C | T | -0.040 | 0.007 | 3.12E-09 | 0.003 | 0.012 | 0.842 |
|  | rs391933 | G | A | 0.037 | 0.007 | 1.46E-08 | -0.002 | 0.012 | 0.832 |
|  | rs4273712 | A | G | -0.047 | 0.007 | 2.56E-12 | 0.003 | 0.014 | 0.812 |
|  | rs4499362 | C | T | 0.044 | 0.007 | 1.53E-09 | -0.012 | 0.015 | 0.423 |
|  | rs476828 | T | C | -0.084 | 0.008 | 4.81E-27 | 0.001 | 0.014 | 0.912 |
|  | rs4930974 | C | T | -0.069 | 0.008 | 4.07E-19 | 0.022 | 0.015 | 0.137 |
|  | rs532504 | G | A | -0.055 | 0.008 | 7.39E-12 | 0.002 | 0.015 | 0.886 |
|  | rs55700915 | G | A | -0.040 | 0.007 | 1.50E-08 | 0.024 | 0.015 | 0.106 |
|  | rs58524310 | A | G | -0.047 | 0.007 | 8.41E-11 | -0.012 | 0.017 | 0.526 |
|  | rs58718028 | C | T | -0.073 | 0.007 | 3.35E-25 | -0.010 | 0.014 | 0.553 |
|  | rs6012878 | G | A | 0.041 | 0.007 | 4.32E-09 | 0.007 | 0.012 | 0.578 |
|  | rs60573766 | C | T | 0.044 | 0.007 | 4.30E-10 | -0.021 | 0.017 | 0.238 |
|  | rs61779313 | T | C | -0.060 | 0.009 | 5.59E-11 | -0.040 | 0.014 | 0.007 |
|  | rs61975988 | A | G | 0.040 | 0.007 | 1.97E-09 | 0.002 | 0.014 | 0.885 |
|  | rs62173901 | A | G | 0.123 | 0.020 | 6.58E-10 | 0.001 | 0.018 | 0.985 |
|  | rs62405419 | G | T | -0.044 | 0.008 | 3.79E-09 | 0.005 | 0.018 | 0.931 |
|  | rs633862 | C | T | -0.039 | 0.007 | 1.26E-08 | -0.008 | 0.012 | 0.499 |
|  | rs6416749 | C | T | 0.052 | 0.008 | 3.40E-12 | 0.000 | 0.013 | 0.972 |
|  | rs6731688 | A | C | -0.098 | 0.012 | 2.55E-17 | -0.023 | 0.016 | 0.181 |
|  | rs703980 | G | A | 0.060 | 0.007 | 6.55E-19 | 0.016 | 0.012 | 0.195 |
|  | rs7107784 | G | A | 0.086 | 0.012 | 2.60E-13 | 0.002 | 0.014 | 0.839 |
|  | rs7109575 | G | A | 0.141 | 0.015 | 5.46E-21 | 0.007 | 0.017 | 0.724 |
|  | rs7250869 | T | C | 0.056 | 0.007 | 2.29E-16 | 0.004 | 0.013 | 0.743 |
|  | rs73085586 | G | A | 0.043 | 0.007 | 1.66E-09 | 0.009 | 0.015 | 0.603 |
|  | rs7313668 | G | T | -0.045 | 0.007 | 4.91E-11 | 0.035 | 0.012 | 0.005 |
|  | rs73347525 | A | G | 0.059 | 0.009 | 7.46E-11 | -0.006 | 0.016 | 0.730 |
|  | rs742762 | A | C | 0.075 | 0.008 | 1.79E-22 | 0.026 | 0.019 | 0.196 |
|  | rs7501939 | T | C | 0.120 | 0.007 | 1.60E-60 | -0.002 | 0.013 | 0.817 |
|  | rs75990271 | T | C | 0.066 | 0.010 | 3.22E-11 | -0.009 | 0.027 | 0.827 |
|  | rs7656416 | C | T | 0.100 | 0.007 | 9.01E-42 | 0.014 | 0.035 | 0.664 |
|  | rs76704029 | T | C | 0.055 | 0.010 | 3.39E-08 | 0.065 | 0.028 | 0.036 |
|  | rs7787720 | C | T | -0.054 | 0.007 | 2.25E-15 | 0.002 | 0.012 | 0.890 |
|  | rs7901695 | T | C | -0.275 | 0.017 | 8.18E-62 | -0.018 | 0.013 | 0.184 |
|  | rs80196932 | T | C | 0.060 | 0.008 | 7.57E-13 | 0.015 | 0.017 | 0.388 |
|  | rs8026714 | G | A | -0.066 | 0.007 | 1.06E-22 | -0.008 | 0.017 | 0.744 |
|  | rs8043085 | G | T | -0.052 | 0.007 | 2.06E-14 | -0.017 | 0.014 | 0.245 |
|  | rs896852 | G | T | 0.042 | 0.007 | 6.42E-09 | 0.000 | 0.012 | 0.999 |
|  | rs9316706 | A | G | 0.041 | 0.007 | 3.33E-09 | 0.008 | 0.016 | 0.572 |
|  | rs9350271 | G | A | -0.193 | 0.007 | 4.95E-183 | 0.020 | 0.013 | 0.116 |
|  | rs9379084 | G | A | 0.072 | 0.009 | 2.20E-14 | -0.031 | 0.020 | 0.109 |
|  | rs9390022 | T | C | 0.048 | 0.008 | 6.35E-09 | -0.004 | 0.013 | 0.733 |
|  | rs9461650 | G | A | 0.060 | 0.009 | 1.99E-12 | -0.025 | 0.017 | 0.129 |
|  | rs9523295 | G | A | 0.078 | 0.009 | 7.24E-18 | 0.002 | 0.014 | 0.857 |
|  | rs9788635 | C | T | 0.058 | 0.008 | 9.43E-14 | -0.020 | 0.018 | 0.267 |
|  | rs9948462 | C | T | -0.047 | 0.008 | 8.70E-10 | -0.020 | 0.013 | 0.100 |
| *Erysipelotrichaceae* | rs10011838 | G | A | 0.073 | 0.007 | 1.43E-27 | -0.012 | 0.011 | 0.322 |
|  | rs1016565 | G | A | -0.038 | 0.007 | 2.18E-08 | 0.008 | 0.011 | 0.447 |
|  | rs10507349 | G | A | 0.064 | 0.007 | 1.69E-21 | 0.015 | 0.012 | 0.197 |
|  | rs10852123 | A | C | 0.060 | 0.008 | 8.38E-13 | -0.011 | 0.012 | 0.321 |
|  | rs10860209 | C | A | 0.040 | 0.007 | 5.67E-09 | -0.003 | 0.014 | 0.849 |
|  | rs10886863 | T | C | -0.060 | 0.007 | 5.28E-17 | 0.099 | 0.033 | 0.006 |
|  | rs10938398 | G | A | -0.046 | 0.007 | 3.84E-10 | -0.002 | 0.011 | 0.893 |
|  | rs10950550 | T | G | 0.065 | 0.007 | 1.75E-19 | 0.002 | 0.011 | 0.855 |
|  | rs10965248 | T | C | 0.183 | 0.007 | 4.42E-164 | -0.005 | 0.014 | 0.739 |
|  | rs111246699 | G | A | -0.061 | 0.008 | 1.54E-15 | 0.002 | 0.012 | 0.863 |
|  | rs113154802 | C | T | 0.060 | 0.011 | 3.51E-08 | -0.001 | 0.018 | 0.907 |
|  | rs1182444 | A | G | -0.047 | 0.007 | 1.67E-12 | 0.002 | 0.011 | 0.889 |
|  | rs11926494 | G | A | 0.112 | 0.009 | 2.69E-37 | 0.017 | 0.018 | 0.316 |
|  | rs1206684 | G | A | 0.040 | 0.007 | 4.42E-09 | -0.015 | 0.011 | 0.149 |
|  | rs123378 | G | A | 0.053 | 0.008 | 2.22E-10 | 0.004 | 0.011 | 0.755 |
|  | rs1260326 | T | C | -0.063 | 0.007 | 1.01E-21 | 0.000 | 0.011 | 0.991 |
|  | rs12610052 | T | C | -0.046 | 0.008 | 1.97E-09 | -0.026 | 0.039 | 0.517 |
|  | rs12625671 | T | C | -0.066 | 0.007 | 2.25E-21 | 0.007 | 0.015 | 0.575 |
|  | rs1266488 | T | C | 0.044 | 0.007 | 6.29E-10 | 0.000 | 0.033 | 0.931 |
|  | rs12698877 | A | G | -0.067 | 0.007 | 6.96E-22 | -0.031 | 0.012 | 0.009 |
|  | rs12818766 | G | A | -0.054 | 0.009 | 2.46E-10 | 0.005 | 0.014 | 0.739 |
|  | rs13092876 | G | A | -0.126 | 0.007 | 1.91E-66 | 0.015 | 0.011 | 0.197 |
|  | rs13266634 | C | T | 0.116 | 0.007 | 3.72E-67 | -0.002 | 0.011 | 0.887 |
|  | rs1328412 | T | C | 0.097 | 0.015 | 6.41E-11 | -0.062 | 0.021 | 0.002 |
|  | rs1421085 | T | C | -0.130 | 0.009 | 1.55E-48 | 0.005 | 0.011 | 0.597 |
|  | rs1426371 | G | A | 0.048 | 0.007 | 7.76E-12 | -0.002 | 0.012 | 0.873 |
|  | rs1459513 | A | C | -0.046 | 0.007 | 3.73E-11 | 0.033 | 0.023 | 0.218 |
|  | rs147707338 | C | T | -0.087 | 0.014 | 6.47E-10 | 0.036 | 0.018 | 0.048 |
|  | rs1513275 | T | C | -0.057 | 0.009 | 2.76E-11 | 0.033 | 0.012 | 0.011 |
|  | rs16884229 | T | C | -0.097 | 0.007 | 1.15E-43 | -0.030 | 0.013 | 0.026 |
|  | rs17168486 | C | T | -0.064 | 0.007 | 8.23E-22 | -0.010 | 0.013 | 0.487 |
|  | rs1850421 | A | C | 0.044 | 0.007 | 1.41E-09 | 0.000 | 0.011 | 0.989 |
|  | rs2074120 | A | C | 0.041 | 0.007 | 8.38E-09 | 0.008 | 0.011 | 0.468 |
|  | rs2092518 | G | T | 0.046 | 0.007 | 1.39E-10 | -0.006 | 0.011 | 0.587 |
|  | rs2126736 | A | G | 0.038 | 0.007 | 1.84E-08 | -0.006 | 0.011 | 0.572 |
|  | rs2240885 | G | A | -0.042 | 0.007 | 2.79E-09 | -0.022 | 0.013 | 0.094 |
|  | rs2269245 | G | A | 0.054 | 0.009 | 5.40E-10 | -0.007 | 0.013 | 0.575 |
|  | rs2327777 | T | C | 0.050 | 0.007 | 1.06E-13 | 0.007 | 0.011 | 0.507 |
|  | rs2583934 | G | T | -0.058 | 0.007 | 4.95E-16 | 0.001 | 0.014 | 0.950 |
|  | rs261982 | C | T | -0.040 | 0.007 | 3.12E-09 | -0.006 | 0.011 | 0.569 |
|  | rs2706710 | C | T | -0.071 | 0.013 | 1.67E-08 | -0.009 | 0.014 | 0.573 |
|  | rs2796441 | G | A | 0.075 | 0.007 | 1.43E-28 | 0.011 | 0.011 | 0.285 |
|  | rs28599782 | G | A | -0.067 | 0.008 | 4.64E-16 | -0.005 | 0.029 | 0.842 |
|  | rs28691713 | C | T | 0.066 | 0.008 | 1.79E-17 | -0.009 | 0.011 | 0.423 |
|  | rs2908279 | T | G | -0.046 | 0.007 | 8.42E-11 | -0.006 | 0.011 | 0.588 |
|  | rs2980883 | T | G | 0.042 | 0.007 | 3.93E-09 | -0.001 | 0.015 | 0.986 |
|  | rs3094508 | T | C | -0.059 | 0.008 | 1.31E-13 | -0.003 | 0.011 | 0.843 |
|  | rs329122 | G | A | -0.039 | 0.007 | 2.22E-08 | 0.007 | 0.011 | 0.488 |
|  | rs34811727 | G | T | -0.076 | 0.013 | 3.32E-09 | 0.010 | 0.021 | 0.559 |
|  | rs349359 | A | C | -0.043 | 0.008 | 3.05E-08 | 0.014 | 0.016 | 0.427 |
|  | rs35589574 | C | T | -0.171 | 0.011 | 1.58E-55 | -0.010 | 0.012 | 0.462 |
|  | rs3735641 | A | G | -0.069 | 0.008 | 3.62E-19 | 0.014 | 0.011 | 0.207 |
|  | rs3751236 | G | A | 0.067 | 0.007 | 6.58E-21 | -0.013 | 0.013 | 0.324 |
|  | rs3852529 | C | T | -0.099 | 0.010 | 2.07E-24 | 0.002 | 0.018 | 0.963 |
|  | rs3887925 | C | T | -0.040 | 0.007 | 3.12E-09 | 0.008 | 0.011 | 0.480 |
|  | rs391933 | G | A | 0.037 | 0.007 | 1.46E-08 | 0.003 | 0.011 | 0.789 |
|  | rs4273712 | A | G | -0.047 | 0.007 | 2.56E-12 | 0.004 | 0.012 | 0.676 |
|  | rs4499362 | C | T | 0.044 | 0.007 | 1.53E-09 | 0.006 | 0.013 | 0.733 |
|  | rs476828 | T | C | -0.084 | 0.008 | 4.81E-27 | 0.002 | 0.012 | 0.885 |
|  | rs4930974 | C | T | -0.069 | 0.008 | 4.07E-19 | -0.004 | 0.013 | 0.747 |
|  | rs532504 | G | A | -0.055 | 0.008 | 7.39E-12 | 0.015 | 0.013 | 0.275 |
|  | rs55700915 | G | A | -0.040 | 0.007 | 1.50E-08 | 0.019 | 0.013 | 0.146 |
|  | rs58524310 | A | G | -0.047 | 0.007 | 8.41E-11 | -0.017 | 0.015 | 0.248 |
|  | rs58718028 | C | T | -0.073 | 0.007 | 3.35E-25 | -0.011 | 0.012 | 0.357 |
|  | rs6012878 | G | A | 0.041 | 0.007 | 4.32E-09 | 0.001 | 0.011 | 0.904 |
|  | rs60573766 | C | T | 0.044 | 0.007 | 4.30E-10 | 0.037 | 0.015 | 0.012 |
|  | rs61779313 | T | C | -0.060 | 0.009 | 5.59E-11 | 0.006 | 0.012 | 0.683 |
|  | rs61975988 | A | G | 0.040 | 0.007 | 1.97E-09 | -0.015 | 0.012 | 0.199 |
|  | rs62173901 | A | G | 0.123 | 0.020 | 6.58E-10 | -0.016 | 0.015 | 0.246 |
|  | rs62405419 | G | T | -0.044 | 0.008 | 3.79E-09 | -0.016 | 0.016 | 0.260 |
|  | rs633862 | C | T | -0.039 | 0.007 | 1.26E-08 | -0.002 | 0.011 | 0.832 |
|  | rs6416749 | C | T | 0.052 | 0.008 | 3.40E-12 | -0.010 | 0.011 | 0.428 |
|  | rs6731688 | A | C | -0.098 | 0.012 | 2.55E-17 | 0.010 | 0.014 | 0.411 |
|  | rs703980 | G | A | 0.060 | 0.007 | 6.55E-19 | -0.010 | 0.011 | 0.353 |
|  | rs7107784 | G | A | 0.086 | 0.012 | 2.60E-13 | -0.013 | 0.012 | 0.372 |
|  | rs7109575 | G | A | 0.141 | 0.015 | 5.46E-21 | -0.012 | 0.015 | 0.474 |
|  | rs7250869 | T | C | 0.056 | 0.007 | 2.29E-16 | 0.013 | 0.011 | 0.266 |
|  | rs7304270 | C | T | 0.068 | 0.010 | 1.04E-12 | -0.030 | 0.057 | 0.579 |
|  | rs73085586 | G | A | 0.043 | 0.007 | 1.66E-09 | -0.008 | 0.013 | 0.604 |
|  | rs7313668 | G | T | -0.045 | 0.007 | 4.91E-11 | 0.002 | 0.011 | 0.874 |
|  | rs73347525 | A | G | 0.059 | 0.009 | 7.46E-11 | 0.023 | 0.014 | 0.071 |
|  | rs742762 | A | C | 0.075 | 0.008 | 1.79E-22 | -0.002 | 0.017 | 0.978 |
|  | rs7501939 | T | C | 0.120 | 0.007 | 1.60E-60 | 0.003 | 0.011 | 0.816 |
|  | rs75990271 | T | C | 0.066 | 0.010 | 3.22E-11 | -0.077 | 0.024 | 0.001 |
|  | rs7656416 | C | T | 0.100 | 0.007 | 9.01E-42 | -0.002 | 0.031 | 0.753 |
|  | rs76704029 | T | C | 0.055 | 0.010 | 3.39E-08 | -0.042 | 0.024 | 0.119 |
|  | rs76878791 | A | G | -0.053 | 0.007 | 2.02E-13 | 0.038 | 0.046 | 0.470 |
|  | rs77789961 | C | T | -0.047 | 0.008 | 1.92E-08 | 0.042 | 0.039 | 0.406 |
|  | rs7787720 | C | T | -0.054 | 0.007 | 2.25E-15 | -0.006 | 0.011 | 0.536 |
|  | rs7901695 | T | C | -0.275 | 0.017 | 8.18E-62 | -0.004 | 0.012 | 0.697 |
|  | rs80196932 | T | C | 0.060 | 0.008 | 7.57E-13 | -0.008 | 0.015 | 0.574 |
|  | rs8026714 | G | A | -0.066 | 0.007 | 1.06E-22 | -0.005 | 0.015 | 0.840 |
|  | rs8043085 | G | T | -0.052 | 0.007 | 2.06E-14 | 0.025 | 0.012 | 0.043 |
|  | rs896852 | G | T | 0.042 | 0.007 | 6.42E-09 | 0.019 | 0.011 | 0.070 |
|  | rs9316706 | A | G | 0.041 | 0.007 | 3.33E-09 | -0.022 | 0.014 | 0.095 |
|  | rs9350271 | G | A | -0.193 | 0.007 | 4.95E-183 | 0.007 | 0.011 | 0.521 |
|  | rs9379084 | G | A | 0.072 | 0.009 | 2.20E-14 | 0.026 | 0.017 | 0.169 |
|  | rs9390022 | T | C | 0.048 | 0.008 | 6.35E-09 | -0.015 | 0.011 | 0.186 |
|  | rs9461650 | G | A | 0.060 | 0.009 | 1.99E-12 | -0.017 | 0.014 | 0.243 |
|  | rs9523295 | G | A | 0.078 | 0.009 | 7.24E-18 | -0.006 | 0.012 | 0.638 |
|  | rs9788635 | C | T | 0.058 | 0.008 | 9.43E-14 | 0.026 | 0.016 | 0.117 |
|  | rs9948462 | C | T | -0.047 | 0.008 | 8.70E-10 | -0.010 | 0.011 | 0.345 |
| *Lachnospiraceae* | rs10011838 | G | A | 0.073 | 0.007 | 1.43E-27 | -0.011 | 0.011 | 0.350 |
|  | rs1016565 | G | A | -0.038 | 0.007 | 2.18E-08 | 0.003 | 0.011 | 0.740 |
|  | rs10507349 | G | A | 0.064 | 0.007 | 1.69E-21 | -0.027 | 0.012 | 0.026 |
|  | rs10852123 | A | C | 0.060 | 0.008 | 8.38E-13 | 0.002 | 0.012 | 0.861 |
|  | rs10860209 | C | A | 0.040 | 0.007 | 5.67E-09 | -0.008 | 0.014 | 0.645 |
|  | rs10886863 | T | C | -0.060 | 0.007 | 5.28E-17 | -0.015 | 0.033 | 0.660 |
|  | rs10938398 | G | A | -0.046 | 0.007 | 3.84E-10 | -0.001 | 0.011 | 0.895 |
|  | rs10950550 | T | G | 0.065 | 0.007 | 1.75E-19 | 0.010 | 0.011 | 0.368 |
|  | rs10965248 | T | C | 0.183 | 0.007 | 4.42E-164 | -0.002 | 0.014 | 0.998 |
|  | rs111246699 | G | A | -0.061 | 0.008 | 1.54E-15 | -0.005 | 0.012 | 0.646 |
|  | rs113154802 | C | T | 0.060 | 0.011 | 3.51E-08 | 0.002 | 0.018 | 0.955 |
|  | rs1182444 | A | G | -0.047 | 0.007 | 1.67E-12 | 0.020 | 0.011 | 0.058 |
|  | rs11926494 | G | A | 0.112 | 0.009 | 2.69E-37 | -0.026 | 0.018 | 0.173 |
|  | rs1206684 | G | A | 0.040 | 0.007 | 4.42E-09 | 0.019 | 0.011 | 0.096 |
|  | rs123378 | G | A | 0.053 | 0.008 | 2.22E-10 | 0.013 | 0.011 | 0.259 |
|  | rs1260326 | T | C | -0.063 | 0.007 | 1.01E-21 | -0.003 | 0.011 | 0.812 |
|  | rs12610052 | T | C | -0.046 | 0.008 | 1.97E-09 | -0.020 | 0.039 | 0.882 |
|  | rs12625671 | T | C | -0.066 | 0.007 | 2.25E-21 | -0.014 | 0.015 | 0.493 |
|  | rs1266488 | T | C | 0.044 | 0.007 | 6.29E-10 | 0.007 | 0.033 | 0.898 |
|  | rs12698877 | A | G | -0.067 | 0.007 | 6.96E-22 | 0.004 | 0.012 | 0.730 |
|  | rs12818766 | G | A | -0.054 | 0.009 | 2.46E-10 | -0.009 | 0.014 | 0.529 |
|  | rs13092876 | G | A | -0.126 | 0.007 | 1.91E-66 | 0.012 | 0.011 | 0.290 |
|  | rs13266634 | C | T | 0.116 | 0.007 | 3.72E-67 | 0.005 | 0.011 | 0.660 |
|  | rs1328412 | T | C | 0.097 | 0.015 | 6.41E-11 | -0.015 | 0.021 | 0.493 |
|  | rs1421085 | T | C | -0.130 | 0.009 | 1.55E-48 | 0.000 | 0.011 | 0.987 |
|  | rs1426371 | G | A | 0.048 | 0.007 | 7.76E-12 | -0.015 | 0.012 | 0.235 |
|  | rs1459513 | A | C | -0.046 | 0.007 | 3.73E-11 | 0.006 | 0.023 | 0.754 |
|  | rs147707338 | C | T | -0.087 | 0.014 | 6.47E-10 | -0.020 | 0.018 | 0.324 |
|  | rs1513275 | T | C | -0.057 | 0.009 | 2.76E-11 | 0.021 | 0.012 | 0.098 |
|  | rs16884229 | T | C | -0.097 | 0.007 | 1.15E-43 | -0.014 | 0.013 | 0.320 |
|  | rs17168486 | C | T | -0.064 | 0.007 | 8.23E-22 | 0.007 | 0.013 | 0.559 |
|  | rs1850421 | A | C | 0.044 | 0.007 | 1.41E-09 | 0.003 | 0.011 | 0.798 |
|  | rs2074120 | A | C | 0.041 | 0.007 | 8.38E-09 | 0.009 | 0.010 | 0.397 |
|  | rs2092518 | G | T | 0.046 | 0.007 | 1.39E-10 | 0.003 | 0.011 | 0.746 |
|  | rs2126736 | A | G | 0.038 | 0.007 | 1.84E-08 | 0.013 | 0.011 | 0.220 |
|  | rs2240885 | G | A | -0.042 | 0.007 | 2.79E-09 | 0.001 | 0.013 | 0.886 |
|  | rs2269245 | G | A | 0.054 | 0.009 | 5.40E-10 | -0.009 | 0.013 | 0.514 |
|  | rs2327777 | T | C | 0.050 | 0.007 | 1.06E-13 | 0.000 | 0.010 | 0.995 |
|  | rs2583934 | G | T | -0.058 | 0.007 | 4.95E-16 | -0.019 | 0.014 | 0.194 |
|  | rs261982 | C | T | -0.040 | 0.007 | 3.12E-09 | -0.015 | 0.011 | 0.177 |
|  | rs2706710 | C | T | -0.071 | 0.013 | 1.67E-08 | -0.006 | 0.014 | 0.634 |
|  | rs2796441 | G | A | 0.075 | 0.007 | 1.43E-28 | -0.009 | 0.011 | 0.397 |
|  | rs28599782 | G | A | -0.067 | 0.008 | 4.64E-16 | -0.009 | 0.029 | 0.747 |
|  | rs28691713 | C | T | 0.066 | 0.008 | 1.79E-17 | -0.014 | 0.011 | 0.178 |
|  | rs2908279 | T | G | -0.046 | 0.007 | 8.42E-11 | -0.013 | 0.010 | 0.207 |
|  | rs2980883 | T | G | 0.042 | 0.007 | 3.93E-09 | -0.026 | 0.015 | 0.090 |
|  | rs3094508 | T | C | -0.059 | 0.008 | 1.31E-13 | 0.007 | 0.011 | 0.529 |
|  | rs329122 | G | A | -0.039 | 0.007 | 2.22E-08 | 0.015 | 0.011 | 0.153 |
|  | rs34811727 | G | T | -0.076 | 0.013 | 3.32E-09 | -0.017 | 0.021 | 0.442 |
|  | rs349359 | A | C | -0.043 | 0.008 | 3.05E-08 | 0.001 | 0.016 | 0.938 |
|  | rs35589574 | C | T | -0.171 | 0.011 | 1.58E-55 | 0.002 | 0.012 | 0.941 |
|  | rs3735641 | A | G | -0.069 | 0.008 | 3.62E-19 | -0.024 | 0.011 | 0.029 |
|  | rs3751236 | G | A | 0.067 | 0.007 | 6.58E-21 | 0.012 | 0.013 | 0.345 |
|  | rs3852529 | C | T | -0.099 | 0.010 | 2.07E-24 | -0.019 | 0.018 | 0.333 |
|  | rs3887925 | C | T | -0.040 | 0.007 | 3.12E-09 | -0.004 | 0.011 | 0.695 |
|  | rs391933 | G | A | 0.037 | 0.007 | 1.46E-08 | 0.011 | 0.011 | 0.284 |
|  | rs4273712 | A | G | -0.047 | 0.007 | 2.56E-12 | 0.014 | 0.012 | 0.229 |
|  | rs4499362 | C | T | 0.044 | 0.007 | 1.53E-09 | -0.014 | 0.013 | 0.281 |
|  | rs476828 | T | C | -0.084 | 0.008 | 4.81E-27 | 0.012 | 0.012 | 0.291 |
|  | rs4930974 | C | T | -0.069 | 0.008 | 4.07E-19 | 0.000 | 0.013 | 0.999 |
|  | rs532504 | G | A | -0.055 | 0.008 | 7.39E-12 | -0.005 | 0.013 | 0.694 |
|  | rs55700915 | G | A | -0.040 | 0.007 | 1.50E-08 | 0.000 | 0.013 | 0.973 |
|  | rs58524310 | A | G | -0.047 | 0.007 | 8.41E-11 | 0.014 | 0.015 | 0.374 |
|  | rs58718028 | C | T | -0.073 | 0.007 | 3.35E-25 | 0.007 | 0.012 | 0.583 |
|  | rs6012878 | G | A | 0.041 | 0.007 | 4.32E-09 | -0.011 | 0.011 | 0.312 |
|  | rs60573766 | C | T | 0.044 | 0.007 | 4.30E-10 | 0.026 | 0.015 | 0.081 |
|  | rs61779313 | T | C | -0.060 | 0.009 | 5.59E-11 | -0.001 | 0.012 | 0.950 |
|  | rs61975988 | A | G | 0.040 | 0.007 | 1.97E-09 | -0.020 | 0.012 | 0.096 |
|  | rs62173901 | A | G | 0.123 | 0.020 | 6.58E-10 | -0.003 | 0.015 | 0.777 |
|  | rs62405419 | G | T | -0.044 | 0.008 | 3.79E-09 | -0.026 | 0.016 | 0.108 |
|  | rs633862 | C | T | -0.039 | 0.007 | 1.26E-08 | -0.009 | 0.011 | 0.411 |
|  | rs6416749 | C | T | 0.052 | 0.008 | 3.40E-12 | 0.007 | 0.011 | 0.510 |
|  | rs6731688 | A | C | -0.098 | 0.012 | 2.55E-17 | 0.015 | 0.014 | 0.283 |
|  | rs703980 | G | A | 0.060 | 0.007 | 6.55E-19 | 0.002 | 0.010 | 0.890 |
|  | rs7107784 | G | A | 0.086 | 0.012 | 2.60E-13 | -0.018 | 0.012 | 0.128 |
|  | rs7109575 | G | A | 0.141 | 0.015 | 5.46E-21 | 0.010 | 0.015 | 0.534 |
|  | rs7250869 | T | C | 0.056 | 0.007 | 2.29E-16 | -0.001 | 0.011 | 0.938 |
|  | rs7304270 | C | T | 0.068 | 0.010 | 1.04E-12 | 0.102 | 0.057 | 0.072 |
|  | rs73085586 | G | A | 0.043 | 0.007 | 1.66E-09 | 0.025 | 0.013 | 0.054 |
|  | rs7313668 | G | T | -0.045 | 0.007 | 4.91E-11 | -0.030 | 0.011 | 0.005 |
|  | rs73347525 | A | G | 0.059 | 0.009 | 7.46E-11 | 0.017 | 0.014 | 0.211 |
|  | rs742762 | A | C | 0.075 | 0.008 | 1.79E-22 | 0.012 | 0.017 | 0.437 |
|  | rs7501939 | T | C | 0.120 | 0.007 | 1.60E-60 | -0.010 | 0.011 | 0.394 |
|  | rs75990271 | T | C | 0.066 | 0.010 | 3.22E-11 | -0.036 | 0.024 | 0.118 |
|  | rs7656416 | C | T | 0.100 | 0.007 | 9.01E-42 | 0.022 | 0.030 | 0.342 |
|  | rs76704029 | T | C | 0.055 | 0.010 | 3.39E-08 | 0.005 | 0.024 | 0.704 |
|  | rs76878791 | A | G | -0.053 | 0.007 | 2.02E-13 | 0.039 | 0.046 | 0.483 |
|  | rs77789961 | C | T | -0.047 | 0.008 | 1.92E-08 | -0.017 | 0.039 | 0.562 |
|  | rs7787720 | C | T | -0.054 | 0.007 | 2.25E-15 | -0.003 | 0.011 | 0.790 |
|  | rs7901695 | T | C | -0.275 | 0.017 | 8.18E-62 | -0.003 | 0.011 | 0.843 |
|  | rs80196932 | T | C | 0.060 | 0.008 | 7.57E-13 | 0.014 | 0.015 | 0.362 |
|  | rs8026714 | G | A | -0.066 | 0.007 | 1.06E-22 | -0.004 | 0.015 | 0.897 |
|  | rs8043085 | G | T | -0.052 | 0.007 | 2.06E-14 | 0.022 | 0.012 | 0.064 |
|  | rs896852 | G | T | 0.042 | 0.007 | 6.42E-09 | -0.004 | 0.011 | 0.691 |
|  | rs9316706 | A | G | 0.041 | 0.007 | 3.33E-09 | -0.005 | 0.014 | 0.719 |
|  | rs9350271 | G | A | -0.193 | 0.007 | 4.95E-183 | -0.017 | 0.011 | 0.118 |
|  | rs9379084 | G | A | 0.072 | 0.009 | 2.20E-14 | 0.018 | 0.017 | 0.298 |
|  | rs9390022 | T | C | 0.048 | 0.008 | 6.35E-09 | -0.009 | 0.011 | 0.428 |
|  | rs9461650 | G | A | 0.060 | 0.009 | 1.99E-12 | -0.010 | 0.014 | 0.510 |
|  | rs9523295 | G | A | 0.078 | 0.009 | 7.24E-18 | -0.008 | 0.012 | 0.540 |
|  | rs9788635 | C | T | 0.058 | 0.008 | 9.43E-14 | 0.001 | 0.016 | 0.859 |
|  | rs9948462 | C | T | -0.047 | 0.008 | 8.70E-10 | 0.006 | 0.011 | 0.573 |
| *Lactobacillaceae* | rs10011838 | G | A | 0.073 | 0.007 | 1.43E-27 | 0.001 | 0.019 | 0.949 |
|  | rs1016565 | G | A | -0.038 | 0.007 | 2.18E-08 | 0.005 | 0.018 | 0.747 |
|  | rs10507349 | G | A | 0.064 | 0.007 | 1.69E-21 | -0.016 | 0.020 | 0.421 |
|  | rs10852123 | A | C | 0.060 | 0.008 | 8.38E-13 | 0.009 | 0.020 | 0.665 |
|  | rs10860209 | C | A | 0.040 | 0.007 | 5.67E-09 | -0.010 | 0.023 | 0.698 |
|  | rs10938398 | G | A | -0.046 | 0.007 | 3.84E-10 | 0.011 | 0.017 | 0.517 |
|  | rs10950550 | T | G | 0.065 | 0.007 | 1.75E-19 | -0.014 | 0.017 | 0.398 |
|  | rs10965248 | T | C | 0.183 | 0.007 | 4.42E-164 | -0.029 | 0.022 | 0.174 |
|  | rs111246699 | G | A | -0.061 | 0.008 | 1.54E-15 | -0.010 | 0.020 | 0.680 |
|  | rs113154802 | C | T | 0.060 | 0.011 | 3.51E-08 | -0.041 | 0.029 | 0.190 |
|  | rs1182444 | A | G | -0.047 | 0.007 | 1.67E-12 | 0.001 | 0.018 | 0.926 |
|  | rs11926494 | G | A | 0.112 | 0.009 | 2.69E-37 | 0.044 | 0.029 | 0.143 |
|  | rs1206684 | G | A | 0.040 | 0.007 | 4.42E-09 | -0.006 | 0.018 | 0.751 |
|  | rs123378 | G | A | 0.053 | 0.008 | 2.22E-10 | 0.002 | 0.018 | 0.970 |
|  | rs1260326 | T | C | -0.063 | 0.007 | 1.01E-21 | 0.003 | 0.017 | 0.848 |
|  | rs12625671 | T | C | -0.066 | 0.007 | 2.25E-21 | 0.018 | 0.025 | 0.412 |
|  | rs12698877 | A | G | -0.067 | 0.007 | 6.96E-22 | 0.028 | 0.019 | 0.146 |
|  | rs12818766 | G | A | -0.054 | 0.009 | 2.46E-10 | -0.021 | 0.023 | 0.368 |
|  | rs13092876 | G | A | -0.126 | 0.007 | 1.91E-66 | 0.010 | 0.018 | 0.614 |
|  | rs13266634 | C | T | 0.116 | 0.007 | 3.72E-67 | -0.024 | 0.018 | 0.195 |
|  | rs1328412 | T | C | 0.097 | 0.015 | 6.41E-11 | -0.081 | 0.034 | 0.014 |
|  | rs1421085 | T | C | -0.130 | 0.009 | 1.55E-48 | -0.013 | 0.017 | 0.438 |
|  | rs1426371 | G | A | 0.048 | 0.007 | 7.76E-12 | 0.052 | 0.019 | 0.005 |
|  | rs1459513 | A | C | -0.046 | 0.007 | 3.73E-11 | -0.025 | 0.036 | 0.852 |
|  | rs147707338 | C | T | -0.087 | 0.014 | 6.47E-10 | -0.015 | 0.029 | 0.675 |
|  | rs1513275 | T | C | -0.057 | 0.009 | 2.76E-11 | 0.006 | 0.020 | 0.720 |
|  | rs16884229 | T | C | -0.097 | 0.007 | 1.15E-43 | 0.001 | 0.021 | 0.977 |
|  | rs17168486 | C | T | -0.064 | 0.007 | 8.23E-22 | 0.043 | 0.022 | 0.048 |
|  | rs1850421 | A | C | 0.044 | 0.007 | 1.41E-09 | 0.001 | 0.018 | 0.975 |
|  | rs2074120 | A | C | 0.041 | 0.007 | 8.38E-09 | -0.029 | 0.017 | 0.084 |
|  | rs2092518 | G | T | 0.046 | 0.007 | 1.39E-10 | -0.011 | 0.017 | 0.529 |
|  | rs2126736 | A | G | 0.038 | 0.007 | 1.84E-08 | -0.026 | 0.017 | 0.125 |
|  | rs2240885 | G | A | -0.042 | 0.007 | 2.79E-09 | -0.033 | 0.020 | 0.109 |
|  | rs2269245 | G | A | 0.054 | 0.009 | 5.40E-10 | -0.023 | 0.021 | 0.290 |
|  | rs2327777 | T | C | 0.050 | 0.007 | 1.06E-13 | -0.013 | 0.017 | 0.450 |
|  | rs2583934 | G | T | -0.058 | 0.007 | 4.95E-16 | -0.016 | 0.023 | 0.521 |
|  | rs261982 | C | T | -0.040 | 0.007 | 3.12E-09 | 0.010 | 0.017 | 0.576 |
|  | rs2706710 | C | T | -0.071 | 0.013 | 1.67E-08 | 0.011 | 0.022 | 0.604 |
|  | rs2796441 | G | A | 0.075 | 0.007 | 1.43E-28 | -0.003 | 0.017 | 0.843 |
|  | rs28691713 | C | T | 0.066 | 0.008 | 1.79E-17 | -0.034 | 0.017 | 0.049 |
|  | rs2908279 | T | G | -0.046 | 0.007 | 8.42E-11 | 0.013 | 0.017 | 0.430 |
|  | rs2980883 | T | G | 0.042 | 0.007 | 3.93E-09 | 0.021 | 0.024 | 0.371 |
|  | rs3094508 | T | C | -0.059 | 0.008 | 1.31E-13 | -0.004 | 0.017 | 0.865 |
|  | rs329122 | G | A | -0.039 | 0.007 | 2.22E-08 | 0.022 | 0.017 | 0.205 |
|  | rs34811727 | G | T | -0.076 | 0.013 | 3.32E-09 | -0.014 | 0.035 | 0.701 |
|  | rs349359 | A | C | -0.043 | 0.008 | 3.05E-08 | 0.034 | 0.026 | 0.211 |
|  | rs35589574 | C | T | -0.171 | 0.011 | 1.58E-55 | -0.009 | 0.020 | 0.718 |
|  | rs3735641 | A | G | -0.069 | 0.008 | 3.62E-19 | -0.002 | 0.017 | 0.912 |
|  | rs3751236 | G | A | 0.067 | 0.007 | 6.58E-21 | 0.011 | 0.021 | 0.616 |
|  | rs3852529 | C | T | -0.099 | 0.010 | 2.07E-24 | 0.036 | 0.030 | 0.205 |
|  | rs3887925 | C | T | -0.040 | 0.007 | 3.12E-09 | 0.001 | 0.017 | 0.966 |
|  | rs391933 | G | A | 0.037 | 0.007 | 1.46E-08 | -0.010 | 0.017 | 0.584 |
|  | rs4273712 | A | G | -0.047 | 0.007 | 2.56E-12 | 0.026 | 0.019 | 0.163 |
|  | rs4499362 | C | T | 0.044 | 0.007 | 1.53E-09 | 0.008 | 0.021 | 0.711 |
|  | rs476828 | T | C | -0.084 | 0.008 | 4.81E-27 | 0.011 | 0.020 | 0.574 |
|  | rs4930974 | C | T | -0.069 | 0.008 | 4.07E-19 | -0.026 | 0.020 | 0.182 |
|  | rs532504 | G | A | -0.055 | 0.008 | 7.39E-12 | 0.003 | 0.021 | 0.870 |
|  | rs55700915 | G | A | -0.040 | 0.007 | 1.50E-08 | 0.038 | 0.021 | 0.059 |
|  | rs58524310 | A | G | -0.047 | 0.007 | 8.41E-11 | 0.012 | 0.023 | 0.645 |
|  | rs58718028 | C | T | -0.073 | 0.007 | 3.35E-25 | 0.006 | 0.020 | 0.746 |
|  | rs6012878 | G | A | 0.041 | 0.007 | 4.32E-09 | -0.004 | 0.017 | 0.827 |
|  | rs60573766 | C | T | 0.044 | 0.007 | 4.30E-10 | 0.014 | 0.024 | 0.525 |
|  | rs61779313 | T | C | -0.060 | 0.009 | 5.59E-11 | -0.015 | 0.020 | 0.436 |
|  | rs61975988 | A | G | 0.040 | 0.007 | 1.97E-09 | 0.016 | 0.019 | 0.442 |
|  | rs62173901 | A | G | 0.123 | 0.020 | 6.58E-10 | 0.021 | 0.025 | 0.423 |
|  | rs62405419 | G | T | -0.044 | 0.008 | 3.79E-09 | -0.010 | 0.026 | 0.818 |
|  | rs633862 | C | T | -0.039 | 0.007 | 1.26E-08 | -0.018 | 0.017 | 0.288 |
|  | rs6416749 | C | T | 0.052 | 0.008 | 3.40E-12 | -0.038 | 0.018 | 0.034 |
|  | rs6731688 | A | C | -0.098 | 0.012 | 2.55E-17 | 0.047 | 0.023 | 0.036 |
|  | rs703980 | G | A | 0.060 | 0.007 | 6.55E-19 | 0.024 | 0.017 | 0.154 |
|  | rs7107784 | G | A | 0.086 | 0.012 | 2.60E-13 | 0.034 | 0.019 | 0.072 |
|  | rs7109575 | G | A | 0.141 | 0.015 | 5.46E-21 | -0.012 | 0.023 | 0.605 |
|  | rs7250869 | T | C | 0.056 | 0.007 | 2.29E-16 | -0.014 | 0.018 | 0.442 |
|  | rs73085586 | G | A | 0.043 | 0.007 | 1.66E-09 | 0.010 | 0.021 | 0.644 |
|  | rs7313668 | G | T | -0.045 | 0.007 | 4.91E-11 | -0.029 | 0.017 | 0.088 |
|  | rs73347525 | A | G | 0.059 | 0.009 | 7.46E-11 | -0.004 | 0.022 | 0.858 |
|  | rs742762 | A | C | 0.075 | 0.008 | 1.79E-22 | 0.017 | 0.027 | 0.668 |
|  | rs7501939 | T | C | 0.120 | 0.007 | 1.60E-60 | 0.019 | 0.017 | 0.271 |
|  | rs75990271 | T | C | 0.066 | 0.010 | 3.22E-11 | -0.026 | 0.037 | 0.483 |
|  | rs76704029 | T | C | 0.055 | 0.010 | 3.39E-08 | 0.010 | 0.042 | 0.942 |
|  | rs7787720 | C | T | -0.054 | 0.007 | 2.25E-15 | -0.020 | 0.017 | 0.247 |
|  | rs7901695 | T | C | -0.275 | 0.017 | 8.18E-62 | -0.049 | 0.019 | 0.009 |
|  | rs80196932 | T | C | 0.060 | 0.008 | 7.57E-13 | 0.012 | 0.024 | 0.522 |
|  | rs8026714 | G | A | -0.066 | 0.007 | 1.06E-22 | 0.028 | 0.024 | 0.291 |
|  | rs8043085 | G | T | -0.052 | 0.007 | 2.06E-14 | 0.001 | 0.020 | 0.936 |
|  | rs896852 | G | T | 0.042 | 0.007 | 6.42E-09 | 0.001 | 0.017 | 0.948 |
|  | rs9316706 | A | G | 0.041 | 0.007 | 3.33E-09 | -0.013 | 0.022 | 0.594 |
|  | rs9350271 | G | A | -0.193 | 0.007 | 4.95E-183 | 0.018 | 0.018 | 0.299 |
|  | rs9379084 | G | A | 0.072 | 0.009 | 2.20E-14 | -0.058 | 0.028 | 0.040 |
|  | rs9390022 | T | C | 0.048 | 0.008 | 6.35E-09 | 0.031 | 0.018 | 0.110 |
|  | rs9461650 | G | A | 0.060 | 0.009 | 1.99E-12 | 0.002 | 0.023 | 0.955 |
|  | rs9523295 | G | A | 0.078 | 0.009 | 7.24E-18 | 0.037 | 0.020 | 0.051 |
|  | rs9788635 | C | T | 0.058 | 0.008 | 9.43E-14 | 0.011 | 0.026 | 0.661 |
|  | rs9948462 | C | T | -0.047 | 0.008 | 8.70E-10 | 0.024 | 0.017 | 0.159 |
| *Methanobacteriaceae* | rs10011838 | G | A | 0.073 | 0.007 | 1.43E-27 | 0.028 | 0.027 | 0.297 |
|  | rs1016565 | G | A | -0.038 | 0.007 | 2.18E-08 | -0.031 | 0.025 | 0.221 |
|  | rs10507349 | G | A | 0.064 | 0.007 | 1.69E-21 | -0.002 | 0.028 | 0.816 |
|  | rs10852123 | A | C | 0.060 | 0.008 | 8.38E-13 | -0.025 | 0.028 | 0.355 |
|  | rs10860209 | C | A | 0.040 | 0.007 | 5.67E-09 | -0.025 | 0.032 | 0.477 |
|  | rs10938398 | G | A | -0.046 | 0.007 | 3.84E-10 | -0.054 | 0.024 | 0.023 |
|  | rs10950550 | T | G | 0.065 | 0.007 | 1.75E-19 | -0.035 | 0.023 | 0.143 |
|  | rs10965248 | T | C | 0.183 | 0.007 | 4.42E-164 | -0.016 | 0.030 | 0.656 |
|  | rs111246699 | G | A | -0.061 | 0.008 | 1.54E-15 | -0.048 | 0.028 | 0.094 |
|  | rs113154802 | C | T | 0.060 | 0.011 | 3.51E-08 | -0.021 | 0.041 | 0.683 |
|  | rs1182444 | A | G | -0.047 | 0.007 | 1.67E-12 | 0.006 | 0.024 | 0.802 |
|  | rs11926494 | G | A | 0.112 | 0.009 | 2.69E-37 | -0.028 | 0.040 | 0.834 |
|  | rs1206684 | G | A | 0.040 | 0.007 | 4.42E-09 | -0.014 | 0.025 | 0.575 |
|  | rs123378 | G | A | 0.053 | 0.008 | 2.22E-10 | -0.022 | 0.025 | 0.381 |
|  | rs1260326 | T | C | -0.063 | 0.007 | 1.01E-21 | 0.023 | 0.024 | 0.343 |
|  | rs12625671 | T | C | -0.066 | 0.007 | 2.25E-21 | 0.019 | 0.035 | 0.604 |
|  | rs12698877 | A | G | -0.067 | 0.007 | 6.96E-22 | -0.009 | 0.026 | 0.716 |
|  | rs12818766 | G | A | -0.054 | 0.009 | 2.46E-10 | -0.018 | 0.033 | 0.582 |
|  | rs13092876 | G | A | -0.126 | 0.007 | 1.91E-66 | -0.017 | 0.025 | 0.484 |
|  | rs13266634 | C | T | 0.116 | 0.007 | 3.72E-67 | 0.007 | 0.025 | 0.808 |
|  | rs1328412 | T | C | 0.097 | 0.015 | 6.41E-11 | -0.018 | 0.048 | 0.681 |
|  | rs1421085 | T | C | -0.130 | 0.009 | 1.55E-48 | -0.044 | 0.024 | 0.067 |
|  | rs1426371 | G | A | 0.048 | 0.007 | 7.76E-12 | 0.013 | 0.027 | 0.647 |
|  | rs1459513 | A | C | -0.046 | 0.007 | 3.73E-11 | -0.015 | 0.053 | 0.794 |
|  | rs147707338 | C | T | -0.087 | 0.014 | 6.47E-10 | 0.003 | 0.039 | 0.970 |
|  | rs1513275 | T | C | -0.057 | 0.009 | 2.76E-11 | -0.050 | 0.028 | 0.099 |
|  | rs16884229 | T | C | -0.097 | 0.007 | 1.15E-43 | -0.043 | 0.030 | 0.131 |
|  | rs17168486 | C | T | -0.064 | 0.007 | 8.23E-22 | 0.006 | 0.031 | 0.911 |
|  | rs1850421 | A | C | 0.044 | 0.007 | 1.41E-09 | -0.003 | 0.025 | 0.886 |
|  | rs2074120 | A | C | 0.041 | 0.007 | 8.38E-09 | 0.035 | 0.023 | 0.147 |
|  | rs2092518 | G | T | 0.046 | 0.007 | 1.39E-10 | 0.011 | 0.024 | 0.650 |
|  | rs2126736 | A | G | 0.038 | 0.007 | 1.84E-08 | 0.007 | 0.023 | 0.759 |
|  | rs2240885 | G | A | -0.042 | 0.007 | 2.79E-09 | -0.008 | 0.028 | 0.869 |
|  | rs2269245 | G | A | 0.054 | 0.009 | 5.40E-10 | 0.017 | 0.029 | 0.592 |
|  | rs2327777 | T | C | 0.050 | 0.007 | 1.06E-13 | -0.008 | 0.023 | 0.723 |
|  | rs2583934 | G | T | -0.058 | 0.007 | 4.95E-16 | 0.008 | 0.032 | 0.722 |
|  | rs261982 | C | T | -0.040 | 0.007 | 3.12E-09 | -0.015 | 0.024 | 0.568 |
|  | rs2706710 | C | T | -0.071 | 0.013 | 1.67E-08 | -0.060 | 0.031 | 0.063 |
|  | rs2796441 | G | A | 0.075 | 0.007 | 1.43E-28 | 0.004 | 0.024 | 0.914 |
|  | rs28691713 | C | T | 0.066 | 0.008 | 1.79E-17 | 0.019 | 0.025 | 0.432 |
|  | rs2908279 | T | G | -0.046 | 0.007 | 8.42E-11 | -0.019 | 0.023 | 0.446 |
|  | rs2980883 | T | G | 0.042 | 0.007 | 3.93E-09 | 0.029 | 0.032 | 0.353 |
|  | rs3094508 | T | C | -0.059 | 0.008 | 1.31E-13 | -0.001 | 0.024 | 0.959 |
|  | rs329122 | G | A | -0.039 | 0.007 | 2.22E-08 | 0.019 | 0.024 | 0.437 |
|  | rs34811727 | G | T | -0.076 | 0.013 | 3.32E-09 | -0.029 | 0.047 | 0.538 |
|  | rs349359 | A | C | -0.043 | 0.008 | 3.05E-08 | 0.000 | 0.036 | 0.941 |
|  | rs35589574 | C | T | -0.171 | 0.011 | 1.58E-55 | 0.017 | 0.029 | 0.524 |
|  | rs3735641 | A | G | -0.069 | 0.008 | 3.62E-19 | 0.009 | 0.024 | 0.692 |
|  | rs3751236 | G | A | 0.067 | 0.007 | 6.58E-21 | -0.019 | 0.029 | 0.537 |
|  | rs3852529 | C | T | -0.099 | 0.010 | 2.07E-24 | -0.044 | 0.042 | 0.252 |
|  | rs3887925 | C | T | -0.040 | 0.007 | 3.12E-09 | -0.042 | 0.023 | 0.073 |
|  | rs391933 | G | A | 0.037 | 0.007 | 1.46E-08 | 0.000 | 0.024 | 0.991 |
|  | rs4273712 | A | G | -0.047 | 0.007 | 2.56E-12 | -0.004 | 0.026 | 0.857 |
|  | rs4499362 | C | T | 0.044 | 0.007 | 1.53E-09 | -0.024 | 0.029 | 0.386 |
|  | rs476828 | T | C | -0.084 | 0.008 | 4.81E-27 | -0.028 | 0.027 | 0.335 |
|  | rs4930974 | C | T | -0.069 | 0.008 | 4.07E-19 | -0.001 | 0.028 | 0.953 |
|  | rs532504 | G | A | -0.055 | 0.008 | 7.39E-12 | -0.042 | 0.030 | 0.178 |
|  | rs55700915 | G | A | -0.040 | 0.007 | 1.50E-08 | 0.034 | 0.030 | 0.299 |
|  | rs58524310 | A | G | -0.047 | 0.007 | 8.41E-11 | 0.068 | 0.032 | 0.037 |
|  | rs58718028 | C | T | -0.073 | 0.007 | 3.35E-25 | -0.017 | 0.027 | 0.532 |
|  | rs6012878 | G | A | 0.041 | 0.007 | 4.32E-09 | -0.027 | 0.025 | 0.258 |
|  | rs60573766 | C | T | 0.044 | 0.007 | 4.30E-10 | 0.069 | 0.034 | 0.034 |
|  | rs61779313 | T | C | -0.060 | 0.009 | 5.59E-11 | -0.053 | 0.028 | 0.058 |
|  | rs61975988 | A | G | 0.040 | 0.007 | 1.97E-09 | 0.010 | 0.027 | 0.703 |
|  | rs62173901 | A | G | 0.123 | 0.020 | 6.58E-10 | 0.020 | 0.034 | 0.550 |
|  | rs62405419 | G | T | -0.044 | 0.008 | 3.79E-09 | -0.024 | 0.037 | 0.395 |
|  | rs633862 | C | T | -0.039 | 0.007 | 1.26E-08 | 0.033 | 0.023 | 0.162 |
|  | rs6416749 | C | T | 0.052 | 0.008 | 3.40E-12 | -0.033 | 0.026 | 0.214 |
|  | rs6731688 | A | C | -0.098 | 0.012 | 2.55E-17 | 0.022 | 0.031 | 0.522 |
|  | rs703980 | G | A | 0.060 | 0.007 | 6.55E-19 | 0.049 | 0.023 | 0.038 |
|  | rs7107784 | G | A | 0.086 | 0.012 | 2.60E-13 | 0.034 | 0.027 | 0.285 |
|  | rs7109575 | G | A | 0.141 | 0.015 | 5.46E-21 | -0.033 | 0.032 | 0.261 |
|  | rs7250869 | T | C | 0.056 | 0.007 | 2.29E-16 | -0.003 | 0.026 | 0.891 |
|  | rs73085586 | G | A | 0.043 | 0.007 | 1.66E-09 | -0.022 | 0.029 | 0.481 |
|  | rs7313668 | G | T | -0.045 | 0.007 | 4.91E-11 | -0.013 | 0.024 | 0.591 |
|  | rs73347525 | A | G | 0.059 | 0.009 | 7.46E-11 | -0.008 | 0.032 | 0.807 |
|  | rs742762 | A | C | 0.075 | 0.008 | 1.79E-22 | 0.048 | 0.038 | 0.134 |
|  | rs7501939 | T | C | 0.120 | 0.007 | 1.60E-60 | 0.034 | 0.024 | 0.129 |
|  | rs7787720 | C | T | -0.054 | 0.007 | 2.25E-15 | -0.010 | 0.023 | 0.674 |
|  | rs7901695 | T | C | -0.275 | 0.017 | 8.18E-62 | 0.020 | 0.025 | 0.443 |
|  | rs80196932 | T | C | 0.060 | 0.008 | 7.57E-13 | -0.005 | 0.033 | 0.874 |
|  | rs8026714 | G | A | -0.066 | 0.007 | 1.06E-22 | 0.026 | 0.034 | 0.434 |
|  | rs8043085 | G | T | -0.052 | 0.007 | 2.06E-14 | 0.030 | 0.027 | 0.288 |
|  | rs896852 | G | T | 0.042 | 0.007 | 6.42E-09 | -0.007 | 0.023 | 0.724 |
|  | rs9316706 | A | G | 0.041 | 0.007 | 3.33E-09 | 0.034 | 0.031 | 0.314 |
|  | rs9350271 | G | A | -0.193 | 0.007 | 4.95E-183 | 0.021 | 0.025 | 0.373 |
|  | rs9379084 | G | A | 0.072 | 0.009 | 2.20E-14 | -0.039 | 0.038 | 0.309 |
|  | rs9390022 | T | C | 0.048 | 0.008 | 6.35E-09 | 0.025 | 0.026 | 0.346 |
|  | rs9461650 | G | A | 0.060 | 0.009 | 1.99E-12 | -0.015 | 0.033 | 0.726 |
|  | rs9523295 | G | A | 0.078 | 0.009 | 7.24E-18 | 0.022 | 0.027 | 0.403 |
|  | rs9788635 | C | T | 0.058 | 0.008 | 9.43E-14 | -0.021 | 0.037 | 0.658 |
|  | rs9948462 | C | T | -0.047 | 0.008 | 8.70E-10 | -0.009 | 0.024 | 0.655 |
| *Oxalobacteraceae* | rs10011838 | G | A | 0.073 | 0.000 | 1.54E+08 | -0.013 | 0.022 | 0.568 |
|  | rs1016565 | G | A | -0.038 | 0.000 | 1.03E+06 | -0.012 | 0.021 | 0.567 |
|  | rs10507349 | G | A | 0.064 | 0.000 | 2.68E+07 | -0.008 | 0.023 | 0.687 |
|  | rs10852123 | A | C | 0.060 | 0.000 | 9.04E+07 | 0.024 | 0.023 | 0.313 |
|  | rs10860209 | C | A | 0.040 | 0.000 | 9.79E+07 | -0.005 | 0.027 | 0.704 |
|  | rs10938398 | G | A | -0.046 | 0.000 | 4.52E+07 | 0.013 | 0.020 | 0.499 |
|  | rs10950550 | T | G | 0.065 | 0.000 | 1.51E+07 | -0.016 | 0.020 | 0.420 |
|  | rs10965248 | T | C | 0.183 | 0.000 | 2.21E+07 | -0.039 | 0.025 | 0.128 |
|  | rs111246699 | G | A | -0.061 | 0.000 | 1.18E+08 | 0.020 | 0.024 | 0.402 |
|  | rs113154802 | C | T | 0.060 | 0.000 | 9.83E+07 | -0.015 | 0.033 | 0.661 |
|  | rs1182444 | A | G | -0.047 | 0.000 | 1.57E+08 | -0.019 | 0.021 | 0.374 |
|  | rs11926494 | G | A | 0.112 | 0.000 | 2.33E+07 | -0.105 | 0.034 | 0.003 |
|  | rs1206684 | G | A | 0.040 | 0.000 | 1.32E+08 | -0.031 | 0.021 | 0.121 |
|  | rs123378 | G | A | 0.053 | 0.000 | 5.11E+07 | -0.025 | 0.021 | 0.245 |
|  | rs1260326 | T | C | -0.063 | 0.000 | 2.77E+07 | 0.011 | 0.020 | 0.593 |
|  | rs12625671 | T | C | -0.066 | 0.000 | 4.30E+07 | 0.045 | 0.030 | 0.092 |
|  | rs12698877 | A | G | -0.067 | 0.000 | 6.97E+07 | 0.010 | 0.022 | 0.681 |
|  | rs12818766 | G | A | -0.054 | 0.000 | 4.38E+06 | 0.011 | 0.027 | 0.694 |
|  | rs13092876 | G | A | -0.126 | 0.000 | 1.85E+08 | 0.010 | 0.021 | 0.613 |
|  | rs13266634 | C | T | 0.116 | 0.000 | 1.18E+08 | -0.040 | 0.021 | 0.064 |
|  | rs1328412 | T | C | 0.097 | 0.000 | 8.19E+07 | 0.073 | 0.040 | 0.055 |
|  | rs1421085 | T | C | -0.130 | 0.000 | 5.38E+07 | 0.035 | 0.020 | 0.075 |
|  | rs1426371 | G | A | 0.048 | 0.000 | 1.09E+08 | -0.030 | 0.022 | 0.197 |
|  | rs1459513 | A | C | -0.046 | 0.000 | 1.15E+08 | 0.055 | 0.045 | 0.293 |
|  | rs147707338 | C | T | -0.087 | 0.000 | 1.22E+08 | -0.022 | 0.032 | 0.528 |
|  | rs1513275 | T | C | -0.057 | 0.000 | 2.83E+07 | -0.028 | 0.024 | 0.234 |
|  | rs16884229 | T | C | -0.097 | 0.000 | 2.09E+07 | 0.004 | 0.025 | 0.870 |
|  | rs17168486 | C | T | -0.064 | 0.000 | 1.49E+07 | -0.032 | 0.026 | 0.210 |
|  | rs1850421 | A | C | 0.044 | 0.000 | 1.52E+08 | -0.005 | 0.021 | 0.795 |
|  | rs2074120 | A | C | 0.041 | 0.000 | 9.31E+07 | -0.012 | 0.020 | 0.553 |
|  | rs2092518 | G | T | 0.046 | 0.000 | 4.28E+07 | -0.022 | 0.020 | 0.264 |
|  | rs2126736 | A | G | 0.038 | 0.000 | 7.46E+07 | 0.034 | 0.020 | 0.086 |
|  | rs2240885 | G | A | -0.042 | 0.000 | 3.65E+06 | 0.032 | 0.024 | 0.171 |
|  | rs2269245 | G | A | 0.054 | 0.000 | 6.41E+07 | 0.026 | 0.025 | 0.276 |
|  | rs2327777 | T | C | 0.050 | 0.000 | 1.37E+08 | 0.002 | 0.020 | 0.924 |
|  | rs2583934 | G | T | -0.058 | 0.000 | 6.62E+07 | 0.034 | 0.028 | 0.274 |
|  | rs261982 | C | T | -0.040 | 0.000 | 9.58E+07 | -0.016 | 0.020 | 0.430 |
|  | rs2706710 | C | T | -0.071 | 0.000 | 6.56E+07 | -0.003 | 0.026 | 0.974 |
|  | rs2796441 | G | A | 0.075 | 0.000 | 8.43E+07 | -0.017 | 0.020 | 0.419 |
|  | rs28691713 | C | T | 0.066 | 0.000 | 5.04E+07 | -0.027 | 0.020 | 0.174 |
|  | rs2908279 | T | G | -0.046 | 0.000 | 4.42E+07 | -0.007 | 0.020 | 0.736 |
|  | rs2980883 | T | G | 0.042 | 0.000 | 1.26E+08 | 0.007 | 0.028 | 0.801 |
|  | rs3094508 | T | C | -0.059 | 0.000 | 3.61E+07 | -0.007 | 0.020 | 0.722 |
|  | rs329122 | G | A | -0.039 | 0.000 | 1.34E+08 | 0.024 | 0.020 | 0.230 |
|  | rs34811727 | G | T | -0.076 | 0.000 | 1.22E+08 | 0.051 | 0.040 | 0.174 |
|  | rs349359 | A | C | -0.043 | 0.000 | 7.35E+07 | 0.009 | 0.030 | 0.813 |
|  | rs35589574 | C | T | -0.171 | 0.000 | 1.28E+08 | 0.009 | 0.023 | 0.721 |
|  | rs3735641 | A | G | -0.069 | 0.000 | 1.27E+08 | -0.017 | 0.020 | 0.414 |
|  | rs3751236 | G | A | 0.067 | 0.000 | 2.80E+07 | 0.041 | 0.024 | 0.095 |
|  | rs3852529 | C | T | -0.099 | 0.000 | 2.83E+06 | 0.037 | 0.035 | 0.313 |
|  | rs3887925 | C | T | -0.040 | 0.000 | 1.87E+08 | 0.014 | 0.020 | 0.485 |
|  | rs391933 | G | A | 0.037 | 0.000 | 3.36E+07 | -0.009 | 0.020 | 0.652 |
|  | rs4273712 | A | G | -0.047 | 0.000 | 1.27E+08 | -0.060 | 0.022 | 0.006 |
|  | rs4499362 | C | T | 0.044 | 0.000 | 1.50E+08 | 0.019 | 0.025 | 0.445 |
|  | rs476828 | T | C | -0.084 | 0.000 | 5.79E+07 | -0.011 | 0.023 | 0.630 |
|  | rs4930974 | C | T | -0.069 | 0.000 | 3.15E+07 | -0.002 | 0.024 | 0.934 |
|  | rs532504 | G | A | -0.055 | 0.000 | 1.78E+08 | 0.016 | 0.025 | 0.562 |
|  | rs55700915 | G | A | -0.040 | 0.000 | 1.03E+08 | 0.042 | 0.025 | 0.096 |
|  | rs58524310 | A | G | -0.047 | 0.000 | 7.74E+07 | 0.004 | 0.027 | 0.903 |
|  | rs58718028 | C | T | -0.073 | 0.000 | 2.36E+07 | 0.026 | 0.023 | 0.265 |
|  | rs6012878 | G | A | 0.041 | 0.000 | 4.88E+07 | 0.009 | 0.020 | 0.677 |
|  | rs60573766 | C | T | 0.044 | 0.000 | 2.07E+07 | -0.071 | 0.028 | 0.011 |
|  | rs61779313 | T | C | -0.060 | 0.000 | 3.99E+07 | 0.000 | 0.023 | 0.962 |
|  | rs61975988 | A | G | 0.040 | 0.000 | 3.88E+07 | 0.009 | 0.023 | 0.656 |
|  | rs62173901 | A | G | 0.123 | 0.000 | 1.66E+08 | -0.022 | 0.028 | 0.420 |
|  | rs62405419 | G | T | -0.044 | 0.000 | 5.08E+07 | 0.038 | 0.031 | 0.193 |
|  | rs633862 | C | T | -0.039 | 0.000 | 1.36E+08 | -0.011 | 0.020 | 0.554 |
|  | rs6416749 | C | T | 0.052 | 0.000 | 7.31E+07 | -0.007 | 0.021 | 0.738 |
|  | rs6731688 | A | C | -0.098 | 0.000 | 6.31E+05 | -0.019 | 0.026 | 0.497 |
|  | rs703980 | G | A | 0.060 | 0.000 | 8.09E+07 | 0.024 | 0.020 | 0.225 |
|  | rs7107784 | G | A | 0.086 | 0.000 | 2.22E+06 | 0.021 | 0.022 | 0.286 |
|  | rs7109575 | G | A | 0.141 | 0.000 | 7.25E+07 | -0.032 | 0.027 | 0.205 |
|  | rs7250869 | T | C | 0.056 | 0.000 | 3.39E+07 | 0.002 | 0.022 | 0.931 |
|  | rs73085586 | G | A | 0.043 | 0.000 | 2.24E+07 | 0.002 | 0.025 | 0.944 |
|  | rs7313668 | G | T | -0.045 | 0.000 | 7.14E+07 | 0.002 | 0.020 | 0.900 |
|  | rs73347525 | A | G | 0.059 | 0.000 | 1.01E+08 | -0.017 | 0.026 | 0.477 |
|  | rs742762 | A | C | 0.075 | 0.000 | 3.90E+07 | 0.020 | 0.032 | 0.518 |
|  | rs7501939 | T | C | 0.120 | 0.000 | 3.61E+07 | 0.026 | 0.020 | 0.194 |
|  | rs75990271 | T | C | 0.066 | 0.000 | 1.02E+08 | -0.038 | 0.043 | 0.333 |
|  | rs76704029 | T | C | 0.055 | 0.000 | 2.85E+07 | -0.023 | 0.049 | 0.807 |
|  | rs7787720 | C | T | -0.054 | 0.000 | 1.39E+07 | 0.011 | 0.020 | 0.594 |
|  | rs7901695 | T | C | -0.275 | 0.000 | 1.15E+08 | -0.005 | 0.022 | 0.865 |
|  | rs80196932 | T | C | 0.060 | 0.000 | 1.18E+08 | 0.006 | 0.028 | 0.854 |
|  | rs8026714 | G | A | -0.066 | 0.000 | 9.15E+07 | -0.031 | 0.029 | 0.274 |
|  | rs8043085 | G | T | -0.052 | 0.000 | 3.88E+07 | -0.003 | 0.023 | 0.870 |
|  | rs896852 | G | T | 0.042 | 0.000 | 9.60E+07 | 0.000 | 0.020 | 0.999 |
|  | rs9316706 | A | G | 0.041 | 0.000 | 2.26E+07 | -0.042 | 0.026 | 0.114 |
|  | rs9350271 | G | A | -0.193 | 0.000 | 2.07E+07 | 0.034 | 0.021 | 0.102 |
|  | rs9379084 | G | A | 0.072 | 0.000 | 7.23E+06 | 0.002 | 0.032 | 0.983 |
|  | rs9390022 | T | C | 0.048 | 0.000 | 1.43E+08 | 0.041 | 0.021 | 0.049 |
|  | rs9461650 | G | A | 0.060 | 0.000 | 3.11E+07 | 0.022 | 0.027 | 0.364 |
|  | rs9523295 | G | A | 0.078 | 0.000 | 9.19E+07 | 0.009 | 0.023 | 0.691 |
|  | rs9788635 | C | T | 0.058 | 0.000 | 6.21E+07 | 0.011 | 0.031 | 0.593 |
|  | rs9948462 | C | T | -0.047 | 0.000 | 7.08E+06 | 0.004 | 0.020 | 0.882 |
| *Pasteurellaceae* | rs10011838 | G | A | 0.073 | 0.007 | 1.43E-27 | -0.016 | 0.016 | 0.314 |
|  | rs1016565 | G | A | -0.038 | 0.007 | 2.18E-08 | 0.002 | 0.015 | 0.879 |
|  | rs10507349 | G | A | 0.064 | 0.007 | 1.69E-21 | -0.021 | 0.017 | 0.204 |
|  | rs10852123 | A | C | 0.060 | 0.008 | 8.38E-13 | 0.002 | 0.017 | 0.888 |
|  | rs10860209 | C | A | 0.040 | 0.007 | 5.67E-09 | -0.007 | 0.020 | 0.692 |
|  | rs10938398 | G | A | -0.046 | 0.007 | 3.84E-10 | -0.017 | 0.015 | 0.244 |
|  | rs10950550 | T | G | 0.065 | 0.007 | 1.75E-19 | -0.002 | 0.015 | 0.880 |
|  | rs10965248 | T | C | 0.183 | 0.007 | 4.42E-164 | -0.006 | 0.019 | 0.815 |
|  | rs111246699 | G | A | -0.061 | 0.008 | 1.54E-15 | -0.024 | 0.017 | 0.193 |
|  | rs113154802 | C | T | 0.060 | 0.011 | 3.51E-08 | -0.043 | 0.025 | 0.103 |
|  | rs1182444 | A | G | -0.047 | 0.007 | 1.67E-12 | -0.014 | 0.015 | 0.373 |
|  | rs11926494 | G | A | 0.112 | 0.009 | 2.69E-37 | 0.028 | 0.025 | 0.303 |
|  | rs1206684 | G | A | 0.040 | 0.007 | 4.42E-09 | 0.008 | 0.015 | 0.602 |
|  | rs123378 | G | A | 0.053 | 0.008 | 2.22E-10 | 0.023 | 0.016 | 0.148 |
|  | rs1260326 | T | C | -0.063 | 0.007 | 1.01E-21 | -0.014 | 0.015 | 0.316 |
|  | rs12625671 | T | C | -0.066 | 0.007 | 2.25E-21 | -0.021 | 0.021 | 0.187 |
|  | rs12698877 | A | G | -0.067 | 0.007 | 6.96E-22 | 0.003 | 0.016 | 0.857 |
|  | rs12818766 | G | A | -0.054 | 0.009 | 2.46E-10 | 0.004 | 0.020 | 0.833 |
|  | rs13092876 | G | A | -0.126 | 0.007 | 1.91E-66 | -0.014 | 0.016 | 0.374 |
|  | rs13266634 | C | T | 0.116 | 0.007 | 3.72E-67 | 0.014 | 0.016 | 0.370 |
|  | rs1328412 | T | C | 0.097 | 0.015 | 6.41E-11 | 0.021 | 0.029 | 0.427 |
|  | rs1421085 | T | C | -0.130 | 0.009 | 1.55E-48 | 0.016 | 0.015 | 0.282 |
|  | rs1426371 | G | A | 0.048 | 0.007 | 7.76E-12 | 0.003 | 0.017 | 0.798 |
|  | rs1459513 | A | C | -0.046 | 0.007 | 3.73E-11 | 0.015 | 0.032 | 0.475 |
|  | rs147707338 | C | T | -0.087 | 0.014 | 6.47E-10 | -0.053 | 0.025 | 0.039 |
|  | rs1513275 | T | C | -0.057 | 0.009 | 2.76E-11 | -0.033 | 0.017 | 0.037 |
|  | rs16884229 | T | C | -0.097 | 0.007 | 1.15E-43 | 0.000 | 0.018 | 0.878 |
|  | rs17168486 | C | T | -0.064 | 0.007 | 8.23E-22 | -0.007 | 0.019 | 0.736 |
|  | rs1850421 | A | C | 0.044 | 0.007 | 1.41E-09 | 0.016 | 0.016 | 0.331 |
|  | rs2074120 | A | C | 0.041 | 0.007 | 8.38E-09 | -0.013 | 0.015 | 0.369 |
|  | rs2092518 | G | T | 0.046 | 0.007 | 1.39E-10 | -0.013 | 0.015 | 0.388 |
|  | rs2126736 | A | G | 0.038 | 0.007 | 1.84E-08 | 0.036 | 0.015 | 0.014 |
|  | rs2240885 | G | A | -0.042 | 0.007 | 2.79E-09 | 0.009 | 0.018 | 0.595 |
|  | rs2269245 | G | A | 0.054 | 0.009 | 5.40E-10 | -0.007 | 0.018 | 0.674 |
|  | rs2327777 | T | C | 0.050 | 0.007 | 1.06E-13 | 0.028 | 0.015 | 0.057 |
|  | rs2583934 | G | T | -0.058 | 0.007 | 4.95E-16 | 0.011 | 0.020 | 0.500 |
|  | rs261982 | C | T | -0.040 | 0.007 | 3.12E-09 | -0.021 | 0.015 | 0.172 |
|  | rs2706710 | C | T | -0.071 | 0.013 | 1.67E-08 | 0.030 | 0.019 | 0.101 |
|  | rs2796441 | G | A | 0.075 | 0.007 | 1.43E-28 | 0.036 | 0.015 | 0.015 |
|  | rs28599782 | G | A | -0.067 | 0.008 | 4.64E-16 | 0.026 | 0.038 | 0.408 |
|  | rs28691713 | C | T | 0.066 | 0.008 | 1.79E-17 | 0.010 | 0.015 | 0.495 |
|  | rs2908279 | T | G | -0.046 | 0.007 | 8.42E-11 | -0.005 | 0.015 | 0.774 |
|  | rs2980883 | T | G | 0.042 | 0.007 | 3.93E-09 | 0.012 | 0.020 | 0.576 |
|  | rs3094508 | T | C | -0.059 | 0.008 | 1.31E-13 | 0.004 | 0.015 | 0.838 |
|  | rs329122 | G | A | -0.039 | 0.007 | 2.22E-08 | 0.002 | 0.015 | 0.898 |
|  | rs34811727 | G | T | -0.076 | 0.013 | 3.32E-09 | -0.070 | 0.030 | 0.024 |
|  | rs349359 | A | C | -0.043 | 0.008 | 3.05E-08 | -0.008 | 0.022 | 0.644 |
|  | rs35589574 | C | T | -0.171 | 0.011 | 1.58E-55 | 0.022 | 0.017 | 0.204 |
|  | rs3735641 | A | G | -0.069 | 0.008 | 3.62E-19 | 0.029 | 0.015 | 0.051 |
|  | rs3751236 | G | A | 0.067 | 0.007 | 6.58E-21 | 0.002 | 0.018 | 0.866 |
|  | rs3852529 | C | T | -0.099 | 0.010 | 2.07E-24 | 0.038 | 0.026 | 0.150 |
|  | rs3887925 | C | T | -0.040 | 0.007 | 3.12E-09 | 0.006 | 0.015 | 0.697 |
|  | rs391933 | G | A | 0.037 | 0.007 | 1.46E-08 | -0.006 | 0.015 | 0.726 |
|  | rs4273712 | A | G | -0.047 | 0.007 | 2.56E-12 | 0.013 | 0.016 | 0.444 |
|  | rs4499362 | C | T | 0.044 | 0.007 | 1.53E-09 | 0.009 | 0.018 | 0.587 |
|  | rs476828 | T | C | -0.084 | 0.008 | 4.81E-27 | 0.020 | 0.017 | 0.255 |
|  | rs4930974 | C | T | -0.069 | 0.008 | 4.07E-19 | 0.000 | 0.018 | 0.992 |
|  | rs532504 | G | A | -0.055 | 0.008 | 7.39E-12 | 0.008 | 0.018 | 0.670 |
|  | rs55700915 | G | A | -0.040 | 0.007 | 1.50E-08 | -0.016 | 0.018 | 0.372 |
|  | rs58524310 | A | G | -0.047 | 0.007 | 8.41E-11 | 0.018 | 0.020 | 0.413 |
|  | rs58718028 | C | T | -0.073 | 0.007 | 3.35E-25 | 0.053 | 0.017 | 0.002 |
|  | rs6012878 | G | A | 0.041 | 0.007 | 4.32E-09 | 0.019 | 0.015 | 0.190 |
|  | rs60573766 | C | T | 0.044 | 0.007 | 4.30E-10 | -0.029 | 0.021 | 0.194 |
|  | rs61779313 | T | C | -0.060 | 0.009 | 5.59E-11 | -0.020 | 0.017 | 0.266 |
|  | rs61975988 | A | G | 0.040 | 0.007 | 1.97E-09 | -0.006 | 0.017 | 0.714 |
|  | rs62173901 | A | G | 0.123 | 0.020 | 6.58E-10 | -0.005 | 0.022 | 0.838 |
|  | rs62405419 | G | T | -0.044 | 0.008 | 3.79E-09 | -0.020 | 0.022 | 0.440 |
|  | rs633862 | C | T | -0.039 | 0.007 | 1.26E-08 | 0.005 | 0.015 | 0.743 |
|  | rs6416749 | C | T | 0.052 | 0.008 | 3.40E-12 | 0.027 | 0.016 | 0.098 |
|  | rs6731688 | A | C | -0.098 | 0.012 | 2.55E-17 | 0.013 | 0.020 | 0.551 |
|  | rs703980 | G | A | 0.060 | 0.007 | 6.55E-19 | 0.010 | 0.015 | 0.475 |
|  | rs7107784 | G | A | 0.086 | 0.012 | 2.60E-13 | -0.010 | 0.016 | 0.548 |
|  | rs7109575 | G | A | 0.141 | 0.015 | 5.46E-21 | -0.041 | 0.020 | 0.044 |
|  | rs7250869 | T | C | 0.056 | 0.007 | 2.29E-16 | 0.016 | 0.016 | 0.334 |
|  | rs73085586 | G | A | 0.043 | 0.007 | 1.66E-09 | 0.024 | 0.018 | 0.179 |
|  | rs7313668 | G | T | -0.045 | 0.007 | 4.91E-11 | -0.032 | 0.015 | 0.025 |
|  | rs73347525 | A | G | 0.059 | 0.009 | 7.46E-11 | 0.019 | 0.019 | 0.323 |
|  | rs742762 | A | C | 0.075 | 0.008 | 1.79E-22 | -0.050 | 0.023 | 0.012 |
|  | rs7501939 | T | C | 0.120 | 0.007 | 1.60E-60 | 0.039 | 0.015 | 0.008 |
|  | rs75990271 | T | C | 0.066 | 0.010 | 3.22E-11 | -0.046 | 0.033 | 0.181 |
|  | rs76704029 | T | C | 0.055 | 0.010 | 3.39E-08 | 0.012 | 0.034 | 0.531 |
|  | rs7787720 | C | T | -0.054 | 0.007 | 2.25E-15 | -0.016 | 0.015 | 0.284 |
|  | rs7901695 | T | C | -0.275 | 0.017 | 8.18E-62 | 0.006 | 0.016 | 0.685 |
|  | rs80196932 | T | C | 0.060 | 0.008 | 7.57E-13 | 0.016 | 0.020 | 0.394 |
|  | rs8026714 | G | A | -0.066 | 0.007 | 1.06E-22 | 0.049 | 0.021 | 0.035 |
|  | rs8043085 | G | T | -0.052 | 0.007 | 2.06E-14 | -0.005 | 0.017 | 0.689 |
|  | rs896852 | G | T | 0.042 | 0.007 | 6.42E-09 | 0.020 | 0.015 | 0.178 |
|  | rs9316706 | A | G | 0.041 | 0.007 | 3.33E-09 | -0.026 | 0.019 | 0.204 |
|  | rs9350271 | G | A | -0.193 | 0.007 | 4.95E-183 | 0.005 | 0.015 | 0.729 |
|  | rs9379084 | G | A | 0.072 | 0.009 | 2.20E-14 | 0.016 | 0.024 | 0.471 |
|  | rs9390022 | T | C | 0.048 | 0.008 | 6.35E-09 | -0.008 | 0.015 | 0.615 |
|  | rs9461650 | G | A | 0.060 | 0.009 | 1.99E-12 | -0.005 | 0.020 | 0.846 |
|  | rs9523295 | G | A | 0.078 | 0.009 | 7.24E-18 | -0.002 | 0.017 | 0.991 |
|  | rs9788635 | C | T | 0.058 | 0.008 | 9.43E-14 | 0.036 | 0.022 | 0.118 |
|  | rs9948462 | C | T | -0.047 | 0.008 | 8.70E-10 | 0.009 | 0.015 | 0.572 |
| *Peptococcaceae* | rs10011838 | G | A | 0.073 | 0.007 | 1.43E-27 | -0.025 | 0.015 | 0.104 |
|  | rs1016565 | G | A | -0.038 | 0.007 | 2.18E-08 | 0.012 | 0.015 | 0.401 |
|  | rs10507349 | G | A | 0.064 | 0.007 | 1.69E-21 | 0.010 | 0.016 | 0.462 |
|  | rs10852123 | A | C | 0.060 | 0.008 | 8.38E-13 | 0.022 | 0.016 | 0.174 |
|  | rs10860209 | C | A | 0.040 | 0.007 | 5.67E-09 | -0.013 | 0.019 | 0.536 |
|  | rs10938398 | G | A | -0.046 | 0.007 | 3.84E-10 | 0.012 | 0.014 | 0.416 |
|  | rs10950550 | T | G | 0.065 | 0.007 | 1.75E-19 | 0.028 | 0.014 | 0.043 |
|  | rs10965248 | T | C | 0.183 | 0.007 | 4.42E-164 | -0.015 | 0.018 | 0.359 |
|  | rs111246699 | G | A | -0.061 | 0.008 | 1.54E-15 | 0.019 | 0.016 | 0.257 |
|  | rs113154802 | C | T | 0.060 | 0.011 | 3.51E-08 | 0.028 | 0.023 | 0.239 |
|  | rs1182444 | A | G | -0.047 | 0.007 | 1.67E-12 | -0.005 | 0.014 | 0.755 |
|  | rs11926494 | G | A | 0.112 | 0.009 | 2.69E-37 | 0.033 | 0.024 | 0.239 |
|  | rs1206684 | G | A | 0.040 | 0.007 | 4.42E-09 | 0.011 | 0.014 | 0.475 |
|  | rs123378 | G | A | 0.053 | 0.008 | 2.22E-10 | -0.005 | 0.015 | 0.772 |
|  | rs1260326 | T | C | -0.063 | 0.007 | 1.01E-21 | -0.018 | 0.014 | 0.191 |
|  | rs12625671 | T | C | -0.066 | 0.007 | 2.25E-21 | 0.025 | 0.021 | 0.227 |
|  | rs12698877 | A | G | -0.067 | 0.007 | 6.96E-22 | -0.013 | 0.015 | 0.409 |
|  | rs12818766 | G | A | -0.054 | 0.009 | 2.46E-10 | -0.002 | 0.018 | 0.978 |
|  | rs13092876 | G | A | -0.126 | 0.007 | 1.91E-66 | 0.030 | 0.015 | 0.040 |
|  | rs13266634 | C | T | 0.116 | 0.007 | 3.72E-67 | 0.017 | 0.015 | 0.263 |
|  | rs1328412 | T | C | 0.097 | 0.015 | 6.41E-11 | -0.007 | 0.027 | 0.806 |
|  | rs1421085 | T | C | -0.130 | 0.009 | 1.55E-48 | -0.005 | 0.014 | 0.683 |
|  | rs1426371 | G | A | 0.048 | 0.007 | 7.76E-12 | -0.005 | 0.016 | 0.756 |
|  | rs1459513 | A | C | -0.046 | 0.007 | 3.73E-11 | -0.037 | 0.031 | 0.201 |
|  | rs147707338 | C | T | -0.087 | 0.014 | 6.47E-10 | 0.019 | 0.023 | 0.390 |
|  | rs1513275 | T | C | -0.057 | 0.009 | 2.76E-11 | 0.015 | 0.016 | 0.326 |
|  | rs16884229 | T | C | -0.097 | 0.007 | 1.15E-43 | -0.005 | 0.017 | 0.730 |
|  | rs17168486 | C | T | -0.064 | 0.007 | 8.23E-22 | 0.002 | 0.018 | 0.894 |
|  | rs1850421 | A | C | 0.044 | 0.007 | 1.41E-09 | -0.007 | 0.015 | 0.621 |
|  | rs2074120 | A | C | 0.041 | 0.007 | 8.38E-09 | -0.011 | 0.014 | 0.412 |
|  | rs2092518 | G | T | 0.046 | 0.007 | 1.39E-10 | -0.006 | 0.014 | 0.689 |
|  | rs2126736 | A | G | 0.038 | 0.007 | 1.84E-08 | -0.017 | 0.014 | 0.219 |
|  | rs2240885 | G | A | -0.042 | 0.007 | 2.79E-09 | -0.029 | 0.017 | 0.091 |
|  | rs2269245 | G | A | 0.054 | 0.009 | 5.40E-10 | 0.009 | 0.017 | 0.605 |
|  | rs2327777 | T | C | 0.050 | 0.007 | 1.06E-13 | -0.012 | 0.014 | 0.394 |
|  | rs2583934 | G | T | -0.058 | 0.007 | 4.95E-16 | 0.010 | 0.019 | 0.609 |
|  | rs261982 | C | T | -0.040 | 0.007 | 3.12E-09 | -0.005 | 0.014 | 0.754 |
|  | rs2706710 | C | T | -0.071 | 0.013 | 1.67E-08 | -0.015 | 0.018 | 0.355 |
|  | rs2796441 | G | A | 0.075 | 0.007 | 1.43E-28 | 0.011 | 0.014 | 0.451 |
|  | rs28599782 | G | A | -0.067 | 0.008 | 4.64E-16 | -0.056 | 0.044 | 0.211 |
|  | rs28691713 | C | T | 0.066 | 0.008 | 1.79E-17 | -0.018 | 0.014 | 0.198 |
|  | rs2908279 | T | G | -0.046 | 0.007 | 8.42E-11 | -0.001 | 0.014 | 0.928 |
|  | rs2980883 | T | G | 0.042 | 0.007 | 3.93E-09 | -0.045 | 0.019 | 0.020 |
|  | rs3094508 | T | C | -0.059 | 0.008 | 1.31E-13 | -0.029 | 0.014 | 0.039 |
|  | rs329122 | G | A | -0.039 | 0.007 | 2.22E-08 | -0.001 | 0.014 | 0.968 |
|  | rs34811727 | G | T | -0.076 | 0.013 | 3.32E-09 | 0.003 | 0.027 | 0.840 |
|  | rs349359 | A | C | -0.043 | 0.008 | 3.05E-08 | 0.001 | 0.021 | 0.843 |
|  | rs35589574 | C | T | -0.171 | 0.011 | 1.58E-55 | 0.016 | 0.016 | 0.314 |
|  | rs3735641 | A | G | -0.069 | 0.008 | 3.62E-19 | -0.002 | 0.014 | 0.889 |
|  | rs3751236 | G | A | 0.067 | 0.007 | 6.58E-21 | -0.019 | 0.017 | 0.293 |
|  | rs3852529 | C | T | -0.099 | 0.010 | 2.07E-24 | -0.005 | 0.024 | 0.693 |
|  | rs3887925 | C | T | -0.040 | 0.007 | 3.12E-09 | 0.029 | 0.014 | 0.037 |
|  | rs391933 | G | A | 0.037 | 0.007 | 1.46E-08 | 0.006 | 0.014 | 0.671 |
|  | rs4273712 | A | G | -0.047 | 0.007 | 2.56E-12 | -0.036 | 0.015 | 0.017 |
|  | rs4499362 | C | T | 0.044 | 0.007 | 1.53E-09 | 0.002 | 0.017 | 0.845 |
|  | rs476828 | T | C | -0.084 | 0.008 | 4.81E-27 | 0.011 | 0.016 | 0.501 |
|  | rs4930974 | C | T | -0.069 | 0.008 | 4.07E-19 | 0.002 | 0.017 | 0.849 |
|  | rs532504 | G | A | -0.055 | 0.008 | 7.39E-12 | 0.030 | 0.017 | 0.078 |
|  | rs55700915 | G | A | -0.040 | 0.007 | 1.50E-08 | -0.017 | 0.017 | 0.307 |
|  | rs58524310 | A | G | -0.047 | 0.007 | 8.41E-11 | 0.002 | 0.019 | 0.913 |
|  | rs58718028 | C | T | -0.073 | 0.007 | 3.35E-25 | -0.002 | 0.016 | 0.922 |
|  | rs6012878 | G | A | 0.041 | 0.007 | 4.32E-09 | -0.002 | 0.014 | 0.872 |
|  | rs60573766 | C | T | 0.044 | 0.007 | 4.30E-10 | -0.006 | 0.020 | 0.799 |
|  | rs61779313 | T | C | -0.060 | 0.009 | 5.59E-11 | 0.003 | 0.016 | 0.828 |
|  | rs61975988 | A | G | 0.040 | 0.007 | 1.97E-09 | -0.017 | 0.016 | 0.277 |
|  | rs62173901 | A | G | 0.123 | 0.020 | 6.58E-10 | 0.004 | 0.020 | 0.843 |
|  | rs62405419 | G | T | -0.044 | 0.008 | 3.79E-09 | 0.019 | 0.021 | 0.312 |
|  | rs633862 | C | T | -0.039 | 0.007 | 1.26E-08 | 0.022 | 0.014 | 0.109 |
|  | rs6416749 | C | T | 0.052 | 0.008 | 3.40E-12 | -0.003 | 0.015 | 0.815 |
|  | rs6731688 | A | C | -0.098 | 0.012 | 2.55E-17 | 0.019 | 0.018 | 0.308 |
|  | rs703980 | G | A | 0.060 | 0.007 | 6.55E-19 | 0.037 | 0.014 | 0.006 |
|  | rs7107784 | G | A | 0.086 | 0.012 | 2.60E-13 | -0.029 | 0.015 | 0.052 |
|  | rs7109575 | G | A | 0.141 | 0.015 | 5.46E-21 | 0.018 | 0.019 | 0.445 |
|  | rs7250869 | T | C | 0.056 | 0.007 | 2.29E-16 | -0.008 | 0.015 | 0.567 |
|  | rs73085586 | G | A | 0.043 | 0.007 | 1.66E-09 | 0.040 | 0.017 | 0.018 |
|  | rs7313668 | G | T | -0.045 | 0.007 | 4.91E-11 | -0.010 | 0.014 | 0.523 |
|  | rs73347525 | A | G | 0.059 | 0.009 | 7.46E-11 | -0.005 | 0.018 | 0.818 |
|  | rs742762 | A | C | 0.075 | 0.008 | 1.79E-22 | 0.023 | 0.022 | 0.303 |
|  | rs7501939 | T | C | 0.120 | 0.007 | 1.60E-60 | 0.029 | 0.014 | 0.037 |
|  | rs75990271 | T | C | 0.066 | 0.010 | 3.22E-11 | 0.027 | 0.030 | 0.377 |
|  | rs76704029 | T | C | 0.055 | 0.010 | 3.39E-08 | 0.010 | 0.033 | 0.645 |
|  | rs7787720 | C | T | -0.054 | 0.007 | 2.25E-15 | -0.020 | 0.014 | 0.139 |
|  | rs7901695 | T | C | -0.275 | 0.017 | 8.18E-62 | 0.011 | 0.015 | 0.457 |
|  | rs80196932 | T | C | 0.060 | 0.008 | 7.57E-13 | 0.025 | 0.019 | 0.204 |
|  | rs8026714 | G | A | -0.066 | 0.007 | 1.06E-22 | -0.008 | 0.020 | 0.775 |
|  | rs8043085 | G | T | -0.052 | 0.007 | 2.06E-14 | 0.001 | 0.016 | 0.967 |
|  | rs896852 | G | T | 0.042 | 0.007 | 6.42E-09 | -0.009 | 0.014 | 0.523 |
|  | rs9316706 | A | G | 0.041 | 0.007 | 3.33E-09 | 0.016 | 0.018 | 0.434 |
|  | rs9350271 | G | A | -0.193 | 0.007 | 4.95E-183 | 0.004 | 0.015 | 0.748 |
|  | rs9379084 | G | A | 0.072 | 0.009 | 2.20E-14 | 0.013 | 0.022 | 0.646 |
|  | rs9390022 | T | C | 0.048 | 0.008 | 6.35E-09 | -0.003 | 0.014 | 0.839 |
|  | rs9461650 | G | A | 0.060 | 0.009 | 1.99E-12 | 0.000 | 0.019 | 0.941 |
|  | rs9523295 | G | A | 0.078 | 0.009 | 7.24E-18 | -0.006 | 0.016 | 0.647 |
|  | rs9788635 | C | T | 0.058 | 0.008 | 9.43E-14 | -0.011 | 0.021 | 0.674 |
|  | rs9948462 | C | T | -0.047 | 0.008 | 8.70E-10 | 0.015 | 0.014 | 0.277 |
| *Peptostreptococcaceae* | rs10011838 | G | A | 0.073 | 0.007 | 1.43E-27 | -0.026 | 0.012 | 0.031 |
|  | rs1016565 | G | A | -0.038 | 0.007 | 2.18E-08 | 0.021 | 0.012 | 0.087 |
|  | rs10507349 | G | A | 0.064 | 0.007 | 1.69E-21 | 0.016 | 0.013 | 0.226 |
|  | rs10852123 | A | C | 0.060 | 0.008 | 8.38E-13 | 0.002 | 0.013 | 0.867 |
|  | rs10860209 | C | A | 0.040 | 0.007 | 5.67E-09 | 0.007 | 0.015 | 0.626 |
|  | rs10886863 | T | C | -0.060 | 0.007 | 5.28E-17 | -0.059 | 0.037 | 0.110 |
|  | rs10938398 | G | A | -0.046 | 0.007 | 3.84E-10 | 0.002 | 0.011 | 0.829 |
|  | rs10950550 | T | G | 0.065 | 0.007 | 1.75E-19 | 0.025 | 0.011 | 0.024 |
|  | rs10965248 | T | C | 0.183 | 0.007 | 4.42E-164 | -0.016 | 0.014 | 0.282 |
|  | rs111246699 | G | A | -0.061 | 0.008 | 1.54E-15 | 0.026 | 0.013 | 0.049 |
|  | rs113154802 | C | T | 0.060 | 0.011 | 3.51E-08 | -0.002 | 0.019 | 0.921 |
|  | rs1182444 | A | G | -0.047 | 0.007 | 1.67E-12 | 0.008 | 0.012 | 0.526 |
|  | rs11926494 | G | A | 0.112 | 0.009 | 2.69E-37 | 0.017 | 0.019 | 0.366 |
|  | rs1206684 | G | A | 0.040 | 0.007 | 4.42E-09 | -0.008 | 0.012 | 0.518 |
|  | rs123378 | G | A | 0.053 | 0.008 | 2.22E-10 | -0.002 | 0.012 | 0.827 |
|  | rs1260326 | T | C | -0.063 | 0.007 | 1.01E-21 | -0.010 | 0.011 | 0.385 |
|  | rs12610052 | T | C | -0.046 | 0.008 | 1.97E-09 | -0.075 | 0.041 | 0.023 |
|  | rs12625671 | T | C | -0.066 | 0.007 | 2.25E-21 | -0.032 | 0.016 | 0.056 |
|  | rs1266488 | T | C | 0.044 | 0.007 | 6.29E-10 | -0.027 | 0.037 | 0.655 |
|  | rs12698877 | A | G | -0.067 | 0.007 | 6.96E-22 | 0.009 | 0.013 | 0.473 |
|  | rs12818766 | G | A | -0.054 | 0.009 | 2.46E-10 | 0.021 | 0.015 | 0.159 |
|  | rs13092876 | G | A | -0.126 | 0.007 | 1.91E-66 | 0.033 | 0.012 | 0.006 |
|  | rs13266634 | C | T | 0.116 | 0.007 | 3.72E-67 | -0.004 | 0.012 | 0.694 |
|  | rs1328412 | T | C | 0.097 | 0.015 | 6.41E-11 | -0.029 | 0.022 | 0.175 |
|  | rs1421085 | T | C | -0.130 | 0.009 | 1.55E-48 | -0.020 | 0.011 | 0.093 |
|  | rs1426371 | G | A | 0.048 | 0.007 | 7.76E-12 | 0.003 | 0.013 | 0.892 |
|  | rs1459513 | A | C | -0.046 | 0.007 | 3.73E-11 | -0.031 | 0.024 | 0.237 |
|  | rs147707338 | C | T | -0.087 | 0.014 | 6.47E-10 | 0.023 | 0.019 | 0.233 |
|  | rs1513275 | T | C | -0.057 | 0.009 | 2.76E-11 | 0.007 | 0.013 | 0.564 |
|  | rs16884229 | T | C | -0.097 | 0.007 | 1.15E-43 | -0.014 | 0.014 | 0.301 |
|  | rs17168486 | C | T | -0.064 | 0.007 | 8.23E-22 | 0.014 | 0.014 | 0.381 |
|  | rs1850421 | A | C | 0.044 | 0.007 | 1.41E-09 | 0.003 | 0.012 | 0.806 |
|  | rs2074120 | A | C | 0.041 | 0.007 | 8.38E-09 | 0.013 | 0.011 | 0.235 |
|  | rs2092518 | G | T | 0.046 | 0.007 | 1.39E-10 | -0.007 | 0.011 | 0.571 |
|  | rs2126736 | A | G | 0.038 | 0.007 | 1.84E-08 | -0.006 | 0.011 | 0.582 |
|  | rs2240885 | G | A | -0.042 | 0.007 | 2.79E-09 | -0.001 | 0.014 | 0.907 |
|  | rs2269245 | G | A | 0.054 | 0.009 | 5.40E-10 | -0.021 | 0.014 | 0.134 |
|  | rs2327777 | T | C | 0.050 | 0.007 | 1.06E-13 | 0.012 | 0.011 | 0.271 |
|  | rs2583934 | G | T | -0.058 | 0.007 | 4.95E-16 | 0.021 | 0.015 | 0.164 |
|  | rs261982 | C | T | -0.040 | 0.007 | 3.12E-09 | 0.014 | 0.011 | 0.226 |
|  | rs2706710 | C | T | -0.071 | 0.013 | 1.67E-08 | -0.008 | 0.015 | 0.720 |
|  | rs2796441 | G | A | 0.075 | 0.007 | 1.43E-28 | 0.008 | 0.011 | 0.509 |
|  | rs28599782 | G | A | -0.067 | 0.008 | 4.64E-16 | -0.049 | 0.031 | 0.152 |
|  | rs28691713 | C | T | 0.066 | 0.008 | 1.79E-17 | -0.014 | 0.011 | 0.220 |
|  | rs2908279 | T | G | -0.046 | 0.007 | 8.42E-11 | 0.001 | 0.011 | 0.923 |
|  | rs2980883 | T | G | 0.042 | 0.007 | 3.93E-09 | 0.002 | 0.016 | 0.853 |
|  | rs3094508 | T | C | -0.059 | 0.008 | 1.31E-13 | -0.003 | 0.012 | 0.770 |
|  | rs329122 | G | A | -0.039 | 0.007 | 2.22E-08 | 0.009 | 0.011 | 0.422 |
|  | rs34811727 | G | T | -0.076 | 0.013 | 3.32E-09 | 0.004 | 0.023 | 0.877 |
|  | rs349359 | A | C | -0.043 | 0.008 | 3.05E-08 | 0.006 | 0.017 | 0.748 |
|  | rs35589574 | C | T | -0.171 | 0.011 | 1.58E-55 | -0.002 | 0.013 | 0.796 |
|  | rs3735641 | A | G | -0.069 | 0.008 | 3.62E-19 | -0.010 | 0.012 | 0.366 |
|  | rs3751236 | G | A | 0.067 | 0.007 | 6.58E-21 | 0.005 | 0.014 | 0.754 |
|  | rs3852529 | C | T | -0.099 | 0.010 | 2.07E-24 | -0.005 | 0.020 | 0.836 |
|  | rs3887925 | C | T | -0.040 | 0.007 | 3.12E-09 | 0.004 | 0.011 | 0.745 |
|  | rs391933 | G | A | 0.037 | 0.007 | 1.46E-08 | 0.009 | 0.011 | 0.411 |
|  | rs4273712 | A | G | -0.047 | 0.007 | 2.56E-12 | 0.013 | 0.012 | 0.291 |
|  | rs4499362 | C | T | 0.044 | 0.007 | 1.53E-09 | -0.042 | 0.014 | 0.003 |
|  | rs476828 | T | C | -0.084 | 0.008 | 4.81E-27 | -0.013 | 0.013 | 0.323 |
|  | rs4930974 | C | T | -0.069 | 0.008 | 4.07E-19 | 0.004 | 0.014 | 0.688 |
|  | rs532504 | G | A | -0.055 | 0.008 | 7.39E-12 | -0.008 | 0.014 | 0.558 |
|  | rs55700915 | G | A | -0.040 | 0.007 | 1.50E-08 | -0.004 | 0.014 | 0.826 |
|  | rs58524310 | A | G | -0.047 | 0.007 | 8.41E-11 | -0.004 | 0.016 | 0.689 |
|  | rs58718028 | C | T | -0.073 | 0.007 | 3.35E-25 | 0.016 | 0.013 | 0.216 |
|  | rs6012878 | G | A | 0.041 | 0.007 | 4.32E-09 | -0.001 | 0.011 | 0.936 |
|  | rs60573766 | C | T | 0.044 | 0.007 | 4.30E-10 | 0.013 | 0.016 | 0.341 |
|  | rs61779313 | T | C | -0.060 | 0.009 | 5.59E-11 | -0.034 | 0.013 | 0.007 |
|  | rs61975988 | A | G | 0.040 | 0.007 | 1.97E-09 | -0.017 | 0.013 | 0.181 |
|  | rs62173901 | A | G | 0.123 | 0.020 | 6.58E-10 | -0.024 | 0.016 | 0.104 |
|  | rs62405419 | G | T | -0.044 | 0.008 | 3.79E-09 | -0.010 | 0.017 | 0.641 |
|  | rs633862 | C | T | -0.039 | 0.007 | 1.26E-08 | 0.000 | 0.011 | 0.956 |
|  | rs6416749 | C | T | 0.052 | 0.008 | 3.40E-12 | 0.014 | 0.012 | 0.243 |
|  | rs6731688 | A | C | -0.098 | 0.012 | 2.55E-17 | -0.008 | 0.015 | 0.576 |
|  | rs703980 | G | A | 0.060 | 0.007 | 6.55E-19 | -0.003 | 0.011 | 0.811 |
|  | rs7107784 | G | A | 0.086 | 0.012 | 2.60E-13 | 0.007 | 0.013 | 0.518 |
|  | rs7109575 | G | A | 0.141 | 0.015 | 5.46E-21 | -0.006 | 0.015 | 0.720 |
|  | rs7250869 | T | C | 0.056 | 0.007 | 2.29E-16 | 0.020 | 0.012 | 0.099 |
|  | rs73085586 | G | A | 0.043 | 0.007 | 1.66E-09 | 0.018 | 0.014 | 0.179 |
|  | rs7313668 | G | T | -0.045 | 0.007 | 4.91E-11 | 0.013 | 0.011 | 0.261 |
|  | rs73347525 | A | G | 0.059 | 0.009 | 7.46E-11 | -0.019 | 0.015 | 0.196 |
|  | rs742762 | A | C | 0.075 | 0.008 | 1.79E-22 | -0.011 | 0.018 | 0.487 |
|  | rs7501939 | T | C | 0.120 | 0.007 | 1.60E-60 | 0.010 | 0.011 | 0.354 |
|  | rs75990271 | T | C | 0.066 | 0.010 | 3.22E-11 | -0.024 | 0.025 | 0.493 |
|  | rs7656416 | C | T | 0.100 | 0.007 | 9.01E-42 | 0.104 | 0.034 | 0.007 |
|  | rs76704029 | T | C | 0.055 | 0.010 | 3.39E-08 | 0.044 | 0.026 | 0.072 |
|  | rs76878791 | A | G | -0.053 | 0.007 | 2.02E-13 | 0.051 | 0.050 | 0.291 |
|  | rs77789961 | C | T | -0.047 | 0.008 | 1.92E-08 | 0.043 | 0.044 | 0.557 |
|  | rs7787720 | C | T | -0.054 | 0.007 | 2.25E-15 | -0.016 | 0.011 | 0.154 |
|  | rs7901695 | T | C | -0.275 | 0.017 | 8.18E-62 | -0.016 | 0.012 | 0.212 |
|  | rs80196932 | T | C | 0.060 | 0.008 | 7.57E-13 | 0.005 | 0.016 | 0.699 |
|  | rs8026714 | G | A | -0.066 | 0.007 | 1.06E-22 | -0.011 | 0.016 | 0.479 |
|  | rs8043085 | G | T | -0.052 | 0.007 | 2.06E-14 | 0.001 | 0.013 | 0.977 |
|  | rs896852 | G | T | 0.042 | 0.007 | 6.42E-09 | -0.011 | 0.011 | 0.339 |
|  | rs9316706 | A | G | 0.041 | 0.007 | 3.33E-09 | 0.013 | 0.015 | 0.384 |
|  | rs9350271 | G | A | -0.193 | 0.007 | 4.95E-183 | 0.010 | 0.012 | 0.397 |
|  | rs9379084 | G | A | 0.072 | 0.009 | 2.20E-14 | -0.024 | 0.018 | 0.175 |
|  | rs9390022 | T | C | 0.048 | 0.008 | 6.35E-09 | -0.003 | 0.012 | 0.775 |
|  | rs9461650 | G | A | 0.060 | 0.009 | 1.99E-12 | -0.034 | 0.015 | 0.031 |
|  | rs9523295 | G | A | 0.078 | 0.009 | 7.24E-18 | 0.012 | 0.013 | 0.355 |
|  | rs9788635 | C | T | 0.058 | 0.008 | 9.43E-14 | 0.036 | 0.017 | 0.048 |
|  | rs9948462 | C | T | -0.047 | 0.008 | 8.70E-10 | 0.004 | 0.012 | 0.743 |
| *Porphyromonadaceae* | rs10011838 | G | A | 0.073 | 0.007 | 1.43E-27 | 0.006 | 0.012 | 0.589 |
|  | rs1016565 | G | A | -0.038 | 0.007 | 2.18E-08 | 0.001 | 0.011 | 0.956 |
|  | rs10507349 | G | A | 0.064 | 0.007 | 1.69E-21 | -0.004 | 0.012 | 0.750 |
|  | rs10852123 | A | C | 0.060 | 0.008 | 8.38E-13 | 0.010 | 0.012 | 0.378 |
|  | rs10860209 | C | A | 0.040 | 0.007 | 5.67E-09 | 0.010 | 0.014 | 0.445 |
|  | rs10886863 | T | C | -0.060 | 0.007 | 5.28E-17 | 0.033 | 0.033 | 0.292 |
|  | rs10938398 | G | A | -0.046 | 0.007 | 3.84E-10 | 0.009 | 0.011 | 0.376 |
|  | rs10950550 | T | G | 0.065 | 0.007 | 1.75E-19 | -0.005 | 0.011 | 0.648 |
|  | rs10965248 | T | C | 0.183 | 0.007 | 4.42E-164 | 0.009 | 0.014 | 0.573 |
|  | rs111246699 | G | A | -0.061 | 0.008 | 1.54E-15 | -0.018 | 0.013 | 0.144 |
|  | rs113154802 | C | T | 0.060 | 0.011 | 3.51E-08 | 0.005 | 0.018 | 0.833 |
|  | rs1182444 | A | G | -0.047 | 0.007 | 1.67E-12 | -0.020 | 0.011 | 0.059 |
|  | rs11926494 | G | A | 0.112 | 0.009 | 2.69E-37 | -0.030 | 0.018 | 0.112 |
|  | rs1206684 | G | A | 0.040 | 0.007 | 4.42E-09 | -0.011 | 0.011 | 0.369 |
|  | rs123378 | G | A | 0.053 | 0.008 | 2.22E-10 | -0.008 | 0.011 | 0.474 |
|  | rs1260326 | T | C | -0.063 | 0.007 | 1.01E-21 | -0.009 | 0.011 | 0.361 |
|  | rs12610052 | T | C | -0.046 | 0.008 | 1.97E-09 | -0.040 | 0.039 | 0.306 |
|  | rs12625671 | T | C | -0.066 | 0.007 | 2.25E-21 | 0.021 | 0.015 | 0.147 |
|  | rs1266488 | T | C | 0.044 | 0.007 | 6.29E-10 | 0.027 | 0.033 | 0.697 |
|  | rs12698877 | A | G | -0.067 | 0.007 | 6.96E-22 | 0.020 | 0.012 | 0.091 |
|  | rs12818766 | G | A | -0.054 | 0.009 | 2.46E-10 | -0.021 | 0.014 | 0.128 |
|  | rs13092876 | G | A | -0.126 | 0.007 | 1.91E-66 | -0.007 | 0.011 | 0.554 |
|  | rs13266634 | C | T | 0.116 | 0.007 | 3.72E-67 | -0.008 | 0.011 | 0.525 |
|  | rs1328412 | T | C | 0.097 | 0.015 | 6.41E-11 | 0.045 | 0.021 | 0.039 |
|  | rs1421085 | T | C | -0.130 | 0.009 | 1.55E-48 | 0.000 | 0.011 | 0.942 |
|  | rs1426371 | G | A | 0.048 | 0.007 | 7.76E-12 | -0.026 | 0.012 | 0.036 |
|  | rs1459513 | A | C | -0.046 | 0.007 | 3.73E-11 | 0.009 | 0.023 | 0.724 |
|  | rs147707338 | C | T | -0.087 | 0.014 | 6.47E-10 | 0.018 | 0.018 | 0.359 |
|  | rs1513275 | T | C | -0.057 | 0.009 | 2.76E-11 | -0.011 | 0.012 | 0.399 |
|  | rs16884229 | T | C | -0.097 | 0.007 | 1.15E-43 | 0.005 | 0.013 | 0.597 |
|  | rs17168486 | C | T | -0.064 | 0.007 | 8.23E-22 | -0.022 | 0.014 | 0.115 |
|  | rs1850421 | A | C | 0.044 | 0.007 | 1.41E-09 | 0.008 | 0.011 | 0.478 |
|  | rs2074120 | A | C | 0.041 | 0.007 | 8.38E-09 | -0.011 | 0.011 | 0.311 |
|  | rs2092518 | G | T | 0.046 | 0.007 | 1.39E-10 | 0.007 | 0.011 | 0.517 |
|  | rs2126736 | A | G | 0.038 | 0.007 | 1.84E-08 | -0.003 | 0.011 | 0.765 |
|  | rs2240885 | G | A | -0.042 | 0.007 | 2.79E-09 | 0.014 | 0.013 | 0.280 |
|  | rs2269245 | G | A | 0.054 | 0.009 | 5.40E-10 | -0.013 | 0.013 | 0.374 |
|  | rs2327777 | T | C | 0.050 | 0.007 | 1.06E-13 | 0.004 | 0.011 | 0.695 |
|  | rs2583934 | G | T | -0.058 | 0.007 | 4.95E-16 | 0.004 | 0.014 | 0.779 |
|  | rs261982 | C | T | -0.040 | 0.007 | 3.12E-09 | -0.008 | 0.011 | 0.445 |
|  | rs2706710 | C | T | -0.071 | 0.013 | 1.67E-08 | 0.001 | 0.014 | 0.972 |
|  | rs2796441 | G | A | 0.075 | 0.007 | 1.43E-28 | -0.006 | 0.011 | 0.566 |
|  | rs28599782 | G | A | -0.067 | 0.008 | 4.64E-16 | -0.028 | 0.029 | 0.355 |
|  | rs28691713 | C | T | 0.066 | 0.008 | 1.79E-17 | 0.009 | 0.011 | 0.425 |
|  | rs2908279 | T | G | -0.046 | 0.007 | 8.42E-11 | -0.002 | 0.011 | 0.827 |
|  | rs2980883 | T | G | 0.042 | 0.007 | 3.93E-09 | 0.026 | 0.015 | 0.078 |
|  | rs3094508 | T | C | -0.059 | 0.008 | 1.31E-13 | 0.010 | 0.011 | 0.373 |
|  | rs329122 | G | A | -0.039 | 0.007 | 2.22E-08 | 0.009 | 0.011 | 0.397 |
|  | rs34811727 | G | T | -0.076 | 0.013 | 3.32E-09 | -0.019 | 0.022 | 0.376 |
|  | rs349359 | A | C | -0.043 | 0.008 | 3.05E-08 | 0.025 | 0.016 | 0.117 |
|  | rs35589574 | C | T | -0.171 | 0.011 | 1.58E-55 | -0.001 | 0.012 | 0.928 |
|  | rs3735641 | A | G | -0.069 | 0.008 | 3.62E-19 | 0.010 | 0.011 | 0.369 |
|  | rs3751236 | G | A | 0.067 | 0.007 | 6.58E-21 | -0.001 | 0.013 | 0.928 |
|  | rs3852529 | C | T | -0.099 | 0.010 | 2.07E-24 | 0.000 | 0.019 | 0.971 |
|  | rs3887925 | C | T | -0.040 | 0.007 | 3.12E-09 | 0.026 | 0.011 | 0.015 |
|  | rs391933 | G | A | 0.037 | 0.007 | 1.46E-08 | 0.003 | 0.011 | 0.828 |
|  | rs4273712 | A | G | -0.047 | 0.007 | 2.56E-12 | -0.015 | 0.012 | 0.213 |
|  | rs4499362 | C | T | 0.044 | 0.007 | 1.53E-09 | 0.024 | 0.013 | 0.057 |
|  | rs476828 | T | C | -0.084 | 0.008 | 4.81E-27 | -0.009 | 0.012 | 0.411 |
|  | rs4930974 | C | T | -0.069 | 0.008 | 4.07E-19 | -0.008 | 0.013 | 0.523 |
|  | rs532504 | G | A | -0.055 | 0.008 | 7.39E-12 | -0.013 | 0.013 | 0.317 |
|  | rs55700915 | G | A | -0.040 | 0.007 | 1.50E-08 | -0.028 | 0.013 | 0.035 |
|  | rs58524310 | A | G | -0.047 | 0.007 | 8.41E-11 | 0.033 | 0.015 | 0.024 |
|  | rs58718028 | C | T | -0.073 | 0.007 | 3.35E-25 | 0.001 | 0.012 | 0.953 |
|  | rs6012878 | G | A | 0.041 | 0.007 | 4.32E-09 | 0.007 | 0.011 | 0.533 |
|  | rs60573766 | C | T | 0.044 | 0.007 | 4.30E-10 | 0.015 | 0.015 | 0.285 |
|  | rs61779313 | T | C | -0.060 | 0.009 | 5.59E-11 | 0.002 | 0.013 | 0.907 |
|  | rs61975988 | A | G | 0.040 | 0.007 | 1.97E-09 | -0.009 | 0.012 | 0.405 |
|  | rs62173901 | A | G | 0.123 | 0.020 | 6.58E-10 | -0.014 | 0.016 | 0.354 |
|  | rs62405419 | G | T | -0.044 | 0.008 | 3.79E-09 | -0.003 | 0.016 | 0.843 |
|  | rs633862 | C | T | -0.039 | 0.007 | 1.26E-08 | 0.022 | 0.011 | 0.036 |
|  | rs6416749 | C | T | 0.052 | 0.008 | 3.40E-12 | 0.006 | 0.011 | 0.646 |
|  | rs6731688 | A | C | -0.098 | 0.012 | 2.55E-17 | -0.018 | 0.014 | 0.204 |
|  | rs703980 | G | A | 0.060 | 0.007 | 6.55E-19 | -0.002 | 0.011 | 0.821 |
|  | rs7107784 | G | A | 0.086 | 0.012 | 2.60E-13 | -0.001 | 0.012 | 0.872 |
|  | rs7109575 | G | A | 0.141 | 0.015 | 5.46E-21 | -0.006 | 0.015 | 0.761 |
|  | rs7250869 | T | C | 0.056 | 0.007 | 2.29E-16 | -0.004 | 0.011 | 0.691 |
|  | rs7304270 | C | T | 0.068 | 0.010 | 1.04E-12 | 0.064 | 0.057 | 0.258 |
|  | rs73085586 | G | A | 0.043 | 0.007 | 1.66E-09 | 0.007 | 0.013 | 0.595 |
|  | rs7313668 | G | T | -0.045 | 0.007 | 4.91E-11 | -0.007 | 0.011 | 0.510 |
|  | rs73347525 | A | G | 0.059 | 0.009 | 7.46E-11 | -0.011 | 0.014 | 0.406 |
|  | rs742762 | A | C | 0.075 | 0.008 | 1.79E-22 | -0.019 | 0.017 | 0.248 |
|  | rs7501939 | T | C | 0.120 | 0.007 | 1.60E-60 | 0.011 | 0.011 | 0.337 |
|  | rs75990271 | T | C | 0.066 | 0.010 | 3.22E-11 | 0.011 | 0.024 | 0.824 |
|  | rs7656416 | C | T | 0.100 | 0.007 | 9.01E-42 | -0.044 | 0.031 | 0.081 |
|  | rs76704029 | T | C | 0.055 | 0.010 | 3.39E-08 | -0.044 | 0.024 | 0.096 |
|  | rs76878791 | A | G | -0.053 | 0.007 | 2.02E-13 | -0.009 | 0.046 | 0.785 |
|  | rs77789961 | C | T | -0.047 | 0.008 | 1.92E-08 | -0.026 | 0.039 | 0.520 |
|  | rs7787720 | C | T | -0.054 | 0.007 | 2.25E-15 | 0.002 | 0.011 | 0.821 |
|  | rs7901695 | T | C | -0.275 | 0.017 | 8.18E-62 | 0.006 | 0.012 | 0.586 |
|  | rs80196932 | T | C | 0.060 | 0.008 | 7.57E-13 | -0.016 | 0.015 | 0.285 |
|  | rs8026714 | G | A | -0.066 | 0.007 | 1.06E-22 | -0.008 | 0.015 | 0.600 |
|  | rs8043085 | G | T | -0.052 | 0.007 | 2.06E-14 | 0.004 | 0.012 | 0.751 |
|  | rs896852 | G | T | 0.042 | 0.007 | 6.42E-09 | 0.007 | 0.011 | 0.515 |
|  | rs9316706 | A | G | 0.041 | 0.007 | 3.33E-09 | 0.016 | 0.014 | 0.235 |
|  | rs9350271 | G | A | -0.193 | 0.007 | 4.95E-183 | 0.006 | 0.011 | 0.611 |
|  | rs9379084 | G | A | 0.072 | 0.009 | 2.20E-14 | 0.003 | 0.017 | 0.945 |
|  | rs9390022 | T | C | 0.048 | 0.008 | 6.35E-09 | 0.003 | 0.011 | 0.782 |
|  | rs9461650 | G | A | 0.060 | 0.009 | 1.99E-12 | -0.001 | 0.015 | 0.942 |
|  | rs9523295 | G | A | 0.078 | 0.009 | 7.24E-18 | -0.013 | 0.012 | 0.291 |
|  | rs9788635 | C | T | 0.058 | 0.008 | 9.43E-14 | 0.005 | 0.016 | 0.737 |
|  | rs9948462 | C | T | -0.047 | 0.008 | 8.70E-10 | -0.002 | 0.011 | 0.837 |
| *Prevotellaceae* | rs10011838 | G | A | 0.073 | 0.007 | 1.43E-27 | -0.011 | 0.013 | 0.389 |
|  | rs1016565 | G | A | -0.038 | 0.007 | 2.18E-08 | -0.005 | 0.012 | 0.669 |
|  | rs10507349 | G | A | 0.064 | 0.007 | 1.69E-21 | 0.002 | 0.013 | 0.807 |
|  | rs10852123 | A | C | 0.060 | 0.008 | 8.38E-13 | -0.005 | 0.014 | 0.731 |
|  | rs10860209 | C | A | 0.040 | 0.007 | 5.67E-09 | -0.002 | 0.016 | 0.961 |
|  | rs10886863 | T | C | -0.060 | 0.007 | 5.28E-17 | -0.007 | 0.034 | 0.994 |
|  | rs10938398 | G | A | -0.046 | 0.007 | 3.84E-10 | 0.012 | 0.012 | 0.312 |
|  | rs10950550 | T | G | 0.065 | 0.007 | 1.75E-19 | -0.012 | 0.012 | 0.281 |
|  | rs10965248 | T | C | 0.183 | 0.007 | 4.42E-164 | 0.002 | 0.015 | 0.839 |
|  | rs111246699 | G | A | -0.061 | 0.008 | 1.54E-15 | -0.016 | 0.014 | 0.224 |
|  | rs113154802 | C | T | 0.060 | 0.011 | 3.51E-08 | 0.023 | 0.020 | 0.219 |
|  | rs1182444 | A | G | -0.047 | 0.007 | 1.67E-12 | 0.003 | 0.012 | 0.818 |
|  | rs11926494 | G | A | 0.112 | 0.009 | 2.69E-37 | 0.017 | 0.020 | 0.439 |
|  | rs1206684 | G | A | 0.040 | 0.007 | 4.42E-09 | 0.009 | 0.012 | 0.467 |
|  | rs123378 | G | A | 0.053 | 0.008 | 2.22E-10 | 0.008 | 0.013 | 0.514 |
|  | rs1260326 | T | C | -0.063 | 0.007 | 1.01E-21 | 0.011 | 0.012 | 0.363 |
|  | rs12610052 | T | C | -0.046 | 0.008 | 1.97E-09 | -0.030 | 0.041 | 0.357 |
|  | rs12625671 | T | C | -0.066 | 0.007 | 2.25E-21 | 0.030 | 0.017 | 0.186 |
|  | rs1266488 | T | C | 0.044 | 0.007 | 6.29E-10 | -0.020 | 0.034 | 0.540 |
|  | rs12698877 | A | G | -0.067 | 0.007 | 6.96E-22 | -0.002 | 0.013 | 0.852 |
|  | rs12818766 | G | A | -0.054 | 0.009 | 2.46E-10 | 0.003 | 0.016 | 0.854 |
|  | rs13092876 | G | A | -0.126 | 0.007 | 1.91E-66 | 0.011 | 0.012 | 0.400 |
|  | rs13266634 | C | T | 0.116 | 0.007 | 3.72E-67 | -0.007 | 0.013 | 0.586 |
|  | rs1328412 | T | C | 0.097 | 0.015 | 6.41E-11 | -0.013 | 0.023 | 0.505 |
|  | rs1421085 | T | C | -0.130 | 0.009 | 1.55E-48 | 0.015 | 0.012 | 0.223 |
|  | rs1426371 | G | A | 0.048 | 0.007 | 7.76E-12 | 0.012 | 0.013 | 0.391 |
|  | rs1459513 | A | C | -0.046 | 0.007 | 3.73E-11 | 0.020 | 0.025 | 0.386 |
|  | rs147707338 | C | T | -0.087 | 0.014 | 6.47E-10 | 0.006 | 0.020 | 0.758 |
|  | rs1513275 | T | C | -0.057 | 0.009 | 2.76E-11 | -0.003 | 0.014 | 0.879 |
|  | rs16884229 | T | C | -0.097 | 0.007 | 1.15E-43 | -0.015 | 0.015 | 0.307 |
|  | rs17168486 | C | T | -0.064 | 0.007 | 8.23E-22 | 0.013 | 0.015 | 0.438 |
|  | rs1850421 | A | C | 0.044 | 0.007 | 1.41E-09 | -0.002 | 0.013 | 0.850 |
|  | rs2074120 | A | C | 0.041 | 0.007 | 8.38E-09 | 0.011 | 0.012 | 0.342 |
|  | rs2092518 | G | T | 0.046 | 0.007 | 1.39E-10 | -0.022 | 0.012 | 0.059 |
|  | rs2126736 | A | G | 0.038 | 0.007 | 1.84E-08 | -0.007 | 0.012 | 0.566 |
|  | rs2240885 | G | A | -0.042 | 0.007 | 2.79E-09 | -0.003 | 0.014 | 0.874 |
|  | rs2269245 | G | A | 0.054 | 0.009 | 5.40E-10 | 0.017 | 0.014 | 0.246 |
|  | rs2327777 | T | C | 0.050 | 0.007 | 1.06E-13 | -0.003 | 0.012 | 0.807 |
|  | rs2583934 | G | T | -0.058 | 0.007 | 4.95E-16 | -0.007 | 0.016 | 0.660 |
|  | rs261982 | C | T | -0.040 | 0.007 | 3.12E-09 | -0.013 | 0.012 | 0.277 |
|  | rs2706710 | C | T | -0.071 | 0.013 | 1.67E-08 | 0.020 | 0.015 | 0.175 |
|  | rs2796441 | G | A | 0.075 | 0.007 | 1.43E-28 | 0.006 | 0.012 | 0.591 |
|  | rs28599782 | G | A | -0.067 | 0.008 | 4.64E-16 | 0.011 | 0.031 | 0.750 |
|  | rs28691713 | C | T | 0.066 | 0.008 | 1.79E-17 | -0.002 | 0.012 | 0.836 |
|  | rs2908279 | T | G | -0.046 | 0.007 | 8.42E-11 | 0.000 | 0.012 | 0.966 |
|  | rs2980883 | T | G | 0.042 | 0.007 | 3.93E-09 | -0.007 | 0.016 | 0.658 |
|  | rs3094508 | T | C | -0.059 | 0.008 | 1.31E-13 | 0.004 | 0.012 | 0.748 |
|  | rs329122 | G | A | -0.039 | 0.007 | 2.22E-08 | -0.023 | 0.012 | 0.048 |
|  | rs34811727 | G | T | -0.076 | 0.013 | 3.32E-09 | 0.008 | 0.024 | 0.823 |
|  | rs349359 | A | C | -0.043 | 0.008 | 3.05E-08 | 0.040 | 0.018 | 0.031 |
|  | rs35589574 | C | T | -0.171 | 0.011 | 1.58E-55 | 0.006 | 0.014 | 0.780 |
|  | rs3735641 | A | G | -0.069 | 0.008 | 3.62E-19 | 0.016 | 0.012 | 0.183 |
|  | rs3751236 | G | A | 0.067 | 0.007 | 6.58E-21 | -0.007 | 0.014 | 0.609 |
|  | rs3852529 | C | T | -0.099 | 0.010 | 2.07E-24 | 0.001 | 0.020 | 0.925 |
|  | rs3887925 | C | T | -0.040 | 0.007 | 3.12E-09 | 0.006 | 0.012 | 0.579 |
|  | rs391933 | G | A | 0.037 | 0.007 | 1.46E-08 | -0.018 | 0.012 | 0.133 |
|  | rs4273712 | A | G | -0.047 | 0.007 | 2.56E-12 | -0.008 | 0.013 | 0.591 |
|  | rs4499362 | C | T | 0.044 | 0.007 | 1.53E-09 | -0.007 | 0.015 | 0.730 |
|  | rs476828 | T | C | -0.084 | 0.008 | 4.81E-27 | 0.007 | 0.014 | 0.663 |
|  | rs4930974 | C | T | -0.069 | 0.008 | 4.07E-19 | -0.027 | 0.014 | 0.061 |
|  | rs532504 | G | A | -0.055 | 0.008 | 7.39E-12 | -0.013 | 0.015 | 0.390 |
|  | rs55700915 | G | A | -0.040 | 0.007 | 1.50E-08 | -0.019 | 0.014 | 0.215 |
|  | rs58524310 | A | G | -0.047 | 0.007 | 8.41E-11 | 0.027 | 0.016 | 0.092 |
|  | rs58718028 | C | T | -0.073 | 0.007 | 3.35E-25 | 0.034 | 0.013 | 0.012 |
|  | rs6012878 | G | A | 0.041 | 0.007 | 4.32E-09 | 0.017 | 0.012 | 0.163 |
|  | rs60573766 | C | T | 0.044 | 0.007 | 4.30E-10 | -0.021 | 0.017 | 0.206 |
|  | rs61779313 | T | C | -0.060 | 0.009 | 5.59E-11 | -0.009 | 0.014 | 0.552 |
|  | rs61975988 | A | G | 0.040 | 0.007 | 1.97E-09 | -0.007 | 0.013 | 0.590 |
|  | rs62173901 | A | G | 0.123 | 0.020 | 6.58E-10 | -0.043 | 0.017 | 0.009 |
|  | rs62405419 | G | T | -0.044 | 0.008 | 3.79E-09 | 0.015 | 0.018 | 0.463 |
|  | rs633862 | C | T | -0.039 | 0.007 | 1.26E-08 | -0.008 | 0.012 | 0.502 |
|  | rs6416749 | C | T | 0.052 | 0.008 | 3.40E-12 | 0.008 | 0.013 | 0.529 |
|  | rs6731688 | A | C | -0.098 | 0.012 | 2.55E-17 | -0.002 | 0.016 | 0.906 |
|  | rs703980 | G | A | 0.060 | 0.007 | 6.55E-19 | 0.008 | 0.012 | 0.463 |
|  | rs7107784 | G | A | 0.086 | 0.012 | 2.60E-13 | -0.015 | 0.013 | 0.234 |
|  | rs7109575 | G | A | 0.141 | 0.015 | 5.46E-21 | -0.007 | 0.016 | 0.606 |
|  | rs7250869 | T | C | 0.056 | 0.007 | 2.29E-16 | -0.005 | 0.013 | 0.703 |
|  | rs73085586 | G | A | 0.043 | 0.007 | 1.66E-09 | -0.022 | 0.014 | 0.142 |
|  | rs7313668 | G | T | -0.045 | 0.007 | 4.91E-11 | -0.001 | 0.012 | 0.937 |
|  | rs73347525 | A | G | 0.059 | 0.009 | 7.46E-11 | -0.012 | 0.015 | 0.454 |
|  | rs742762 | A | C | 0.075 | 0.008 | 1.79E-22 | 0.001 | 0.018 | 0.984 |
|  | rs7501939 | T | C | 0.120 | 0.007 | 1.60E-60 | -0.011 | 0.012 | 0.329 |
|  | rs75990271 | T | C | 0.066 | 0.010 | 3.22E-11 | 0.007 | 0.026 | 0.759 |
|  | rs7656416 | C | T | 0.100 | 0.007 | 9.01E-42 | -0.040 | 0.032 | 0.359 |
|  | rs76704029 | T | C | 0.055 | 0.010 | 3.39E-08 | 0.039 | 0.026 | 0.176 |
|  | rs76878791 | A | G | -0.053 | 0.007 | 2.02E-13 | 0.029 | 0.048 | 0.483 |
|  | rs77789961 | C | T | -0.047 | 0.008 | 1.92E-08 | 0.072 | 0.040 | 0.042 |
|  | rs7787720 | C | T | -0.054 | 0.007 | 2.25E-15 | -0.018 | 0.012 | 0.135 |
|  | rs7901695 | T | C | -0.275 | 0.017 | 8.18E-62 | 0.002 | 0.013 | 0.909 |
|  | rs80196932 | T | C | 0.060 | 0.008 | 7.57E-13 | 0.006 | 0.016 | 0.762 |
|  | rs8026714 | G | A | -0.066 | 0.007 | 1.06E-22 | -0.039 | 0.017 | 0.023 |
|  | rs8043085 | G | T | -0.052 | 0.007 | 2.06E-14 | -0.009 | 0.013 | 0.511 |
|  | rs896852 | G | T | 0.042 | 0.007 | 6.42E-09 | 0.009 | 0.012 | 0.459 |
|  | rs9316706 | A | G | 0.041 | 0.007 | 3.33E-09 | 0.018 | 0.015 | 0.231 |
|  | rs9350271 | G | A | -0.193 | 0.007 | 4.95E-183 | -0.003 | 0.012 | 0.873 |
|  | rs9379084 | G | A | 0.072 | 0.009 | 2.20E-14 | -0.012 | 0.019 | 0.664 |
|  | rs9390022 | T | C | 0.048 | 0.008 | 6.35E-09 | 0.014 | 0.012 | 0.250 |
|  | rs9461650 | G | A | 0.060 | 0.009 | 1.99E-12 | 0.001 | 0.016 | 0.978 |
|  | rs9523295 | G | A | 0.078 | 0.009 | 7.24E-18 | 0.009 | 0.014 | 0.511 |
|  | rs9788635 | C | T | 0.058 | 0.008 | 9.43E-14 | 0.003 | 0.018 | 0.862 |
|  | rs9948462 | C | T | -0.047 | 0.008 | 8.70E-10 | -0.006 | 0.012 | 0.618 |
| *Rhodospirillaceae* | rs10011838 | G | A | 0.073 | 0.007 | 1.43E-27 | 0.025 | 0.017 | 0.151 |
|  | rs1016565 | G | A | -0.038 | 0.007 | 2.18E-08 | -0.012 | 0.016 | 0.448 |
|  | rs10507349 | G | A | 0.064 | 0.007 | 1.69E-21 | -0.003 | 0.018 | 0.865 |
|  | rs10852123 | A | C | 0.060 | 0.008 | 8.38E-13 | -0.007 | 0.018 | 0.674 |
|  | rs10860209 | C | A | 0.040 | 0.007 | 5.67E-09 | 0.006 | 0.021 | 0.837 |
|  | rs10938398 | G | A | -0.046 | 0.007 | 3.84E-10 | 0.028 | 0.016 | 0.070 |
|  | rs10950550 | T | G | 0.065 | 0.007 | 1.75E-19 | -0.005 | 0.015 | 0.765 |
|  | rs10965248 | T | C | 0.183 | 0.007 | 4.42E-164 | 0.020 | 0.020 | 0.331 |
|  | rs111246699 | G | A | -0.061 | 0.008 | 1.54E-15 | -0.016 | 0.018 | 0.377 |
|  | rs113154802 | C | T | 0.060 | 0.011 | 3.51E-08 | 0.007 | 0.026 | 0.774 |
|  | rs1182444 | A | G | -0.047 | 0.007 | 1.67E-12 | 0.004 | 0.016 | 0.852 |
|  | rs11926494 | G | A | 0.112 | 0.009 | 2.69E-37 | -0.014 | 0.027 | 0.571 |
|  | rs1206684 | G | A | 0.040 | 0.007 | 4.42E-09 | -0.001 | 0.016 | 0.936 |
|  | rs123378 | G | A | 0.053 | 0.008 | 2.22E-10 | -0.012 | 0.017 | 0.483 |
|  | rs1260326 | T | C | -0.063 | 0.007 | 1.01E-21 | 0.043 | 0.016 | 0.006 |
|  | rs12625671 | T | C | -0.066 | 0.007 | 2.25E-21 | 0.019 | 0.023 | 0.405 |
|  | rs12698877 | A | G | -0.067 | 0.007 | 6.96E-22 | 0.007 | 0.017 | 0.706 |
|  | rs12818766 | G | A | -0.054 | 0.009 | 2.46E-10 | 0.008 | 0.021 | 0.704 |
|  | rs13092876 | G | A | -0.126 | 0.007 | 1.91E-66 | 0.002 | 0.016 | 0.910 |
|  | rs13266634 | C | T | 0.116 | 0.007 | 3.72E-67 | 0.016 | 0.017 | 0.360 |
|  | rs1328412 | T | C | 0.097 | 0.015 | 6.41E-11 | 0.030 | 0.030 | 0.302 |
|  | rs1421085 | T | C | -0.130 | 0.009 | 1.55E-48 | -0.013 | 0.016 | 0.409 |
|  | rs1426371 | G | A | 0.048 | 0.007 | 7.76E-12 | -0.013 | 0.018 | 0.502 |
|  | rs1459513 | A | C | -0.046 | 0.007 | 3.73E-11 | -0.017 | 0.035 | 0.668 |
|  | rs147707338 | C | T | -0.087 | 0.014 | 6.47E-10 | 0.002 | 0.026 | 0.913 |
|  | rs1513275 | T | C | -0.057 | 0.009 | 2.76E-11 | -0.039 | 0.018 | 0.040 |
|  | rs16884229 | T | C | -0.097 | 0.007 | 1.15E-43 | -0.008 | 0.020 | 0.704 |
|  | rs17168486 | C | T | -0.064 | 0.007 | 8.23E-22 | -0.019 | 0.020 | 0.332 |
|  | rs1850421 | A | C | 0.044 | 0.007 | 1.41E-09 | 0.032 | 0.017 | 0.060 |
|  | rs2074120 | A | C | 0.041 | 0.007 | 8.38E-09 | -0.001 | 0.015 | 0.969 |
|  | rs2092518 | G | T | 0.046 | 0.007 | 1.39E-10 | -0.011 | 0.016 | 0.495 |
|  | rs2126736 | A | G | 0.038 | 0.007 | 1.84E-08 | -0.006 | 0.015 | 0.697 |
|  | rs2240885 | G | A | -0.042 | 0.007 | 2.79E-09 | 0.047 | 0.019 | 0.011 |
|  | rs2269245 | G | A | 0.054 | 0.009 | 5.40E-10 | -0.041 | 0.019 | 0.036 |
|  | rs2327777 | T | C | 0.050 | 0.007 | 1.06E-13 | 0.014 | 0.015 | 0.373 |
|  | rs2583934 | G | T | -0.058 | 0.007 | 4.95E-16 | -0.032 | 0.021 | 0.158 |
|  | rs261982 | C | T | -0.040 | 0.007 | 3.12E-09 | 0.015 | 0.016 | 0.356 |
|  | rs2706710 | C | T | -0.071 | 0.013 | 1.67E-08 | 0.034 | 0.020 | 0.114 |
|  | rs2796441 | G | A | 0.075 | 0.007 | 1.43E-28 | -0.010 | 0.016 | 0.528 |
|  | rs28691713 | C | T | 0.066 | 0.008 | 1.79E-17 | 0.012 | 0.016 | 0.463 |
|  | rs2908279 | T | G | -0.046 | 0.007 | 8.42E-11 | 0.017 | 0.015 | 0.264 |
|  | rs2980883 | T | G | 0.042 | 0.007 | 3.93E-09 | -0.013 | 0.022 | 0.553 |
|  | rs3094508 | T | C | -0.059 | 0.008 | 1.31E-13 | -0.020 | 0.016 | 0.194 |
|  | rs329122 | G | A | -0.039 | 0.007 | 2.22E-08 | 0.005 | 0.016 | 0.720 |
|  | rs34811727 | G | T | -0.076 | 0.013 | 3.32E-09 | 0.057 | 0.031 | 0.055 |
|  | rs349359 | A | C | -0.043 | 0.008 | 3.05E-08 | -0.044 | 0.024 | 0.055 |
|  | rs35589574 | C | T | -0.171 | 0.011 | 1.58E-55 | -0.011 | 0.018 | 0.580 |
|  | rs3735641 | A | G | -0.069 | 0.008 | 3.62E-19 | 0.002 | 0.016 | 0.881 |
|  | rs3751236 | G | A | 0.067 | 0.007 | 6.58E-21 | -0.022 | 0.019 | 0.255 |
|  | rs3852529 | C | T | -0.099 | 0.010 | 2.07E-24 | -0.002 | 0.027 | 0.861 |
|  | rs3887925 | C | T | -0.040 | 0.007 | 3.12E-09 | -0.015 | 0.015 | 0.335 |
|  | rs391933 | G | A | 0.037 | 0.007 | 1.46E-08 | -0.022 | 0.016 | 0.157 |
|  | rs4273712 | A | G | -0.047 | 0.007 | 2.56E-12 | -0.028 | 0.017 | 0.093 |
|  | rs4499362 | C | T | 0.044 | 0.007 | 1.53E-09 | 0.018 | 0.019 | 0.344 |
|  | rs476828 | T | C | -0.084 | 0.008 | 4.81E-27 | 0.008 | 0.018 | 0.653 |
|  | rs4930974 | C | T | -0.069 | 0.008 | 4.07E-19 | 0.036 | 0.019 | 0.059 |
|  | rs532504 | G | A | -0.055 | 0.008 | 7.39E-12 | 0.003 | 0.019 | 0.925 |
|  | rs55700915 | G | A | -0.040 | 0.007 | 1.50E-08 | -0.018 | 0.019 | 0.365 |
|  | rs58524310 | A | G | -0.047 | 0.007 | 8.41E-11 | -0.018 | 0.021 | 0.448 |
|  | rs58718028 | C | T | -0.073 | 0.007 | 3.35E-25 | 0.030 | 0.018 | 0.089 |
|  | rs6012878 | G | A | 0.041 | 0.007 | 4.32E-09 | 0.006 | 0.016 | 0.716 |
|  | rs60573766 | C | T | 0.044 | 0.007 | 4.30E-10 | -0.033 | 0.022 | 0.147 |
|  | rs61779313 | T | C | -0.060 | 0.009 | 5.59E-11 | 0.015 | 0.018 | 0.398 |
|  | rs61975988 | A | G | 0.040 | 0.007 | 1.97E-09 | -0.021 | 0.018 | 0.227 |
|  | rs62173901 | A | G | 0.123 | 0.020 | 6.58E-10 | 0.026 | 0.022 | 0.256 |
|  | rs62405419 | G | T | -0.044 | 0.008 | 3.79E-09 | -0.026 | 0.024 | 0.304 |
|  | rs633862 | C | T | -0.039 | 0.007 | 1.26E-08 | 0.031 | 0.015 | 0.046 |
|  | rs6416749 | C | T | 0.052 | 0.008 | 3.40E-12 | -0.038 | 0.017 | 0.026 |
|  | rs6731688 | A | C | -0.098 | 0.012 | 2.55E-17 | -0.011 | 0.020 | 0.612 |
|  | rs703980 | G | A | 0.060 | 0.007 | 6.55E-19 | 0.008 | 0.015 | 0.633 |
|  | rs7107784 | G | A | 0.086 | 0.012 | 2.60E-13 | 0.023 | 0.017 | 0.186 |
|  | rs7109575 | G | A | 0.141 | 0.015 | 5.46E-21 | 0.020 | 0.021 | 0.309 |
|  | rs7250869 | T | C | 0.056 | 0.007 | 2.29E-16 | 0.023 | 0.017 | 0.189 |
|  | rs73085586 | G | A | 0.043 | 0.007 | 1.66E-09 | -0.016 | 0.019 | 0.403 |
|  | rs7313668 | G | T | -0.045 | 0.007 | 4.91E-11 | 0.003 | 0.016 | 0.869 |
|  | rs73347525 | A | G | 0.059 | 0.009 | 7.46E-11 | 0.004 | 0.020 | 0.903 |
|  | rs742762 | A | C | 0.075 | 0.008 | 1.79E-22 | 0.039 | 0.025 | 0.110 |
|  | rs7501939 | T | C | 0.120 | 0.007 | 1.60E-60 | 0.022 | 0.016 | 0.171 |
|  | rs75990271 | T | C | 0.066 | 0.010 | 3.22E-11 | 0.011 | 0.035 | 0.826 |
|  | rs76704029 | T | C | 0.055 | 0.010 | 3.39E-08 | -0.034 | 0.035 | 0.421 |
|  | rs7787720 | C | T | -0.054 | 0.007 | 2.25E-15 | 0.026 | 0.015 | 0.098 |
|  | rs7901695 | T | C | -0.275 | 0.017 | 8.18E-62 | 0.018 | 0.017 | 0.269 |
|  | rs80196932 | T | C | 0.060 | 0.008 | 7.57E-13 | -0.011 | 0.022 | 0.630 |
|  | rs8026714 | G | A | -0.066 | 0.007 | 1.06E-22 | -0.048 | 0.022 | 0.026 |
|  | rs8043085 | G | T | -0.052 | 0.007 | 2.06E-14 | -0.004 | 0.018 | 0.849 |
|  | rs896852 | G | T | 0.042 | 0.007 | 6.42E-09 | 0.012 | 0.015 | 0.428 |
|  | rs9316706 | A | G | 0.041 | 0.007 | 3.33E-09 | -0.008 | 0.020 | 0.742 |
|  | rs9350271 | G | A | -0.193 | 0.007 | 4.95E-183 | -0.004 | 0.016 | 0.783 |
|  | rs9379084 | G | A | 0.072 | 0.009 | 2.20E-14 | -0.010 | 0.025 | 0.624 |
|  | rs9390022 | T | C | 0.048 | 0.008 | 6.35E-09 | 0.003 | 0.016 | 0.866 |
|  | rs9461650 | G | A | 0.060 | 0.009 | 1.99E-12 | 0.019 | 0.021 | 0.380 |
|  | rs9523295 | G | A | 0.078 | 0.009 | 7.24E-18 | 0.011 | 0.018 | 0.542 |
|  | rs9788635 | C | T | 0.058 | 0.008 | 9.43E-14 | 0.010 | 0.024 | 0.716 |
|  | rs9948462 | C | T | -0.047 | 0.008 | 8.70E-10 | -0.007 | 0.016 | 0.640 |
| *Rikenellaceae* | rs10011838 | G | A | 0.073 | 0.007 | 1.43E-27 | 0.022 | 0.012 | 0.067 |
|  | rs1016565 | G | A | -0.038 | 0.007 | 2.18E-08 | -0.004 | 0.011 | 0.723 |
|  | rs10507349 | G | A | 0.064 | 0.007 | 1.69E-21 | 0.010 | 0.012 | 0.403 |
|  | rs10852123 | A | C | 0.060 | 0.008 | 8.38E-13 | -0.002 | 0.012 | 0.866 |
|  | rs10860209 | C | A | 0.040 | 0.007 | 5.67E-09 | 0.021 | 0.015 | 0.165 |
|  | rs10886863 | T | C | -0.060 | 0.007 | 5.28E-17 | -0.023 | 0.035 | 0.294 |
|  | rs10938398 | G | A | -0.046 | 0.007 | 3.84E-10 | -0.008 | 0.011 | 0.426 |
|  | rs10950550 | T | G | 0.065 | 0.007 | 1.75E-19 | 0.006 | 0.011 | 0.548 |
|  | rs10965248 | T | C | 0.183 | 0.007 | 4.42E-164 | 0.004 | 0.014 | 0.679 |
|  | rs111246699 | G | A | -0.061 | 0.008 | 1.54E-15 | 0.004 | 0.013 | 0.813 |
|  | rs113154802 | C | T | 0.060 | 0.011 | 3.51E-08 | -0.001 | 0.018 | 0.850 |
|  | rs1182444 | A | G | -0.047 | 0.007 | 1.67E-12 | -0.007 | 0.011 | 0.518 |
|  | rs11926494 | G | A | 0.112 | 0.009 | 2.69E-37 | -0.006 | 0.018 | 0.770 |
|  | rs1206684 | G | A | 0.040 | 0.007 | 4.42E-09 | 0.002 | 0.011 | 0.785 |
|  | rs123378 | G | A | 0.053 | 0.008 | 2.22E-10 | -0.010 | 0.012 | 0.403 |
|  | rs1260326 | T | C | -0.063 | 0.007 | 1.01E-21 | 0.016 | 0.011 | 0.141 |
|  | rs12610052 | T | C | -0.046 | 0.008 | 1.97E-09 | -0.013 | 0.040 | 0.363 |
|  | rs12625671 | T | C | -0.066 | 0.007 | 2.25E-21 | 0.026 | 0.016 | 0.104 |
|  | rs1266488 | T | C | 0.044 | 0.007 | 6.29E-10 | -0.022 | 0.035 | 0.332 |
|  | rs12698877 | A | G | -0.067 | 0.007 | 6.96E-22 | 0.013 | 0.012 | 0.275 |
|  | rs12818766 | G | A | -0.054 | 0.009 | 2.46E-10 | 0.002 | 0.014 | 0.931 |
|  | rs13092876 | G | A | -0.126 | 0.007 | 1.91E-66 | -0.009 | 0.011 | 0.442 |
|  | rs13266634 | C | T | 0.116 | 0.007 | 3.72E-67 | -0.008 | 0.012 | 0.515 |
|  | rs1328412 | T | C | 0.097 | 0.015 | 6.41E-11 | 0.036 | 0.021 | 0.085 |
|  | rs1421085 | T | C | -0.130 | 0.009 | 1.55E-48 | -0.013 | 0.011 | 0.242 |
|  | rs1426371 | G | A | 0.048 | 0.007 | 7.76E-12 | -0.011 | 0.012 | 0.347 |
|  | rs1459513 | A | C | -0.046 | 0.007 | 3.73E-11 | 0.007 | 0.023 | 0.943 |
|  | rs147707338 | C | T | -0.087 | 0.014 | 6.47E-10 | 0.021 | 0.018 | 0.280 |
|  | rs1513275 | T | C | -0.057 | 0.009 | 2.76E-11 | -0.021 | 0.013 | 0.108 |
|  | rs16884229 | T | C | -0.097 | 0.007 | 1.15E-43 | -0.019 | 0.013 | 0.194 |
|  | rs17168486 | C | T | -0.064 | 0.007 | 8.23E-22 | 0.000 | 0.014 | 0.999 |
|  | rs1850421 | A | C | 0.044 | 0.007 | 1.41E-09 | -0.014 | 0.012 | 0.223 |
|  | rs2074120 | A | C | 0.041 | 0.007 | 8.38E-09 | -0.014 | 0.011 | 0.200 |
|  | rs2092518 | G | T | 0.046 | 0.007 | 1.39E-10 | -0.005 | 0.011 | 0.609 |
|  | rs2126736 | A | G | 0.038 | 0.007 | 1.84E-08 | 0.020 | 0.011 | 0.055 |
|  | rs2240885 | G | A | -0.042 | 0.007 | 2.79E-09 | -0.003 | 0.013 | 0.892 |
|  | rs2269245 | G | A | 0.054 | 0.009 | 5.40E-10 | -0.003 | 0.013 | 0.867 |
|  | rs2327777 | T | C | 0.050 | 0.007 | 1.06E-13 | 0.011 | 0.011 | 0.321 |
|  | rs2583934 | G | T | -0.058 | 0.007 | 4.95E-16 | 0.006 | 0.015 | 0.716 |
|  | rs261982 | C | T | -0.040 | 0.007 | 3.12E-09 | -0.014 | 0.011 | 0.179 |
|  | rs2706710 | C | T | -0.071 | 0.013 | 1.67E-08 | -0.010 | 0.014 | 0.521 |
|  | rs2796441 | G | A | 0.075 | 0.007 | 1.43E-28 | 0.012 | 0.011 | 0.277 |
|  | rs28599782 | G | A | -0.067 | 0.008 | 4.64E-16 | -0.022 | 0.031 | 0.392 |
|  | rs28691713 | C | T | 0.066 | 0.008 | 1.79E-17 | -0.004 | 0.011 | 0.687 |
|  | rs2908279 | T | G | -0.046 | 0.007 | 8.42E-11 | 0.029 | 0.011 | 0.007 |
|  | rs2980883 | T | G | 0.042 | 0.007 | 3.93E-09 | -0.003 | 0.015 | 0.845 |
|  | rs3094508 | T | C | -0.059 | 0.008 | 1.31E-13 | -0.009 | 0.011 | 0.396 |
|  | rs329122 | G | A | -0.039 | 0.007 | 2.22E-08 | 0.006 | 0.011 | 0.612 |
|  | rs34811727 | G | T | -0.076 | 0.013 | 3.32E-09 | -0.015 | 0.022 | 0.435 |
|  | rs349359 | A | C | -0.043 | 0.008 | 3.05E-08 | 0.004 | 0.016 | 0.759 |
|  | rs35589574 | C | T | -0.171 | 0.011 | 1.58E-55 | 0.011 | 0.013 | 0.366 |
|  | rs3735641 | A | G | -0.069 | 0.008 | 3.62E-19 | 0.006 | 0.011 | 0.576 |
|  | rs3751236 | G | A | 0.067 | 0.007 | 6.58E-21 | 0.004 | 0.013 | 0.724 |
|  | rs3852529 | C | T | -0.099 | 0.010 | 2.07E-24 | 0.009 | 0.019 | 0.586 |
|  | rs3887925 | C | T | -0.040 | 0.007 | 3.12E-09 | 0.016 | 0.011 | 0.132 |
|  | rs391933 | G | A | 0.037 | 0.007 | 1.46E-08 | -0.001 | 0.011 | 0.907 |
|  | rs4273712 | A | G | -0.047 | 0.007 | 2.56E-12 | -0.007 | 0.012 | 0.552 |
|  | rs4499362 | C | T | 0.044 | 0.007 | 1.53E-09 | -0.004 | 0.013 | 0.863 |
|  | rs476828 | T | C | -0.084 | 0.008 | 4.81E-27 | -0.008 | 0.012 | 0.500 |
|  | rs4930974 | C | T | -0.069 | 0.008 | 4.07E-19 | 0.003 | 0.013 | 0.802 |
|  | rs532504 | G | A | -0.055 | 0.008 | 7.39E-12 | -0.004 | 0.013 | 0.723 |
|  | rs55700915 | G | A | -0.040 | 0.007 | 1.50E-08 | 0.002 | 0.013 | 0.894 |
|  | rs58524310 | A | G | -0.047 | 0.007 | 8.41E-11 | 0.007 | 0.015 | 0.586 |
|  | rs58718028 | C | T | -0.073 | 0.007 | 3.35E-25 | -0.025 | 0.012 | 0.043 |
|  | rs6012878 | G | A | 0.041 | 0.007 | 4.32E-09 | 0.007 | 0.011 | 0.539 |
|  | rs60573766 | C | T | 0.044 | 0.007 | 4.30E-10 | -0.015 | 0.015 | 0.341 |
|  | rs61779313 | T | C | -0.060 | 0.009 | 5.59E-11 | -0.003 | 0.013 | 0.828 |
|  | rs61975988 | A | G | 0.040 | 0.007 | 1.97E-09 | -0.018 | 0.012 | 0.149 |
|  | rs62173901 | A | G | 0.123 | 0.020 | 6.58E-10 | 0.006 | 0.016 | 0.877 |
|  | rs62405419 | G | T | -0.044 | 0.008 | 3.79E-09 | 0.002 | 0.016 | 0.900 |
|  | rs633862 | C | T | -0.039 | 0.007 | 1.26E-08 | 0.000 | 0.011 | 0.978 |
|  | rs6416749 | C | T | 0.052 | 0.008 | 3.40E-12 | -0.004 | 0.012 | 0.689 |
|  | rs6731688 | A | C | -0.098 | 0.012 | 2.55E-17 | -0.014 | 0.014 | 0.396 |
|  | rs703980 | G | A | 0.060 | 0.007 | 6.55E-19 | 0.002 | 0.011 | 0.871 |
|  | rs7107784 | G | A | 0.086 | 0.012 | 2.60E-13 | 0.013 | 0.012 | 0.309 |
|  | rs7109575 | G | A | 0.141 | 0.015 | 5.46E-21 | -0.005 | 0.015 | 0.707 |
|  | rs7250869 | T | C | 0.056 | 0.007 | 2.29E-16 | 0.009 | 0.012 | 0.459 |
|  | rs7304270 | C | T | 0.068 | 0.010 | 1.04E-12 | -0.026 | 0.057 | 0.700 |
|  | rs73085586 | G | A | 0.043 | 0.007 | 1.66E-09 | -0.007 | 0.013 | 0.588 |
|  | rs7313668 | G | T | -0.045 | 0.007 | 4.91E-11 | 0.000 | 0.011 | 0.974 |
|  | rs73347525 | A | G | 0.059 | 0.009 | 7.46E-11 | -0.015 | 0.014 | 0.244 |
|  | rs742762 | A | C | 0.075 | 0.008 | 1.79E-22 | -0.001 | 0.017 | 0.985 |
|  | rs7501939 | T | C | 0.120 | 0.007 | 1.60E-60 | -0.011 | 0.011 | 0.317 |
|  | rs75990271 | T | C | 0.066 | 0.010 | 3.22E-11 | -0.017 | 0.024 | 0.561 |
|  | rs7656416 | C | T | 0.100 | 0.007 | 9.01E-42 | -0.015 | 0.032 | 0.719 |
|  | rs76704029 | T | C | 0.055 | 0.010 | 3.39E-08 | -0.008 | 0.025 | 0.937 |
|  | rs76878791 | A | G | -0.053 | 0.007 | 2.02E-13 | 0.015 | 0.048 | 0.753 |
|  | rs77789961 | C | T | -0.047 | 0.008 | 1.92E-08 | 0.009 | 0.042 | 0.946 |
|  | rs7787720 | C | T | -0.054 | 0.007 | 2.25E-15 | -0.004 | 0.011 | 0.687 |
|  | rs7901695 | T | C | -0.275 | 0.017 | 8.18E-62 | 0.009 | 0.012 | 0.485 |
|  | rs80196932 | T | C | 0.060 | 0.008 | 7.57E-13 | -0.021 | 0.015 | 0.122 |
|  | rs8026714 | G | A | -0.066 | 0.007 | 1.06E-22 | 0.020 | 0.015 | 0.200 |
|  | rs8043085 | G | T | -0.052 | 0.007 | 2.06E-14 | 0.007 | 0.012 | 0.504 |
|  | rs896852 | G | T | 0.042 | 0.007 | 6.42E-09 | -0.017 | 0.011 | 0.117 |
|  | rs9316706 | A | G | 0.041 | 0.007 | 3.33E-09 | -0.008 | 0.014 | 0.597 |
|  | rs9350271 | G | A | -0.193 | 0.007 | 4.95E-183 | -0.015 | 0.011 | 0.178 |
|  | rs9379084 | G | A | 0.072 | 0.009 | 2.20E-14 | -0.007 | 0.017 | 0.705 |
|  | rs9390022 | T | C | 0.048 | 0.008 | 6.35E-09 | 0.023 | 0.011 | 0.039 |
|  | rs9461650 | G | A | 0.060 | 0.009 | 1.99E-12 | 0.003 | 0.015 | 0.732 |
|  | rs9523295 | G | A | 0.078 | 0.009 | 7.24E-18 | -0.021 | 0.012 | 0.085 |
|  | rs9788635 | C | T | 0.058 | 0.008 | 9.43E-14 | 0.020 | 0.016 | 0.181 |
|  | rs9948462 | C | T | -0.047 | 0.008 | 8.70E-10 | -0.002 | 0.011 | 0.877 |
| *Ruminococcaceae* | rs10011838 | G | A | 0.073 | 0.007 | 1.43E-27 | -0.005 | 0.011 | 0.642 |
|  | rs1016565 | G | A | -0.038 | 0.007 | 2.18E-08 | 0.005 | 0.011 | 0.669 |
|  | rs10507349 | G | A | 0.064 | 0.007 | 1.69E-21 | 0.004 | 0.012 | 0.823 |
|  | rs10852123 | A | C | 0.060 | 0.008 | 8.38E-13 | -0.012 | 0.012 | 0.321 |
|  | rs10860209 | C | A | 0.040 | 0.007 | 5.67E-09 | -0.016 | 0.014 | 0.225 |
|  | rs10886863 | T | C | -0.060 | 0.007 | 5.28E-17 | 0.007 | 0.033 | 0.858 |
|  | rs10938398 | G | A | -0.046 | 0.007 | 3.84E-10 | -0.022 | 0.011 | 0.035 |
|  | rs10950550 | T | G | 0.065 | 0.007 | 1.75E-19 | -0.015 | 0.010 | 0.164 |
|  | rs10965248 | T | C | 0.183 | 0.007 | 4.42E-164 | 0.004 | 0.014 | 0.767 |
|  | rs111246699 | G | A | -0.061 | 0.008 | 1.54E-15 | 0.023 | 0.012 | 0.066 |
|  | rs113154802 | C | T | 0.060 | 0.011 | 3.51E-08 | 0.003 | 0.018 | 0.986 |
|  | rs1182444 | A | G | -0.047 | 0.007 | 1.67E-12 | 0.008 | 0.011 | 0.452 |
|  | rs11926494 | G | A | 0.112 | 0.009 | 2.69E-37 | -0.029 | 0.018 | 0.133 |
|  | rs1206684 | G | A | 0.040 | 0.007 | 4.42E-09 | 0.004 | 0.011 | 0.706 |
|  | rs123378 | G | A | 0.053 | 0.008 | 2.22E-10 | 0.005 | 0.011 | 0.649 |
|  | rs1260326 | T | C | -0.063 | 0.007 | 1.01E-21 | 0.017 | 0.011 | 0.122 |
|  | rs12610052 | T | C | -0.046 | 0.008 | 1.97E-09 | 0.031 | 0.039 | 0.413 |
|  | rs12625671 | T | C | -0.066 | 0.007 | 2.25E-21 | -0.038 | 0.015 | 0.012 |
|  | rs1266488 | T | C | 0.044 | 0.007 | 6.29E-10 | 0.004 | 0.033 | 0.898 |
|  | rs12698877 | A | G | -0.067 | 0.007 | 6.96E-22 | 0.002 | 0.012 | 0.866 |
|  | rs12818766 | G | A | -0.054 | 0.009 | 2.46E-10 | -0.016 | 0.014 | 0.238 |
|  | rs13092876 | G | A | -0.126 | 0.007 | 1.91E-66 | 0.006 | 0.011 | 0.595 |
|  | rs13266634 | C | T | 0.116 | 0.007 | 3.72E-67 | 0.004 | 0.011 | 0.773 |
|  | rs1328412 | T | C | 0.097 | 0.015 | 6.41E-11 | 0.030 | 0.021 | 0.149 |
|  | rs1421085 | T | C | -0.130 | 0.009 | 1.55E-48 | 0.003 | 0.011 | 0.768 |
|  | rs1426371 | G | A | 0.048 | 0.007 | 7.76E-12 | 0.012 | 0.012 | 0.324 |
|  | rs1459513 | A | C | -0.046 | 0.007 | 3.73E-11 | 0.010 | 0.023 | 0.788 |
|  | rs147707338 | C | T | -0.087 | 0.014 | 6.47E-10 | 0.019 | 0.018 | 0.331 |
|  | rs1513275 | T | C | -0.057 | 0.009 | 2.76E-11 | -0.025 | 0.012 | 0.041 |
|  | rs16884229 | T | C | -0.097 | 0.007 | 1.15E-43 | -0.009 | 0.013 | 0.524 |
|  | rs17168486 | C | T | -0.064 | 0.007 | 8.23E-22 | -0.002 | 0.013 | 0.856 |
|  | rs1850421 | A | C | 0.044 | 0.007 | 1.41E-09 | 0.007 | 0.011 | 0.551 |
|  | rs2074120 | A | C | 0.041 | 0.007 | 8.38E-09 | -0.020 | 0.010 | 0.048 |
|  | rs2092518 | G | T | 0.046 | 0.007 | 1.39E-10 | 0.008 | 0.011 | 0.442 |
|  | rs2126736 | A | G | 0.038 | 0.007 | 1.84E-08 | -0.011 | 0.011 | 0.277 |
|  | rs2240885 | G | A | -0.042 | 0.007 | 2.79E-09 | 0.007 | 0.013 | 0.537 |
|  | rs2269245 | G | A | 0.054 | 0.009 | 5.40E-10 | -0.005 | 0.013 | 0.750 |
|  | rs2327777 | T | C | 0.050 | 0.007 | 1.06E-13 | 0.016 | 0.010 | 0.124 |
|  | rs2583934 | G | T | -0.058 | 0.007 | 4.95E-16 | -0.012 | 0.014 | 0.365 |
|  | rs261982 | C | T | -0.040 | 0.007 | 3.12E-09 | -0.002 | 0.011 | 0.810 |
|  | rs2706710 | C | T | -0.071 | 0.013 | 1.67E-08 | -0.032 | 0.014 | 0.023 |
|  | rs2796441 | G | A | 0.075 | 0.007 | 1.43E-28 | 0.005 | 0.011 | 0.665 |
|  | rs28599782 | G | A | -0.067 | 0.008 | 4.64E-16 | 0.011 | 0.029 | 0.988 |
|  | rs28691713 | C | T | 0.066 | 0.008 | 1.79E-17 | 0.005 | 0.011 | 0.658 |
|  | rs2908279 | T | G | -0.046 | 0.007 | 8.42E-11 | -0.017 | 0.010 | 0.093 |
|  | rs2980883 | T | G | 0.042 | 0.007 | 3.93E-09 | 0.003 | 0.015 | 0.904 |
|  | rs3094508 | T | C | -0.059 | 0.008 | 1.31E-13 | -0.003 | 0.011 | 0.753 |
|  | rs329122 | G | A | -0.039 | 0.007 | 2.22E-08 | -0.012 | 0.011 | 0.274 |
|  | rs34811727 | G | T | -0.076 | 0.013 | 3.32E-09 | -0.020 | 0.021 | 0.383 |
|  | rs349359 | A | C | -0.043 | 0.008 | 3.05E-08 | -0.007 | 0.016 | 0.752 |
|  | rs35589574 | C | T | -0.171 | 0.011 | 1.58E-55 | 0.013 | 0.012 | 0.402 |
|  | rs3735641 | A | G | -0.069 | 0.008 | 3.62E-19 | -0.015 | 0.011 | 0.175 |
|  | rs3751236 | G | A | 0.067 | 0.007 | 6.58E-21 | -0.020 | 0.013 | 0.107 |
|  | rs3852529 | C | T | -0.099 | 0.010 | 2.07E-24 | -0.004 | 0.018 | 0.671 |
|  | rs3887925 | C | T | -0.040 | 0.007 | 3.12E-09 | 0.019 | 0.011 | 0.071 |
|  | rs391933 | G | A | 0.037 | 0.007 | 1.46E-08 | -0.004 | 0.011 | 0.709 |
|  | rs4273712 | A | G | -0.047 | 0.007 | 2.56E-12 | -0.023 | 0.012 | 0.046 |
|  | rs4499362 | C | T | 0.044 | 0.007 | 1.53E-09 | 0.020 | 0.013 | 0.116 |
|  | rs476828 | T | C | -0.084 | 0.008 | 4.81E-27 | -0.006 | 0.012 | 0.689 |
|  | rs4930974 | C | T | -0.069 | 0.008 | 4.07E-19 | 0.013 | 0.013 | 0.342 |
|  | rs532504 | G | A | -0.055 | 0.008 | 7.39E-12 | -0.009 | 0.013 | 0.444 |
|  | rs55700915 | G | A | -0.040 | 0.007 | 1.50E-08 | 0.000 | 0.013 | 0.994 |
|  | rs58524310 | A | G | -0.047 | 0.007 | 8.41E-11 | -0.001 | 0.015 | 0.904 |
|  | rs58718028 | C | T | -0.073 | 0.007 | 3.35E-25 | -0.005 | 0.012 | 0.702 |
|  | rs6012878 | G | A | 0.041 | 0.007 | 4.32E-09 | -0.021 | 0.011 | 0.053 |
|  | rs60573766 | C | T | 0.044 | 0.007 | 4.30E-10 | 0.005 | 0.015 | 0.715 |
|  | rs61779313 | T | C | -0.060 | 0.009 | 5.59E-11 | -0.018 | 0.012 | 0.140 |
|  | rs61975988 | A | G | 0.040 | 0.007 | 1.97E-09 | 0.011 | 0.012 | 0.372 |
|  | rs62173901 | A | G | 0.123 | 0.020 | 6.58E-10 | -0.019 | 0.015 | 0.186 |
|  | rs62405419 | G | T | -0.044 | 0.008 | 3.79E-09 | 0.005 | 0.016 | 0.705 |
|  | rs633862 | C | T | -0.039 | 0.007 | 1.26E-08 | -0.002 | 0.011 | 0.871 |
|  | rs6416749 | C | T | 0.052 | 0.008 | 3.40E-12 | -0.009 | 0.011 | 0.442 |
|  | rs6731688 | A | C | -0.098 | 0.012 | 2.55E-17 | -0.011 | 0.014 | 0.527 |
|  | rs703980 | G | A | 0.060 | 0.007 | 6.55E-19 | -0.009 | 0.010 | 0.405 |
|  | rs7107784 | G | A | 0.086 | 0.012 | 2.60E-13 | 0.005 | 0.012 | 0.654 |
|  | rs7109575 | G | A | 0.141 | 0.015 | 5.46E-21 | -0.008 | 0.015 | 0.576 |
|  | rs7250869 | T | C | 0.056 | 0.007 | 2.29E-16 | 0.010 | 0.011 | 0.386 |
|  | rs7304270 | C | T | 0.068 | 0.010 | 1.04E-12 | -0.023 | 0.057 | 0.657 |
|  | rs73085586 | G | A | 0.043 | 0.007 | 1.66E-09 | -0.008 | 0.013 | 0.421 |
|  | rs7313668 | G | T | -0.045 | 0.007 | 4.91E-11 | 0.016 | 0.011 | 0.129 |
|  | rs73347525 | A | G | 0.059 | 0.009 | 7.46E-11 | 0.010 | 0.014 | 0.527 |
|  | rs742762 | A | C | 0.075 | 0.008 | 1.79E-22 | -0.002 | 0.017 | 0.956 |
|  | rs7501939 | T | C | 0.120 | 0.007 | 1.60E-60 | 0.010 | 0.011 | 0.349 |
|  | rs75990271 | T | C | 0.066 | 0.010 | 3.22E-11 | 0.016 | 0.024 | 0.397 |
|  | rs7656416 | C | T | 0.100 | 0.007 | 9.01E-42 | 0.012 | 0.030 | 0.754 |
|  | rs76704029 | T | C | 0.055 | 0.010 | 3.39E-08 | -0.039 | 0.024 | 0.106 |
|  | rs76878791 | A | G | -0.053 | 0.007 | 2.02E-13 | -0.005 | 0.046 | 0.955 |
|  | rs77789961 | C | T | -0.047 | 0.008 | 1.92E-08 | 0.020 | 0.039 | 0.536 |
|  | rs7787720 | C | T | -0.054 | 0.007 | 2.25E-15 | 0.006 | 0.011 | 0.592 |
|  | rs7901695 | T | C | -0.275 | 0.017 | 8.18E-62 | -0.001 | 0.011 | 0.928 |
|  | rs80196932 | T | C | 0.060 | 0.008 | 7.57E-13 | 0.001 | 0.015 | 0.851 |
|  | rs8026714 | G | A | -0.066 | 0.007 | 1.06E-22 | 0.000 | 0.015 | 0.986 |
|  | rs8043085 | G | T | -0.052 | 0.007 | 2.06E-14 | 0.006 | 0.012 | 0.639 |
|  | rs896852 | G | T | 0.042 | 0.007 | 6.42E-09 | 0.013 | 0.011 | 0.239 |
|  | rs9316706 | A | G | 0.041 | 0.007 | 3.33E-09 | -0.010 | 0.014 | 0.467 |
|  | rs9350271 | G | A | -0.193 | 0.007 | 4.95E-183 | -0.013 | 0.011 | 0.252 |
|  | rs9379084 | G | A | 0.072 | 0.009 | 2.20E-14 | -0.006 | 0.017 | 0.758 |
|  | rs9390022 | T | C | 0.048 | 0.008 | 6.35E-09 | -0.004 | 0.011 | 0.721 |
|  | rs9461650 | G | A | 0.060 | 0.009 | 1.99E-12 | -0.034 | 0.014 | 0.019 |
|  | rs9523295 | G | A | 0.078 | 0.009 | 7.24E-18 | -0.014 | 0.012 | 0.259 |
|  | rs9788635 | C | T | 0.058 | 0.008 | 9.43E-14 | -0.019 | 0.016 | 0.260 |
|  | rs9948462 | C | T | -0.047 | 0.008 | 8.70E-10 | -0.007 | 0.011 | 0.553 |
| *Streptococcaceae* | rs10011838 | G | A | 0.073 | 0.007 | 1.43E-27 | 0.007 | 0.012 | 0.517 |
|  | rs1016565 | G | A | -0.038 | 0.007 | 2.18E-08 | 0.019 | 0.012 | 0.106 |
|  | rs10507349 | G | A | 0.064 | 0.007 | 1.69E-21 | 0.016 | 0.013 | 0.223 |
|  | rs10852123 | A | C | 0.060 | 0.008 | 8.38E-13 | -0.002 | 0.013 | 0.886 |
|  | rs10860209 | C | A | 0.040 | 0.007 | 5.67E-09 | -0.016 | 0.015 | 0.393 |
|  | rs10886863 | T | C | -0.060 | 0.007 | 5.28E-17 | 0.033 | 0.037 | 0.539 |
|  | rs10938398 | G | A | -0.046 | 0.007 | 3.84E-10 | -0.005 | 0.011 | 0.689 |
|  | rs10950550 | T | G | 0.065 | 0.007 | 1.75E-19 | -0.001 | 0.011 | 0.894 |
|  | rs10965248 | T | C | 0.183 | 0.007 | 4.42E-164 | -0.017 | 0.014 | 0.255 |
|  | rs111246699 | G | A | -0.061 | 0.008 | 1.54E-15 | 0.009 | 0.013 | 0.456 |
|  | rs113154802 | C | T | 0.060 | 0.011 | 3.51E-08 | -0.002 | 0.019 | 0.966 |
|  | rs1182444 | A | G | -0.047 | 0.007 | 1.67E-12 | 0.005 | 0.012 | 0.645 |
|  | rs11926494 | G | A | 0.112 | 0.009 | 2.69E-37 | 0.028 | 0.019 | 0.178 |
|  | rs1206684 | G | A | 0.040 | 0.007 | 4.42E-09 | 0.007 | 0.011 | 0.501 |
|  | rs123378 | G | A | 0.053 | 0.008 | 2.22E-10 | 0.011 | 0.012 | 0.361 |
|  | rs1260326 | T | C | -0.063 | 0.007 | 1.01E-21 | 0.010 | 0.011 | 0.377 |
|  | rs12610052 | T | C | -0.046 | 0.008 | 1.97E-09 | -0.015 | 0.041 | 0.460 |
|  | rs12625671 | T | C | -0.066 | 0.007 | 2.25E-21 | 0.016 | 0.016 | 0.333 |
|  | rs1266488 | T | C | 0.044 | 0.007 | 6.29E-10 | -0.020 | 0.036 | 0.498 |
|  | rs12698877 | A | G | -0.067 | 0.007 | 6.96E-22 | -0.002 | 0.012 | 0.906 |
|  | rs12818766 | G | A | -0.054 | 0.009 | 2.46E-10 | -0.013 | 0.015 | 0.333 |
|  | rs13092876 | G | A | -0.126 | 0.007 | 1.91E-66 | 0.000 | 0.012 | 0.992 |
|  | rs13266634 | C | T | 0.116 | 0.007 | 3.72E-67 | 0.007 | 0.012 | 0.553 |
|  | rs1328412 | T | C | 0.097 | 0.015 | 6.41E-11 | 0.003 | 0.022 | 0.881 |
|  | rs1421085 | T | C | -0.130 | 0.009 | 1.55E-48 | -0.007 | 0.011 | 0.557 |
|  | rs1426371 | G | A | 0.048 | 0.007 | 7.76E-12 | 0.010 | 0.013 | 0.434 |
|  | rs1459513 | A | C | -0.046 | 0.007 | 3.73E-11 | 0.002 | 0.024 | 0.920 |
|  | rs147707338 | C | T | -0.087 | 0.014 | 6.47E-10 | -0.006 | 0.019 | 0.729 |
|  | rs1513275 | T | C | -0.057 | 0.009 | 2.76E-11 | -0.018 | 0.013 | 0.191 |
|  | rs16884229 | T | C | -0.097 | 0.007 | 1.15E-43 | -0.004 | 0.014 | 0.702 |
|  | rs17168486 | C | T | -0.064 | 0.007 | 8.23E-22 | -0.008 | 0.014 | 0.501 |
|  | rs1850421 | A | C | 0.044 | 0.007 | 1.41E-09 | 0.006 | 0.012 | 0.604 |
|  | rs2074120 | A | C | 0.041 | 0.007 | 8.38E-09 | 0.003 | 0.011 | 0.762 |
|  | rs2092518 | G | T | 0.046 | 0.007 | 1.39E-10 | 0.001 | 0.011 | 0.936 |
|  | rs2126736 | A | G | 0.038 | 0.007 | 1.84E-08 | -0.010 | 0.011 | 0.359 |
|  | rs2240885 | G | A | -0.042 | 0.007 | 2.79E-09 | -0.003 | 0.013 | 0.869 |
|  | rs2269245 | G | A | 0.054 | 0.009 | 5.40E-10 | 0.014 | 0.014 | 0.325 |
|  | rs2327777 | T | C | 0.050 | 0.007 | 1.06E-13 | 0.009 | 0.011 | 0.416 |
|  | rs2583934 | G | T | -0.058 | 0.007 | 4.95E-16 | 0.001 | 0.015 | 0.885 |
|  | rs261982 | C | T | -0.040 | 0.007 | 3.12E-09 | 0.015 | 0.011 | 0.167 |
|  | rs2706710 | C | T | -0.071 | 0.013 | 1.67E-08 | 0.040 | 0.015 | 0.006 |
|  | rs2796441 | G | A | 0.075 | 0.007 | 1.43E-28 | -0.012 | 0.011 | 0.291 |
|  | rs28599782 | G | A | -0.067 | 0.008 | 4.64E-16 | -0.015 | 0.032 | 0.786 |
|  | rs28691713 | C | T | 0.066 | 0.008 | 1.79E-17 | 0.002 | 0.011 | 0.876 |
|  | rs2908279 | T | G | -0.046 | 0.007 | 8.42E-11 | -0.002 | 0.011 | 0.842 |
|  | rs2980883 | T | G | 0.042 | 0.007 | 3.93E-09 | 0.002 | 0.015 | 0.865 |
|  | rs3094508 | T | C | -0.059 | 0.008 | 1.31E-13 | 0.001 | 0.011 | 0.986 |
|  | rs329122 | G | A | -0.039 | 0.007 | 2.22E-08 | 0.007 | 0.011 | 0.561 |
|  | rs34811727 | G | T | -0.076 | 0.013 | 3.32E-09 | 0.013 | 0.022 | 0.564 |
|  | rs349359 | A | C | -0.043 | 0.008 | 3.05E-08 | 0.015 | 0.017 | 0.395 |
|  | rs35589574 | C | T | -0.171 | 0.011 | 1.58E-55 | -0.013 | 0.013 | 0.357 |
|  | rs3735641 | A | G | -0.069 | 0.008 | 3.62E-19 | 0.011 | 0.011 | 0.338 |
|  | rs3751236 | G | A | 0.067 | 0.007 | 6.58E-21 | 0.028 | 0.014 | 0.037 |
|  | rs3852529 | C | T | -0.099 | 0.010 | 2.07E-24 | 0.012 | 0.019 | 0.542 |
|  | rs3887925 | C | T | -0.040 | 0.007 | 3.12E-09 | -0.007 | 0.011 | 0.513 |
|  | rs391933 | G | A | 0.037 | 0.007 | 1.46E-08 | 0.002 | 0.011 | 0.876 |
|  | rs4273712 | A | G | -0.047 | 0.007 | 2.56E-12 | -0.014 | 0.012 | 0.322 |
|  | rs4499362 | C | T | 0.044 | 0.007 | 1.53E-09 | -0.026 | 0.014 | 0.053 |
|  | rs476828 | T | C | -0.084 | 0.008 | 4.81E-27 | 0.026 | 0.013 | 0.033 |
|  | rs4930974 | C | T | -0.069 | 0.008 | 4.07E-19 | 0.003 | 0.013 | 0.806 |
|  | rs532504 | G | A | -0.055 | 0.008 | 7.39E-12 | 0.013 | 0.014 | 0.314 |
|  | rs55700915 | G | A | -0.040 | 0.007 | 1.50E-08 | 0.000 | 0.014 | 0.941 |
|  | rs58524310 | A | G | -0.047 | 0.007 | 8.41E-11 | 0.006 | 0.015 | 0.727 |
|  | rs58718028 | C | T | -0.073 | 0.007 | 3.35E-25 | 0.029 | 0.013 | 0.020 |
|  | rs6012878 | G | A | 0.041 | 0.007 | 4.32E-09 | 0.015 | 0.011 | 0.181 |
|  | rs60573766 | C | T | 0.044 | 0.007 | 4.30E-10 | 0.018 | 0.016 | 0.252 |
|  | rs61779313 | T | C | -0.060 | 0.009 | 5.59E-11 | -0.026 | 0.013 | 0.040 |
|  | rs61975988 | A | G | 0.040 | 0.007 | 1.97E-09 | -0.012 | 0.013 | 0.336 |
|  | rs62173901 | A | G | 0.123 | 0.020 | 6.58E-10 | 0.006 | 0.016 | 0.727 |
|  | rs62405419 | G | T | -0.044 | 0.008 | 3.79E-09 | -0.002 | 0.017 | 0.863 |
|  | rs633862 | C | T | -0.039 | 0.007 | 1.26E-08 | 0.014 | 0.011 | 0.210 |
|  | rs6416749 | C | T | 0.052 | 0.008 | 3.40E-12 | 0.013 | 0.012 | 0.284 |
|  | rs6731688 | A | C | -0.098 | 0.012 | 2.55E-17 | 0.017 | 0.015 | 0.241 |
|  | rs703980 | G | A | 0.060 | 0.007 | 6.55E-19 | -0.006 | 0.011 | 0.579 |
|  | rs7107784 | G | A | 0.086 | 0.012 | 2.60E-13 | -0.013 | 0.012 | 0.288 |
|  | rs7109575 | G | A | 0.141 | 0.015 | 5.46E-21 | 0.023 | 0.015 | 0.140 |
|  | rs7250869 | T | C | 0.056 | 0.007 | 2.29E-16 | -0.015 | 0.012 | 0.205 |
|  | rs73085586 | G | A | 0.043 | 0.007 | 1.66E-09 | 0.011 | 0.014 | 0.402 |
|  | rs7313668 | G | T | -0.045 | 0.007 | 4.91E-11 | -0.002 | 0.011 | 0.840 |
|  | rs73347525 | A | G | 0.059 | 0.009 | 7.46E-11 | 0.024 | 0.014 | 0.088 |
|  | rs742762 | A | C | 0.075 | 0.008 | 1.79E-22 | -0.014 | 0.017 | 0.413 |
|  | rs7501939 | T | C | 0.120 | 0.007 | 1.60E-60 | 0.024 | 0.011 | 0.036 |
|  | rs75990271 | T | C | 0.066 | 0.010 | 3.22E-11 | -0.044 | 0.025 | 0.061 |
|  | rs7656416 | C | T | 0.100 | 0.007 | 9.01E-42 | -0.001 | 0.034 | 0.932 |
|  | rs76704029 | T | C | 0.055 | 0.010 | 3.39E-08 | 0.013 | 0.026 | 0.615 |
|  | rs76878791 | A | G | -0.053 | 0.007 | 2.02E-13 | -0.076 | 0.051 | 0.156 |
|  | rs77789961 | C | T | -0.047 | 0.008 | 1.92E-08 | 0.085 | 0.044 | 0.208 |
|  | rs7787720 | C | T | -0.054 | 0.007 | 2.25E-15 | -0.015 | 0.011 | 0.174 |
|  | rs7901695 | T | C | -0.275 | 0.017 | 8.18E-62 | -0.011 | 0.012 | 0.354 |
|  | rs80196932 | T | C | 0.060 | 0.008 | 7.57E-13 | 0.000 | 0.015 | 0.948 |
|  | rs8026714 | G | A | -0.066 | 0.007 | 1.06E-22 | -0.004 | 0.016 | 0.751 |
|  | rs8043085 | G | T | -0.052 | 0.007 | 2.06E-14 | 0.016 | 0.013 | 0.231 |
|  | rs896852 | G | T | 0.042 | 0.007 | 6.42E-09 | -0.002 | 0.011 | 0.823 |
|  | rs9316706 | A | G | 0.041 | 0.007 | 3.33E-09 | -0.014 | 0.015 | 0.369 |
|  | rs9350271 | G | A | -0.193 | 0.007 | 4.95E-183 | 0.017 | 0.012 | 0.147 |
|  | rs9379084 | G | A | 0.072 | 0.009 | 2.20E-14 | -0.043 | 0.018 | 0.016 |
|  | rs9390022 | T | C | 0.048 | 0.008 | 6.35E-09 | -0.013 | 0.012 | 0.270 |
|  | rs9461650 | G | A | 0.060 | 0.009 | 1.99E-12 | 0.013 | 0.015 | 0.440 |
|  | rs9523295 | G | A | 0.078 | 0.009 | 7.24E-18 | 0.018 | 0.013 | 0.149 |
|  | rs9788635 | C | T | 0.058 | 0.008 | 9.43E-14 | 0.015 | 0.017 | 0.322 |
|  | rs9948462 | C | T | -0.047 | 0.008 | 8.70E-10 | 0.015 | 0.011 | 0.176 |
| *Veillonellaceae* | rs10011838 | G | A | 0.073 | 0.007 | 1.43E-27 | 0.007 | 0.012 | 0.534 |
|  | rs1016565 | G | A | -0.038 | 0.007 | 2.18E-08 | 0.015 | 0.012 | 0.218 |
|  | rs10507349 | G | A | 0.064 | 0.007 | 1.69E-21 | -0.011 | 0.013 | 0.367 |
|  | rs10852123 | A | C | 0.060 | 0.008 | 8.38E-13 | 0.019 | 0.013 | 0.155 |
|  | rs10860209 | C | A | 0.040 | 0.007 | 5.67E-09 | -0.003 | 0.015 | 0.842 |
|  | rs10886863 | T | C | -0.060 | 0.007 | 5.28E-17 | 0.019 | 0.034 | 0.361 |
|  | rs10938398 | G | A | -0.046 | 0.007 | 3.84E-10 | 0.015 | 0.011 | 0.219 |
|  | rs10950550 | T | G | 0.065 | 0.007 | 1.75E-19 | -0.011 | 0.011 | 0.337 |
|  | rs10965248 | T | C | 0.183 | 0.007 | 4.42E-164 | 0.008 | 0.015 | 0.587 |
|  | rs111246699 | G | A | -0.061 | 0.008 | 1.54E-15 | -0.005 | 0.013 | 0.735 |
|  | rs113154802 | C | T | 0.060 | 0.011 | 3.51E-08 | -0.001 | 0.019 | 0.952 |
|  | rs1182444 | A | G | -0.047 | 0.007 | 1.67E-12 | -0.022 | 0.012 | 0.062 |
|  | rs11926494 | G | A | 0.112 | 0.009 | 2.69E-37 | -0.009 | 0.019 | 0.727 |
|  | rs1206684 | G | A | 0.040 | 0.007 | 4.42E-09 | -0.015 | 0.012 | 0.224 |
|  | rs123378 | G | A | 0.053 | 0.008 | 2.22E-10 | 0.014 | 0.012 | 0.267 |
|  | rs1260326 | T | C | -0.063 | 0.007 | 1.01E-21 | 0.008 | 0.011 | 0.514 |
|  | rs12610052 | T | C | -0.046 | 0.008 | 1.97E-09 | 0.005 | 0.040 | 0.717 |
|  | rs12625671 | T | C | -0.066 | 0.007 | 2.25E-21 | -0.001 | 0.016 | 0.584 |
|  | rs1266488 | T | C | 0.044 | 0.007 | 6.29E-10 | -0.075 | 0.035 | 0.057 |
|  | rs12698877 | A | G | -0.067 | 0.007 | 6.96E-22 | -0.001 | 0.013 | 0.942 |
|  | rs12818766 | G | A | -0.054 | 0.009 | 2.46E-10 | 0.016 | 0.015 | 0.308 |
|  | rs13092876 | G | A | -0.126 | 0.007 | 1.91E-66 | 0.000 | 0.012 | 0.991 |
|  | rs13266634 | C | T | 0.116 | 0.007 | 3.72E-67 | 0.015 | 0.012 | 0.204 |
|  | rs1328412 | T | C | 0.097 | 0.015 | 6.41E-11 | 0.011 | 0.022 | 0.720 |
|  | rs1421085 | T | C | -0.130 | 0.009 | 1.55E-48 | -0.005 | 0.011 | 0.684 |
|  | rs1426371 | G | A | 0.048 | 0.007 | 7.76E-12 | 0.000 | 0.013 | 0.931 |
|  | rs1459513 | A | C | -0.046 | 0.007 | 3.73E-11 | 0.024 | 0.024 | 0.272 |
|  | rs147707338 | C | T | -0.087 | 0.014 | 6.47E-10 | 0.013 | 0.019 | 0.482 |
|  | rs1513275 | T | C | -0.057 | 0.009 | 2.76E-11 | 0.000 | 0.013 | 0.893 |
|  | rs16884229 | T | C | -0.097 | 0.007 | 1.15E-43 | 0.014 | 0.014 | 0.312 |
|  | rs17168486 | C | T | -0.064 | 0.007 | 8.23E-22 | -0.013 | 0.014 | 0.321 |
|  | rs1850421 | A | C | 0.044 | 0.007 | 1.41E-09 | 0.000 | 0.012 | 0.971 |
|  | rs2074120 | A | C | 0.041 | 0.007 | 8.38E-09 | -0.020 | 0.011 | 0.076 |
|  | rs2092518 | G | T | 0.046 | 0.007 | 1.39E-10 | 0.001 | 0.011 | 0.941 |
|  | rs2126736 | A | G | 0.038 | 0.007 | 1.84E-08 | -0.001 | 0.011 | 0.920 |
|  | rs2240885 | G | A | -0.042 | 0.007 | 2.79E-09 | -0.004 | 0.014 | 0.826 |
|  | rs2269245 | G | A | 0.054 | 0.009 | 5.40E-10 | -0.027 | 0.014 | 0.059 |
|  | rs2327777 | T | C | 0.050 | 0.007 | 1.06E-13 | -0.027 | 0.011 | 0.016 |
|  | rs2583934 | G | T | -0.058 | 0.007 | 4.95E-16 | -0.001 | 0.015 | 0.905 |
|  | rs261982 | C | T | -0.040 | 0.007 | 3.12E-09 | -0.003 | 0.011 | 0.802 |
|  | rs2706710 | C | T | -0.071 | 0.013 | 1.67E-08 | -0.019 | 0.015 | 0.222 |
|  | rs2796441 | G | A | 0.075 | 0.007 | 1.43E-28 | -0.002 | 0.011 | 0.813 |
|  | rs28599782 | G | A | -0.067 | 0.008 | 4.64E-16 | 0.002 | 0.030 | 0.788 |
|  | rs28691713 | C | T | 0.066 | 0.008 | 1.79E-17 | 0.015 | 0.011 | 0.180 |
|  | rs2908279 | T | G | -0.046 | 0.007 | 8.42E-11 | 0.012 | 0.011 | 0.304 |
|  | rs2980883 | T | G | 0.042 | 0.007 | 3.93E-09 | 0.015 | 0.016 | 0.346 |
|  | rs3094508 | T | C | -0.059 | 0.008 | 1.31E-13 | 0.007 | 0.012 | 0.553 |
|  | rs329122 | G | A | -0.039 | 0.007 | 2.22E-08 | -0.006 | 0.011 | 0.586 |
|  | rs34811727 | G | T | -0.076 | 0.013 | 3.32E-09 | 0.023 | 0.023 | 0.320 |
|  | rs349359 | A | C | -0.043 | 0.008 | 3.05E-08 | -0.020 | 0.017 | 0.173 |
|  | rs35589574 | C | T | -0.171 | 0.011 | 1.58E-55 | 0.016 | 0.013 | 0.237 |
|  | rs3735641 | A | G | -0.069 | 0.008 | 3.62E-19 | -0.016 | 0.012 | 0.164 |
|  | rs3751236 | G | A | 0.067 | 0.007 | 6.58E-21 | 0.017 | 0.014 | 0.234 |
|  | rs3852529 | C | T | -0.099 | 0.010 | 2.07E-24 | 0.009 | 0.020 | 0.663 |
|  | rs3887925 | C | T | -0.040 | 0.007 | 3.12E-09 | 0.005 | 0.011 | 0.638 |
|  | rs391933 | G | A | 0.037 | 0.007 | 1.46E-08 | 0.009 | 0.011 | 0.427 |
|  | rs4273712 | A | G | -0.047 | 0.007 | 2.56E-12 | -0.003 | 0.013 | 0.786 |
|  | rs4499362 | C | T | 0.044 | 0.007 | 1.53E-09 | 0.006 | 0.014 | 0.659 |
|  | rs476828 | T | C | -0.084 | 0.008 | 4.81E-27 | -0.003 | 0.013 | 0.732 |
|  | rs4930974 | C | T | -0.069 | 0.008 | 4.07E-19 | 0.011 | 0.014 | 0.391 |
|  | rs532504 | G | A | -0.055 | 0.008 | 7.39E-12 | -0.010 | 0.014 | 0.519 |
|  | rs55700915 | G | A | -0.040 | 0.007 | 1.50E-08 | -0.016 | 0.014 | 0.258 |
|  | rs58524310 | A | G | -0.047 | 0.007 | 8.41E-11 | -0.033 | 0.016 | 0.036 |
|  | rs58718028 | C | T | -0.073 | 0.007 | 3.35E-25 | -0.001 | 0.013 | 0.909 |
|  | rs6012878 | G | A | 0.041 | 0.007 | 4.32E-09 | -0.021 | 0.011 | 0.062 |
|  | rs60573766 | C | T | 0.044 | 0.007 | 4.30E-10 | -0.013 | 0.016 | 0.423 |
|  | rs61779313 | T | C | -0.060 | 0.009 | 5.59E-11 | -0.027 | 0.013 | 0.035 |
|  | rs61975988 | A | G | 0.040 | 0.007 | 1.97E-09 | -0.005 | 0.013 | 0.742 |
|  | rs62173901 | A | G | 0.123 | 0.020 | 6.58E-10 | 0.013 | 0.017 | 0.376 |
|  | rs62405419 | G | T | -0.044 | 0.008 | 3.79E-09 | 0.008 | 0.017 | 0.685 |
|  | rs633862 | C | T | -0.039 | 0.007 | 1.26E-08 | 0.002 | 0.011 | 0.832 |
|  | rs6416749 | C | T | 0.052 | 0.008 | 3.40E-12 | 0.008 | 0.012 | 0.507 |
|  | rs6731688 | A | C | -0.098 | 0.012 | 2.55E-17 | -0.003 | 0.015 | 0.963 |
|  | rs703980 | G | A | 0.060 | 0.007 | 6.55E-19 | -0.005 | 0.011 | 0.640 |
|  | rs7107784 | G | A | 0.086 | 0.012 | 2.60E-13 | 0.003 | 0.013 | 0.869 |
|  | rs7109575 | G | A | 0.141 | 0.015 | 5.46E-21 | -0.010 | 0.016 | 0.664 |
|  | rs7250869 | T | C | 0.056 | 0.007 | 2.29E-16 | -0.011 | 0.012 | 0.363 |
|  | rs7304270 | C | T | 0.068 | 0.010 | 1.04E-12 | 0.086 | 0.058 | 0.160 |
|  | rs73085586 | G | A | 0.043 | 0.007 | 1.66E-09 | -0.017 | 0.014 | 0.233 |
|  | rs7313668 | G | T | -0.045 | 0.007 | 4.91E-11 | -0.003 | 0.011 | 0.755 |
|  | rs73347525 | A | G | 0.059 | 0.009 | 7.46E-11 | -0.012 | 0.015 | 0.438 |
|  | rs742762 | A | C | 0.075 | 0.008 | 1.79E-22 | -0.011 | 0.018 | 0.546 |
|  | rs7501939 | T | C | 0.120 | 0.007 | 1.60E-60 | -0.014 | 0.012 | 0.218 |
|  | rs75990271 | T | C | 0.066 | 0.010 | 3.22E-11 | -0.003 | 0.025 | 0.938 |
|  | rs7656416 | C | T | 0.100 | 0.007 | 9.01E-42 | 0.000 | 0.032 | 0.939 |
|  | rs76704029 | T | C | 0.055 | 0.010 | 3.39E-08 | -0.011 | 0.026 | 0.643 |
|  | rs76878791 | A | G | -0.053 | 0.007 | 2.02E-13 | 0.064 | 0.048 | 0.169 |
|  | rs77789961 | C | T | -0.047 | 0.008 | 1.92E-08 | -0.013 | 0.041 | 0.937 |
|  | rs7787720 | C | T | -0.054 | 0.007 | 2.25E-15 | 0.005 | 0.011 | 0.680 |
|  | rs7901695 | T | C | -0.275 | 0.017 | 8.18E-62 | -0.003 | 0.012 | 0.824 |
|  | rs80196932 | T | C | 0.060 | 0.008 | 7.57E-13 | -0.010 | 0.016 | 0.517 |
|  | rs8026714 | G | A | -0.066 | 0.007 | 1.06E-22 | -0.005 | 0.016 | 0.628 |
|  | rs8043085 | G | T | -0.052 | 0.007 | 2.06E-14 | -0.002 | 0.013 | 0.813 |
|  | rs896852 | G | T | 0.042 | 0.007 | 6.42E-09 | 0.000 | 0.011 | 0.983 |
|  | rs9316706 | A | G | 0.041 | 0.007 | 3.33E-09 | 0.006 | 0.015 | 0.674 |
|  | rs9350271 | G | A | -0.193 | 0.007 | 4.95E-183 | 0.009 | 0.012 | 0.429 |
|  | rs9379084 | G | A | 0.072 | 0.009 | 2.20E-14 | -0.012 | 0.018 | 0.548 |
|  | rs9390022 | T | C | 0.048 | 0.008 | 6.35E-09 | 0.008 | 0.012 | 0.529 |
|  | rs9461650 | G | A | 0.060 | 0.009 | 1.99E-12 | -0.032 | 0.015 | 0.042 |
|  | rs9523295 | G | A | 0.078 | 0.009 | 7.24E-18 | 0.032 | 0.013 | 0.012 |
|  | rs9788635 | C | T | 0.058 | 0.008 | 9.43E-14 | 0.005 | 0.017 | 0.695 |
|  | rs9948462 | C | T | -0.047 | 0.008 | 8.70E-10 | 0.020 | 0.012 | 0.095 |
| *Verrucomicrobiaceae* | rs10011838 | G | A | 0.073 | 0.007 | 1.43E-27 | -0.006 | 0.014 | 0.674 |
|  | rs1016565 | G | A | -0.038 | 0.007 | 2.18E-08 | -0.013 | 0.014 | 0.378 |
|  | rs10507349 | G | A | 0.064 | 0.007 | 1.69E-21 | 0.011 | 0.015 | 0.436 |
|  | rs10852123 | A | C | 0.060 | 0.008 | 8.38E-13 | 0.009 | 0.015 | 0.563 |
|  | rs10860209 | C | A | 0.040 | 0.007 | 5.67E-09 | 0.011 | 0.018 | 0.433 |
|  | rs10938398 | G | A | -0.046 | 0.007 | 3.84E-10 | -0.014 | 0.013 | 0.287 |
|  | rs10950550 | T | G | 0.065 | 0.007 | 1.75E-19 | 0.001 | 0.013 | 0.910 |
|  | rs10965248 | T | C | 0.183 | 0.007 | 4.42E-164 | 0.027 | 0.017 | 0.108 |
|  | rs111246699 | G | A | -0.061 | 0.008 | 1.54E-15 | 0.017 | 0.015 | 0.284 |
|  | rs113154802 | C | T | 0.060 | 0.011 | 3.51E-08 | -0.013 | 0.022 | 0.526 |
|  | rs1182444 | A | G | -0.047 | 0.007 | 1.67E-12 | -0.009 | 0.014 | 0.506 |
|  | rs11926494 | G | A | 0.112 | 0.009 | 2.69E-37 | -0.018 | 0.023 | 0.480 |
|  | rs1206684 | G | A | 0.040 | 0.007 | 4.42E-09 | -0.027 | 0.013 | 0.047 |
|  | rs123378 | G | A | 0.053 | 0.008 | 2.22E-10 | 0.000 | 0.014 | 0.992 |
|  | rs1260326 | T | C | -0.063 | 0.007 | 1.01E-21 | 0.001 | 0.013 | 0.958 |
|  | rs12625671 | T | C | -0.066 | 0.007 | 2.25E-21 | 0.012 | 0.019 | 0.415 |
|  | rs12698877 | A | G | -0.067 | 0.007 | 6.96E-22 | 0.003 | 0.015 | 0.802 |
|  | rs12818766 | G | A | -0.054 | 0.009 | 2.46E-10 | -0.011 | 0.017 | 0.564 |
|  | rs13092876 | G | A | -0.126 | 0.007 | 1.91E-66 | -0.022 | 0.014 | 0.119 |
|  | rs13266634 | C | T | 0.116 | 0.007 | 3.72E-67 | 0.033 | 0.014 | 0.017 |
|  | rs1328412 | T | C | 0.097 | 0.015 | 6.41E-11 | 0.029 | 0.025 | 0.253 |
|  | rs1421085 | T | C | -0.130 | 0.009 | 1.55E-48 | -0.012 | 0.013 | 0.401 |
|  | rs1426371 | G | A | 0.048 | 0.007 | 7.76E-12 | 0.019 | 0.015 | 0.205 |
|  | rs1459513 | A | C | -0.046 | 0.007 | 3.73E-11 | -0.003 | 0.029 | 0.727 |
|  | rs147707338 | C | T | -0.087 | 0.014 | 6.47E-10 | 0.011 | 0.022 | 0.575 |
|  | rs1513275 | T | C | -0.057 | 0.009 | 2.76E-11 | 0.006 | 0.016 | 0.856 |
|  | rs16884229 | T | C | -0.097 | 0.007 | 1.15E-43 | -0.024 | 0.016 | 0.121 |
|  | rs17168486 | C | T | -0.064 | 0.007 | 8.23E-22 | 0.010 | 0.017 | 0.572 |
|  | rs1850421 | A | C | 0.044 | 0.007 | 1.41E-09 | 0.000 | 0.014 | 0.966 |
|  | rs2074120 | A | C | 0.041 | 0.007 | 8.38E-09 | -0.004 | 0.013 | 0.728 |
|  | rs2092518 | G | T | 0.046 | 0.007 | 1.39E-10 | -0.003 | 0.013 | 0.804 |
|  | rs2126736 | A | G | 0.038 | 0.007 | 1.84E-08 | -0.014 | 0.013 | 0.277 |
|  | rs2240885 | G | A | -0.042 | 0.007 | 2.79E-09 | 0.000 | 0.016 | 0.946 |
|  | rs2269245 | G | A | 0.054 | 0.009 | 5.40E-10 | 0.015 | 0.016 | 0.303 |
|  | rs2327777 | T | C | 0.050 | 0.007 | 1.06E-13 | 0.007 | 0.013 | 0.574 |
|  | rs2583934 | G | T | -0.058 | 0.007 | 4.95E-16 | 0.032 | 0.018 | 0.084 |
|  | rs261982 | C | T | -0.040 | 0.007 | 3.12E-09 | 0.015 | 0.013 | 0.261 |
|  | rs2706710 | C | T | -0.071 | 0.013 | 1.67E-08 | -0.016 | 0.017 | 0.347 |
|  | rs2796441 | G | A | 0.075 | 0.007 | 1.43E-28 | -0.009 | 0.013 | 0.471 |
|  | rs28599782 | G | A | -0.067 | 0.008 | 4.64E-16 | 0.015 | 0.039 | 0.837 |
|  | rs28691713 | C | T | 0.066 | 0.008 | 1.79E-17 | 0.002 | 0.013 | 0.879 |
|  | rs2908279 | T | G | -0.046 | 0.007 | 8.42E-11 | -0.015 | 0.013 | 0.257 |
|  | rs2980883 | T | G | 0.042 | 0.007 | 3.93E-09 | 0.009 | 0.018 | 0.643 |
|  | rs3094508 | T | C | -0.059 | 0.008 | 1.31E-13 | -0.005 | 0.013 | 0.681 |
|  | rs329122 | G | A | -0.039 | 0.007 | 2.22E-08 | 0.005 | 0.013 | 0.684 |
|  | rs34811727 | G | T | -0.076 | 0.013 | 3.32E-09 | 0.067 | 0.026 | 0.012 |
|  | rs349359 | A | C | -0.043 | 0.008 | 3.05E-08 | -0.014 | 0.020 | 0.461 |
|  | rs35589574 | C | T | -0.171 | 0.011 | 1.58E-55 | -0.011 | 0.015 | 0.509 |
|  | rs3735641 | A | G | -0.069 | 0.008 | 3.62E-19 | -0.018 | 0.013 | 0.187 |
|  | rs3751236 | G | A | 0.067 | 0.007 | 6.58E-21 | 0.005 | 0.016 | 0.806 |
|  | rs3852529 | C | T | -0.099 | 0.010 | 2.07E-24 | 0.001 | 0.023 | 0.916 |
|  | rs3887925 | C | T | -0.040 | 0.007 | 3.12E-09 | -0.006 | 0.013 | 0.639 |
|  | rs391933 | G | A | 0.037 | 0.007 | 1.46E-08 | -0.016 | 0.013 | 0.221 |
|  | rs4273712 | A | G | -0.047 | 0.007 | 2.56E-12 | 0.024 | 0.015 | 0.118 |
|  | rs4499362 | C | T | 0.044 | 0.007 | 1.53E-09 | 0.000 | 0.016 | 0.991 |
|  | rs476828 | T | C | -0.084 | 0.008 | 4.81E-27 | -0.012 | 0.015 | 0.407 |
|  | rs4930974 | C | T | -0.069 | 0.008 | 4.07E-19 | 0.008 | 0.016 | 0.638 |
|  | rs532504 | G | A | -0.055 | 0.008 | 7.39E-12 | 0.030 | 0.016 | 0.065 |
|  | rs55700915 | G | A | -0.040 | 0.007 | 1.50E-08 | -0.004 | 0.016 | 0.852 |
|  | rs58524310 | A | G | -0.047 | 0.007 | 8.41E-11 | -0.007 | 0.018 | 0.743 |
|  | rs58718028 | C | T | -0.073 | 0.007 | 3.35E-25 | -0.022 | 0.015 | 0.147 |
|  | rs6012878 | G | A | 0.041 | 0.007 | 4.32E-09 | -0.010 | 0.013 | 0.440 |
|  | rs60573766 | C | T | 0.044 | 0.007 | 4.30E-10 | -0.028 | 0.019 | 0.163 |
|  | rs61779313 | T | C | -0.060 | 0.009 | 5.59E-11 | 0.001 | 0.015 | 0.917 |
|  | rs61975988 | A | G | 0.040 | 0.007 | 1.97E-09 | -0.002 | 0.015 | 0.875 |
|  | rs62173901 | A | G | 0.123 | 0.020 | 6.58E-10 | 0.011 | 0.019 | 0.579 |
|  | rs62405419 | G | T | -0.044 | 0.008 | 3.79E-09 | 0.006 | 0.020 | 0.538 |
|  | rs633862 | C | T | -0.039 | 0.007 | 1.26E-08 | -0.003 | 0.013 | 0.793 |
|  | rs6416749 | C | T | 0.052 | 0.008 | 3.40E-12 | 0.003 | 0.014 | 0.793 |
|  | rs6731688 | A | C | -0.098 | 0.012 | 2.55E-17 | 0.039 | 0.017 | 0.017 |
|  | rs703980 | G | A | 0.060 | 0.007 | 6.55E-19 | -0.006 | 0.013 | 0.675 |
|  | rs7107784 | G | A | 0.086 | 0.012 | 2.60E-13 | -0.026 | 0.014 | 0.066 |
|  | rs7109575 | G | A | 0.141 | 0.015 | 5.46E-21 | 0.017 | 0.018 | 0.270 |
|  | rs7250869 | T | C | 0.056 | 0.007 | 2.29E-16 | 0.004 | 0.014 | 0.806 |
|  | rs73085586 | G | A | 0.043 | 0.007 | 1.66E-09 | -0.003 | 0.016 | 0.821 |
|  | rs7313668 | G | T | -0.045 | 0.007 | 4.91E-11 | -0.006 | 0.013 | 0.622 |
|  | rs73347525 | A | G | 0.059 | 0.009 | 7.46E-11 | -0.020 | 0.017 | 0.256 |
|  | rs742762 | A | C | 0.075 | 0.008 | 1.79E-22 | -0.015 | 0.021 | 0.631 |
|  | rs7501939 | T | C | 0.120 | 0.007 | 1.60E-60 | 0.016 | 0.013 | 0.240 |
|  | rs75990271 | T | C | 0.066 | 0.010 | 3.22E-11 | 0.042 | 0.029 | 0.123 |
|  | rs76704029 | T | C | 0.055 | 0.010 | 3.39E-08 | -0.027 | 0.030 | 0.268 |
|  | rs7787720 | C | T | -0.054 | 0.007 | 2.25E-15 | 0.013 | 0.013 | 0.322 |
|  | rs7901695 | T | C | -0.275 | 0.017 | 8.18E-62 | 0.013 | 0.014 | 0.351 |
|  | rs80196932 | T | C | 0.060 | 0.008 | 7.57E-13 | -0.002 | 0.018 | 0.963 |
|  | rs8026714 | G | A | -0.066 | 0.007 | 1.06E-22 | 0.000 | 0.019 | 0.904 |
|  | rs8043085 | G | T | -0.052 | 0.007 | 2.06E-14 | -0.006 | 0.015 | 0.716 |
|  | rs896852 | G | T | 0.042 | 0.007 | 6.42E-09 | 0.013 | 0.013 | 0.332 |
|  | rs9316706 | A | G | 0.041 | 0.007 | 3.33E-09 | -0.016 | 0.017 | 0.318 |
|  | rs9350271 | G | A | -0.193 | 0.007 | 4.95E-183 | -0.004 | 0.014 | 0.739 |
|  | rs9379084 | G | A | 0.072 | 0.009 | 2.20E-14 | 0.003 | 0.021 | 0.970 |
|  | rs9390022 | T | C | 0.048 | 0.008 | 6.35E-09 | -0.001 | 0.014 | 0.973 |
|  | rs9461650 | G | A | 0.060 | 0.009 | 1.99E-12 | -0.008 | 0.018 | 0.677 |
|  | rs9523295 | G | A | 0.078 | 0.009 | 7.24E-18 | -0.007 | 0.015 | 0.599 |
|  | rs9788635 | C | T | 0.058 | 0.008 | 9.43E-14 | -0.031 | 0.020 | 0.114 |
|  | rs9948462 | C | T | -0.047 | 0.008 | 8.70E-10 | -0.004 | 0.013 | 0.778 |

Abbreviations: SNP, single nucleotide polymorphism; IVs, instrumental variables; T2DM, type 2 diabetes mellitus; GWAS, genome-wide association study.
