# Supplementary material for: Genetically Predicted Causality of 28 Gut Microbiome Families and Type 2 Diabetes Mellitus Risk
Source: Front Endocrinol (Lausanne). 2022 Feb 3;13:780133. doi: 10.3389/fendo.2022.780133 (PMC8851667; doi:10.3389/fendo.2022.780133)
Supplement: Supplementary file 9 [file Table_7.docx]

| **Supplementary Table 7. MR estimates of IVs for T2DM and gut microbiome (European)** | | | | | | | | | | | | | | | | |
| --- | --- | --- | --- | --- | --- | --- | --- | --- | --- | --- | --- | --- | --- | --- | --- | --- |
| **Outcome** | **Nsnp** | **Methods** | **Beta** | **SE** | **OR (95% CI)** | ***P* value** | **FDR *P* value** | **Horizontal pleiotropy** | | | | | | | **Heterogeneity** | |
|  |  |  |  |  |  |  |  | **MR-Egger regression** | | | **MR-PRESSO** | | | | **Cochran’s *Q*** | ***P* value** |
|  |  |  |  |  |  |  |  | **Egger intercept** | **SE** | ***P* value** | **Global test *P* value** | **Outliers** | **OR (95% CI)** | ***P* value** |  |  |
| *Acidaminococcaceae* | 107 | IVW | -0.03 | 0.02 | 0.97 (0.94-1.01) | 0.186 | 0.772 | 0.00 | 0.00 | 0.846 | 0.906 | - | - | - | 85.96 | 0.923 |
|  |  | MR Egger | -0.02 | 0.04 | 0.98 (0.90-1.07) | 0.684 | 0.990 |  |  |  |  |  |  |  |  |  |
|  |  | Weighted median | -0.02 | 0.04 | 0.98 (0.91-1.05) | 0.590 | 0.879 |  |  |  |  |  |  |  |  |  |
| *Actinomycetaceae* | 107 | IVW | 0.03 | 0.03 | 1.03 (0.98-1.08) | 0.223 | 0.781 | 0.00 | 0.00 | 0.613 | 0.219 | - | - | - | 117.47 | 0.210 |
|  |  | MR Egger | 0.00 | 0.06 | 1.00 (0.90-1.13) | 0.936 | 0.990 |  |  |  |  |  |  |  |  |  |
|  |  | Weighted median | 0.02 | 0.05 | 1.02 (0.93-1.12) | 0.622 | 0.879 |  |  |  |  |  |  |  |  |  |
| *Alcaligenaceae* | 107 | IVW | 0.01 | 0.02 | 1.01 (0.97-1.04) | 0.756 | 0.956 | 0.00 | 0.00 | 0.255 | 0.956 | - | - | - | 82.84 | 0.953 |
|  |  | MR Egger | -0.03 | 0.04 | 0.97 (0.90-1.04) | 0.375 | 0.990 |  |  |  |  |  |  |  |  |  |
|  |  | Weighted median | 0.01 | 0.03 | 0.99 (0.94-1.04) | 0.589 | 0.879 |  |  |  |  |  |  |  |  |  |
| *Bacteroidaceae* | 108 | IVW | -0.03 | 0.02 | 0.97 (0.94-0.99) | 0.042 | 0.588 | 0.00 | 0.00 | 0.203 | 0.404 | - | - | - | 109.52 | 0.414 |
|  |  | MR Egger | -0.07 | 0.04 | 0.93 (0.86-0.99) | 0.043 | 0.770 |  |  |  |  |  |  |  |  |  |
|  |  | Weighted median | -0.08 | 0.03 | 0.92 (0.88-0.97) | 0.002 | 0.056 |  |  |  |  |  |  |  |  |  |
| *Bacteroidales_S24-7* | 107 | IVW | 0.01 | 0.03 | 1.01 (0.96-1.06) | 0.765 | 0.956 | 0.00 | 0.00 | 0.830 | 0.118 | - | - | - | 122.11 | 0.136 |
|  |  | MR Egger | 0.00 | 0.06 | 1.00 (0.89-1.12) | 0.950 | 0.990 |  |  |  |  |  |  |  |  |  |
|  |  | Weighted median | -0.02 | 0.05 | 0.98 (0.89-1.07) | 0.616 | 0.879 |  |  |  |  |  |  |  |  |  |
| *Bifidobacteriaceae* | 50 | IVW | -0.02 | 0.04 | 0.98 (0.92-1.06) | 0.659 | 0.772 | -0.01 | 0.01 | 0.012 | <0.001 | rs2796441, rs7903146 | 0.93 (0.86-1.01) | 0.091 | 114.43 | <0.001 |
|  |  | MR Egger | 0.13 | 0.07 | 1.14 (1.00-1.30) | 0.052 | 0.770 |  |  |  |  |  | 1.01 (0.79-1.29) | 0.963 |  |  |
|  |  | Weighted median | -0.01 | 0.04 | 0.99 (0.92-1.08) | 0.868 | 0.224 |  |  |  |  |  | 0.88 (0.80-0.98) | 0.016 |  |  |
| *Christensenellaceae* | 108 | IVW | -0.01 | 0.02 | 0.99 (0.95-1.02) | 0.447 | 0.890 | 0.00 | 0.00 | 0.593 | 0.313 | - | - | - | 116.20 | 0.256 |
|  |  | MR Egger | 0.01 | 0.04 | 1.01 (0.93-1.09) | 0.883 | 0.990 |  |  |  |  |  |  |  |  |  |
|  |  | Weighted median | -0.01 | 0.03 | 0.99 (0.93-1.05) | 0.691 | 0.879 |  |  |  |  |  |  |  |  |  |
| *Clostridiaceae_1* | 107 | IVW | 0.00 | 0.02 | 1.00 (0.96-1.04) | 0.946 | 0.961 | 0.00 | 0.00 | 0.527 | 0.097 | - | - | - | 126.96 | 0.081 |
|  |  | MR Egger | 0.03 | 0.04 | 1.03 (0.94-1.12) | 0.550 | 0.990 |  |  |  |  |  |  |  |  |  |
|  |  | Weighted median | 0.01 | 0.04 | 1.01 (0.94-1.08) | 0.752 | 0.915 |  |  |  |  |  |  |  |  |  |
| *Coriobacteriaceae* | 108 | IVW | 0.01 | 0.02 | 1.01 (0.98-1.05) | 0.553 | 0.911 | 0.00 | 0.00 | 0.602 | 0.159 | - | - | - | 122.85 | 0.140 |
|  |  | MR Egger | 0.03 | 0.04 | 1.03 (0.95-1.11) | 0.467 | 0.990 |  |  |  |  |  |  |  |  |  |
|  |  | Weighted median | 0.02 | 0.03 | 1.02 (0.97-1.08) | 0.403 | 0.879 |  |  |  |  |  |  |  |  |  |
| *Defluviitaleaceae* | 107 | IVW | 0.00 | 0.02 | 1.00 (0.96-1.05) | 0.961 | 0.961 | 0.00 | 0.00 | 0.638 | 0.677 | - | - | - | 97.59 | 0.708 |
|  |  | MR Egger | 0.02 | 0.05 | 1.02 (0.92-1.14) | 0.657 | 0.990 |  |  |  |  |  |  |  |  |  |
|  |  | Weighted median | 0.02 | 0.04 | 1.02 (0.94-1.11) | 0.612 | 0.879 |  |  |  |  |  |  |  |  |  |
| *Desulfovibrionaceae* | 107 | IVW | 0.02 | 0.02 | 1.02 (0.99-1.06) | 0.193 | 0.772 | -4.64 | 0.00 | 0.987 | 0.723 | - | - | - | 97.31 | 0.715 |
|  |  | MR Egger | 0.02 | 0.04 | 1.02 (0.95-1.11) | 0.558 | 0.990 |  |  |  |  |  |  |  |  |  |
|  |  | Weighted median | 0.03 | 0.03 | 1.03 (0.97-1.10) | 0.301 | 0.879 |  |  |  |  |  |  |  |  |  |
| *Enterobacteriaceae* | 107 | IVW | -0.01 | 0.02 | 0.99 (0.96-1.03) | 0.738 | 0.956 | 0.00 | 0.00 | 0.760 | 0.281 | - | - | - | 114.33 | 0.273 |
|  |  | MR Egger | 0.01 | 0.04 | 1.01 (0.92-1.10) | 0.898 | 0.990 |  |  |  |  |  |  |  |  |  |
|  |  | Weighted median | 0.01 | 0.03 | 1.01 (0.96-1.08) | 0.638 | 0.879 |  |  |  |  |  |  |  |  |  |
| *Erysipelotrichaceae* | 108 | IVW | -0.01 | 0.02 | 0.99 (0.95-1.02) | 0.438 | 0.890 | 0.00 | 0.00 | 0.964 | 0.192 | - | - | - | 120.82 | 0.171 |
|  |  | MR Egger | -0.01 | 0.04 | 0.99 (0.92-1.07) | 0.766 | 0.990 |  |  |  |  |  |  |  |  |  |
|  |  | Weighted median | -0.01 | 0.03 | 0.99 (0.94-1.04) | 0.671 | 0.879 |  |  |  |  |  |  |  |  |  |
| *Lachnospiraceae* | 108 | IVW | 0.00 | 0.02 | 1.00 (0.96-1.03) | 0.886 | 0.961 | 0.00 | 0.00 | 0.955 | 0.160 | - | - | - | 122.38 | 0.147 |
|  |  | MR Egger | 0.00 | 0.04 | 1.00 (0.93-1.08) | 0.990 | 0.990 |  |  |  |  |  |  |  |  |  |
|  |  | Weighted median | 0.00 | 0.03 | 1.00 (0.95-1.07) | 0.892 | 0.945 |  |  |  |  |  |  |  |  |  |
| *Lactobacillaceae* | 107 | IVW | -0.02 | 0.03 | 0.98 (0.94-1.03) | 0.526 | 0.911 | -0.01 | 0.00 | 0.223 | 0.382 | - | - | - | 107.20 | 0.449 |
|  |  | MR Egger | 0.05 | 0.06 | 1.05 (0.94-1.18) | 0.414 | 0.990 |  |  |  |  |  |  |  |  |  |
|  |  | Weighted median | 0.01 | 0.04 | 1.01 (0.93-1.09) | 0.865 | 0.945 |  |  |  |  |  |  |  |  |  |
| *Methanobacteriaceae* | 106 | IVW | -0.01 | 0.04 | 0.99 (0.92-1.06) | 0.819 | 0.956 | 0.01 | 0.01 | 0.285 | 0.457 | - | - | - | 106.66 | 0.436 |
|  |  | MR Egger | -0.09 | 0.08 | 0.92 (0.78-1.07) | 0.290 | 0.990 |  |  |  |  |  |  |  |  |  |
|  |  | Weighted median | -0.06 | 0.07 | 0.95 (0.83-1.08) | 0.412 | 0.879 |  |  |  |  |  |  |  |  |  |
| *Oxalobacteraceae* | 107 | IVW | -0.07 | 0.03 | 0.94 (0.88-0.99) | 0.030 | 0.588 | 0.00 | 0.01 | 0.351 | 0.301 | - | - | - | 111.98 | 0.327 |
|  |  | MR Egger | -0.13 | 0.07 | 0.88 (0.77-1.01) | 0.075 | 0.770 |  |  |  |  |  |  |  |  |  |
|  |  | Weighted median | -0.07 | 0.05 | 0.94 (0.85-1.03) | 0.189 | 0.879 |  |  |  |  |  |  |  |  |  |
| *Pasteurellaceae* | 107 | IVW | 0.01 | 0.02 | 1.01 (0.97-1.06) | 0.629 | 0.956 | 0.00 | 0.00 | 0.832 | 0.612 | - | - | - | 101.25 | 0.612 |
|  |  | MR Egger | 0.00 | 0.05 | 1.00 (0.91-1.11) | 0.983 | 0.990 |  |  |  |  |  |  |  |  |  |
|  |  | Weighted median | -0.01 | 0.04 | 0.99 (0.91-1.07) | 0.788 | 0.919 |  |  |  |  |  |  |  |  |  |
| *Peptococcaceae* | 107 | IVW | -0.02 | 0.02 | 0.98 (0.94-1.03) | 0.477 | 0.890 | 0.00 | 0.00 | 0.974 | 0.214 | - | - | - | 117.56 | 0.208 |
|  |  | MR Egger | -0.01 | 0.05 | 0.99 (0.89-1.09) | 0.778 | 0.990 |  |  |  |  |  |  |  |  |  |
|  |  | Weighted median | -0.02 | 0.04 | 0.98 (0.91-1.06) | 0.684 | 0.879 |  |  |  |  |  |  |  |  |  |
| *Peptostreptococcaceae* | 108 | IVW | -0.01 | 0.02 | 0.99 (0.96-1.03) | 0.693 | 0.956 | 0.00 | 0.00 | 0.490 | 0.564 | - | - | - | 103.72 | 0.572 |
|  |  | MR Egger | 0.02 | 0.04 | 1.02 (0.94-1.10) | 0.655 | 0.990 |  |  |  |  |  |  |  |  |  |
|  |  | Weighted median | 0.02 | 0.03 | 1.03 (0.97-1.08) | 0.353 | 0.879 |  |  |  |  |  |  |  |  |  |
| *Porphyromonadaceae* | 108 | IVW | -0.01 | 0.02 | 0.99 ( 0.95-1.02) | 0.352 | 0.890 | 0.00 | 0.00 | 0.423 | 0.939 | - | - | - | 85.39 | 0.939 |
|  |  | MR Egger | -0.04 | 0.04 | 0.96 (0.89-1.03) | 0.260 | 0.990 |  |  |  |  |  |  |  |  |  |
|  |  | Weighted median | -0.03 | 0.03 | 0.97 (0.92-1.02) | 0.235 | 0.879 |  |  |  |  |  |  |  |  |  |
| *Prevotellaceae* | 107 | IVW | 0.00 | 0.02 | 1.00 (0.97-1.04) | 0.918 | 0.961 | 0.00 | 0.00 | 0.551 | 0.452 | - | - | - | 107.24 | 0.448 |
|  |  | MR Egger | 0.02 | 0.04 | 1.02 (0.95-1.11) | 0.562 | 0.990 |  |  |  |  |  |  |  |  |  |
|  |  | Weighted median | 0.00 | 0.03 | 1.00 (0.94-1.07) | 0.945 | 0.945 |  |  |  |  |  |  |  |  |  |
| *Rhodospirillaceae* | 107 | IVW | 0.01 | 0.03 | 1.01 (0.95-1.06) | 0.814 | 0.956 | 0.00 | 0.00 | 0.846 | 0.013^a^ | - | - | - | 142.92 | 0.010 |
|  |  | MR Egger | 0.02 | 0.06 | 1.02 (0.90-1.15) | 0.782 | 0.990 |  |  |  |  |  |  |  |  |  |
|  |  | Weighted median | -0.04 | 0.04 | 0.96 (0.89-1.04) | 0.342 | 0.879 |  |  |  |  |  |  |  |  |  |
| *Rikenellaceae* | 108 | IVW | -0.02 | 0.02 | 0.98 (0.95-1.01) | 0.188 | 0.772 | 0.00 | 0.00 | 0.542 | 0.479 | - | - | - | 107.31 | 0.473 |
|  |  | MR Egger | -0.04 | 0.04 | 0.96 (0.89-1.03) | 0.262 | 0.990 |  |  |  |  |  |  |  |  |  |
|  |  | Weighted median | -0.02 | 0.03 | 0.98 (0.92-1.04) | 0.422 | 0.879 |  |  |  |  |  |  |  |  |  |
| *Ruminococcaceae* | 108 | IVW | 0.02 | 0.02 | 1.02 (0.99-1.06) | 0.142 | 0.772 | 0.00 | 0.00 | 0.321 | 0.435 | - | - | - | 109.61 | 0.412 |
|  |  | MR Egger | -0.01 | 0.04 | 0.99 (0.92-1.06) | 0.804 | 0.990 |  |  |  |  |  |  |  |  |  |
|  |  | Weighted median | 0.00 | 0.03 | 1.00 (0.94-1.05) | 0.913 | 0.945 |  |  |  |  |  |  |  |  |  |
| *Streptococcaceae* | 108 | IVW | 0.01 | 0.02 | 1.01 (0.98-1.05) | 0.380 | 0.890 | 0.00 | 0.00 | 0.459 | 0.933 | - | - | - | 86.29 | 0.930 |
|  |  | MR Egger | 0.04 | 0.04 | 1.04 (0.97-1.12) | 0.294 | 0.990 |  |  |  |  |  |  |  |  |  |
|  |  | Weighted median | 0.04 | 0.03 | 1.04 (0.98-1.10) | 0.206 | 0.879 |  |  |  |  |  |  |  |  |  |
| *Veillonellaceae* | 107 | IVW | -0.01 | 0.02 | 0.99 (0.95-1.02) | 0.439 | 0.890 | 0.00 | 0.00 | 0.376 | 0.078 | - | - | - | 126.13 | 0.089 |
|  |  | MR Egger | 0.02 | 0.04 | 1.02 (0.94-1.11) | 0.647 | 0.990 |  |  |  |  |  |  |  |  |  |
|  |  | Weighted median | 0.02 | 0.03 | 1.02 (0.96-1.08) | 0.567 | 0.879 |  |  |  |  |  |  |  |  |  |
| *Verrucomicrobiaceae* | 107 | IVW | -0.02 | 0.02 | 0.98 (0.94-1.02) | 0.348 | 0.890 | 0.00 | 0.00 | 0.404 | 0.318 | - | - | - | 113.50 | 0.291 |
|  |  | MR Egger | 0.02 | 0.05 | 1.02 (0.93-1.11) | 0.735 | 0.990 |  |  |  |  |  |  |  |  |  |
|  |  | Weighted median | -0.02 | 0.04 | 0.98 (0.91-1.05) | 0.524 | 0.879 |  |  |  |  |  |  |  |  |  |

a. MR-PRESSO could not identify significant outliers

Abbreviations: MR, Mendelian randomization; SNP, single nucleotide polymorphism; IVW, inverse variance weighted; IVs, instrumental variables; FDR, false discovery rate; T2DM, type 2 diabetes mellitus; OR, odds ratio; MR-PRESSO, Mendelian randomization pleiotropy residual sum and outlier.
