# Supplementary material for: Genetically Predicted Causality of 28 Gut Microbiome Families and Type 2 Diabetes Mellitus Risk
Source: Front Endocrinol (Lausanne). 2022 Feb 3;13:780133. doi: 10.3389/fendo.2022.780133 (PMC8851667; doi:10.3389/fendo.2022.780133)
Supplement: Supplementary file 10 [file Table_8.docx]

| **Supplementary Table 8. MR estimates of IVs for T2DM and gut microbiome (Asian)** | | | | | | | | | | | | | | | | |
| --- | --- | --- | --- | --- | --- | --- | --- | --- | --- | --- | --- | --- | --- | --- | --- | --- |
| **Outcome** | **Nsnp** | **Methods** | **Beta** | **SE** | **OR (95% CI)** | ***P* value** | **FDR *P* value** | **Horizontal pleiotropy** | | | | | | | **Heterogeneity** | |
|  |  |  |  |  |  |  |  | **MR-Egger regression** | | | **MR-PRESSO** | | | | **Cochran's *Q*** | ***P* value** |
|  |  |  |  |  |  |  |  | **Egger intercept** | **SE** | ***P* value** | **Global test *P* value** | **Outliers** | **OR (95% CI)** | ***P* value** |  |  |
| *Acidaminococcaceae* | 97 | IVW | -0.02 | 0.02 | 0.98 (0.94-1.02) | 0.329 | 0.946 | 0.00 | 0.00 | 0.955 | 0.502 | - | - | - | 95.46 | 0.496 |
|  |  | MR Egger | -0.02 | 0.04 | 0.98 (0.91-1.06) | 0.659 | 0.986 |  |  |  |  |  |  |  |  |  |
|  |  | Weighted median | -0.03 | 0.03 | 0.97 (0.91-1.04) | 0.388 | 0.821 |  |  |  |  |  |  |  |  |  |
| *Actinomycetaceae* | 94 | IVW | 0.01 | 0.03 | 1.01 (0.96-1.06) | 0.758 | 0.946 | 0.00 | 0.00 | 0.971 | 0.807 | - | - | - | 81.16 | 0.805 |
|  |  | MR Egger | 0.01 | 0.05 | 1.01 (0.92-1.11) | 0.852 | 0.986 |  |  |  |  |  |  |  |  |  |
|  |  | Weighted median | 0.03 | 0.04 | 1.03 (0.94-1.12) | 0.561 | 0.821 |  |  |  |  |  |  |  |  |  |
| *Alcaligenaceae* | 102 | IVW | -0.01 | 0.02 | 0.99 (0.96-1.03) | 0.625 | 0.946 | 0.00 | 0.00 | 0.860 | 0.095 | - | - | - | 120.57 | 0.090 |
|  |  | MR Egger | -0.01 | 0.04 | 0.99 (0.92-1.06) | 0.692 | 0.986 |  |  |  |  |  |  |  |  |  |
|  |  | Weighted median | -0.02 | 0.03 | 0.98 (0.92-1.03) | 0.411 | 0.821 |  |  |  |  |  |  |  |  |  |
| *Bacteroidaceae* | 102 | IVW | 0.00 | 0.02 | 1.00 (0.96-1.03) | 0.773 | 0.946 | 0.00 | 0.00 | 0.948 | 0.383 | - | - | - | 103.77 | 0.405 |
|  |  | MR Egger | -0.01 | 0.03 | 0.99 (0.93-1.06) | 0.841 | 0.986 |  |  |  |  |  |  |  |  |  |
|  |  | Weighted median | -0.01 | 0.03 | 0.99 (0.94-1.04) | 0.655 | 0.821 |  |  |  |  |  |  |  |  |  |
| *Bacteroidales_S24-7* | 94 | IVW | 0.01 | 0.03 | 1.01 (0.96-1.06) | 0.621 | 0.946 | 0.00 | 0.00 | 0.280 | 0.413 | - | - | - | 95.77 | 0.401 |
|  |  | MR Egger | -0.03 | 0.05 | 0.97 (0.88-1.07) | 0.492 | 0.986 |  |  |  |  |  |  |  |  |  |
|  |  | Weighted median | 0.02 | 0.05 | 1.02 (0.93-1.12) | 0.646 | 0.821 |  |  |  |  |  |  |  |  |  |
| *Bifidobacteriaceae* | 101 | IVW | 0.00 | 0.02 | 1.00 (0.96-1.04) | 0.988 | 0.946 | -0.01 | 0.00 | 0.088 | 0.019 | rs7901695 | 0.98 (0.94-1.02) | 0.286 | 131.30 | 0.020 |
|  |  | MR Egger | 0.06 | 0.04 | 1.06 (0.98-1.15) | 0.142 | 0.986 |  |  |  |  |  | 1.00 (0.91-1.10) | 0.986 |  |  |
|  |  | Weighted median | 0.02 | 0.03 | 1.02 (0.96-1.09) | 0.437 | 0.821 |  |  |  |  |  | 0.98 (0.92-1.04) | 0.445 |  |  |
| *Christensenellaceae* | 101 | IVW | 0.01 | 0.02 | 1.01 (0.98-1.05) | 0.412 | 0.946 | 0.00 | 0.00 | 0.862 | 0.532 | - | - | - | 99.28 | 0.502 |
|  |  | MR Egger | 0.01 | 0.03 | 1.01 (0.94-1.08) | 0.793 | 0.986 |  |  |  |  |  |  |  |  |  |
|  |  | Weighted median | 0.01 | 0.03 | 1.01 (0.95- 1.06) | 0.832 | 0.832 |  |  |  |  |  |  |  |  |  |
| *Clostridiaceae_1* | 98 | IVW | 0.00 | 0.02 | 1.00 (0.97-1.04) | 0.800 | 0.946 | 0.00 | 0.00 | 0.928 | 0.251 | - | - | - | 106.43 | 0.241 |
|  |  | MR Egger | 0.00 | 0.04 | 1.00 (0.93-1.08) | 0.961 | 0.986 |  |  |  |  |  |  |  |  |  |
|  |  | Weighted median | 0.03 | 0.03 | 1.03 (0.97-1.09) | 0.349 | 0.821 |  |  |  |  |  |  |  |  |  |
| *Coriobacteriaceae* | 102 | IVW | 0.00 | 0.02 | 1.00 (0.97-1.04) | 0.962 | 0.962 | 0.00 | 0.00 | 0.422 | 0.160 | - | - | - | 116.98 | 0.132 |
|  |  | MR Egger | 0.03 | 0.04 | 1.03 (0.96-1.10) | 0.473 | 0.986 |  |  |  |  |  |  |  |  |  |
|  |  | Weighted median | 0.01 | 0.02 | 1.01 (0.96-1.06) | 0.675 | 0.821 |  |  |  |  |  |  |  |  |  |
| *Defluviitaleaceae* | 94 | IVW | 0.01 | 0.02 | 1.01 (0.97-1.06) | 0.607 | 0.946 | 0.00 | 0.00 | 0.615 | 0.645 | - | - | - | 87.78 | 0.633 |
|  |  | MR Egger | -0.01 | 0.05 | 0.99 (0.90-1.09) | 0.861 | 0.986 |  |  |  |  |  |  |  |  |  |
|  |  | Weighted median | 0.05 | 0.04 | 1.05 (0.97-1.13) | 0.246 | 0.821 |  |  |  |  |  |  |  |  |  |
| *Desulfovibrionaceae* | 99 | IVW | 0.02 | 0.02 | 1.02 (0.99-1.06) | 0.235 | 0.946 | 0.00 | 0.00 | 0.730 | 0.534 | - | - | - | 96.37 | 0.528 |
|  |  | MR Egger | 0.03 | 0.04 | 1.03 (0.96-1.11) | 0.372 | 0.986 |  |  |  |  |  |  |  |  |  |
|  |  | Weighted median | 0.01 | 0.03 | 1.01 (0.95-1.07) | 0.807 | 0.832 |  |  |  |  |  |  |  |  |  |
| *Enterobacteriaceae* | 99 | IVW | 0.00 | 0.02 | 1.00 (0.96-1.05) | 0.844 | 0.946 | 0.00 | 0.00 | 0.617 | 0.072 | - | - | - | 120.11 | 0.064 |
|  |  | MR Egger | 0.02 | 0.04 | 1.02 (0.94-1.11) | 0.595 | 0.986 |  |  |  |  |  |  |  |  |  |
|  |  | Weighted median | 0.02 | 0.03 | 1.02 (0.96-1.09) | 0.425 | 0.821 |  |  |  |  |  |  |  |  |  |
| *Erysipelotrichaceae* | 102 | IVW | -0.02 | 0.02 | 0.98 (0.94-1.02) | 0.254 | 0.946 | 0.00 | 0.00 | 0.588 | 0.053 | - | - | - | 126.20 | 0.046 |
|  |  | MR Egger | 0.00 | 0.04 | 1.00 (0.93-1.07) | 0.917 | 0.986 |  |  |  |  |  |  |  |  |  |
|  |  | Weighted median | -0.02 | 0.03 | 0.98 (0.93-1.03) | 0.432 | 0.821 |  |  |  |  |  |  |  |  |  |
| *Lachnospiraceae* | 102 | IVW | 0.00 | 0.02 | 1.00 (0.97-1.04) | 0.792 | 0.946 | 0.00 | 0.00 | 0.901 | 0.381 | - | - | - | 104.20 | 0.394 |
|  |  | MR Egger | 0.00 | 0.03 | 1.00 (0.94-1.07) | 0.981 | 0.986 |  |  |  |  |  |  |  |  |  |
|  |  | Weighted median | 0.01 | 0.03 | 1.01 (0.95-1.07) | 0.790 | 0.832 |  |  |  |  |  |  |  |  |  |
| *Lactobacillaceae* | 94 | IVW | -0.01 | 0.03 | 0.99 (0.94-1.05) | 0.777 | 0.946 | -0.01 | 0.00 | 0.117 | 0.080 | - | - | - | 110.55 | 0.103 |
|  |  | MR Egger | 0.07 | 0.06 | 1.07 (0.96-1.20) | 0.226 | 0.986 |  |  |  |  |  |  |  |  |  |
|  |  | Weighted median | 0.01 | 0.04 | 1.01 (0.93-1.10) | 0.762 | 0.832 |  |  |  |  |  |  |  |  |  |
| *Methanobacteriaceae* | 92 | IVW | 0.05 | 0.04 | 1.05 (0.97-1.12) | 0.221 | 0.946 | 0.01 | 0.01 | 0.126 | 0.643 | - | - | - | 85.35 | 0.647 |
|  |  | MR Egger | -0.05 | 0.07 | 0.95 (0.82-1.09) | 0.481 | 0.986 |  |  |  |  |  |  |  |  |  |
|  |  | Weighted median | -0.07 | 0.06 | 0.94 (0.83-1.05) | 0.263 | 0.821 |  |  |  |  |  |  |  |  |  |
| *Oxalobacteraceae* | 94 | IVW | -0.07 | 0.03 | 0.94 (0.88-0.99) | 0.036 | 0.946 | 0.00 | 0.00 | 0.649 | 0.378 | - | - | - | 97.76 | 0.347 |
|  |  | MR Egger | -0.09 | 0.06 | 0.91 (0.81-1.03) | 0.149 | 0.986 |  |  |  |  |  |  |  |  |  |
|  |  | Weighted median | -0.07 | 0.05 | 0.93 (0.84-1.03) | 0.152 | 0.821 |  |  |  |  |  |  |  |  |  |
| *Pasteurellaceae* | 95 | IVW | 0.01 | 0.03 | 1.01 (0.96-1.07) | 0.703 | 0.946 | 0.01 | 0.00 | 0.057 | 0.008^a^ | - | - | - | 131.21 | 0.007 |
|  |  | MR Egger | -0.08 | 0.05 | 0.93 (0.83-1.03) | 0.145 | 0.986 |  |  |  |  |  |  |  |  |  |
|  |  | Weighted median | -0.03 | 0.04 | 0.97 (0.90-1.05) | 0.515 | 0.821 |  |  |  |  |  |  |  |  |  |
| *Peptococcaceae* | 95 | IVW | 0.00 | 0.02 | 1.00 (0.95-1.04) | 0.845 | 0.946 | 0.00 | 0.00 | 0.762 | 0.181 | - | - | - | 106.57 | 0.177 |
|  |  | MR Egger | -0.02 | 0.05 | 0.98 (0.90-1.08) | 0.720 | 0.986 |  |  |  |  |  |  |  |  |  |
|  |  | Weighted median | -0.04 | 0.03 | 0.96 (0.90-1.03) | 0.285 | 0.821 |  |  |  |  |  |  |  |  |  |
| *Peptostreptococcaceae* | 101 | IVW | 0.00 | 0.02 | 1.00 (0.96-1.04) | 0.953 | 0.962 | 0.00 | 0.00 | 0.582 | 0.009 | rs7656416 | 1.00 (0.96-1.04) | 0.935 | 138.20 | 0.007 |
|  |  | MR Egger | 0.02 | 0.04 | 1.02 (0.94-1.11) | 0.614 | 0.986 |  |  |  |  |  | 1.02 (0.94-1.10) | 0.669 |  |  |
|  |  | Weighted median | 0.01 | 0.03 | 1.01 (0.95-1.07) | 0.682 | 0.821 |  |  |  |  |  | 1.02 (0.94-1.10) | 0.379 |  |  |
| *Porphyromonadaceae* | 102 | IVW | 0.00 | 0.02 | 1.00 (0.96-1.03) | 0.792 | 0.946 | 0.00 | 0.00 | 0.743 | 0.473 | - | - | - | 101.41 | 0.470 |
|  |  | MR Egger | -0.01 | 0.03 | 0.99 (0.92-1.05) | 0.678 | 0.986 |  |  |  |  |  |  |  |  |  |
|  |  | Weighted median | -0.02 | 0.03 | 0.98 (0.93-1.03) | 0.426 | 0.821 |  |  |  |  |  |  |  |  |  |
| *Prevotellaceae* | 101 | IVW | -0.02 | 0.02 | 0.98 (0.94-1.01) | 0.224 | 0.946 | 0.00 | 0.00 | 0.383 | 0.631 | - | - | - | 95.86 | 0.599 |
|  |  | MR Egger | -0.05 | 0.04 | 0.95 (0.89-1.02) | 0.174 | 0.986 |  |  |  |  |  |  |  |  |  |
|  |  | Weighted median | -0.01 | 0.03 | 0.99 (0.93-1.04) | 0.628 | 0.821 |  |  |  |  |  |  |  |  |  |
| *Rhodospirillaceae* | 94 | IVW | 0.01 | 0.03 | 1.01 (0.96-1.06) | 0.731 | 0.946 | 0.00 | 0.00 | 0.286 | 0.067 | - | - | - | 114.94 | 0.061 |
|  |  | MR Egger | 0.06 | 0.05 | 1.06 (0.96-1.18) | 0.275 | 0.986 |  |  |  |  |  |  |  |  |  |
|  |  | Weighted median | 0.02 | 0.04 | 1.02 (0.95-1.11) | 0.574 | 0.821 |  |  |  |  |  |  |  |  |  |
| *Rikenellaceae* | 102 | IVW | 0.00 | 0.02 | 1.00 (0.97-1.04) | 0.832 | 0.946 | 0.00 | 0.00 | 0.595 | 0.846 | - | - | - | 86.37 | 0.850 |
|  |  | MR Egger | 0.02 | 0.03 | 1.02 (0.95-1.09) | 0.572 | 0.986 |  |  |  |  |  |  |  |  |  |
|  |  | Weighted median | -0.03 | 0.03 | 0.97 (0.92-1.03) | 0.315 | 0.821 |  |  |  |  |  |  |  |  |  |
| *Ruminococcaceae* | 102 | IVW | 0.01 | 0.02 | 1.01 (0.97-1.04) | 0.744 | 0.946 | 0.00 | 0.00 | 0.993 | 0.384 | - | - | - | 105.61 | 0.357 |
|  |  | MR Egger | 0.01 | 0.03 | 1.01 (0.94-1.07) | 0.876 | 0.986 |  |  |  |  |  |  |  |  |  |
|  |  | Weighted median | 0.01 | 0.03 | 1.01 (0.96-1.07) | 0.609 | 0.821 |  |  |  |  |  |  |  |  |  |
| *Streptococcaceae* | 101 | IVW | 0.00 | 0.02 | 1.00 (0.97-1.04) | 0.942 | 0.962 | 0.00 | 0.00 | 0.367 | 0.185 | - | - | - | 111.93 | 0.195 |
|  |  | MR Egger | 0.03 | 0.04 | 1.03 (0.96-1.11) | 0.415 | 0.986 |  |  |  |  |  |  |  |  |  |
|  |  | Weighted median | 0.04 | 0.03 | 1.04 (0.98-1.10) | 0.206 | 0.821 |  |  |  |  |  |  |  |  |  |
| *Veillonellaceae* | 102 | IVW | 0.00 | 0.02 | 0.99 (0.96-1.02) | 0.571 | 0.946 | 0.00 | 0.00 | 0.509 | 0.637 | - | - | - | 95.86 | 0.626 |
|  |  | MR Egger | 0.01 | 0.03 | 1.01 (0.94-1.08) | 0.774 | 0.986 |  |  |  |  |  |  |  |  |  |
|  |  | Weighted median | 0.01 | 0.03 | 1.01 (0.95-1.06) | 0.824 | 0.832 |  |  |  |  |  |  |  |  |  |
| *Verrucomicrobiaceae* | 95 | IVW | 0.02 | 0.02 | 1.02 (0.98-1.06) | 0.454 | 0.946 | 0.00 | 0.00 | 0.185 | 0.717 | - | - | - | 84.53 | 0.747 |
|  |  | MR Egger | 0.06 | 0.04 | 1.06 (0.98-1.15) | 0.129 | 0.986 |  |  |  |  |  |  |  |  |  |
|  |  | Weighted median | 0.03 | 0.03 | 1.06 (0.98-1.15) | 0.442 | 0.821 |  |  |  |  |  |  |  |  |  |

a. MR-PRESSO could not identify significant outliers

Abbreviations: MR, Mendelian randomization; SNP, single nucleotide polymorphism; IVW, inverse variance weighted; IVs, instrumental variables; FDR, false discovery rate; T2DM, type 2 diabetes mellitus; OR, odds ratio; MR-PRESSO, Mendelian randomization pleiotropy residual sum and outlier.
